# Vitamin and Mineral Supplementation and Rate of Weight Gain during the First Trimester of Gestation in Beef Heifers Alters the Fetal Liver Amino Acid, Carbohydrate, and Energy Profile at Day 83 of Gestation

**DOI:** 10.3390/metabo12080696

**Published:** 2022-07-27

**Authors:** Matthew S. Crouse, Kacie L. McCarthy, Ana Clara B. Menezes, Cierrah J. Kassetas, Friederike Baumgaertner, James D. Kirsch, Sheri Dorsam, Tammi L. Neville, Alison K. Ward, Pawel P. Borowicz, Lawrence P. Reynolds, Kevin K. Sedivec, J. Chris Forcherio, Ronald Scott, Joel S. Caton, Carl R. Dahlen

**Affiliations:** 1 United States Department of Agriculture, Agriculture Research Service, U.S. Meat Animal Research Center, Clay Center, NE 68933, USA; 2 Department of Animal Sciences, University of Nebraska Lincoln, Lincoln, NE 68588, USA; kacie.mccarthy@unl.edu; 3 Department of Animal Sciences, Center for Nutrition and Pregnancy, North Dakota State University, Fargo, ND 58108, USA; anaclara.menezes@ndsu.edu (A.C.B.M.); cierrah.kassetas@ndsu.edu (C.J.K.); friederike.baumgrtne@ndsu.edu (F.B.); james.kirsch@ndsu.edu (J.D.K.); sheri.dorsam@ndsu.edu (S.D.); tammi.neville@ndsu.edu (T.L.N.); alison.ward@ndsu.edu (A.K.W.); pawel.borowicz@ndsu.edu (P.P.B.); larry.reynolds@ndsu.edu (L.P.R.); joel.caton@ndsu.edu (J.S.C.); carl.dahlen@ndsu.edu (C.R.D.); 4 Central Grasslands Research Extension Center, North Dakota State University, Streeter, ND 58483, USA; kevin.sedivec@ndsu.edu; 5 Purina Animal Nutrition LLC, Grays Summit, MO 63039, USA; jcforcherio@landolakes.com (J.C.F.); rrscott@landolakes.com (R.S.)

**Keywords:** early gestation, fetal liver, metabolome, mineral, rate of weight gain, vitamin

## Abstract

The objective of this study was to evaluate the effects of feeding heifers a vitamin and mineral supplement and targeting divergent rates of weight gain during early gestation on the fetal liver amino acid, carbohydrate, and energy profile at d 83 of gestation. Seventy-two crossbred Angus heifers were randomly assigned in a 2 × 2 factorial arrangement to one of four treatments comprising the main effects of vitamin and mineral supplementation (VTM or NOVTM) and feeding to achieve different rates of weight gain (low gain [LG] 0.28 kg/day vs. moderate gain [MG] 0.79 kg/day). Thirty-five gestating heifers with female fetuses were ovariohysterectomized on d 83 of gestation and fetal liver was collected and analyzed by reverse phase UPLC-tandem mass spectrometry with positive and negative ion mode electrospray ionization, as well as by hydrophilic interaction liquid chromatography UPLC-MS/MS with negative ion mode ESI for compounds of known identity. The Glycine, Serine, and Threonine metabolism pathway and the Leucine, Isoleucine, and Valine metabolism pathway had a greater total metabolite abundance in the liver of the NOVTM-LG group and least in the VTM-LG group (*p* < 0.01). Finally, both the TCA Cycle and Oxidative Phosphorylation pathways within the Energy Metabolism superpathway were differentially affected by the main effect of VTM, where the TCA cycle metabolites were greater (*p* = 0.04) in the NOVTM fetal livers and the Oxidative Phosphorylation biochemicals were greater (*p* = 0.02) in the fetal livers of the VTM supplemented heifers. These data demonstrate that the majority of metabolites that are affected by rate of weight gain or vitamin/mineral supplementation are decreased in heifers on a greater rate of weight gain or vitamin/mineral supplementation.

## 1. Introduction

Amino acids, carbohydrates, and energy metabolites are important in supporting fetal and placental growth and are key energy sources, proteinogenic compounds, osmoregulators of fetal fluids, maternal-embryo signals, and modulators of gene expression during development [1,2,3]. Similarly, minerals and vitamins support fetal growth through hormone production, as cofactors in enzyme and metabolic reactions, and for tissue synthesis, oxygen transport, vascular development and energy production [4,5].

Angus-cross heifers are expected to be pubertal at 60% of mature body weight [5]. Multiple heifer development strategies, such as a lighter body weight at breeding [6,7,8], a stair-step nutritional program [9,10,11,12,13] or a constant high rate of weight gain or overnourishment [9,14] have been employed to decrease age at puberty, increase ovarian antral follicle counts, and evaluate the effects of heifer development on lifetime productivity. There are limited data on the effects of post-breeding rate of weight gain on fetal development and postnatal performance with focuses on immediate fertility [15,16] or fetal metabolites in maternal and fetal fluids or the fetal transcriptome [3,17].

The most recent National Animal Health Monitoring System [18] reported that 51.7% of producers fed a trace mineral salt and 49.7% of producers fed a complete mineral supplement to their cows during the spring/summer (April through September), which is when the majority of breeding occurs in the Great Plains region. Furthermore, Davy et al. [19] reported that only one-half of California’s breeding cows and heifers were supplemented with mineral, which resulted in deficiencies of several trace minerals such as Cu, Zn, Se, and Mn. Minerals and vitamins are critical to normal metabolism and growth, playing important roles in bone and tissue formation, as cofactors of enzymes, balancing charges in reactions, serving as osmoregulators, maintaining membrane potentials, and serving as electron acceptors and donors. Both minerals and vitamins are transferred efficiently to the fetus via the placenta, and are used for immediate metabolism or stored for postnatal reserves [20,21]. Thus, with roughly one-half of breeding cows receiving a complete or trace mineral supplement throughout the breeding season and early gestation, it is important to establish the immediate and long-term effects of supplementation on fetal development and calf performance.

A recent publication using data from the study in the current report Menezes et al. [22] showed differences in the concentrations of amino acids in serum, and allantoic and amniotic fluids of heifers at d 83 of gestation. These investigators reported increases in the concentrations of neutral amino acids in the allantoic fluid of vitamin and mineral supplemented heifers vs. those not supplemented. Those heifers on the moderate rate of weight gain had increased concentrations of cysteine, arginine, and anionic amino acids in allantoic fluid compared with those on the low rate of weight gain. Building upon these data to determine the concentrations in fetal liver would connect maternal and fetal metabolism and elucidate the interactive effects of heifer rate of weight gain and vitamin/mineral supplementation on immediate fetal metabolism *in utero*.

Therefore, the objectives of this study were to measure the effects of the Angus-cross heifers’ rate of weight gain and maternal vitamin/mineral supplementation on the fetal hepatic amino acid, carbohydrate, and energy metabolomes at d 83 of gestation. We hypothesized that vitamin and mineral supplementation and moderate rates of weight gain would positively impact fetal metabolism as evidenced by altered abundance of metabolites involved in energetic pathways.

## 2. Materials and Methods

All animal procedures were approved by the North Dakota State University Institutional Animal Care and Use Committee (#A19012).

### 2.1. Animals, Experimental Design, and Dietary Treatments

Complete detail describing animals, experimental design and treatments have been previously reported by Menezes et al. [22,23]. Briefly, *Bos taurus* heifers (Angus; *n* = 72; initial BW = 359.5 ± 7.1 kg) were randomly assigned to 1 of 4 treatments in a 2 × 2 factorial arrangement, with the main effects of vitamin and mineral supplementation (supplemented [**VTM**] vs. not supplemented [**NOVTM**]) and rate of weight gain (low gain [**LG**], 0.28 kg/day vs. moderate gain [**MG**], 0.79 kg/day).

Heifers were managed pre-study so they were adapted to an indoor facility, stratified by BW, and randomly assigned to receive a vitamin and mineral supplement (VTM; *n* = 36) or not (NOVTM; *n* = 36) for a controlled pre-breeding depletion period. The VTM factor was initiated prior to breeding to ensure adequate vitamin and mineral reserves at breeding (VTM) or to deplete vitamin and mineral reserves (NOVTM). Heifers were individually fed daily in an electronic head gate facility (American Calan; Northwood, NH, USA). Diets were delivered once daily via a total mixed ration (**TMR**) and consisted of triticale hay, corn silage, modified distillers grains plus solubles, ground corn, and if indicated by treatment, VTM premix. The VTM premix was fed at a 0.45 kg/heifer/day, provided macro and trace minerals and vitamins A, D, and E to meet 110% of the requirements specified by NASEM for the heifer’s physiological state and body size [5], and consisted of ground corn and a loose VTM supplement (113 g of Purina Wind & Rain Storm All-Season 7.5 Complete, Land O’Lakes, Inc., Arden Hills, MN, USA; and 337 g of a carrier). All heifers were subjected to the 7-day CO-Synch + CIDR estrus synchronization protocol [24], bred via artificial insemination with female sexed semen from a single sire. At breeding, heifers were randomly assigned to either LG or MG treatments within their respective VTM treatment, thus completing the 2 × 2 factorial (VTM-LG, VTM-MG, NOVTM-LG, NOVTM-MG). Heifers on the LG treatment were maintained on the basal TMR, and to achieve the requirements for MG, heifers were supplemented with a blend of ground corn, dried distillers grains plus solubles, wheat midds, fish oil, urea, and ethoxyquin top dressed on the TMR at 0.58% of BW as-fed daily. Heifers were weighed weekly and feed intake was adjusted during the study to achieve the targeted BW gains. Fetal sex was confirmed via transrectal ultrasonography at 65 d of gestation. Of the original 72 heifers bred, 35 were pregnant with female fetuses in the following treatment combinations: NOVTM-LG (*n* = 9), NOVTM-MG (*n* = 9), VTM-LG (*n* = 9), and VTM-MG (*n* = 8). Heifers received treatment diets until d 83 ± 0.27 of gestation, at which time they were ovariohysterectomized as previously described by McLean et al. [25].

### 2.2. Sample Collection

After ovariohysterectomy, the fetus was removed from the uterine body and fetal liver was weighed, collected, and frozen in aliquots on dry ice. Fifty milligrams of tissue was shipped frozen to Metabolon Inc. (Morrisville, NC, USA) and maintained at −80 °C until processed.

### 2.3. Sample Preparation, UPLC-MS/MS, and QC

Several recovery standards were added prior to the first step in the extraction process for quality control (**QC**) purposes. Liver samples were prepared using the automated MicroLab STAR^®^ system (Hamilton Company, Reno, NV, USA), precipitated with methanol with vigorous shaking for 2 min (GenoGrinder 200, Glen Mills, Clifton, NJ, USA) to remove protein, dissociate small molecules bound to protein or trapped in the precipitated protein matrix, and finally recover chemically diverse metabolites after centrifugation. The resulting extract was divided into five fractions: two for analysis by two separate reverse phase (**RP**)/UPLC-tandem mass spectrometry (**MS/MS**) methods with positive ion mode electrospray ionization (**ESI**), one for analysis by RP/UPLC-MS/MS with negative ion mode ESI, one for analysis by hydrophilic interaction liquid chromatography (**HILIC**)/UPLC-MS/MS with negative ion mode ESI, and one sample reserved for backup. Samples were placed briefly on a TurboVap^®^ (Zymark, Hopkinton, MA, USA) to remove the organic solvent and the sample extracts stored overnight under nitrogen.

All methods utilized Waters ACQUITY ultra-performance liquid chromatography (**UPLC**) and a Thermo Scientific Q-Exactive high resolution/accurate mass spectrometer interfaced with a heated electrospray ionization (HESI-II) source and Orbitrap mass analyzer operated at 35,000 mass resolution. The sample extract was dried then reconstituted in solvents compatible with each of the four methods. Each reconstitution solvent contained a series of standards at fixed concentrations to ensure injection and chromatographic consistency. One aliquot was analyzed using acidic positive ion conditions, chromatographically optimized for more hydrophilic compounds. In this method, the extract was gradient eluted from a C18 column (Waters UPLC BEH C18−2.1 × 100 mm, 1.7 µm) using water and methanol, containing 0.05% perfluoropentanoic acid (**PFPA**) and 0.1% formic acid (**FA**). Another aliquot was also analyzed using acidic positive ion conditions; however, it was chromatographically optimized for more hydrophobic compounds. In this method, the extract was gradient eluted from the same aforementioned C18 column using methanol, acetonitrile, water, 0.05% PFPA and 0.01% FA and was operated at an overall higher organic content. Another aliquot was analyzed using basic negative ion optimized conditions using a separate dedicated C18 column. The basic extracts were gradient eluted from the column using methanol and water, with 6.5 mM Ammonium Bicarbonate at pH 8. The fourth aliquot was analyzed via negative ionization following elution from a HILIC column (Waters UPLC BEH Amide 2.1 × 150 mm, 1.7 µm) using a gradient consisting of water and acetonitrile with 10-mM Ammonium Formate, pH 10.8. The MS analysis alternated between MS and data-dependent sequential mass spectrometry scans using dynamic exclusion. The scan range varied slighted between methods but covered a mass to charge ratio of 70–1000 *m*/*z*. Instrument variability was determined by calculating the median relative standard deviation (**RSD**) for the standards that were added to each sample before injection into the mass spectrometers. Overall process variability was determined by calculating the median RSD for all endogenous metabolites (i.e., non-instrument standards) present in 100% of the pooled matrix samples. Experimental samples were randomized across the platform run with QC samples spaced evenly among the injections. The total mean instrument variability for internal standards was 3% and the total process variability for endogenous biochemicals/metabolites was 7%.

### 2.4. Data Extraction and Compound Identification

Raw data were extracted, peak-identified and QC processed using Metabolon’s hardware and software. The informatics system consisted of four major components: the Laboratory Information Management System (**LIMS**), the data extraction and peak-identification software, data processing tools for QC and compound identification, and a collection of information interpretation and visualization tools for use by data analysts. Compounds were identified by comparison to library entries of purified standards or recurrent unknown entities. Metabolon maintains a library based on authenticated standards that contains the retention time/index (**RI**), *m*/*z*, and chromatographic data (including MS/MS spectral data) on all molecules present in the library. Furthermore, biochemical identifications are based on three criteria: retention index within a narrow RI window of the proposed identification, accurate mass match to the library +/− 10 ppm, and the MS/MS forward and reverse scores between the experimental data and authentic standards. Additional mass spectral entries have been created for structurally unnamed biochemicals, which have been identified by virtue of their recurrent nature (both chromatographic and mass spectral). These compounds have the potential to be identified by future acquisition of a matching purified standard or by classical structural analysis.

### 2.5. Statistical Analysis

Biochemical/metabolite data were log transformed, adjusted for fetal liver weight, and analyzed by two-way ANOVA for main effects of vitamin/mineral supplementation, rate of weight gain, and their interaction. Furthermore, contrasts between treatments were conducted by Welch’s two-sample *t*-test. All *p*-values less than or equal to 0.05 were considered significant, with tendencies being considered as greater than 0.05 but less than 0.10. Pathway enrichment was calculated within the MetaboLync Pathway Analysis software using the following formula: $\left(\frac{k}{m}\right)/\left(\frac{n}{N}\right)$ where *k* = the number of significant metabolites per pathway, *m* = the total number of detected metabolites per pathway, *n* = the number of significant metabolites in the study, and *N* = the total number of detected metabolites in the study, as previously described by Simintiras et al. [26]. Pathways with enrichment scores >1 have more metabolites with statistically significant fold changes compared to all other pathways within the study allowing for highlighting specific pathways of importance that are affected by heifer weight gain, vitamin/mineral supplementation, or their interaction.

Total pathway analysis was conducted by averaging relative metabolite abundances within a super-pathway and sub-pathways and analyzed with the MIXED procedure of SAS [27] with fixed effects of gain (LG vs. MG) and vitamin/mineral (VTM vs. NOVTM) as well as the gain × vitamin/mineral supplement interaction, along with the random effect of heifer to determine super- and sub-pathway directionality and effects of treatment on fetal liver metabolism.

## 3. Results

The complete data including *p*-values, fold changes, mean values, and percent filled values for all results in this manuscript can be found in the Supplementary Table S1 File. Box plots for all biochemicals are included in the Supplementary Figure S1: Supplementary Figures Box Plots file. For brevity, not all tendencies are discussed.

In total, 305 biochemicals/metabolites were consistently identified relating to amino acid and peptide metabolism (*n* = 242), carbohydrate metabolism (*n* = 52), and energy metabolism (*n* = 11). Of these biochemicals, 16 were influenced by a gain × vitamin/mineral interaction (*p* ≤ 0.05) and 10 tended to be influenced by a gain × vitamin/mineral interaction (0.05 < *p* ≤ 0.10). There were 27 biochemicals that were influenced by a main effect of rate of weight gain (*p* ≤ 0.05) and 28 tended to be influenced by rate of weight gain (0.05 < *p* ≤ 0.10). Finally, there were 15 biochemicals that were influenced by a main effect of vitamin/mineral supplementation (*p* ≤ 0.05) and 18 that tended to be influenced by vitamin/mineral supplementation (0.05 < *p* ≤ 0.10).

### 3.1. Specific Interactive and Main Effects

#### 3.1.1. Amino Acid Metabolism

Of the 15 amino acid pathways, the pathways that were enriched for the gain × vitamin/mineral interaction were Glycine, Serine and Threonine Metabolism (5.62), followed by Glutamate Metabolism (3.81); Urea Cycle: Arginine and Proline Metabolism (2.59); Leucine, Isoleucine, and Valine Metabolism (1.80); Tyrosine Metabolism (1.32); and Methionine, Cysteine, *S*-adenosyl methionine (**SAM**), and Taurine Metabolism (0.58; Table 1). The remaining pathways had an enrichment score of 0 for the gain × vitamin/mineral interaction.

Lastly, the enriched pathways for the main effect of vitamin/mineral supplementation were Glutamate Metabolism (4.53); Lysine Metabolism (3.25); Glycine, Serine, and Threonine Metabolism (1.56); Histidine Metabolism (0.88); and Methionine, Cysteine, SAM, and Taurine Metabolism (0.68). The remaining pathways had an enrichment score of 0 for the main effect of vitamin/mineral supplementation.

The enriched pathways for the main effect of gain were Alanine and Aspartate Metabolism (1.06); Histidine Metabolism (1.01); Glutamate Metabolism (0.65); Leucine, Isoleucine, and Valine Metabolism (0.62), Glutathione Metabolism (0.56), Lysine Metabolism (0.46); Urea Cycle: Arginine and Proline Metabolism (0.44); Tryptophan Metabolism (0.35); Glycine, Serine, and Threonine Metabolism (0.35); and Methionine, Cysteine, SAM, and Taurine Metabolism (0.31; Table 1). The remaining pathways had an enrichment score of 0 for the main effect of gain.

The significant biochemicals/metabolites in the Glycine, Serine, and Threonine Metabolism for the gain × vitamin/mineral interaction were Sarcosine, Dimethylglycine, Betaine, Serine, and Homoserine (Table 2; *p* ≤ 0.05). Dimethylglycine and Betaine abundance followed similar patterns, in which VTM-LG was nearly 1.5-fold lower than any other treatment. Serine abundance was influenced by a main effect of gain and was decreased in fetal liver from MG compared with LG heifers (*p* = 0.05).

The Alanine and Aspartate Metabolism pathway did not have any biochemicals/metabolites affected by the gain × vitamin/mineral interaction (*p* ≥ 0.22); however, aspartate and *N*-acetyl asparagine were both greater in fetal liver of LG compared with MG heifers (Table 2; *p* ≤ 0.02). Furthermore, aspartate tended (*p* = 0.08) to be less in VTM compared with NOVTM fetuses.

The significant biochemicals in the Glutamate Metabolism pathway for the gain × vitamin/mineral interaction were *N*-acetylglutamate, *N*-acetyl-aspartyl-glutamate, and carboxyethyl-GABA (Table 2; *p* ≤ 0.02). Additionally, 4-hydroxyglutamate, gammacarboxyglutamate, and *n*-methyl-GABA were not affected by a gain × vitamin/mineral interaction but were decreased in VTM supplemented compared with NOVTM heifers (*p* ≤ 0.05), and glutamate as well as 4-hydroxyglutamate were also greater in fetal liver from LG vs. MG heifers (Table 2; *p* = 0.02).

The Lysine Metabolism pathway did not have any biochemicals/metabolites affected by the gain × vitamin/mineral interaction (*p* ≥ 0.08). Saccharopine and pipecolate abundance were decreased (Table 2; *p* ≤ 0.03) in fetal livers from MG compared with LG heifers, and *N*6-methyllysine, *N*6,*N*6-dimethyllysine, and *N*,*N*,*N*,-trimethyl-5-aminovalerate were decreased (Table 2; *p* ≤ 0.03) in fetal livers from VTM compared with NOVTM heifers.

For the Tyrosine Metabolism pathway, only *O*-methyltyrosine was impacted by the gain × vitamin/mineral interaction (Table 2; *p* = 0.04). Furthermore, 3-methoxytyrosine was less (*p* = 0.01) in fetal liver from MG compared with LG heifers (Table 3).

The Histidine Metabolism pathway did not have any biochemicals/metabolites affected by the gain × vitamin/mineral interaction (*p* ≥ 0.11); however, 1-methylhistidine, hydantoin-5-propionate, imidazole propionate, formiminoglutamate, and 1-methyl-5-imidazoleacetate were all affected by a main effect of gain (Table 2; *p* ≤ 0.04). Lastly, histidine methyl ester was decreased (Table 2; *p* = 0.05) in fetal liver from VTM supplemented compared with NOVTM heifers.

The Tryptophan Metabolism pathway did not have any biochemicals/metabolites affected by the gain × vitamin/mineral interaction (*p* ≥ 0.13). Indolelactate was greater (Table 3; *p* < 0.01) in fetal liver from MG compared with LG heifers. Indoleacetylglycine and 3-indoxylsulfate were greater (Table 3; *p* ≤ 0.05) in fetal liver from NOVTM compared with VTM supplemented heifers.

The significant biochemicals/metabolites in the Leucine, Isoleucine, and Valine Metabolism pathway for the gain × vitamin/mineral interaction were *N*-acetylisoleucine, 2-methylbutyrilcarnitine, and tiglylcarnitine (Table 3; *p* ≤ 0.03). Abundance of 1-carboxyethylleucine, ethylmalonate, valine, and alpha-hydroxyisovalerate were decreased (Table 3; *p* ≤ 0.03) in fetal liver of MG compared with LG heifers, and leucine tended (Table 3; *p* = 0.08) to be less in MG compared with LG fetal livers.

In the Methionine, Cysteine, SAM, and Taurine Metabolism pathway, only taurine was affected by the gain × vitamin/mineral interaction, being decreased (Table 3; *p* = 0.04) in the VTM-LG fetuses compared with all other treatments. Methionine sulfone was altered by the main effects of gain and vitamin/mineral supplementation (Table 3; *p* ≤ 0.04), being decreased in fetal livers of MG compared with LG and VTM compared with NOVTM heifers. Furthermore, hypotaurine was increased (Table 3; *p* = 0.01) in fetal livers of MG compared with LG heifers.

The significant biochemicals/metabolites in the Urea Cycle: Arginine and Proline Metabolism pathway impacted by the gain × vitamin/mineral interaction were citrulline, homoarginine, and prolylhydroxyproline (Table 4; *p* ≤ 0.05). Homocitrulline and *N*-methylproline were not affected by a gain × vitamin/mineral interaction but were increased in fetal liver from MG compared with LG heifers (Table 4; *p* < 0.01). Furthermore, urea and ornithine tended to be affected by main effects of gain and vitamin/mineral, respectively, with urea tending (Table 4; *p* = 0.06) to be greater in fetal liver from MG compared with LG heifers and ornithine tending (Table 4; *p* = 0.07) to be greater in fetal liver of NOVTM compared with VTM supplemented heifers.

The Glutathione Metabolism pathway did not have any biochemicals/metabolites affected by the gain × vitamin/mineral interaction or the main effect of vitamin/mineral supplementation (*p* ≥ 0.23); however, 5-oxoproline and ophthalmate were greater in fetal liver of LG compared with MG heifers (Table 4; *p* ≤ 0.04).

There were no biochemicals/metabolites affected by the gain × vitamin/mineral interaction for Phenylalanine Metabolism, Creatine Metabolism, Polyamine Metabolism, or Guanidino and Acetamido Metabolism (Table 2 and Table 4; *p* ≥ 0.07). Furthermore, no biochemicals/metabolites were affected by the main effects of gain or vitamin/mineral supplementation for Phenylalanine Metabolism; Polyamine Metabolism; or Guanidino and Acetamido Metabolism (*p* ≥ 0.07); however, creatinine was greater (Table 2 and Table 4; *p* = 0.03) in fetal livers from LG compared with MG heifers.

#### 3.1.2. Carbohydrate Metabolism

Of the eight Carbohydrate Metabolism pathways, only the Fructose, Mannose, and Galactose Metabolism pathway was enriched for a gain × vitamin/mineral interaction (2.28; Table 1). Furthermore, only the Glycolysis, Gluconeogenesis, and Pyruvate Metabolism pathway (1.34) and Aminosugar Metabolism pathway (2.09) were enriched for the main effect of vitamin/mineral supplementation. No pathways were enriched for the main effect of gain.

Only three biochemicals in the Carbohydrate Metabolism superpathway were altered by an interaction or the main effects of gain or vitamin/mineral supplementation. Mannitol/Sorbitol were affected by the gain × vitamin/mineral interaction (Table 5; *p* = 0.02) where the VTM-LG fetal livers had decreased mannitol/sorbitol compared with all other treatments. Glucose-1-phosphate was increased (Table 5; *p* = 0.01) in fetal livers from VTM compared with NOVTM heifers; in contrast, *N*-acetylglucosamine/*N*-acetylgalactosamine was decreased (Table 5; *p* = 0.04) in fetal livers from VTM compared with NOVTM heifers.

#### 3.1.3. Energy Metabolism

Of the two pathways comprising the energy metabolism superpathway, neither the TCA Cycle nor Oxidative Phosphorylation pathways were enriched for the gain × vitamin/mineral interaction (Table 1). The TCA cycle pathway was enriched for both the main effects of gain (0.47) and vitamin/mineral supplementation (2.09; Table 1).

In the TCA cycle pathway, no biochemicals/metabolites were differentially abundant due to the gain × vitamin/mineral interaction (Table 6; *p* ≥ 0.10). Tricarballylate was greater (*p* = 0.02) in fetal liver from MG compared with LG heifers, and greater (*p* < 0.01) in fetal liver from NOVTM compared with VTM supplemented heifers (Table 6). Additionally, Fumarate tended (Table 6; *p* = 0.08) to be greater in fetal liver of NOVTM compared with VTM supplemented heifers.

In the Oxidative Phosphorylation pathway, no biochemicals/metabolites were differentially abundant due to the gain × vitamin/mineral interaction (Table 6; *p* = 0.08); however, acetylphosphate tended (Table 6; *p* = 0.08) to be greater in fetal liver of VTM-LG heifers compared with those from all other treatments.

### 3.2. Specific Interactive and Main Effects

#### 3.2.1. Amino Acids

**Figure 1:**
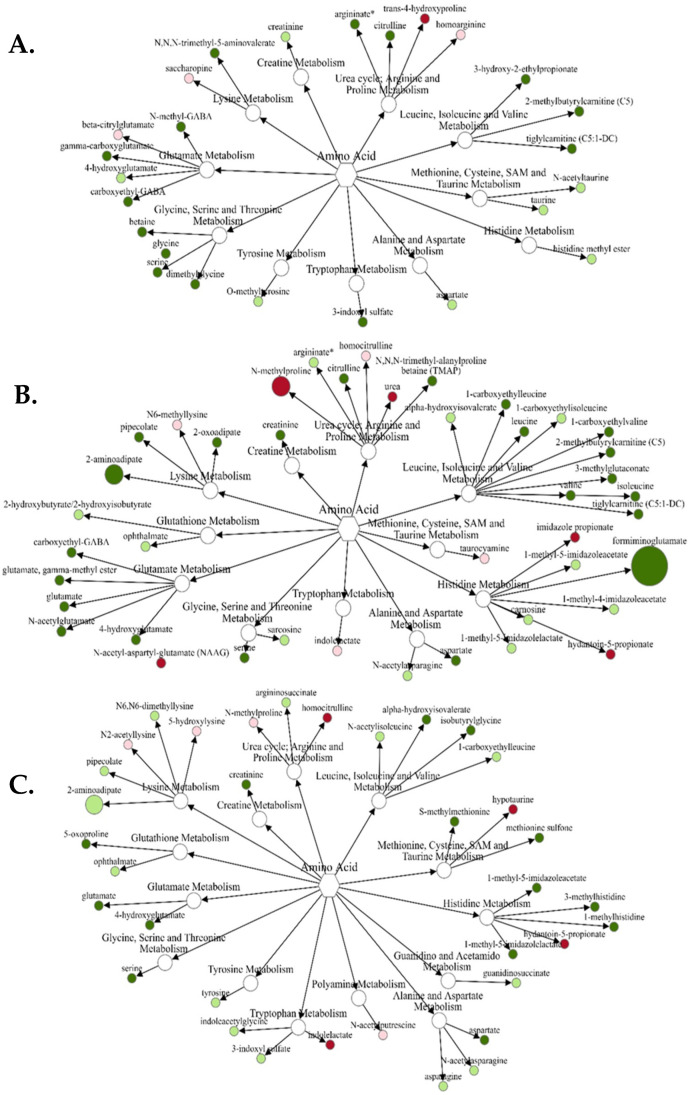
Network comparison of amino acid metabolites relative flux in fetal liver of VTM-LG vs. NOVTM-LG (**A**), NOVTM-MG vs. NOVTM-LG (**B**), and VTM-MG vs. NOVTM-LG (**C**). **VTM** = supplemented with vitamin/mineral. **NOVTM** = not supplemented with vitamin/mineral. **LG** = fed to gain 0.28 kg/day. **MG** = fed to gain 0.79 kg/day. Node diameter is proportional to the fold-change observed. Node color represented the significance of the change: dark red is an increase (*p* ≤ 0.05), light red is a tendency to increase (0.05 < *p* ≤ 0.10), dark green is a decrease (*p* ≤ 0.05), and light green is a tendency to decrease (0.05 < *p* ≤ 0.10). Only significant biochemicals are presented.

For the gain x vitamin/mineral interaction (Figure 1A), only trans 4-hydroxyproline was increased (*p* = 0.05) in VTM-LG compared with NOVTM-LG. Conversely, 14 amino acids and metabolites were decreased (*p* ≤ 0.05), with the greatest fold-change decrease being *N*-methyl GABA. Comparing NOVTM-MG with NOVTM-LG (Figure 1B), 5 amino acids and metabolites were increased (*p* ≤ 0.05) in NOVTM-MG compared with NOVTM-LG. The greatest fold change was for *N*-methylproline, which was 2.39-fold greater (*p* = 0.0084) in NOVTM-MG compared with NOVTM-LG. Compared to the previous contrast (*p* ≤ 0.05), there were 21 amino acids or metabolites decreased in NOVTM-MG compared with NOVTM-LG, with the greatest fold differences being formiminoglutamate (0.21-fold; *p* = 0.03) and 2-aminoadipate (0.45-fold; *p* = 0.05). Lastly, comparison of VTM-MG with NOVTM-LG (Figure 1C), 4 amino acids and metabolites were increased in abundance (*p* ≤ 0.05), with hypotaurine having the greatest (1.53-fold; *p* = 0.0043) increase in abundance. There were 21 amino acids and related metabolites that were decreased (*p* ≤ 0.05) in VTM-MG compared with NOVTM-LG; however, the greatest fold change decrease was for 2-aminoadipate, which tended to be decreased (0.47-fold; *p* = 0.07) in the VTM-MG group.

#### 3.2.2. Carbohydrates and Energy

**Figure 2:**
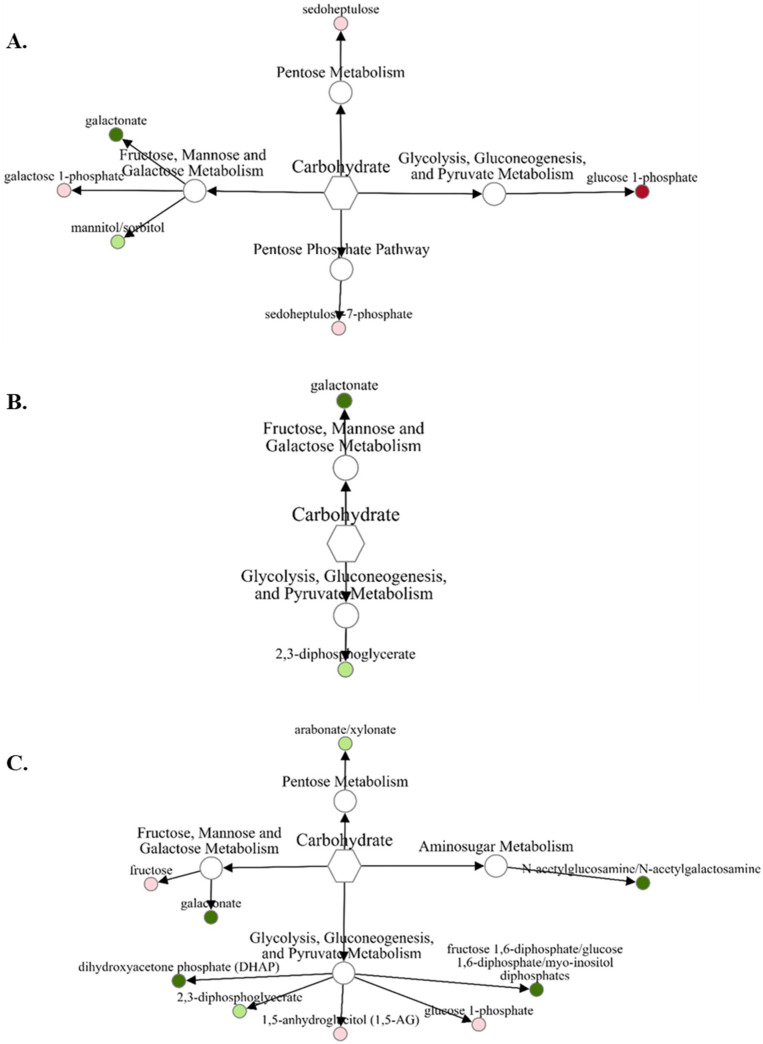
Network comparison of carbohydrate metabolites relative flux in fetal liver of VTM-LG vs. NOVTM-LG (**A**), NOVTM-MG vs. NOVTM-LG (**B**), and VTM-MG vs. NOVTM-LG (**C**). VTM = supplemented with vitamin/mineral. NOVTM = not supplemented with vitamin/mineral. LG = fed to gain 0.28 kg/day. MG = fed to gain 0.79 kg/day. Node diameter is proportional to the fold-change observed. Node color represented the significance of the change: dark red is an increase (*p* ≤ 0.05), light red is a tendency to increase (0.05 < *p* ≤ 0.10), dark green is a decrease (*p* ≤ 0.05), and light green is a tendency to decrease (0.05 < *p* ≤ 0.10). Only significant biochemicals are presented.

There were far fewer carbohydrate and energy metabolites that were differentially abundant in fetal liver compared with amino acids. Comparing abundances of carbohydrates and related metabolites in VTM-LG compared with NOVTM-LG (Figure 2A), only glucose-1-phosphate increased (1.49-fold; *p* = 0.02) and galactonate decreased (0.64-fold; *p* = 0.03) in abundance. For NOVTM-MG compared with NOVTM-LG (Figure 2B), only galactonate was decreased (0.65-fold; *p* = 0.04) in abundance. For VTM-MG compared with NOVTM-LG (Figure 2C), 4 carbohydrates and related metabolites were differentially abundant (*p* ≤ 0.05), with fructose 1,6-diphosphate/glucose 1,6-diphosphate being the least abundant (0.58-fold lower in VTM-MG; *p* = 0.02).

**Figure 3:**
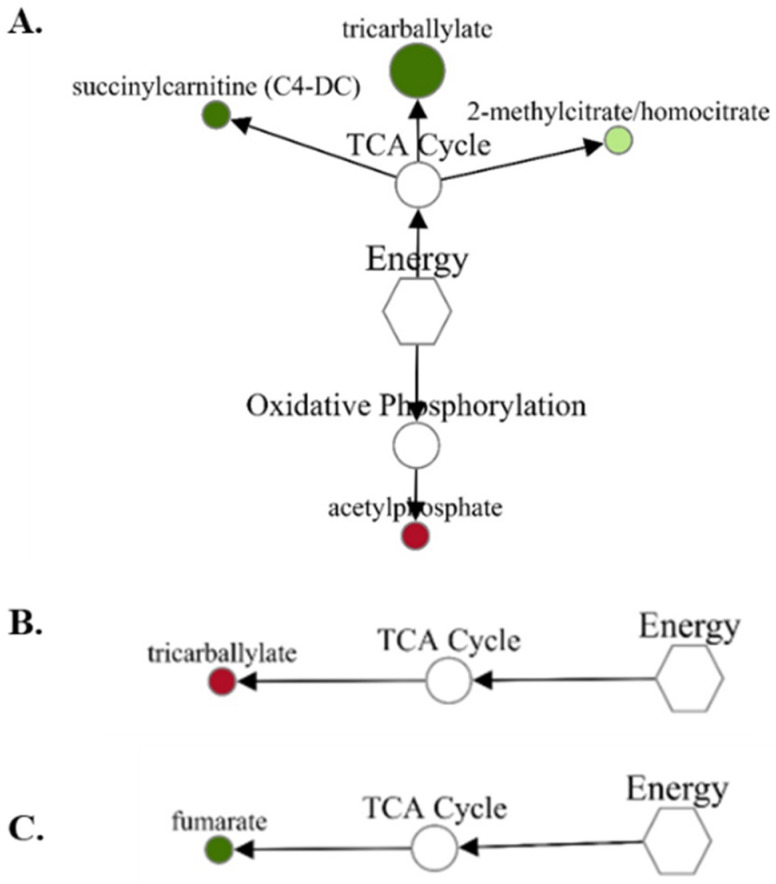
Network comparison of energy metabolites relative flux in fetal liver of VTM-LG vs. NOVTM-LG (**A**), NOVTM-MG vs. NOVTM-LG (**B**), and VTM-MG vs. NOVTM-LG (**C**). VTM = supplemented with vitamin/mineral. NOVTM = not supplemented with vitamin/mineral. LG = fed to gain 0.28 kg/day. MG = fed to gain 0.79 kg/day. Node diameter is proportional to the fold-change observed. Node color represented the significance of the change: dark red is an increase (*p* ≤ 0.05), light red is a tendency to increase (0.05 < *p* ≤ 0.10), dark green is a decrease (*p* ≤ 0.05), and light green is a tendency to decrease (0.05 < *p* ≤ 0.10). Only significant biochemicals are presented.

While the energy and related metabolites were made up of the fewest metabolites, the fold-change differences observed were some of the greatest changes between treatments. For the VTM-LG vs. NOVTM-LG comparison (Figure 3A), acetylphosphate was the only metabolite to increase (1.43-fold; *p* = 0.01) in abundance in VTM-LG compared with NOVTM-LG; in contrast, tricarballylate was decreased (0.49-fold less; *p* = 0.03) in VTM-LG compared with the NOVTM-LG fetal livers. Tricaballylate was increased (1.71-fold; *p* = 0.04) in the fetal livers of the NOVTM-MG compared with the NOVTM-LG heifers (Figure 3B). Lastly, fumarate was decreased (0.89-fold; *p* = 0.02) in the fetal livers of VTM-MG compared with those from the NOVTM-LG heifers (Figure 3C).

### 3.3. Total Pathway Analysis

#### 3.3.1. Amino Acids

Two subpathways, the Glycine, Serine, and Threonine Metabolism as well as the Leucine, Isoleucine, and Valine Metabolism pathways, were altered by the gain × vitamin/mineral interaction (*p* ≤ 0.02; Table 7). In the Glycine, Serine, and Threonine Metabolism pathway, the abundance of metabolites was greater (*p* < 0.01) in fetal livers from NOVTM-LG compared with the NOVTM-MG and VTM-LG heifers; furthermore, VTM-LG fetal livers were less than NOVTM-LG and VTM-MG.

In the Leucine, Isoleucine, and Valine subpathways, the concentration of metabolites was greatest (*p* = 0.02) in fetal livers of NOVTM-LG heifers compared with all other treatments. When evaluating all amino acid metabolites together, the total amino acid abundance tended (*p* = 0.07) to be greater in fetal livers of heifers on the NOVTM compared with those of the VTM treatments. Additionally, the abundance of metabolites in the Gaunidino and Acetamido subpathway tended (*p* = 0.09) to be greater in the fetal livers of the NOVTM compared with VTM supplemented heifers. Lastly, the relative abundances of Glutathione metabolites tended (*p* = 0.09) to be greater in the fetal livers of LG compared with MG heifers (Table 7).

#### 3.3.2. Carbohydrates and Energy

None of the Carbohydrate pathways (Table 7) were affected by the gain × vitamin/mineral interaction (*p* ≥ 0.11), or the main effects of gain (*p* ≥ 0.09) or vitamin (*p* ≥ 0.07). The relative abundance of metabolites in the Advanced Glycation End-Products subpathway tended (*p* = 0.09) to be greater in fetal livers of LG compared with MG heifers. Furthermore, metabolites in the Pentose Phosphate Pathway tended (*p* = 0.07) to be greater in fetal livers of VTM compared with NOVTM supplemented heifers (Table 7).

For the Energy pathways (Table 7), the Oxidative Phosphorylation subpathway tended (*p* = 0.08) to be greater in the VTM-LG fetal livers compared with all other treatments, with the total abundance being least in the NOVTM-LG fetal livers. Additionally, the relative abundance of metabolites in the TCA cycle were greater (*p* = 0.04) in fetal livers from the NOVTM compared with VTM supplemented heifers and tended to be greater (*p* = 0.09) in the fetal livers from the MG compared with LG heifers.

## 4. Discussion

These data are the first to report the combined effects of rate of weight gain and vitamin-mineral supplementation of heifers during the periconceptual period on the fetal hepatic amino acid, carbohydrate, and energy metabolomes. Coupled with previously published data on the maternal and fetal fluid amino acid profiles from this same study [22], the present data allow for a more complete description of the effects of maternal nutrition on fetal metabolism. As mentioned, the majority of research has focused on heifer development leading up to the breeding season with fewer studies investigating the effects of nutrition immediately post-breeding.

The major findings of this study are that out of the 305 biochemicals/metabolites identified in fetal liver pertaining to amino acid, carbohydrate, or energy metabolism, nearly 17% were affected by an interaction of gain × vitamin/mineral supplementation or main effects of rate of weight gain or vitamin/mineral supplementation. Furthermore, the majority of the metabolites that were affected by a main effect of gain had a decreased abundance in fetal liver of MG compared with LG heifers. All differentially abundant metabolites in the Urea Cycle pathway increased in the MG compared with LG for fetal livers, thus suggesting that more amino acids were being catabolized for energy and urea produced in MG fetal liver. This is in agreement with classic fetal flux studies in which α-amino nitrogen flux is towards the fetus and urea nitrogen flux away from the fetus to the dam [28] or towards fetal urine [29]. When evaluating the main effect of vitamin/mineral supplementation, the majority of the metabolites were less abundant in fetal livers of VTM supplemented heifers compared with NOVTM.

The changes in metabolite abundance associated with energy and vitamin/mineral status of the dam may have several explanations:

(1) Fetuses from LG and NOVTM heifers may be storing metabolites for use due to limited or reduced nutrient availability. This is supported by research from Crouse et al. [17] and Diniz et al. [30] that found fetuses from heifers on a restricted diet during the first 50 d of gestation had an increased number of genes upregulated in their liver, muscle, and cerebrum compared with fetuses from control dams, which suggests that when nutrients are restored, a compensatory mechanism is in place to recapture growth.
(2) Vitamins and minerals are cofactors for multiple metabolic reactions. Menezes et al. [31] reported that multiple minerals were decreased in NOVTM fetal liver compared with VTM fetal liver including Se, Cu, Mn, and Co that are involved in multiple metabolic reactions including those involved in high energy phosphate bonds as well as being involved in the electron transport chain. Furthermore, cAMP levels in the same study (NOVTM-LG: 0.9585, VTM-LG: 0.9666, NOVTM-MG: 0.9781, and VTM-MG: 1.1080; gain: *p* = 0.03 and vitamin: *p* = 0.06) were decreased in LG compared with MG, and NOVTM compared with VTM. Cyclic AMP is involved in regulating protein kinases and is a marker of energy abundance. The decrease in cAMP demonstrates decreased energy availability in the LG and NOVTM compared with the MG and VTM fetal livers and suggests that fetuses do require their dams to be on an increased rate of weight gain to meet the requirements for proper fetal metabolism and growth. Crouse et al. [17] reported that fetuses from restricted vs. control dams had a greater transcript abundance of Serine/Threonine protein kinases, ATP-binding, and nucleotide-binding genes, which further supports the above hypothesis that fetuses from heifers fed on a lower rate of weight gain may be compensating for reduced energy and growth in anticipation of restored energy intake. Prezotto et al. [32] reported that when dams were feed restricted in early to mid-gestation and subsequently realimented during mid to late-gestation (Restricted-Restricted-Control or Restricted-Control-Control) the fetal liver weight was increased compared with fetal livers from dams on the control treatment (Control, Control, Control). These data demonstrate the compensation response to nutrient restriction in early gestation followed by realimentation. However, it must be noted that impacts of nutrient restriction such as that of intrauterine growth restriction on postnatal performance (metabolic, reproductive, etc.) should continue and understanding the impacts of the severity and duration of nutrient restriction and the timing of realimentation are warranted.
(3) There is greater fetal hepatic metabolic activity in the MG and VTM fetuses which would explain the decreased metabolites and increased urea in fetal liver and is confirmed by the increase in markers of energy metabolism such as cAMP. The increased metabolic activity in MG compared with LG fetal livers is further evidenced by Baumgaertner et al. [33], who reported that heifers fed to gain 0.75 kg/day from breeding to d 84 of gestation were not only 41.5 kg heaver at parturition, but birthed calves were 2.1 kg heavier with a larger chest circumference compared with calves born to heifers fed to gain 0.20 kg/day, which are similar targeted gains to those in this study. Furthermore, when combining the ultrasonography measurements from the current study with data from Baumgaertner et al. [33] in which heifers were from the same genetic makeup and on similar rates of weight gains and diets, fetuses from the MG treatments had a greater biparietal distance at d 83 of gestation than those on the LG treatment (26.38 ± 0.18 mm vs. 25.88 ± 0.18 mm; *p* = 0.05).

There is a growing body of literature supporting changes in growth of the calf due to early maternal nutrition in cattle [17,30,32,34]; however, a heavy emphasis is still being placed on maternal nutrition during the second and third trimesters when secondary myogenesis, muscle hypertrophy, and adipogenesis occur [35,36]. The present data are novel and demonstrate that even early nutritional programming can alter fetal metabolism and affect immediate fetal size which is sustained through gestation and leads to increased calf birth weight. Further research is needed to determine whether these programmed changes in heifer fetuses (all fetuses on these studies are female) result in meaningful impacts on puberty attainment and fertility. Additionally, further research to determine the effects of maternal nutrition on male offspring development is warranted to determine sex specific differences as well as to better understand the maternal nutrition impacts to bull development and steer performance through the feedlot.

In intrauterine growth restricted (IUGR) fetuses, hepatic and skeletal muscle metabolism are altered, which leads to incidences of metabolic syndrome later in life [37,38,39]. Biomedical models of growth restriction in sheep induced by increased heat and humidity which results in placental insufficiency alters fetal hepatic metabolism such that the concentration of physiological fuels such as Val, Leu, and Ile are increased in IUGR fetal liver [40] which is similar to our study where we report NOVTM-LG fetal livers to be increased in branched chain amino acids. Furthermore, there is a decrease in mitochondrial intermediates, and markers of energy status such as adenosine in IUGR fetal liver which was similar to our study where we report decreases in TCA cycle intermediates and cAMP in LG compared with MG fetal livers [40]. Although this study did not result in the clinical signs of IUGR, the changes in liver weight to body size as reported by Menezes et al., [23] as well as the similarities in metabolite abundance and direction of change may suggest that fetal livers from heifers gaining weight at a slower rate may inevitably become growth restricted compared to fetuses born to heifers gaining at a moderate rate of gain.

Comparison of the relative abundance of metabolites in fetal liver to amino acid analysis in maternal and fetal fluids from the same heifers on the same study presents some interesting and often converse findings. Amino acids in allantoic fluid from the same study [22] found that 18 of 20 amino acids measured were differentially abundant due to the interaction of gain × vitamin/mineral supplementation or the main effect of gain or vitamin/mineral supplementation. Furthermore, the concentrations of amino acids were affected more by vitamin/mineral supplementation, such that those heifers supplemented with the vitamin/mineral mix had greater concentrations of amino acids in their allantoic fluid compared with those not supplemented during the periconceptual period. When evaluating total amino acid metabolite abundance in fetal liver, there was a tendency for amino acid metabolites to be decreased in fetal liver of VTM heifers. Furthermore, those metabolites that were increased in allantoic fluid of MG heifers compared with heifers on the LG treatment, including Glu, Asp, Cys, and Arg, were either decreased in abundance in fetal liver of MG treated heifers or not affected by gain. The fetus and allantoic fluid compartments are linked by the urachus, which drains fetal urine to the allantois; however, the composition of fetal urine and allantoic fluid differ substantially [29]. The present data show that the concentrations of metabolites in allantoic fluid may not have a direct relationship to that of the fetus, but they may reflect the energy balance of the heifer. Further work needs to be carried out to determine the relationship between heifer and fetal metabolism and the fluid compartments surrounding the fetus.

The metabolites with the greatest fold changes were imidazole propionate, tricarballylate, and *N*-methylproline. Imidazole propionate is a product in histidine metabolism, which in humans is used as a marker of diabetes, glucose tolerance, and insulin resistance [41,42]. Increased imidazole propionate, such as that in the NOVTM-MG fetal liver, may suggest altered and perhaps even programmed glucose metabolism that could affect heifer calf performance. Tricarballylate, which also was increased in the NOVTM-MG compared with VTM supplemented fetal livers, binds magnesium and is a competitive inhibitor of aconitate hydratase that isomerizes citrate to isocitrate via the intermediate metabolite aconitase [43]. Aconitase was not affected by treatment and isocitrate was not detected in fetal livers from this study. Furthermore, maternal metabolite abundances did not enable us to determine whether tricarballylate, a rumen product, was endogenous to the fetus or came from maternal circulation; however, fetal Mg concentrations were not affected by treatment and thus, we cannot conclude that the differences in tricarballylate involve a mechanistic response to other metabolites or minerals in the fetus.

When determining pathway directionality, two pathways: (1) the Glycine, Serine, and Threonine pathway and the (2) Leucine, Isoleucine, and Valine pathway were differentially affected by maternal nutritional treatment. Both pathways were affected similarly such that NOVTM-LG was greatest in abundance. Both pathways ultimately function to produce acetyl-CoA, pyruvate, or metabolites within the TCA cycle to generate energy under fasting conditions [44]. Increases in metabolites from these pathways may support the concept that the fetuses of the NOVTM-LG heifers were in a nutritionally restricted state and exhibited slower growth rates to compensate for nutritional restriction of the dams.

## 5. Conclusions

These data are the first to report on the effects of vitamin/mineral supplementation as well as heifer rate of weight gain in early gestation on fetal hepatic metabolism. These data demonstrate that the majority of metabolites that are affected by rate of weight gain or vitamin/mineral supplementation are decreased in heifers on a greater rate of weight gain or vitamin/mineral supplementation. These findings also support greater metabolic activity in fetuses from heifers on an increased rate of weight gain and supplemented with vitamin/mineral compared with those on a slower rate of weight gain or not supplemented with vitamin/mineral. Lastly, these data demonstrate that fetal amino acid, carbohydrate, and energy metabolism can be altered by maternal nutrition during the first trimester of gestation, and suggest, along with other data, that these changes manifest in altered fetal size and calf birthweights. Further work needs to be carried out to determine whether the metabolic changes demonstrated in this study resulted in measurable changes to feed, growth efficiency or reproductive efficiency in the heifer offspring.

## Figures and Tables

**Table 1:** Pathway enrichment scores for each pathway identified. Pathways with enrichment scores >1 have more metabolites with statistically significant fold changes compared to all other pathways within the study.

Superpathway	Subpathway	Pathway Enrichment Score ^1^		
		Gain	Vitamin	Gain × Vitamin
Amino Acid Metabolism	Glycine, Serine, and Threonine	0.35	1.56	5.62
	Alanine and Aspartate	1.06	0.00	0.00
	Glutamate	0.65	4.53	3.81
	Histidine	1.01	0.88	0.00
	Lysine	0.46	3.25	0.00
	Phenylalanine	0.00	0.00	0.00
	Tyrosine	0.35	0.00	1.32
	Tryptophan	0.35	1.56	0.00
	Leucine, Isoleucine, and Valine	0.00	0.00	1.8
	Methionine, Cysteine, SAM, and Taurine	0.31	0.68	0.58
	Urea Cycle; Arginine and Proline	0.44	0.00	2.59
	Creatine	0.00	0.00	0.00
	Polyamine	0.00	0.00	0.00
	Guanidino and Acetamido	0.00	0.00	0.00
	Glutathione	0.56	0.00	0.00
Carbohydrate Metabolism	Glycolysis, Gluconeogenesis, and Pyruvate	0.00	1.34	0.00
	Pentose Phosphate Pathway	0.00	0.00	0.00
	Pentose	0.00	0.00	0.00
	Glycogen	0.00	0.00	0.00
	Fructose, Mannose, and Galactose	0.00	0.00	2.28
	Nucleotide Sugar	0.00	0.00	0.00
	Aminosugar	0.00	2.09	0.00
	Advanced Glycation End-product	0.00	0.00	0.00
Energy Metabolism	TCA Cycle	0.47	2.09	0.00
	Oxidative Phosphorylation	0.00	0.00	0.00

^1^ Pathway enrichment was calculated within the MetaboLync Pathway Analysis software using the following formula: $\left(\frac{k}{m}\right)/\left(\frac{n}{N}\right)$ where *k* = the number of significant metabolites per pathway, *m* = the total number of detected metabolites per pathway, *n* = the number of significant metabolites in the study, and *N* = the total number of detected metabolites in the study.

**Table 2:** Metabolites involved in amino acid metabolism pathways including: (1) Glycine, Serine, and Threonine, (2) Alanine and Aspartate, (3) Glutamine, (4) Histidine, and (5) Lysine metabolism.

	Metabolite	Two-Way ANOVA Main Effects			Two-Way ANOVA Contrasts					
		Gain	Vitamin	Gain:Vitamin	NOVTM-MG	VTM-LG	NOVTM-MG	VTM-MG	VTM-MG	VTM-MG
					NOVTM-LG	NOVTM-LG	VTM-LG	NOVTM-LG	VTM-LG	NOVTM-MG
Glycine, Serine and Threonine Metabolism	glycine	0.6901	0.1548	0.1680	0.94	0.91	1.03	0.94	1.03	1.00
	*N*-acetylglycine	0.6664	0.1463	0.9750	1.04	0.92	1.13	0.94	1.03	0.91
	sarcosine	0.5676	0.4727	0.0497	0.71	0.85	0.84	1.01	1.19	1.42
	dimethylglycine	0.0588	0.0611	0.0132	0.96	0.66	1.45	0.99	1.50	1.03
	betaine	0.0708	0.0197	0.0195	0.97	0.67	1.45	0.95	1.42	0.98
	betaine aldehyde	0.8557	0.5481	0.8424	0.98	0.97	1.00	0.93	0.95	0.95
	serine	0.0475	0.0730	0.0825	0.86	0.87	0.99	0.86	0.99	1.00
	*N*-acetylserine	0.6045	0.3075	0.7645	0.99	0.97	1.02	0.94	0.97	0.95
	threonine	0.9211	0.8148	0.2988	0.94	0.97	0.97	1.01	1.04	1.07
	*N*-acetylthreonine	0.6312	0.7174	0.2792	0.94	0.95	0.99	0.97	1.02	1.03
	homoserine	0.8946	0.9145	0.0467	0.89	0.88	1.01	1.03	1.16	1.15
	homoserine lactone	0.3595	0.9494	0.2773	0.85	0.94	0.90	0.92	0.98	1.09
Alanine and Aspartate Metabolism	alanine	0.7514	0.2222	0.9764	0.99	1.05	0.94	1.03	0.99	1.05
	*N*-acetylalanine	0.8720	0.3951	0.7095	1.02	0.98	1.04	0.98	0.99	0.96
	*N*,*N*-dimethylalanine	0.0746	0.2257	0.5782	1.11	0.86	1.29	1.04	1.21	0.94
	aspartate	0.0183	0.0779	0.4362	0.87	0.90	0.97	0.84	0.93	0.96
	*N*-acetylaspartate (NAA)	0.7745	0.5859	0.6148	0.95	0.76	1.26	0.80	1.06	0.84
	asparagine	0.1065	0.4365	0.6115	0.93	0.96	0.97	0.92	0.96	0.99
	*N*-acetylasparagine	0.0217	0.8178	0.9221	0.82	0.97	0.84	0.82	0.85	1.00
	hydroxyasparagine **	0.9401	0.8651	0.2157	0.96	0.96	1.00	0.99	1.03	1.03
Glutamate Metabolism	glutamate	0.0007	0.3580	0.9168	0.91	0.98	0.93	0.89	0.91	0.98
	glutamine	0.5533	0.6350	0.3371	1.04	1.20	0.86	1.03	0.86	0.99
	alpha-ketoglutaramate *	0.4965	0.9267	0.1820	0.83	0.87	0.96	0.93	1.08	1.12
	*N*-acetylglutamate	0.1907	0.7624	0.0165	0.77	0.86	0.90	0.94	1.10	1.22
	*N*-acetylglutamine	0.6920	0.2855	0.7559	0.99	0.97	1.02	0.93	0.95	0.94
	4-hydroxyglutamate	0.0185	0.0414	0.6237	0.82	0.85	0.96	0.73	0.86	0.89
	gamma-carboxyglutamate	0.8963	0.0472	0.1480	0.95	0.84	1.13	0.91	1.08	0.96
	glutamate, gamma-methyl ester	0.0925	0.5781	0.2678	0.92	0.99	0.93	0.97	0.98	1.05
	*N*-acetyl-aspartyl-glutamate (NAAG)	0.9706	0.3730	0.0069	1.23	1.15	1.07	0.94	0.82	0.77
	beta-citrylglutamate	0.1796	0.2694	0.1725	0.99	1.11	0.90	0.98	0.88	0.99
	carboxyethyl-GABA	0.4769	0.2633	0.0227	0.75	0.72	1.05	0.85	1.18	1.13
	*N*-methyl-GABA	0.1183	0.0161	0.2525	1.04	0.63	1.64	0.89	1.40	0.85
	*S*-1-pyrroline-5-carboxylate	0.7169	0.2078	0.4386	1.04	0.96	1.08	0.89	0.92	0.85
Histidine Metabolism	histidine	0.3143	0.9044	0.3428	0.95	0.97	0.98	0.97	1.00	1.02
	1-methylhistidine	0.0428	0.2295	0.9430	0.84	0.90	0.93	0.75	0.84	0.90
	3-methylhistidine	0.0945	0.1445	0.6573	0.92	0.93	0.99	0.80	0.86	0.87
	*N*-acetylhistidine	0.9703	0.1184	0.6513	1.05	1.16	0.90	1.13	0.97	1.08
	1-carboxyethylhistidine	0.4200	0.9911	0.1420	0.82	0.91	0.90	0.93	1.01	1.13
	hydantoin-5-propionate	0.0193	0.5519	0.4896	1.60	1.23	1.29	1.45	1.18	0.91
	imidazole propionate	0.0115	0.2605	0.6547	1.91	0.79	2.43	1.25	1.59	0.65
	formiminoglutamate	0.0292	0.7594	0.3609	0.21	0.50	0.42	0.41	0.83	1.96
	imidazole lactate	0.4196	0.6874	0.2216	1.85	1.52	1.22	1.28	0.84	0.69
	carnosine	0.3240	0.5726	0.1250	0.84	0.92	0.91	0.96	1.04	1.15
	homocarnosine	0.8970	0.6877	0.1204	0.91	0.94	0.97	1.04	1.11	1.14
	*N*-acetylcarnosine	0.9427	0.9365	0.1132	0.92	0.90	1.02	0.99	1.10	1.08
	anserine	0.2994	0.2889	0.6144	0.92	0.92	1.01	0.90	0.98	0.97
	histamine	0.6434	0.4986	0.2738	0.81	0.91	0.90	1.05	1.16	1.29
	1-methylhistamine	0.6493	0.4623	0.9973	1.00	0.88	1.14	0.88	1.01	0.89
	1-methyl-4-imidazoleacetate	0.0684	0.6038	0.4095	0.74	0.87	0.85	0.78	0.90	1.06
	1-methyl-5-imidazoleacetate	0.0128	0.2055	0.9617	0.77	0.88	0.87	0.70	0.79	0.91
	1-methyl-5-imidazolelactate	0.0576	0.2149	0.5774	0.78	0.83	0.94	0.74	0.89	0.95
	1-ribosyl-imidazoleacetate *	0.8999	0.7201	0.7769	1.09	1.05	1.04	1.09	1.04	1.00
	4-imidazoleacetate	0.6833	0.7021	0.3190	1.25	1.08	1.16	1.01	0.94	0.81
	histidine methyl ester	0.7766	0.0492	0.7283	0.96	0.87	1.11	0.86	0.99	0.89
Lysine Metabolism	lysine	0.2567	0.6274	0.4615	0.95	0.97	0.98	0.96	0.99	1.01
	*N*2-acetyllysine	0.0627	0.3400	0.7550	1.11	1.06	1.04	1.25	1.18	1.13
	*N*6-acetyllysine	0.8815	0.1030	0.4186	0.97	1.04	0.93	1.06	1.02	1.10
	*N*6-methyllysine	0.2053	0.0056	0.1704	1.46	0.87	1.68	0.82	0.94	0.56
	*N*6,*N*6-dimethyllysine	0.8597	0.0295	0.9459	1.00	0.90	1.11	0.89	0.99	0.89
	*N*6,*N*6,*N*6-trimethyllysine	0.8456	0.0704	0.7623	0.99	0.95	1.05	0.95	1.01	0.96
	hydroxy-*N*6,*N*6,*N*6-trimethyllysine *	0.2745	0.5473	0.3013	0.99	0.87	1.14	1.04	1.20	1.05
	5-hydroxylysine	0.1056	0.2497	0.6162	1.08	1.07	1.02	1.11	1.04	1.03
	5-(galactosylhydroxy)-l-lysine	0.6524	0.3259	0.9437	0.98	0.95	1.03	0.93	0.98	0.95
	fructosyllysine	0.2990	0.6047	0.3281	0.98	0.89	1.10	1.01	1.13	1.03
	saccharopine	0.0063	0.2847	0.1755	0.75	1.28	0.58	0.72	0.56	0.97
	2-aminoadipate	0.0893	0.3553	0.2686	0.45	0.51	0.88	0.47	0.93	1.05
	2-oxoadipate	0.0690	0.6160	0.2133	0.61	0.70	0.86	0.62	0.88	1.02
	glutarylcarnitine (C5-DC)	0.0868	0.5505	0.8451	0.74	1.15	0.64	0.91	0.79	1.24
	pipecolate	0.0271	0.7271	0.5053	0.79	0.90	0.88	0.81	0.91	1.03
	6-oxopiperidine-2-carboxylate	0.9827	0.0855	0.0779	0.81	0.97	0.84	1.32	1.37	1.62
	5-aminovalerate	0.1118	0.6292	0.5313	1.10	1.03	1.07	1.22	1.19	1.12
	*N*,*N*,*N*-trimethyl-5-aminovalerate	0.1651	0.0204	0.5350	1.22	0.64	1.90	0.85	1.32	0.69

Red and green shaded cells indicate *p* ≤ 0.05 (red indicates that the mean values are significantly higher for that comparison; green values significantly lower). Light red and light green shaded cells indicate 0.05 < *p* < 0.10 (light red indicates that the mean values trend higher for that comparison; light green values trend lower). For the ANOVA, blue-shaded cells indicate *p* ≤ 0.05; light blue-shaded cells indicate 0.05 < *p* < 0.10. Metabolites denoted with * indicates a compound that has not been confirmed based on a standard but are confident in its identity. Metabolites denoted with ** indicates a compound for which a standard is not available, but we are reasonably confident in its identity.

**Table 3:** Metabolites involved in amino acid metabolism pathways: (1) Phenylalanine, (2) Tyrosine, (3) Tryptophan, (4) Leucine, Isoleucine, and Valine, and (5) Methionine, Cysteine, SAM, and Taurine Metabolism.

Pathway	Metabolite	Two-Way ANOVA Main Effects			Two-Way ANOVA Contrasts					
		Gain	Vitamin	Gain:Vitamin	NOVTM-MG	VTM-LG	NOVTM-MG	VTM-MG	VTM-MG	VTM-MG
					NOVTM-LG	NOVTM-LG	VTM-LG	NOVTM-LG	VTM-LG	NOVTM-MG
Phenylalanine Metabolism	phenylalanine	0.5262	0.1995	0.3534	0.97	1.03	0.95	1.13	1.09	1.16
	*N*-acetylphenylalanine	0.1710	0.8251	0.9814	0.93	1.01	0.92	0.95	0.94	1.02
	1-carboxyethylphenylalanine	0.9206	0.5831	0.2268	0.90	0.99	0.91	1.06	1.07	1.18
	phenyllactate (PLA)	0.1825	0.7811	0.3047	1.00	0.93	1.08	1.12	1.20	1.12
Tyrosine Metabolism	tyrosine	0.2839	0.1136	0.5203	0.90	0.87	1.04	0.85	0.98	0.94
	*N*-acetyltyrosine	0.1829	0.3086	0.7077	0.95	1.10	0.86	0.99	0.90	1.04
	1-carboxyethyltyrosine	0.3515	0.6551	0.2005	0.82	0.87	0.94	0.88	1.02	1.08
	4-hydroxyphenylpyruvate	0.6571	0.7175	0.4672	0.91	0.95	0.96	0.93	0.99	1.03
	3-(4-hydroxyphenyl)lactate	0.1194	0.7794	0.1299	1.01	0.94	1.08	1.14	1.21	1.12
	phenol sulfate	0.7156	0.6369	0.9161	1.01	1.16	0.87	1.09	0.94	1.07
	4-methoxyphenol sulfate	0.4551	0.6799	0.2942	1.15	0.95	1.21	0.35	0.37	0.31
	vanillactate	0.2774	0.5352	0.5575	0.92	1.15	0.80	0.95	0.82	1.02
	3-methoxytyrosine	0.0112	0.7596	0.2593	0.85	1.17	0.73	0.80	0.69	0.94
	*O*-methyltyrosine	0.6826	0.6289	0.0352	0.93	0.90	1.03	0.99	1.10	1.07
	dopamine 4-sulfate	0.7152	0.2172	0.7477	0.98	0.94	1.05	0.90	0.96	0.92
	*N*-formylphenylalanine	0.2733	0.7616	0.8693	0.77	1.03	0.75	0.92	0.89	1.20
Tryptophan Metabolism	tryptophan	0.3681	0.9275	0.1313	0.97	0.95	1.02	1.05	1.11	1.08
	*N*-acetyltryptophan	0.1276	0.7993	0.8266	0.89	1.06	0.85	0.91	0.86	1.02
	*C*-glycosyltryptophan	0.4762	0.4647	0.7849	1.02	1.02	1.00	1.06	1.04	1.04
	oxindolylalanine	0.9601	0.2572	0.2679	0.94	0.89	1.07	0.96	1.09	1.02
	kynurenine	0.6684	0.6015	0.4260	0.88	0.79	1.12	0.89	1.13	1.01
	kynurenate	0.6685	0.3135	0.8373	0.96	0.95	1.01	0.83	0.87	0.86
	*N*-formylanthranilic acid	0.7313	0.4260	0.7723	1.00	0.96	1.04	0.88	0.91	0.87
	picolinate	0.3397	0.1535	0.8080	0.88	0.78	1.13	0.61	0.78	0.69
	serotonin	0.3438	0.4071	0.5512	1.01	1.00	1.00	1.21	1.21	1.21
	indolelactate	0.0007	0.8106	0.1900	1.20	0.93	1.29	1.33	1.43	1.11
	indoleacetylglycine	0.4239	0.0513	0.8167	0.95	0.83	1.15	0.74	0.90	0.78
	3-hydroxy-2-ethylpropionate	0.5943	0.0291	0.3183	0.88	0.74	1.19	0.77	1.04	0.87
Leucine, Isoleucine and Valine Metabolism	leucine	0.0750	0.9078	0.1090	0.90	0.95	0.95	0.94	0.99	1.05
	*N*-acetylleucine	0.6266	0.1516	0.5715	0.87	0.82	1.06	0.81	0.99	0.93
	1-carboxyethylleucine	0.0289	0.8616	0.3462	0.79	0.94	0.84	0.84	0.89	1.06
	alpha-hydroxyisocaproate	0.6909	0.8436	0.3239	0.85	0.91	0.94	0.96	1.06	1.12
	isovalerylglycine	0.6364	0.1351	0.5646	0.94	0.79	1.18	0.82	1.04	0.87
	beta-hydroxyisovalerate	0.1620	0.6902	0.9776	1.22	1.06	1.16	1.27	1.20	1.04
	beta-hydroxyisovaleroylcarnitine	0.5844	0.8602	0.2612	0.96	0.92	1.05	1.03	1.12	1.07
	3-methylglutaconate	0.0897	0.6683	0.2842	0.65	0.90	0.73	0.78	0.87	1.19
	isoleucine	0.1041	0.8273	0.2485	0.93	0.97	0.96	0.95	0.99	1.03
	*N*-acetylisoleucine	0.0883	0.2759	0.0122	1.10	1.15	0.96	0.72	0.62	0.65
	1-carboxyethylisoleucine	0.3750	0.9650	0.1246	0.82	0.91	0.90	0.92	1.01	1.12
	2-hydroxy-3-methylvalerate	0.5237	0.8014	0.4875	0.76	0.80	0.95	0.81	1.01	1.06
	2-methylbutyrylcarnitine (C5)	0.3794	0.1415	0.0255	0.77	0.74	1.04	0.85	1.16	1.11
	2-methylbutyrylglycine	0.5173	0.4655	0.2779	0.97	0.84	1.15	0.99	1.18	1.03
	tiglylcarnitine (C5:1-DC)	0.7393	0.3314	0.0011	0.77	0.70	1.10	0.99	1.40	1.28
	3-hydroxy-2-ethylpropionate	0.7844	0.1849	0.1361	0.92	0.84	1.10	0.93	1.11	1.01
	butyryl/isobutyryl CoA	0.9978	0.8187	0.5227	0.87	0.86	1.00	0.96	1.11	1.11
	ethylmalonate	0.0175	0.6535	0.7402	0.88	1.07	0.82	0.88	0.82	1.01
	methylsuccinate	0.6889	0.0841	0.6010	0.91	0.79	1.15	0.78	0.98	0.86
	valine	0.0089	0.3786	0.2571	0.88	0.99	0.89	0.94	0.95	1.07
	*N*-acetylvaline	0.8181	0.7265	0.1477	0.90	0.87	1.04	0.96	1.11	1.07
	1-carboxyethylvaline	0.0769	0.8592	0.2042	0.81	0.92	0.88	0.87	0.95	1.08
	3-methyl-2-oxobutyrate	0.1586	0.9675	0.8355	0.83	0.98	0.84	0.84	0.86	1.02
	alpha-hydroxyisovalerate	0.0277	0.3426	0.8951	0.86	0.94	0.91	0.81	0.87	0.95
	isobutyrylcarnitine (C4)	0.5091	0.5229	0.9301	0.89	0.90	0.99	0.87	0.96	0.97
	isobutyrylglycine	0.0777	0.1447	0.3581	0.91	0.94	0.98	0.72	0.77	0.78
	3-hydroxyisobutyrate	0.3841	0.8606	0.2047	0.86	0.91	0.95	0.93	1.02	1.08
Methionine, Cysteine, SAM and Taurine Metabolism	methionine	0.8320	0.9846	0.7597	0.97	0.98	0.99	0.99	1.01	1.02
	*N*-acetylmethionine	0.7988	0.6990	0.3953	0.92	0.91	1.02	0.94	1.03	1.01
	*N*-formylmethionine	0.8162	0.6270	0.7966	1.02	1.00	1.02	0.94	0.94	0.91
	*S*-methylmethionine	0.2451	0.0804	0.2018	0.99	0.94	1.05	0.79	0.83	0.80
	methionine sulfone	0.0048	0.0341	0.2338	0.85	0.89	0.96	0.62	0.69	0.72
	methionine sulfoxide	0.9801	0.5705	0.9165	1.01	1.05	0.96	1.05	1.00	1.04
	*N*-acetylmethionine sulfoxide	0.8310	0.9902	0.7681	1.07	1.07	1.01	1.05	0.98	0.97
	*S*-adenosylmethionine (SAM)	0.5308	0.3777	0.4083	0.91	1.02	0.89	1.00	0.99	1.11
	*S*-adenosylhomocysteine (SAH)	0.3151	0.2339	0.2600	0.99	1.01	0.98	1.11	1.09	1.12
	5-methylthioribose **	0.4375	0.5182	0.8871	1.04	0.98	1.06	1.01	1.03	0.97
	2,3-dihydroxy-5-methylthio-4-pentenoate	0.9072	0.0509	0.3284	0.97	1.03	0.94	1.07	1.04	1.11
	2-hydroxy-4-(methylthio)butanoic	0.2529	0.6619	0.6947	1.44	1.21	1.19	1.50	1.24	1.04
	homocysteine	0.2852	0.2269	0.9529	0.92	1.13	0.81	0.99	0.88	1.08
	cystathionine	0.3153	0.4010	0.9092	0.94	1.05	0.89	0.99	0.94	1.05
	cysteine	0.8144	0.5822	0.5550	1.05	1.13	0.93	1.04	0.92	0.99
	*N*-acetylcysteine	0.3834	0.3336	0.3481	0.98	1.17	0.83	0.98	0.83	1.00
	*S*-methylcysteine	0.3039	0.6297	0.3937	1.05	0.97	1.09	1.49	1.54	1.41
	*S*-methylcysteine sulfoxide	0.0657	0.7767	0.0704	1.01	0.84	1.19	1.12	1.33	1.11
	cysteine s-sulfate	0.6742	0.9318	0.9945	1.05	1.02	1.03	1.10	1.08	1.04
	cystine	0.9628	0.5386	0.8525	0.83	0.80	1.04	0.88	1.11	1.06
	cysteine sulfinic acid	0.9516	0.4598	0.7822	0.98	1.14	0.86	1.05	0.92	1.07
	hypotaurine	0.0125	0.0937	0.3937	1.19	1.09	1.09	1.53	1.41	1.29
	taurine	0.0925	0.5849	0.0396	0.98	0.85	1.16	1.08	1.27	1.10
	*N*-acetyltaurine	0.8753	0.3345	0.0888	0.88	0.78	1.12	0.93	1.19	1.06
	succinoyltaurine	0.0619	0.6499	0.3868	1.09	0.84	1.29	1.19	1.41	1.09
	taurocyamine	0.2638	0.1720	0.2067	1.66	1.01	1.64	1.08	1.07	0.65
	3-sulfo-l-alanine	0.7564	0.4612	0.2764	1.16	1.17	0.99	1.07	0.91	0.92

Red and green shaded cells indicate *p* ≤ 0.05 (red indicates that the mean values are significantly higher for that comparison; green values significantly lower). Light red and light green shaded cells indicate 0.05 < *p* < 0.10 (light red indicates that the mean values trend higher for that comparison; light green values trend lower). For the ANOVA, blue-shaded cells indicate *p* ≤ 0.05; light blue-shaded cells indicate 0.05 < *p* < 0.10. Metabolites denoted with ** indicates a compound for which a standard is not available, but we are reasonably confident in its identity.

**Table 4:** Metabolites involved in amino acid metabolism pathways including: (1) Urea cycle, Arginine, and Proline, (2) Creatine, (3) Polyamine, (4) Guanidino and Acetamido, and (5) Glutathione metabolism.

Pathway	Metabolite	Two-Way ANOVA Main Effects			Two-Way ANOVA Contrasts					
		Gain	Vitamin	Gain:Vitamin	NOVTM-MG	VTM-LG	NOVTM-MG	VTM-MG	VTM-MG	VTM-MG
					NOVTM-LG	NOVTM-LG	VTM-LG	NOVTM-LG	VTM-LG	NOVTM-MG
Urea cycle; Arginine and Proline Metabolism	arginine	0.8478	0.1751	0.4076	1.05	0.97	1.08	0.95	0.98	0.91
	argininosuccinate	0.3443	0.1471	0.6434	0.98	0.93	1.06	0.84	0.90	0.85
	urea	0.0540	0.8952	0.2783	1.17	1.06	1.10	1.11	1.05	0.95
	ornithine	0.9752	0.0671	0.9283	1.01	0.93	1.08	0.93	1.00	0.92
	3-amino-2-piperidone	0.7296	0.3557	0.5588	1.01	0.99	1.02	0.95	0.96	0.94
	2-oxoarginine *	0.6161	0.7755	0.2100	0.83	0.84	0.98	1.00	1.18	1.21
	citrulline	0.1861	0.3133	0.0377	0.74	0.77	0.97	0.84	1.10	1.13
	homoarginine	0.3467	0.9645	0.0129	1.15	1.25	0.92	0.94	0.75	0.82
	homocitrulline	0.0038	0.9535	0.6506	1.29	0.94	1.38	1.39	1.49	1.08
	proline	0.4551	0.9809	0.2805	0.99	0.97	1.02	1.03	1.06	1.04
	dimethylarginine (SDMA + ADMA)	0.8330	0.6314	0.4786	1.05	1.01	1.03	0.98	0.97	0.94
	*N*-acetylarginine	0.2266	0.1772	0.5058	0.92	1.03	0.89	1.01	0.98	1.09
	*N*-delta-acetylornithine	0.1555	0.6141	0.6692	1.04	0.80	1.30	1.03	1.28	0.98
	trans-4-hydroxyproline	0.5460	0.3362	0.0697	1.04	1.08	0.96	1.01	0.94	0.98
	pro-hydroxy-pro	0.9930	0.8139	0.0483	0.91	0.92	0.98	1.01	1.10	1.11
	*N*-methylproline	0.0091	0.8882	0.2628	2.39	1.20	2.00	1.54	1.28	0.64
	*N*,*N*,*N*-trimethyl-alanylproline betaine (TMAP)	0.1842	0.3672	0.0642	0.79	0.93	0.85	0.97	1.04	1.23
	argininate *	0.6041	0.2495	0.0575	0.73	0.66	1.10	0.84	1.27	1.15
	dimethylguanidino valeric acid (DMGV) *	0.9002	0.9542	0.6950	0.91	0.96	0.95	0.99	1.03	1.08
Creatine Metabolism	guanidinoacetate	0.2718	0.1371	0.9793	1.09	0.91	1.20	0.97	1.06	0.88
	creatine	0.3056	0.3923	0.3726	0.92	0.93	0.99	0.92	1.00	1.00
	creatinine	0.0263	0.3155	0.1463	0.81	0.87	0.93	0.84	0.96	1.04
	creatine phosphate	0.4778	0.3963	0.1362	0.82	0.97	0.85	1.03	1.07	1.26
Polyamine Metabolism	putrescine	0.5608	0.8046	0.7262	1.00	1.00	1.00	1.04	1.04	1.04
	*N*-acetylputrescine	0.4842	0.0708	0.4958	1.00	1.07	0.93	1.14	1.07	1.15
	*N*-acetyl-isoputreanine	0.6131	0.6805	0.4274	1.16	1.04	1.12	1.05	1.01	0.90
	spermidine	0.8145	0.2942	0.8581	0.97	0.89	1.08	0.88	0.99	0.91
	*N*(1′)-acetylspermidine	0.4290	0.4354	0.8203	1.24	0.95	1.30	1.00	1.05	0.81
	spermine	0.1310	0.7210	0.9401	0.89	0.97	0.92	0.85	0.88	0.96
	*N*1,*N*12-diacetylspermine	0.2559	0.1314	0.6602	1.23	0.52	2.38	0.72	1.39	0.58
	5-methylthioadenosine (MTA)	0.8384	0.7439	0.4964	0.95	1.00	0.95	1.00	1.00	1.05
	4-acetamidobutanoate	0.4564	0.4522	0.8593	0.95	0.97	0.98	0.90	0.93	0.94
Guanidino and Acetamido Metabolism	4-guanidinobutanoate	0.6857	0.4207	0.0671	1.86	1.05	1.78	0.82	0.79	0.44
	guanidinosuccinate	0.2206	0.1726	0.8548	0.65	0.74	0.89	0.53	0.72	0.82
Glutathione Metabolism	glutathione, reduced (GSH)	0.9470	0.5327	0.8642	0.98	1.08	0.91	1.07	0.99	1.09
	glutathione, oxidized (GSSG)	0.6325	0.1749	0.7609	1.02	1.07	0.95	1.13	1.06	1.11
	cysteine-glutathione disulfide	0.9078	0.8347	0.5081	0.93	0.91	1.03	0.99	1.09	1.06
	*S*-methylglutathione	0.9103	0.4645	0.5765	0.97	1.02	0.95	1.04	1.02	1.07
	*S*-lactoylglutathione	0.3135	0.5594	0.8245	0.82	0.87	0.94	0.74	0.86	0.91
	cysteinylglycine	0.7261	0.9655	0.7495	0.96	0.99	0.96	0.87	0.87	0.91
	cysteinylglycine disulfide *	0.8032	0.2323	0.5772	0.85	0.64	1.32	0.87	1.36	1.03
	cys-gly, oxidized	0.9903	0.5298	0.4338	0.89	0.80	1.11	0.95	1.19	1.07
	5-oxoproline	0.0403	0.3065	0.2612	0.89	1.01	0.88	0.73	0.73	0.82
	2-hydroxybutyrate/2-hydroxyisobutyrate	0.2133	0.4544	0.2458	0.88	0.90	0.98	0.90	1.01	1.03
	ophthalmate	0.0062	0.9343	0.8049	0.75	1.07	0.70	0.77	0.72	1.02
	*S*-(1,2-dicarboxyethyl)glutathione	0.5503	0.5480	0.9660	0.96	0.98	0.98	0.92	0.95	0.96
	4-hydroxy-nonenal-glutathione	0.2902	0.5776	0.2652	1.02	1.27	0.81	0.93	0.73	0.91
	3′-dephospho-CoA-glutathione *	0.4941	0.9283	0.6141	1.00	0.93	1.07	1.02	1.09	1.02
	CoA-glutathione *	0.1656	0.5005	0.3299	1.07	0.99	1.08	1.21	1.22	1.13

Red and green shaded cells indicate *p* ≤ 0.05 (red indicates that the mean values are significantly higher for that comparison; green values significantly lower). Light red and light green shaded cells indicate 0.05 < *p* < 0.10 (light red indicates that the mean values trend higher for that comparison; light green values trend lower). For the ANOVA, blue-shaded cells indicate *p* ≤ 0.05; light blue-shaded cells indicate 0.05 < *p* < 0.10. Metabolites denoted with * indicates a compound that has not been confirmed based on a standard but are confident in its identity.

**Table 5:** Metabolites involved in carbohydrate metabolism pathways including: (1) Glycolysis, Gluconeogenesis, and Pyruvate, (2) Pentose Phosphate Pathway, (3) Pentose, (4) Glycogen, (5) Fructose, Mannose, and Galactose, (6) Nucleotide Sugar, (7) Aminosugar, (8) Advanced Glycation End-Product metabolism.

Pathway	Metabolite	Two-Way ANOVA Main Effects			Two-Way ANOVA Contrasts					
		Gain	Vitamin	Gain:Vitamin	NOVTM-MG	VTM-LG	NOVTM-MG	VTM-MG	VTM-MG	VTM-MG
					NOVTM-LG	NOVTM-LG	VTM-LG	NOVTM-LG	VTM-LG	NOVTM-MG
Glycolysis, Gluconeogenesis, and Pyruvate Metabolism	1,5-anhydroglucitol (1,5-AG)	0.0585	0.6067	0.3522	1.21	0.96	1.27	1.40	1.46	1.15
	glucose	0.3648	0.9280	0.6851	0.97	0.89	1.08	1.02	1.14	1.05
	glucose 6-phosphate	0.5025	0.7956	0.0634	1.11	1.26	0.88	0.98	0.77	0.88
	glucose 1-phosphate	0.9300	0.0131	0.5007	1.06	1.49	0.71	1.40	0.94	1.32
	fructose-6-phosphate	0.1441	0.5077	0.1772	0.91	1.13	0.81	0.71	0.63	0.78
	fructose 1,6-diphosphate/glucose 1,6-diphosphate/myo-inositol diphosphates	0.0776	0.1107	0.7328	0.75	0.82	0.91	0.58	0.70	0.77
	2,3-diphosphoglycerate	0.0880	0.2964	0.3735	0.78	0.86	0.91	0.78	0.91	1.00
	dihydroxyacetone phosphate (DHAP)	0.0805	0.1028	0.9521	0.78	0.81	0.97	0.61	0.75	0.77
	2-phosphoglycerate	0.9482	0.6197	0.3701	1.03	1.02	1.01	0.98	0.96	0.95
	3-phosphoglycerate	0.7457	0.3718	0.6649	1.00	1.00	1.01	0.92	0.93	0.92
	phosphoenolpyruvate (PEP)	0.7712	0.3166	0.7888	0.99	1.00	0.99	0.89	0.90	0.90
	pyruvate	0.4192	0.4839	0.5195	0.74	0.84	0.89	0.79	0.94	1.06
	lactate	0.7617	0.6477	0.2892	0.95	0.98	0.97	1.01	1.03	1.05
	glycerate	0.1138	0.6427	0.6373	0.95	1.04	0.91	0.95	0.91	1.00
Pentose Phosphate Pathway	6-phosphogluconate	0.3169	0.3609	0.5766	1.15	1.16	0.99	1.22	1.05	1.06
	ribulose/xylulose 5-phosphate	0.8198	0.1524	0.4411	1.55	2.55	0.61	1.69	0.66	1.09
	ribose 5-phosphate	0.9493	0.9017	0.1523	1.12	1.17	0.96	0.99	0.84	0.88
	ribose 1-phosphate	0.8756	0.8145	0.7914	1.02	1.03	0.99	0.97	0.94	0.95
	sedoheptulose-7-phosphate	0.9215	0.0933	0.3919	1.12	1.41	0.80	1.30	0.93	1.16
Pentose Metabolism	ribose	0.3843	0.1546	0.7655	0.97	0.86	1.12	0.70	0.81	0.72
	ribitol	0.5039	0.5481	0.6527	0.95	0.95	1.00	0.94	0.99	0.99
	ribonate	0.3689	0.4237	0.3819	0.99	1.00	0.99	0.91	0.91	0.92
	ribulose/xylulose	0.2077	0.4605	0.7281	0.95	0.97	0.98	0.81	0.84	0.86
	arabitol/xylitol	0.3537	0.3204	0.8100	1.07	0.93	1.15	0.99	1.07	0.93
	arabonate/xylonate	0.2243	0.1567	0.8196	0.93	0.92	1.02	0.84	0.91	0.89
	sedoheptulose	0.0620	0.1501	0.3486	0.94	1.25	0.75	0.97	0.78	1.04
	lyxonate	0.8156	0.6675	0.7056	0.98	0.92	1.07	0.98	1.06	0.99
Glycogen Metabolism	maltotetraose	0.8424	0.8885	0.2153	1.35	1.54	0.88	1.05	0.68	0.78
	maltotriose	0.9137	0.7785	0.2539	1.32	1.47	0.90	1.07	0.73	0.81
	maltose	0.5077	0.9582	0.6315	1.27	1.08	1.17	1.13	1.05	0.90
Fructose, Mannose and Galactose Metabolism	fructose	0.3265	0.1126	0.3292	1.00	1.03	0.97	1.14	1.11	1.14
	mannitol/sorbitol	0.1781	0.6695	0.0241	0.96	0.90	1.07	1.04	1.15	1.08
	mannose	0.4865	0.7626	0.8222	0.97	1.05	0.92	0.93	0.89	0.96
	mannose-6-phosphate	0.3729	0.6587	0.1689	1.00	1.18	0.85	0.96	0.81	0.95
	galactose 1-phosphate	0.6926	0.2442	0.2481	1.14	1.23	0.92	1.15	0.93	1.01
	2-ketogluconate	0.5514	0.0969	0.3804	1.01	0.96	1.05	0.82	0.85	0.82
	galactonate	0.0934	0.0538	0.2374	0.65	0.64	1.02	0.62	0.98	0.96
Nucleotide Sugar	UDP-glucose	0.5041	0.7344	0.7394	1.20	1.07	1.12	1.11	1.04	0.93
	UDP-galactose	0.7730	0.2830	0.6958	1.13	0.94	1.20	0.85	0.90	0.75
	UDP-glucuronate	0.9565	0.3460	0.3111	0.91	0.99	0.92	1.08	1.09	1.19
	UDP-*N*-acetylglucosamine/galactosamine	0.9726	0.2023	0.9219	0.94	1.13	0.83	1.15	1.02	1.23
	cytidine 5′-monophospho-*N*-acetylneuraminic acid	0.6326	0.5254	0.5279	1.02	1.26	0.81	1.00	0.79	0.98
Aminosugar Metabolism	glucosamine-6-phosphate	0.4187	0.7581	0.1837	1.00	1.17	0.86	0.94	0.80	0.94
	glucuronate	0.3349	0.7981	0.8994	1.22	0.99	1.23	1.10	1.11	0.90
	*N*-acetylglucosamine 6-phosphate	0.4180	0.3776	0.9366	0.89	0.92	0.97	0.83	0.90	0.93
	*N*-acetyl-glucosamine 1-phosphate	0.6283	0.7579	0.6548	1.08	1.11	0.97	0.95	0.86	0.88
	*N*-acetylneuraminate	0.4911	0.8673	0.4253	1.02	1.04	0.98	0.94	0.90	0.92
	*N*-acetylglucosaminylasparagine	0.5502	0.4829	0.5675	1.03	1.00	1.03	1.00	1.00	0.97
	erythronate *	0.4003	0.9382	0.2993	0.91	0.95	0.97	0.95	1.01	1.04
	*N*-acetylglucosamine/*N*-acetylgalactosamine	0.2608	0.0385	0.4075	0.95	0.83	1.15	0.64	0.77	0.67
	*N*-glycolylneuraminate	0.2983	0.9645	0.8015	0.93	0.99	0.94	0.92	0.94	0.99
Advanced Glycation End-product	*N*6-carboxymethyllysine	0.0866	0.7937	0.6084	0.91	1.02	0.89	0.89	0.87	0.98

Red and green shaded cells indicate *p* ≤ 0.05 (red indicates that the mean values are significantly higher for that comparison; green values significantly lower). Light red and light green shaded cells indicate 0.05 < *p* < 0.10 (light red indicates that the mean values trend higher for that comparison; light green values trend lower). For the ANOVA, blue-shaded cells indicate *p* ≤ 0.05; light blue-shaded cells indicate 0.05 < *p* < 0.10. Metabolites denoted with * indicates a compound that has not been confirmed based on a standard but are confident in its identity.

**Table 6:** Metabolites involved in mitochondrial respiration including: (1) TCA Cycle, and (2) Oxidative Phosphorylation pathways.

Pathway	Metabolite	Two-Way ANOVA Main Effects			Two-Way ANOVA Contrasts					
		Gain	Vitamin	Gain:Vitamin	NOVTM-MG	VTM-LG	NOVTM-MG	VTM-MG	VTM-MG	VTM-MG
					NOVTM-LG	NOVTM-LG	VTM-LG	NOVTM-LG	VTM-LG	NOVTM-MG
TCA Cycle	citrate	0.3331	0.8607	0.3612	0.96	1.17	0.82	0.81	0.69	0.85
	aconitate [cis or trans]	0.1831	0.4470	0.7333	0.87	1.30	0.67	0.80	0.61	0.92
	alpha-ketoglutarate	0.4885	0.7102	0.8549	0.91	0.99	0.92	0.94	0.95	1.04
	succinylcarnitine (C4-DC)	0.6616	0.2368	0.1037	0.90	0.81	1.12	0.92	1.15	1.03
	succinate	0.3246	0.9539	0.1196	0.90	0.88	1.02	1.24	1.41	1.38
	fumarate	0.1093	0.0759	0.6135	0.96	0.96	1.00	0.89	0.93	0.93
	malate	0.7981	0.4002	0.4979	1.07	1.00	1.07	0.95	0.94	0.88
	tricarballylate	0.0218	0.0007	0.5281	1.71	0.49	3.49	0.68	1.38	0.39
	2-methylcitrate/homocitrate	0.6635	0.1431	0.2733	0.94	0.90	1.05	0.92	1.03	0.98
Oxidative Phosphorylation	acetylphosphate	0.4156	0.0617	0.0840	1.10	1.43	0.77	1.13	0.79	1.02
	phosphate	0.4261	0.6431	0.1278	0.98	0.95	1.03	1.01	1.06	1.03

Red and green shaded cells indicate *p* ≤ 0.05 (red indicates that the mean values are significantly higher for that comparison; green values significantly lower). Light red and light green shaded cells indicate 0.05 < *p* < 0.10 (light red indicates that the mean values trend higher for that comparison; light green values trend lower). For the ANOVA, blue-shaded cells indicate *p* ≤ 0.05; light blue-shaded cells indicate 0.05 < *p* < 0.10.

**Table 7:** Average abundances of metabolites within a subpathway.

Superpathway	Subpathway	Treatment ^1^				SEM ^3^	*p*-Value ^2^		
		NOVTM-LG	NOVTM-MG	VTM-LG	VTM-MG		Gain	Vitamin	Gain × Vitamin
Amino Acid Metabolism	Total ^4^	1.13	1.08	1.04	1.03	0.037	0.43	0.07	0.63
	Glycine, Serine, and Threonine	1.09 ^a^	1.00 ^bc^	0.96 ^c^	1.05 ^ab^	0.028	0.98	0.11	<0.01
	Alanine and Aspartate	1.19	1.14	1.08	1.08	0.091	0.78	0.38	0.75
	Glutamate	1.08	1.02	1.00	0.98	0.035	0.24	0.12	0.58
	Histidine	1.25	1.12	1.11	1.09	0.101	0.46	0.41	0.59
	Lysine	1.18	1.07	1.06	1.06	0.055	0.31	0.26	0.32
	Phenylalanine	1.02	0.97	1.00	1.08	0.058	0.83	0.38	0.29
	Tyrosine	1.07	1.00	1.06	0.95	0.060	0.14	0.67	0.70
	Tryptophan	1.08	1.04	0.97	1.00	0.051	0.85	0.17	0.51
	Leucine, Isoleucine, and Valine	1.10 ^a^	0.96 ^b^	0.99 ^b^	0.98 ^b^	0.026	0.01	0.08	0.02
	Methionine, Cysteine, SAM, and Taurine	1.04	1.08	1.04	1.10	0.028	0.11	0.75	0.71
	Urea Cycle; Arginine and Proline	1.04	1.07	0.99	1.05	0.040	0.29	0.35	0.66
	Creatine	1.07	0.97	0.99	1.01	0.041	0.35	0.52	0.15
	Polyamine	1.07	1.11	0.99	1.01	0.071	0.66	0.22	0.87
	Gaunidino and Acetamido	1.54	1.64	1.30	0.97	0.259	0.67	0.09	0.41
	Glutathione	1.10	1.01	1.05	1.01	0.035	0.09	0.48	0.48
Carbohydrate Metabolism	Total	1.05	1.09	1.15	1.03	0.057	0.54	0.80	0.15
	Glycolysis, Gluconeogenesis, and Pyruvate	1.08	1.01	1.07	0.98	0.053	0.11	0.66	0.87
	Pentose Phosphate Pathway	0.83	0.95	1.11	0.97	0.079	0.96	0.07	0.11
	Pentose	1.10	1.07	1.06	0.97	0.056	0.28	0.25	0.59
	Glycogen	1.18	1.54	1.62	1.27	0.308	0.97	0.79	0.25
	Fructose, Mannose, and Galactose	1.10	1.03	1.06	1.01	0.044	0.17	0.54	0.79
	Nucleotide Sugar	0.96	1.00	1.03	1.00	0.101	0.99	0.72	0.74
	Aminosugar	1.07	1.07	1.07	0.98	0.046	0.33	0.29	0.34
	Advanced Glycation End-product	1.03	0.94	1.05	0.92	0.075	0.09	0.79	0.61
Energy Metabolism	Total	1.07	1.06	1.07	1.02	0.027	0.19	0.48	0.43
	TCA Cycle	1.13	1.19	1.06	1.01	0.063	0.09	0.04	0.39
	Oxidative Phosphorylation	0.94	0.97	1.10	1.00	0.038	0.38	0.02	0.08

^1^ NOVTM-LG (*n* = 9): fetal livers from heifers not receiving a vitamin/mineral supplement and fed to gain 0.28 kg/day, NOVTM-MG (*n* = 9): fetal livers from heifers not receiving a vitamin/mineral supplement and fed to gain 0.75 kg/day, VTM-LG (*n* = 9): fetal livers from heifers receiving a vitamin/mineral supplement and fed to gain 0.28 kg/day, and VTM-MG (*n* = 8): fetal livers from heifers receiving a vitamin/mineral supplement and fed to gain 0.75 kg/day. ^2^ Probability values for the main effect of rate of weight gain, vitamin/mineral supplementation as well as their interaction. ^3^ Average standard error of the mean for the gain × vitamin interaction. ^4^ Total = average of the sub-pathway metabolite abundance within the Super-pathway. ^a–c^ Means without a common superscript are different by the interaction of rate of weight gain and vitamin/mineral supplementation (*p* ≤ 0.05).

## Data Availability

The data presented in this study are available in the main article and the supplementary materials.

## Supplementary Materials

The following supporting information can be downloaded at: https://www.mdpi.com/article/10.3390/metabo12080696/s1, Table S1: The complete data including *p*-values, FDR protected q-values, fold changes, mean values, and percent filled values for all results in this manuscript. Figure S1: Supplementary Figures Box Plots.
