# Supplementary material for: Vitamin and Mineral Supplementation and Rate of Weight Gain during the First Trimester of Gestation in Beef Heifers Alters the Fetal Liver Amino Acid, Carbohydrate, and Energy Profile at Day 83 of Gestation
Source: Metabolites. 2022 Jul 27;12(8):696. doi: 10.3390/metabo12080696 (PMC9416667; doi:10.3390/metabo12080696)
Supplement: Supplementary file 1 [file metabolites-12-00696-s001.zip › Supplementary Figure S1. Figures Box Plots.pdf]

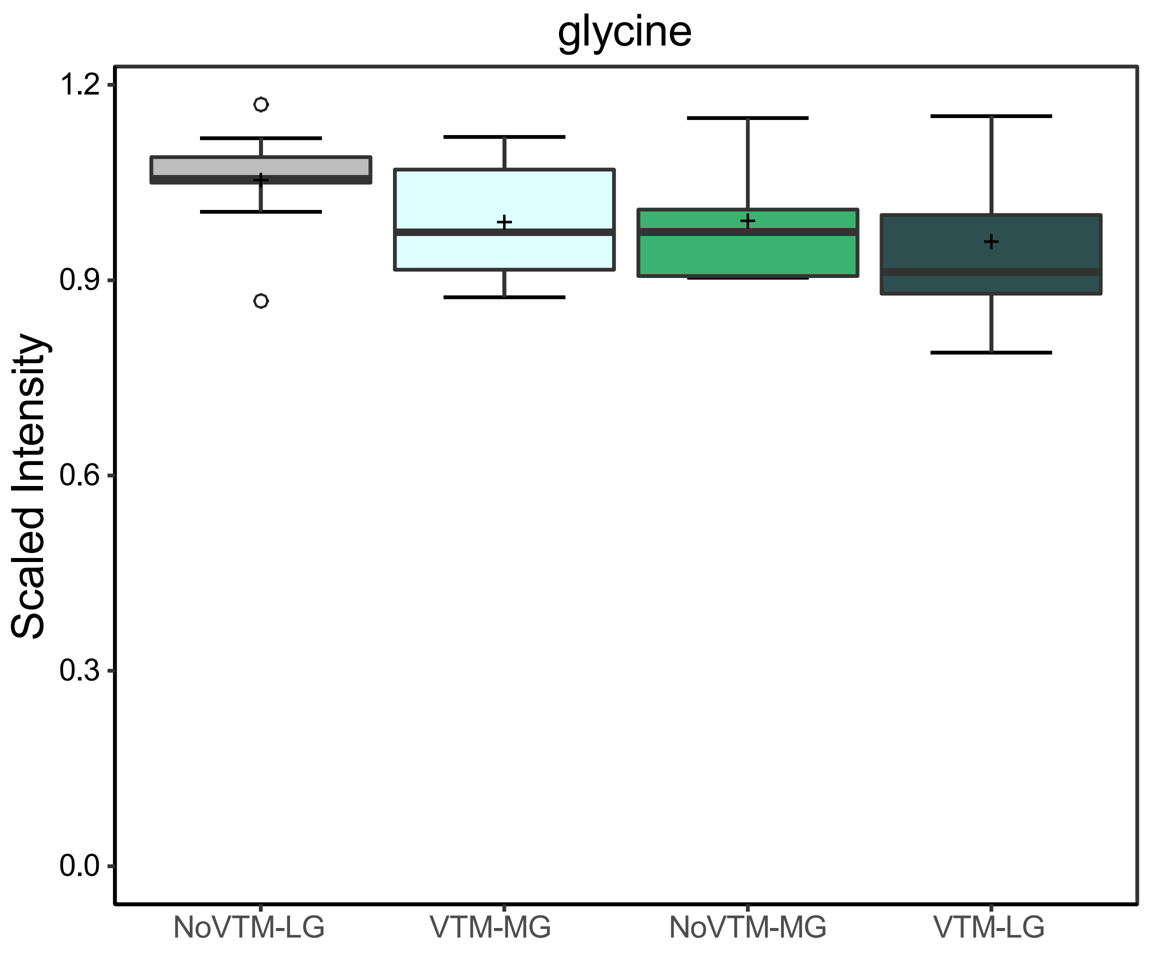

glycine
Scaled Intensity
1.2
0.9
0.6
0.3
0.0
NoVTM-LG
VTM-MG
NoVTM-MG
VTM-LG

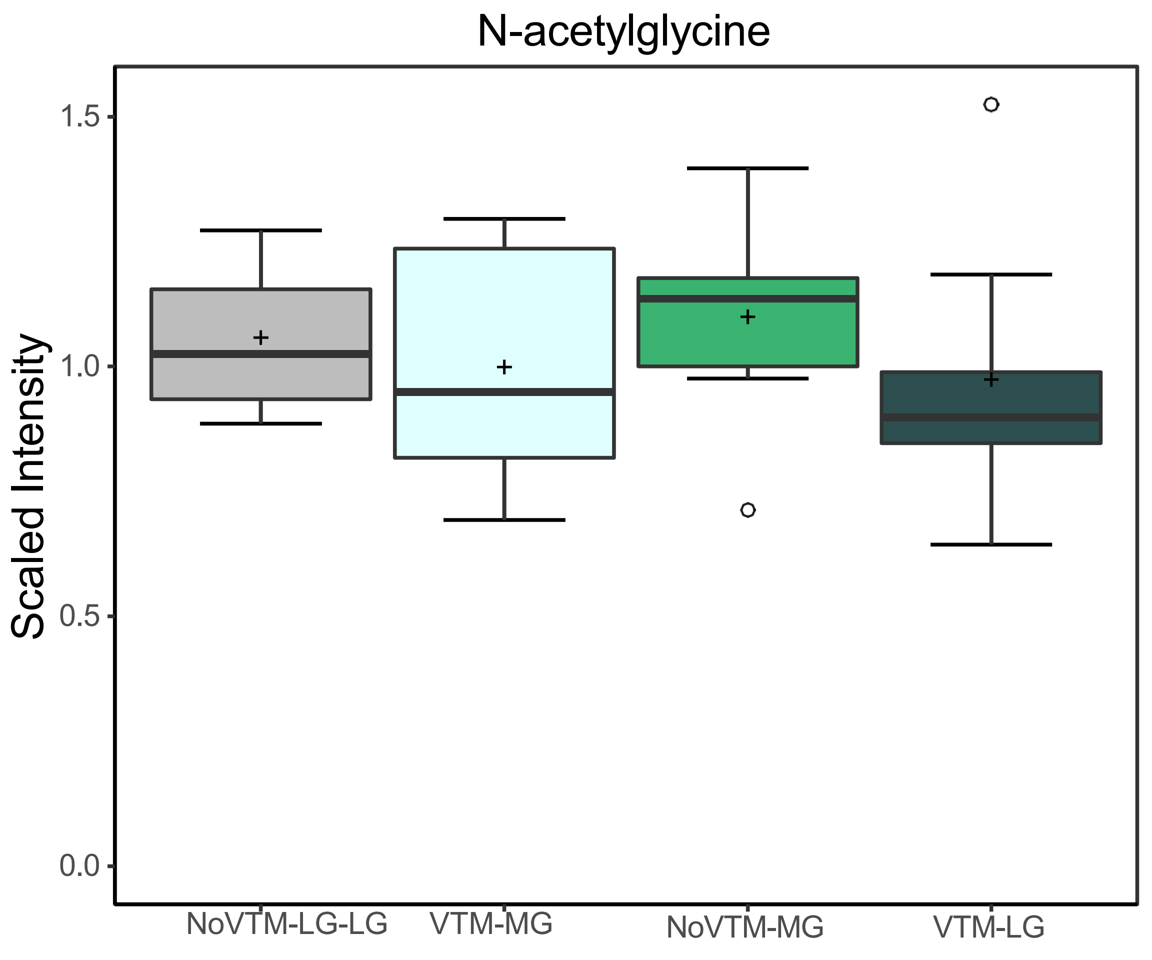

N-acetylglycine
Scaled Intensity
1.5
1.0
0.5
0.0
NoVTM-LG-LG
VTM-MG
NoVTM-MG
VTM-LG

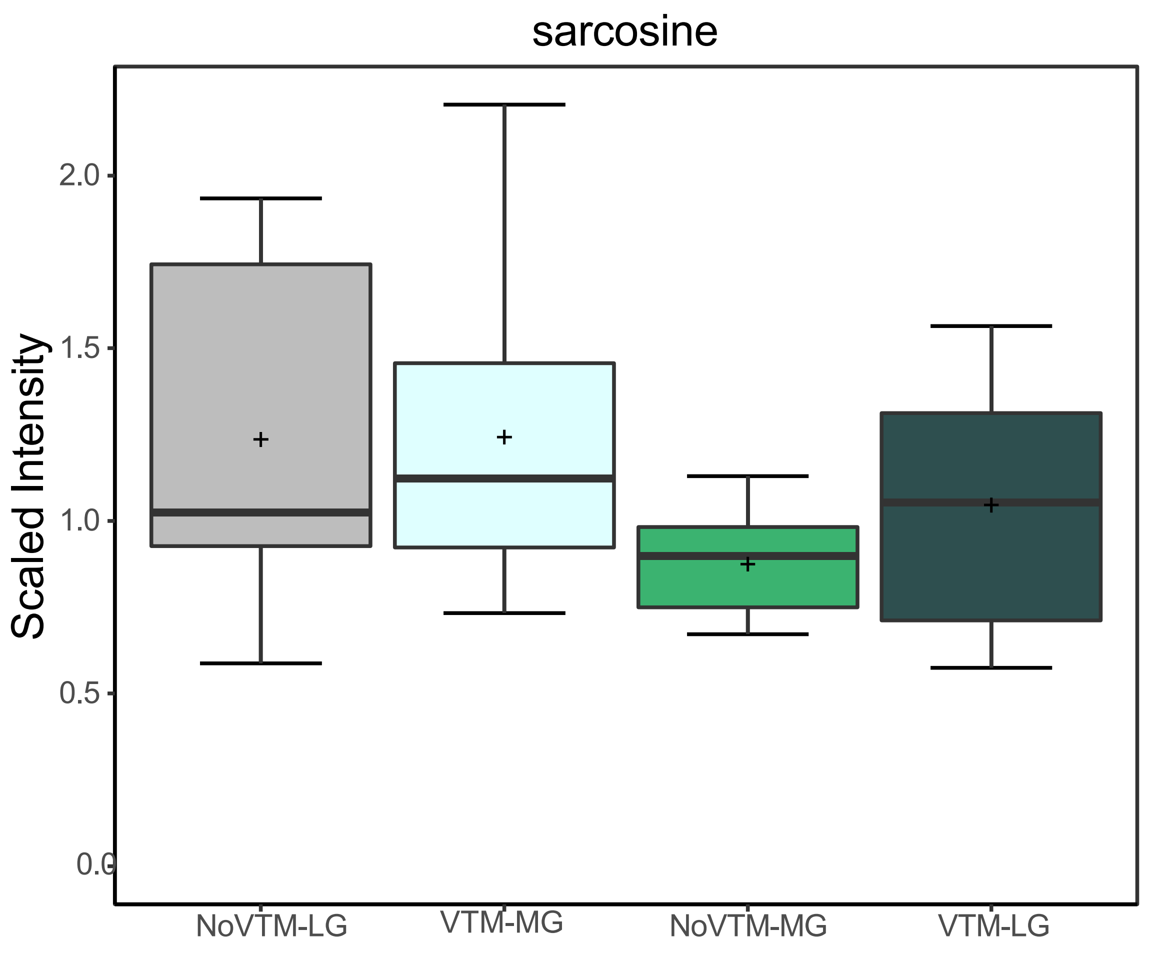

sarcosine
Scaled Intensity
2.0
1.5
1.0
0.5
0.0
NoVTM-LG
VTM-MG
NoVTM-MG
VTM-LG

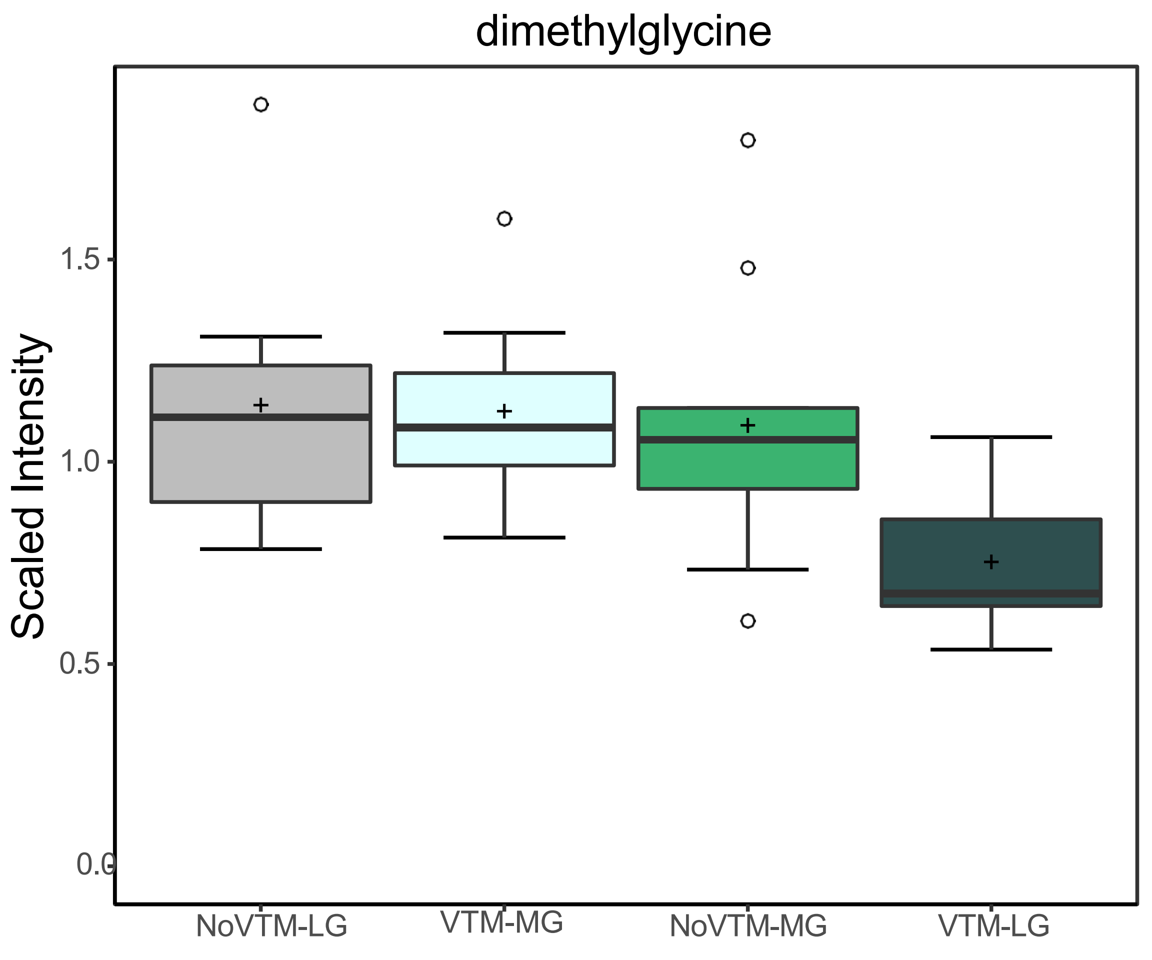
dimethylglycine
Scaled Intensity
1.5
1.0
0.5
0.0
NoVTM-LG
VTM-MG
NoVTM-MG
VTM-LG

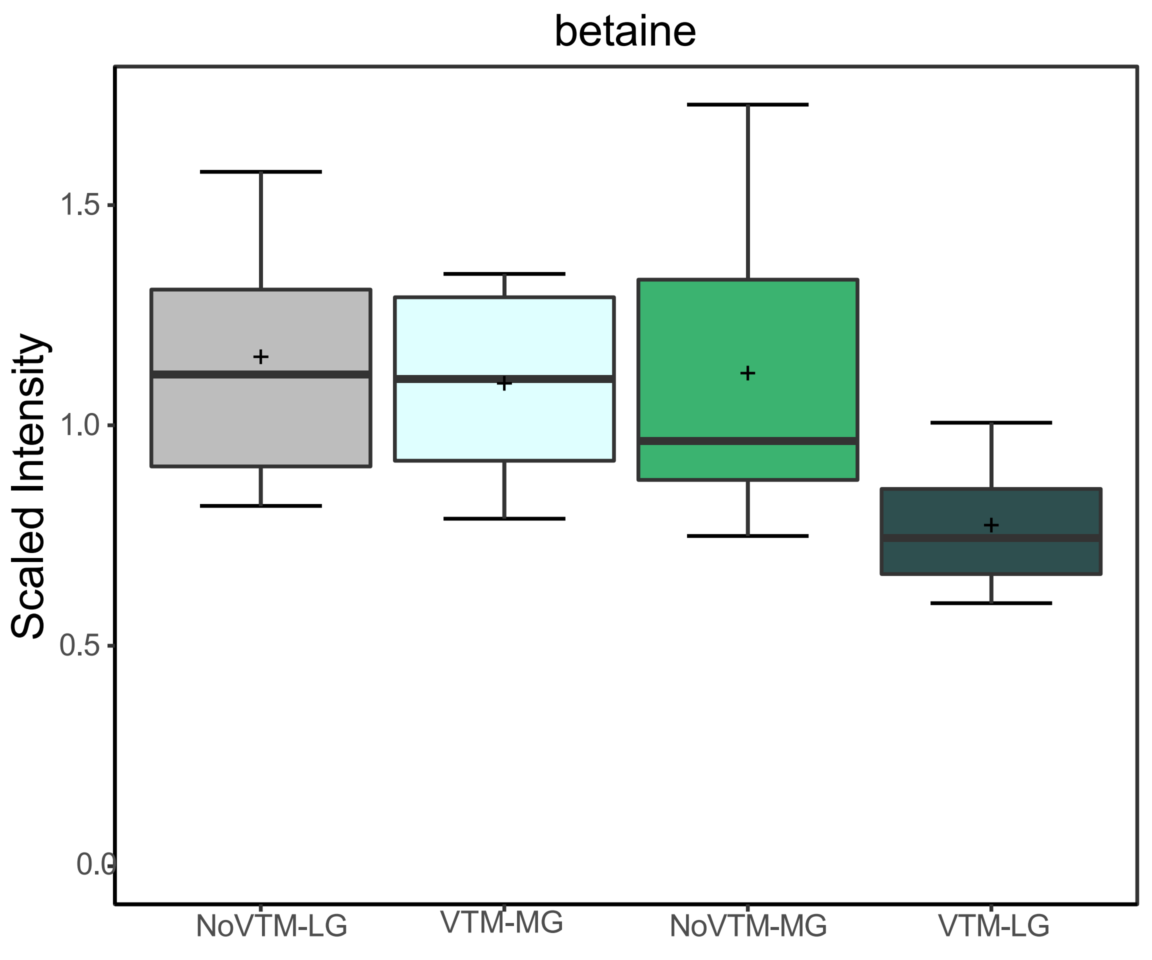
betaine
Scaled Intensity
1.5
1.0
0.5
0.0
NoVTM-LG
VTM-MG
NoVTM-MG
VTM-LG

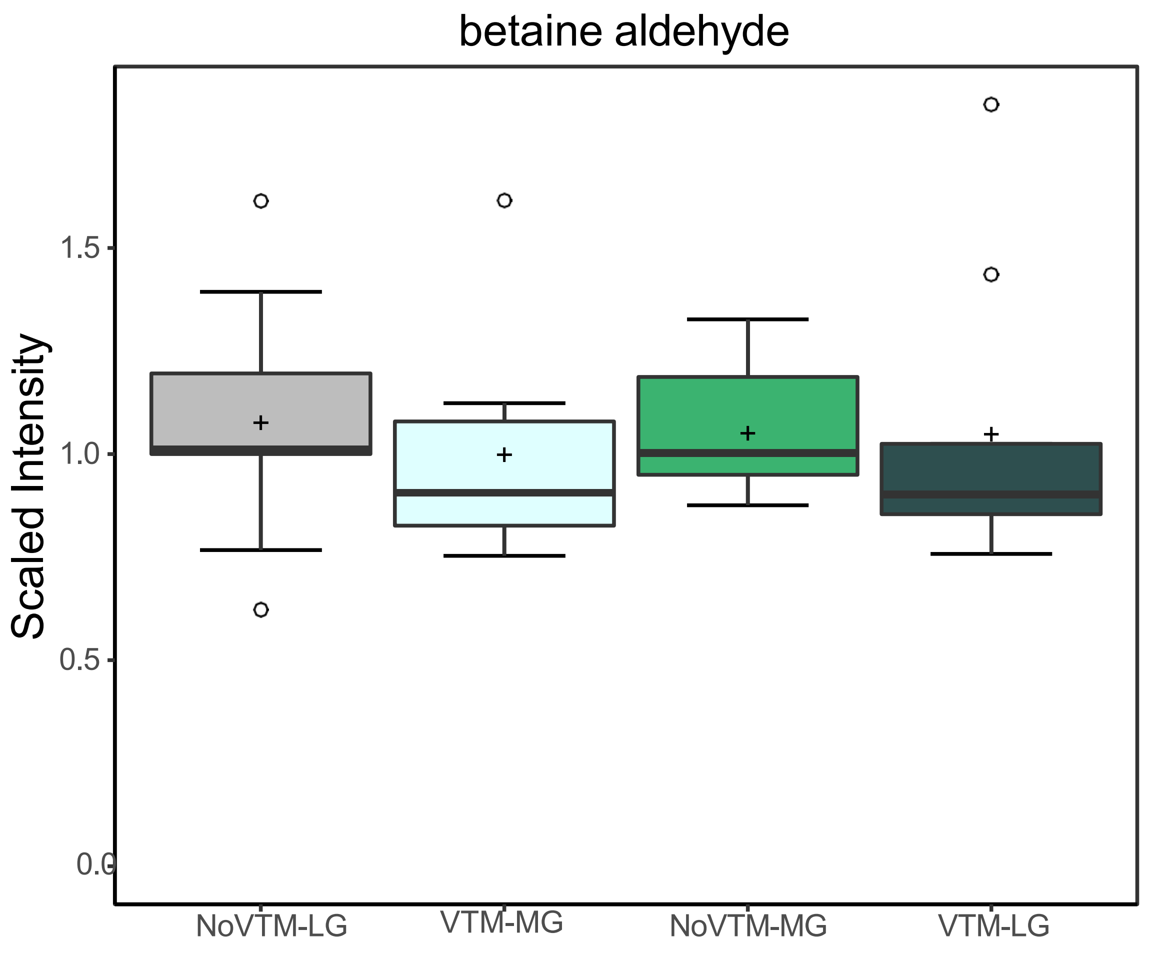
betaine aldehyde
Scaled Intensity
1.5
1.0
0.5
0.0
NoVTM-LG
VTM-MG
NoVTM-MG
VTM-LG

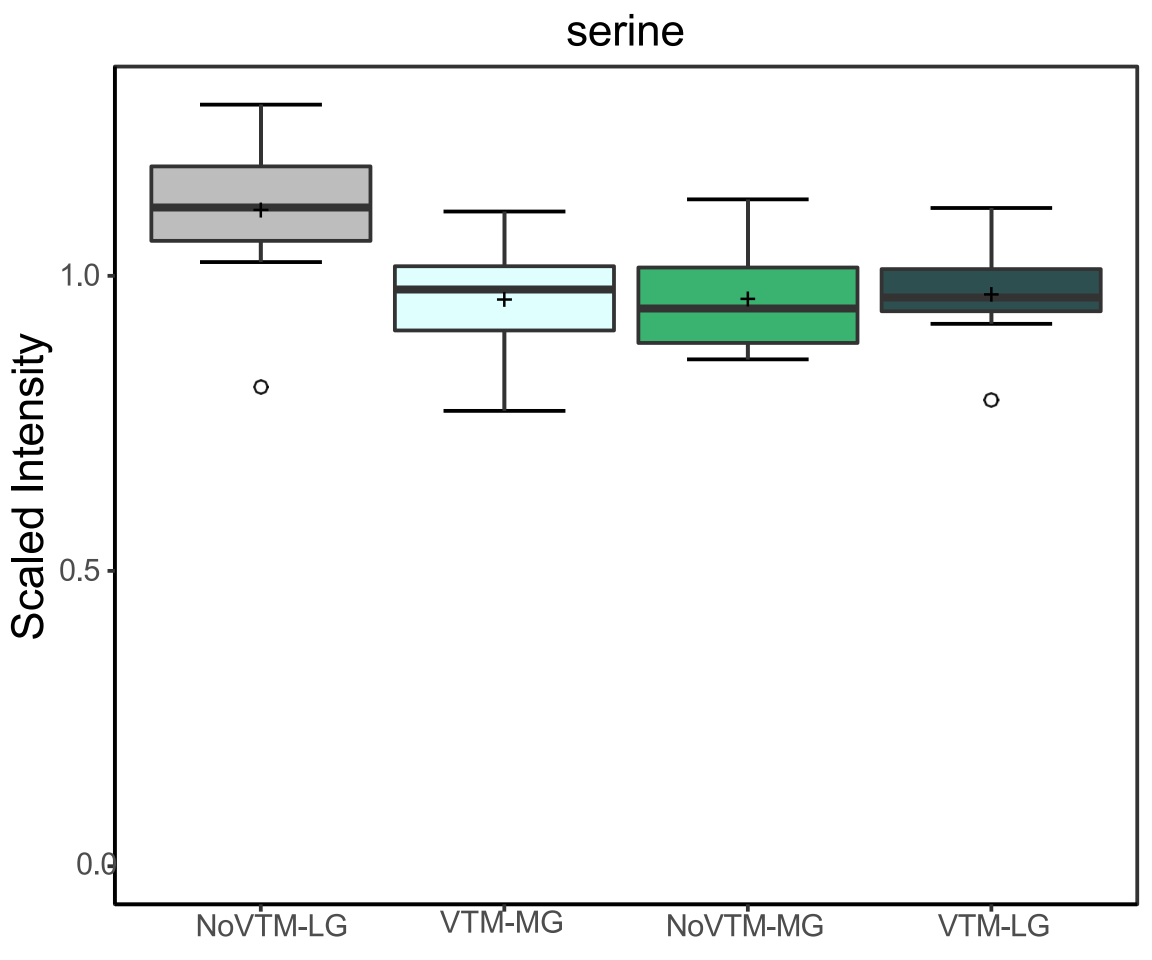

serine
Scaled Intensity
1.0
0.5
0.0
NoVTM-LG
VTM-MG
NoVTM-MG
VTM-LG

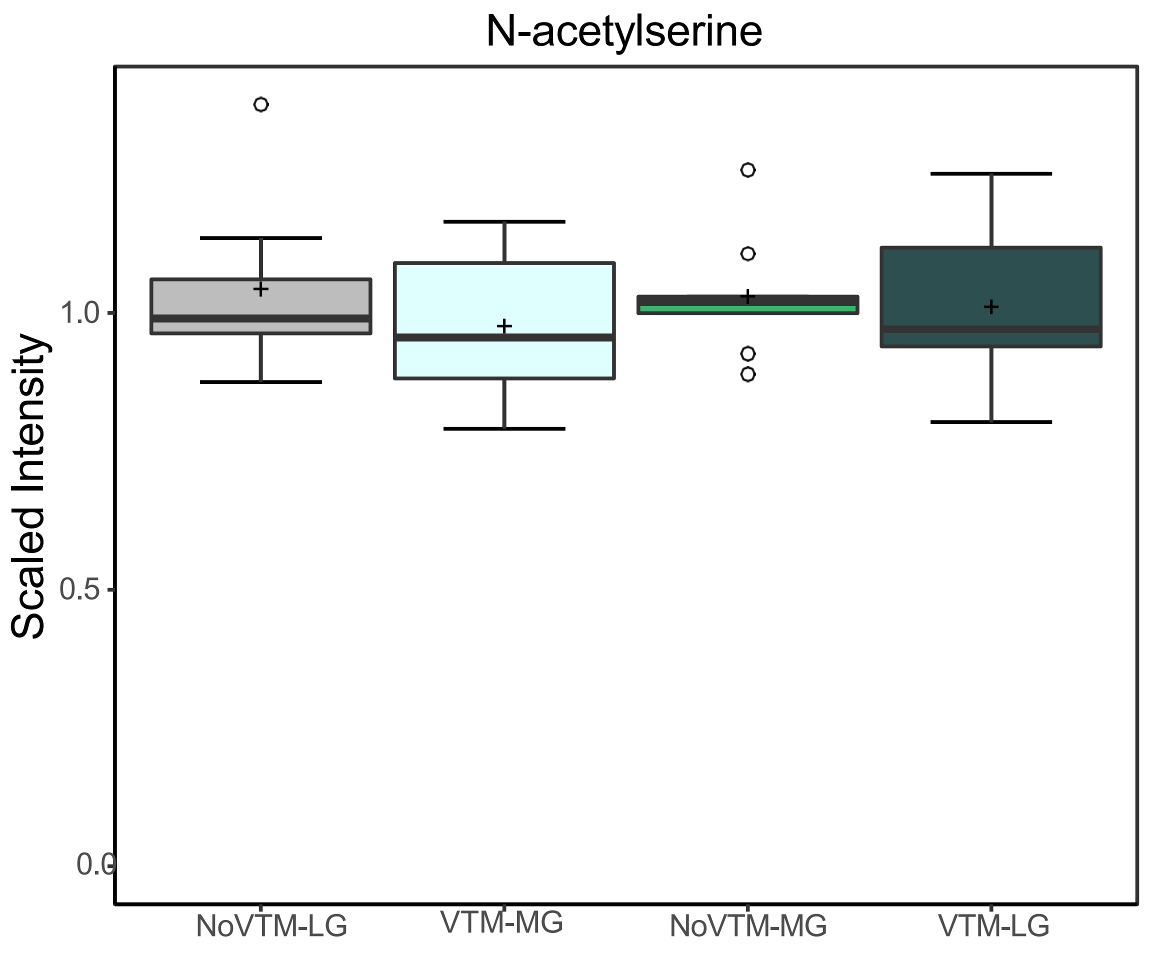
N-acetylserine
Scaled Intensity
1.0
0.5
0.0
NoVTM-LG
VTM-MG
NoVTM-MG
VTM-LG

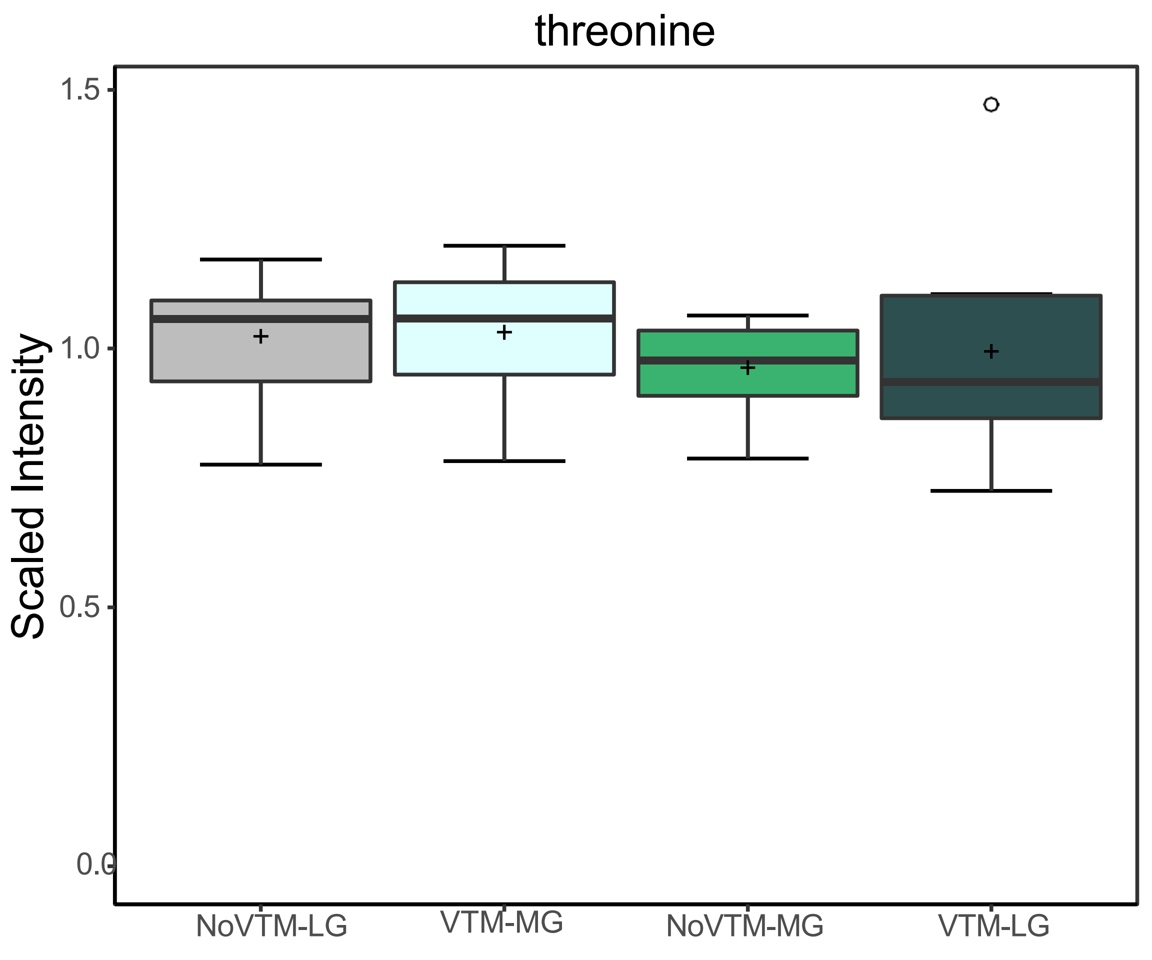
threonine
Scaled Intensity
1.5
1.0
0.5
0.0
NoVTM-LG
VTM-MG
NoVTM-MG
VTM-LG

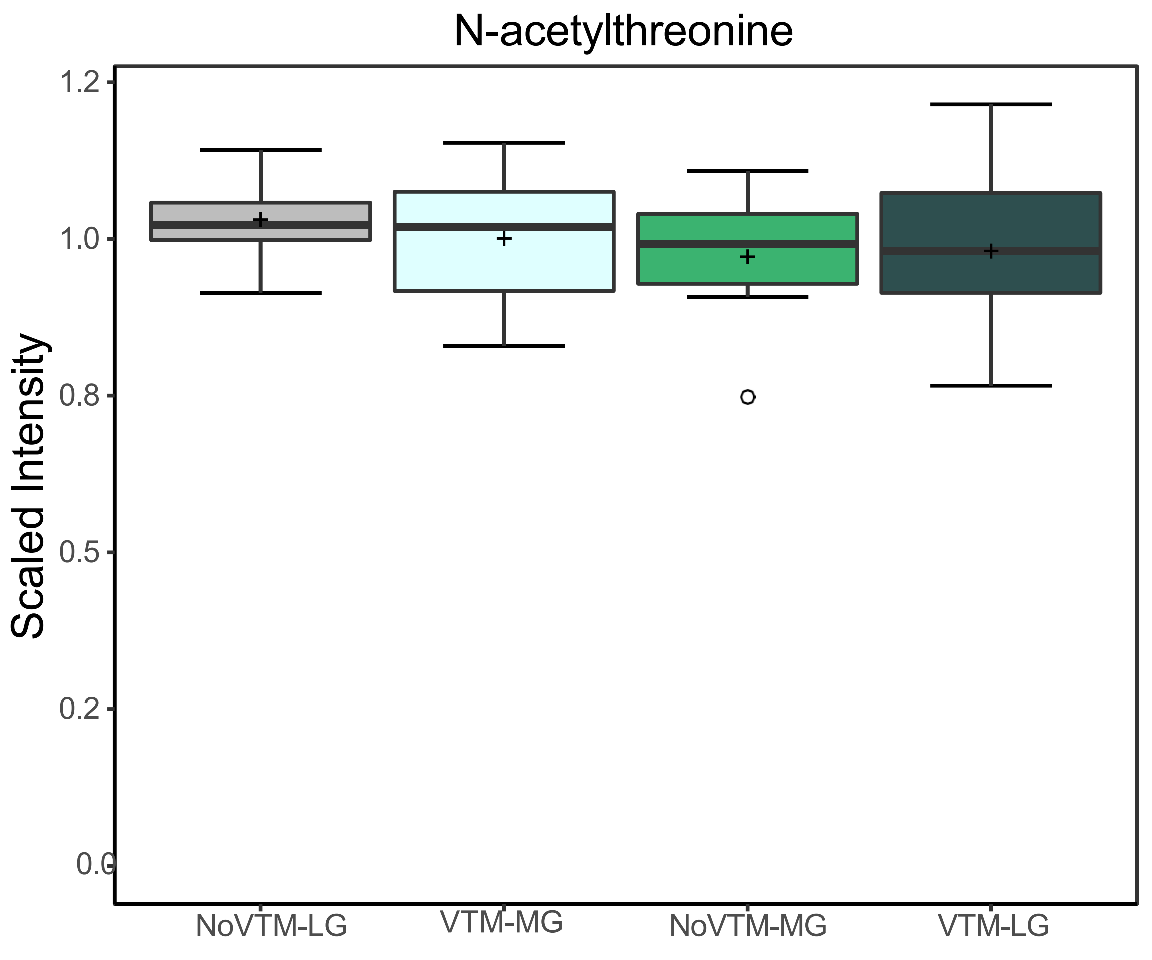

N-acetylthreonine
Scaled Intensity
1.2
1.0
0.8
0.5
0.2
0.0
NoVTM-LG
VTM-MG
NoVTM-MG
VTM-LG

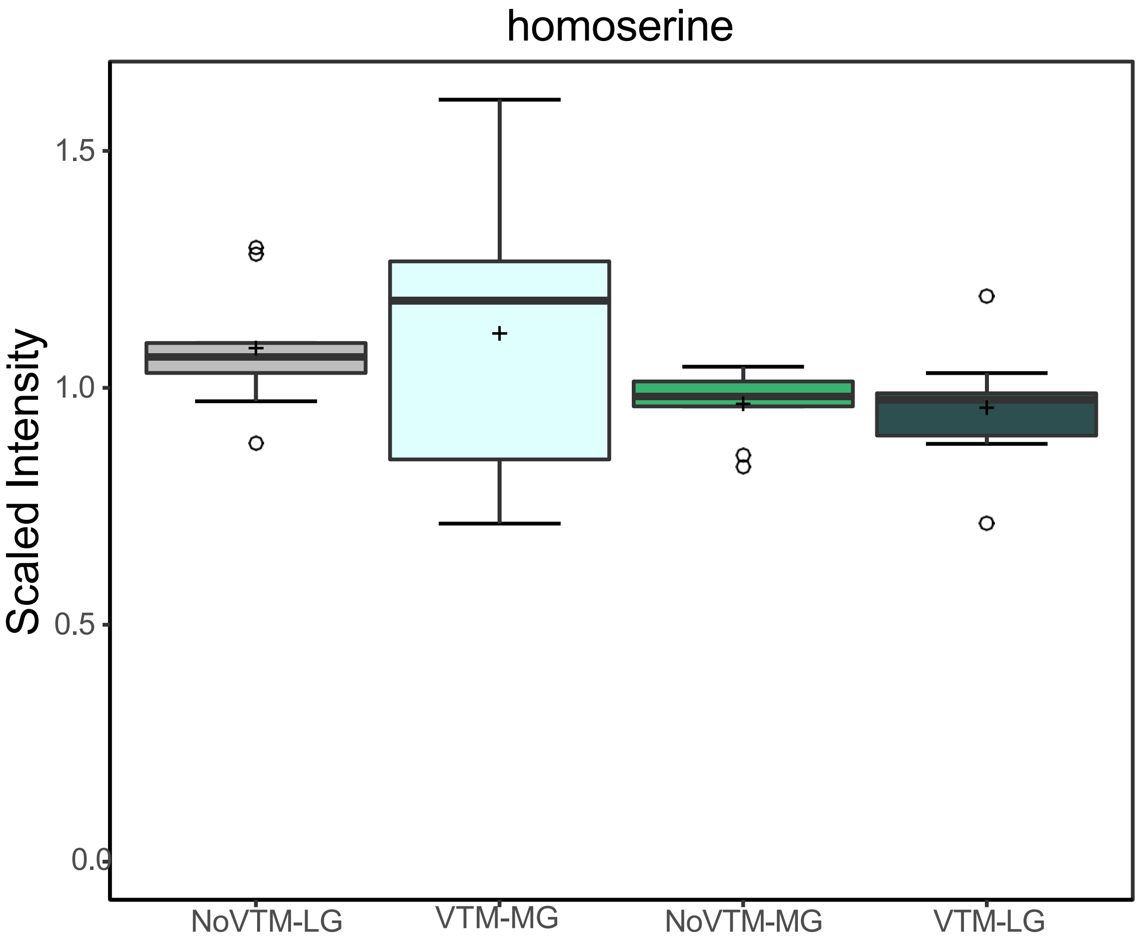
homoserine
Scaled Intensity
1.5
1.0
0.5
0.0
NoVTM-LG
VTM-MG
NoVTM-MG
VTM-LG

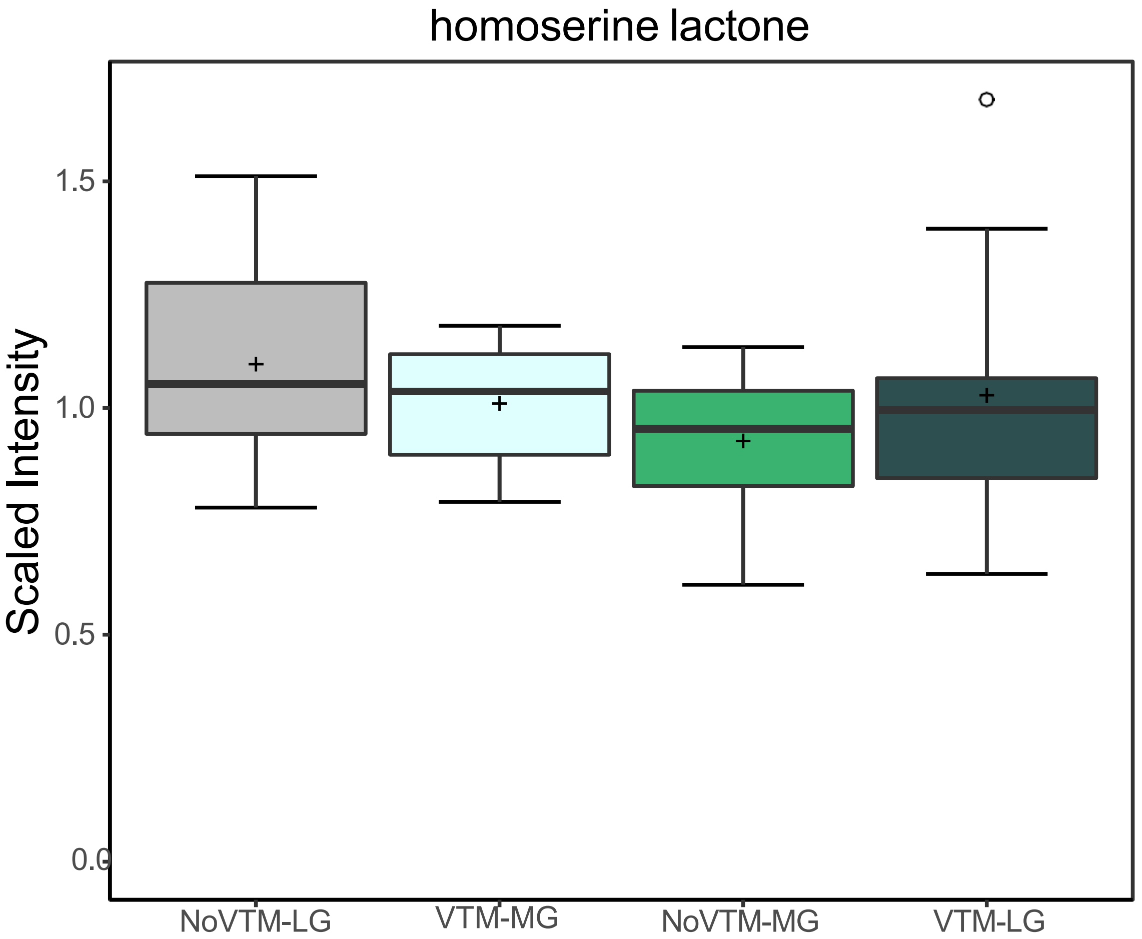
homoserine lactone
Scaled Intensity
1.5
1.0
0.5
0.0
NoVTM-LG
VTM-MG
NoVTM-MG
VTM-LG

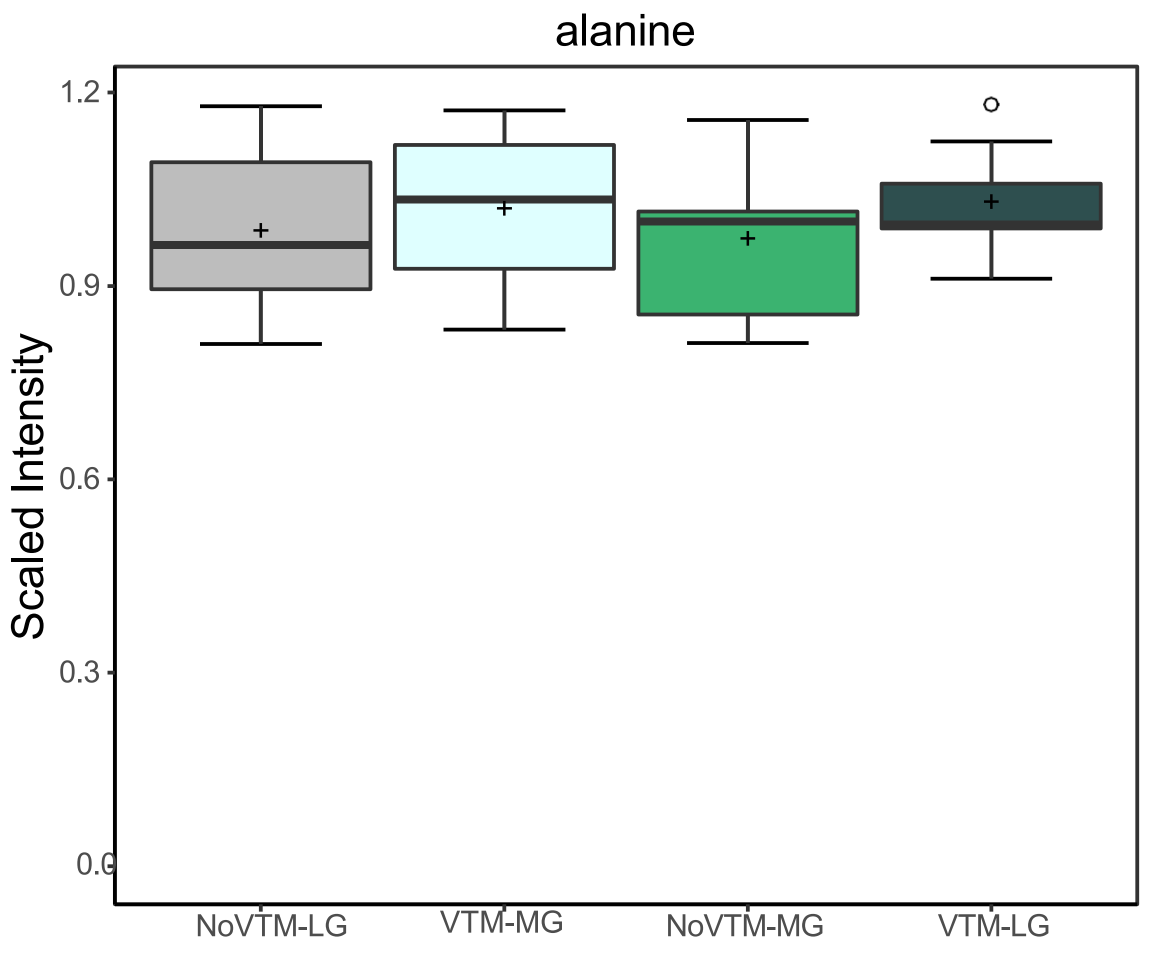
alanine
Scaled Intensity
1.2
0.9
0.6
0.3
0.0
NoVTM-LG
VTM-MG
NoVTM-MG
VTM-LG

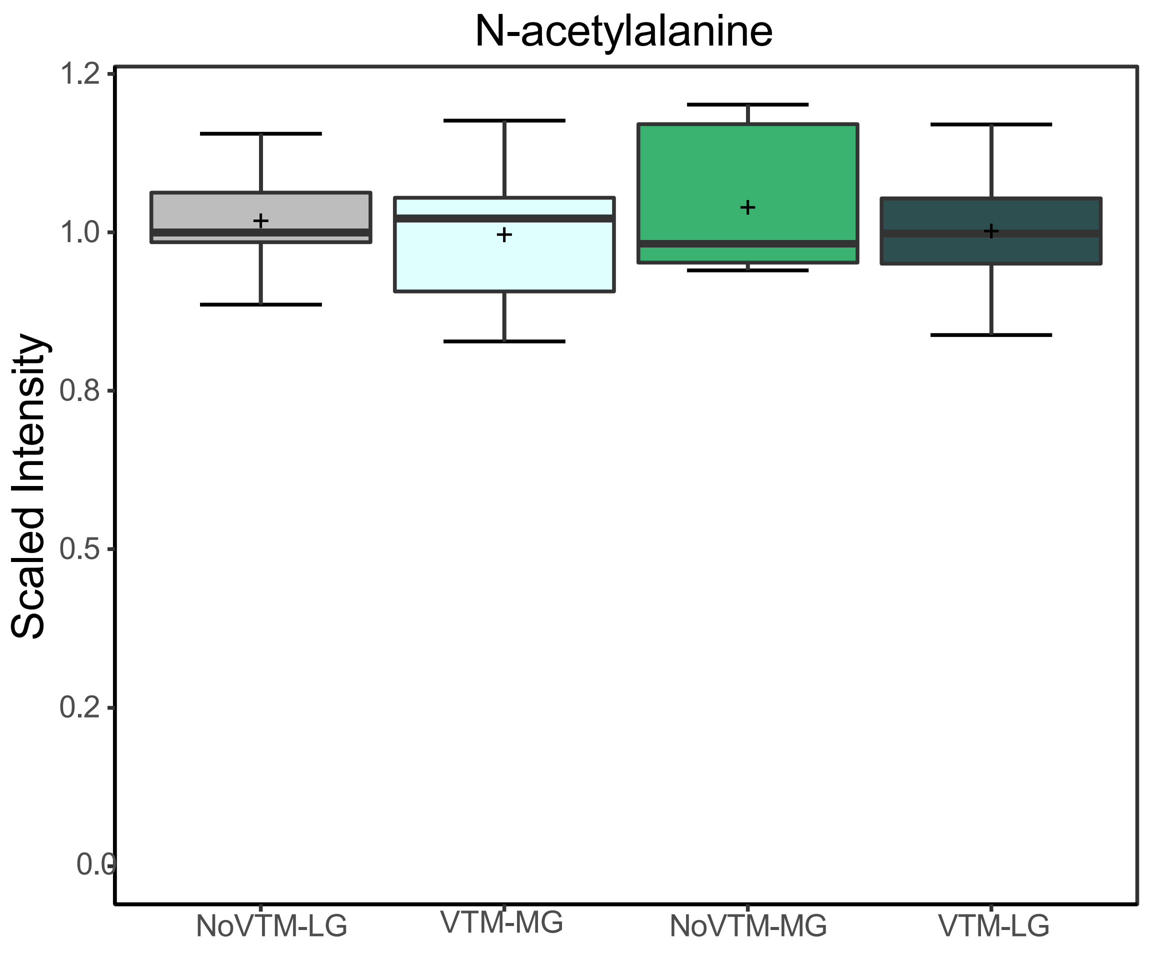
N-acetylalanine
Scaled Intensity
1.2
1.0
0.8
0.5
0.2
0.0
NoVTM-LG
VTM-MG
NoVTM-MG
VTM-LG

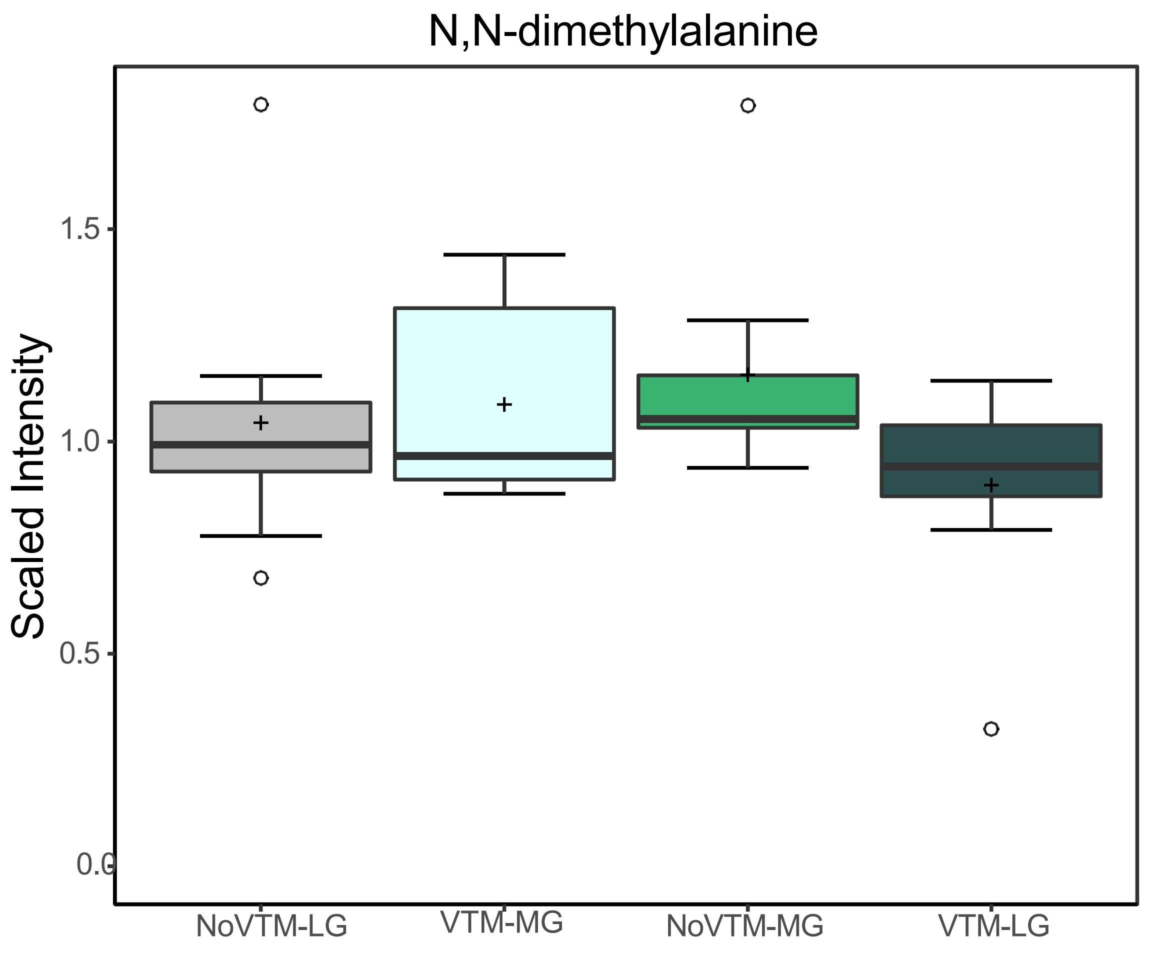

N,N-dimethylalanine
Scaled Intensity
1.5
1.0
0.5
0.0
NoVTM-LG
VTM-MG
NoVTM-MG
VTM-LG

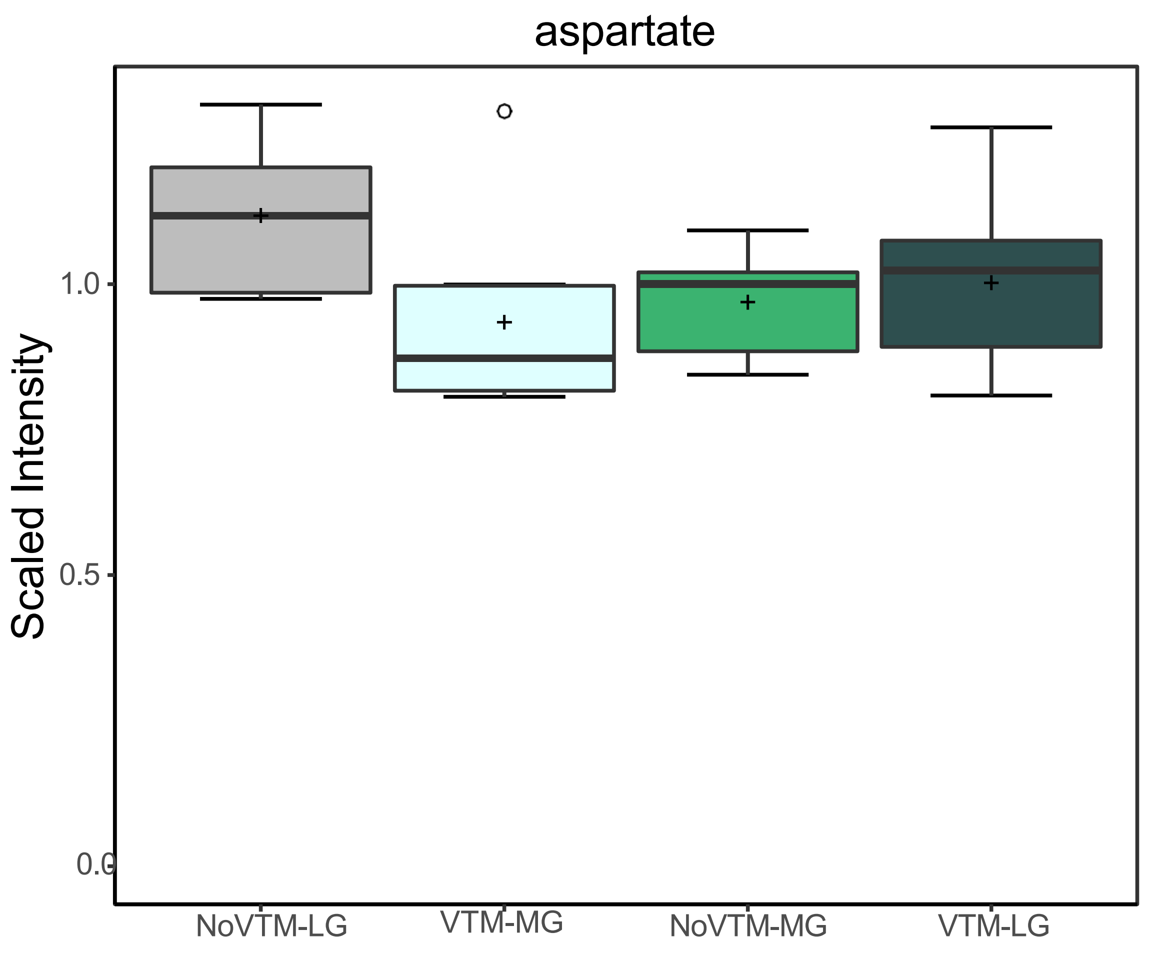

aspartate
Scaled Intensity
1.0
0.5
0.0
NoVTM-LG
VTM-MG
NoVTM-MG
VTM-LG

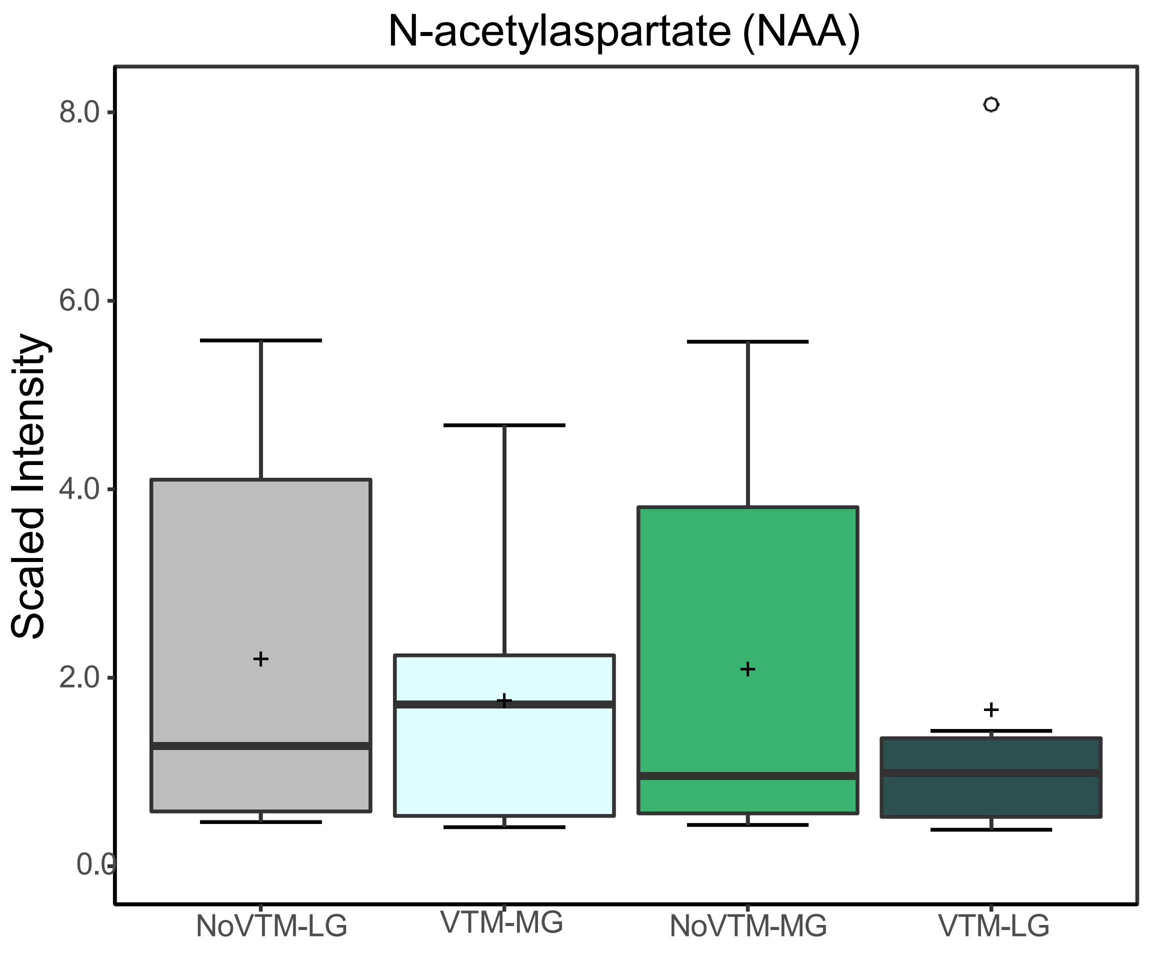
N-acetylaspartate (NAA)
Scaled Intensity
8.0
6.0
4.0
2.0
0.0
NoVTM-LG
VTM-MG
NoVTM-MG
VTM-LG

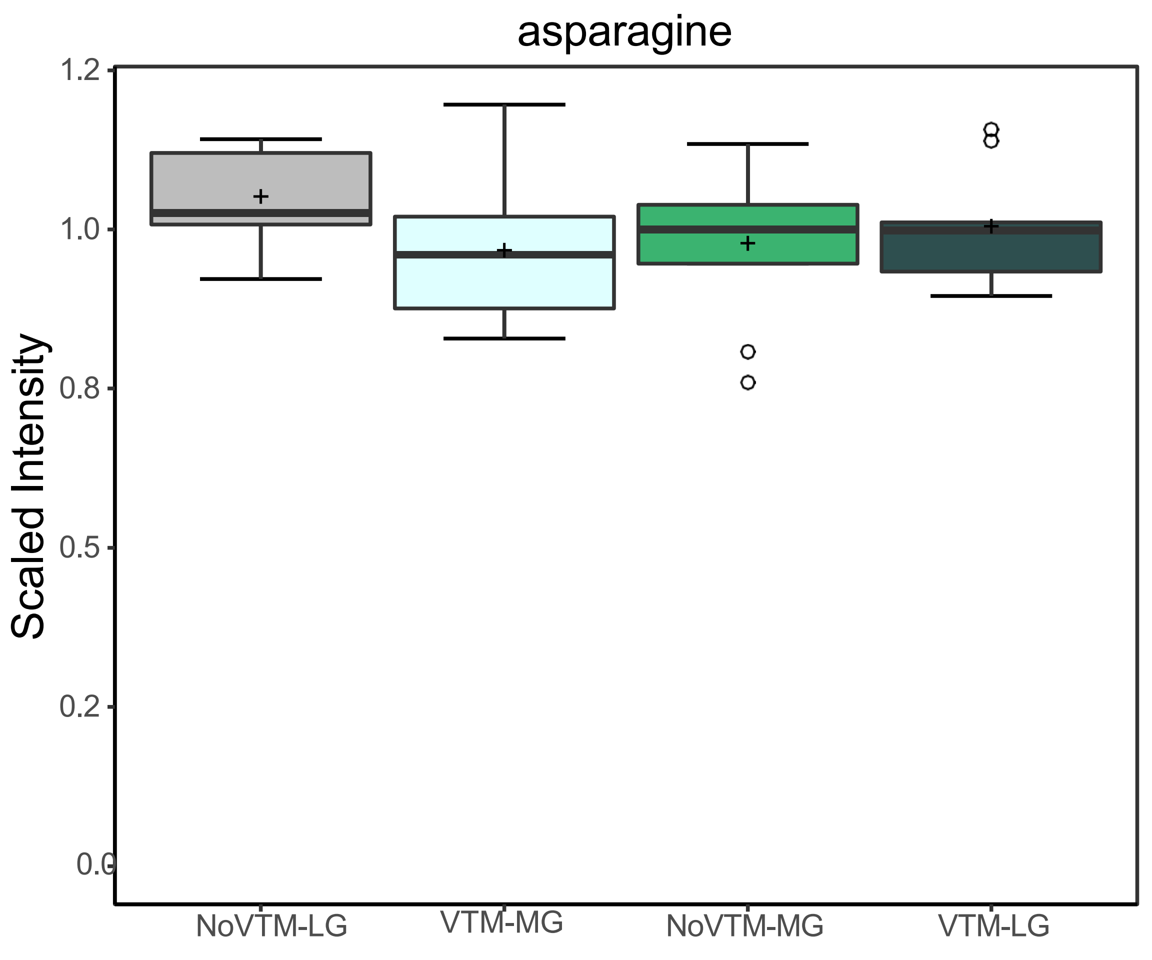
asparagine
Scaled Intensity
1.2
1.0
0.8
0.5
0.2
0.0
NoVTM-LG
VTM-MG
NoVTM-MG
VTM-LG

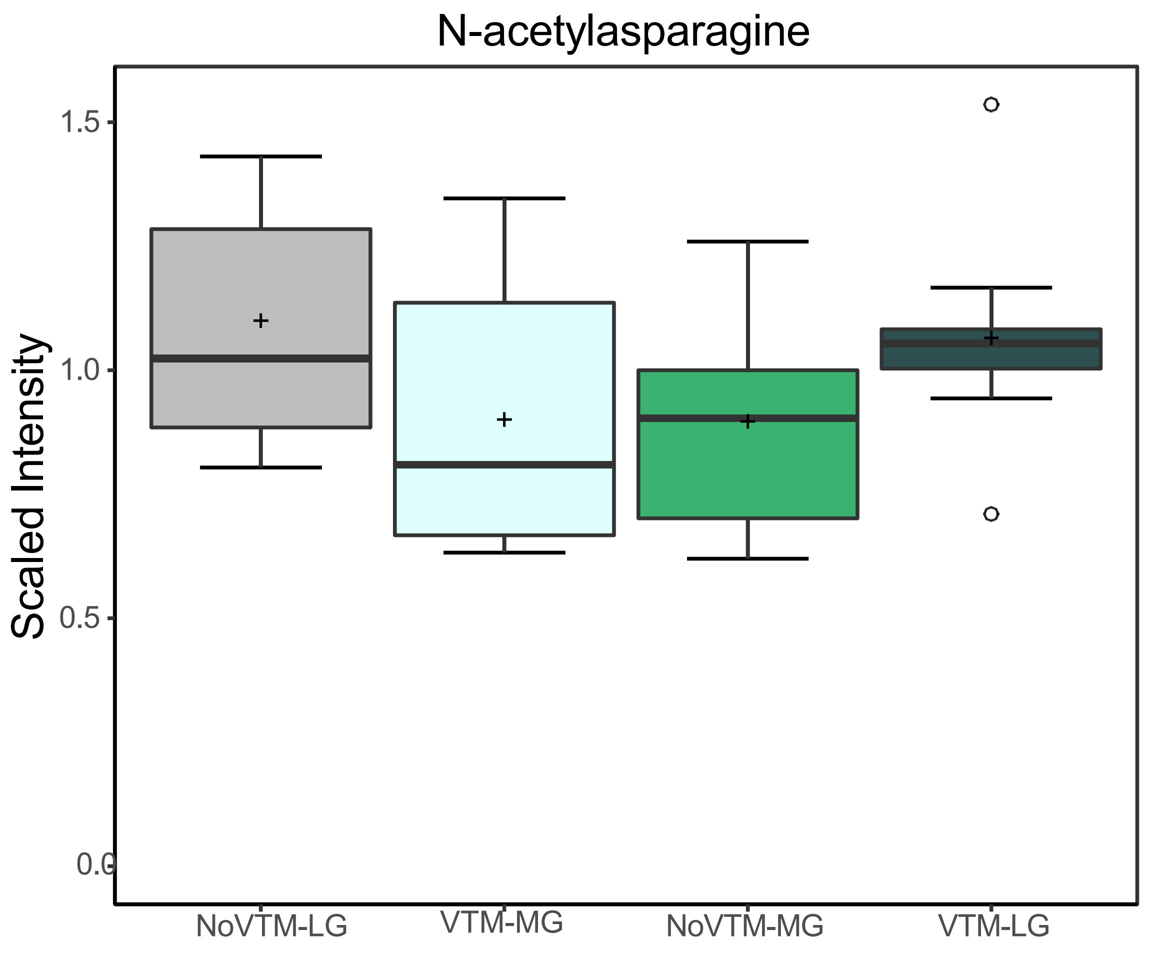
N-acetylasparagine
Scaled Intensity
1.5
1.0
0.5
0.0
NoVTM-LG
VTM-MG
NoVTM-MG
VTM-LG

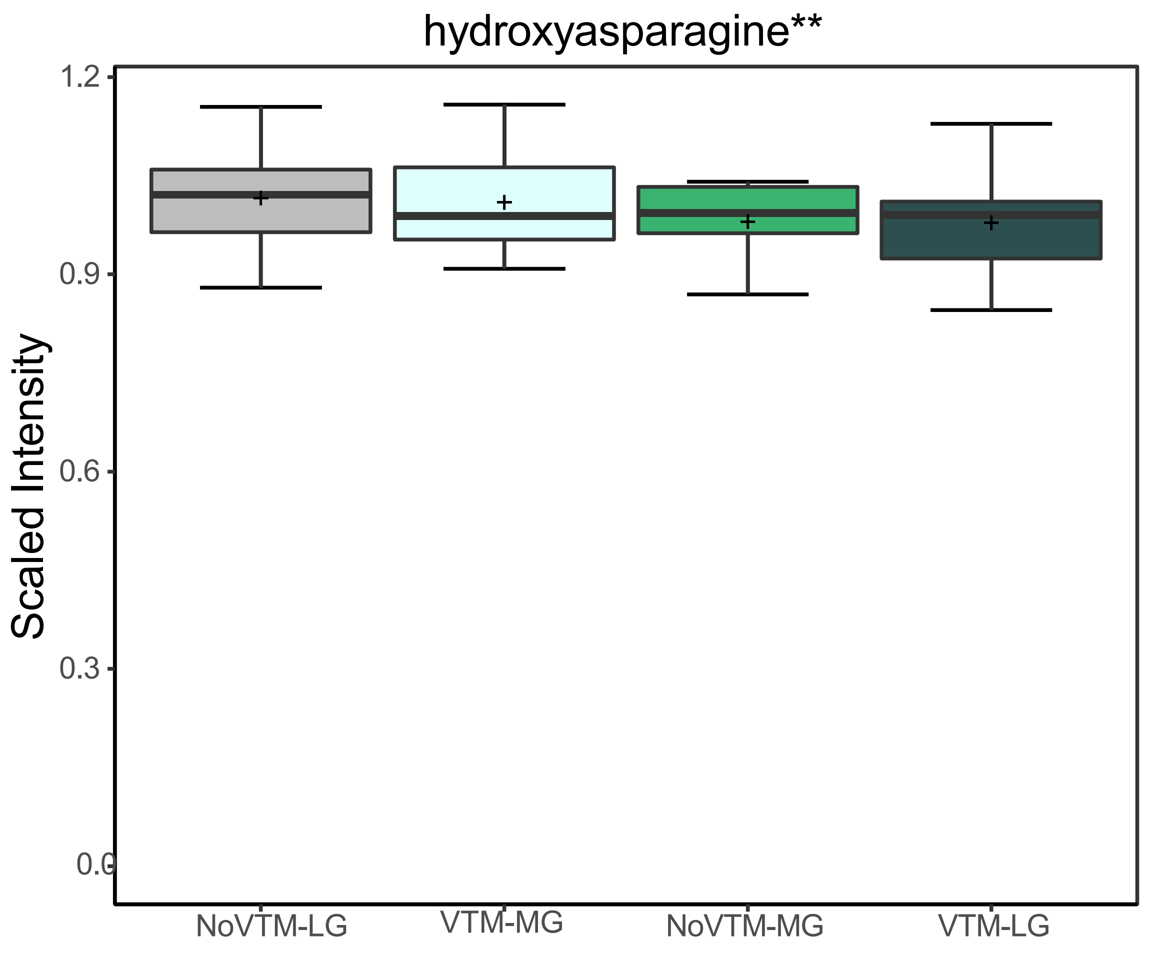
hydroxyasparagine**
Scaled Intensity
1.2
0.9
0.6
0.3
0.0
NoVTM-LG
VTM-MG
NoVTM-MG
VTM-LG

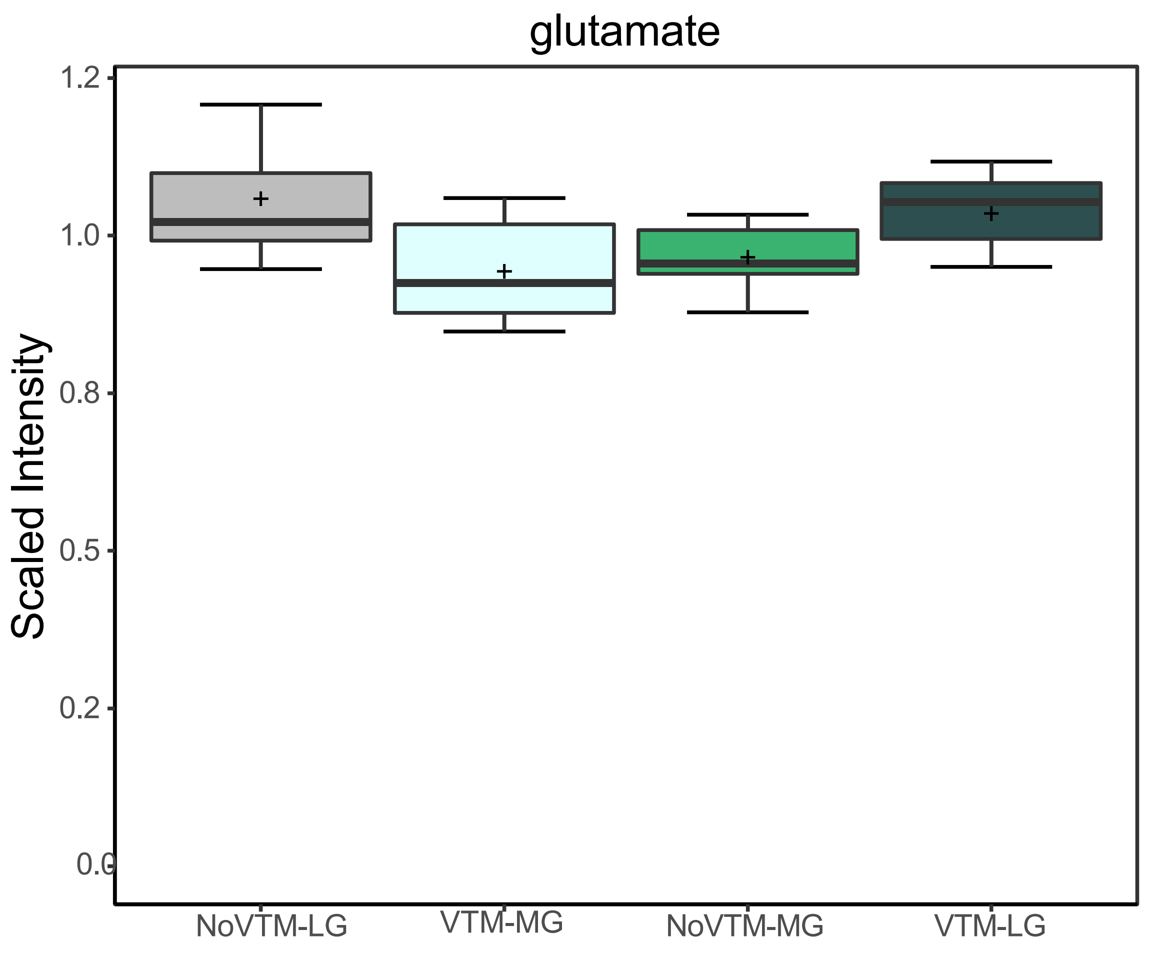
glutamate
Scaled Intensity
1.2
1.0
0.8
0.5
0.2
0.0
NoVTM-LG
VTM-MG
NoVTM-MG
VTM-LG

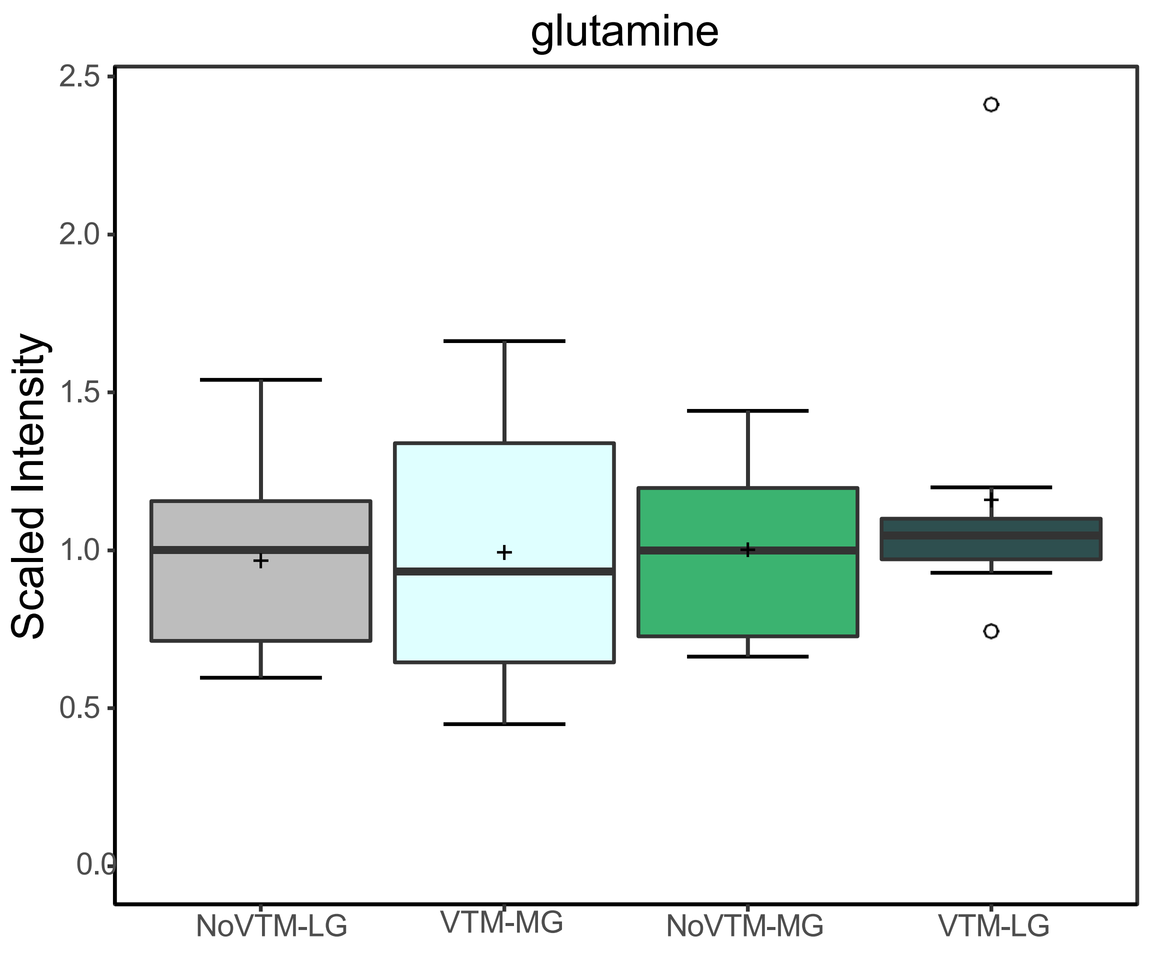

glutamine
Scaled Intensity
2.5
2.0
1.5
1.0
0.5
0.0
NoVTM-LG
VTM-MG
NoVTM-MG
VTM-LG

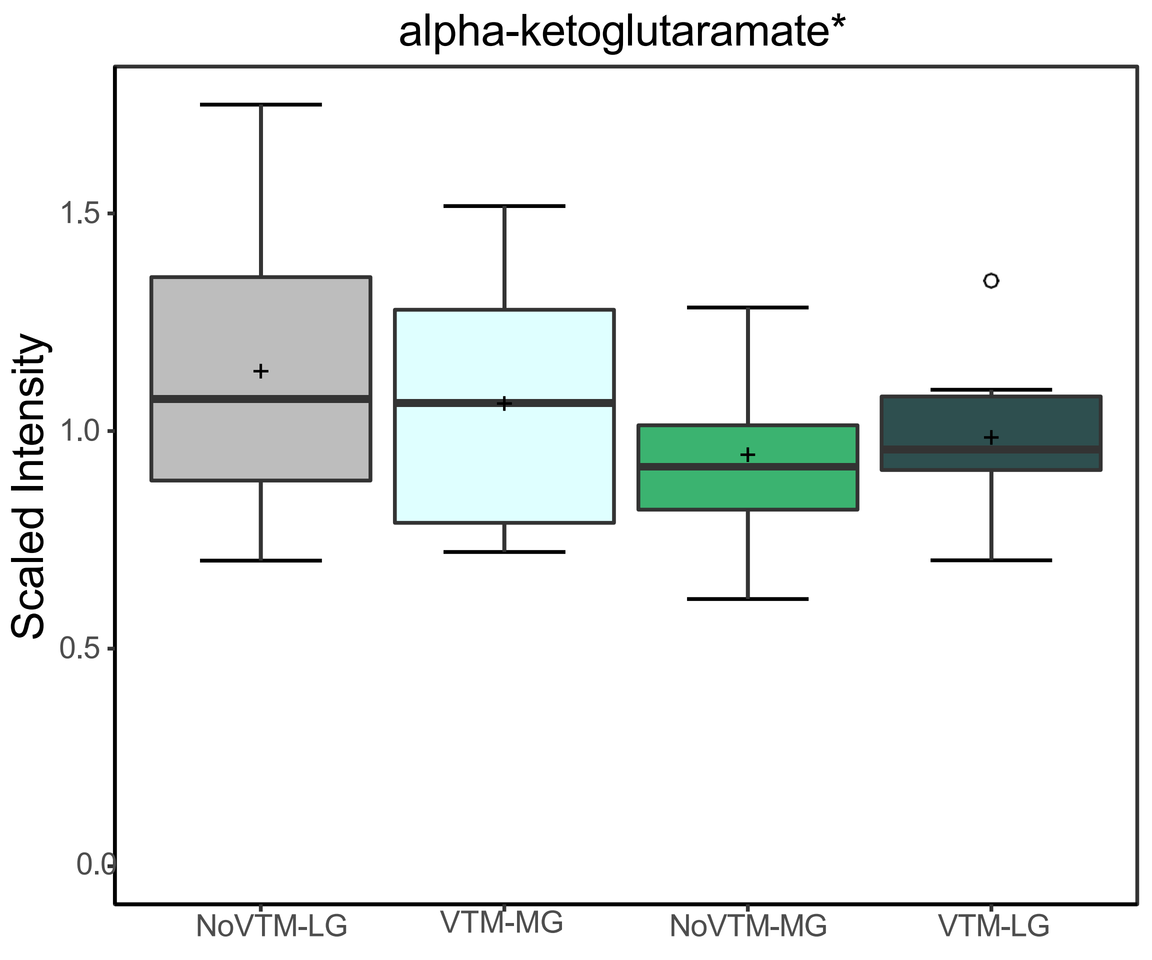
alpha-ketoglutaramate*
Scaled Intensity
1.5
1.0
0.5
0.0
NoVTM-LG
VTM-MG
NoVTM-MG
VTM-LG

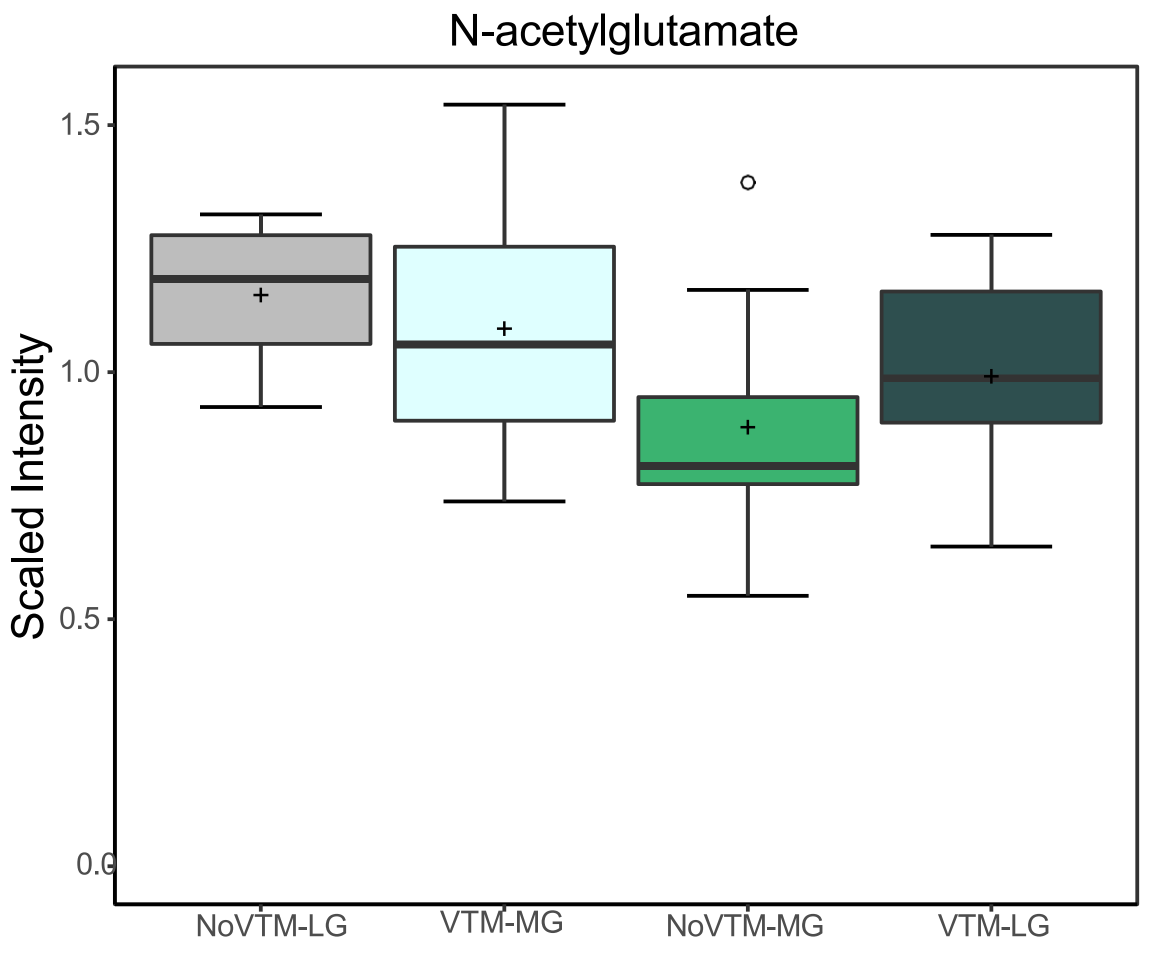

N-acetylglutamate
Scaled Intensity
1.5
1.0
0.5
0.0
NoVTM-LG
VTM-MG
NoVTM-MG
VTM-LG

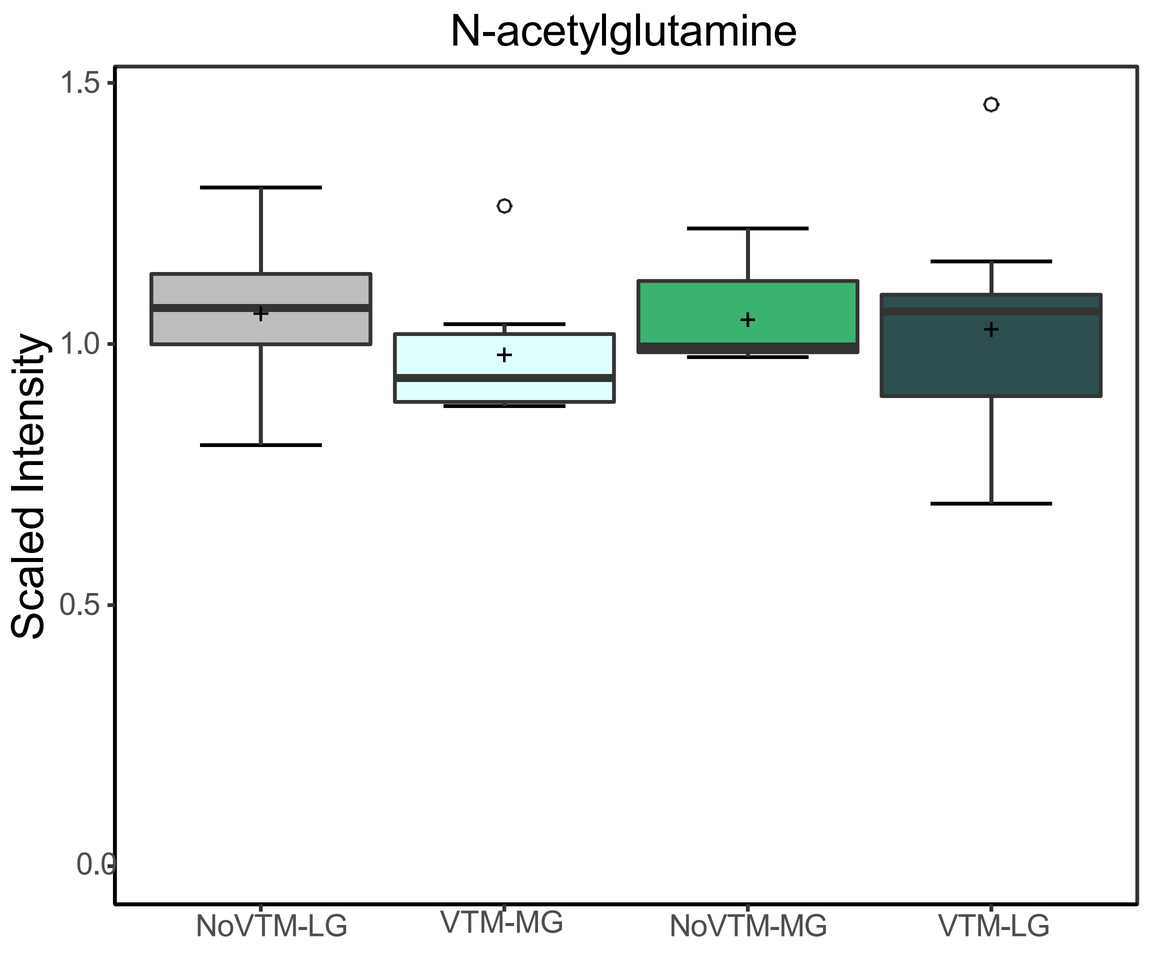

N-acetylglutamine
Scaled Intensity
1.5
1.0
0.5
0.0
NoVTM-LG
VTM-MG
NoVTM-MG
VTM-LG

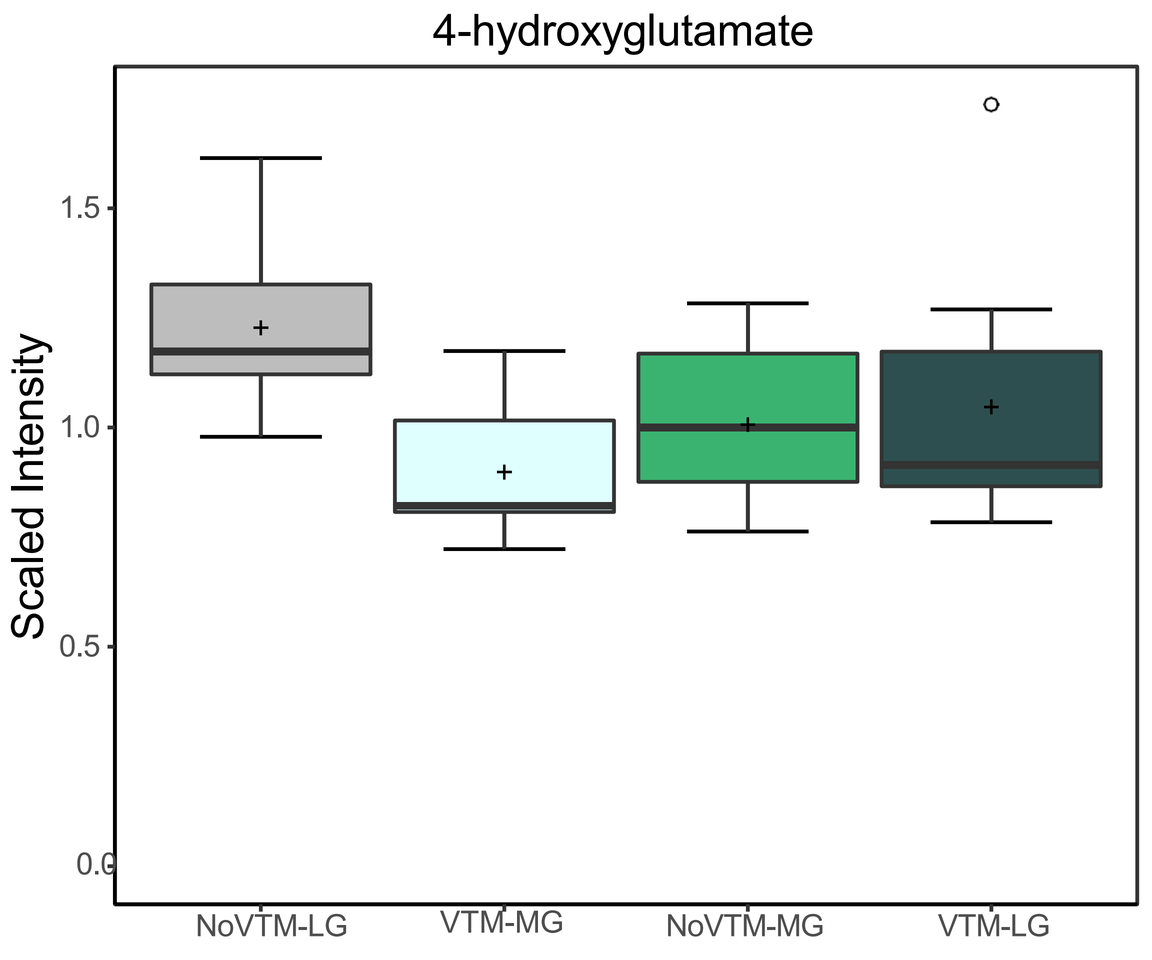
4-hydroxyglutamate
Scaled Intensity
1.5
1.0
0.5
0.0
NoVTM-LG
VTM-MG
NoVTM-MG
VTM-LG

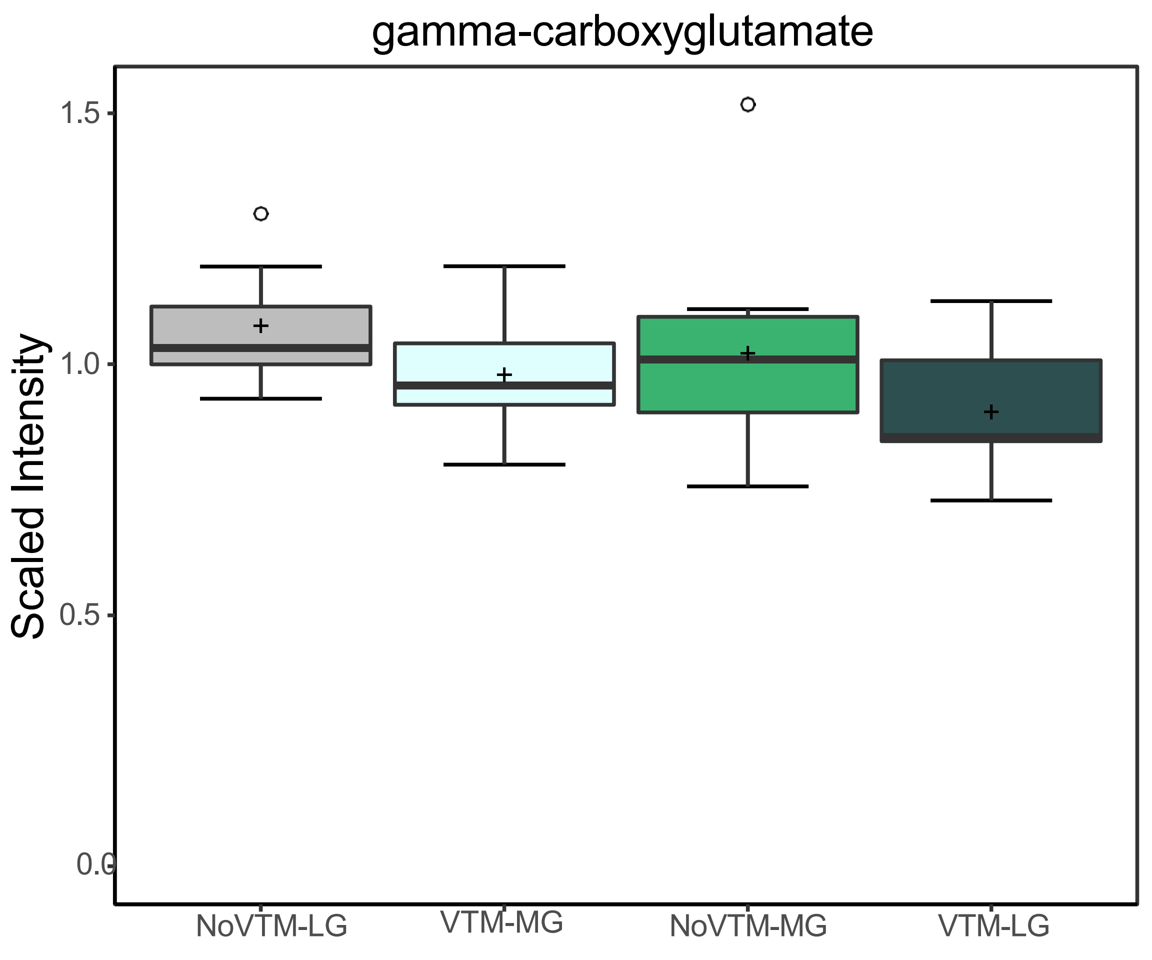
gamma-carboxyglutamate
Scaled Intensity
1.5
1.0
0.5
0.0
NoVTM-LG
VTM-MG
NoVTM-MG
VTM-LG

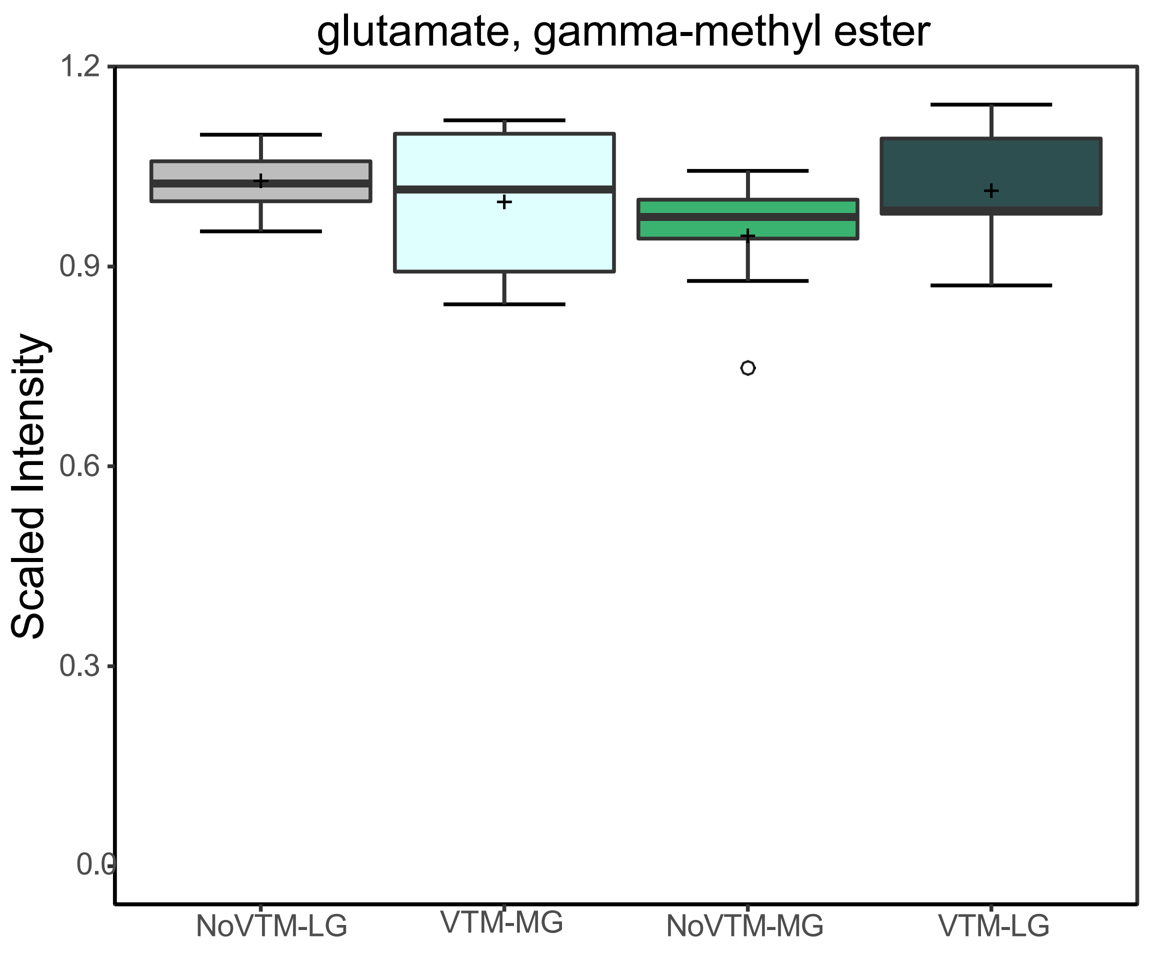
glutamate, gamma-methyl ester
Scaled Intensity
1.2
0.9
0.6
0.3
0.0
NoVTM-LG
VTM-MG
NoVTM-MG
VTM-LG

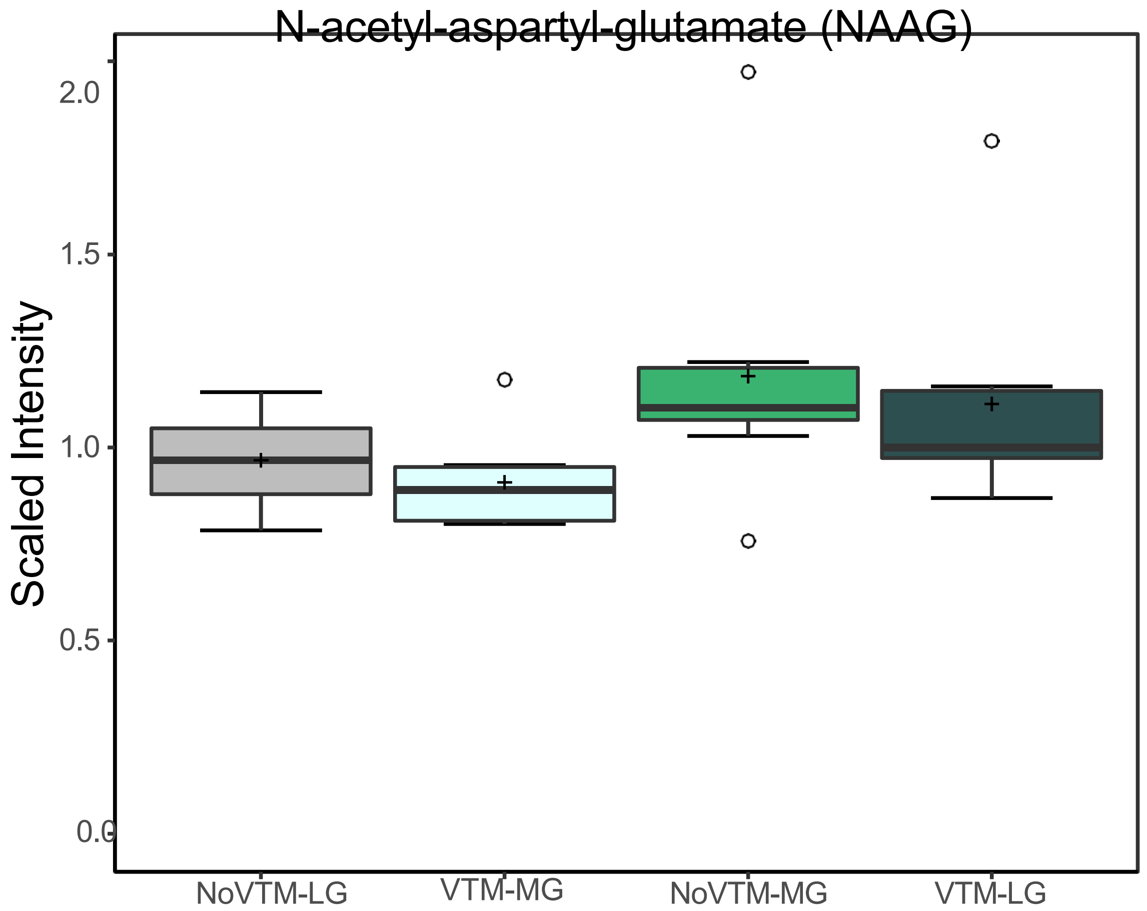
N-acetyl-aspartyl-glutamate (NAAG)
Scaled Intensity
2.0
1.5
1.0
0.5
0.0
NoVTM-LG
VTM-MG
NoVTM-MG
VTM-LG

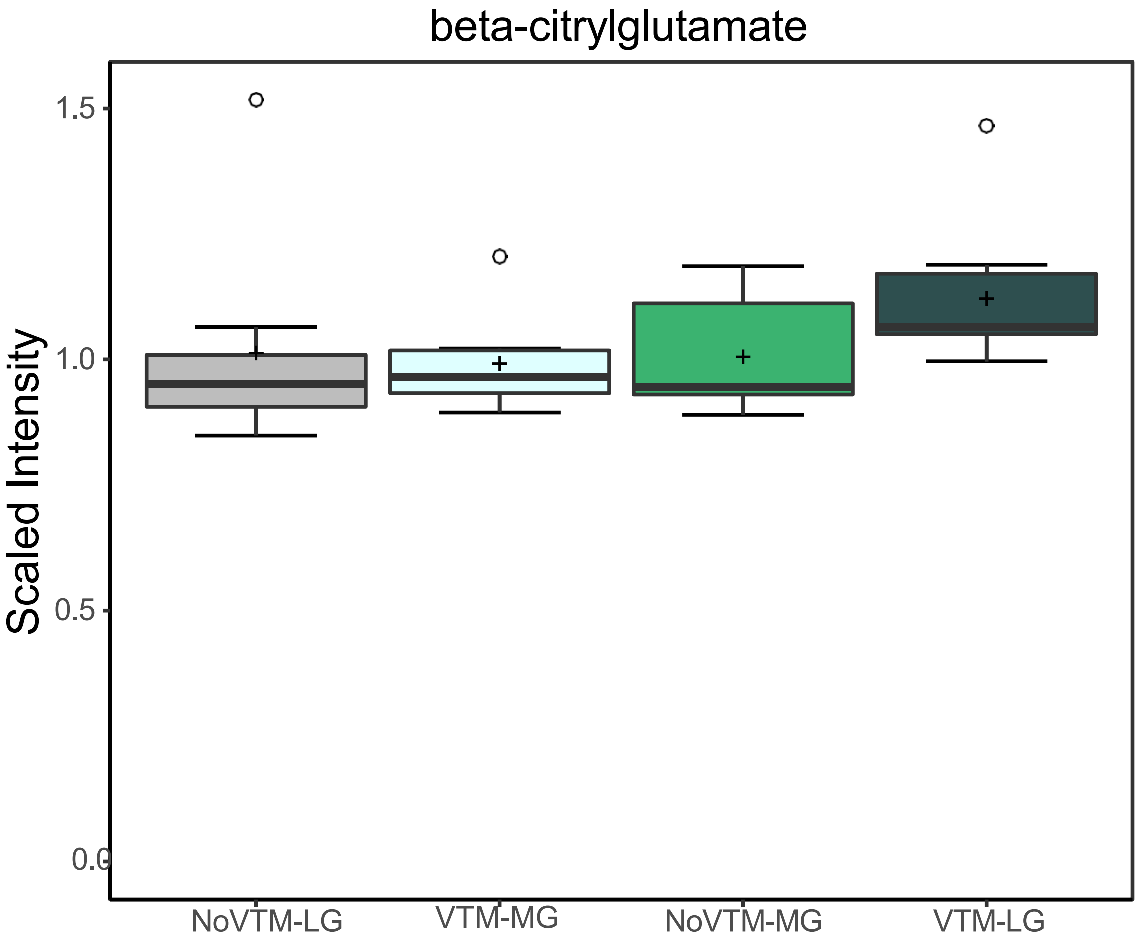

beta-citrylglutamate
Scaled Intensity
1.5
1.0
0.5
0.0
NoVTM-LG
VTM-MG
NoVTM-MG
VTM-LG

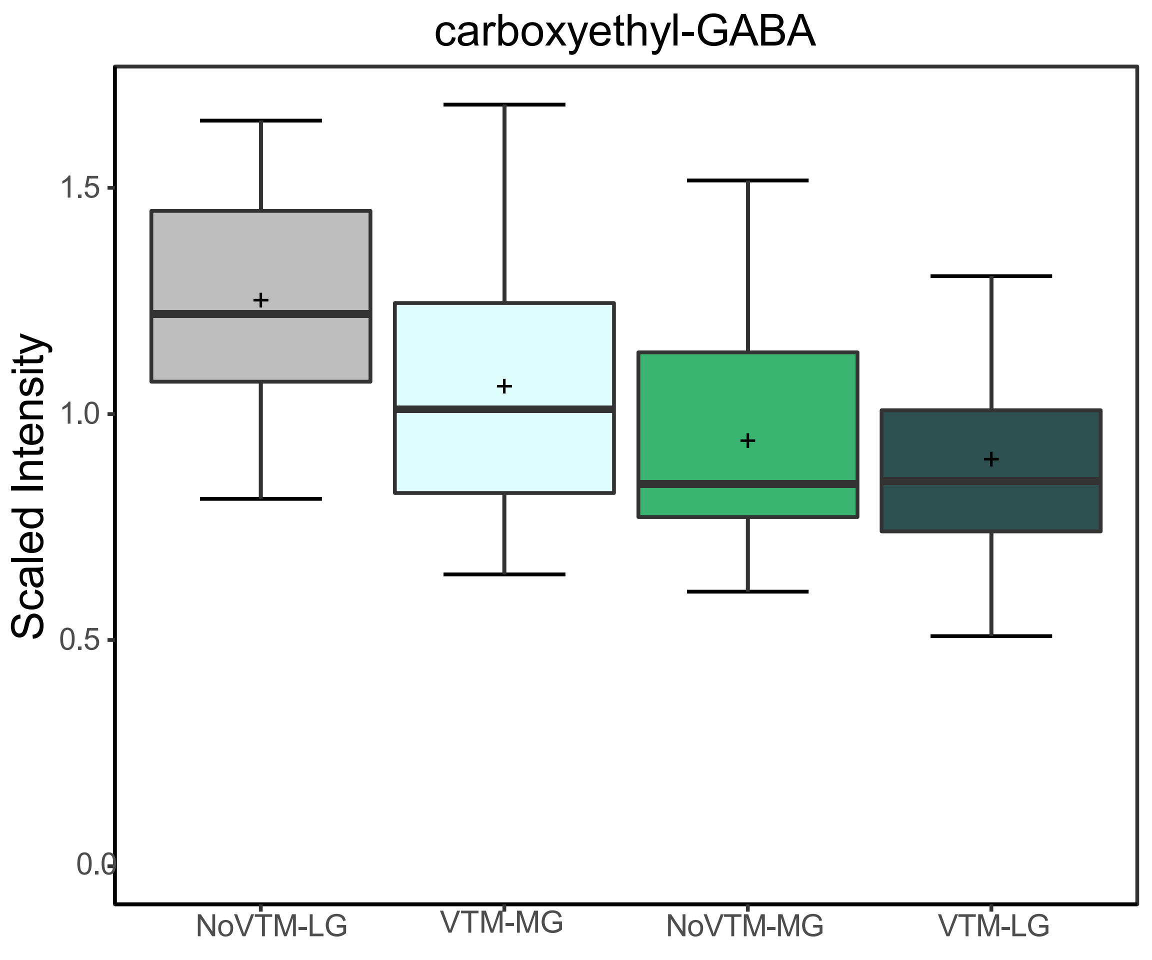
carboxyethyl-GABA
Scaled Intensity
1.5
1.0
0.5
0.0
NoVTM-LG
VTM-MG
NoVTM-MG
VTM-LG

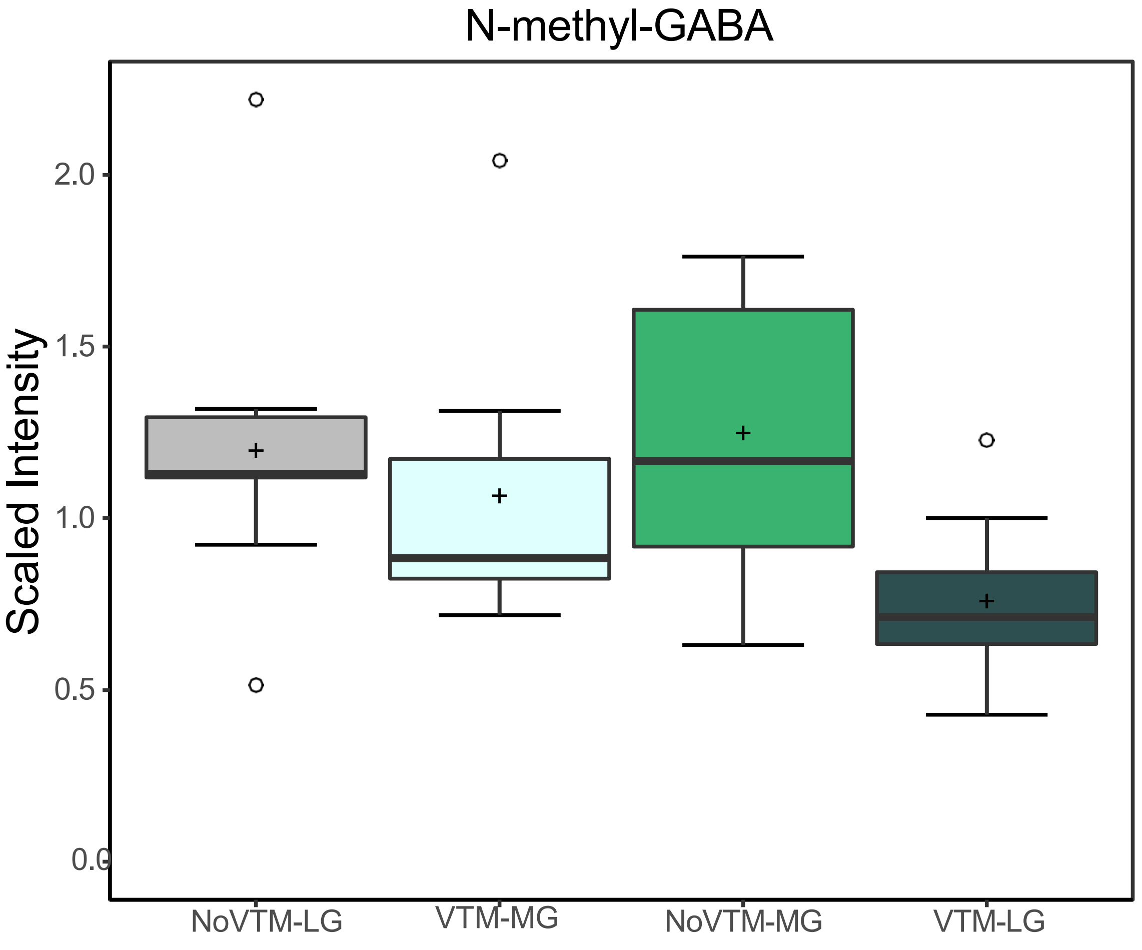

N-methyl-GABA
Scaled Intensity
2.0
1.5
1.0
0.5
0.0
NoVTM-LG
VTM-MG
NoVTM-MG
VTM-LG

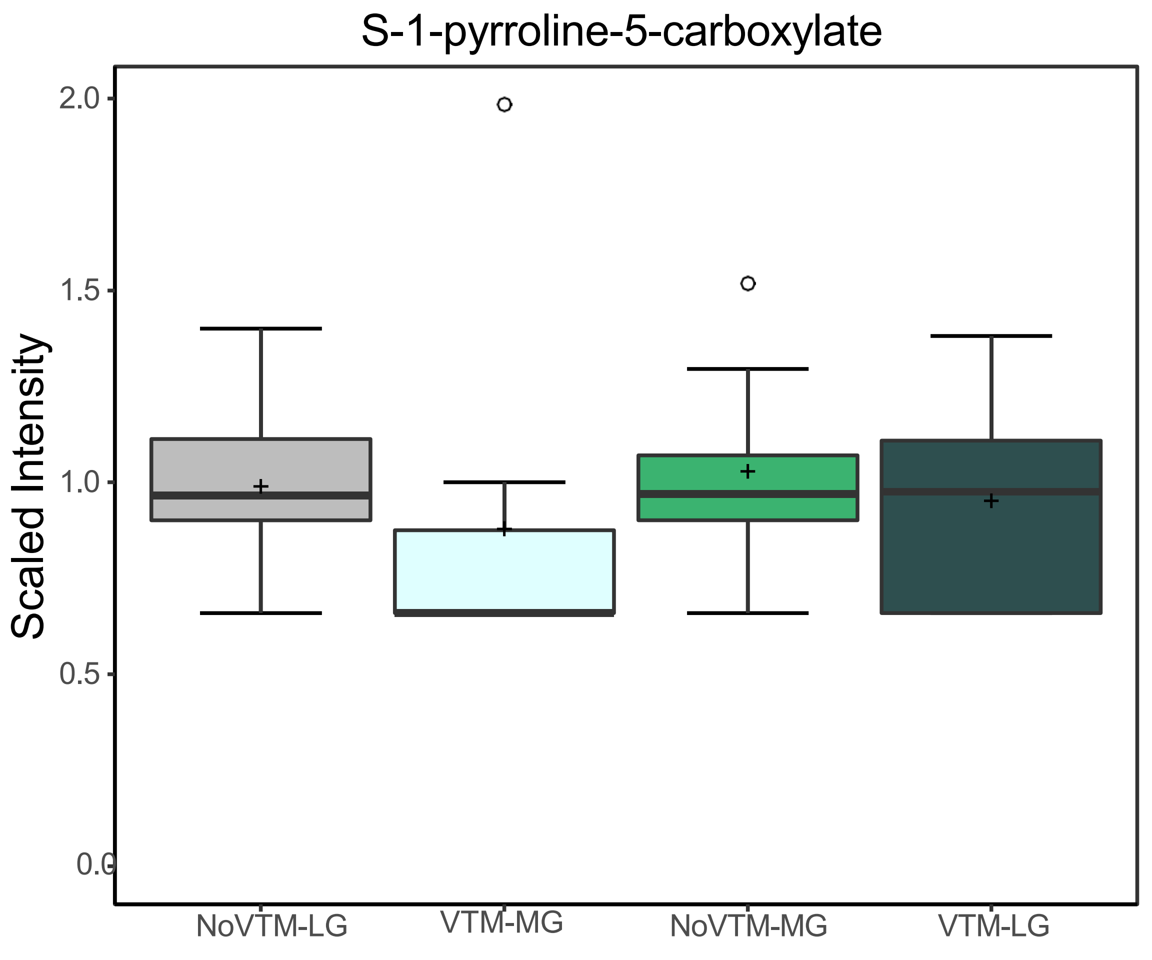
S-1-pyrroline-5-carboxylate
Scaled Intensity
2.0
1.5
1.0
0.5
0.0
NoVTM-LG
VTM-MG
NoVTM-MG
VTM-LG

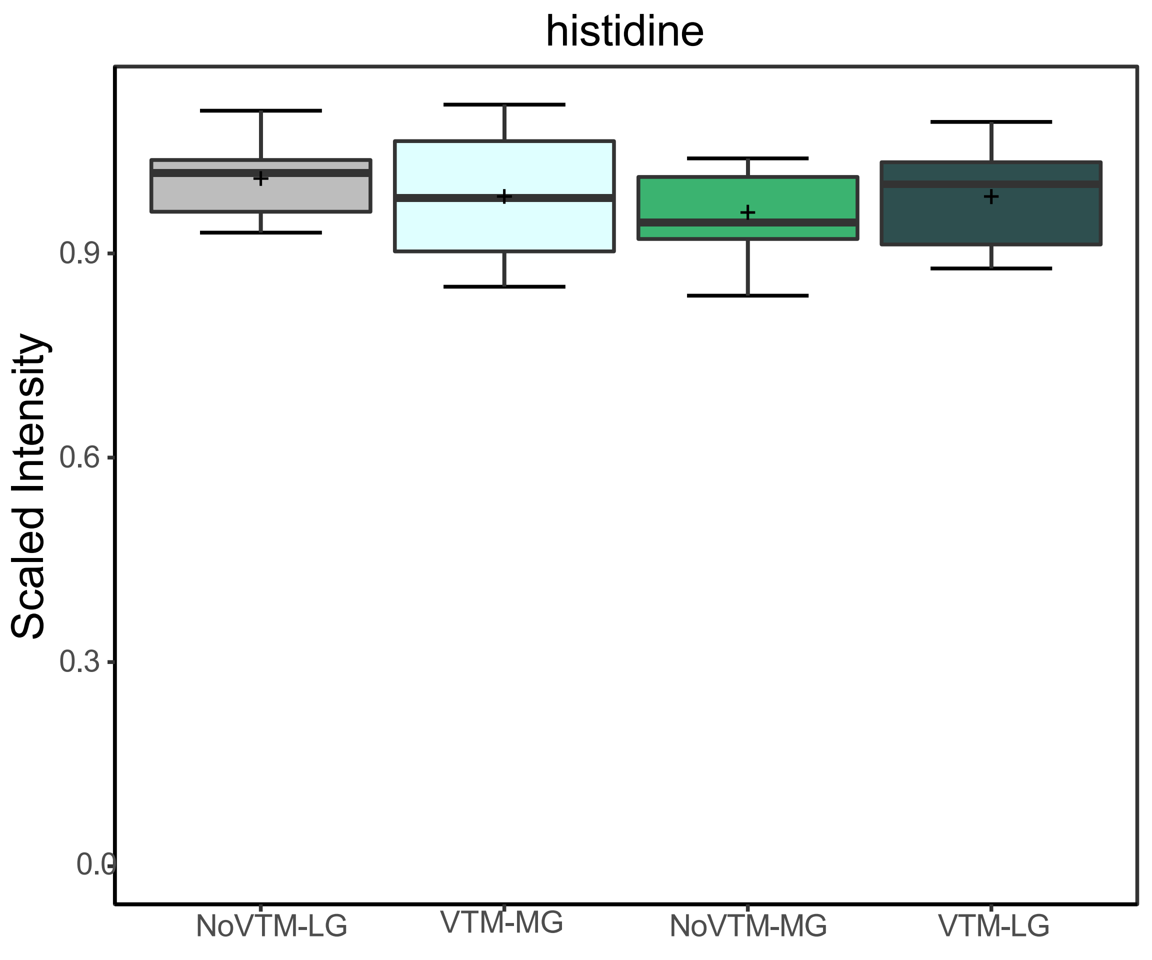
histidine
Scaled Intensity
0.0
0.3
0.6
0.9
NoVTM-LG
VTM-MG
NoVTM-MG
VTM-LG

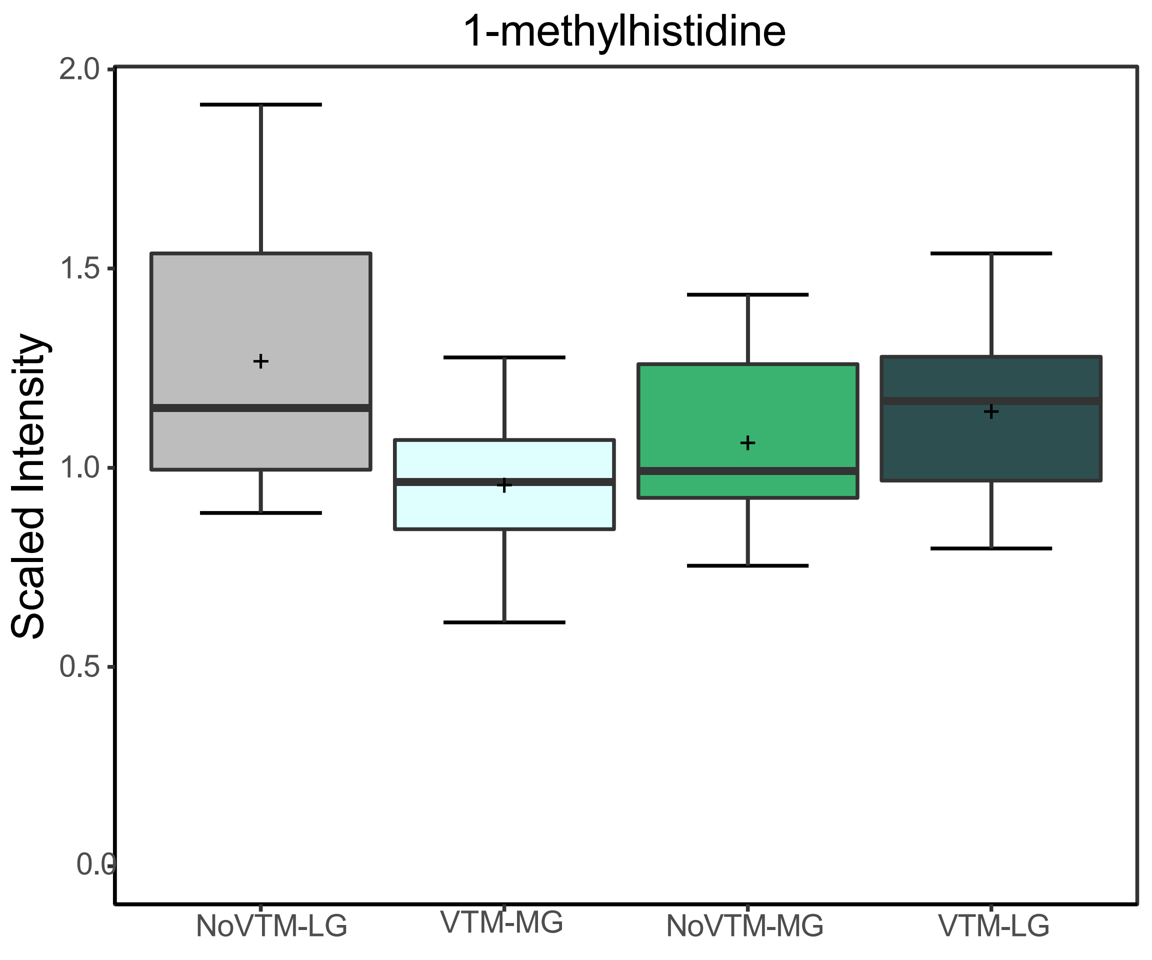

1-methylhistidine
Scaled Intensity
2.0
1.5
1.0
0.5
0.0
NoVTM-LG
VTM-MG
NoVTM-MG
VTM-LG

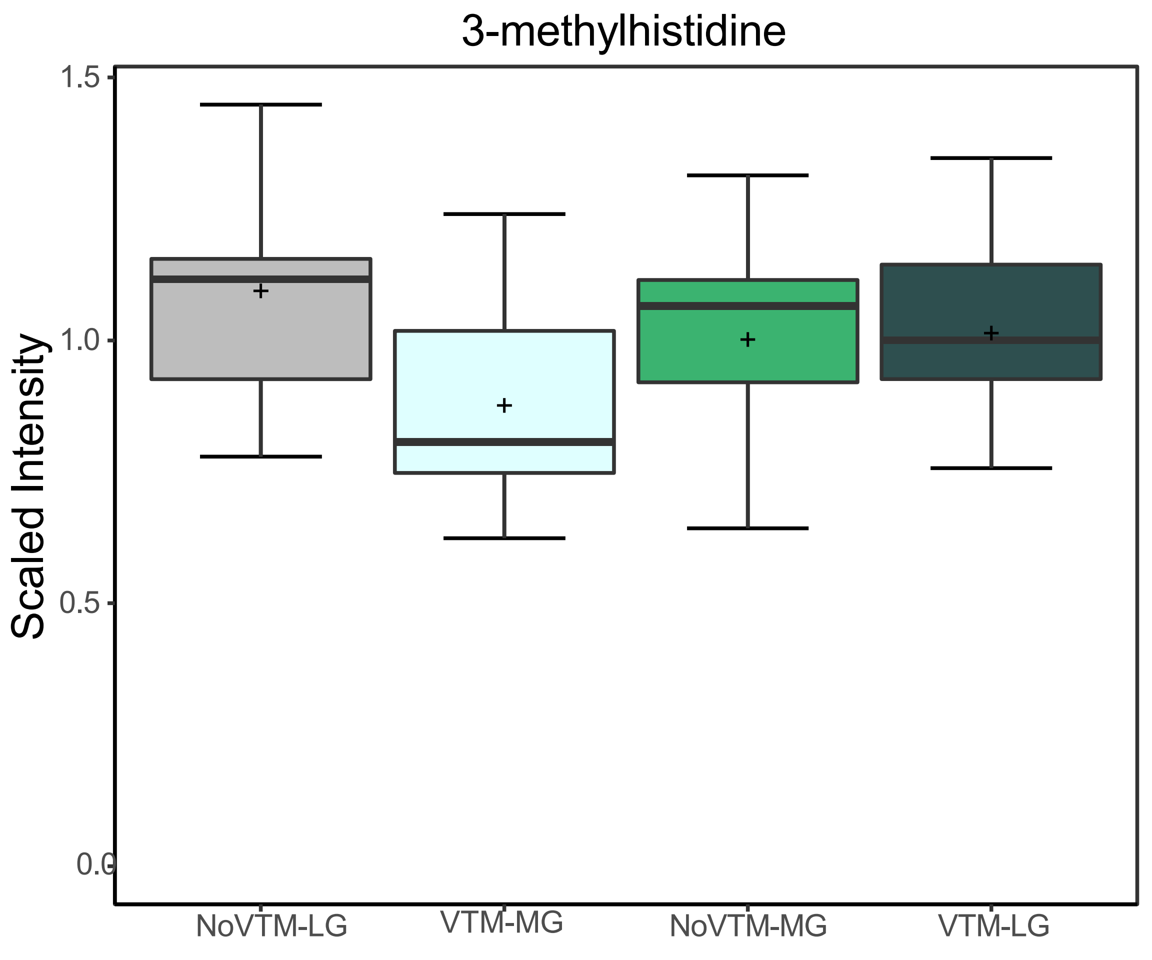
3-methylhistidine
Scaled Intensity
1.5
1.0
0.5
0.0
NoVTM-LG
VTM-MG
NoVTM-MG
VTM-LG

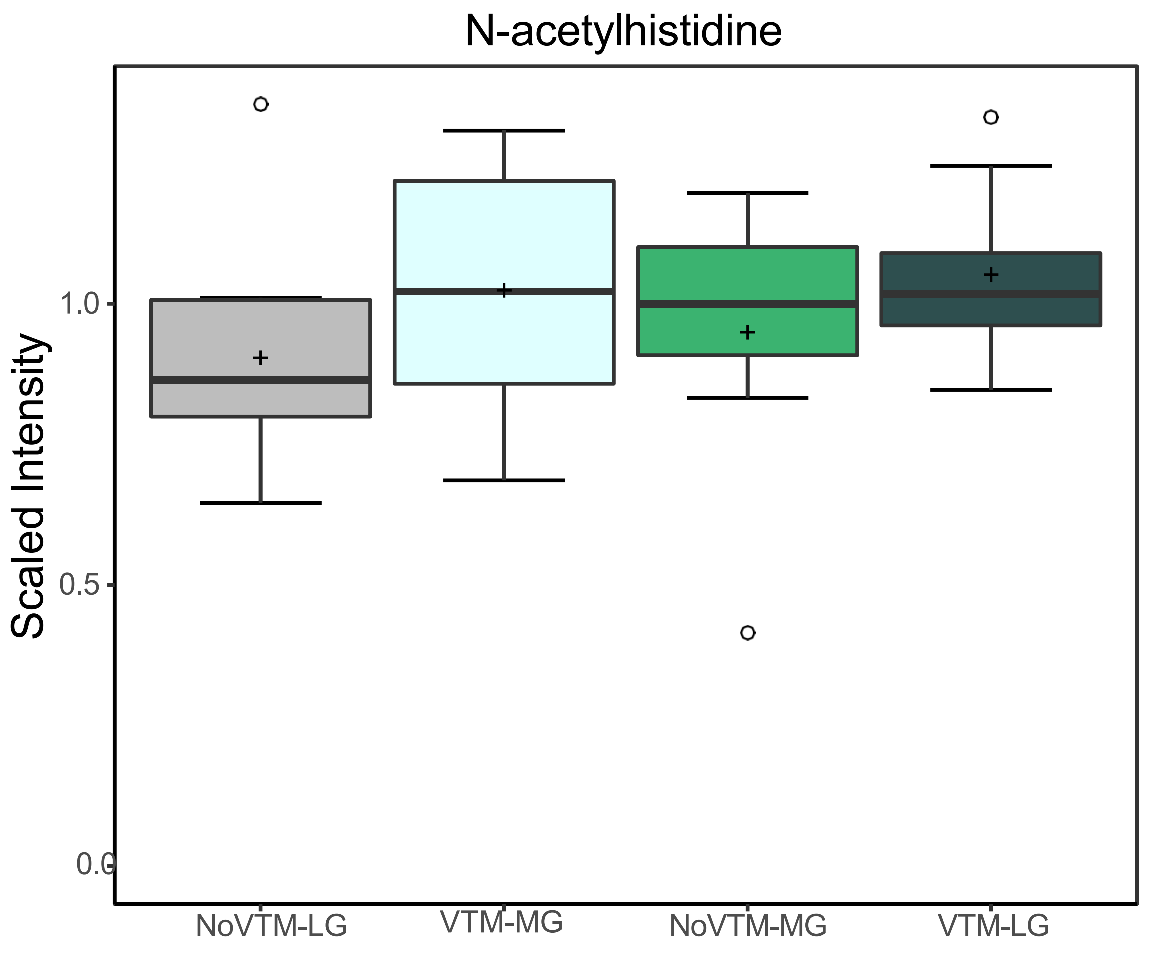
N-acetylhistidine
Scaled Intensity
1.0
0.5
0.0
NoVTM-LG
VTM-MG
NoVTM-MG
VTM-LG

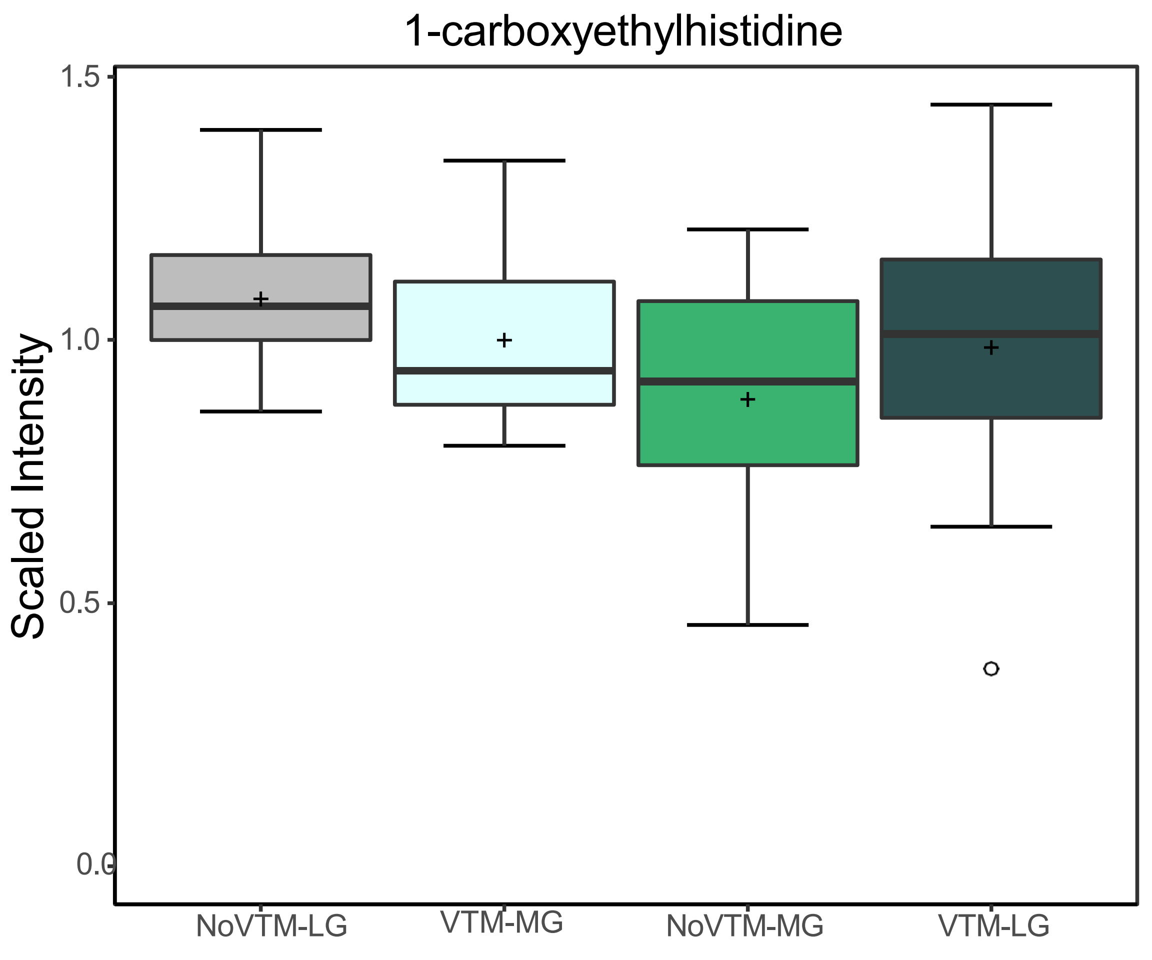
1-carboxyethylhistidine
Scaled Intensity
1.5
1.0
0.5
0.0
NoVTM-LG
VTM-MG
NoVTM-MG
VTM-LG

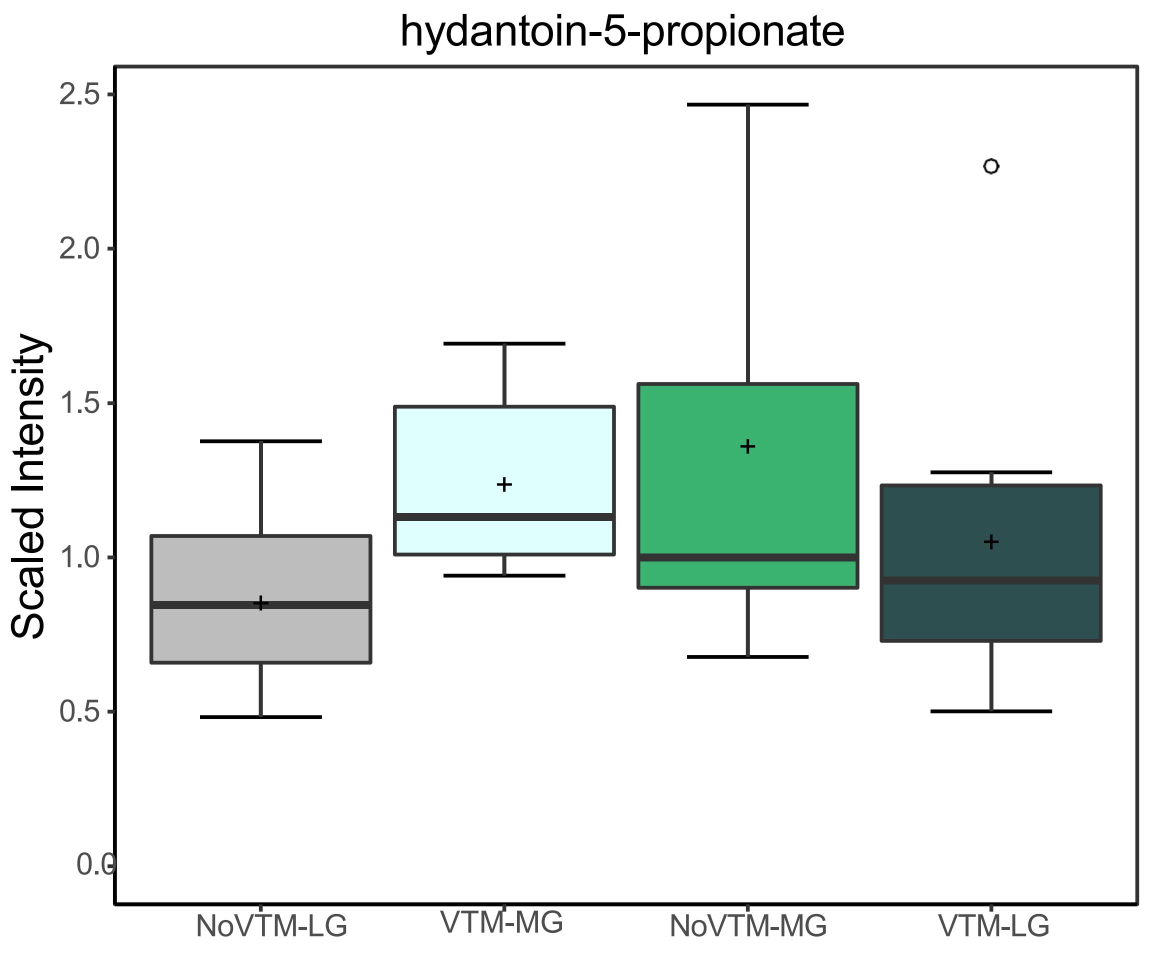
hydantoin-5-propionate
Scaled Intensity
2.5
2.0
1.5
1.0
0.5
0.0
NoVTM-LG
VTM-MG
NoVTM-MG
VTM-LG

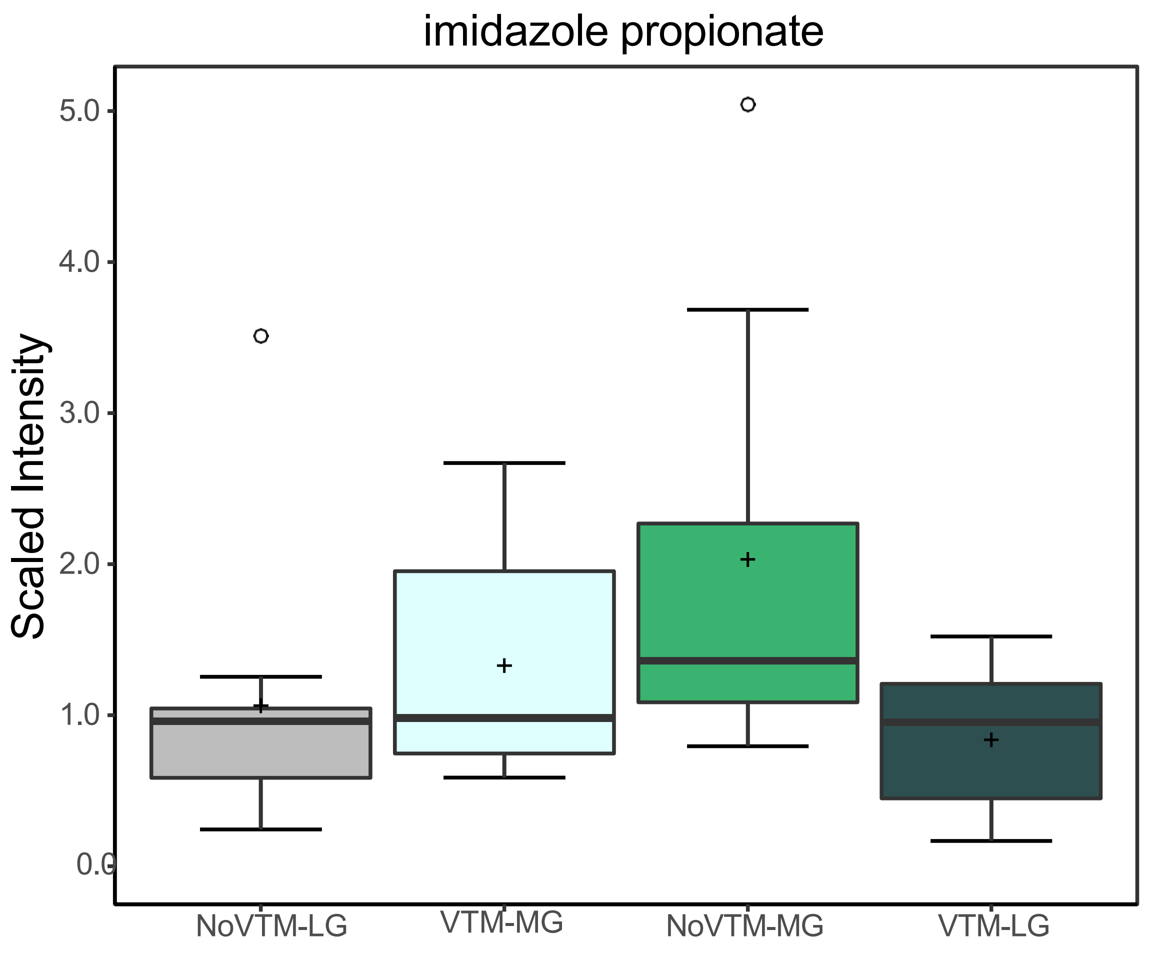
imidazole propionate
Scaled Intensity
5.0
4.0
3.0
2.0
1.0
0.0
NoVTM-LG
VTM-MG
NoVTM-MG
VTM-LG

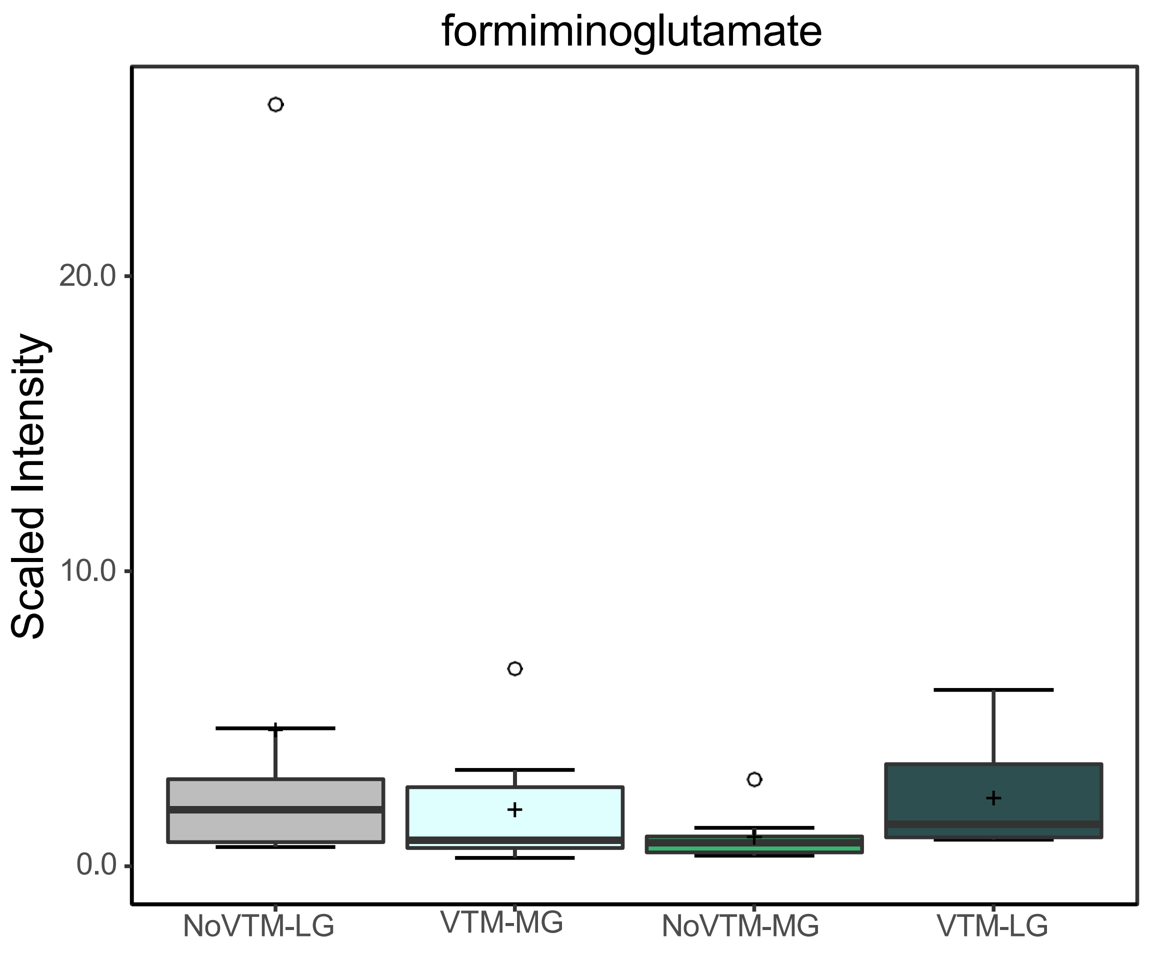

formiminoglutamate
Scaled Intensity
20.0
10.0
0.0
NoVTM-LG
VTM-MG
NoVTM-MG
VTM-LG

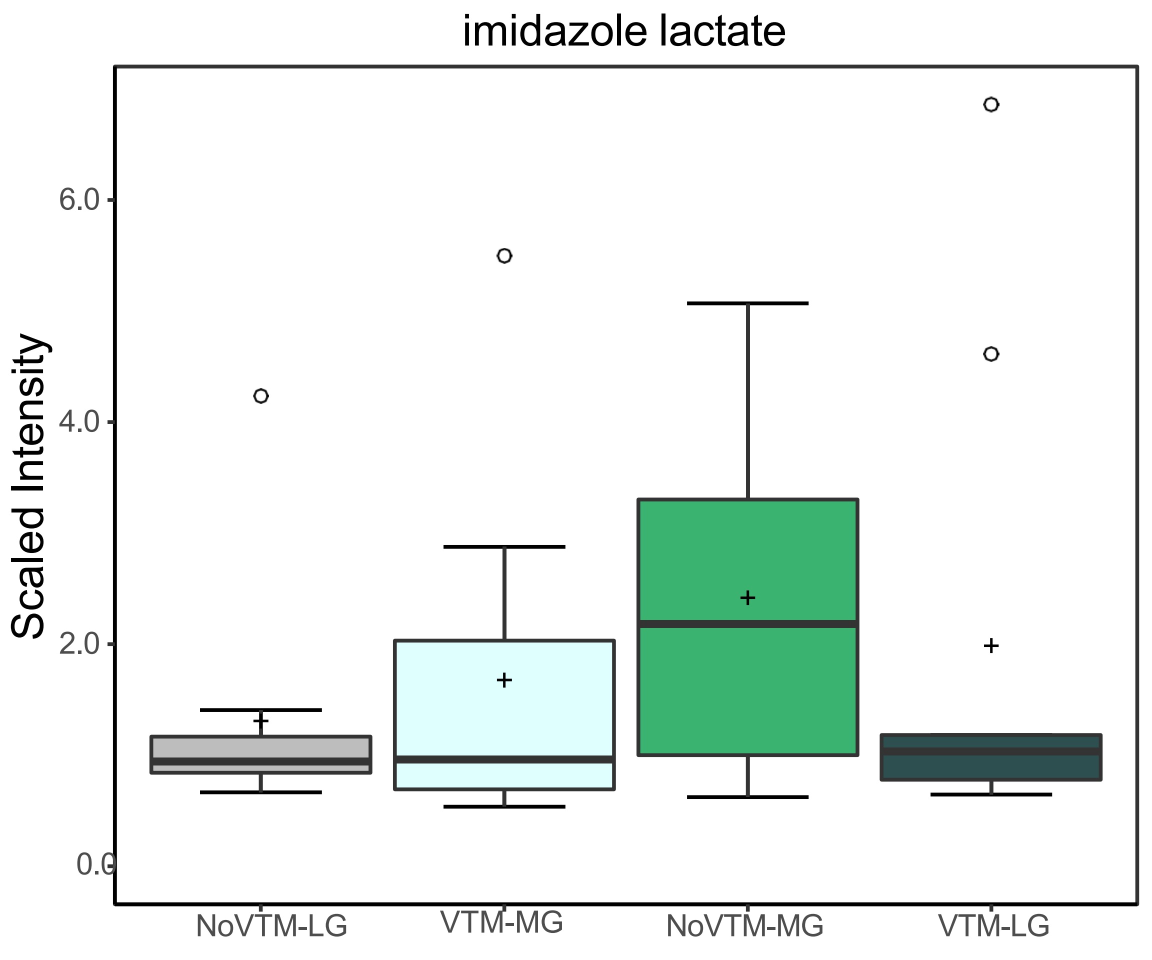
imidazole lactate
Scaled Intensity
6.0
4.0
2.0
0.0
NoVTM-LG
VTM-MG
NoVTM-MG
VTM-LG

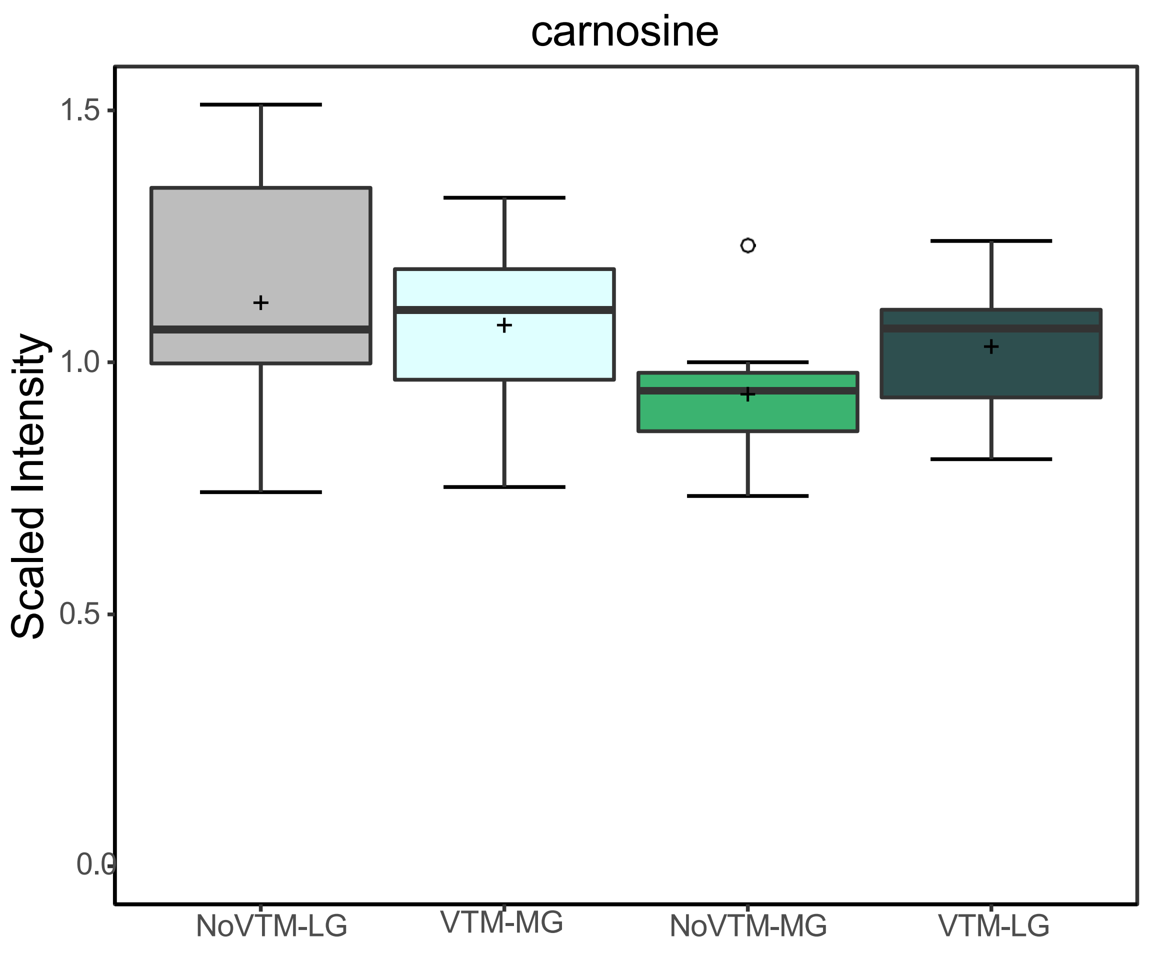
carnosine
Scaled Intensity
1.5
1.0
0.5
0.0
NoVTM-LG
VTM-MG
NoVTM-MG
VTM-LG

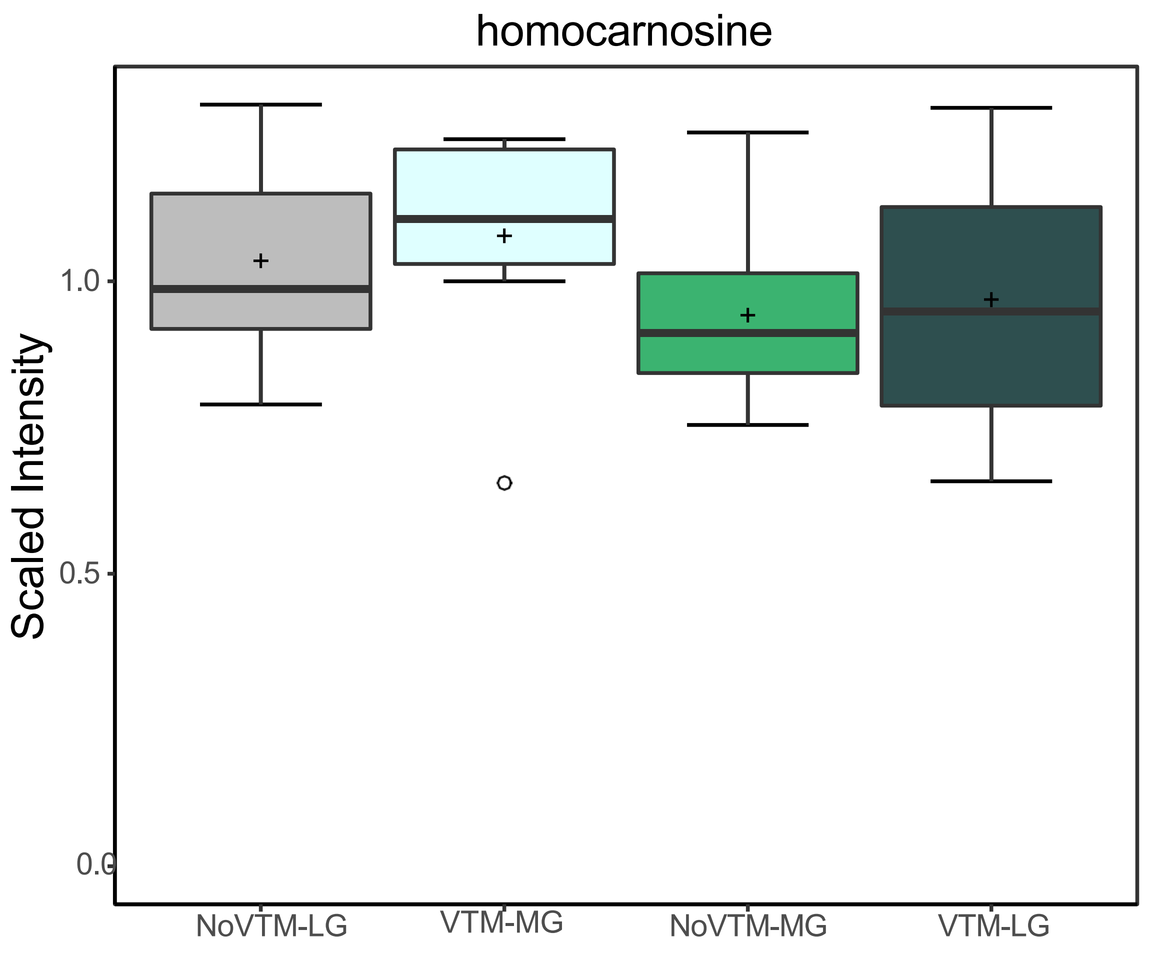
homocarnosine
Scaled Intensity
1.0
0.5
0.0
NoVTM-LG
VTM-MG
NoVTM-MG
VTM-LG

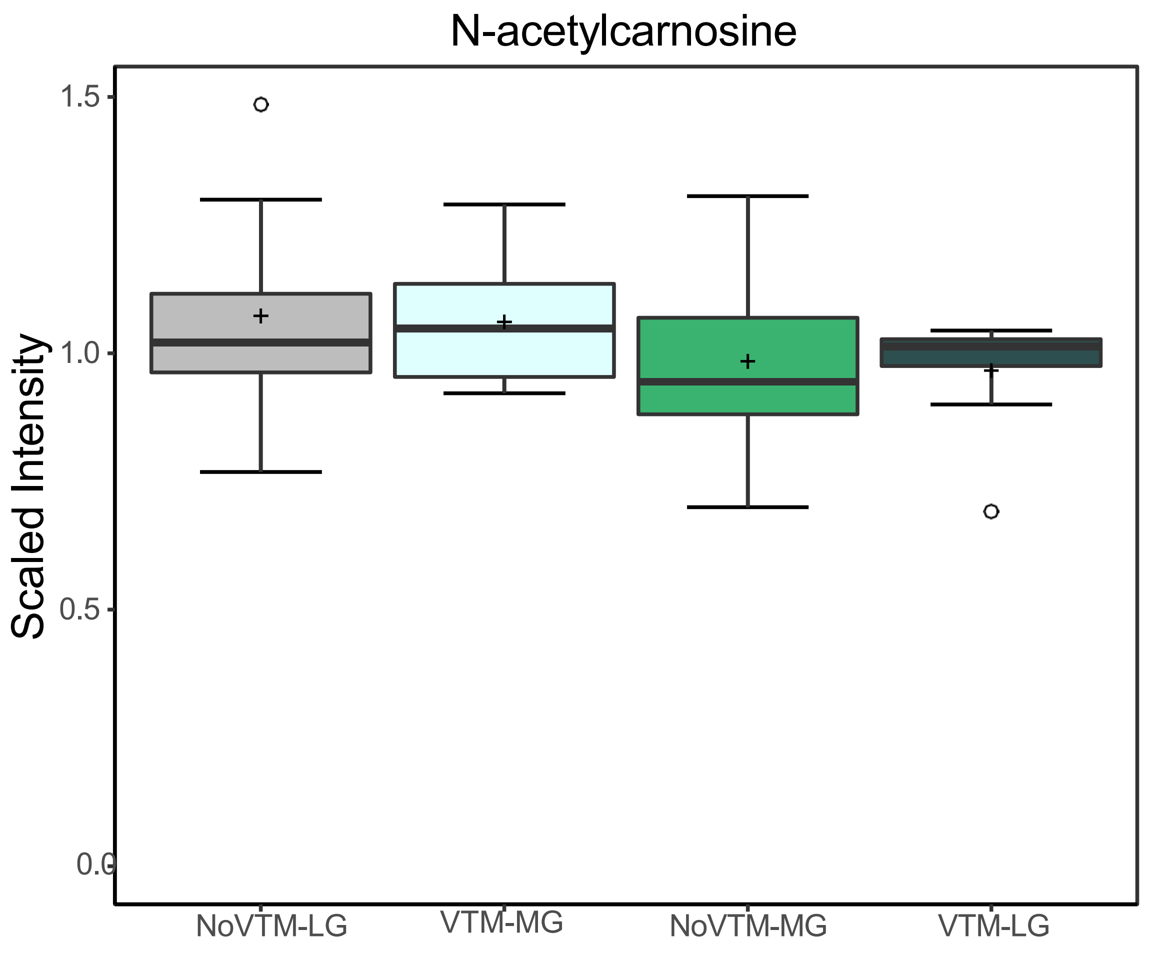
N-acetylcarnosine
Scaled Intensity
1.5
1.0
0.5
0.0
NoVTM-LG
VTM-MG
NoVTM-MG
VTM-LG

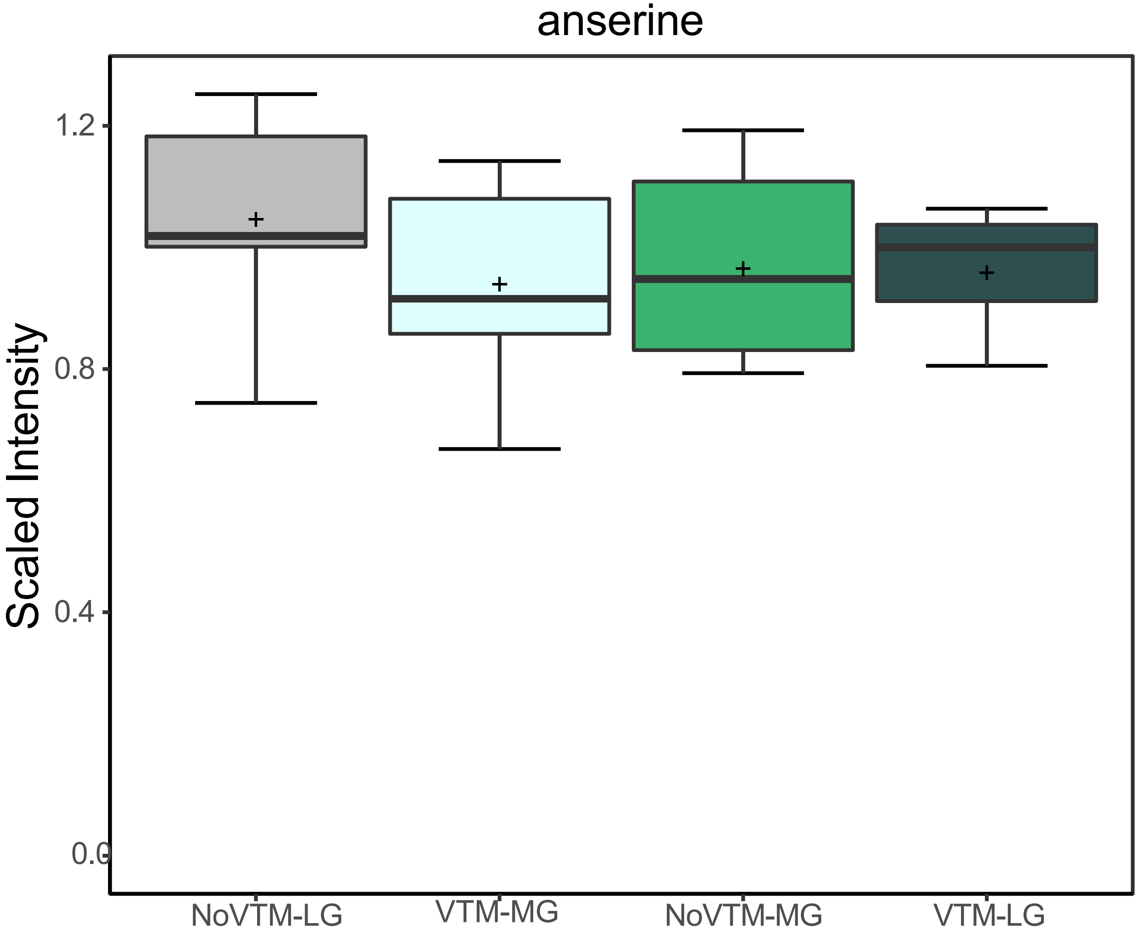
anserine
Scaled Intensity
1.2
0.8
0.4
0.0
NoVTM-LG
VTM-MG
NoVTM-MG
VTM-LG

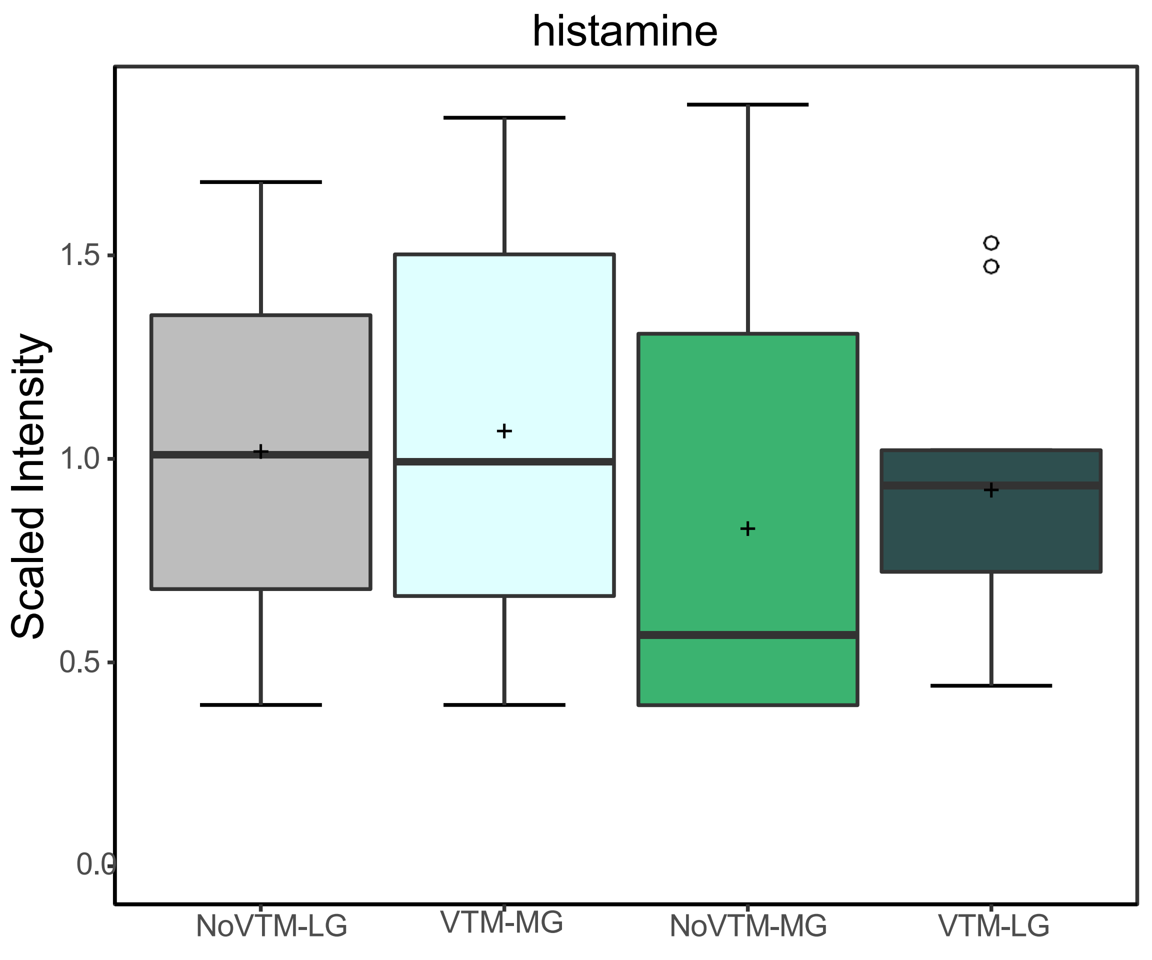
histamine
Scaled Intensity
1.5
1.0
0.5
0.0
NoVTM-LG
VTM-MG
NoVTM-MG
VTM-LG

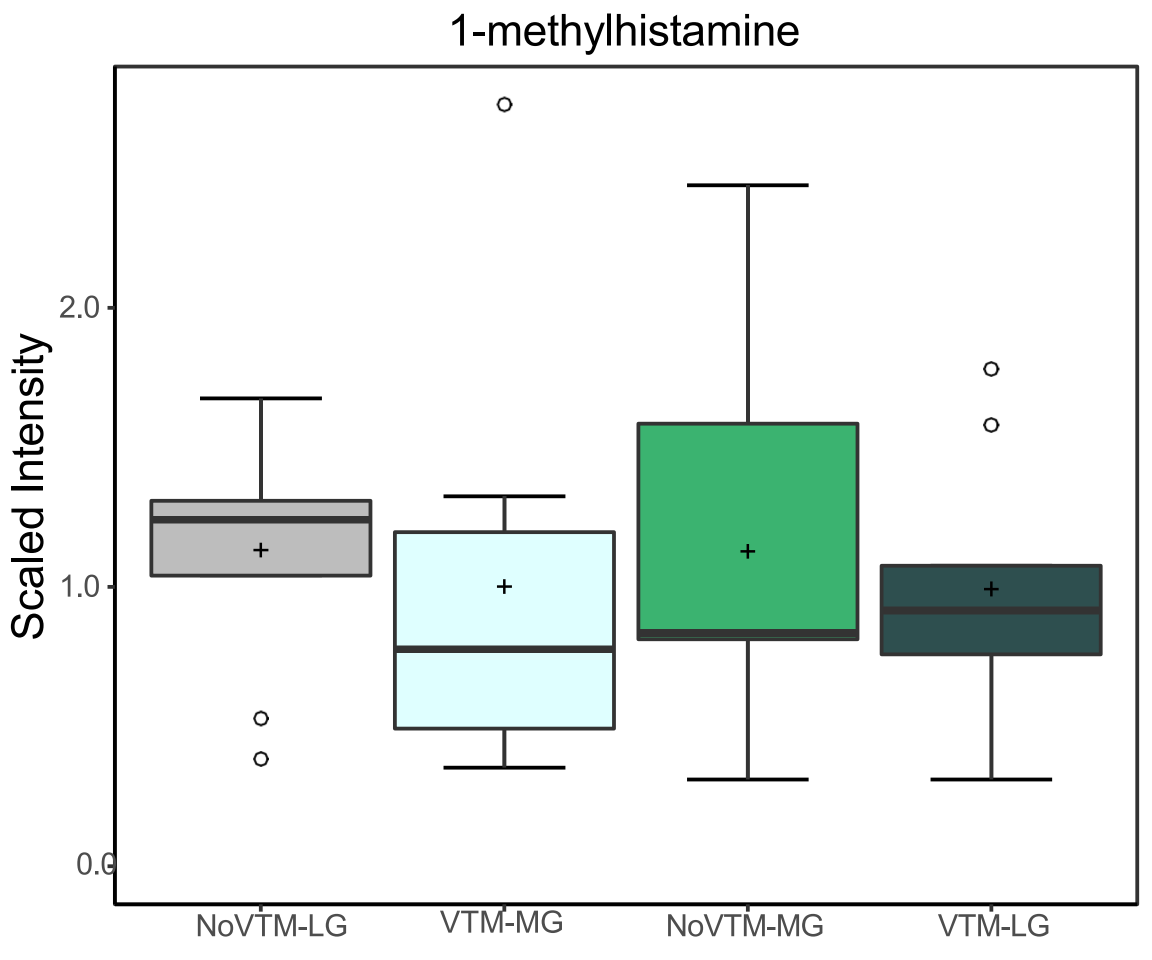
1-methylhistamine
Scaled Intensity
2.0
1.0
0.0
NoVTM-LG
VTM-MG
NoVTM-MG
VTM-LG

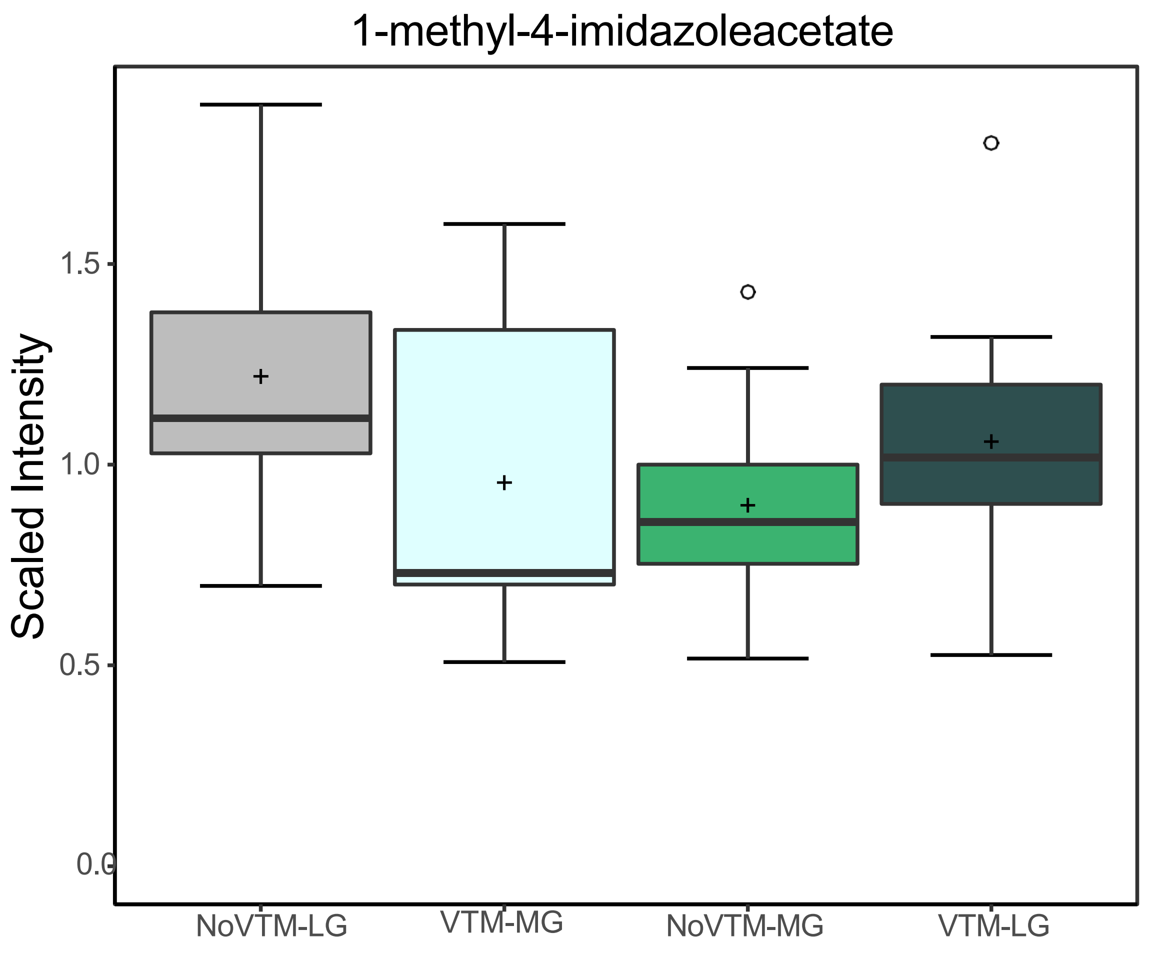

1-methyl-4-imidazoleacetate
Scaled Intensity
1.5
1.0
0.5
0.0
NoVTM-LG
VTM-MG
NoVTM-MG
VTM-LG

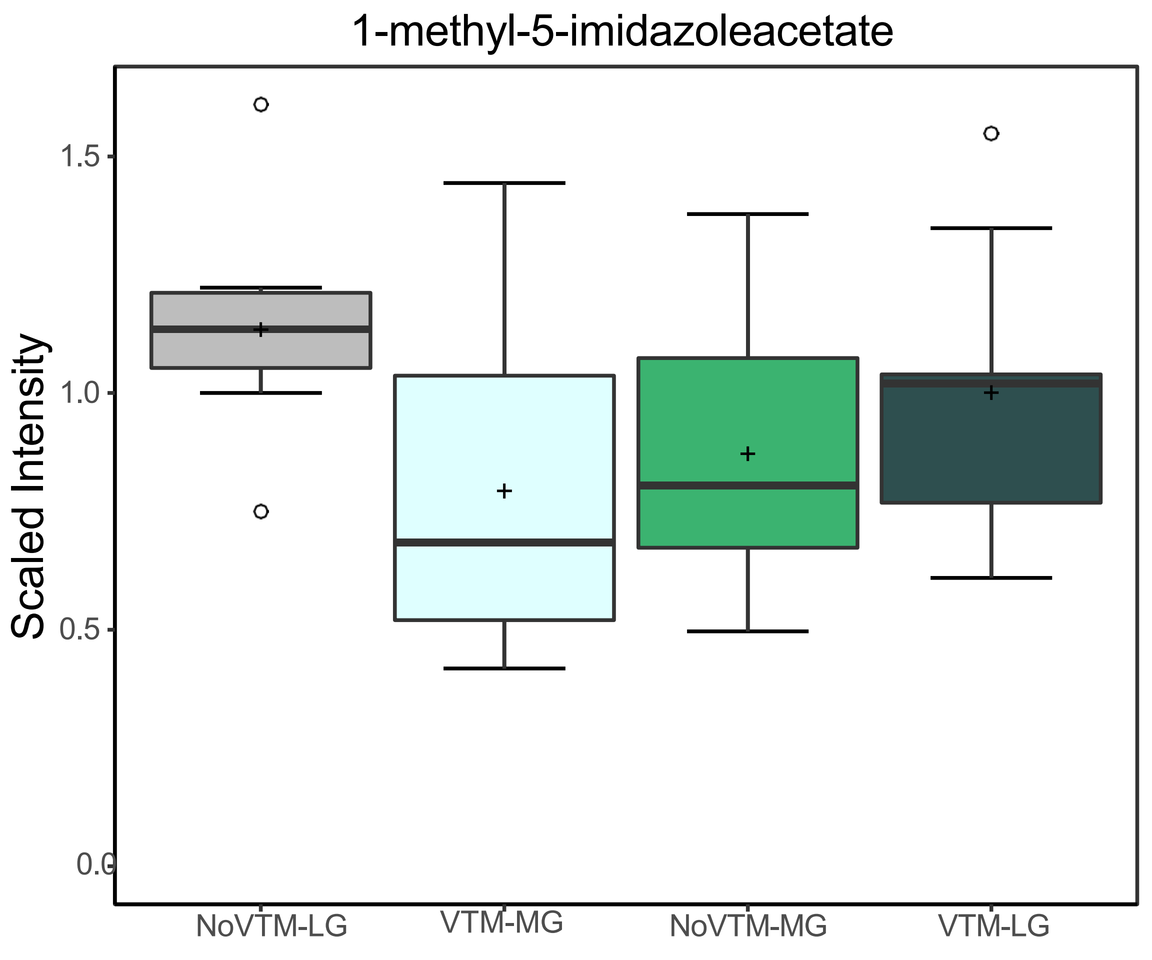
1-methyl-5-imidazoleacetate
Scaled Intensity
1.5
1.0
0.5
0.0
NoVTM-LG
VTM-MG
NoVTM-MG
VTM-LG

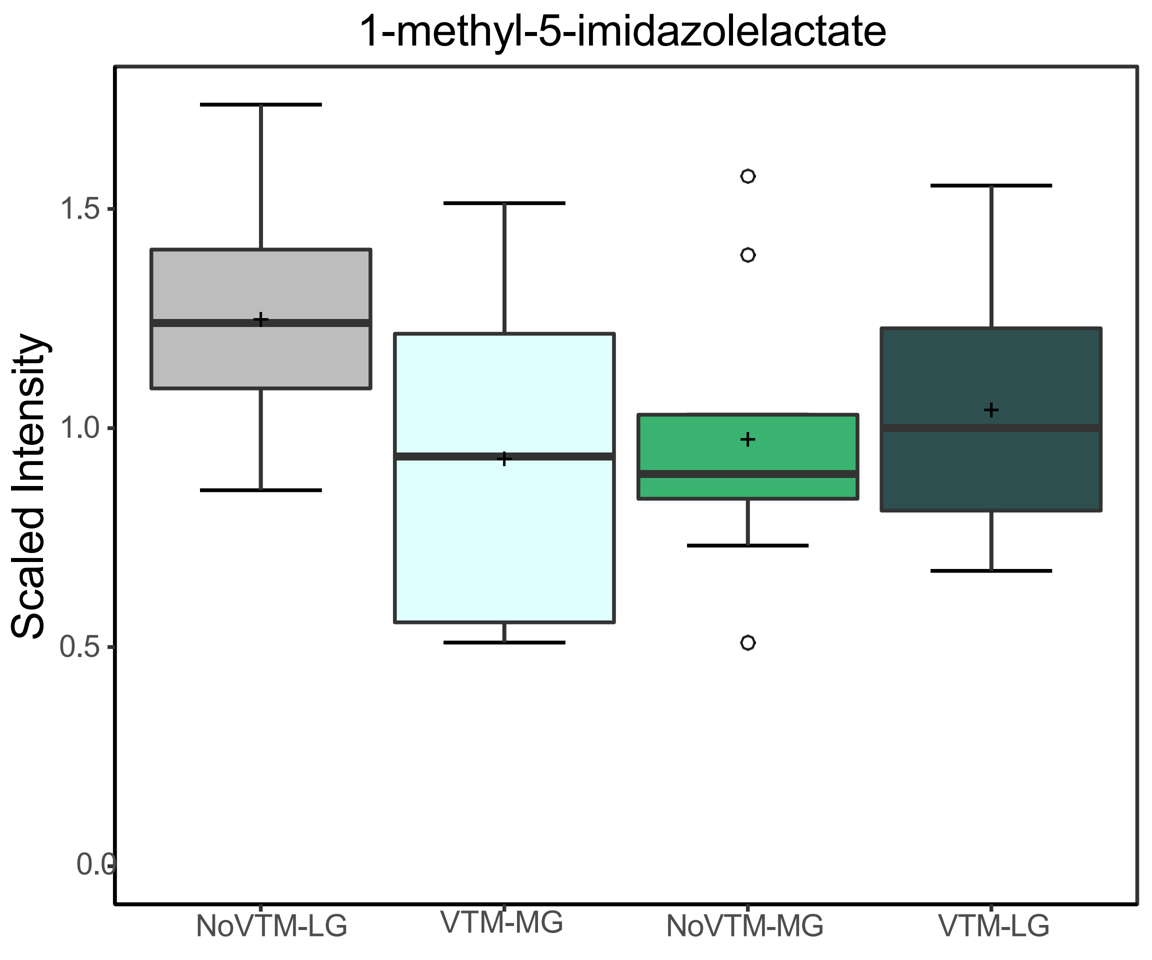

1-methyl-5-imidazolelactate
Scaled Intensity
1.5
1.0
0.5
0.0
NoVTM-LG
VTM-MG
NoVTM-MG
VTM-LG

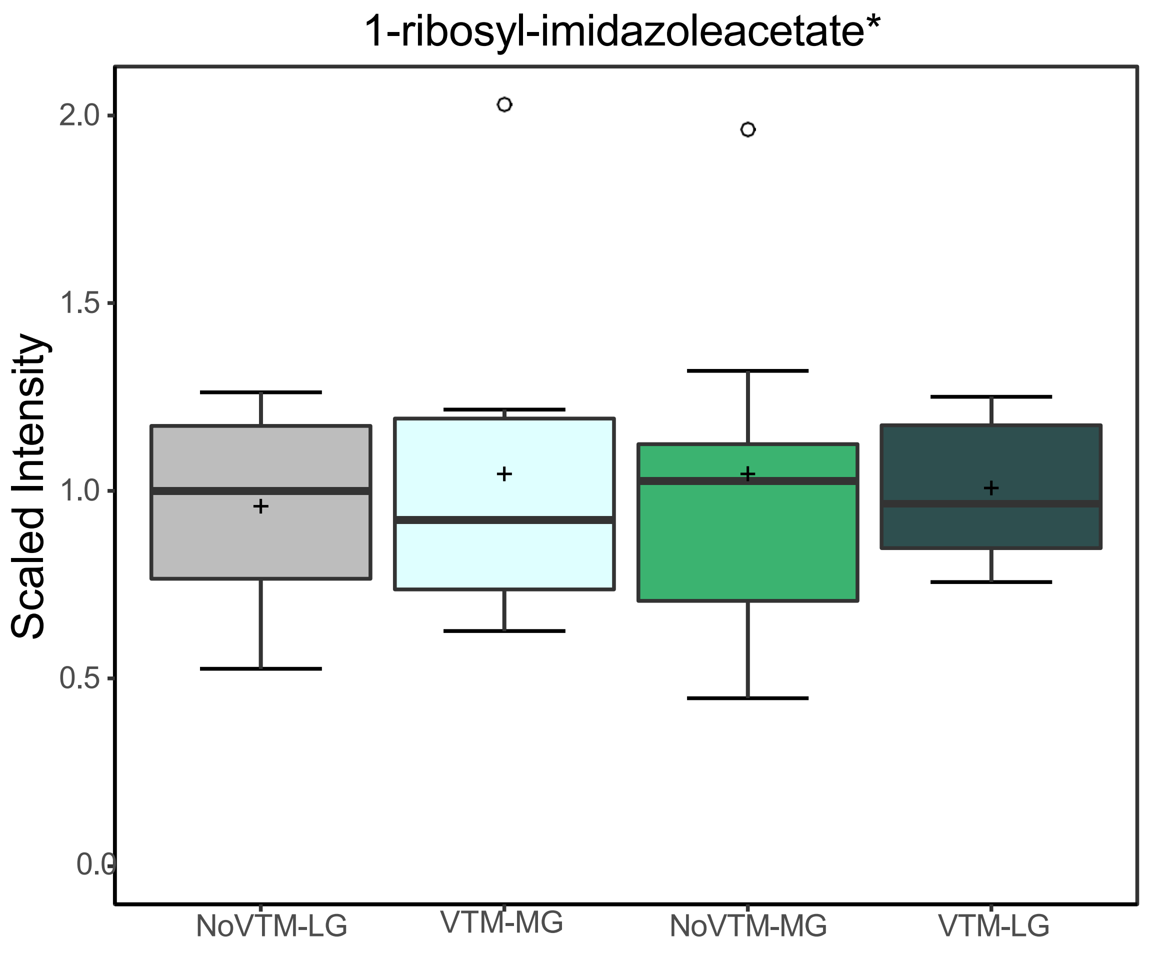
1-ribosyl-imidazoleacetate*
Scaled Intensity
2.0
1.5
1.0
0.5
0.0
NoVTM-LG
VTM-MG
NoVTM-MG
VTM-LG

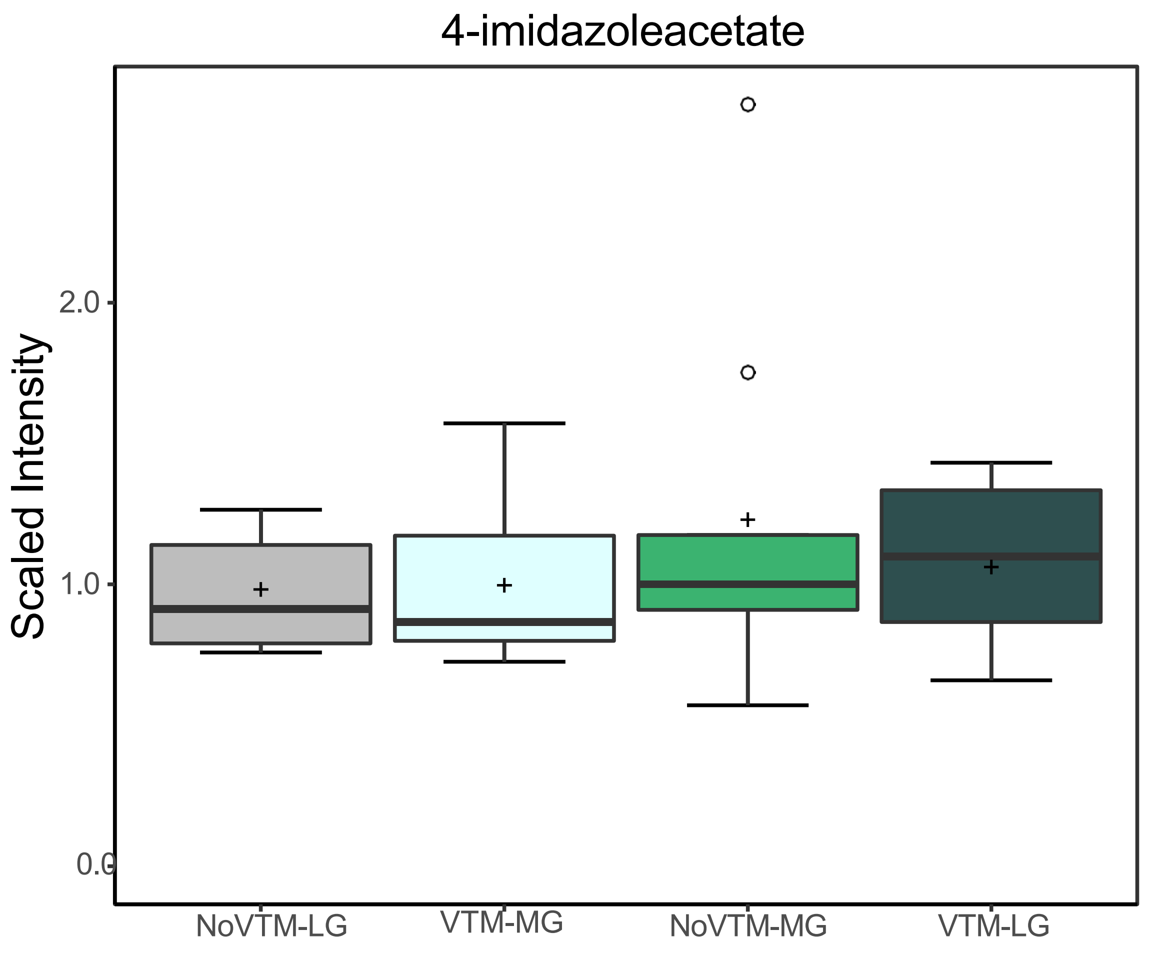
4-imidazoleacetate
Scaled Intensity
2.0
1.0
0.0
NoVTM-LG
VTM-MG
NoVTM-MG
VTM-LG

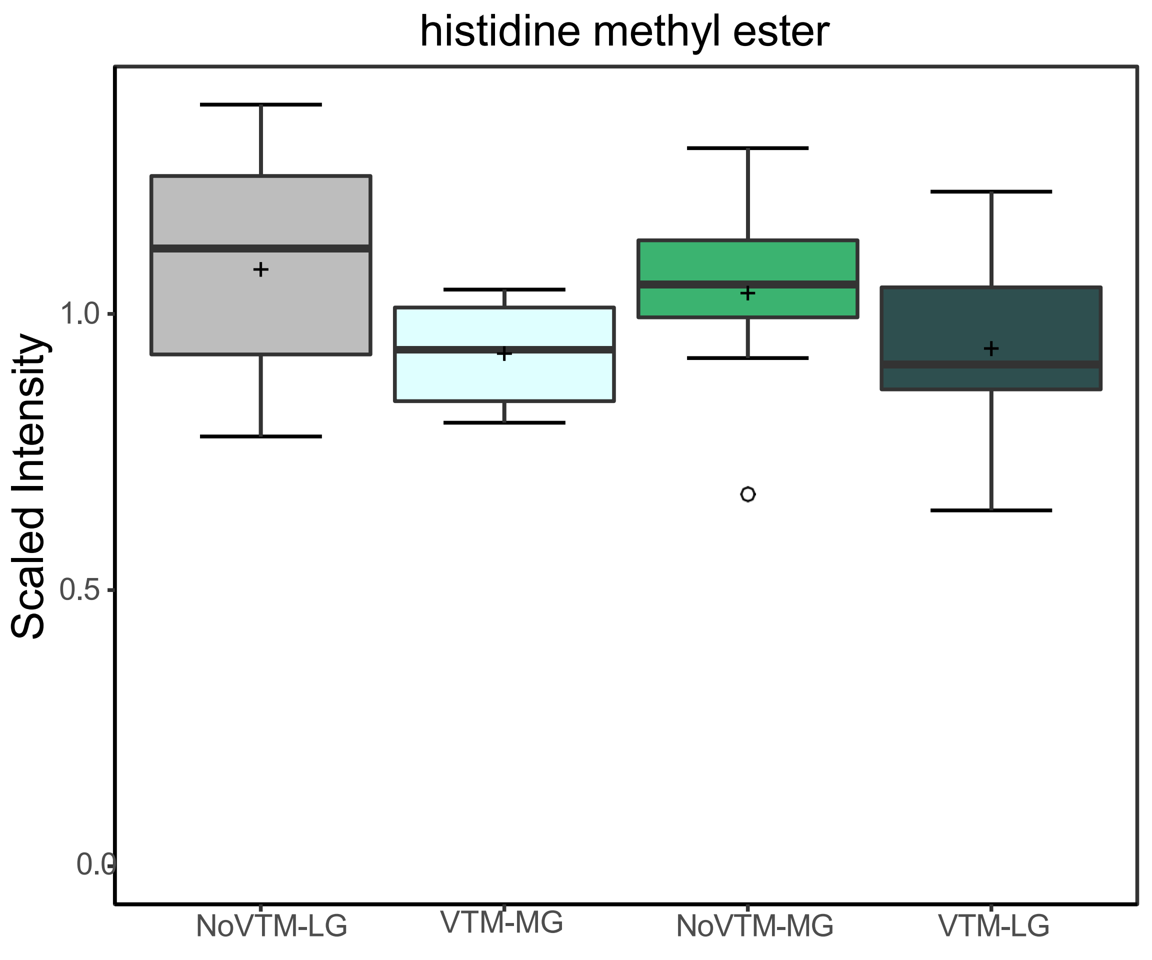
histidine methyl ester
Scaled Intensity
1.0
0.5
0.0
NoVTM-LG
VTM-MG
NoVTM-MG
VTM-LG

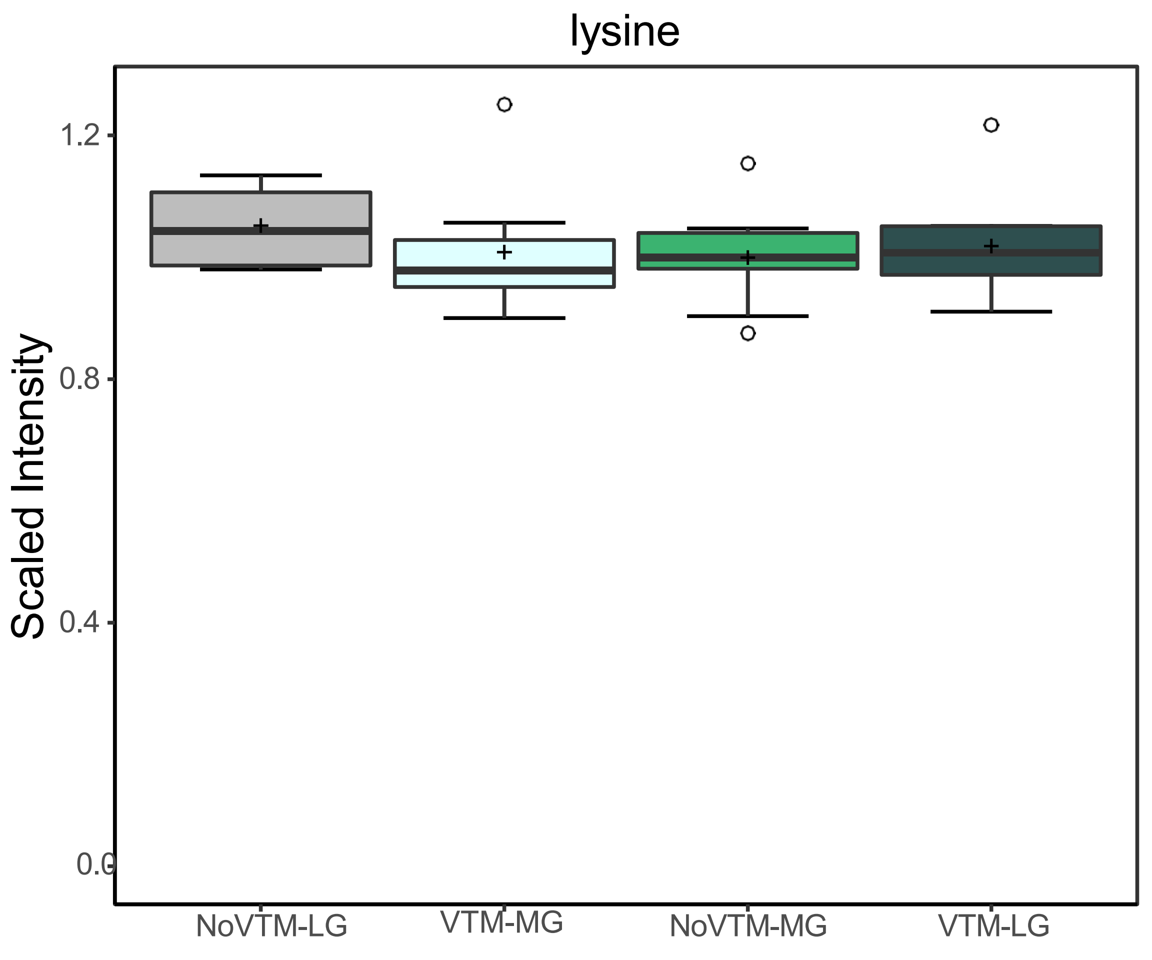
lysine
Scaled Intensity
1.2
0.8
0.4
0.0
NoVTM-LG
VTM-MG
NoVTM-MG
VTM-LG

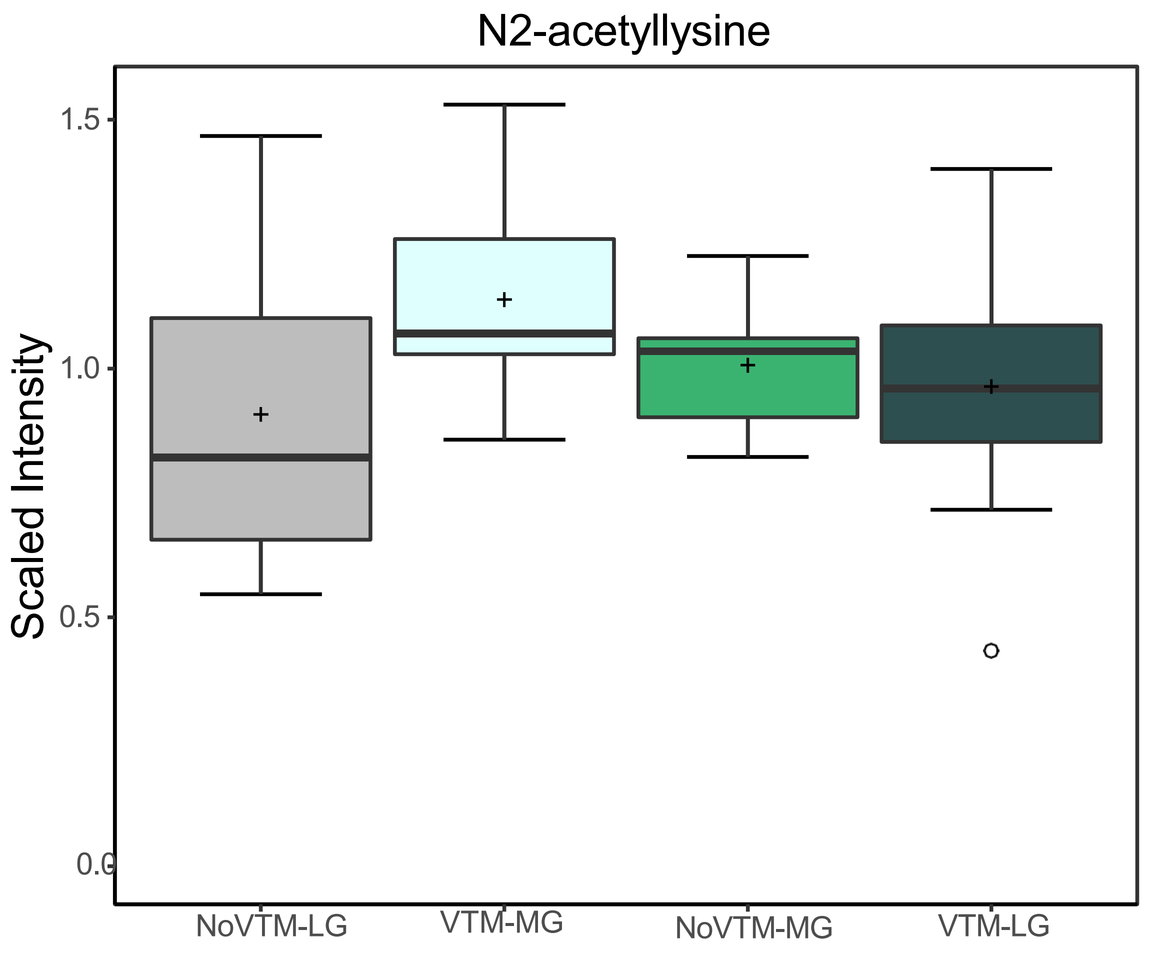
N2-acetyllysine
Scaled Intensity
1.5
1.0
0.5
0.0
NoVTM-LG
VTM-MG
NoVTM-MG
VTM-LG

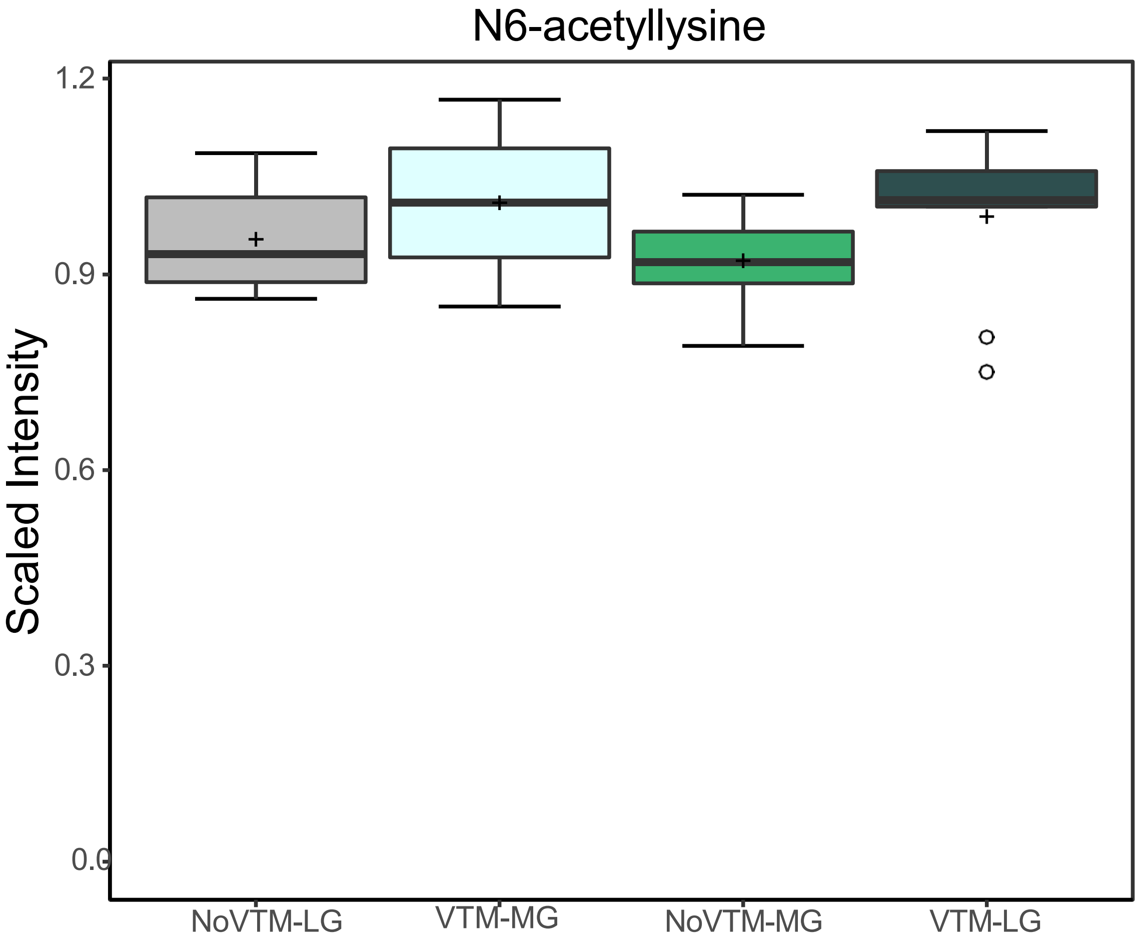

N6-acetyllysine
Scaled Intensity
1.2
0.9
0.6
0.3
0.0
NoVTM-LG
VTM-MG
NoVTM-MG
VTM-LG

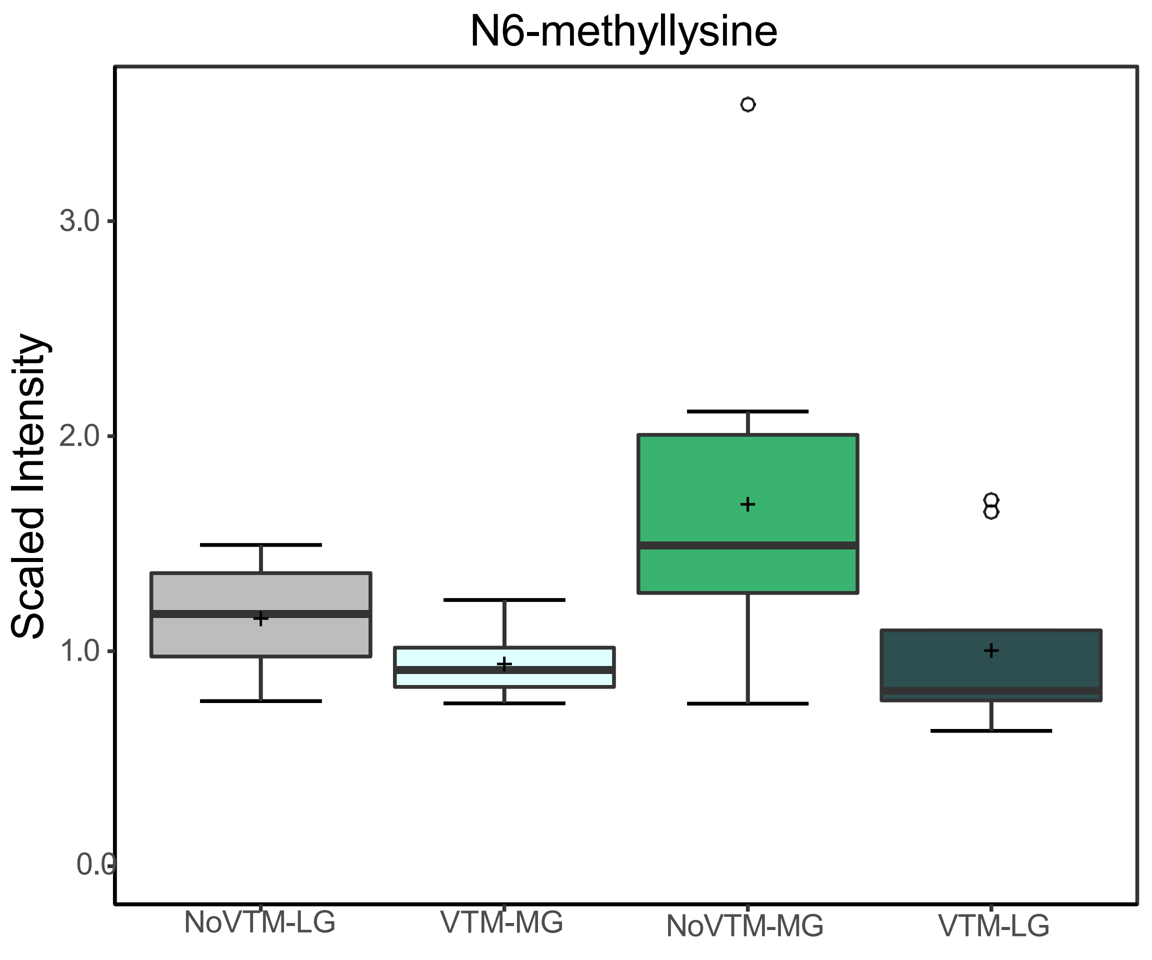
N6-methyllysine
Scaled Intensity
3.0
2.0
1.0
0.0
NoVTM-LG
VTM-MG
NoVTM-MG
VTM-LG

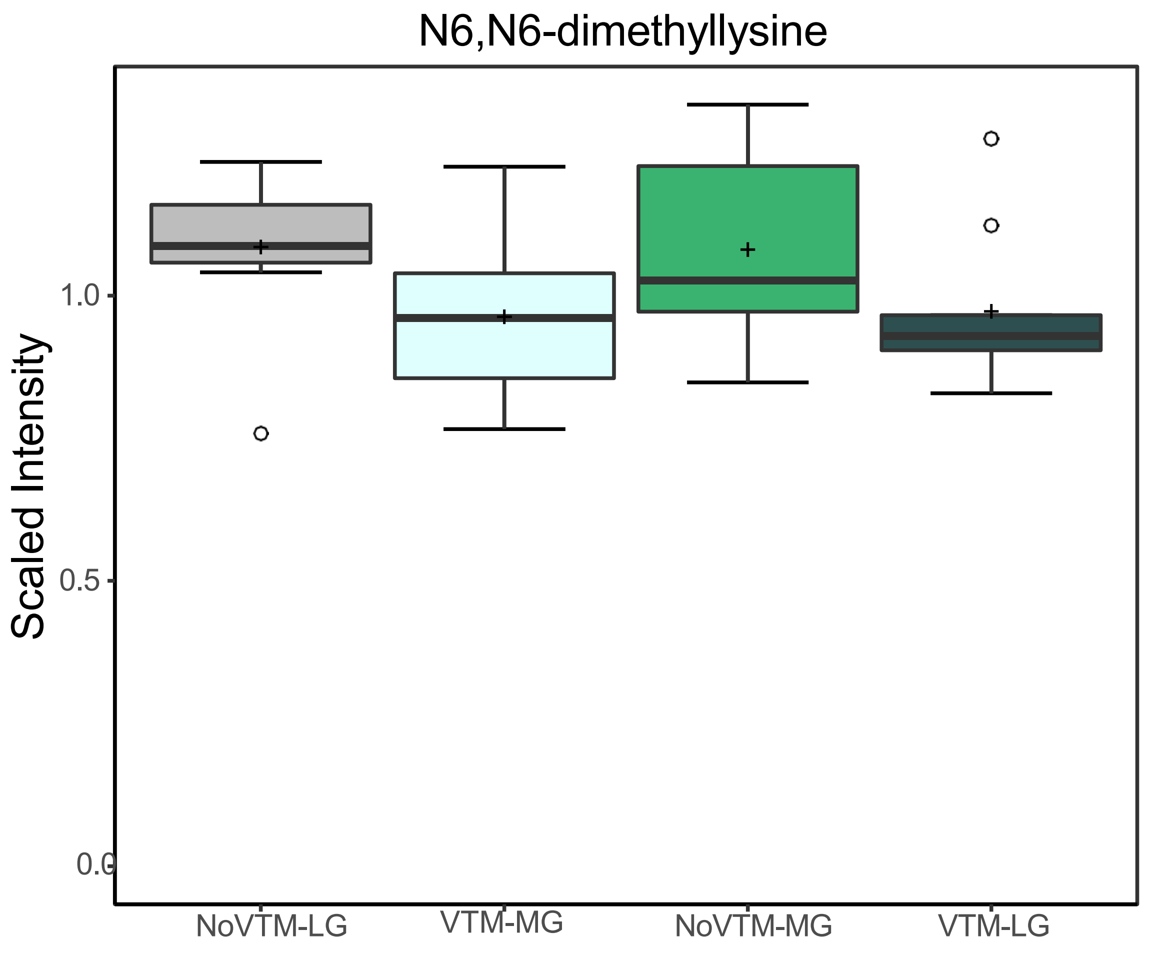
N6,N6-dimethyllysine
Scaled Intensity
1.0
0.5
0.0
NoVTM-LG
VTM-MG
NoVTM-MG
VTM-LG

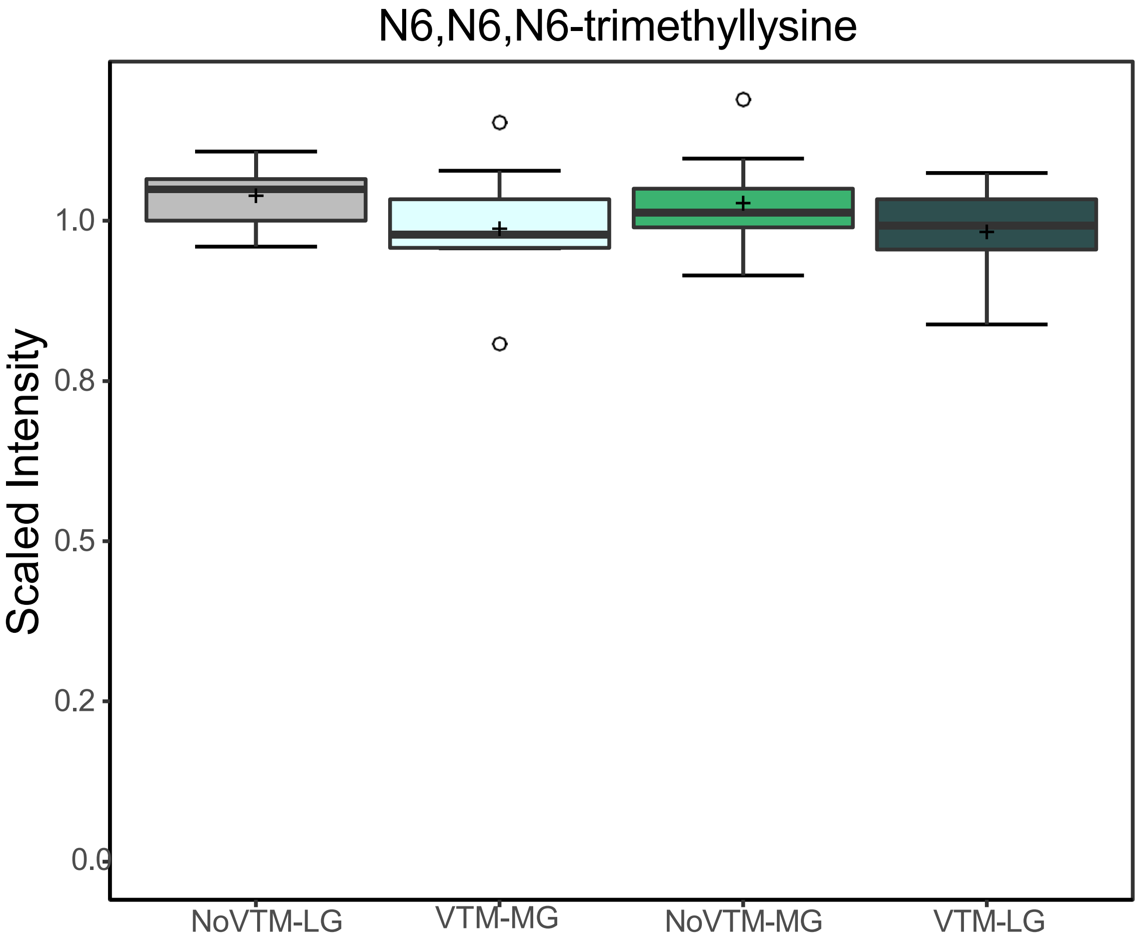
N6,N6,N6-trimethyllysine
Scaled Intensity
1.0
0.8
0.5
0.2
0.0
NoVTM-LG
VTM-MG
NoVTM-MG
VTM-LG

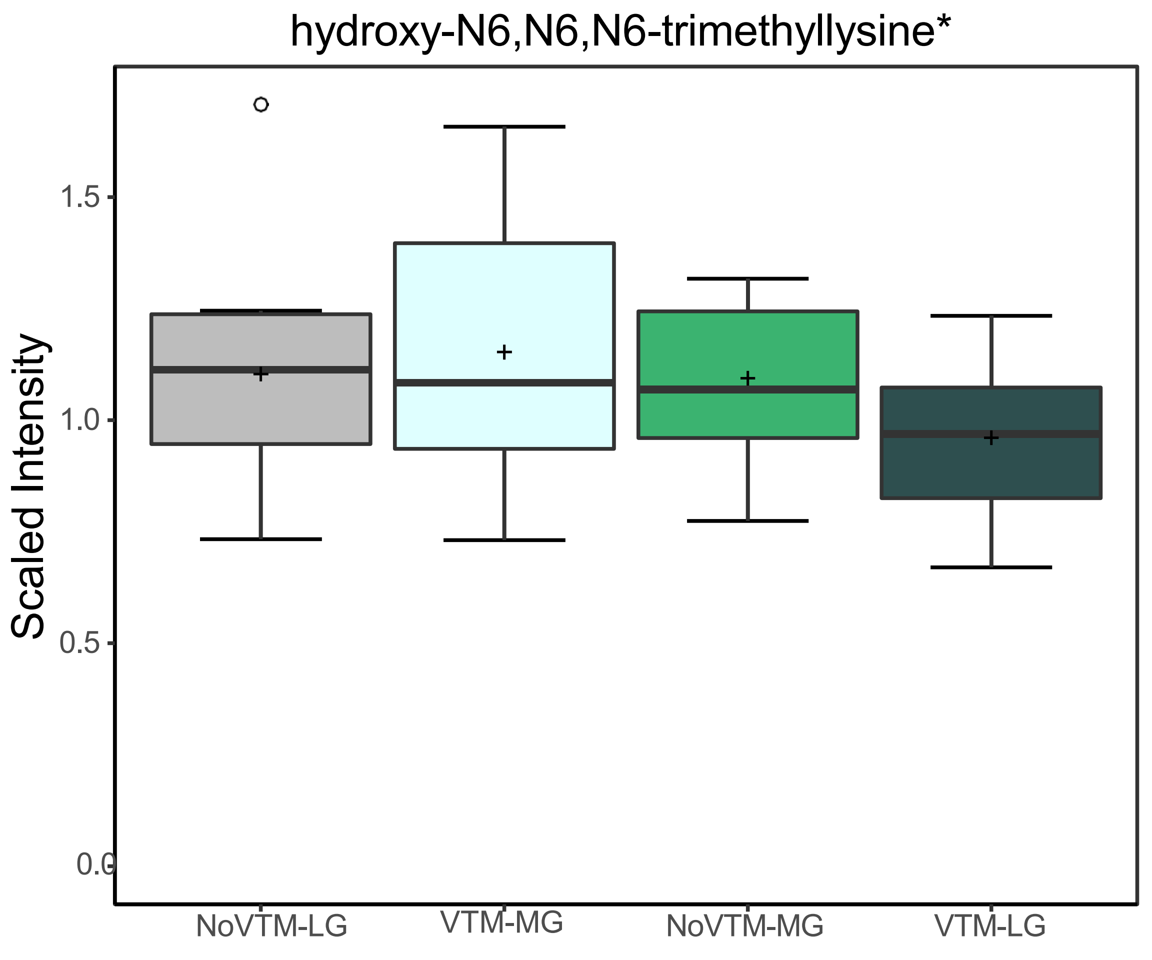
hydroxy-N6,N6,N6-trimethyllysine*
Scaled Intensity
1.5
1.0
0.5
0.0
NoVTM-LG
VTM-MG
NoVTM-MG
VTM-LG

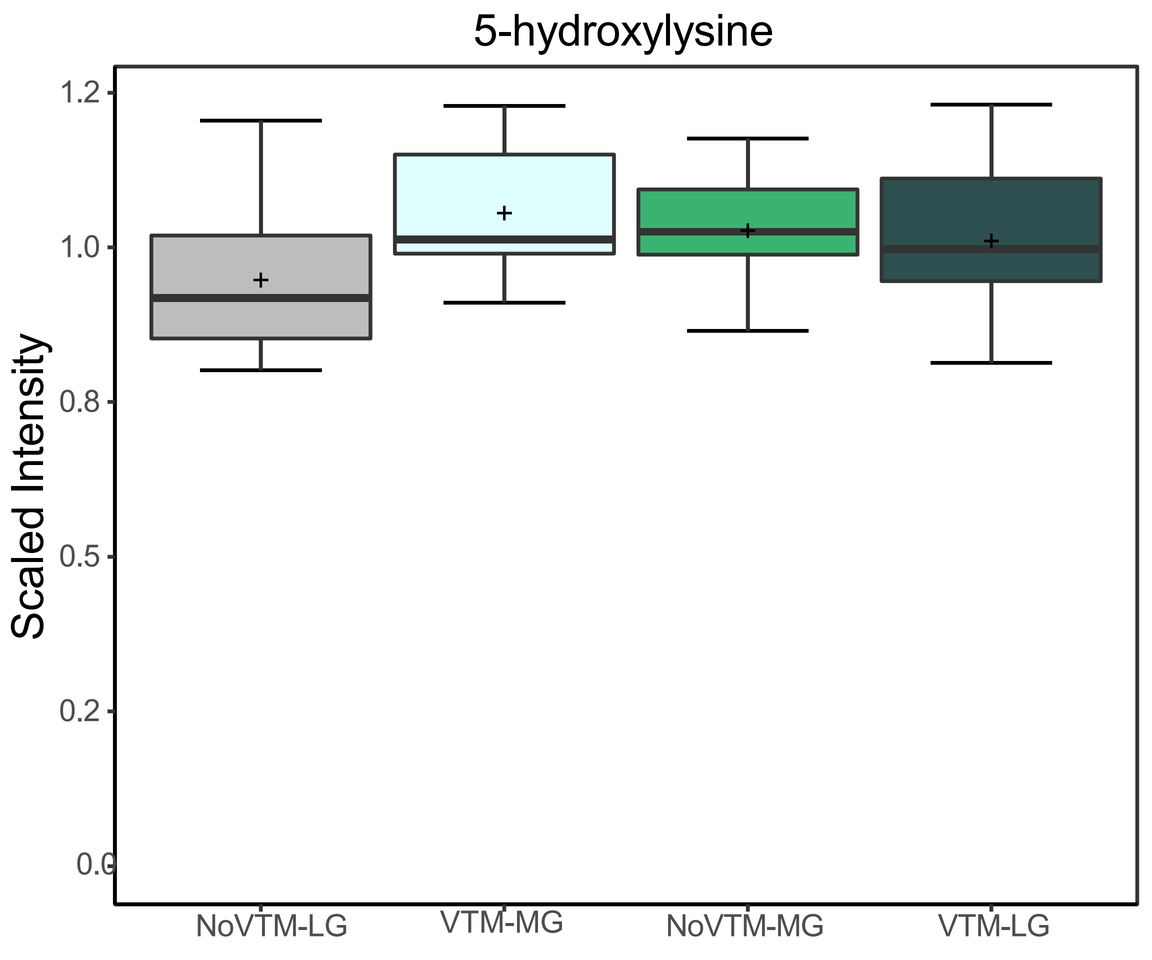
5-hydroxylysine
Scaled Intensity
1.2
1.0
0.8
0.5
0.2
0.0
NoVTM-LG
VTM-MG
NoVTM-MG
VTM-LG

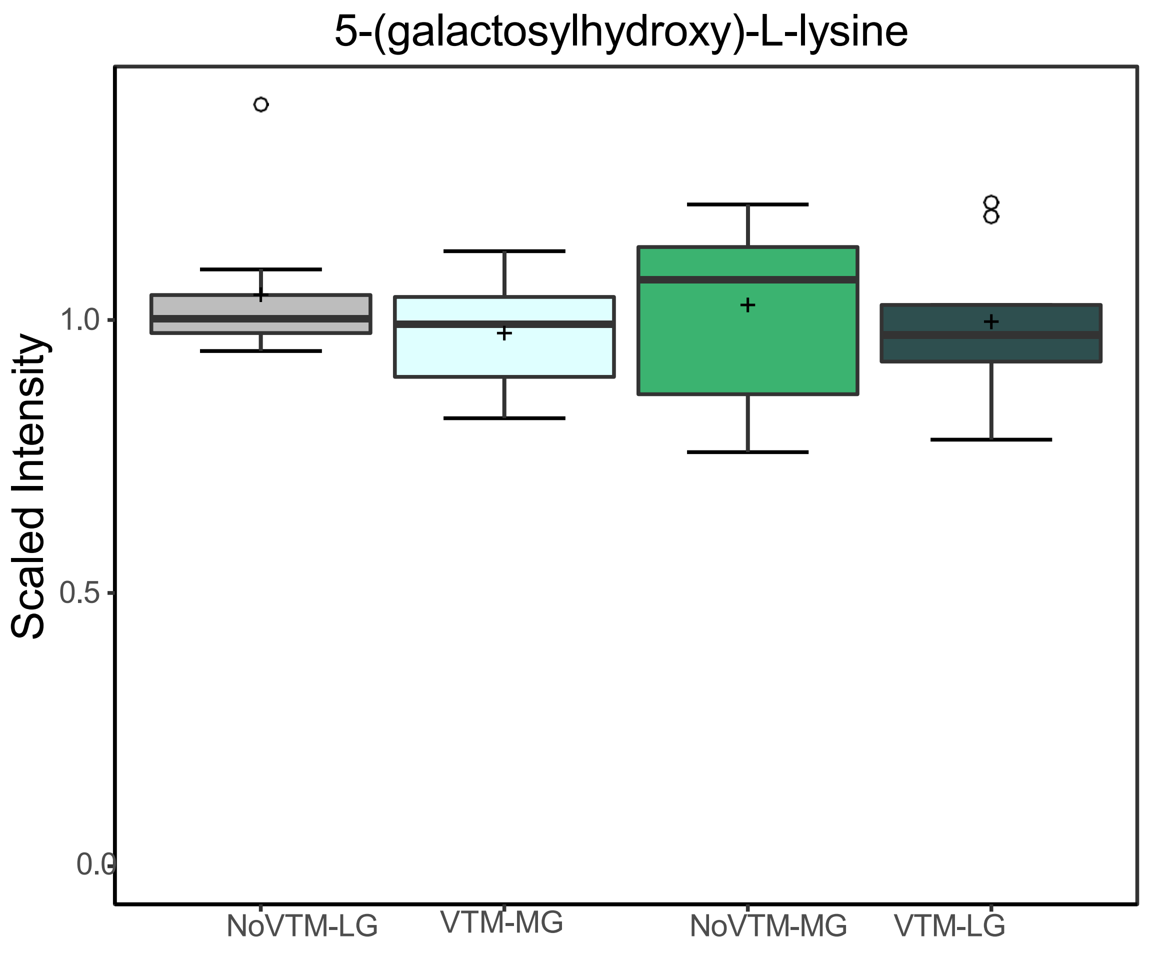
5-(galactosylhydroxy)-L-lysine
Scaled Intensity
1.0
0.5
0.0
NoVTM-LG
VTM-MG
NoVTM-MG
VTM-LG

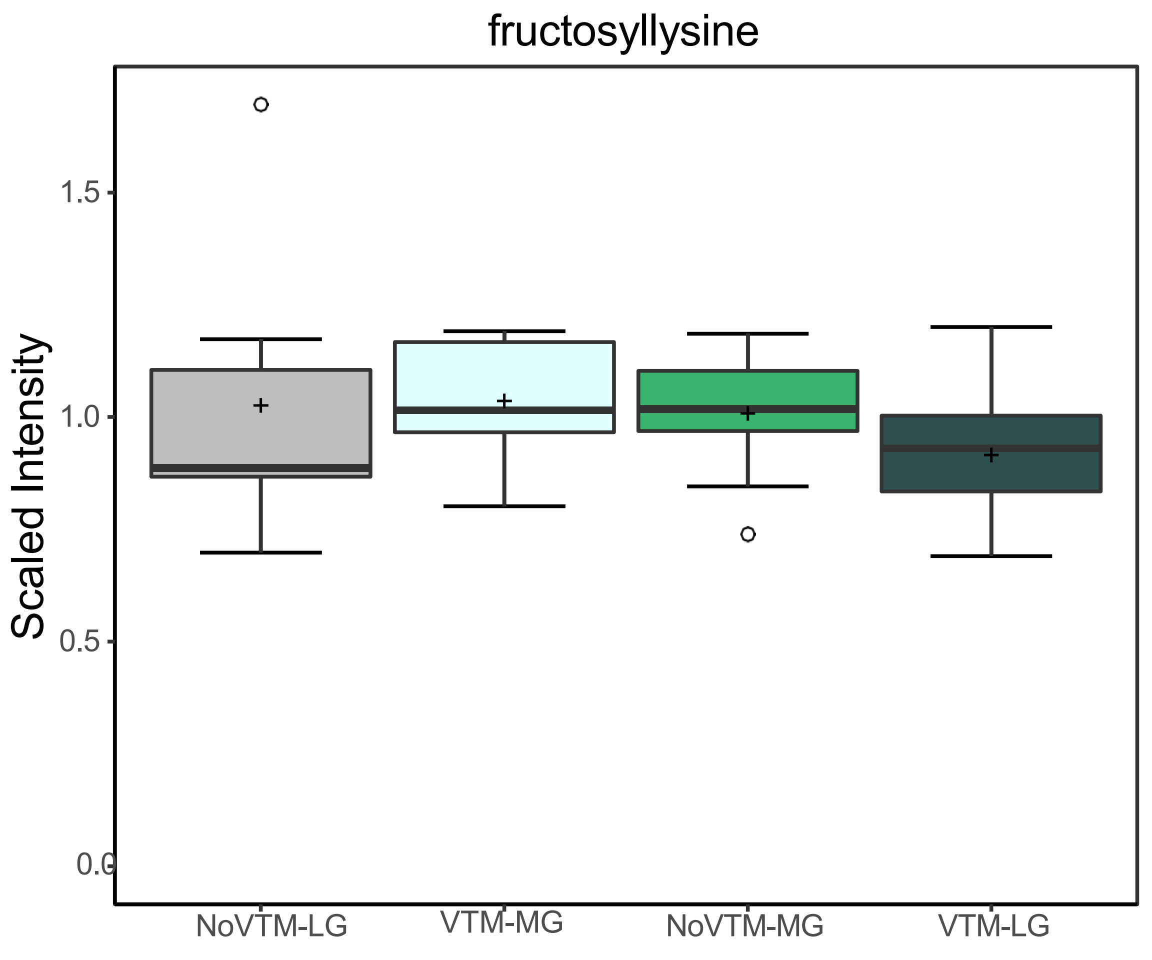
fructosyllysine
Scaled Intensity
1.5
1.0
0.5
0.0
NoVTM-LG
VTM-MG
NoVTM-MG
VTM-LG

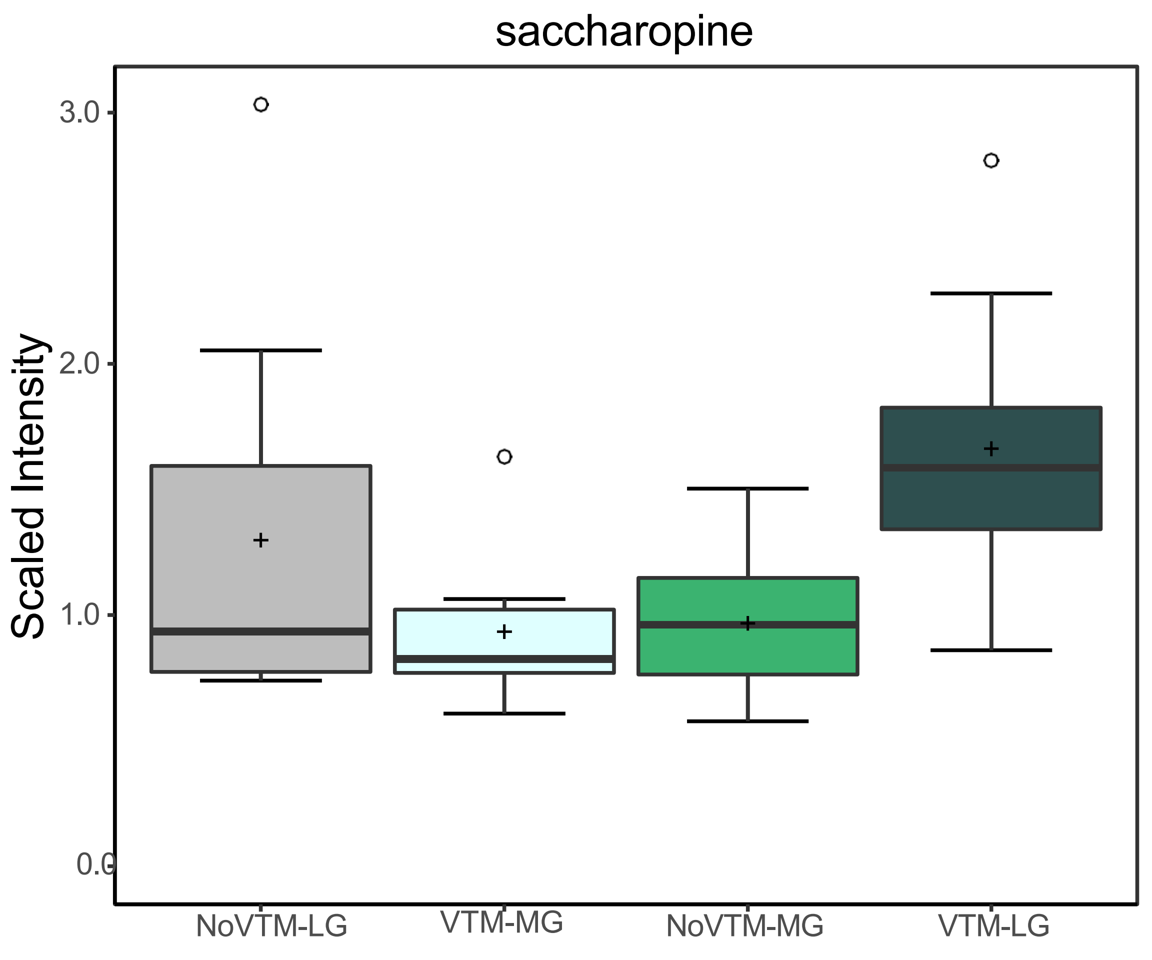
saccharopine
Scaled Intensity
3.0
2.0
1.0
0.0
NoVTM-LG
VTM-MG
NoVTM-MG
VTM-LG

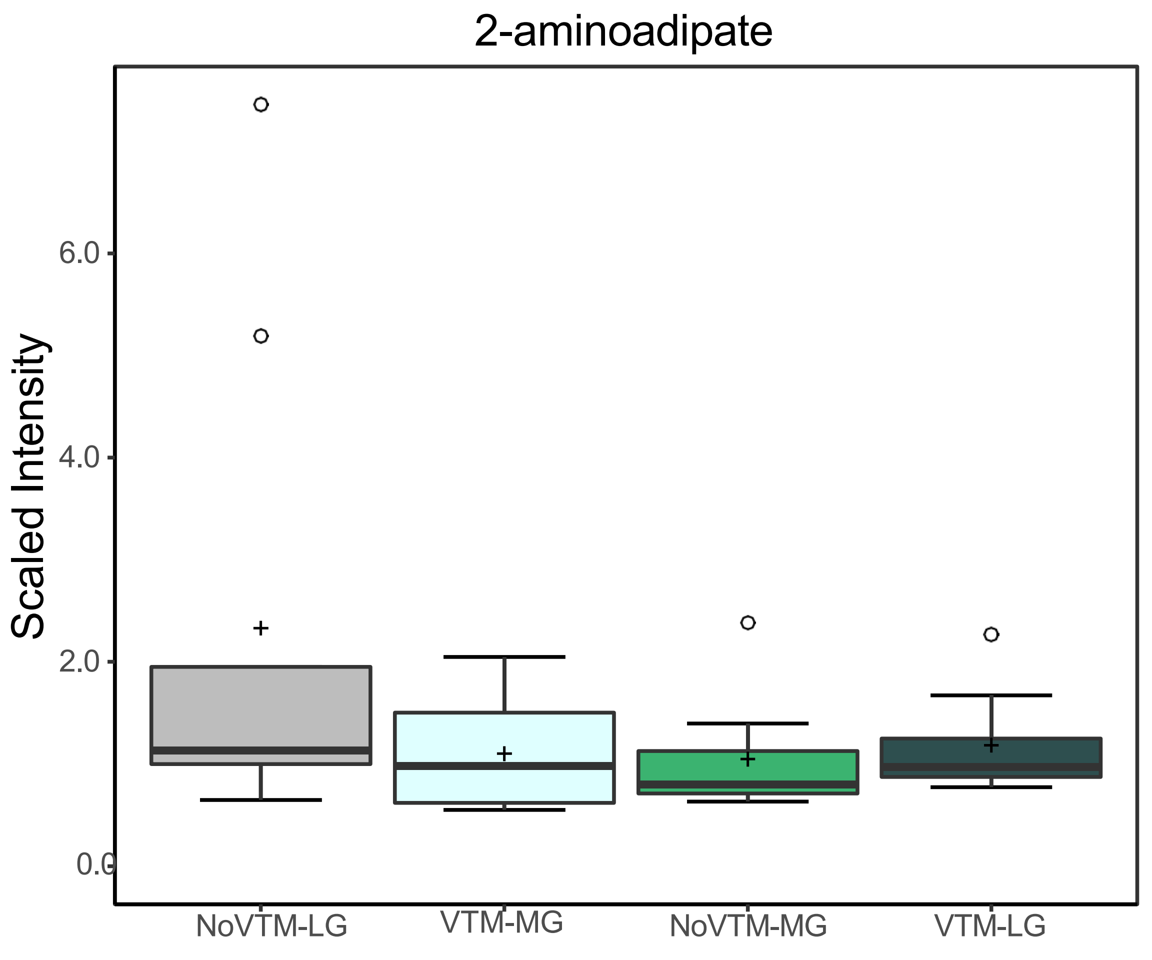
2-aminoadipate
Scaled Intensity
6.0
4.0
2.0
0.0
NoVTM-LG
VTM-MG
NoVTM-MG
VTM-LG

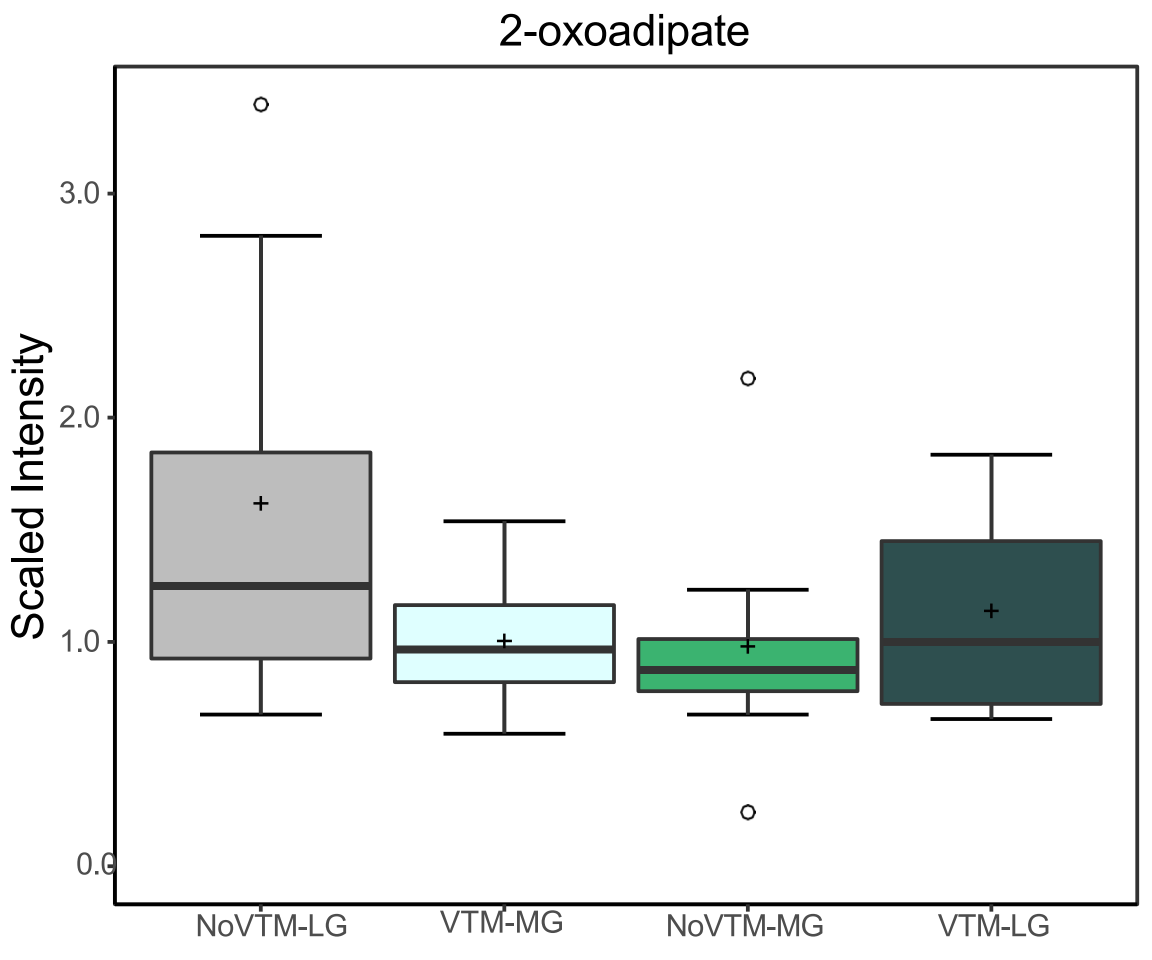
2-oxoadipate
Scaled Intensity
3.0
2.0
1.0
0.0
NoVTM-LG
VTM-MG
NoVTM-MG
VTM-LG

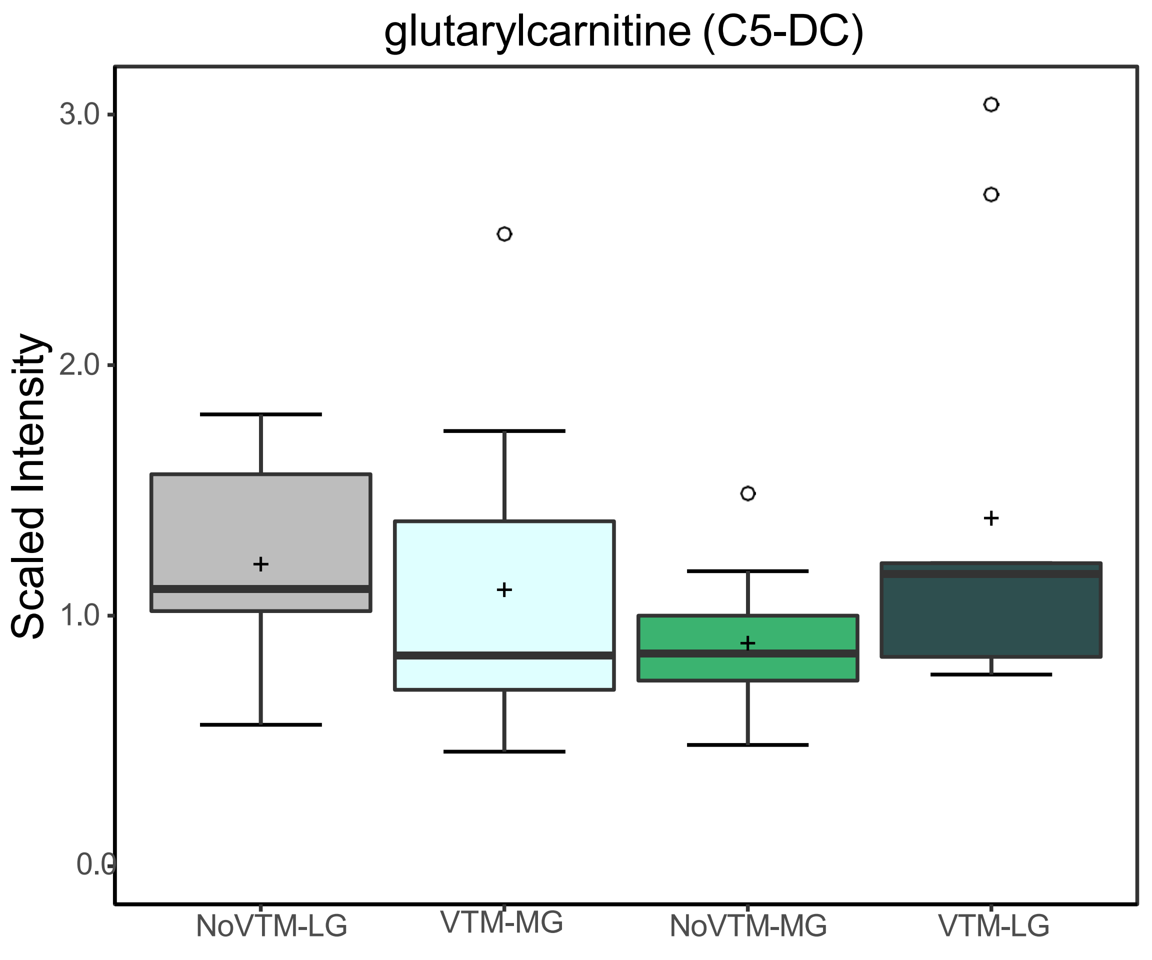

glutarylcarnitine (C5-DC)
Scaled Intensity
3.0
2.0
1.0
0.0
NoVTM-LG
VTM-MG
NoVTM-MG
VTM-LG

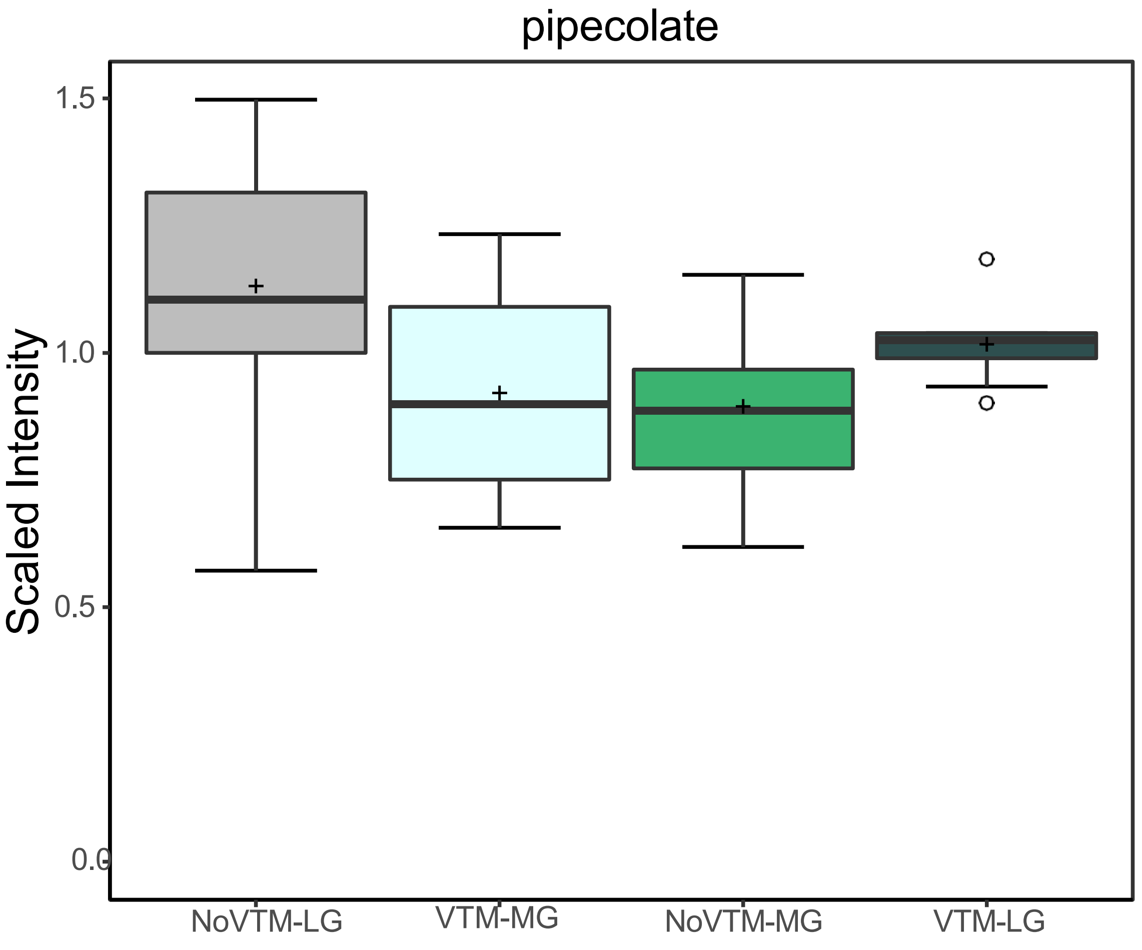
pipecolate
Scaled Intensity
1.5
1.0
0.5
0.0
NoVTM-LG
VTM-MG
NoVTM-MG
VTM-LG

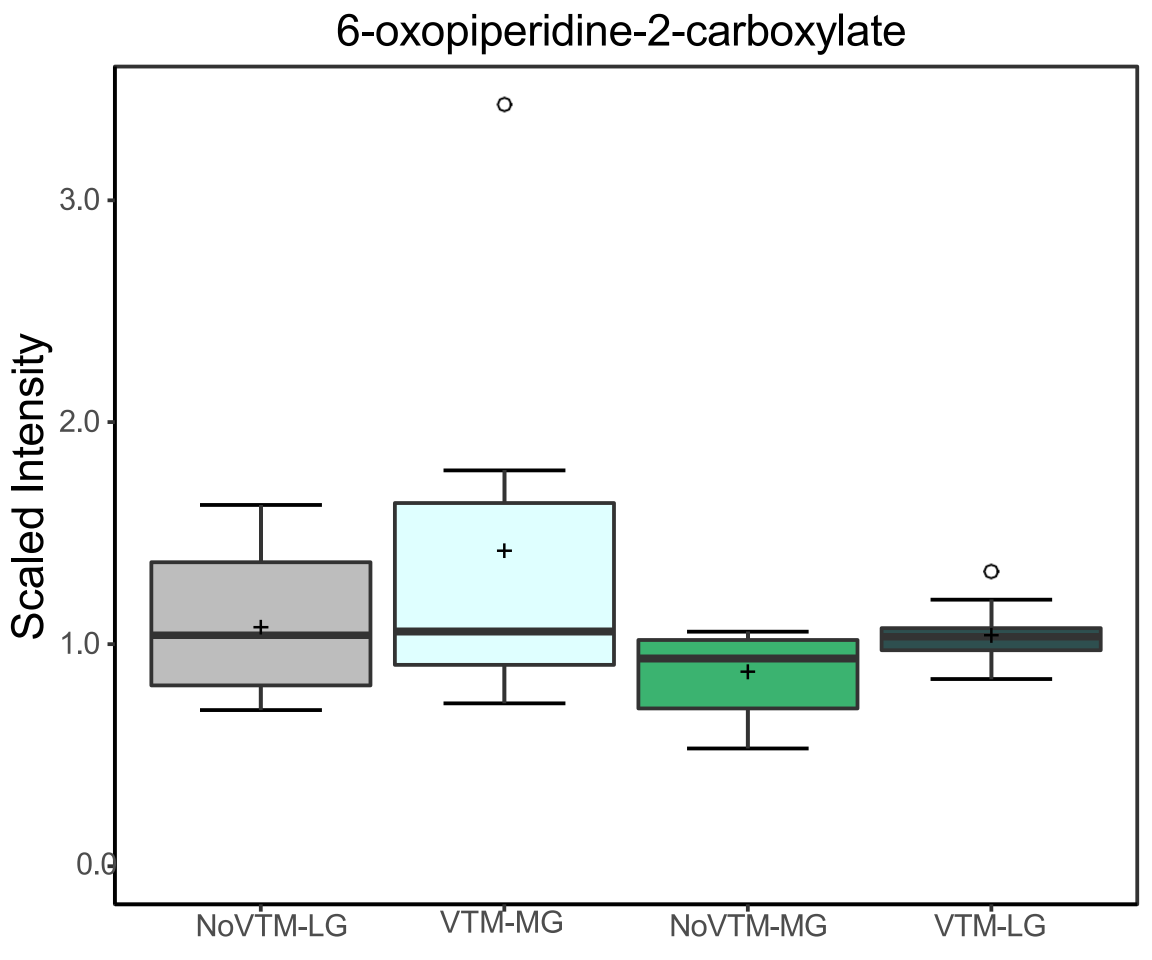
6-oxopiperidine-2-carboxylate
Scaled Intensity
3.0
2.0
1.0
0.0
NoVTM-LG
VTM-MG
NoVTM-MG
VTM-LG

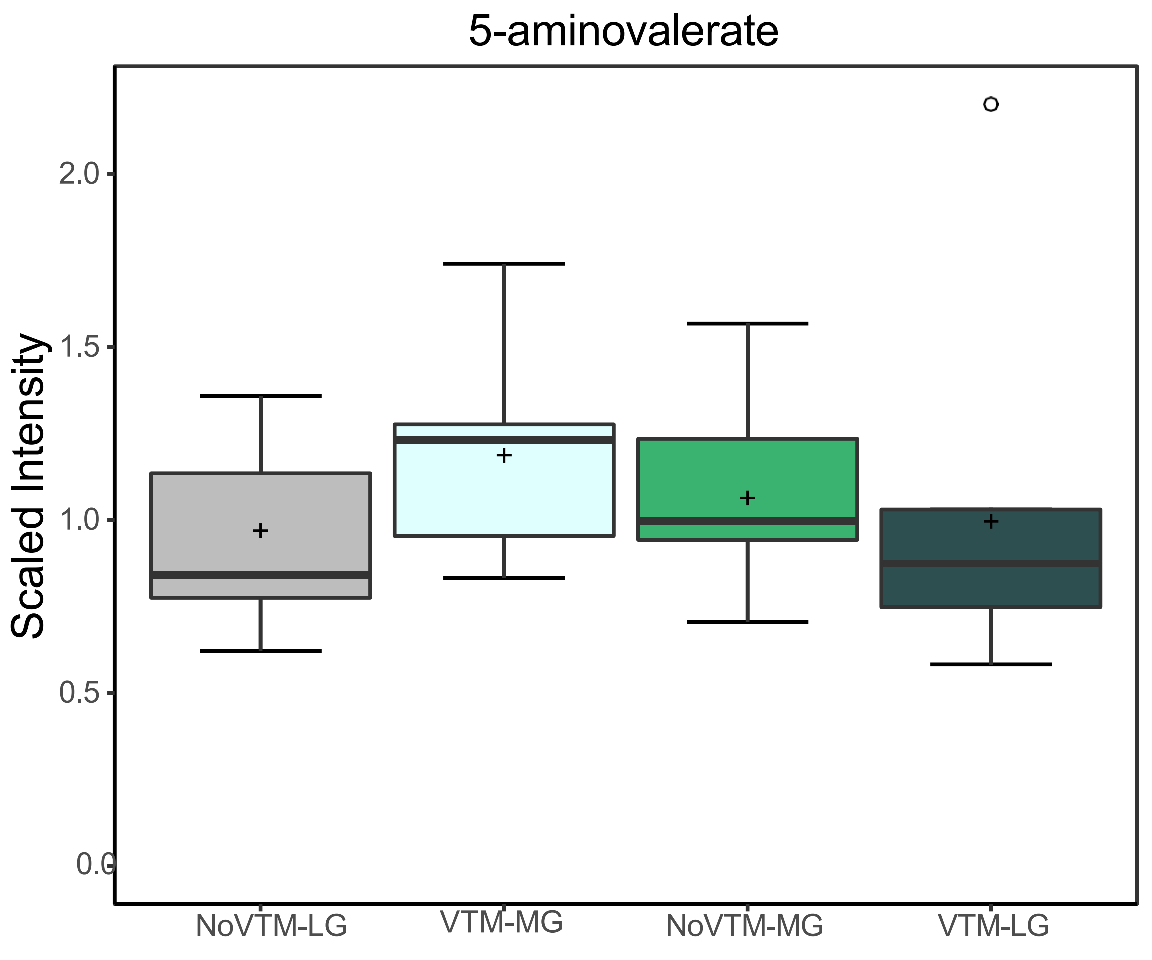
5-aminovalerate
Scaled Intensity
2.0
1.5
1.0
0.5
0.0
NoVTM-LG
VTM-MG
NoVTM-MG
VTM-LG

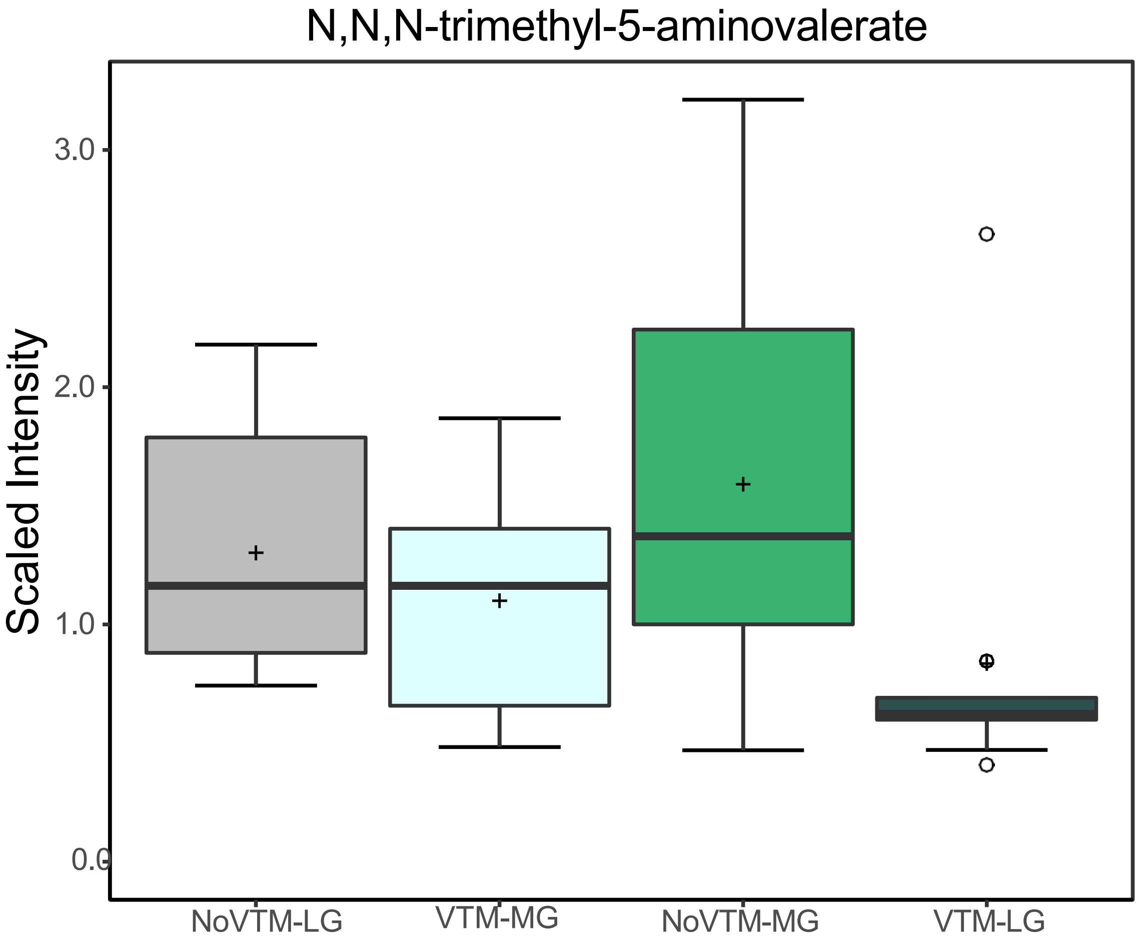

N,N,N-trimethyl-5-aminovalerate
Scaled Intensity
3.0
2.0
1.0
0.0
NoVTM-LG
VTM-MG
NoVTM-MG
VTM-LG

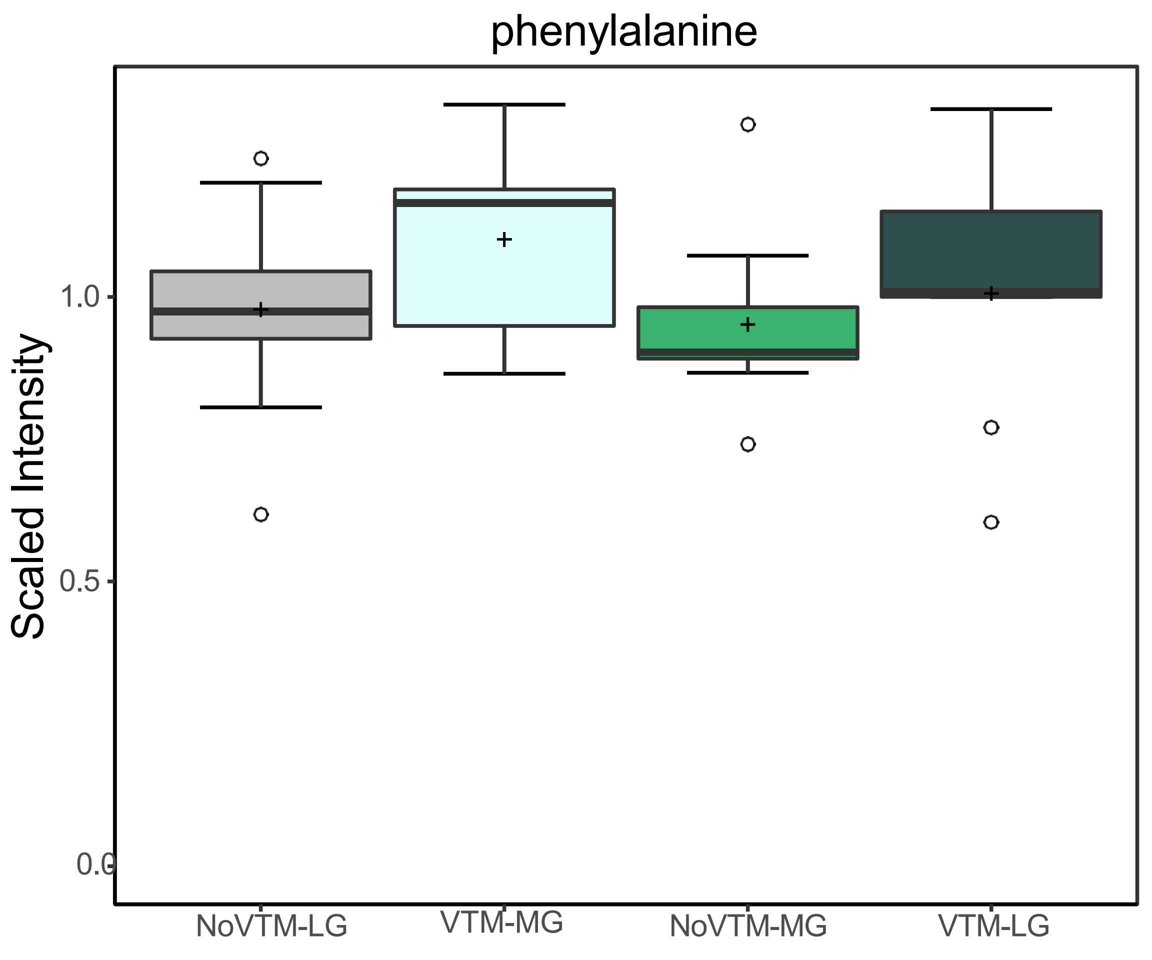
phenylalanine
Scaled Intensity
1.0
0.5
0.0
NoVTM-LG
VTM-MG
NoVTM-MG
VTM-LG

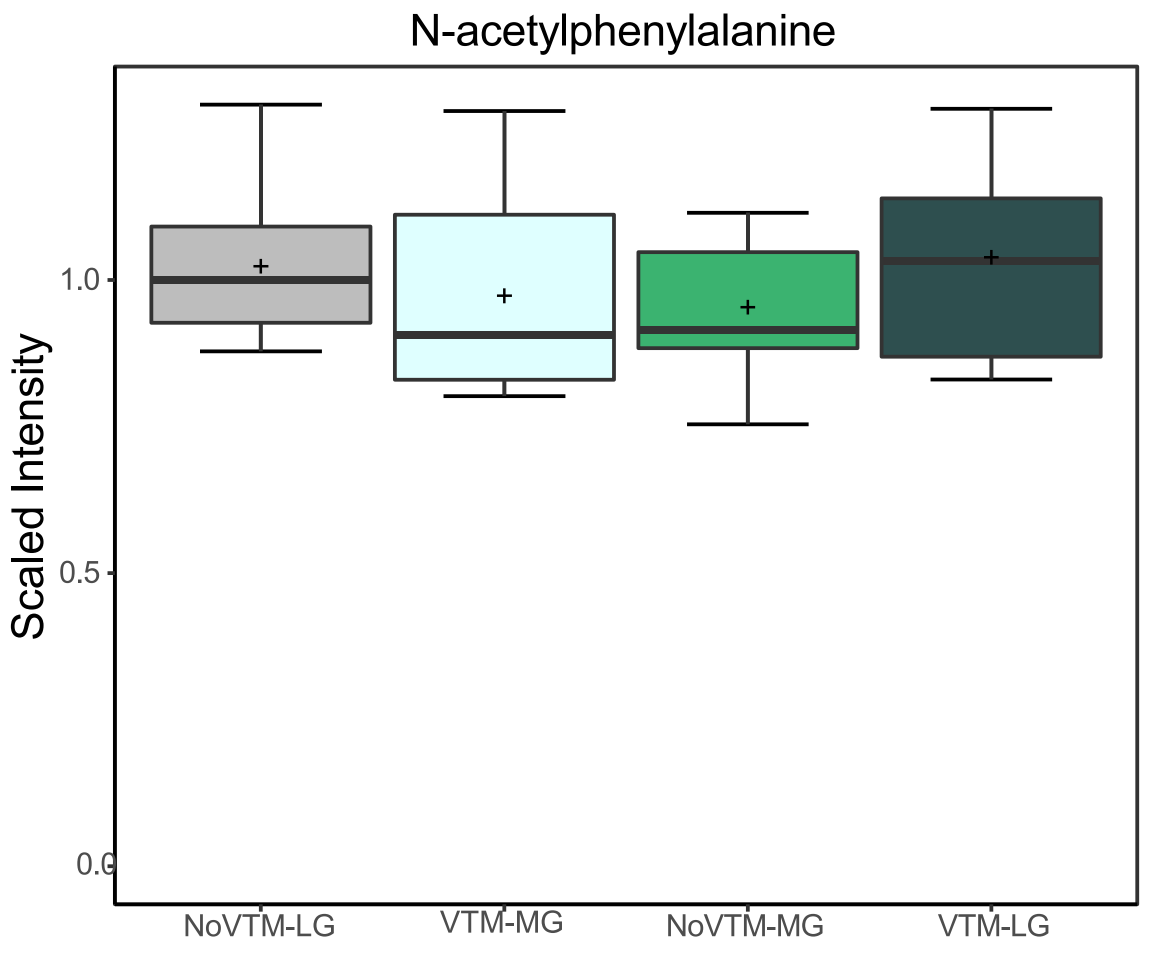
N-acetylphenylalanine
Scaled Intensity
1.0
0.5
0.0
NoVTM-LG
VTM-MG
NoVTM-MG
VTM-LG

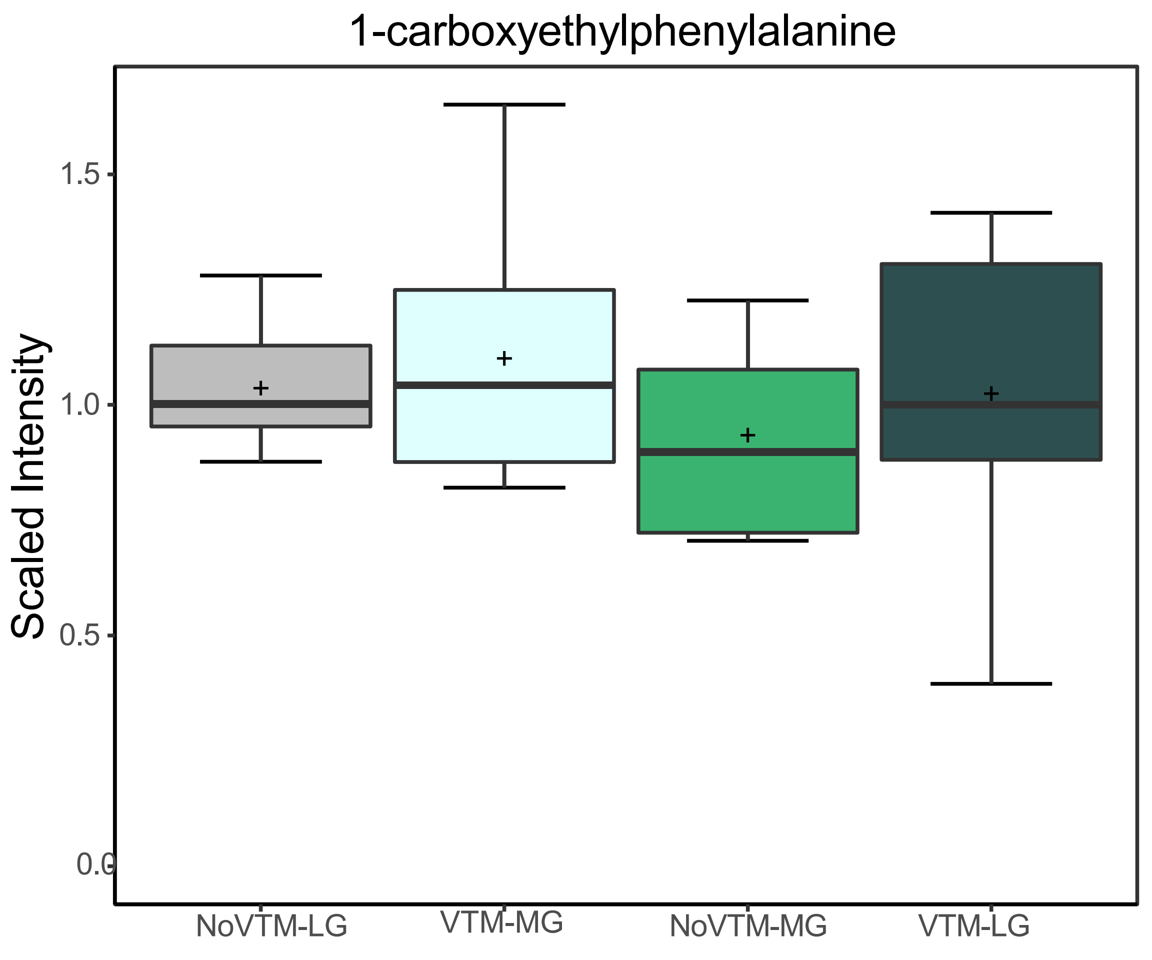

1-carboxyethylphenylalanine
Scaled Intensity
1.5
1.0
0.5
0.0
NoVTM-LG
VTM-MG
NoVTM-MG
VTM-LG

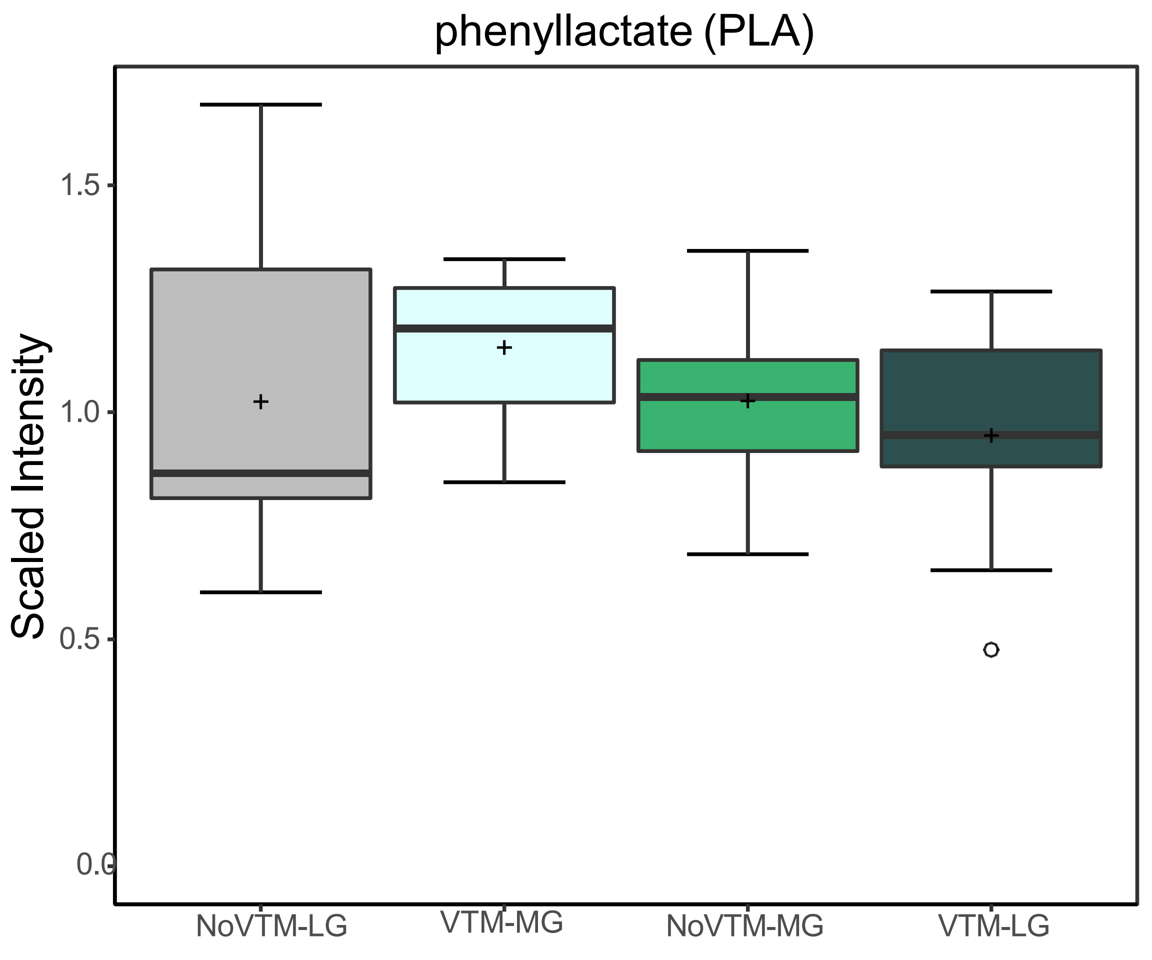
phenyllactate (PLA)
Scaled Intensity
1.5
1.0
0.5
0.0
NoVTM-LG
VTM-MG
NoVTM-MG
VTM-LG

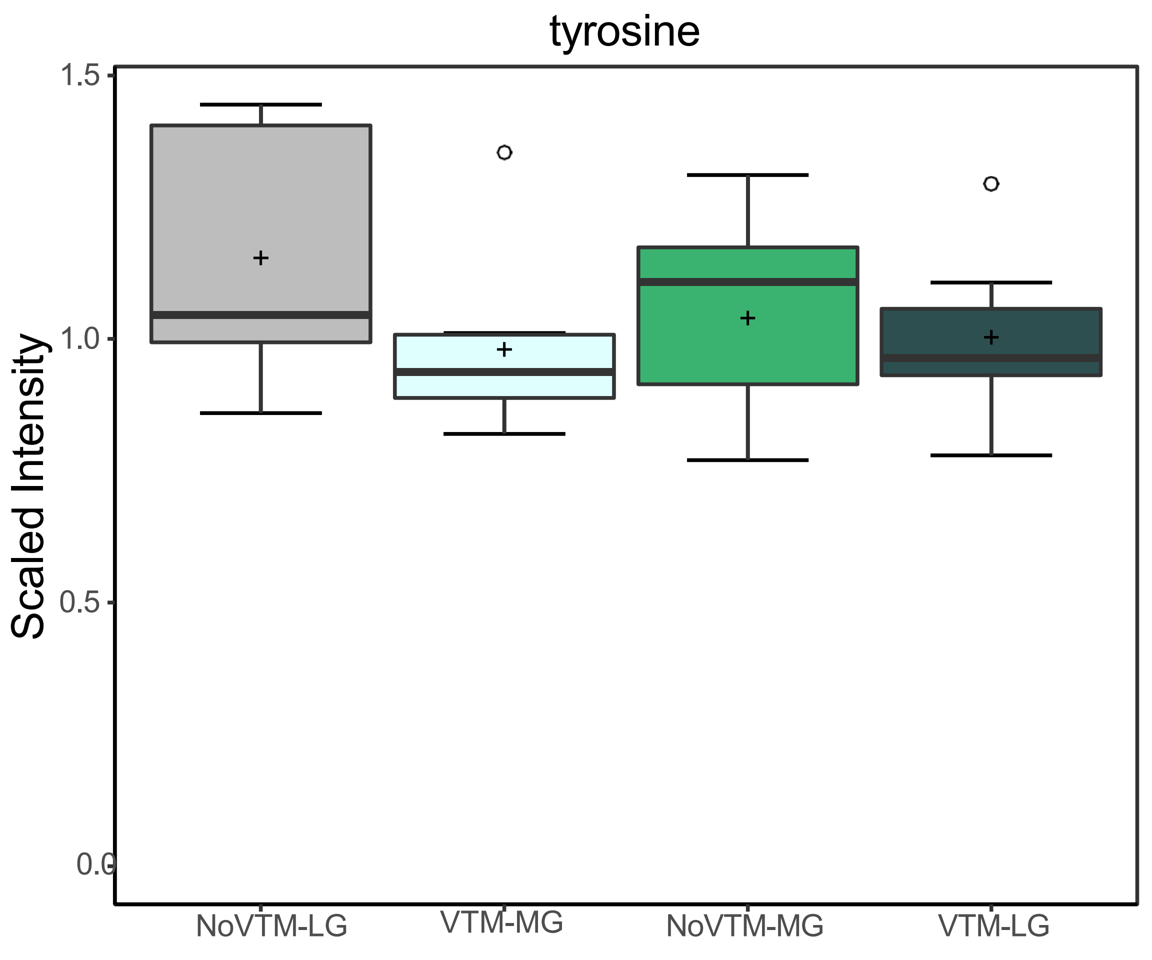

tyrosine
Scaled Intensity
1.5
1.0
0.5
0.0
NoVTM-LG
VTM-MG
NoVTM-MG
VTM-LG

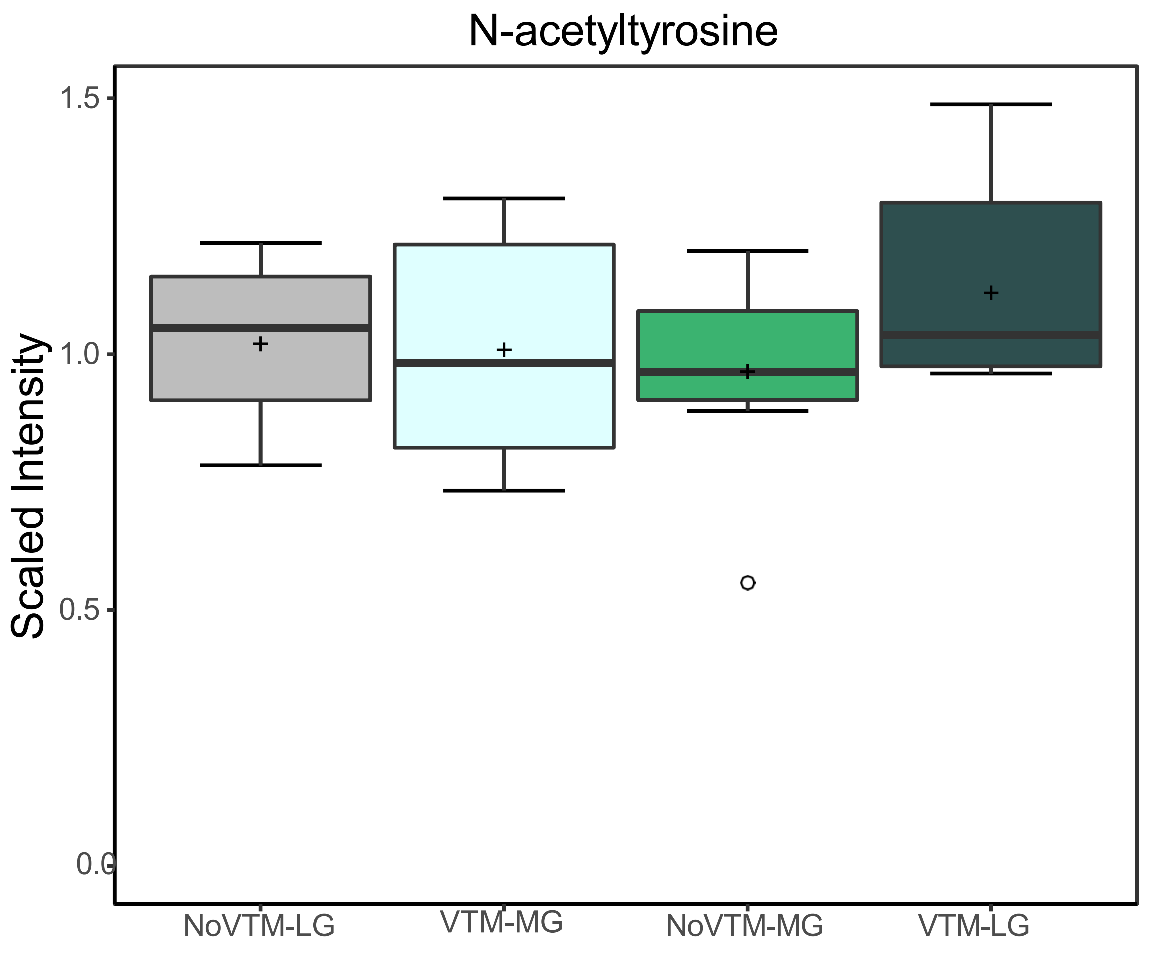

N-acetyltyrosine
Scaled Intensity
1.5
1.0
0.5
0.0
NoVTM-LG
VTM-MG
NoVTM-MG
VTM-LG

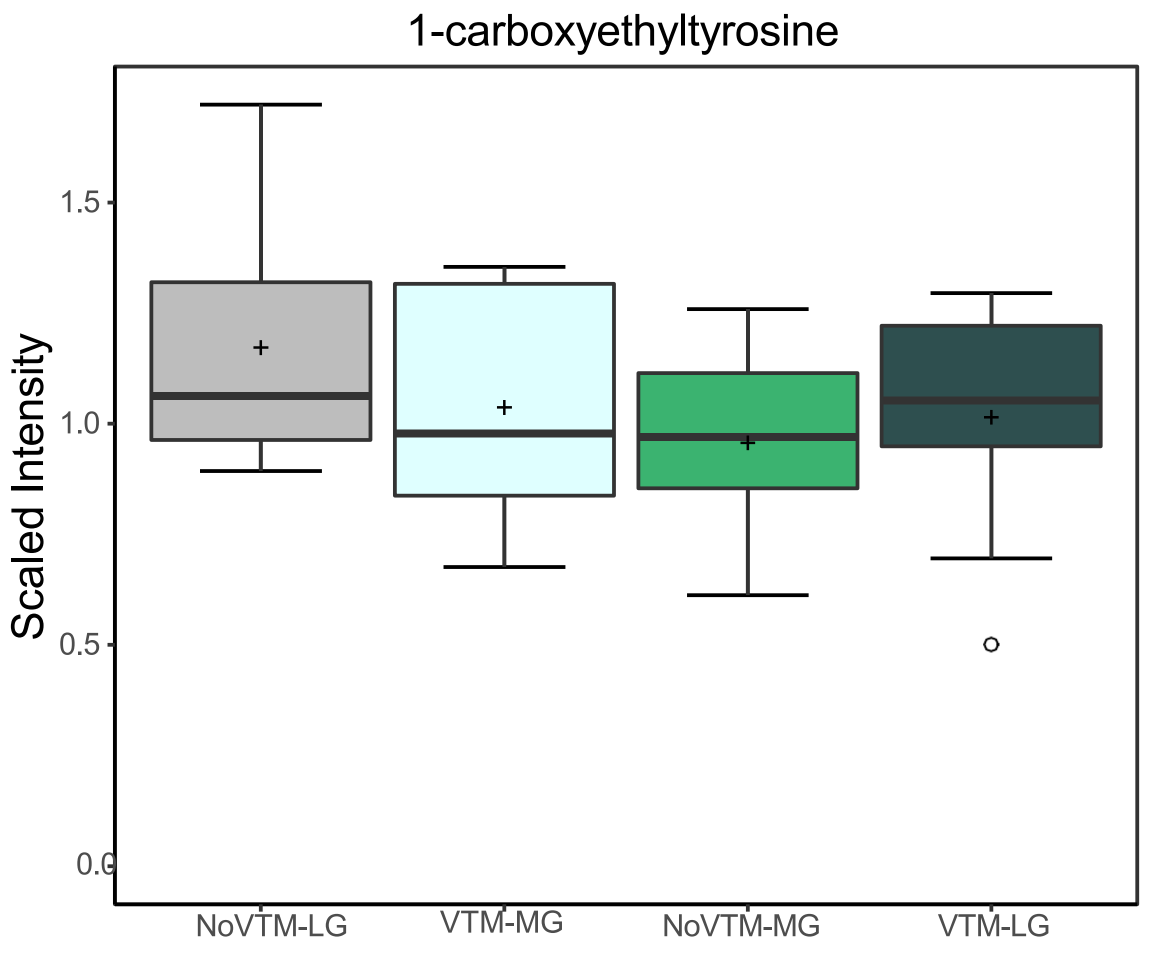
1-carboxyethyltyrosine
Scaled Intensity
1.5
1.0
0.5
0.0
NoVTM-LG
VTM-MG
NoVTM-MG
VTM-LG

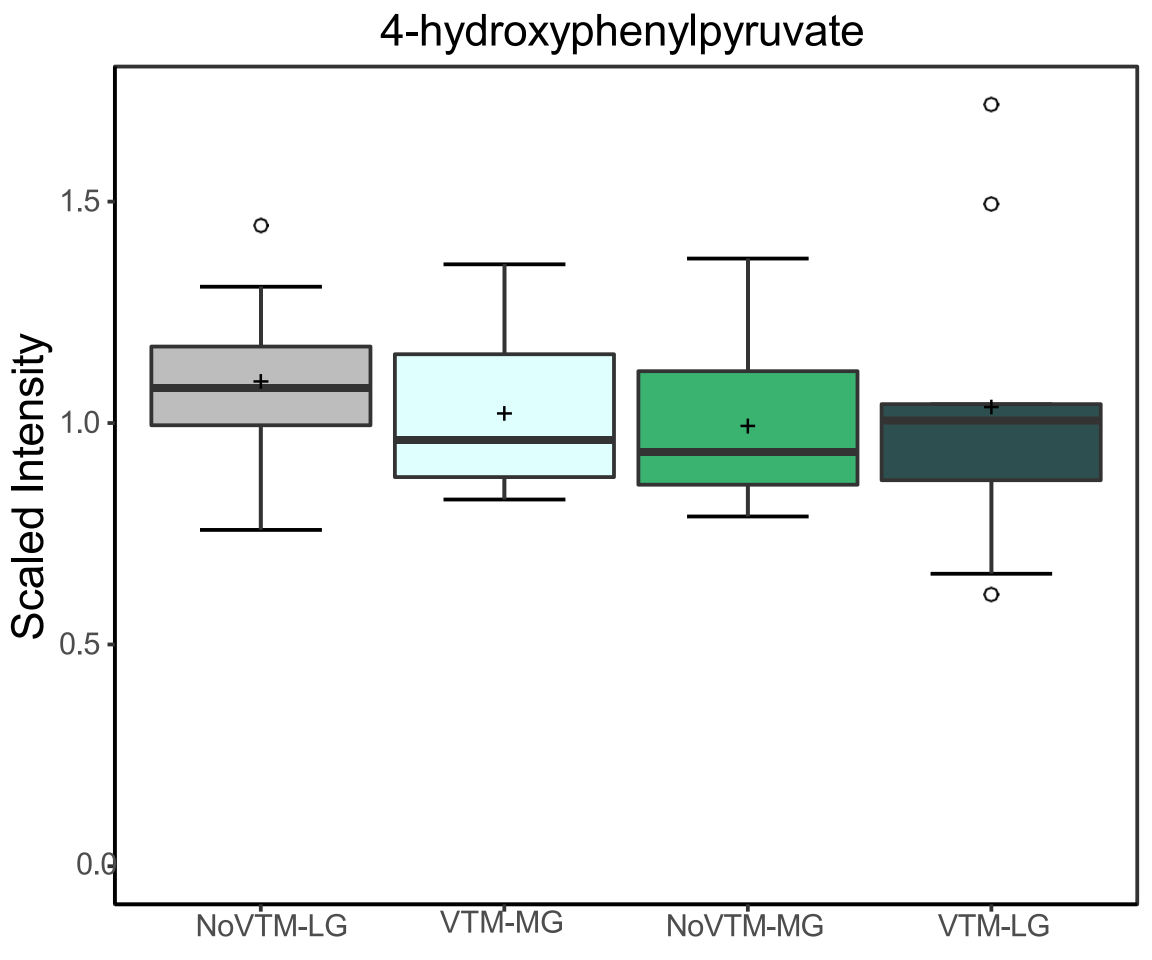

4-hydroxyphenylpyruvate
Scaled Intensity
1.5
1.0
0.5
0.0
NoVTM-LG
VTM-MG
NoVTM-MG
VTM-LG

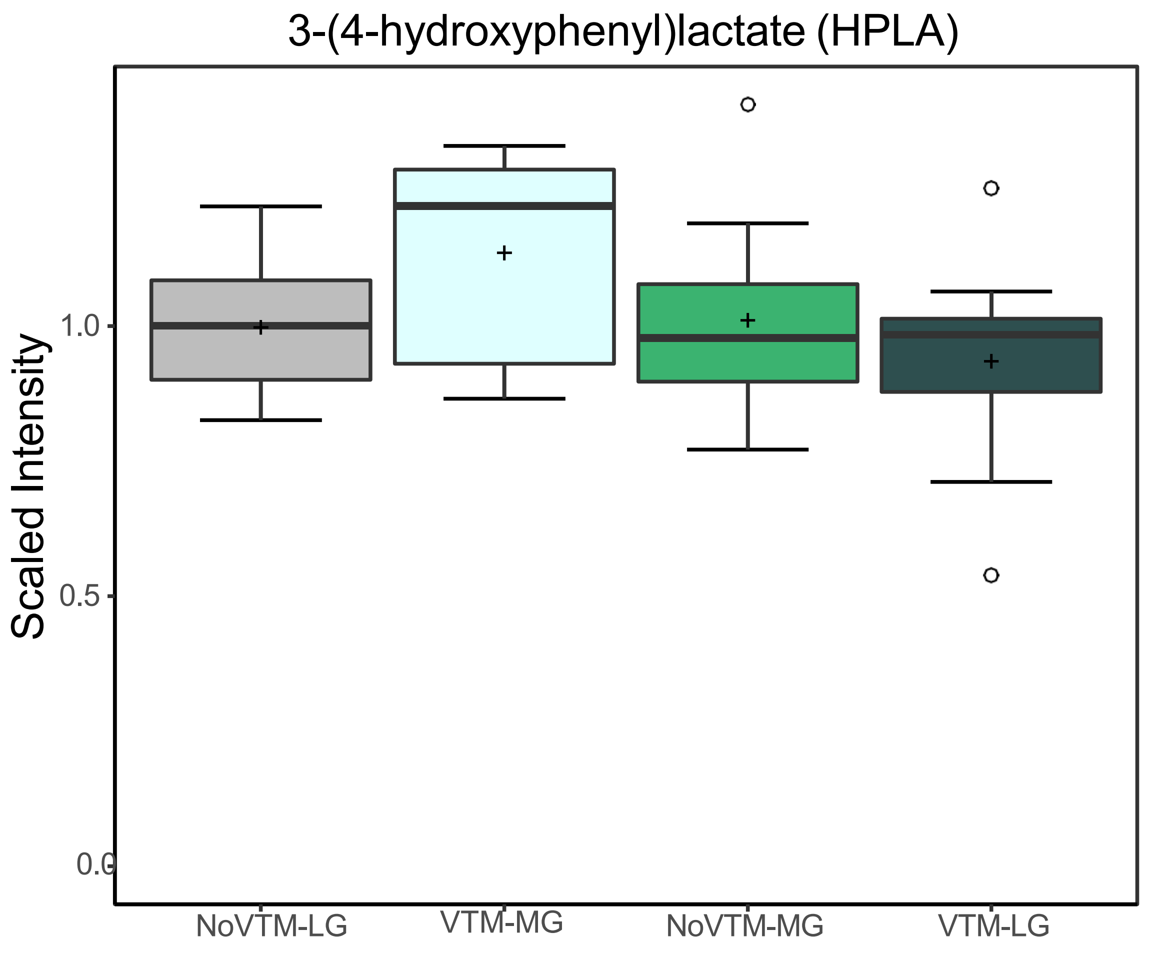
3-(4-hydroxyphenyl)lactate (HPLA)
Scaled Intensity
1.0
0.5
0.0
NoVTM-LG
VTM-MG
NoVTM-MG
VTM-LG

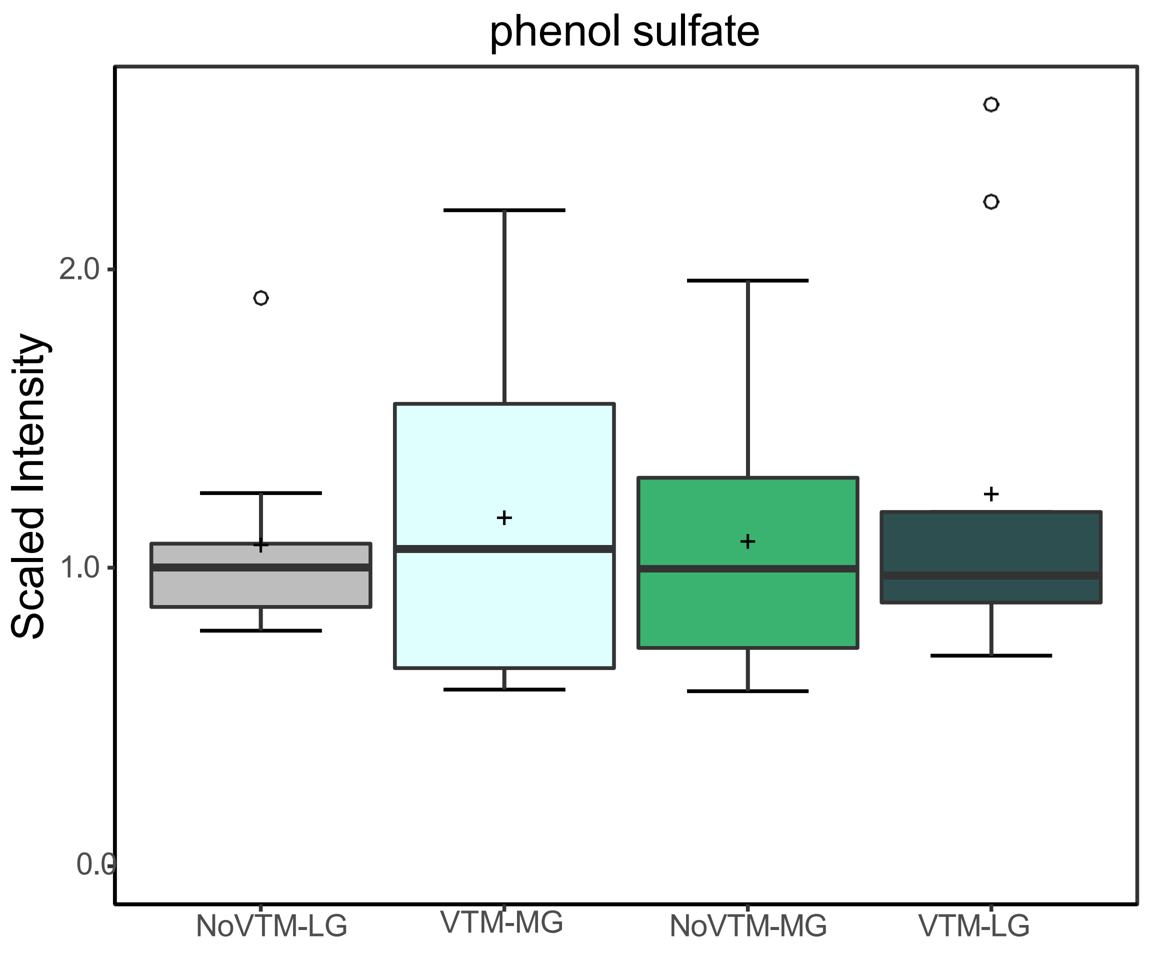
phenol sulfate
Scaled Intensity
2.0
1.0
0.0
NoVTM-LG
VTM-MG
NoVTM-MG
VTM-LG

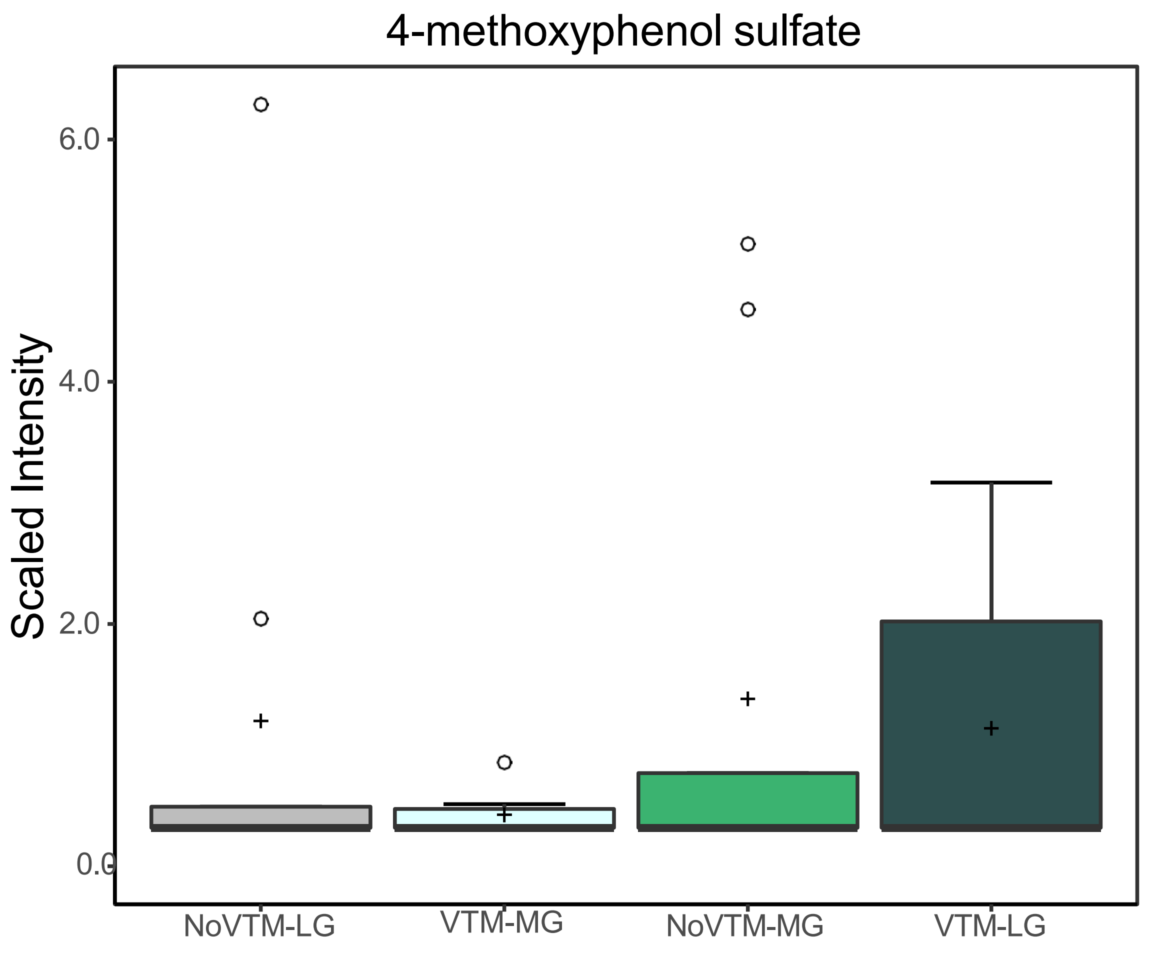
4-methoxyphenol sulfate
Scaled Intensity
6.0
4.0
2.0
0.0
NoVTM-LG
VTM-MG
NoVTM-MG
VTM-LG

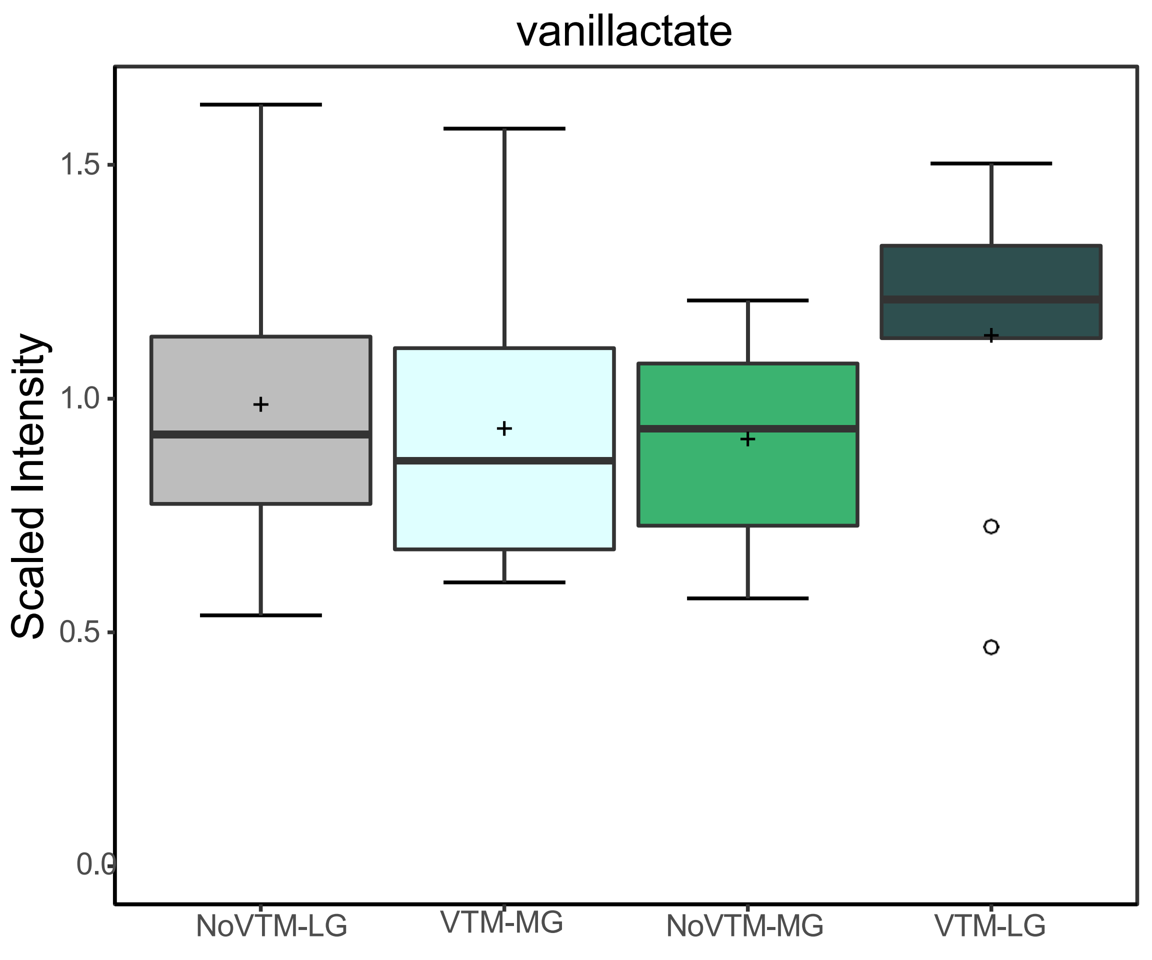
vanillactate
Scaled Intensity
1.5
1.0
0.5
0.0
NoVTM-LG
VTM-MG
NoVTM-MG
VTM-LG

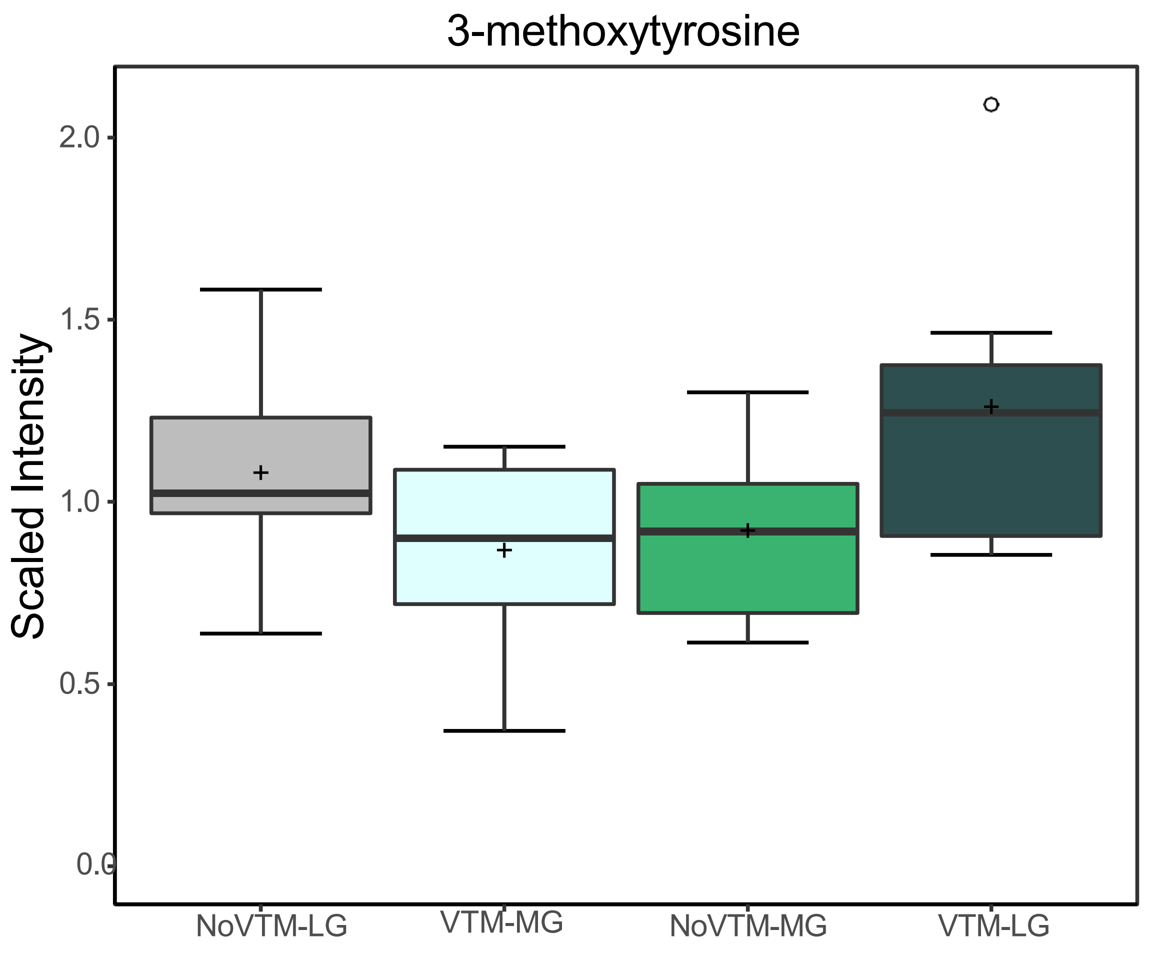
3-methoxytyrosine
Scaled Intensity
2.0
1.5
1.0
0.5
0.0
NoVTM-LG
VTM-MG
NoVTM-MG
VTM-LG

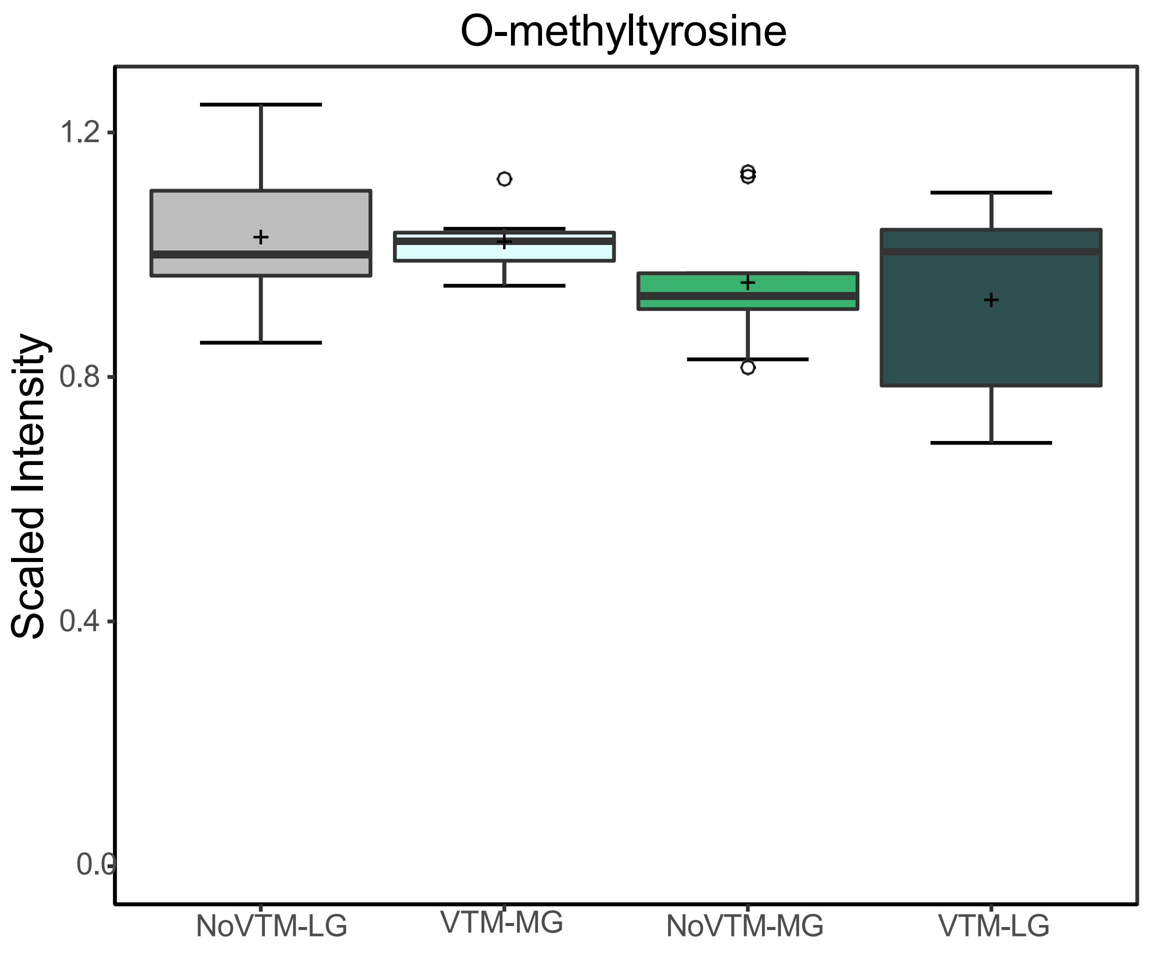

O-methyltyrosine
Scaled Intensity
1.2
0.8
0.4
0.0
NoVTM-LG
VTM-MG
NoVTM-MG
VTM-LG

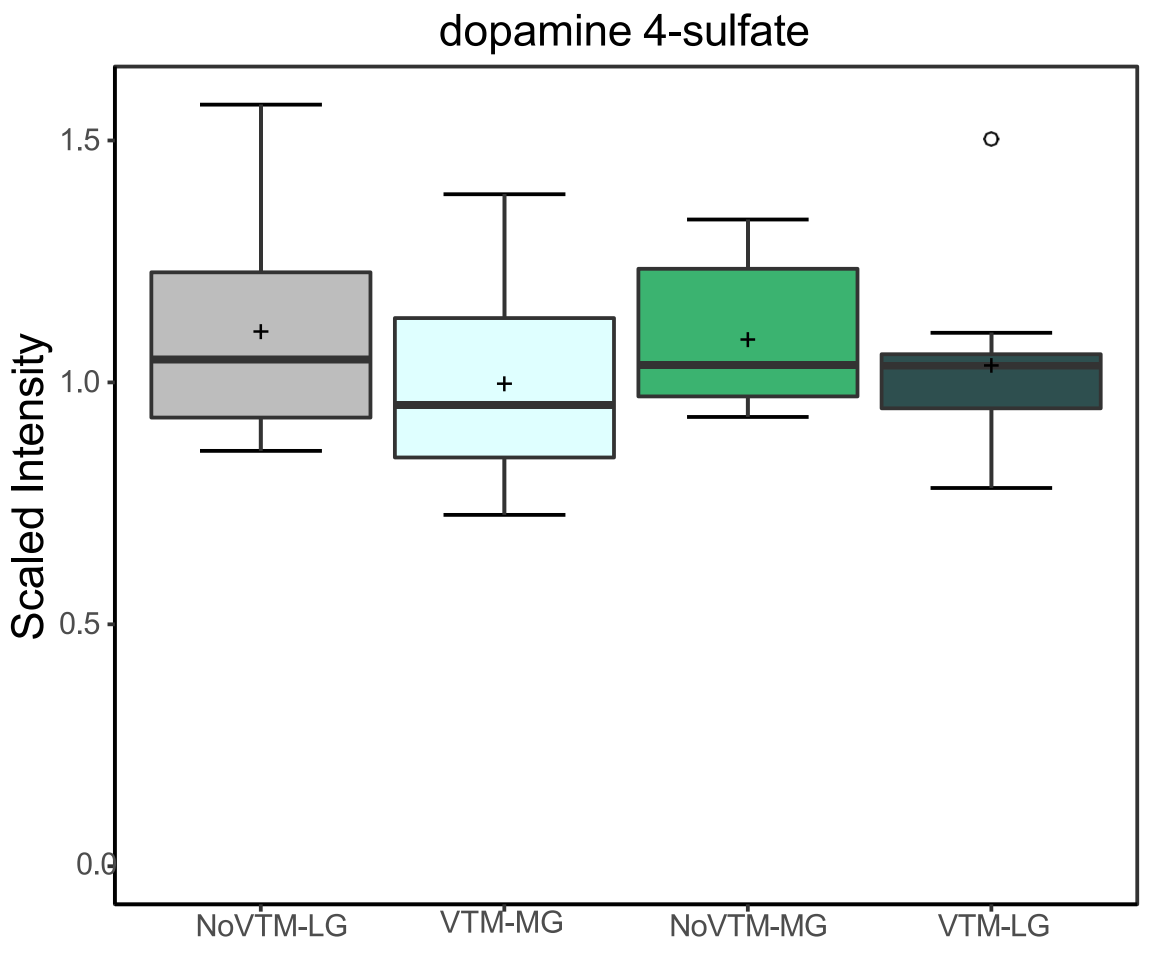

dopamine 4-sulfate
Scaled Intensity
1.5
1.0
0.5
0.0
NoVTM-LG
VTM-MG
NoVTM-MG
VTM-LG

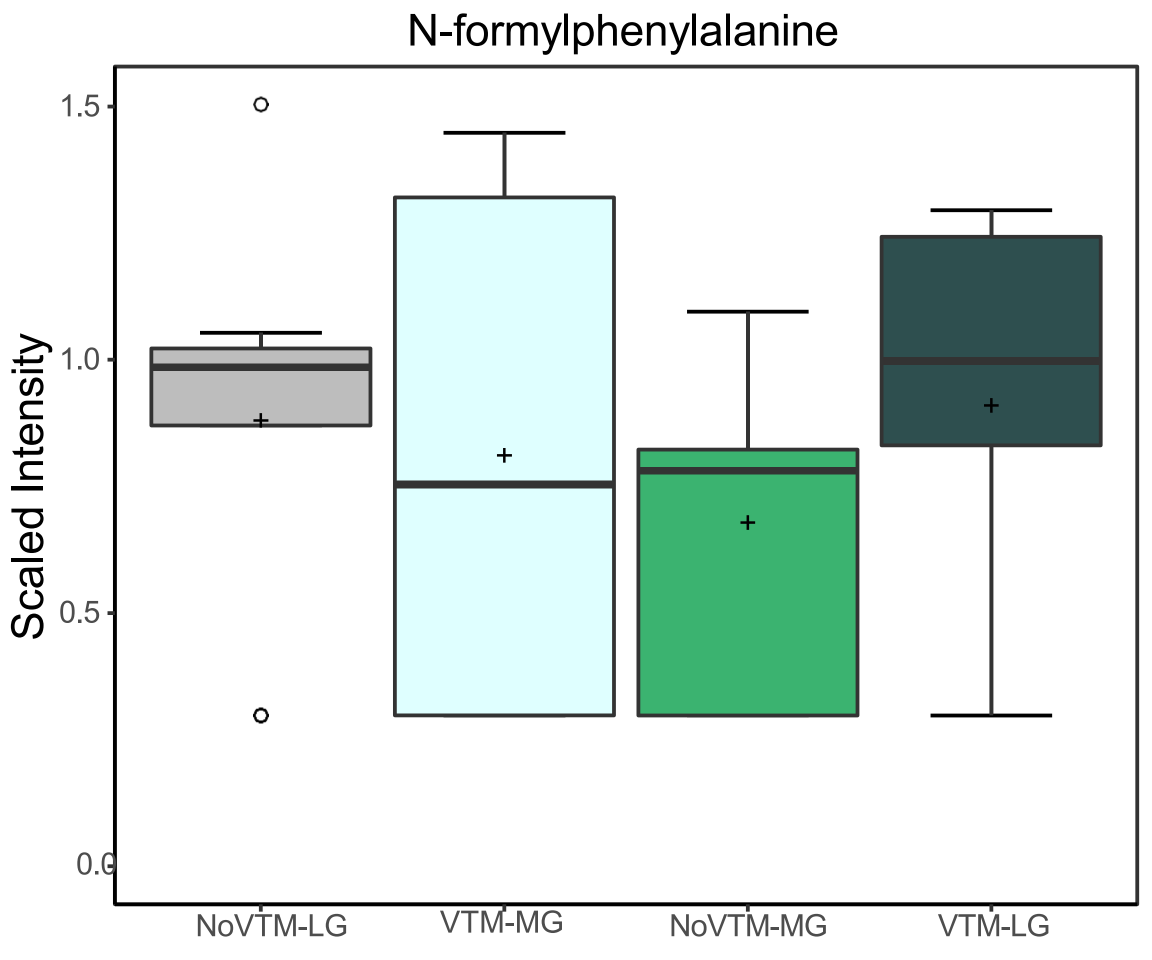

N-formylphenylalanine
Scaled Intensity
1.5
1.0
0.5
0.0
NoVTM-LG
VTM-MG
NoVTM-MG
VTM-LG

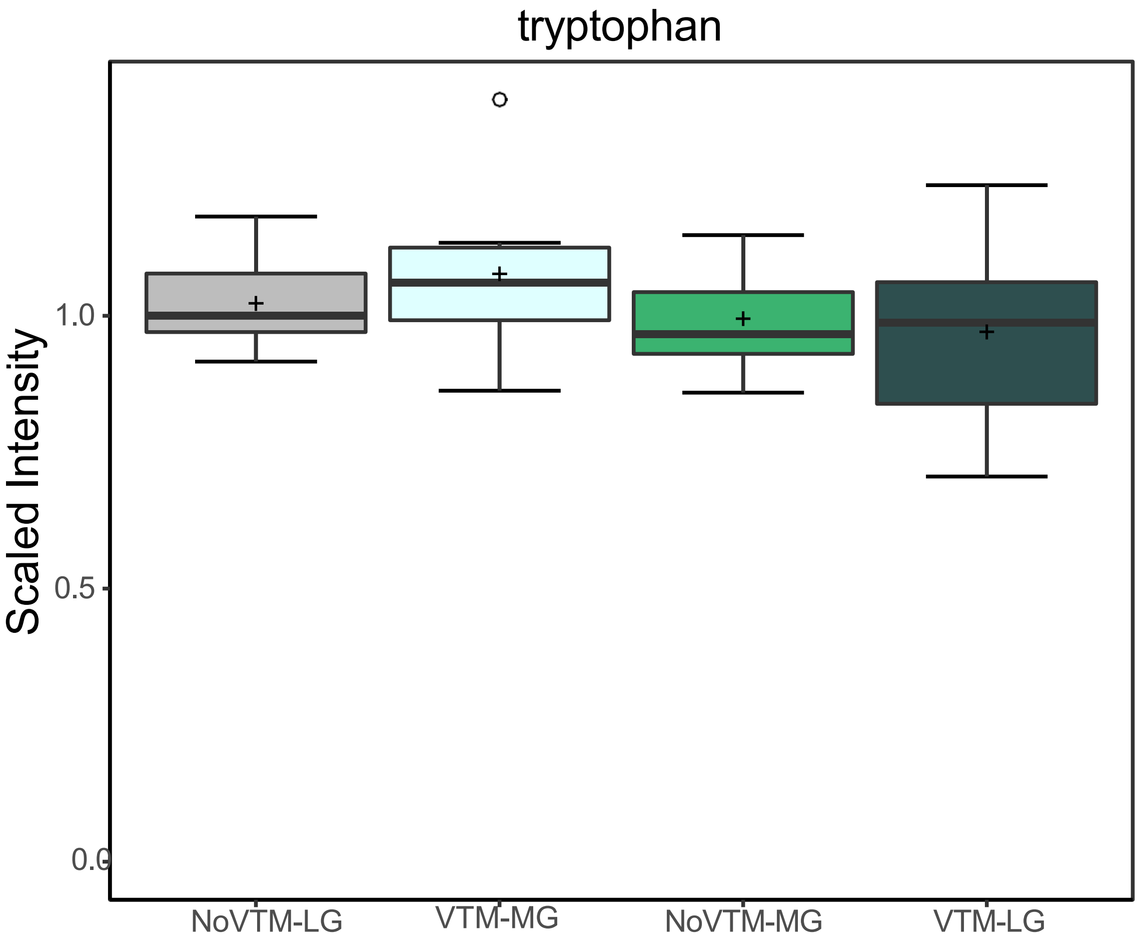

tryptophan
Scaled Intensity
1.0
0.5
0.0
NoVTM-LG
VTM-MG
NoVTM-MG
VTM-LG

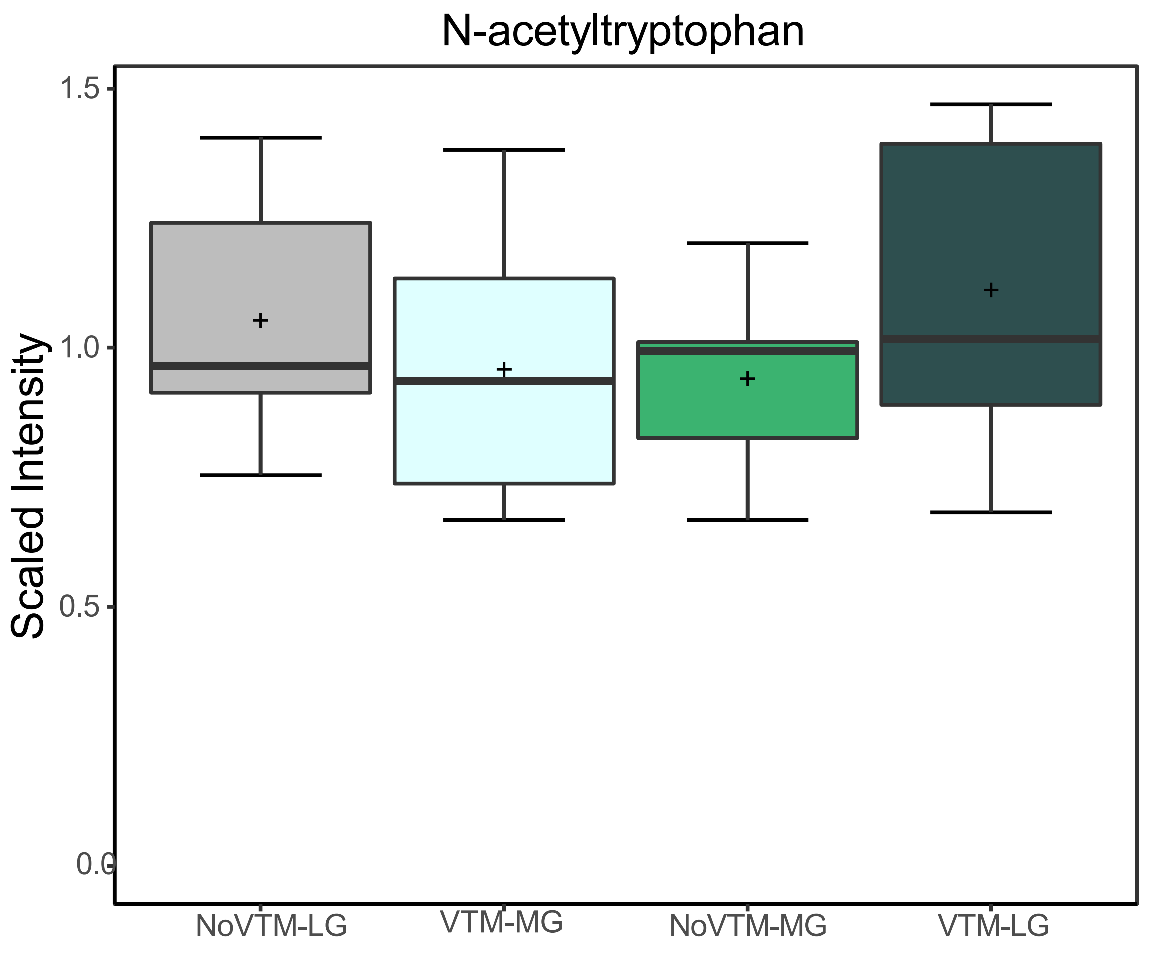
N-acetyltryptophan
Scaled Intensity
1.5
1.0
0.5
0.0
NoVTM-LG
VTM-MG
NoVTM-MG
VTM-LG

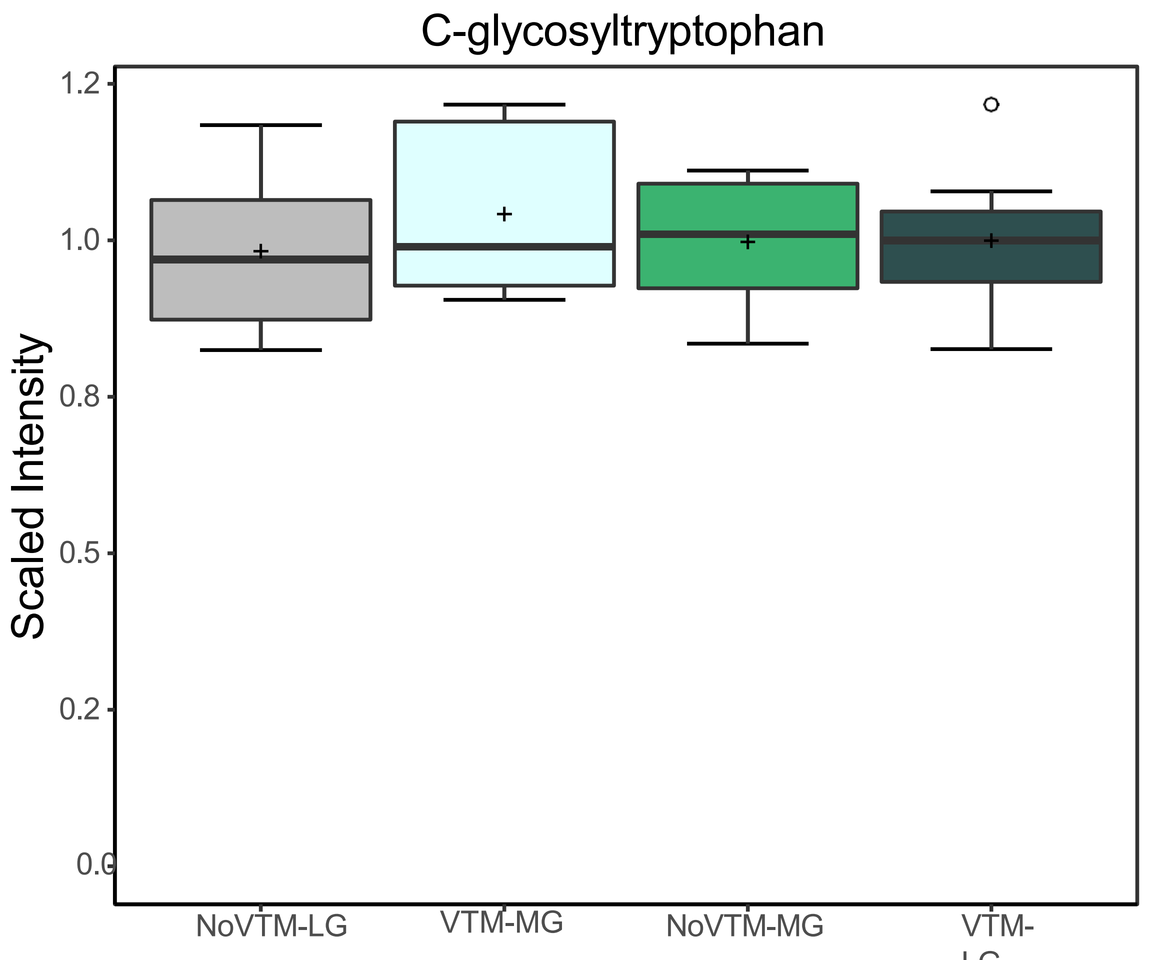
C-glycosyltryptophan
Scaled Intensity
1.2
1.0
0.8
0.5
0.2
0.0
NoVTM-LG
VTM-MG
NoVTM-MG
VTM-

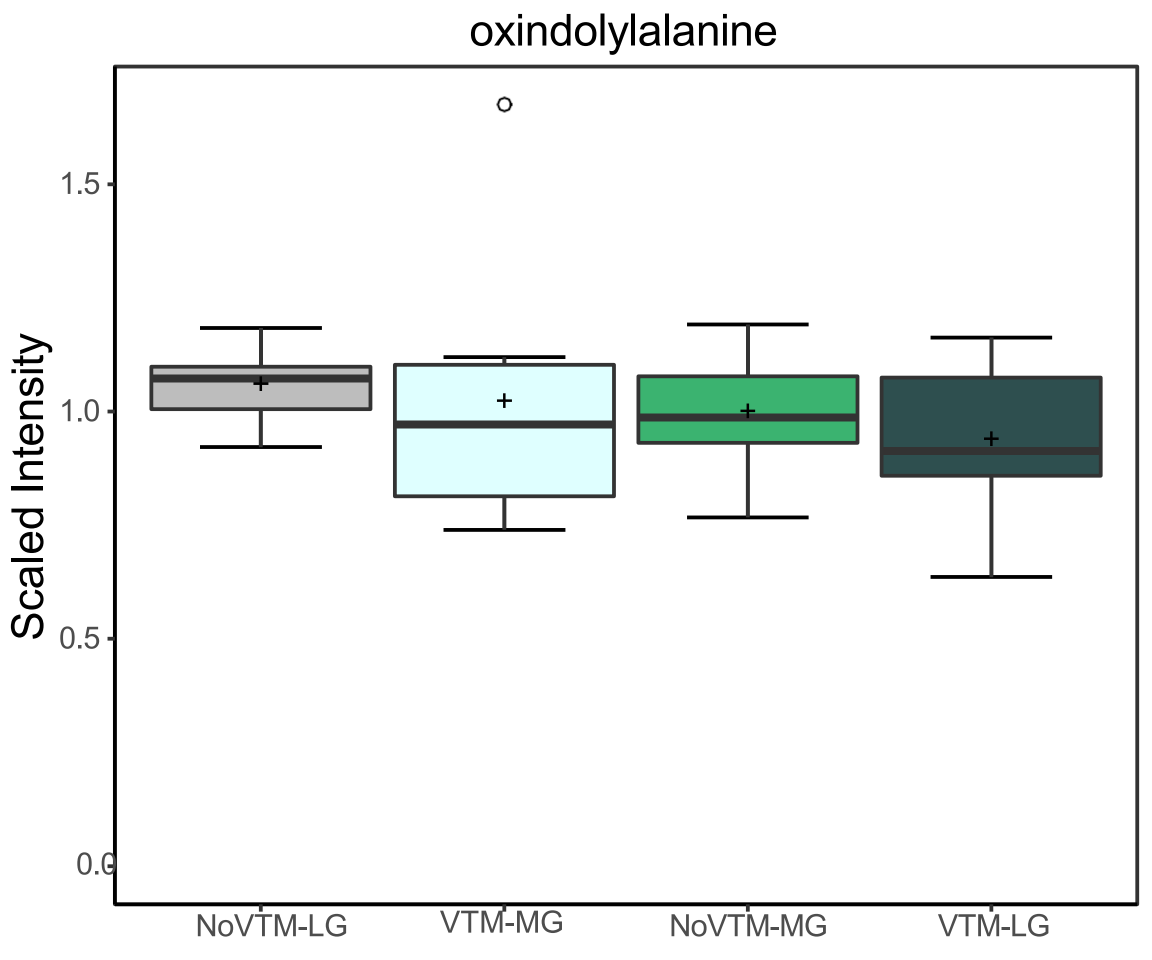
oxindolylalanine
Scaled Intensity
1.5
1.0
0.5
0.0
NoVTM-LG
VTM-MG
NoVTM-MG
VTM-LG

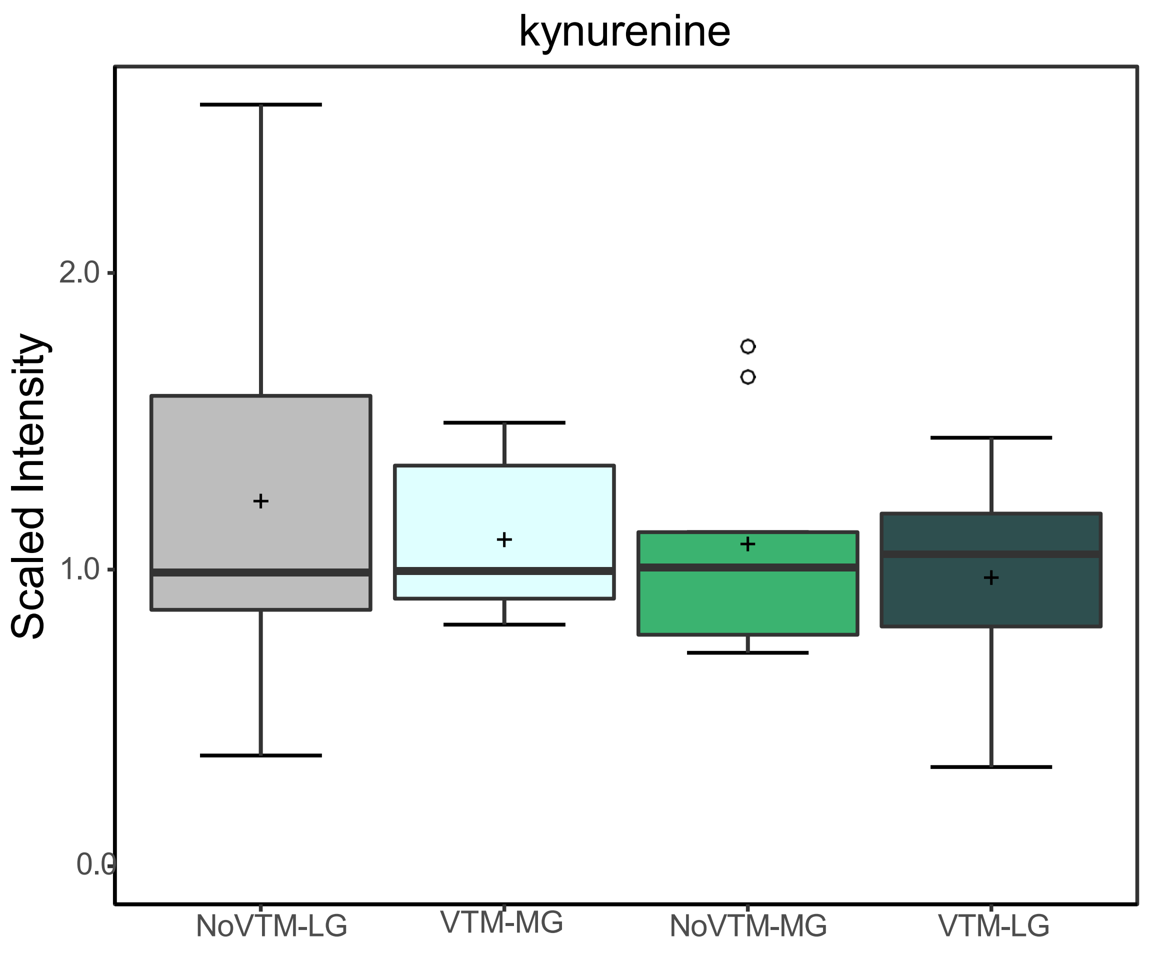
kynurenine
Scaled Intensity
2.0
1.0
0.0
NoVTM-LG
VTM-MG
NoVTM-MG
VTM-LG

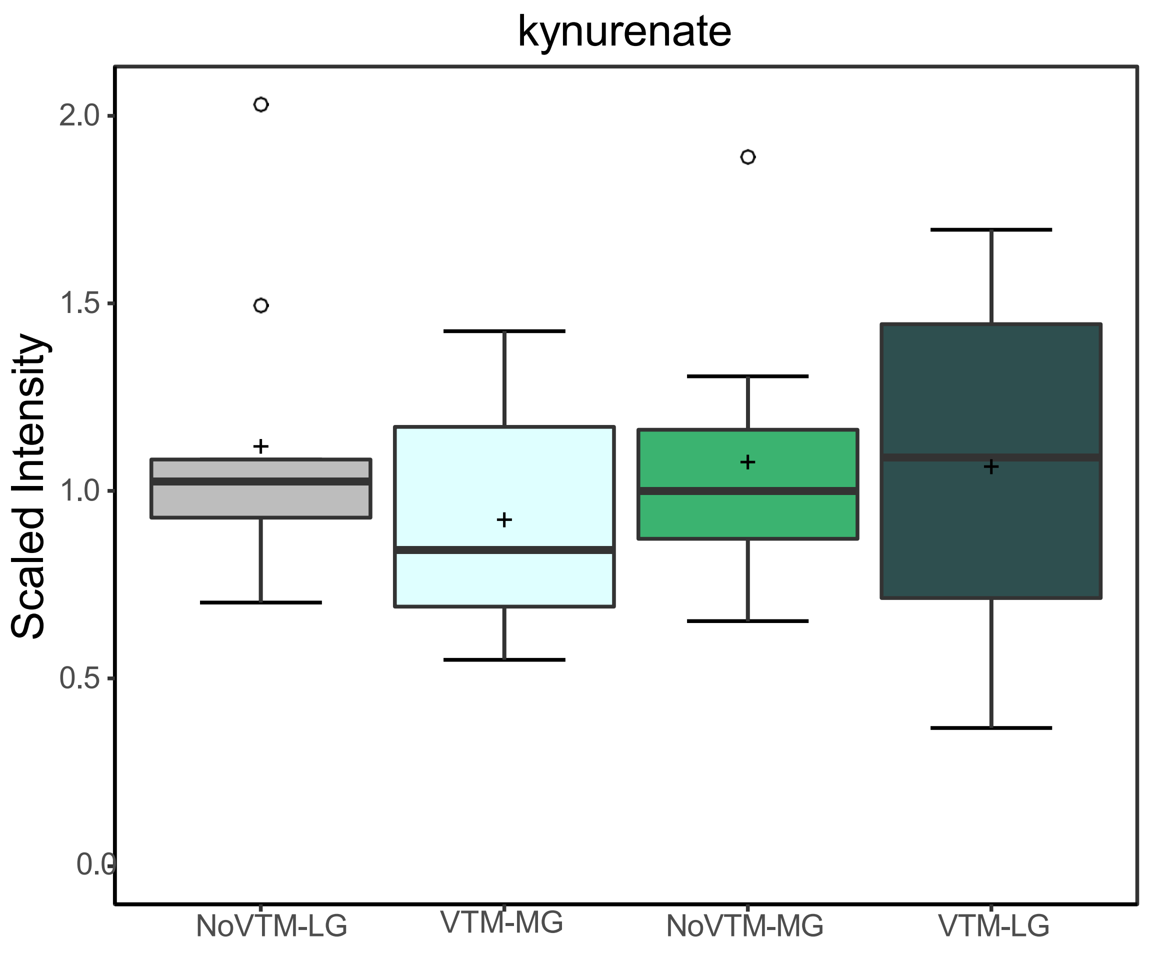
kynurenate
Scaled Intensity
2.0
1.5
1.0
0.5
0.0
NoVTM-LG
VTM-MG
NoVTM-MG
VTM-LG

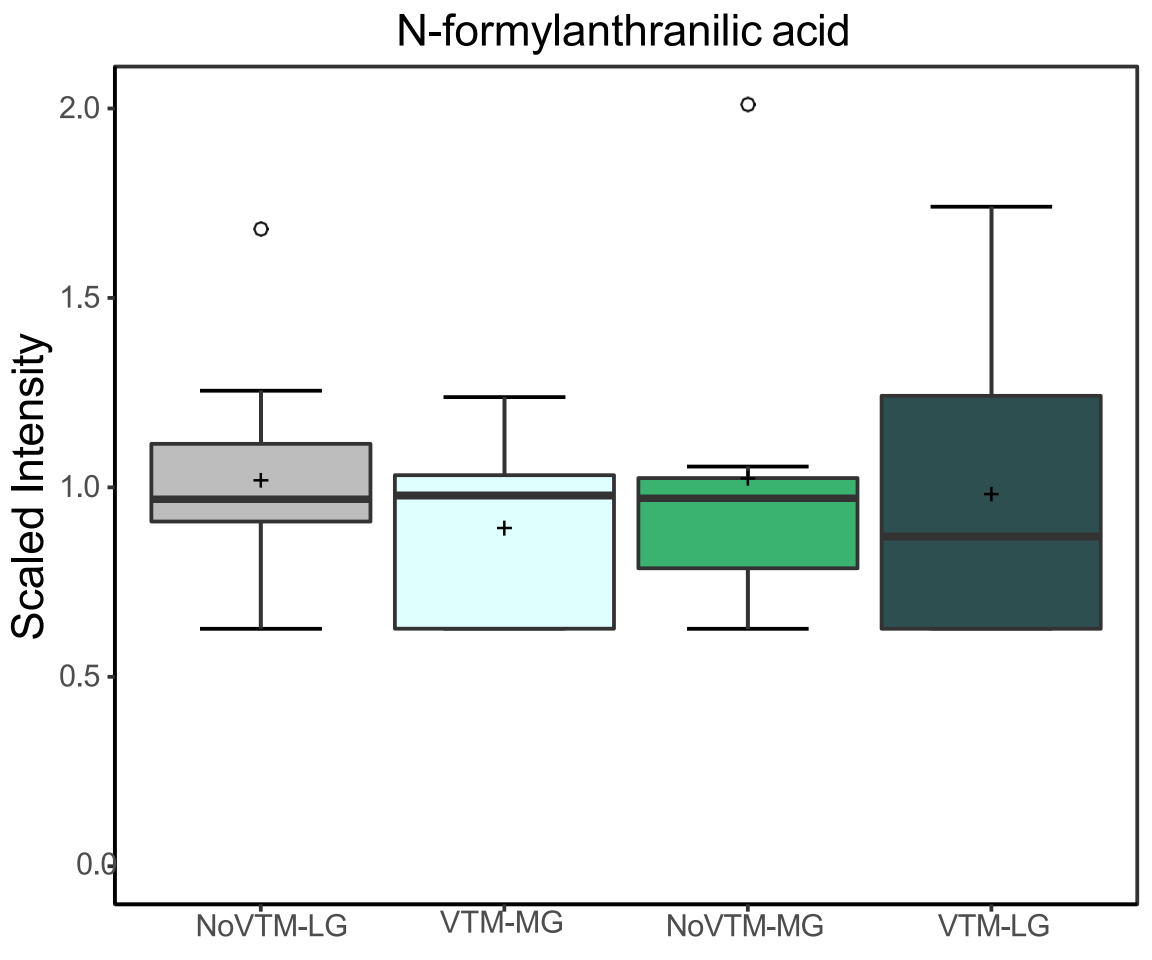

N-formylanthranilic acid
Scaled Intensity
2.0
1.5
1.0
0.5
0.0
NoVTM-LG
VTM-MG
NoVTM-MG
VTM-LG

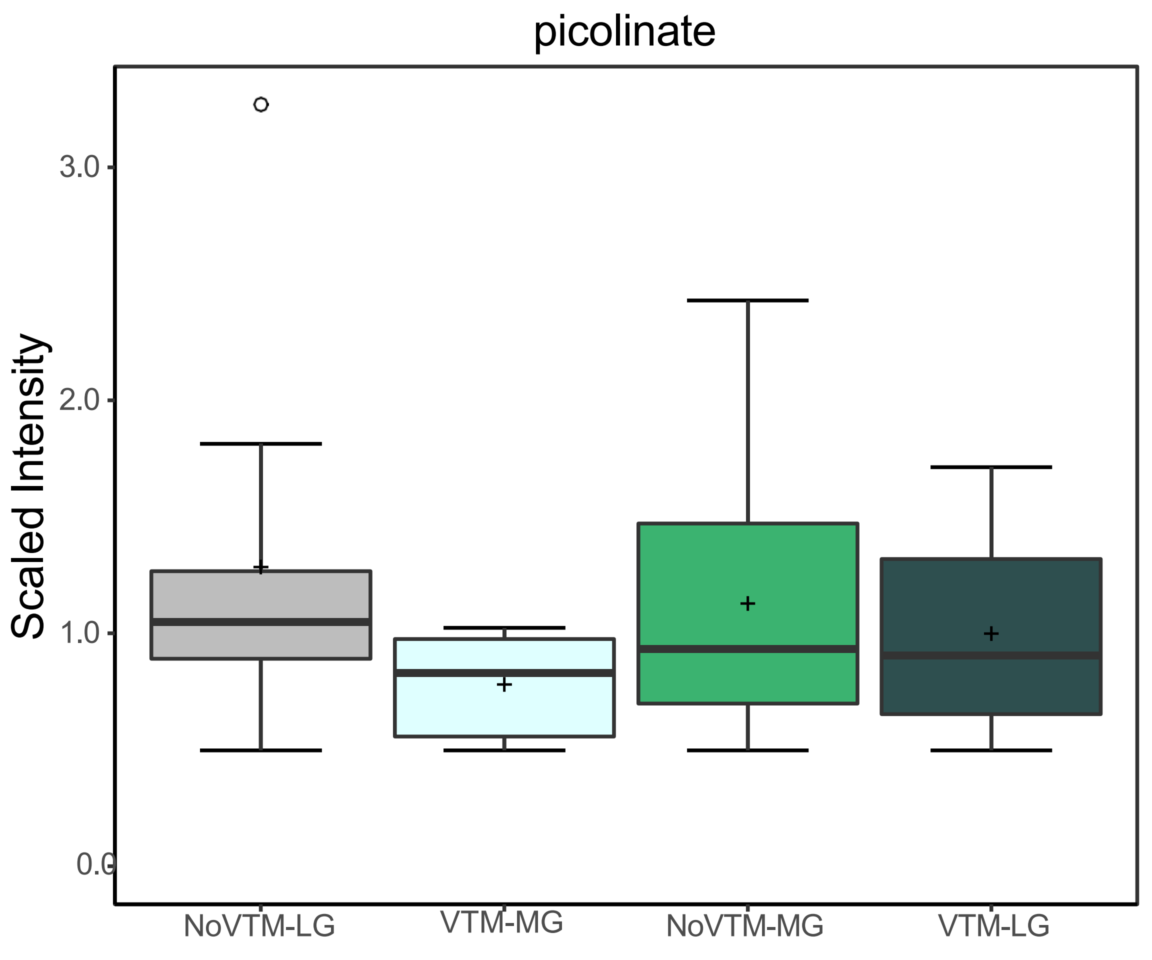
picolinate
Scaled Intensity
3.0
2.0
1.0
0.0
NoVTM-LG
VTM-MG
NoVTM-MG
VTM-LG

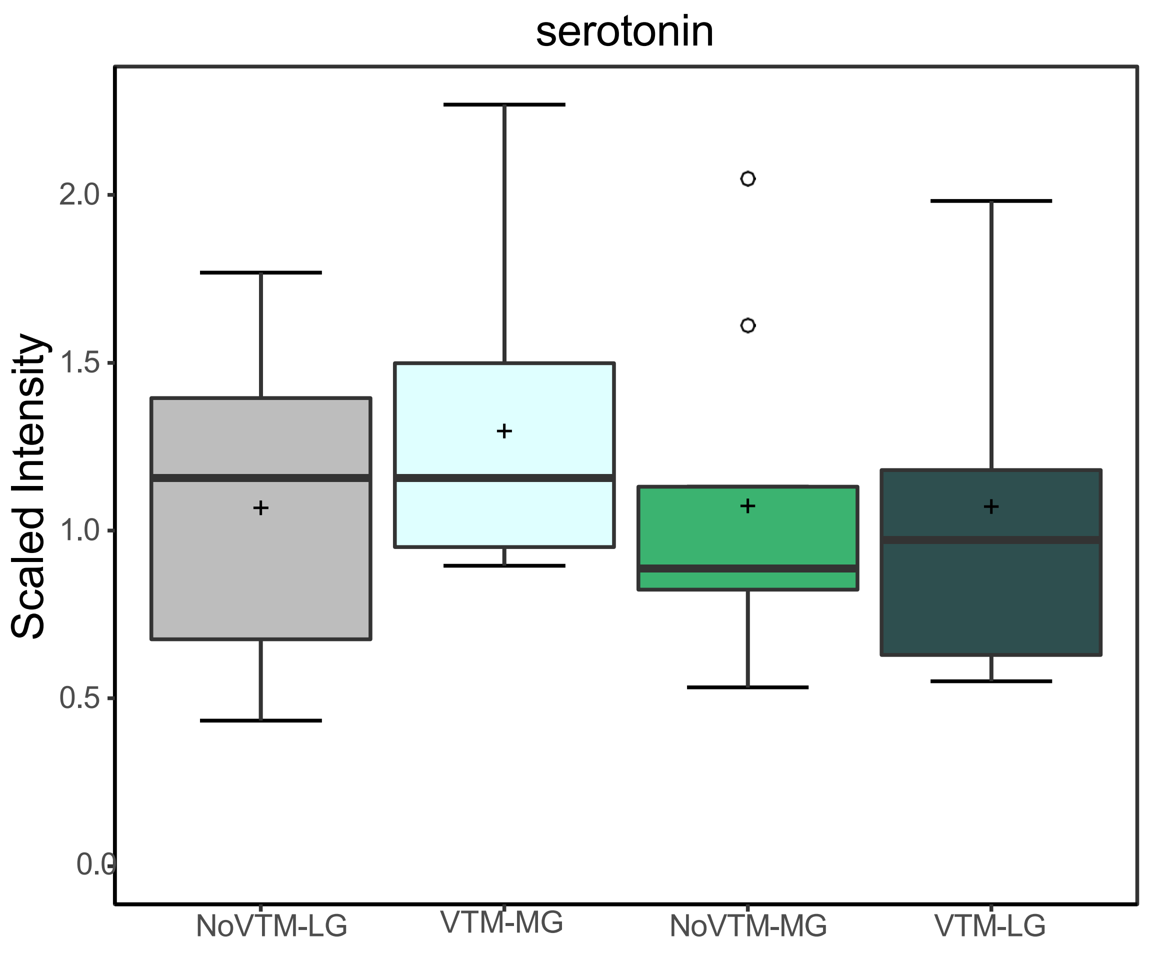
serotonin
Scaled Intensity
2.0
1.5
1.0
0.5
0.0
NoVTM-LG
VTM-MG
NoVTM-MG
VTM-LG

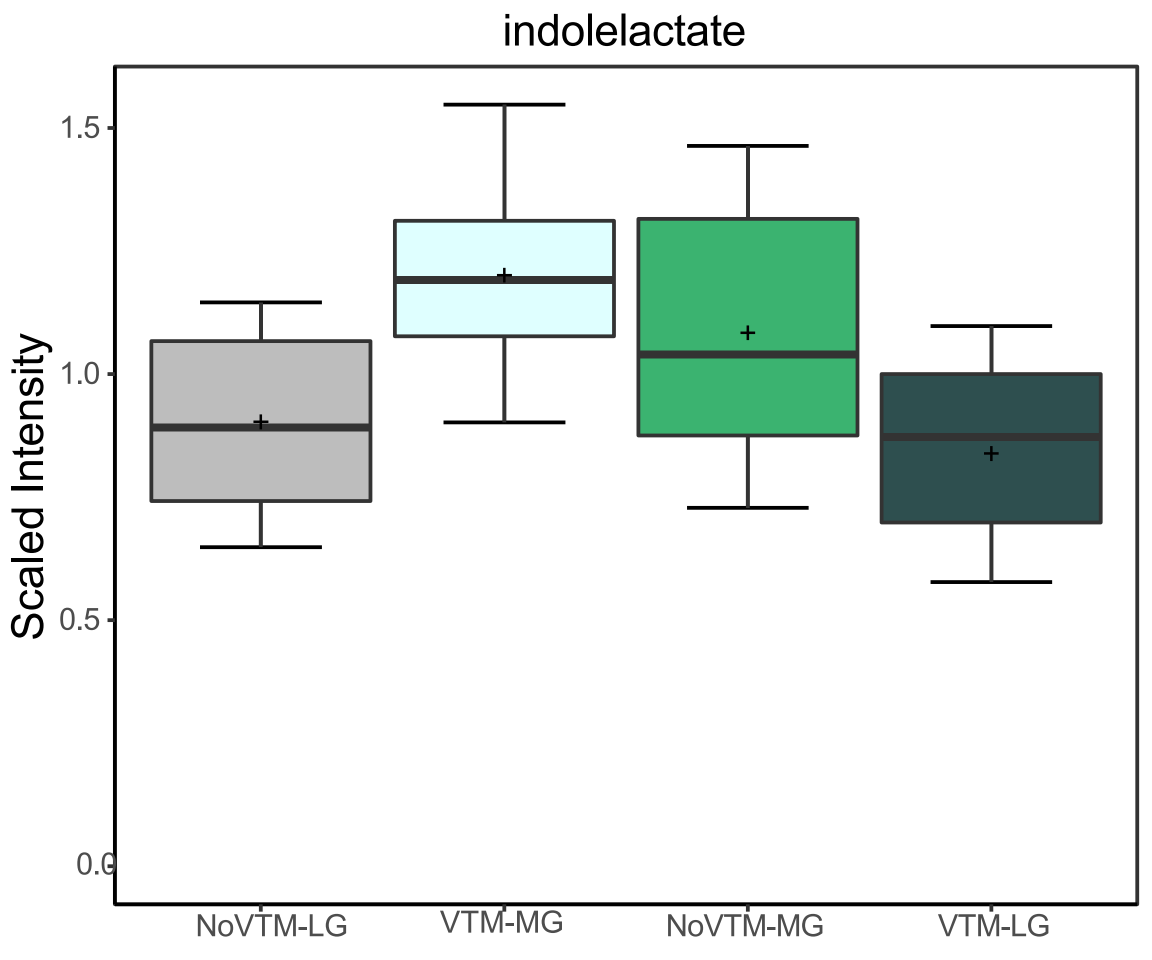

indolelactate
Scaled Intensity
1.5
1.0
0.5
0.0
NoVTM-LG
VTM-MG
NoVTM-MG
VTM-LG

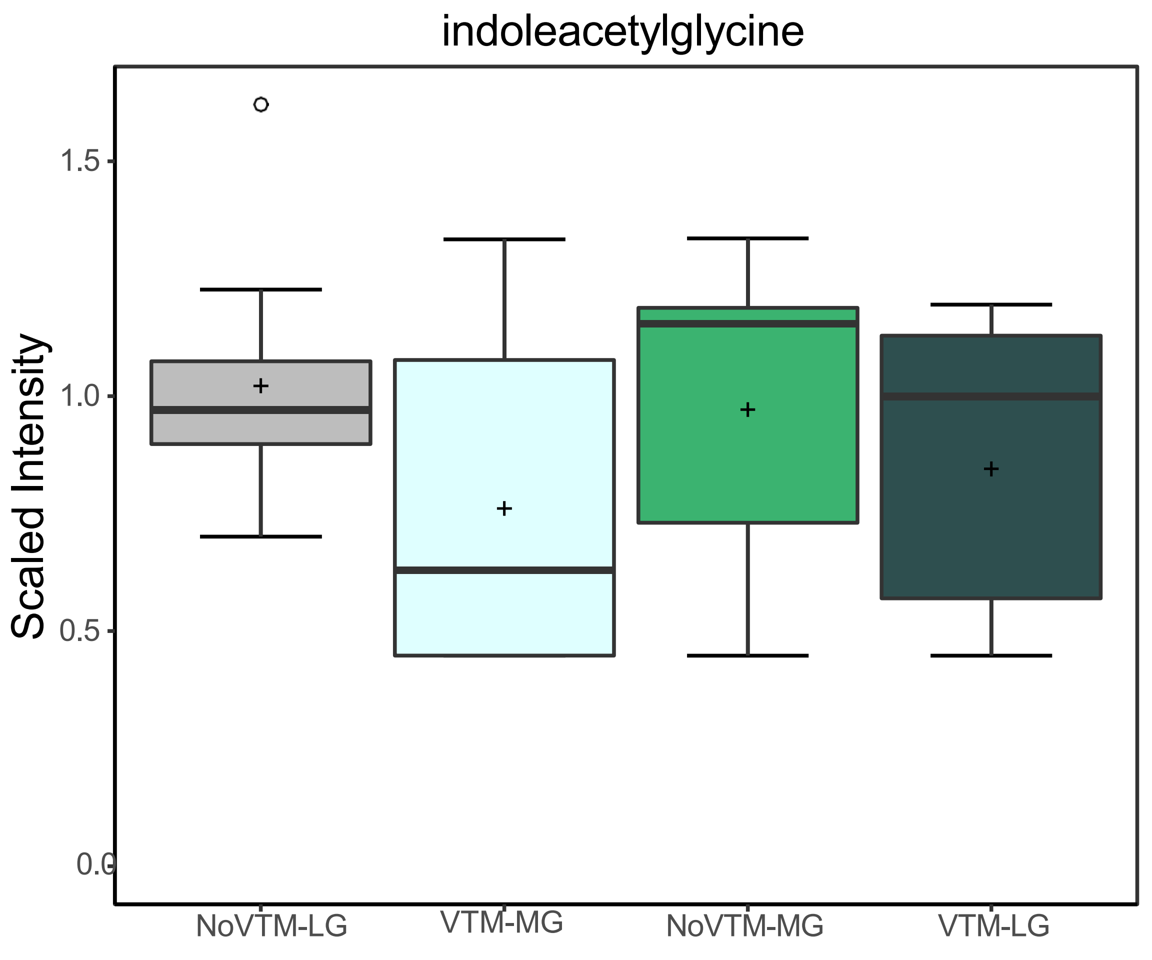

indoleacetylglycine
Scaled Intensity
1.5
1.0
0.5
0.0
NoVTM-LG
VTM-MG
NoVTM-MG
VTM-LG

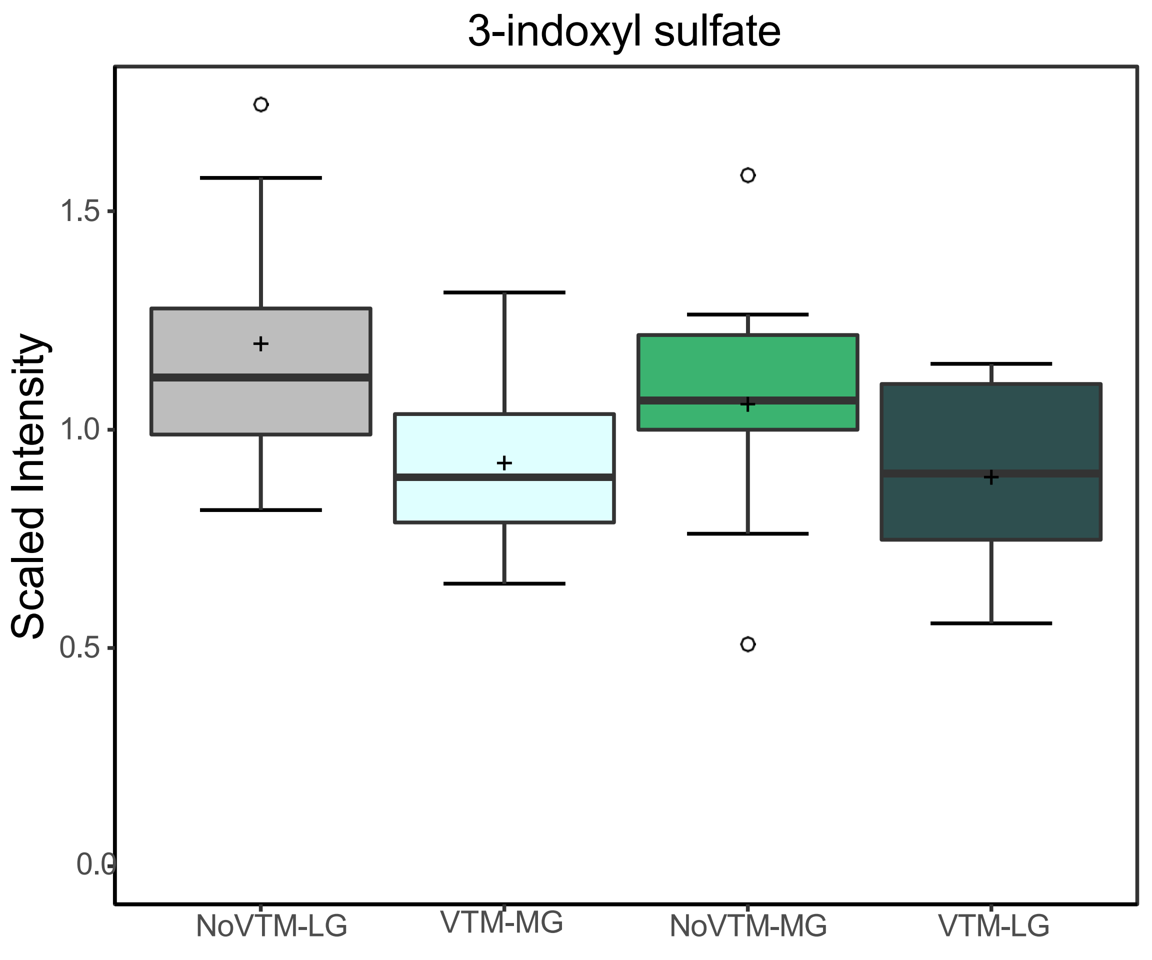
3-indoxyl sulfate
Scaled Intensity
1.5
1.0
0.5
0.0
NoVTM-LG
VTM-MG
NoVTM-MG
VTM-LG

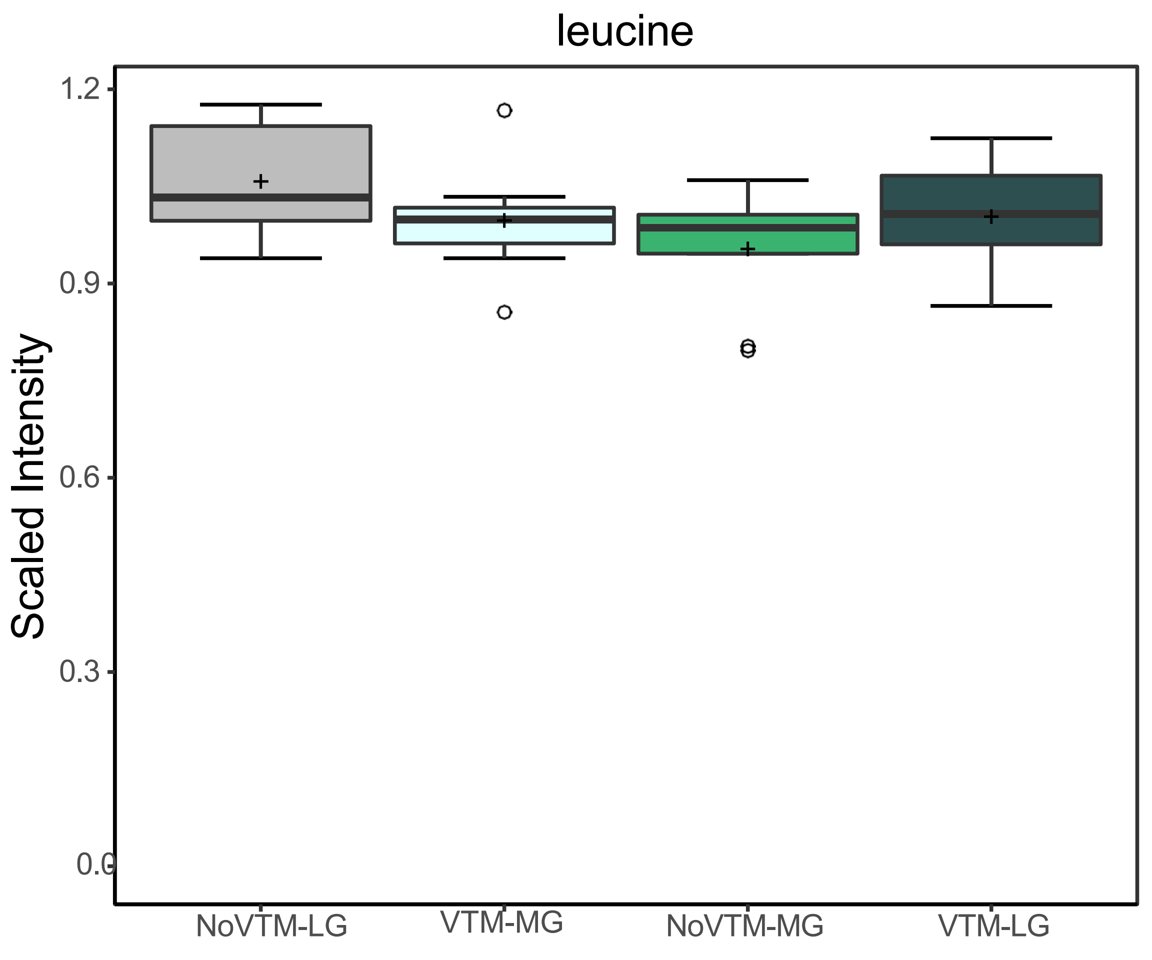
leucine
Scaled Intensity
1.2
0.9
0.6
0.3
0.0
NoVTM-LG
VTM-MG
NoVTM-MG
VTM-LG

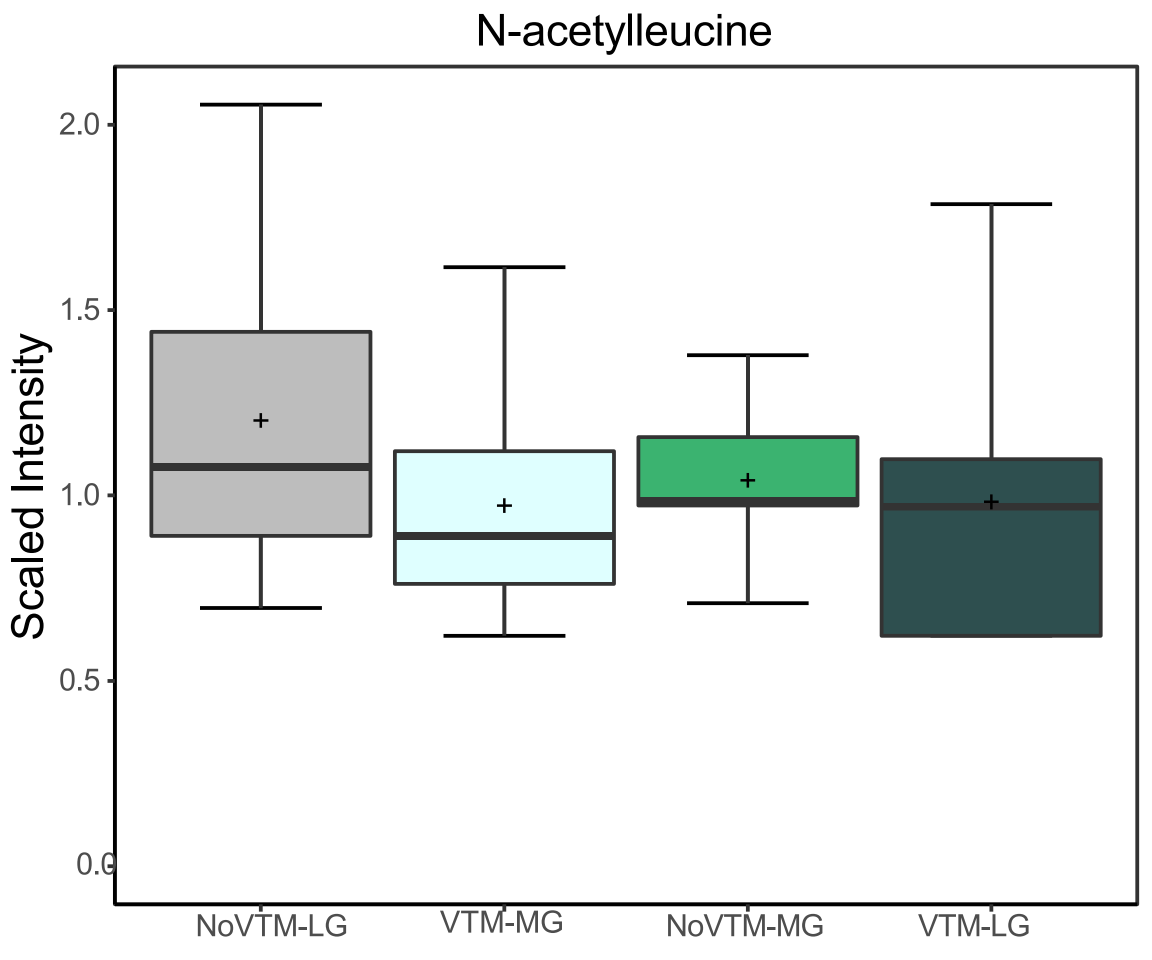
N-acetylleucine
Scaled Intensity
2.0
1.5
1.0
0.5
0.0
NoVTM-LG
VTM-MG
NoVTM-MG
VTM-LG

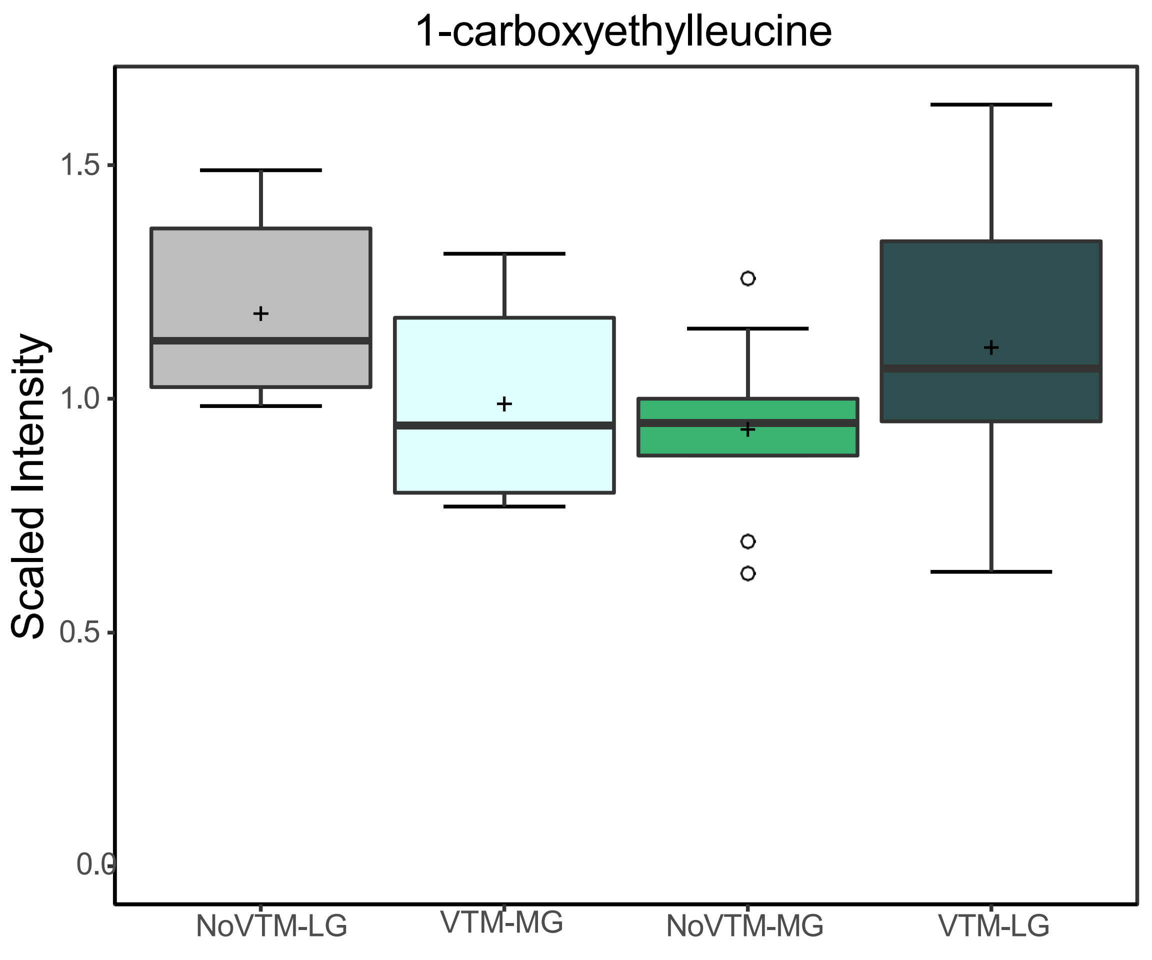

1-carboxyethylleucine
Scaled Intensity
1.5
1.0
0.5
0.0
NoVTM-LG
VTM-MG
NoVTM-MG
VTM-LG

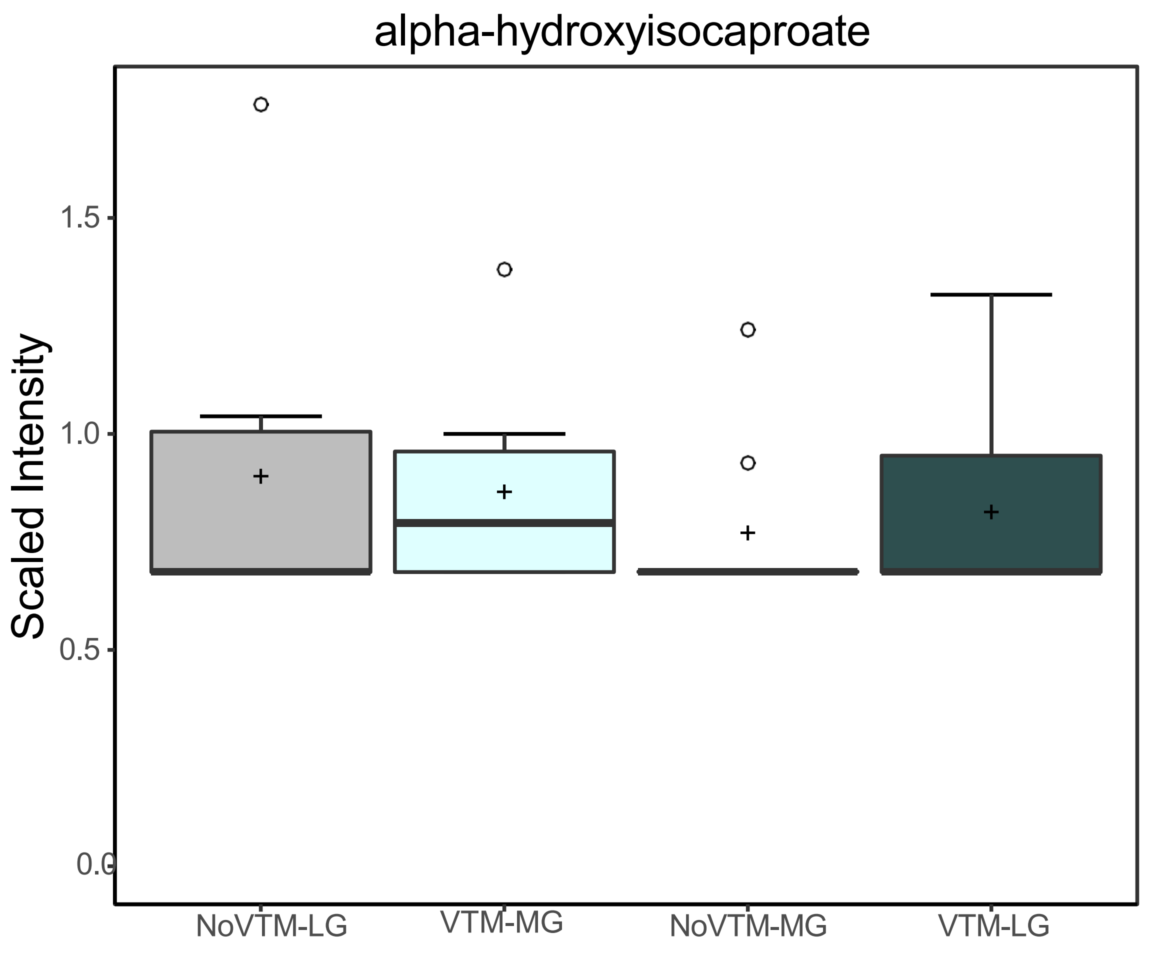

alpha-hydroxyisocaproate
Scaled Intensity
1.5
1.0
0.5
0.0
NoVTM-LG
VTM-MG
NoVTM-MG
VTM-LG

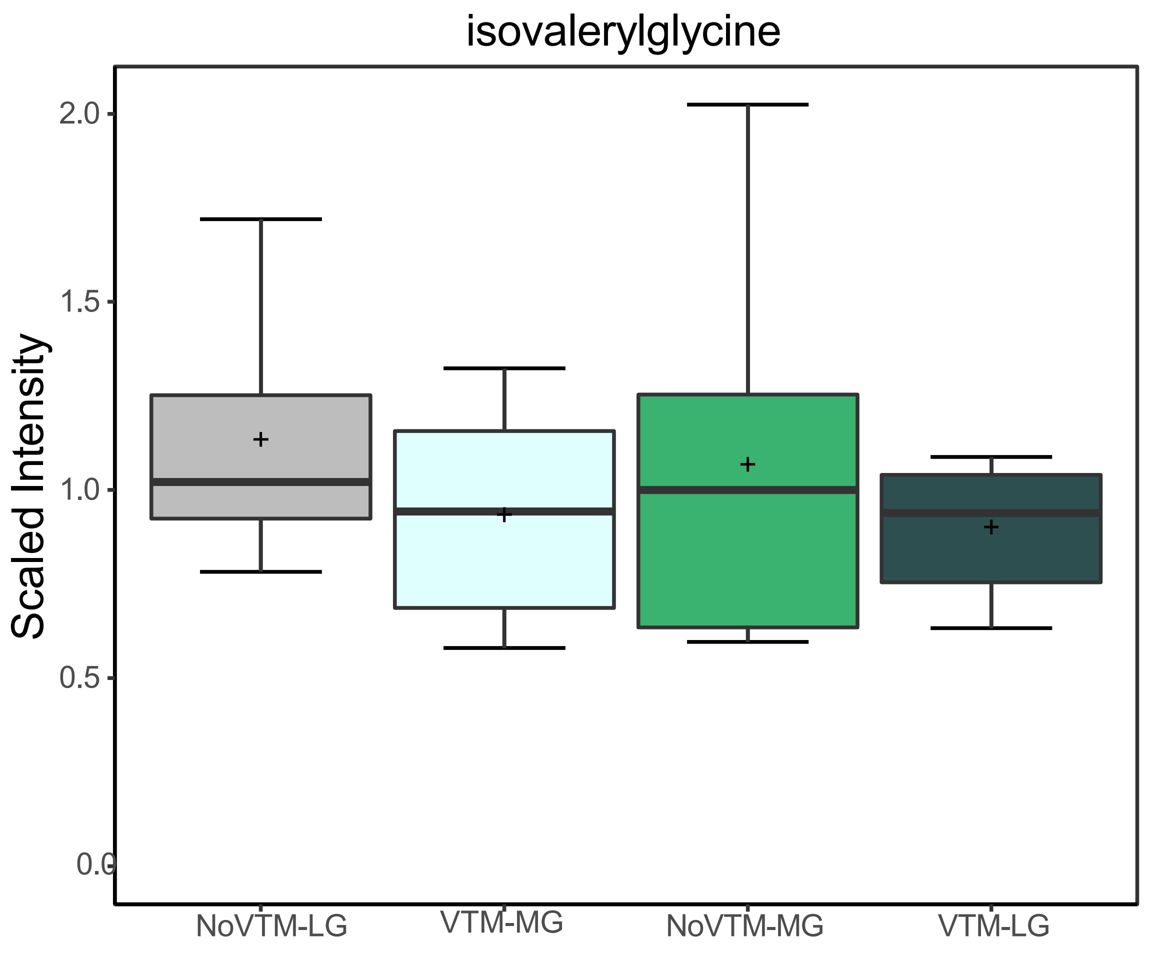

isovalerylglycine
Scaled Intensity
2.0
1.5
1.0
0.5
0.0
NoVTM-LG
VTM-MG
NoVTM-MG
VTM-LG

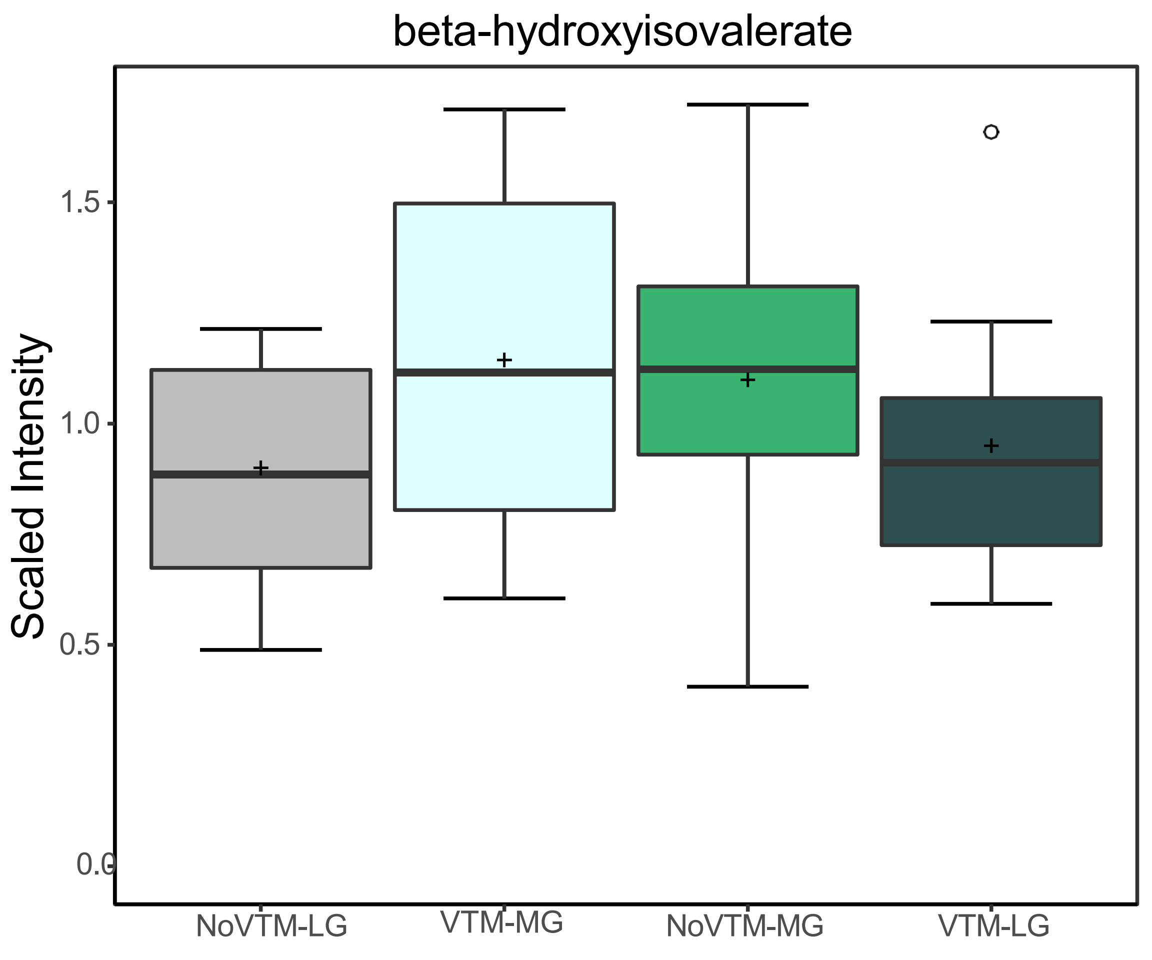
beta-hydroxyisovalerate
Scaled Intensity
1.5
1.0
0.5
0.0
NoVTM-LG
VTM-MG
NoVTM-MG
VTM-LG

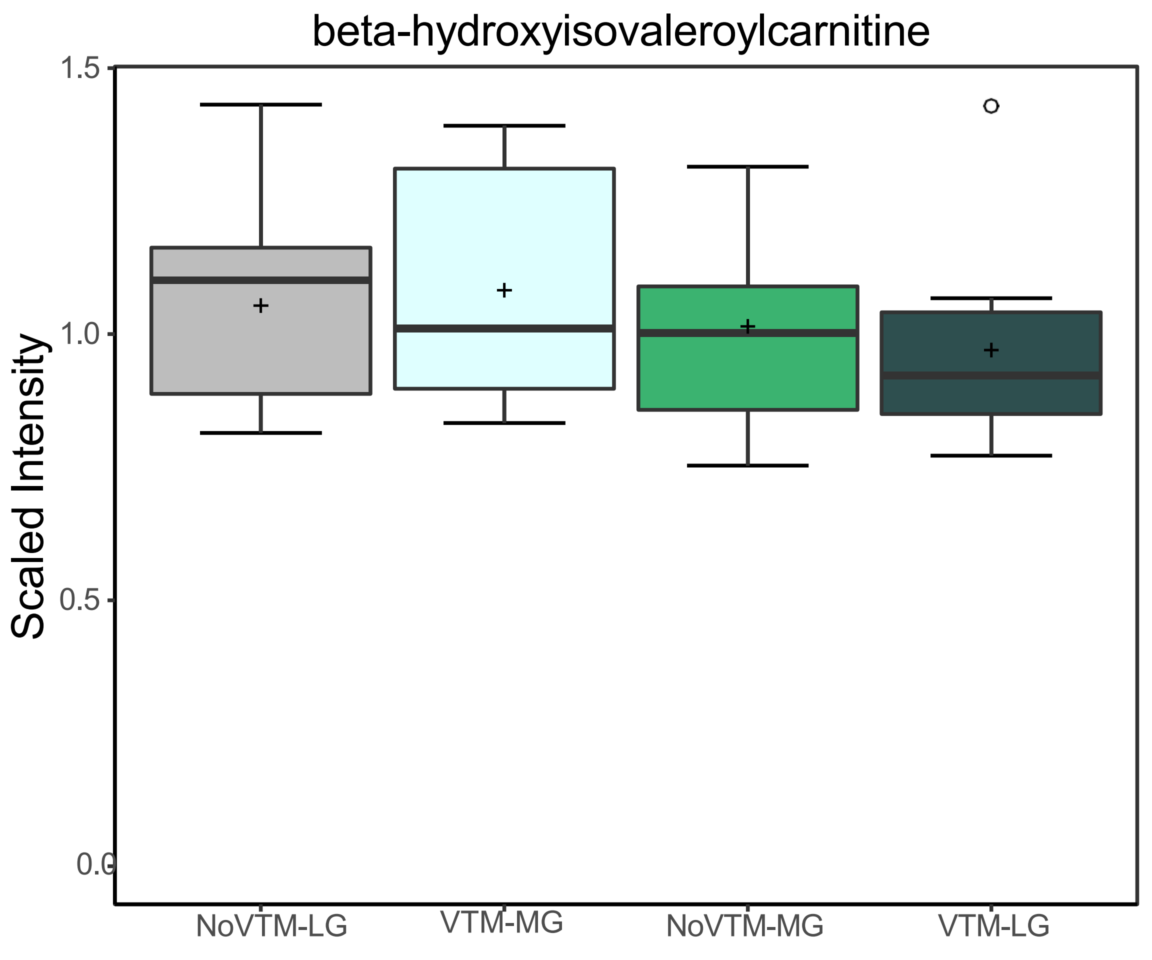
beta-hydroxyisovaleroylcarnitine
Scaled Intensity
1.5
1.0
0.5
0.0
NoVTM-LG
VTM-MG
NoVTM-MG
VTM-LG

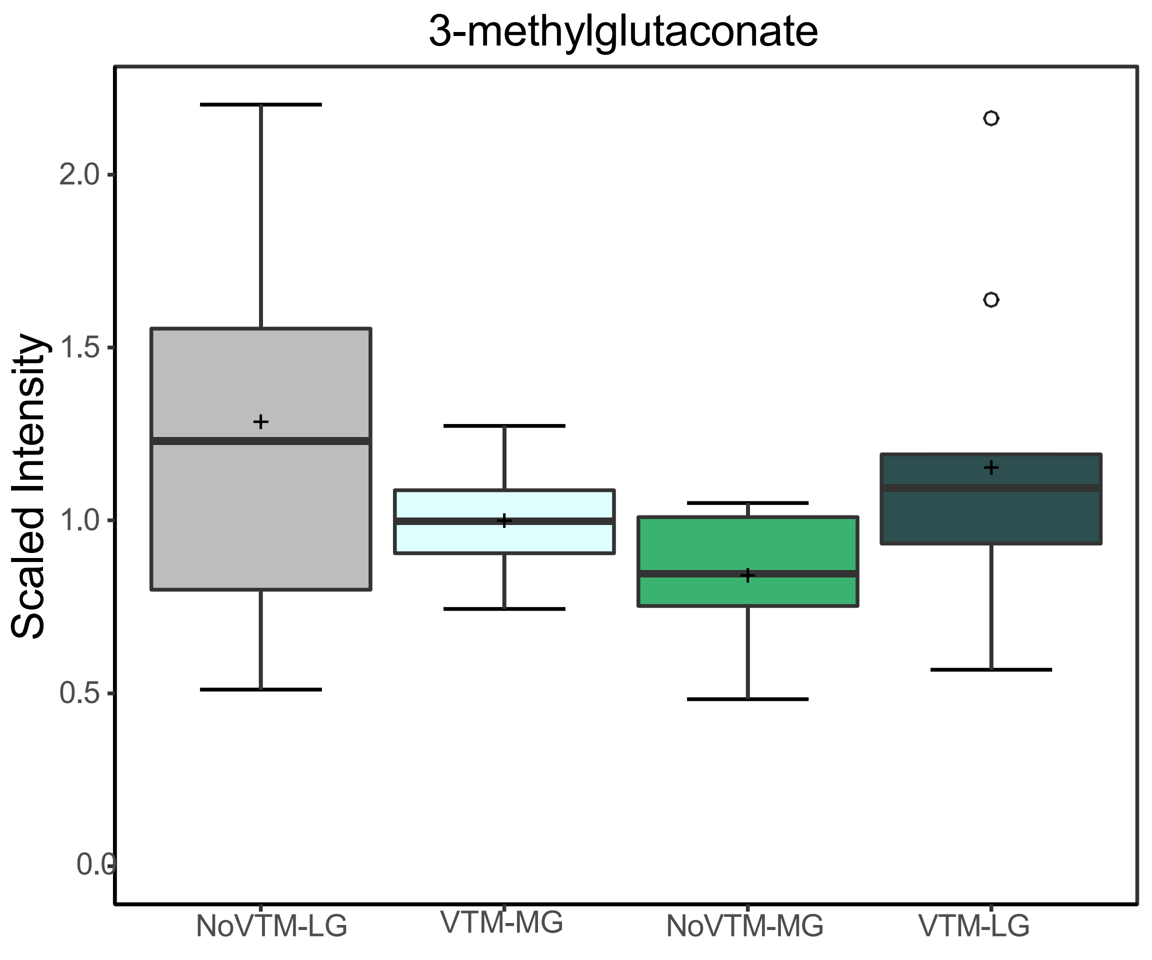
3-methylglutaconate
Scaled Intensity
2.0
1.5
1.0
0.5
0.0
NoVTM-LG
VTM-MG
NoVTM-MG
VTM-LG

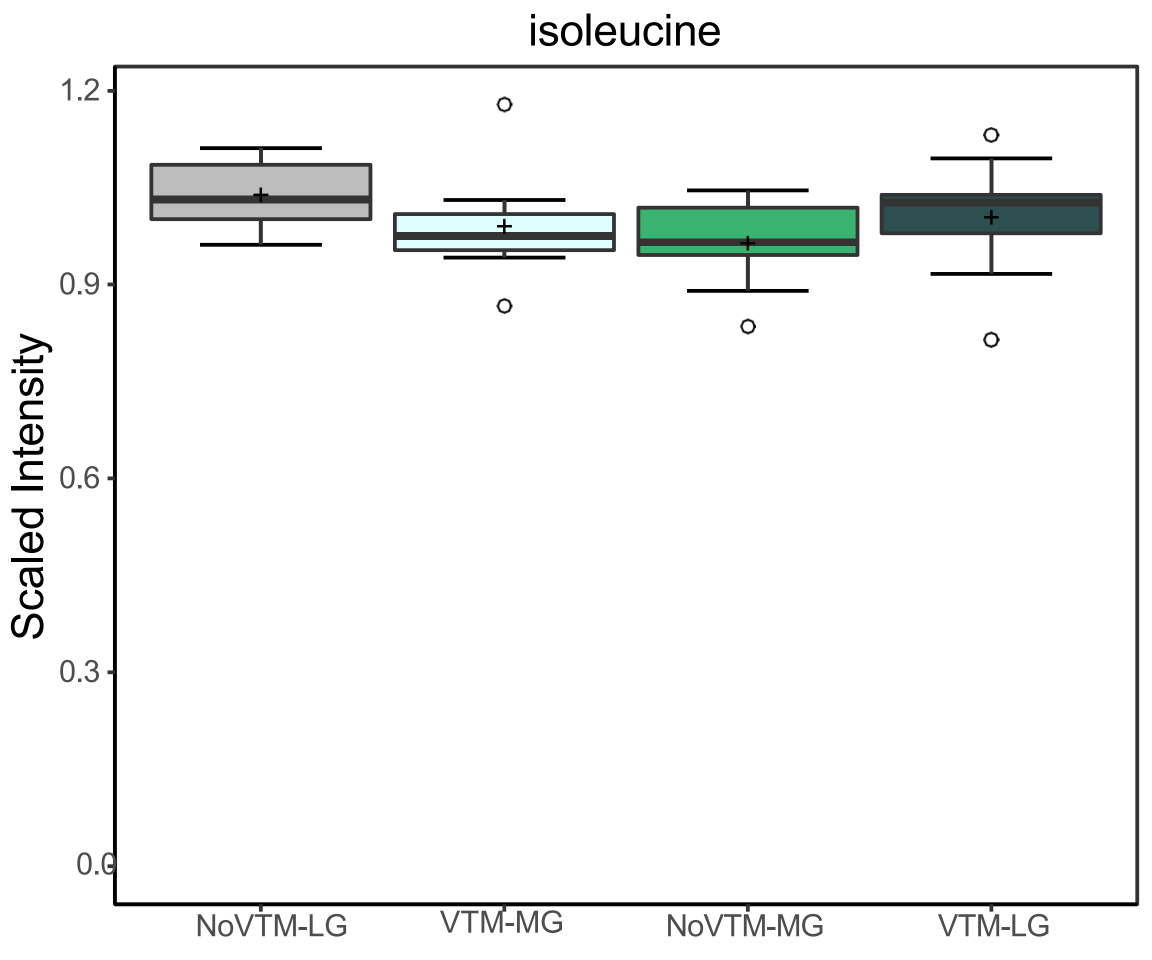
isoleucine
Scaled Intensity
1.2
0.9
0.6
0.3
0.0
NoVTM-LG
VTM-MG
NoVTM-MG
VTM-LG

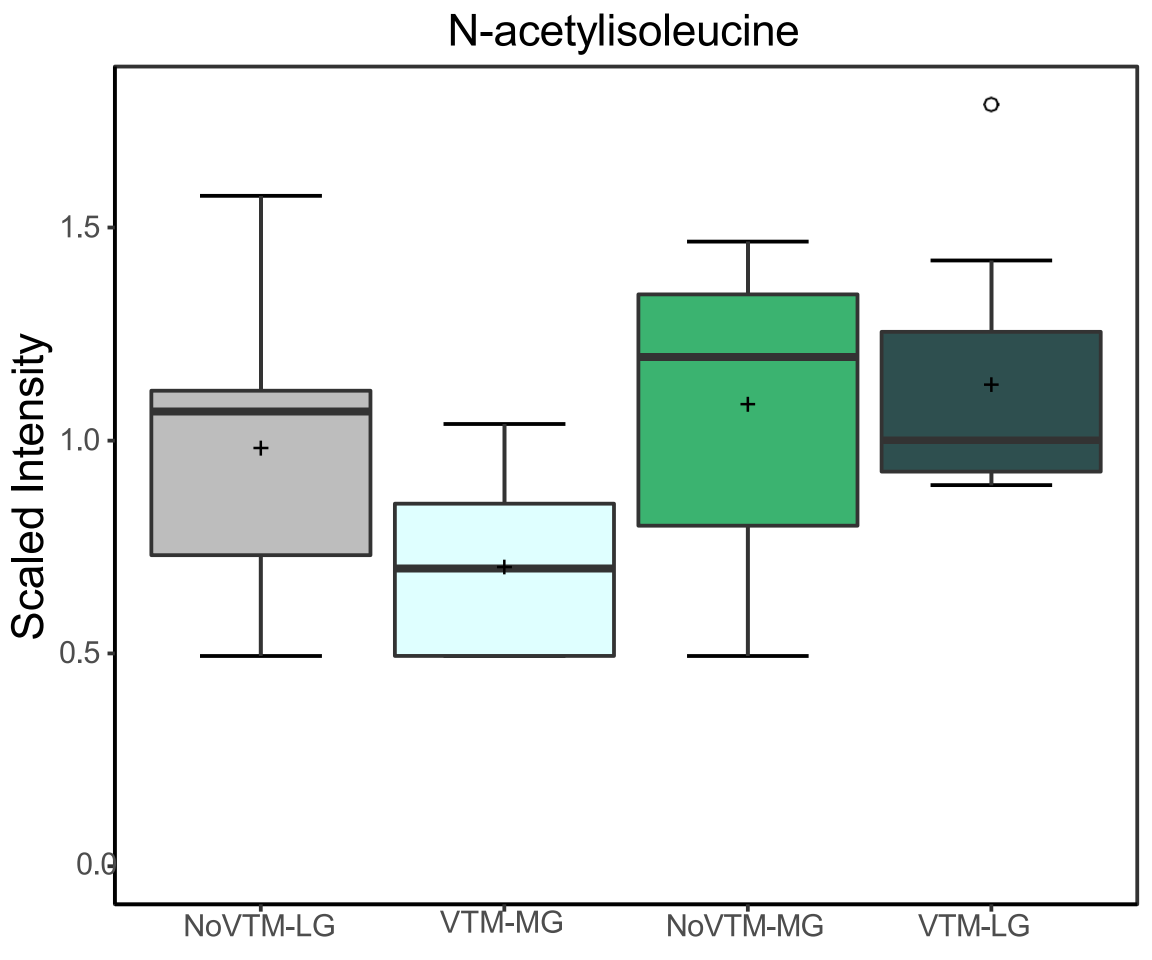

N-acetylisoleucine
Scaled Intensity
1.5
1.0
0.5
0.0
NoVTM-LG
VTM-MG
NoVTM-MG
VTM-LG

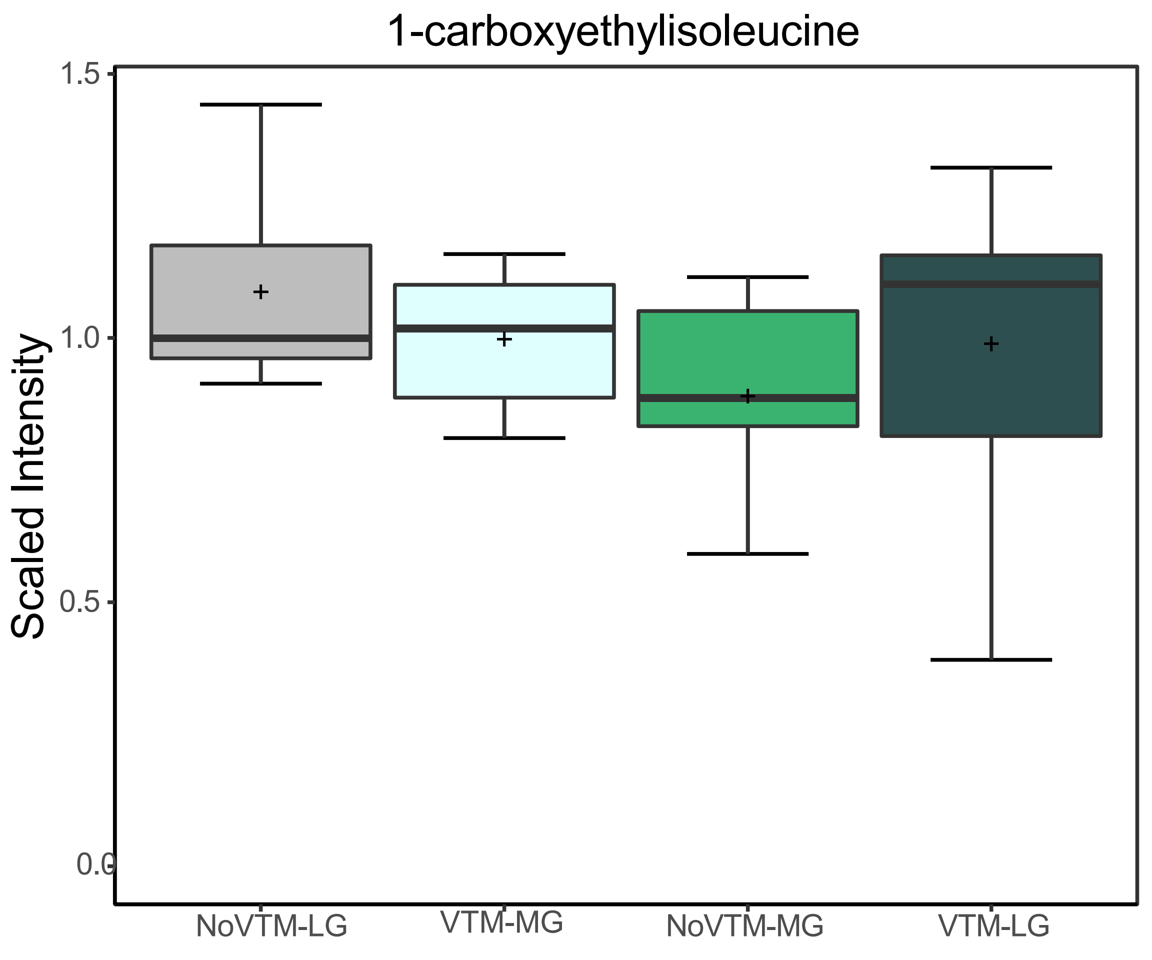
1-carboxyethylisoleucine
Scaled Intensity
1.5
1.0
0.5
0.0
NoVTM-LG
VTM-MG
NoVTM-MG
VTM-LG

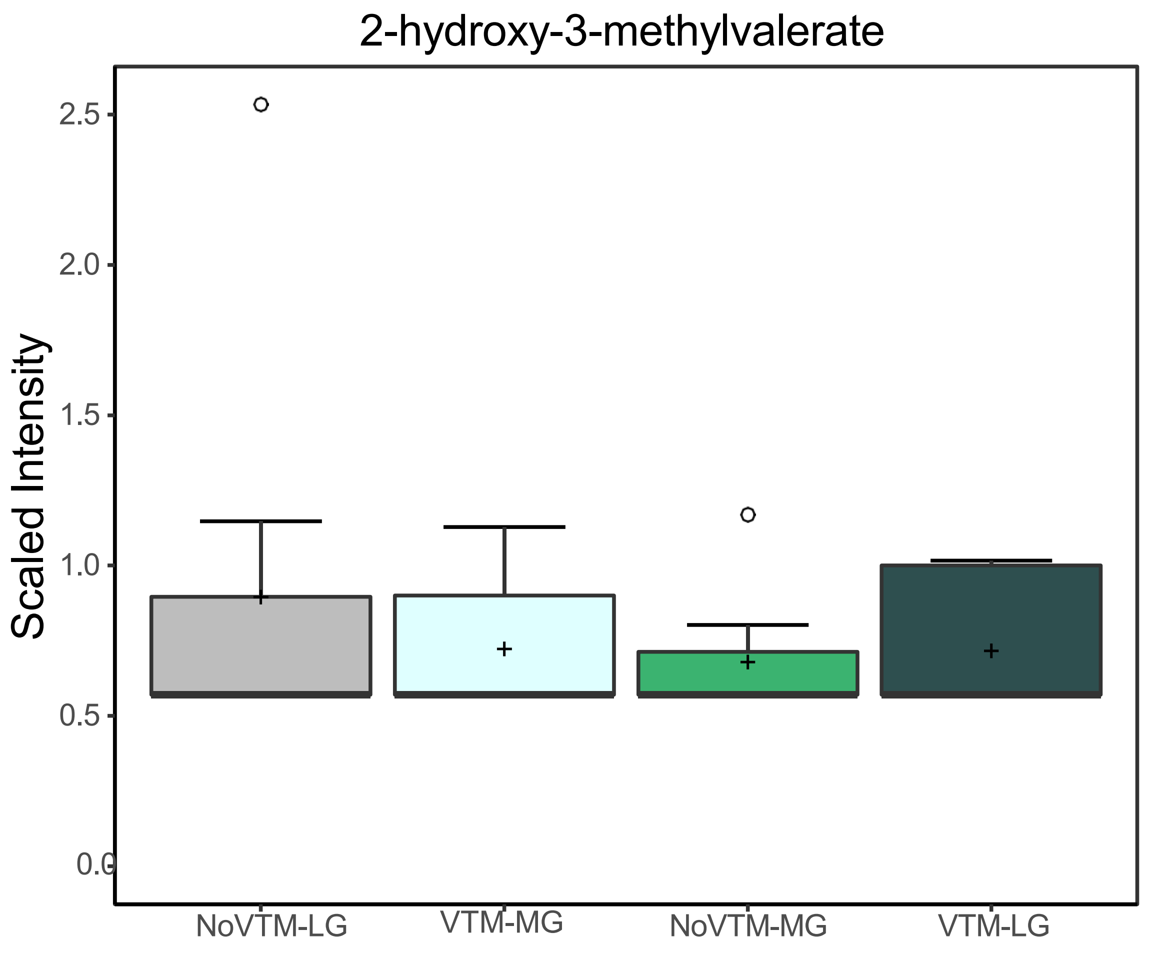
2-hydroxy-3-methylvalerate
Scaled Intensity
2.5
2.0
1.5
1.0
0.5
0.0
NoVTM-LG
VTM-MG
NoVTM-MG
VTM-LG

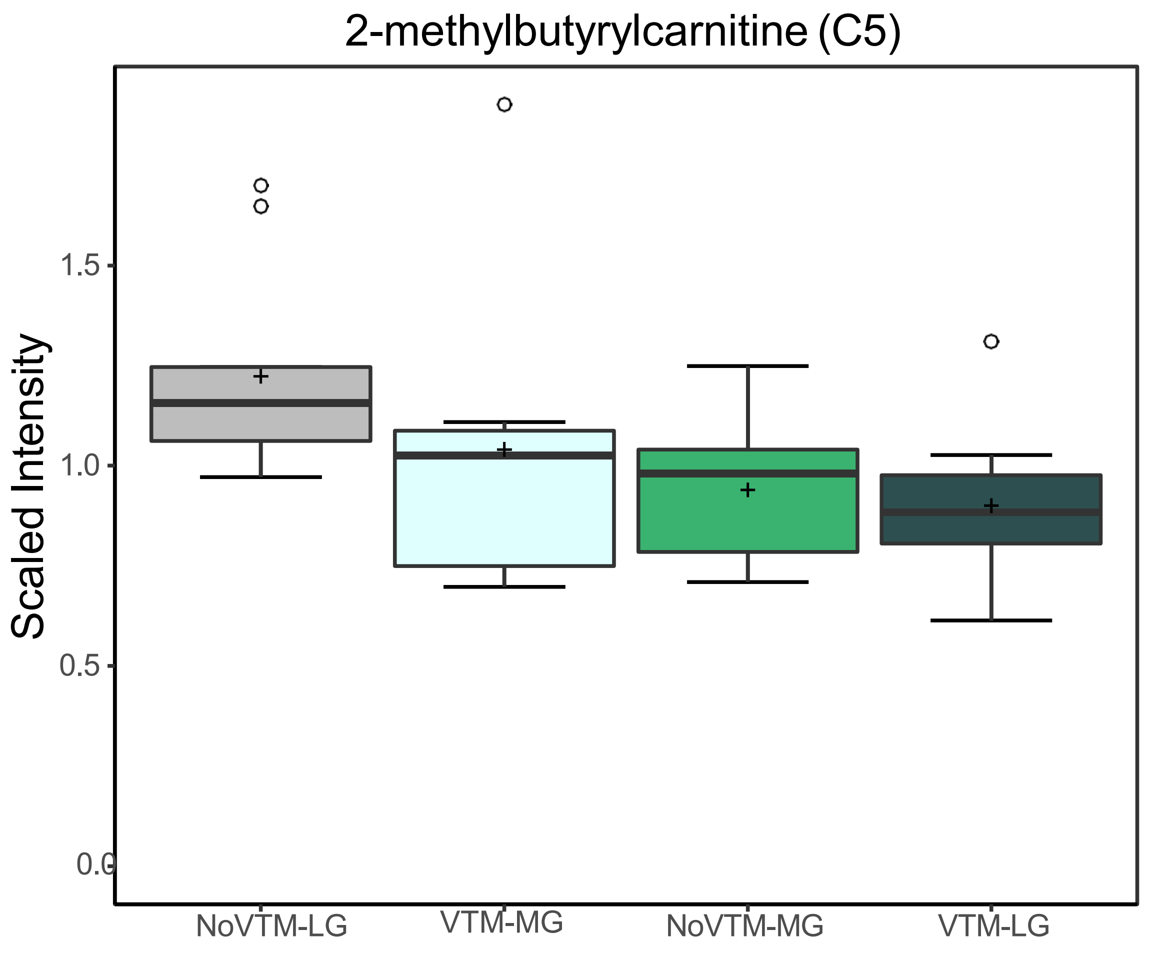
2-methylbutyrylcarnitine (C5)
Scaled Intensity
1.5
1.0
0.5
0.0
NoVTM-LG
VTM-MG
NoVTM-MG
VTM-LG

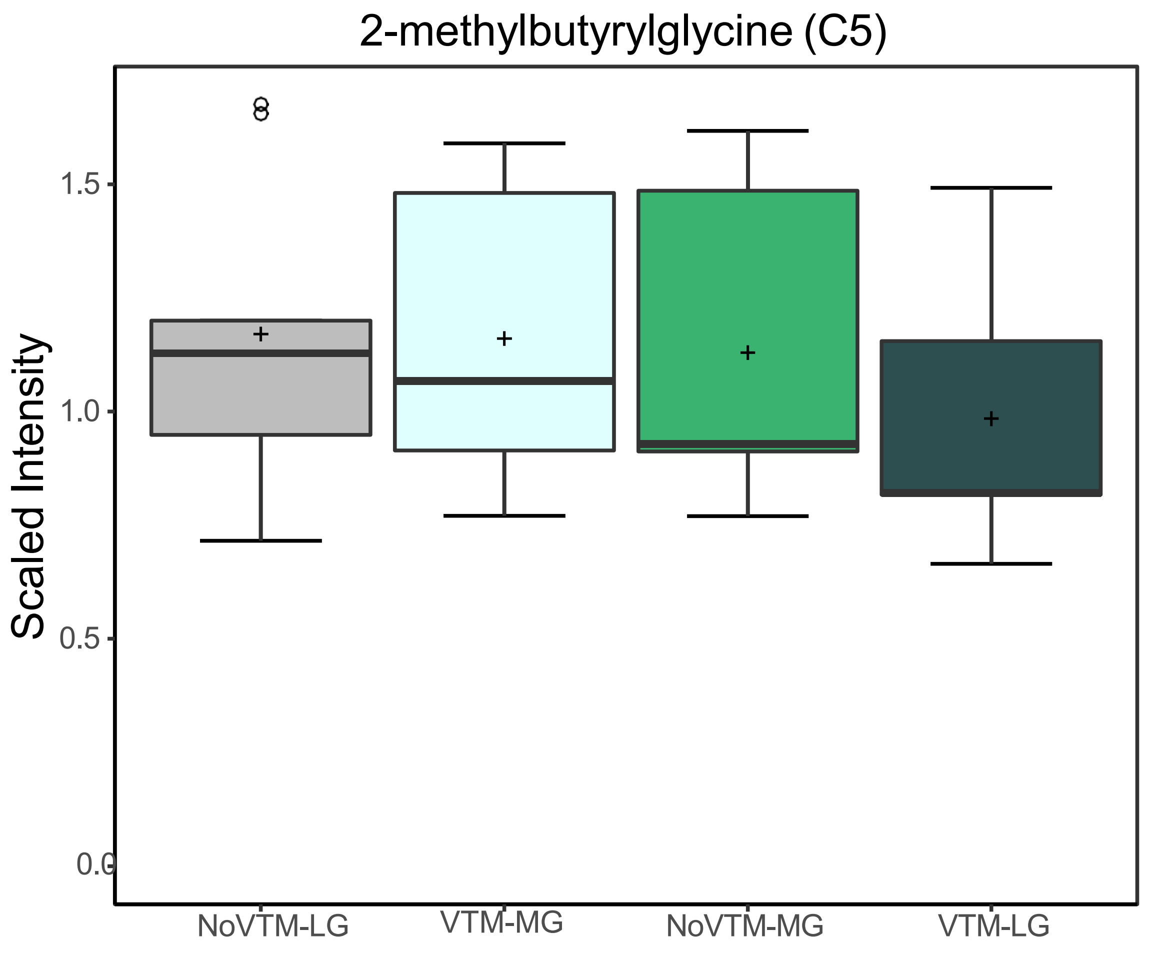
2-methylbutyrylglycine (C5)
Scaled Intensity
1.5
1.0
0.5
0.0
NoVTM-LG
VTM-MG
NoVTM-MG
VTM-LG

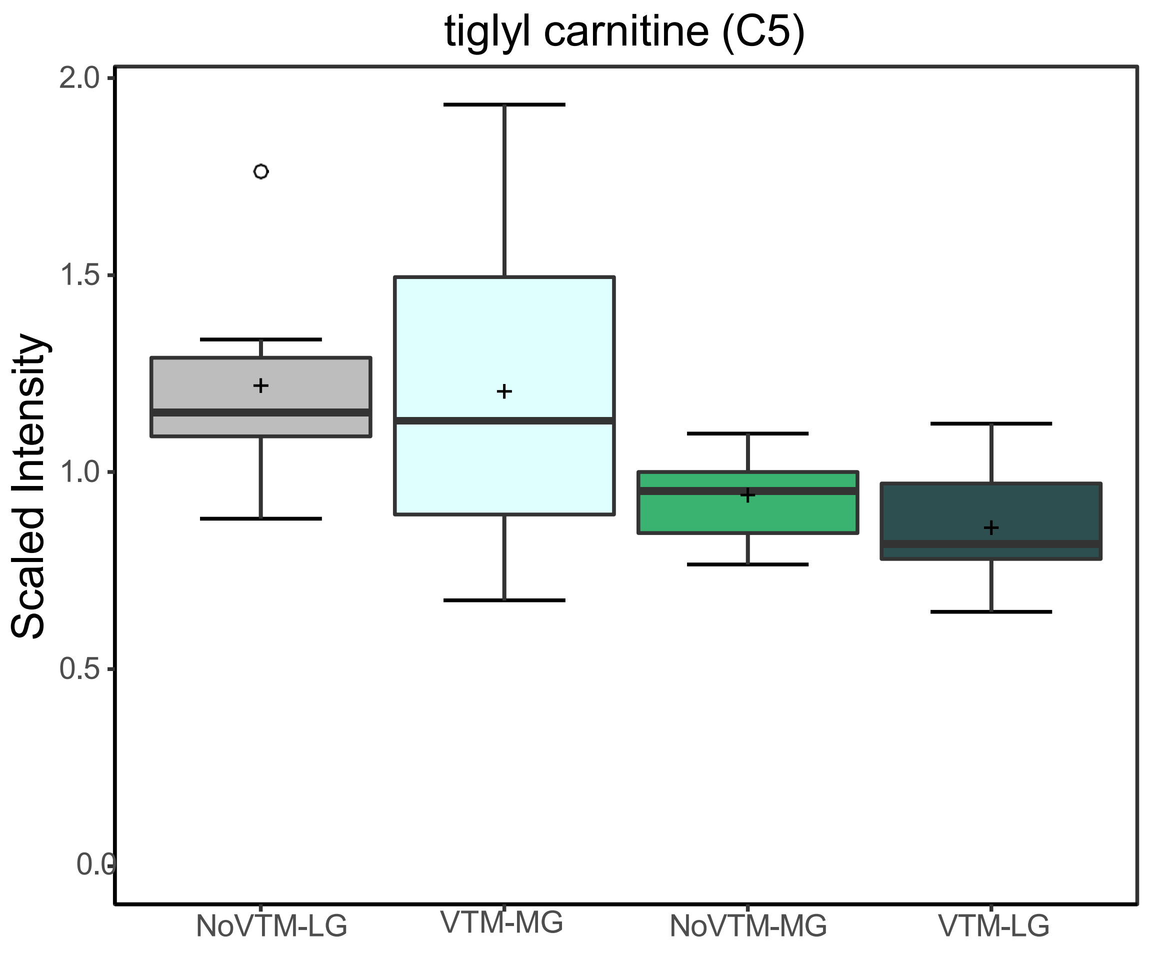

tiglyl carnitine (C5)
Scaled Intensity
2.0
1.5
1.0
0.5
0.0
NoVTM-LG
VTM-MG
NoVTM-MG
VTM-LG

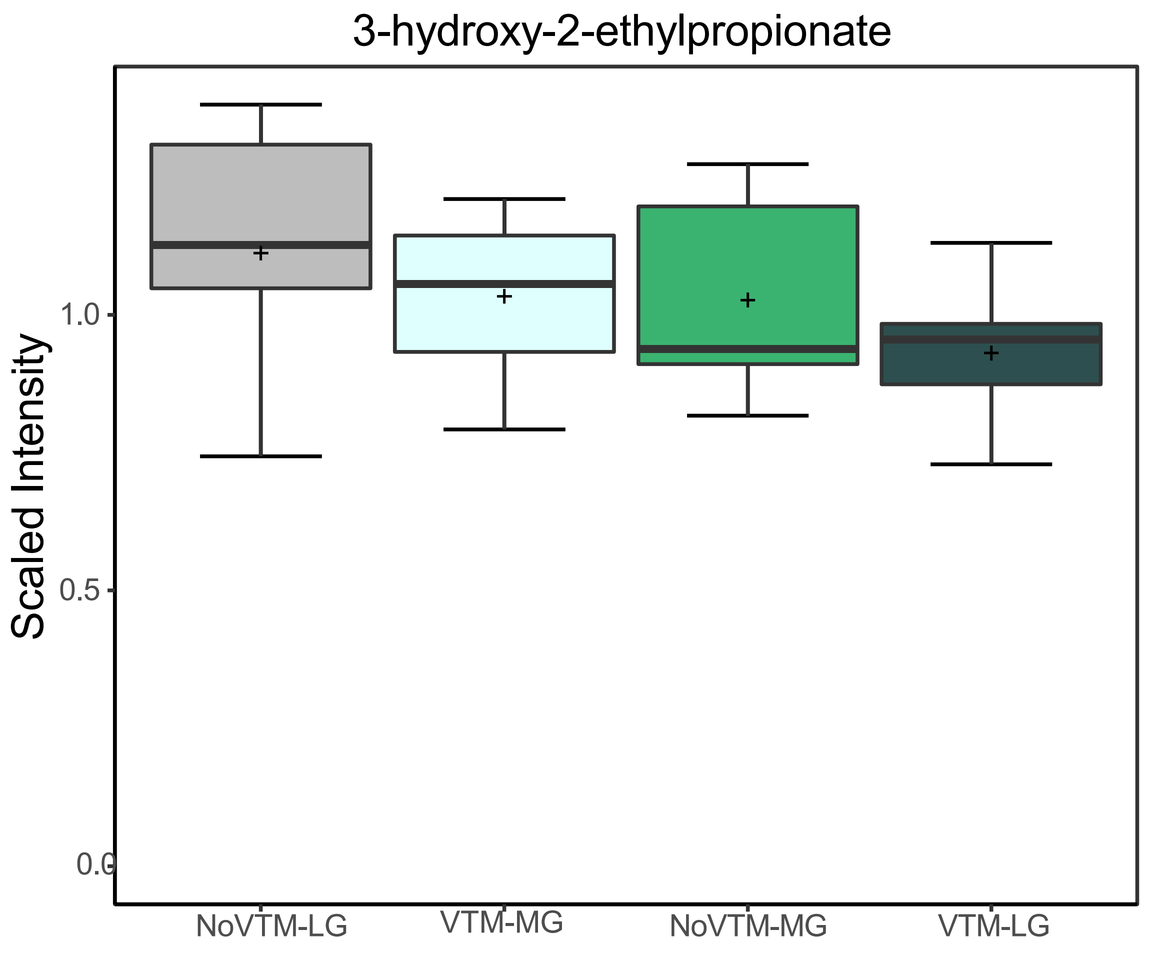
3-hydroxy-2-ethylpropionate
Scaled Intensity
1.0
0.5
0.0
NoVTM-LG
VTM-MG
NoVTM-MG
VTM-LG

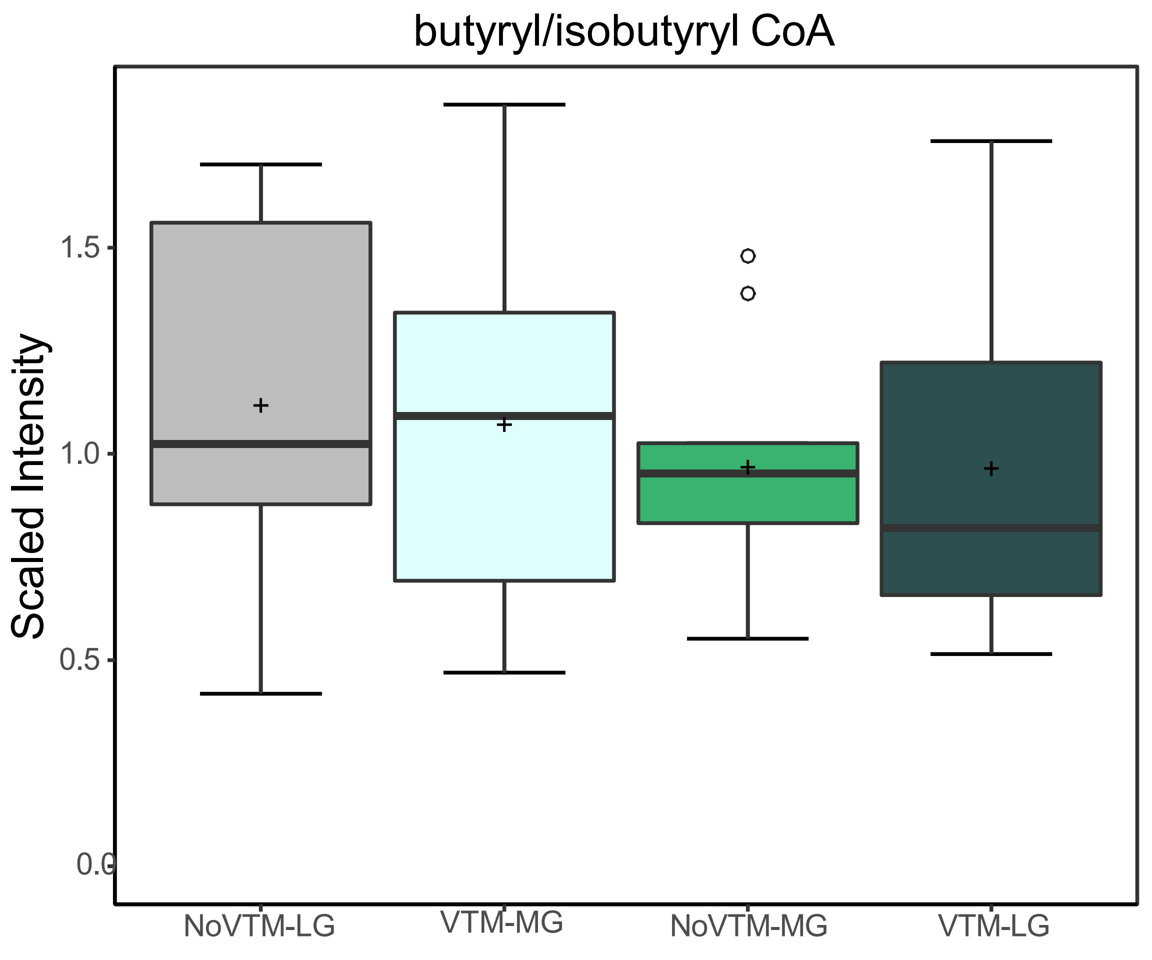
butyryl/isobutyryl CoA
Scaled Intensity
1.5
1.0
0.5
0.0
NoVTM-LG
VTM-MG
NoVTM-MG
VTM-LG

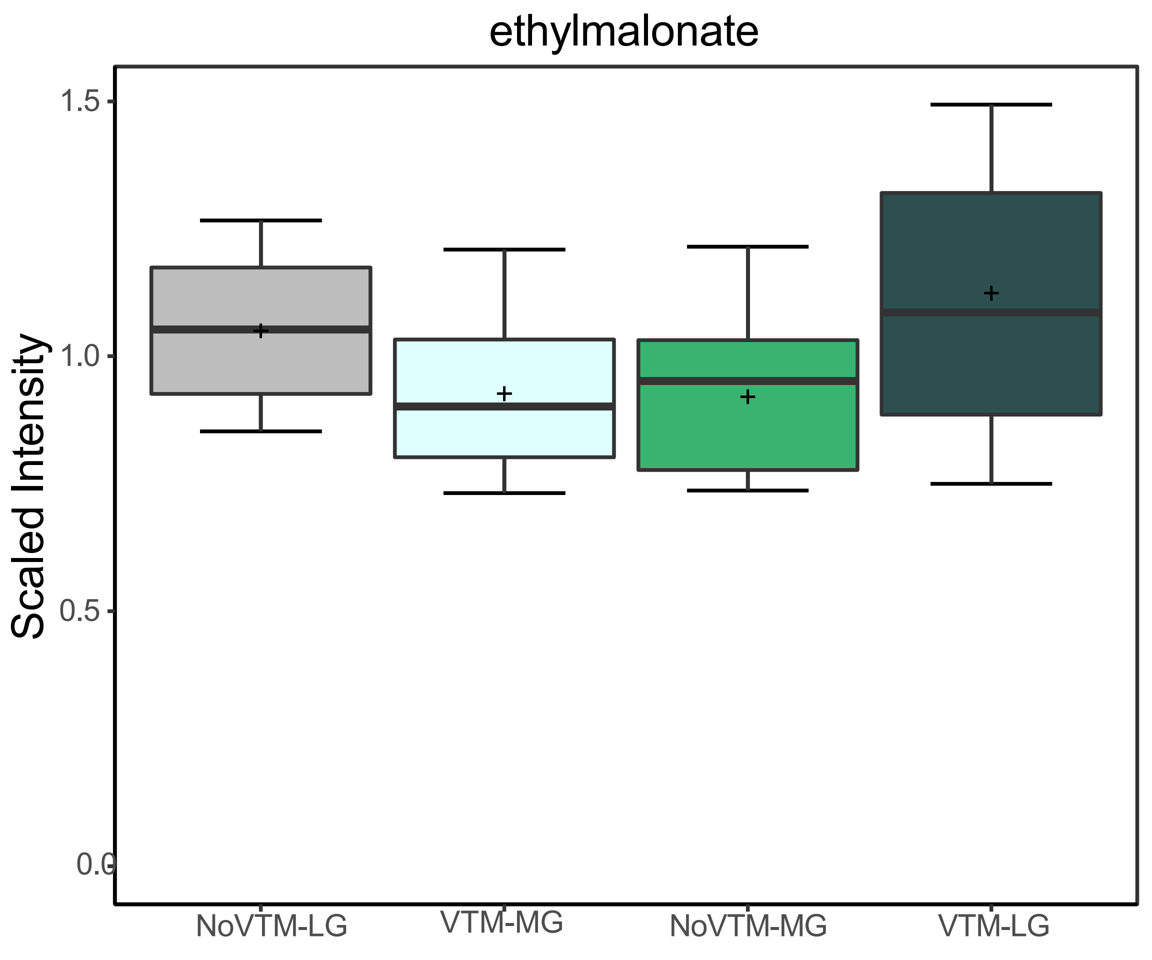
ethylmalonate
Scaled Intensity
1.5
1.0
0.5
0.0
NoVTM-LG
VTM-MG
NoVTM-MG
VTM-LG

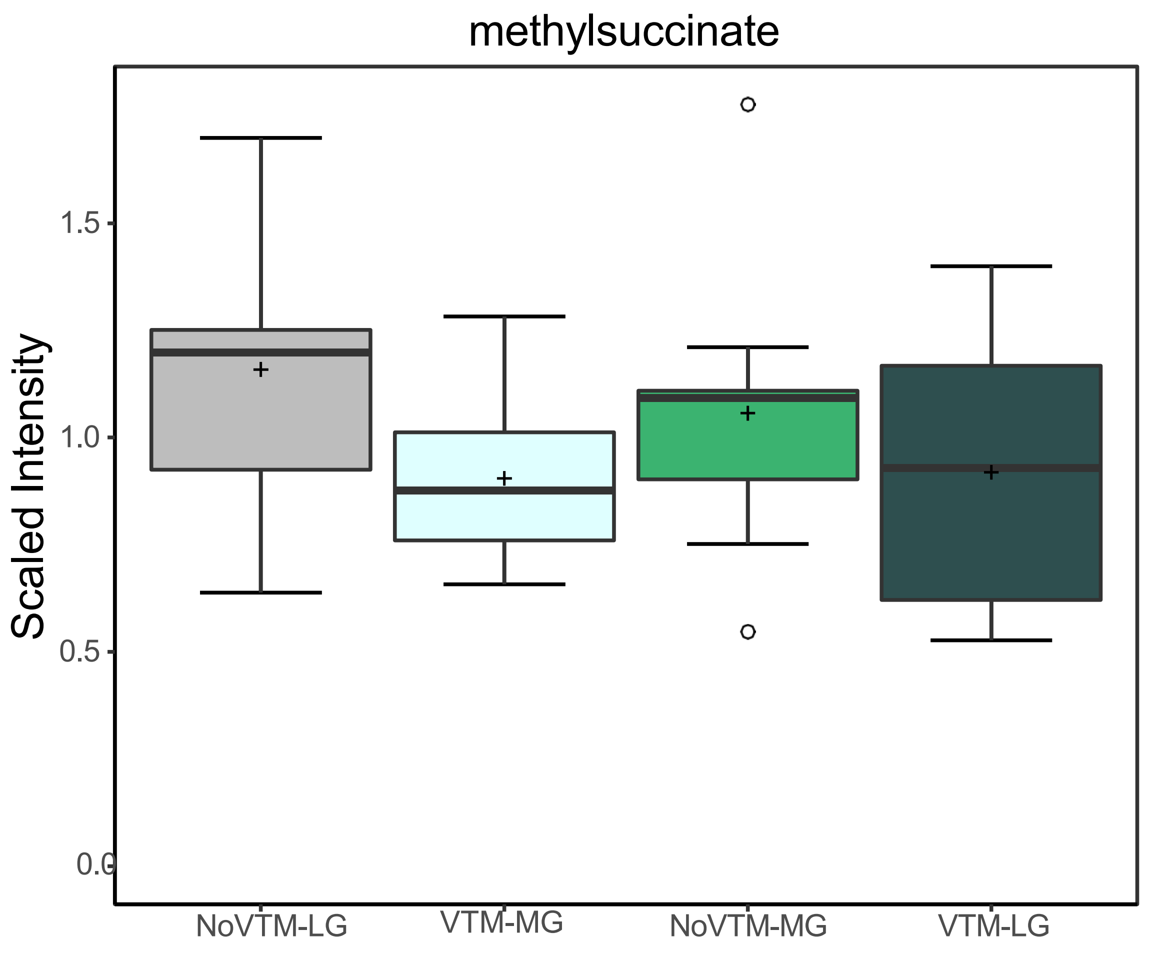

methylsuccinate
Scaled Intensity
1.5
1.0
0.5
0.0
NoVTM-LG
VTM-MG
NoVTM-MG
VTM-LG

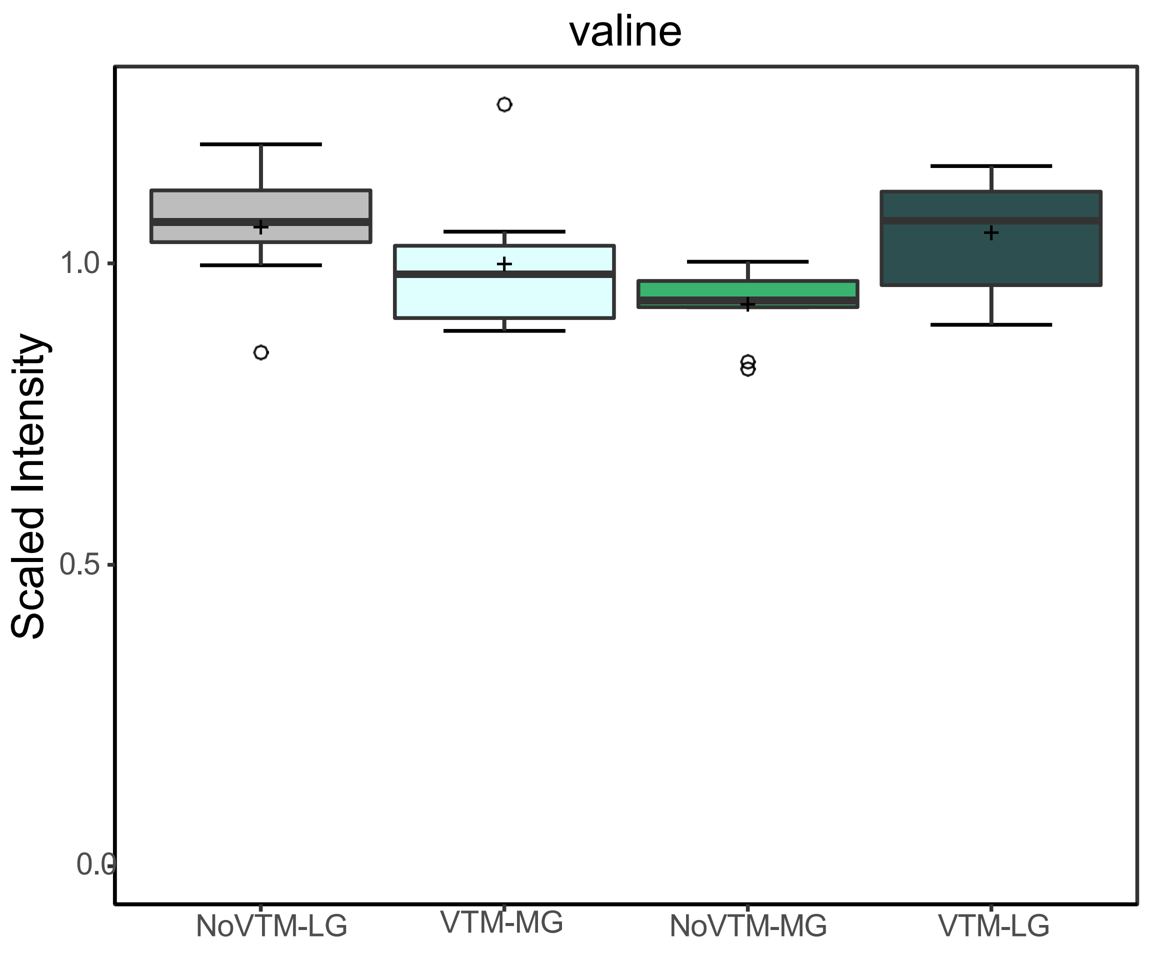
valine
Scaled Intensity
1.0
0.5
0.0
NoVTM-LG
VTM-MG
NoVTM-MG
VTM-LG

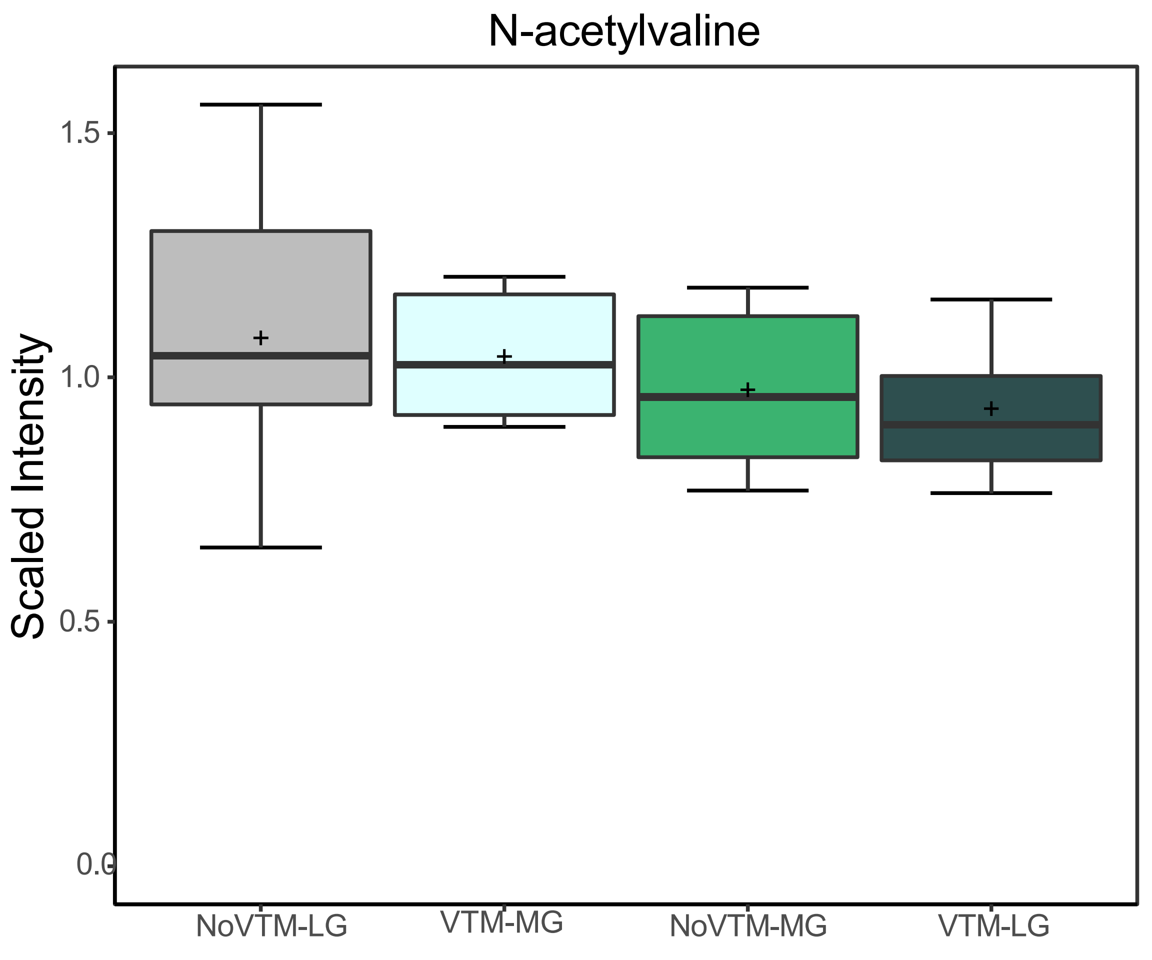
N-acetylvaline
Scaled Intensity
1.5
1.0
0.5
0.0
NoVTM-LG
VTM-MG
NoVTM-MG
VTM-LG

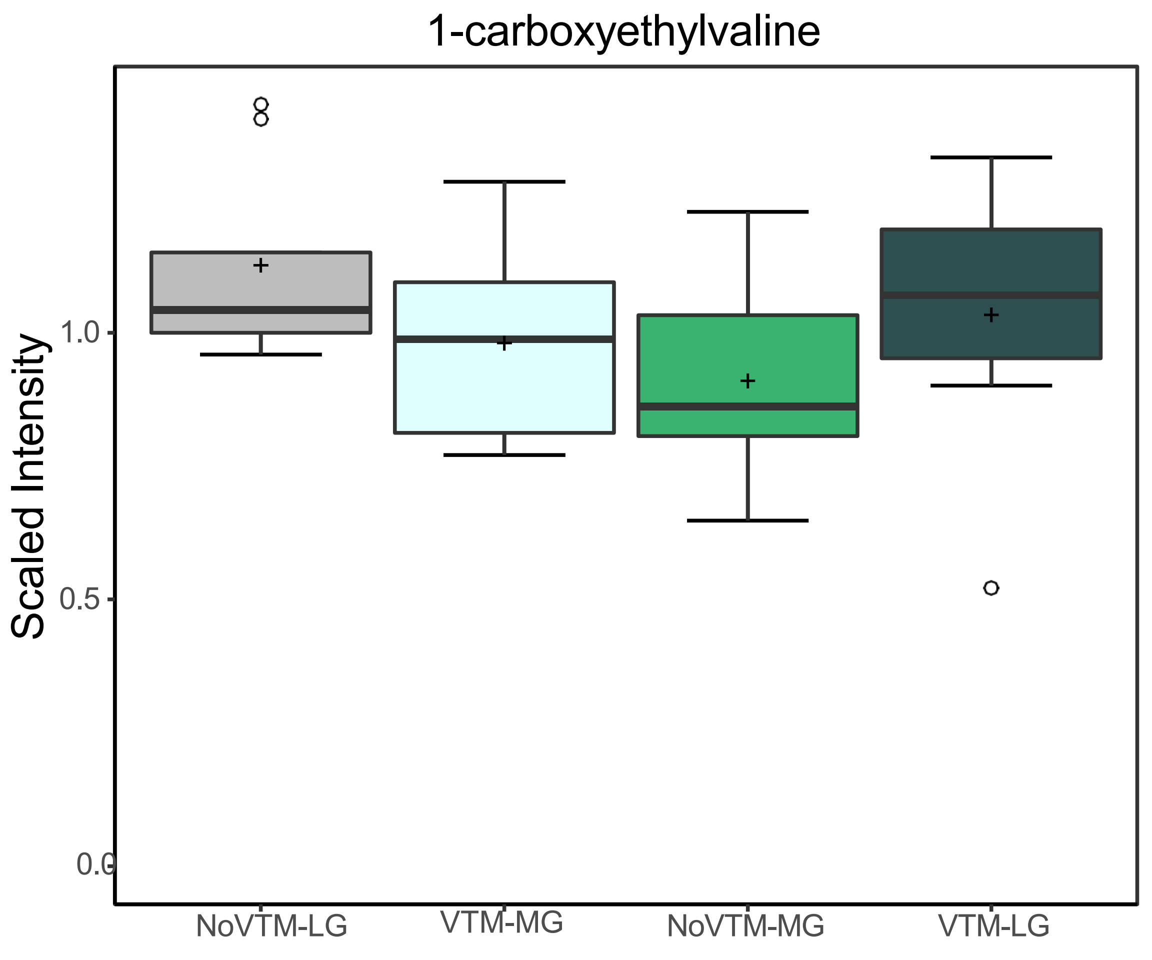
1-carboxyethylvaline
Scaled Intensity
1.0
0.5
0.0
NoVTM-LG
VTM-MG
NoVTM-MG
VTM-LG

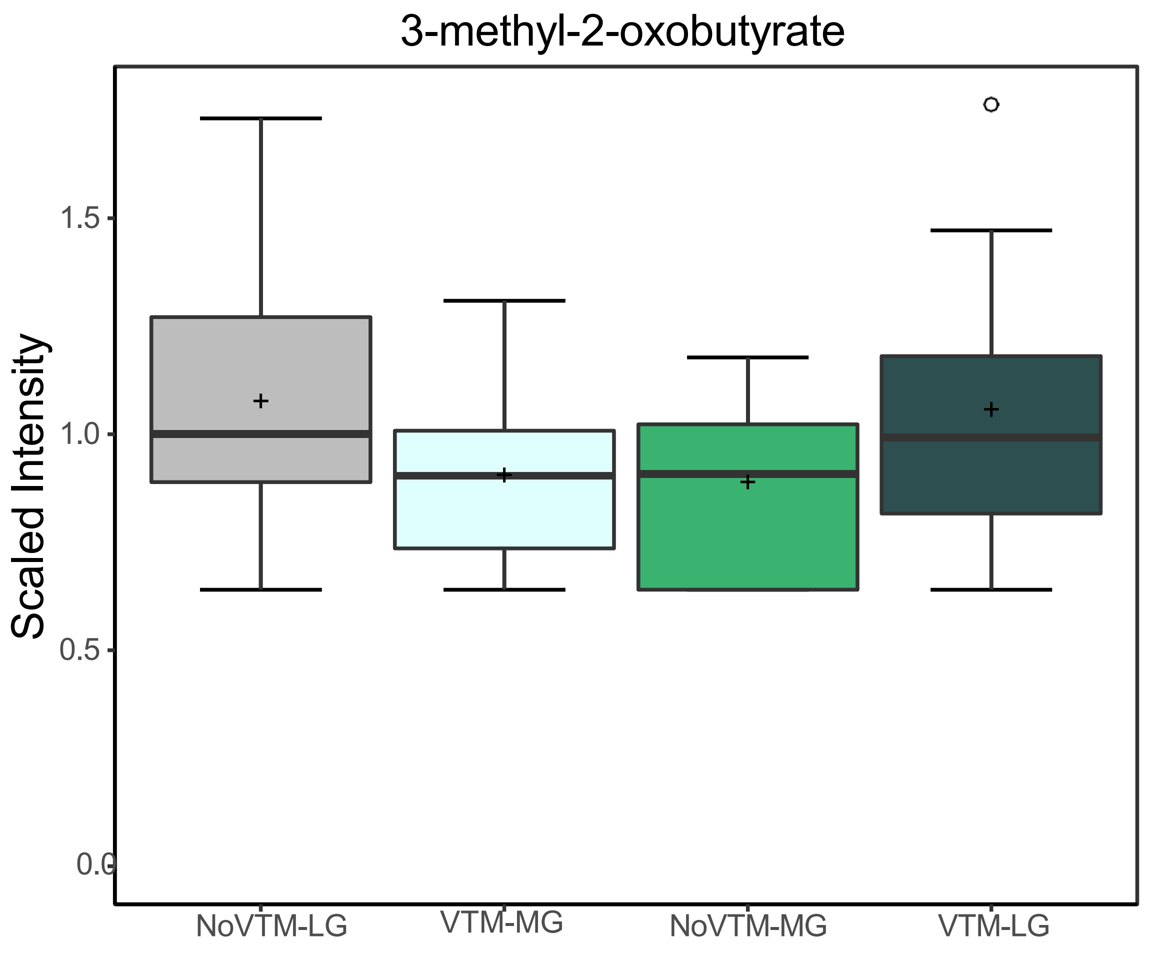

3-methyl-2-oxobutyrate
Scaled Intensity
1.5
1.0
0.5
0.0
NoVTM-LG
VTM-MG
NoVTM-MG
VTM-LG

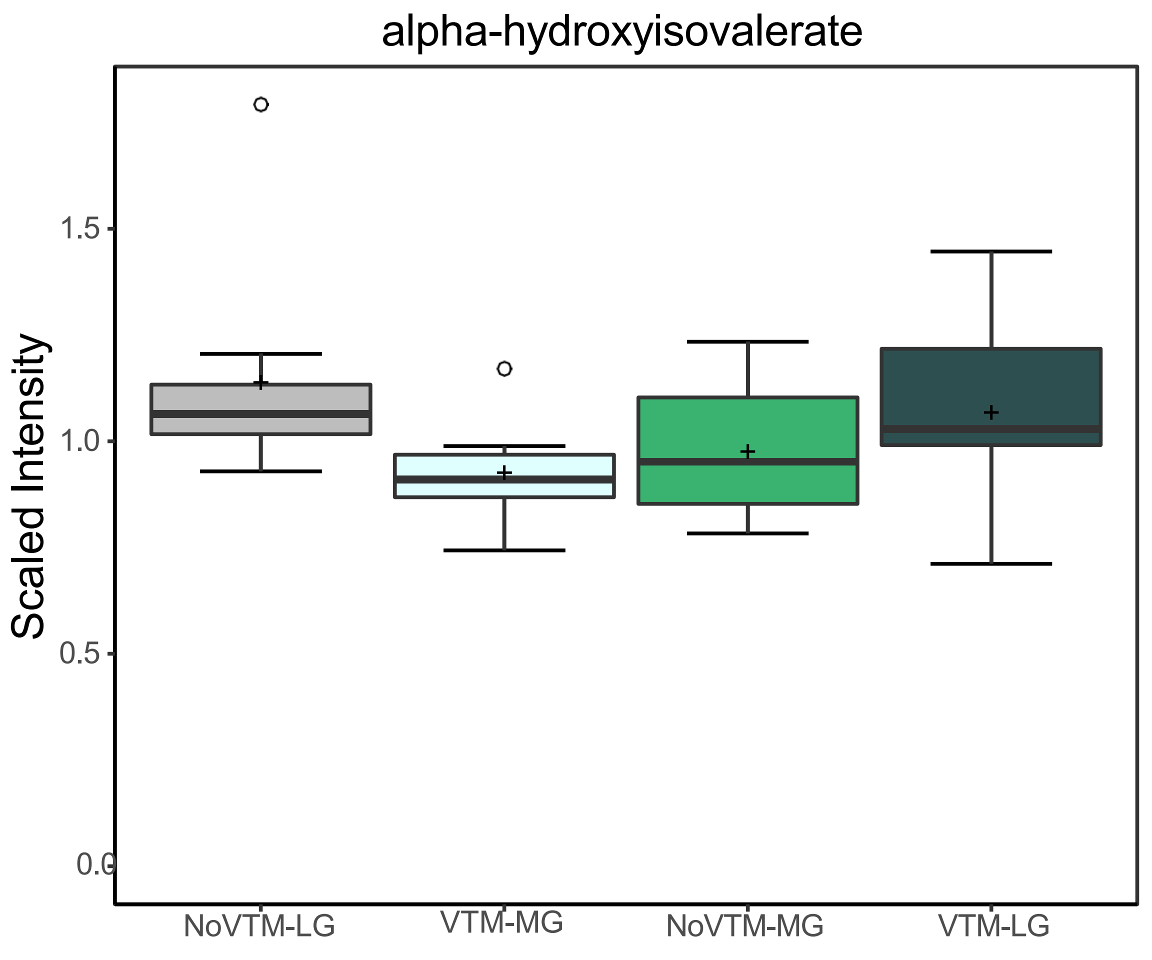
alpha-hydroxyisovalerate
Scaled Intensity
1.5
1.0
0.5
0.0
NoVTM-LG
VTM-MG
NoVTM-MG
VTM-LG

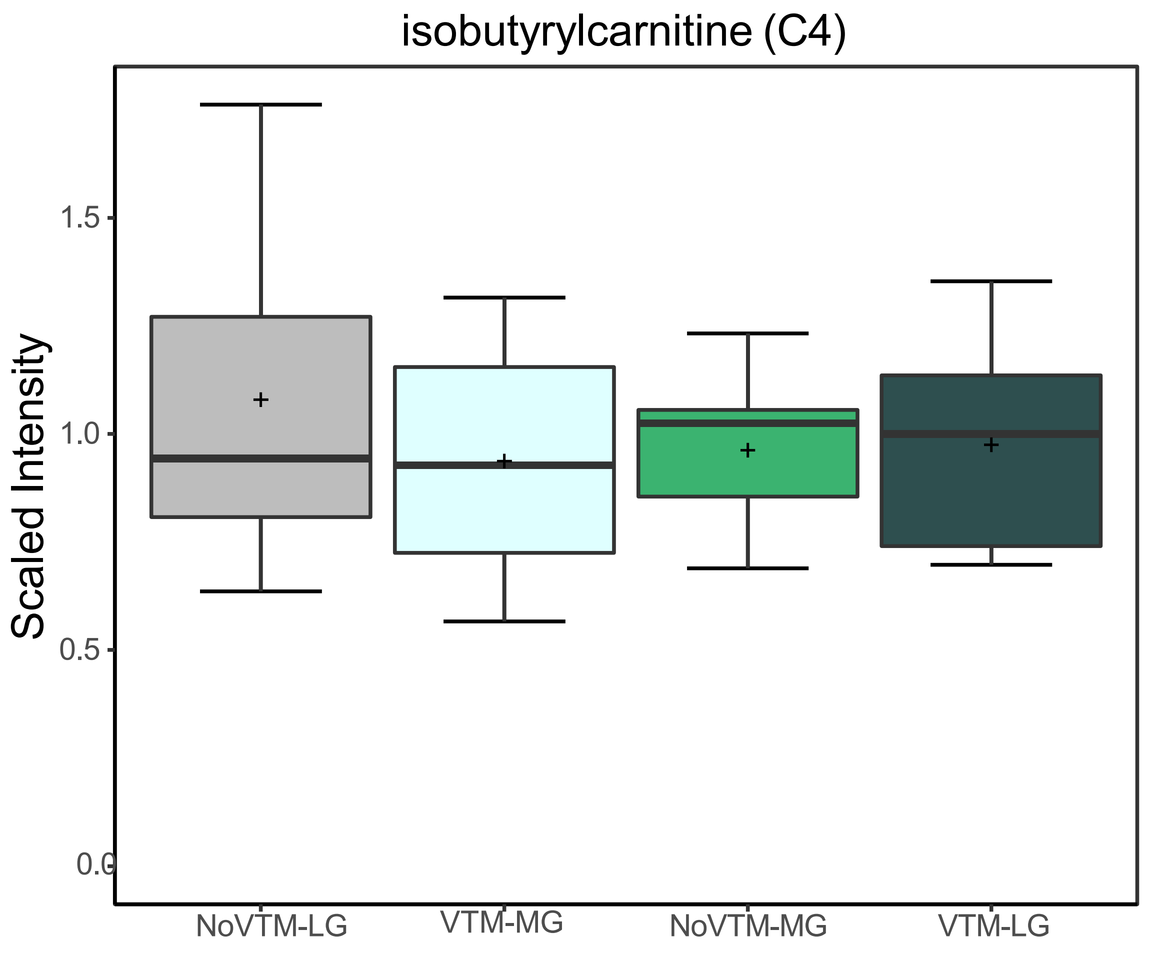
isobutyrylcarnitine (C4)
Scaled Intensity
1.5
1.0
0.5
0.0
NoVTM-LG
VTM-MG
NoVTM-MG
VTM-LG

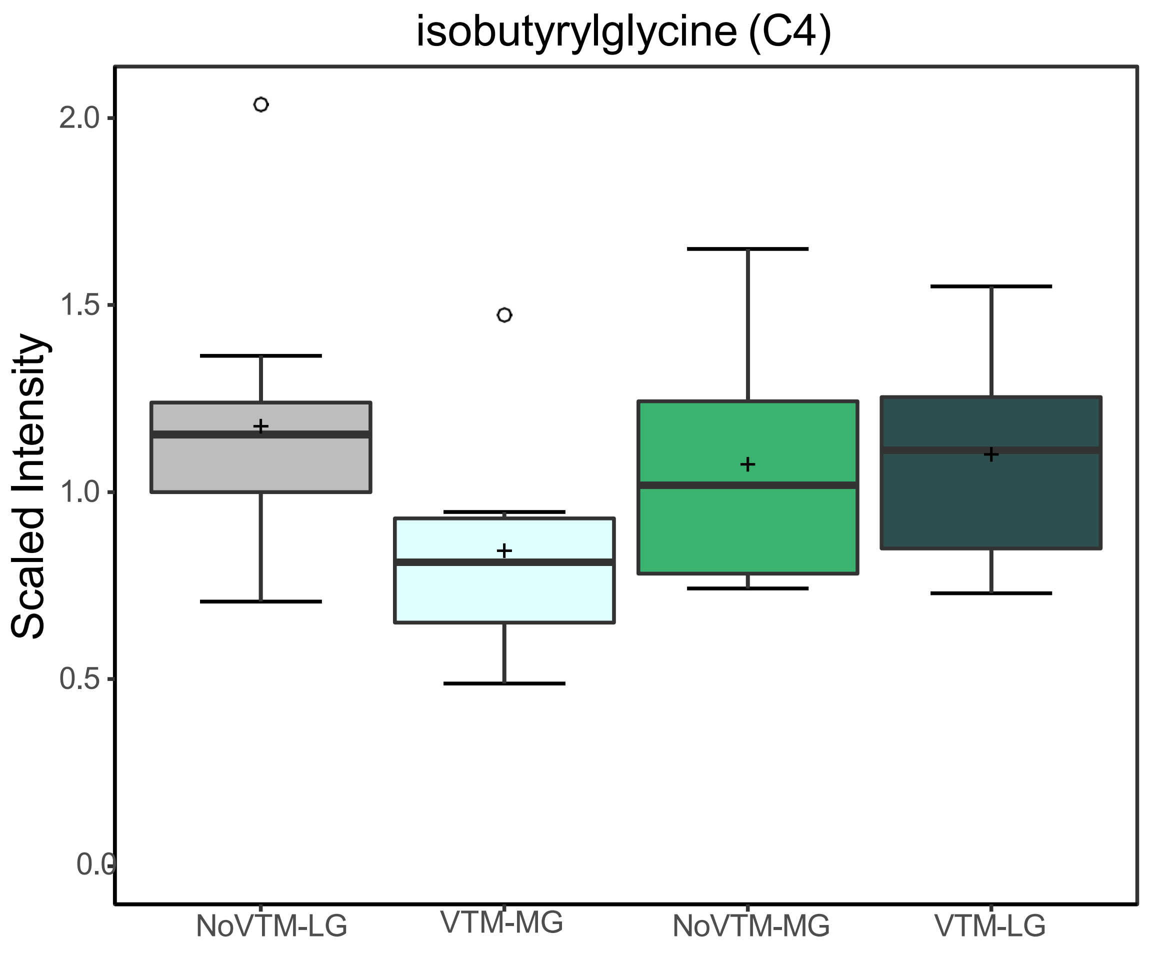

isobutyrylglycine (C4)
Scaled Intensity
2.0
1.5
1.0
0.5
0.0
NoVTM-LG
VTM-MG
NoVTM-MG
VTM-LG

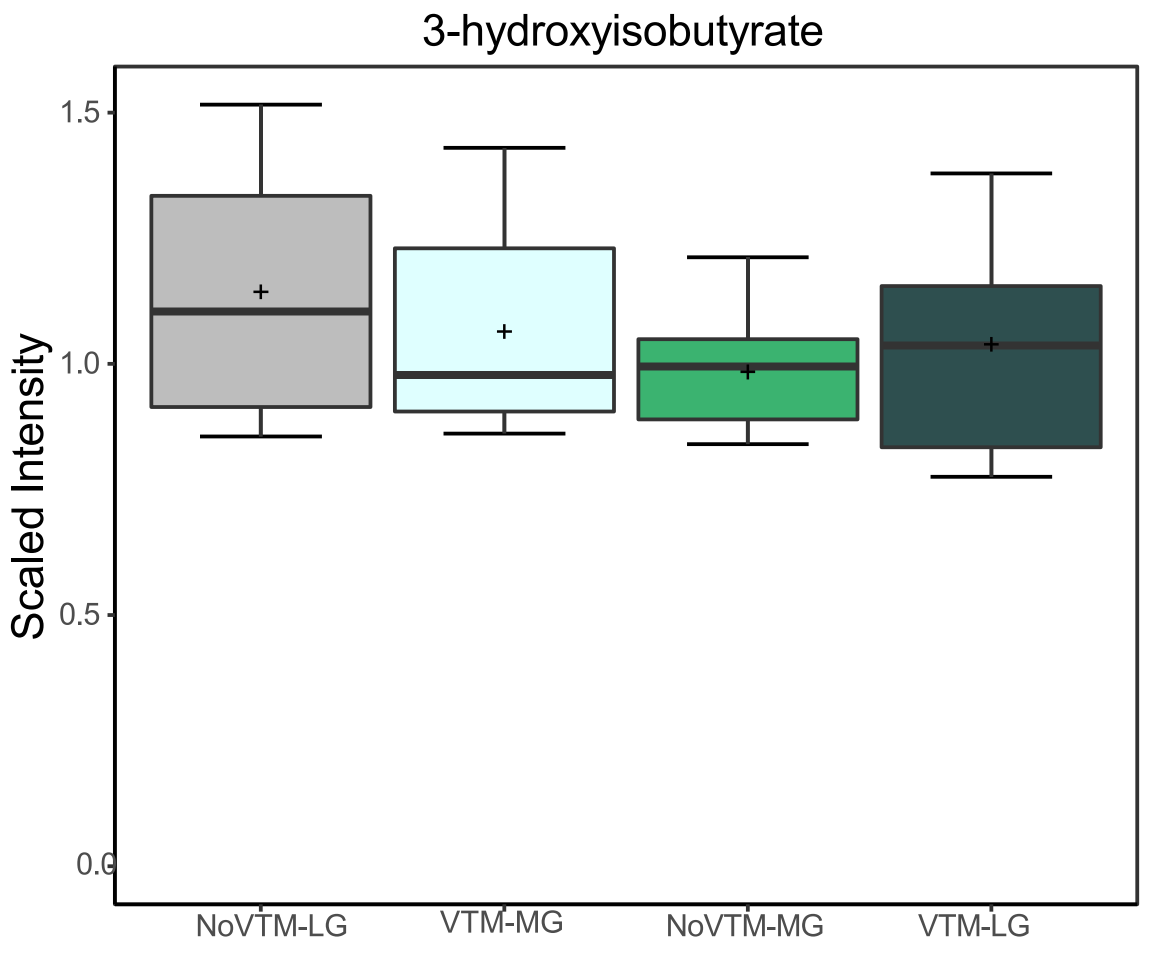
3-hydroxyisobutyrate
Scaled Intensity
1.5
1.0
0.5
0.0
NoVTM-LG
VTM-MG
NoVTM-MG
VTM-LG

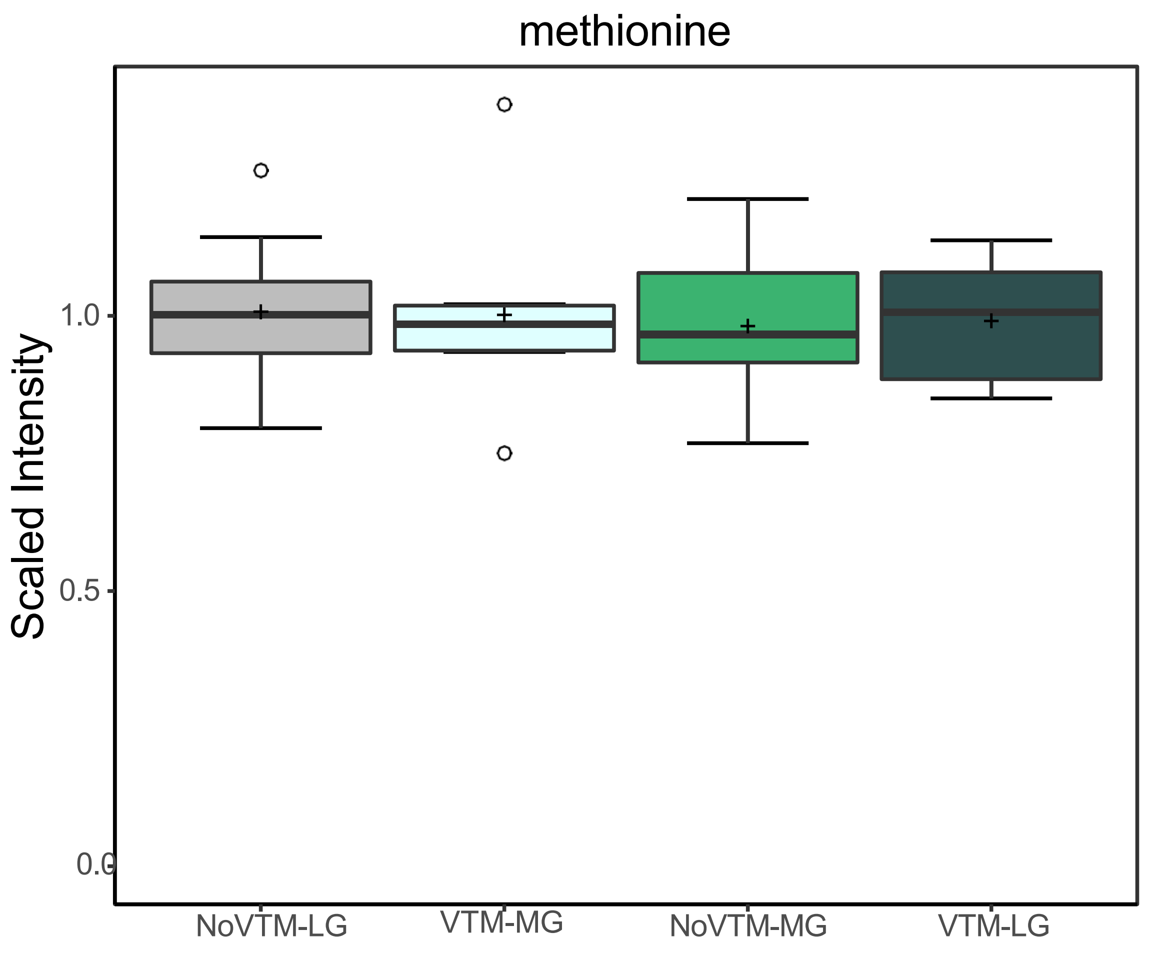

methionine
Scaled Intensity
1.0
0.5
0.0
NoVTM-LG
VTM-MG
NoVTM-MG
VTM-LG

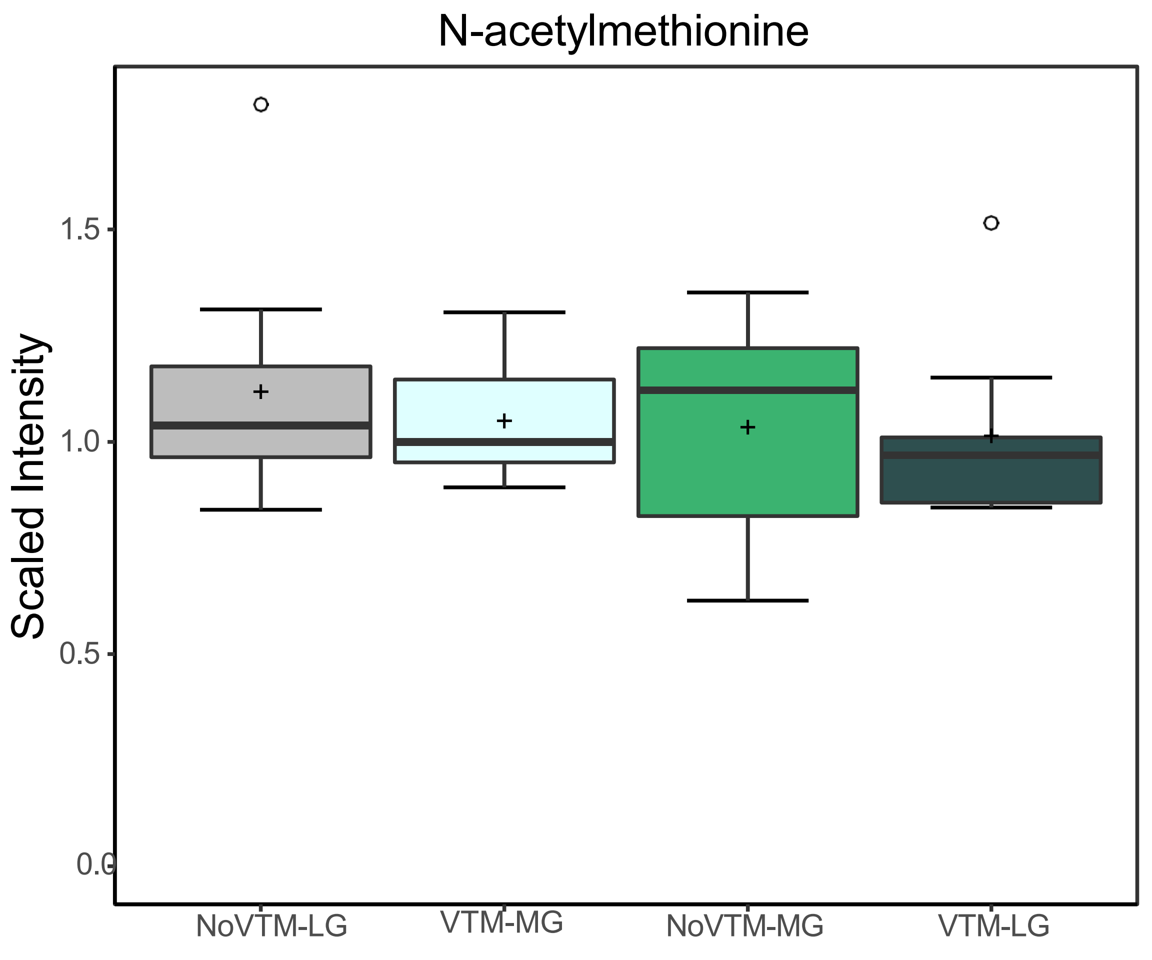

N-acetylmethionine
Scaled Intensity
1.5
1.0
0.5
0.0
NoVTM-LG
VTM-MG
NoVTM-MG
VTM-LG

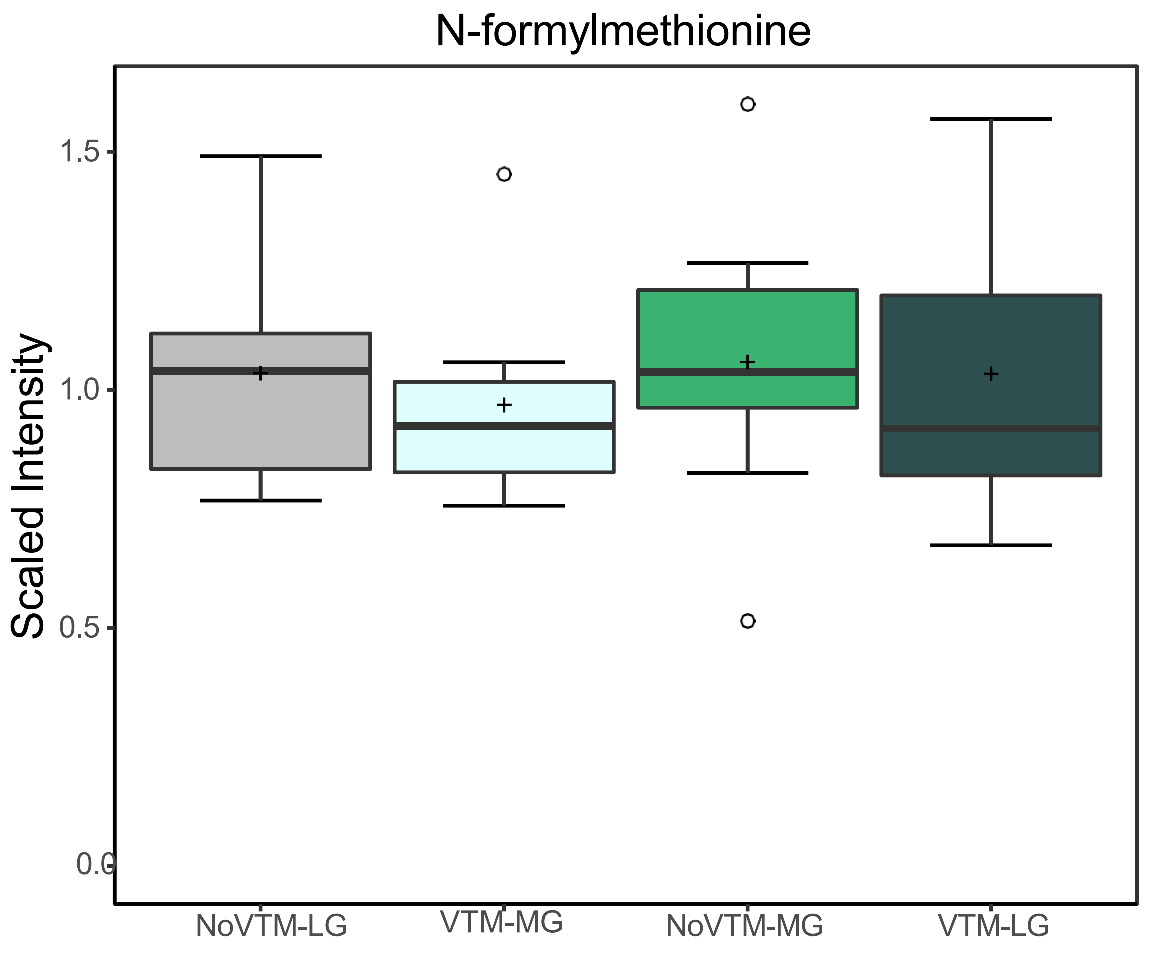
N-formylmethionine
Scaled Intensity
1.5
1.0
0.5
0.0
NoVTM-LG
VTM-MG
NoVTM-MG
VTM-LG

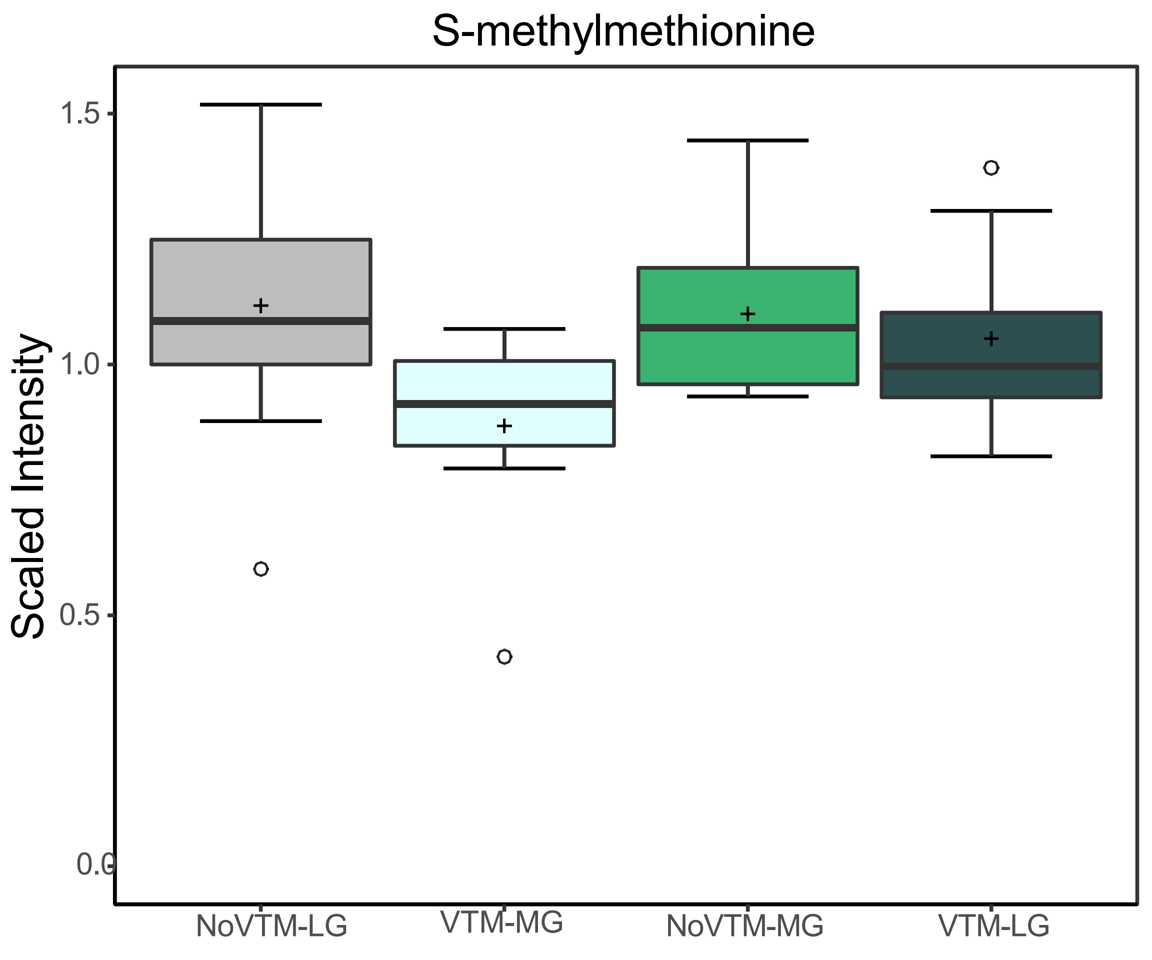

S-methylmethionine
Scaled Intensity
1.5
1.0
0.5
0.0
NoVTM-LG
VTM-MG
NoVTM-MG
VTM-LG

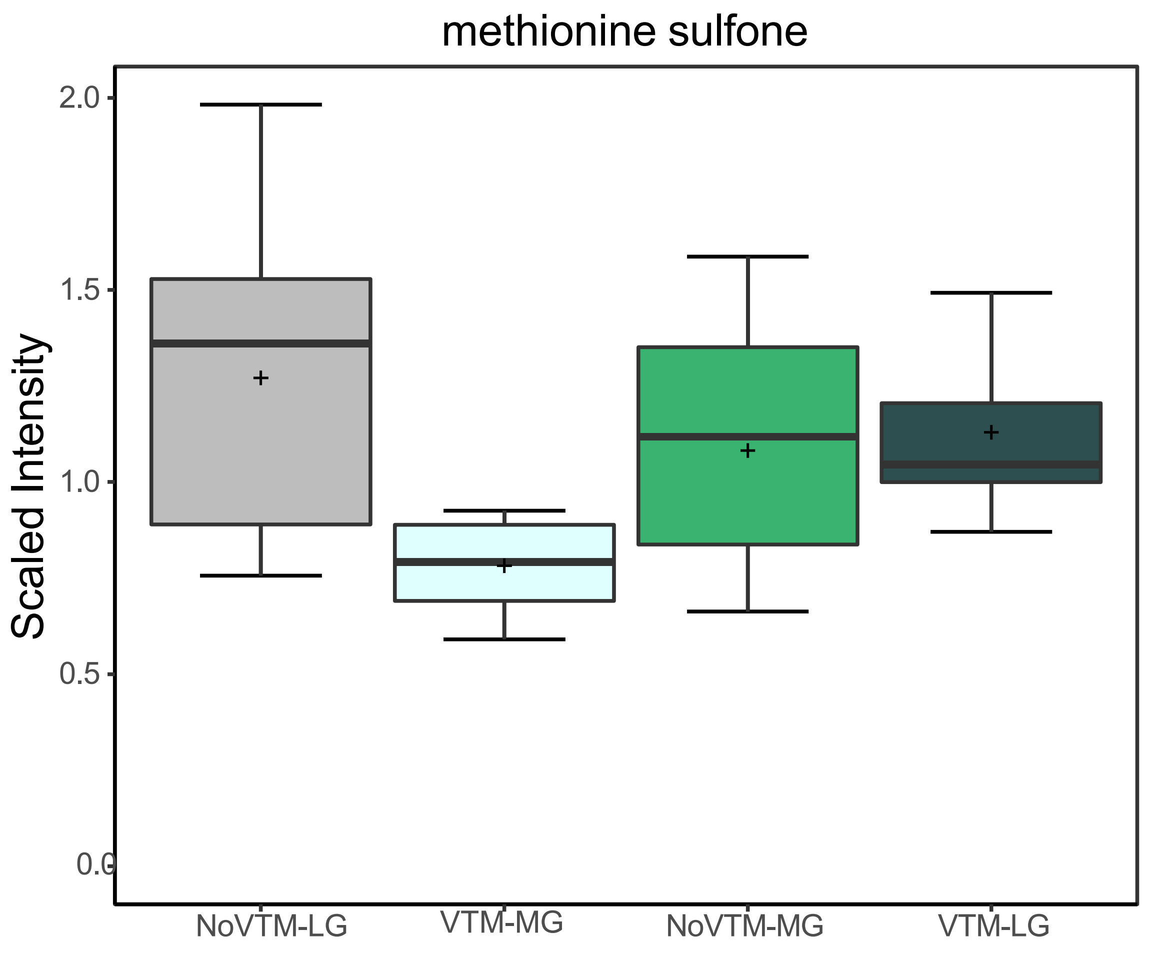

methionine sulfone
Scaled Intensity
2.0
1.5
1.0
0.5
0.0
NoVTM-LG
VTM-MG
NoVTM-MG
VTM-LG

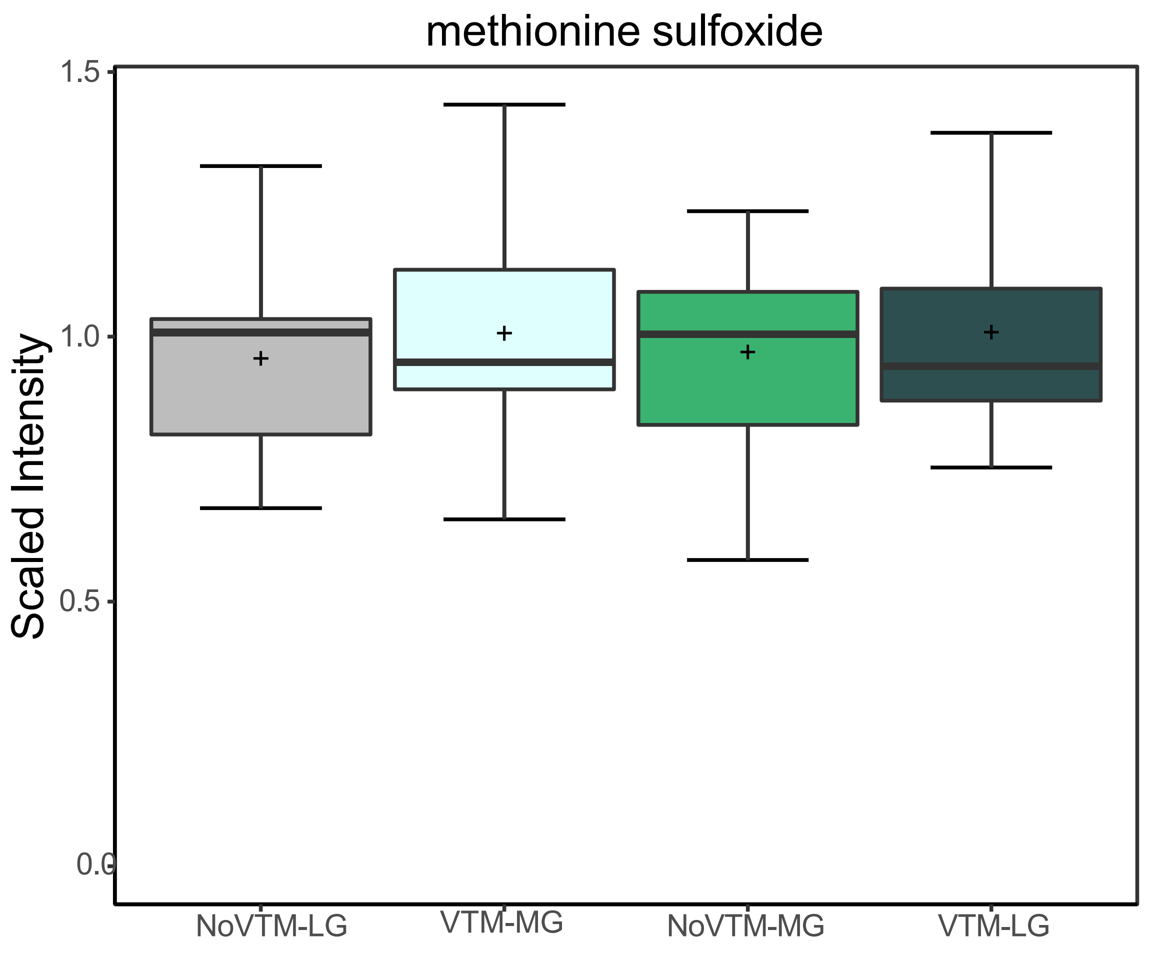
methionine sulfoxide
Scaled Intensity
1.5
1.0
0.5
0.0
NoVTM-LG
VTM-MG
NoVTM-MG
VTM-LG

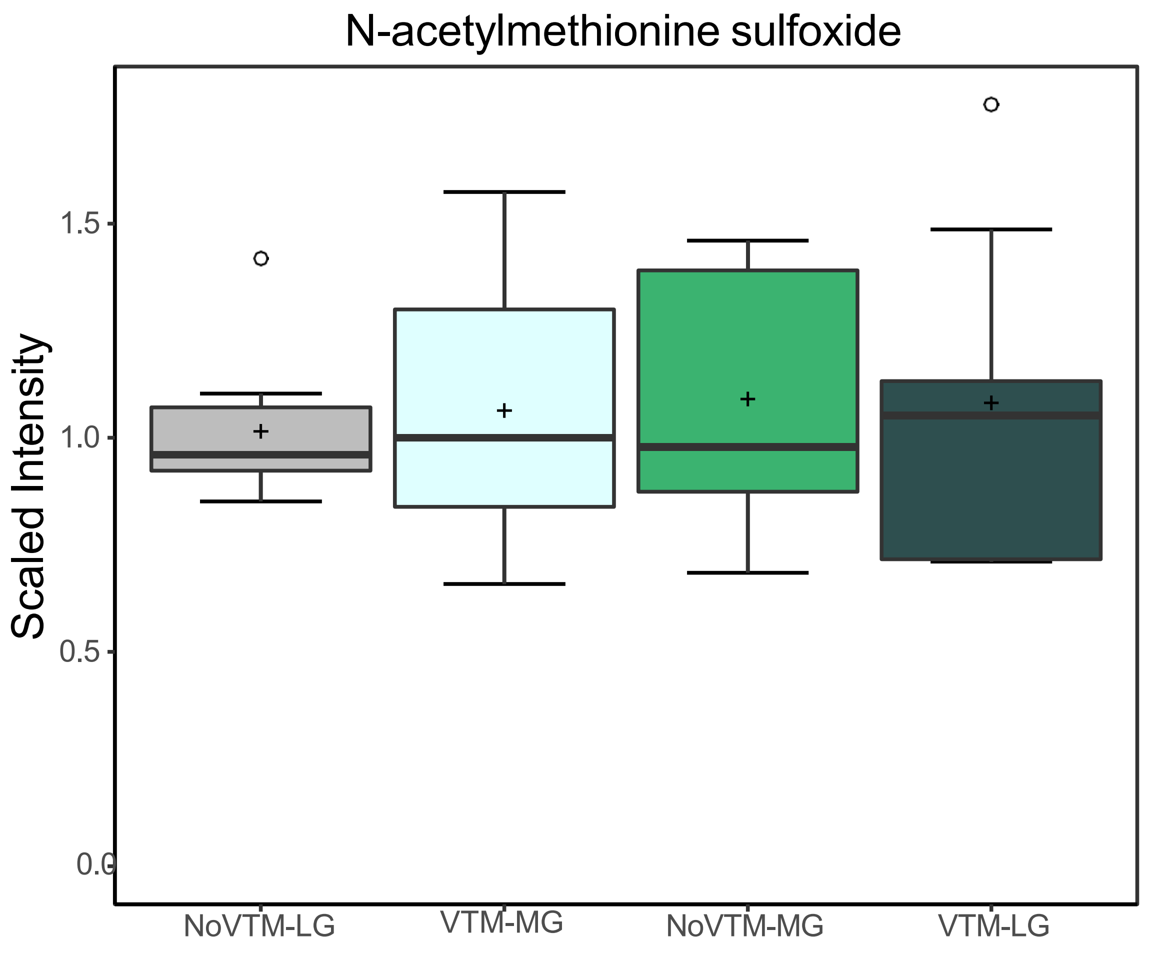
N-acetylmethionine sulfoxide
Scaled Intensity
1.5
1.0
0.5
0.0
NoVTM-LG
VTM-MG
NoVTM-MG
VTM-LG

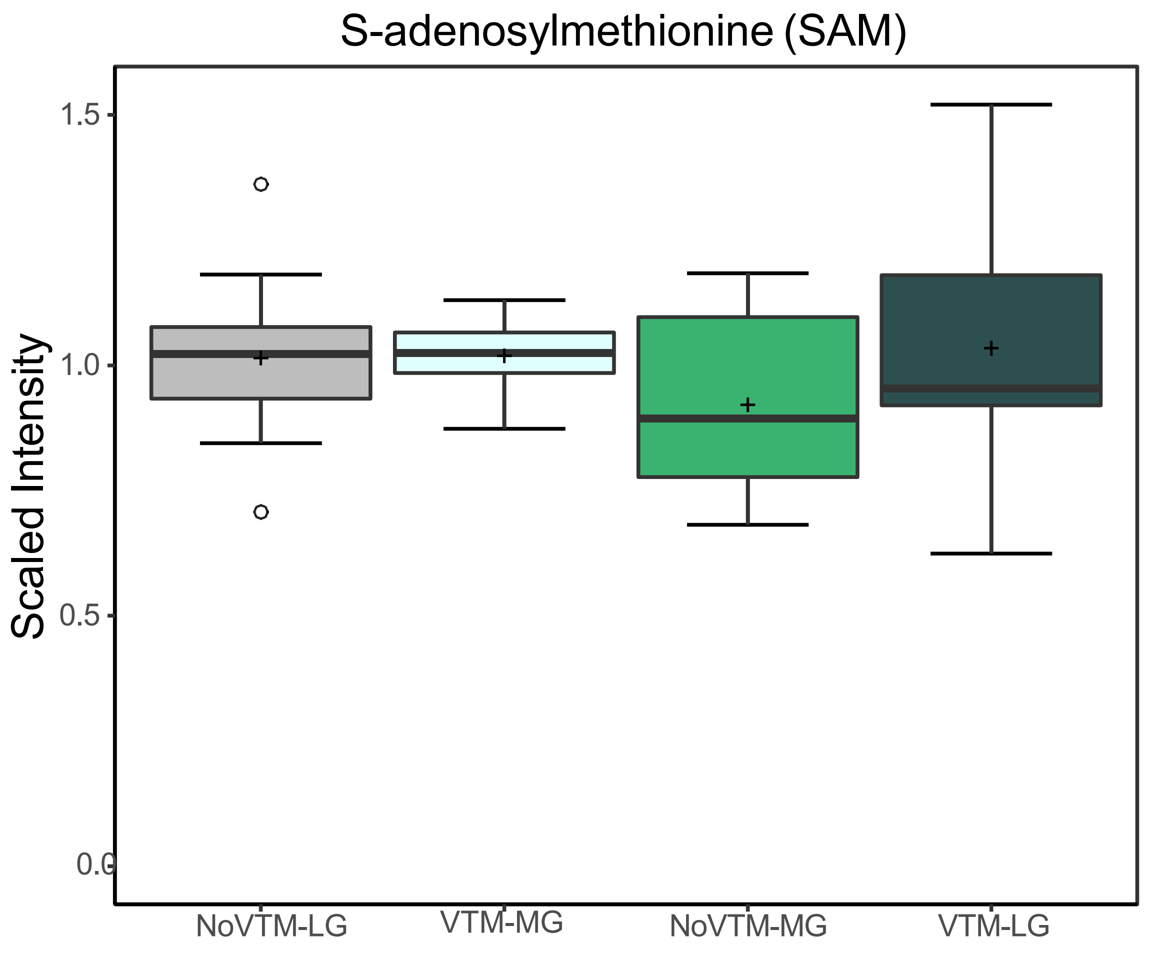
S-adenosylmethionine (SAM)
Scaled Intensity
1.5
1.0
0.5
0.0
NoVTM-LG
VTM-MG
NoVTM-MG
VTM-LG

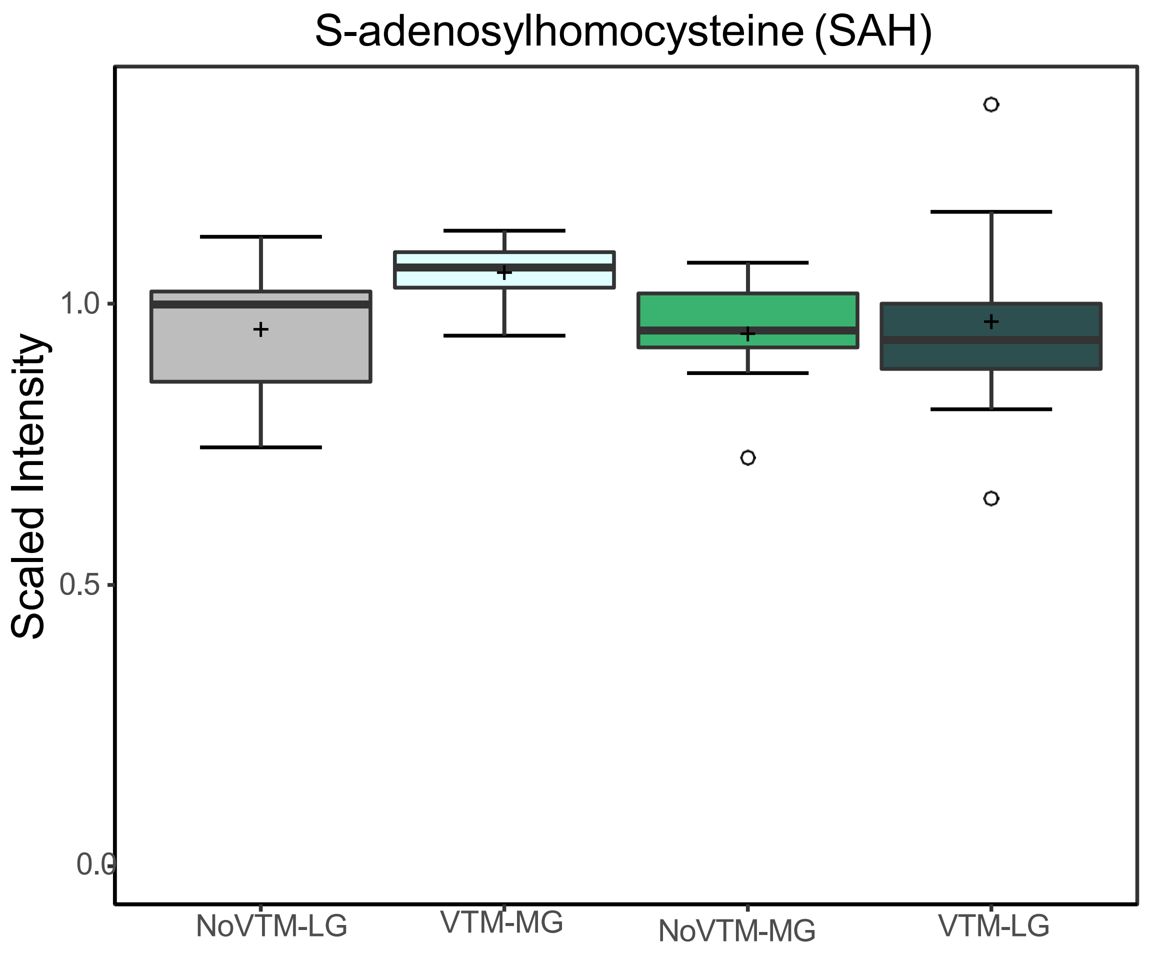
S-adenosylhomocysteine (SAH)
Scaled Intensity
1.0
0.5
0.0
NoVTM-LG
VTM-MG
NoVTM-MG
VTM-LG

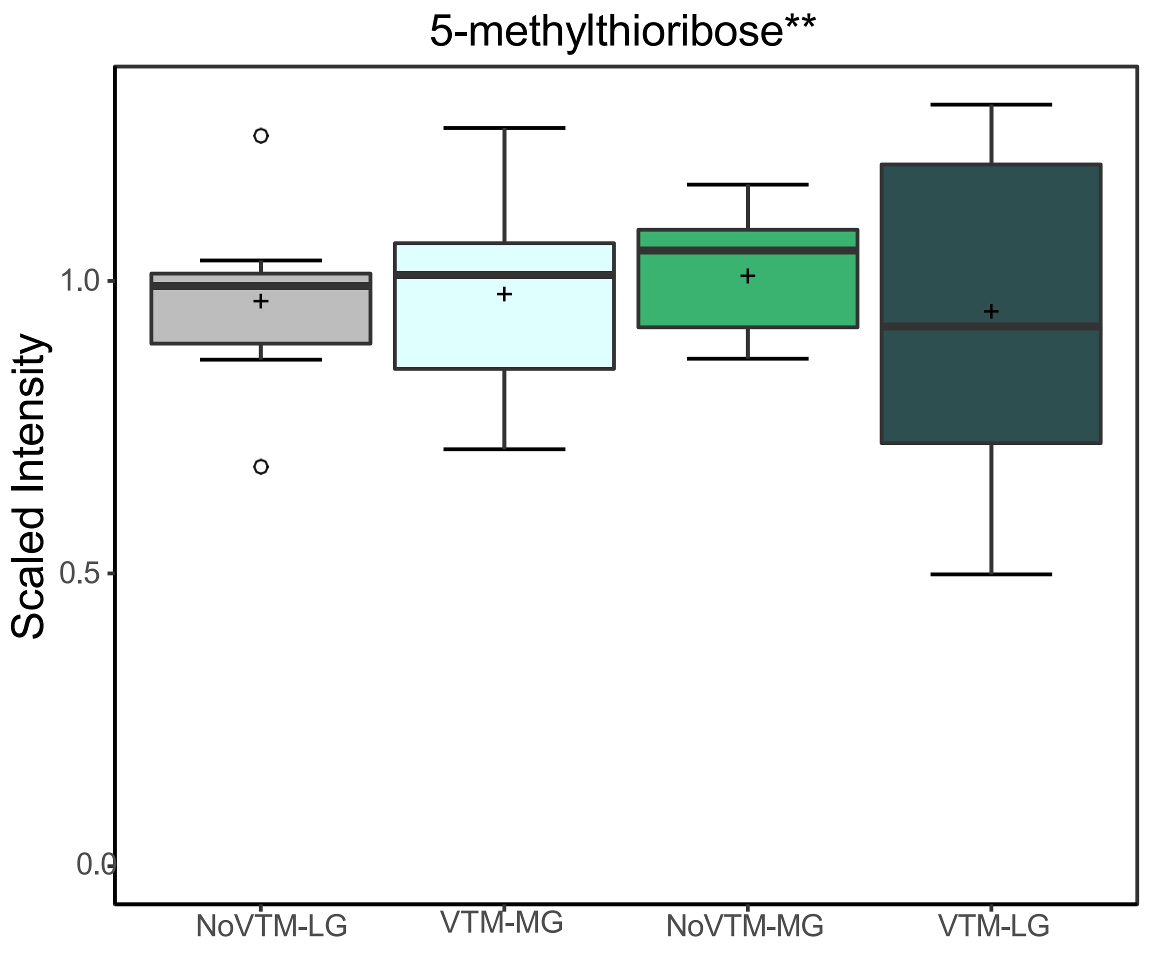
5-methylthioribose**
Scaled Intensity
1.0
0.5
0.0
NoVTM-LG
VTM-MG
NoVTM-MG
VTM-LG

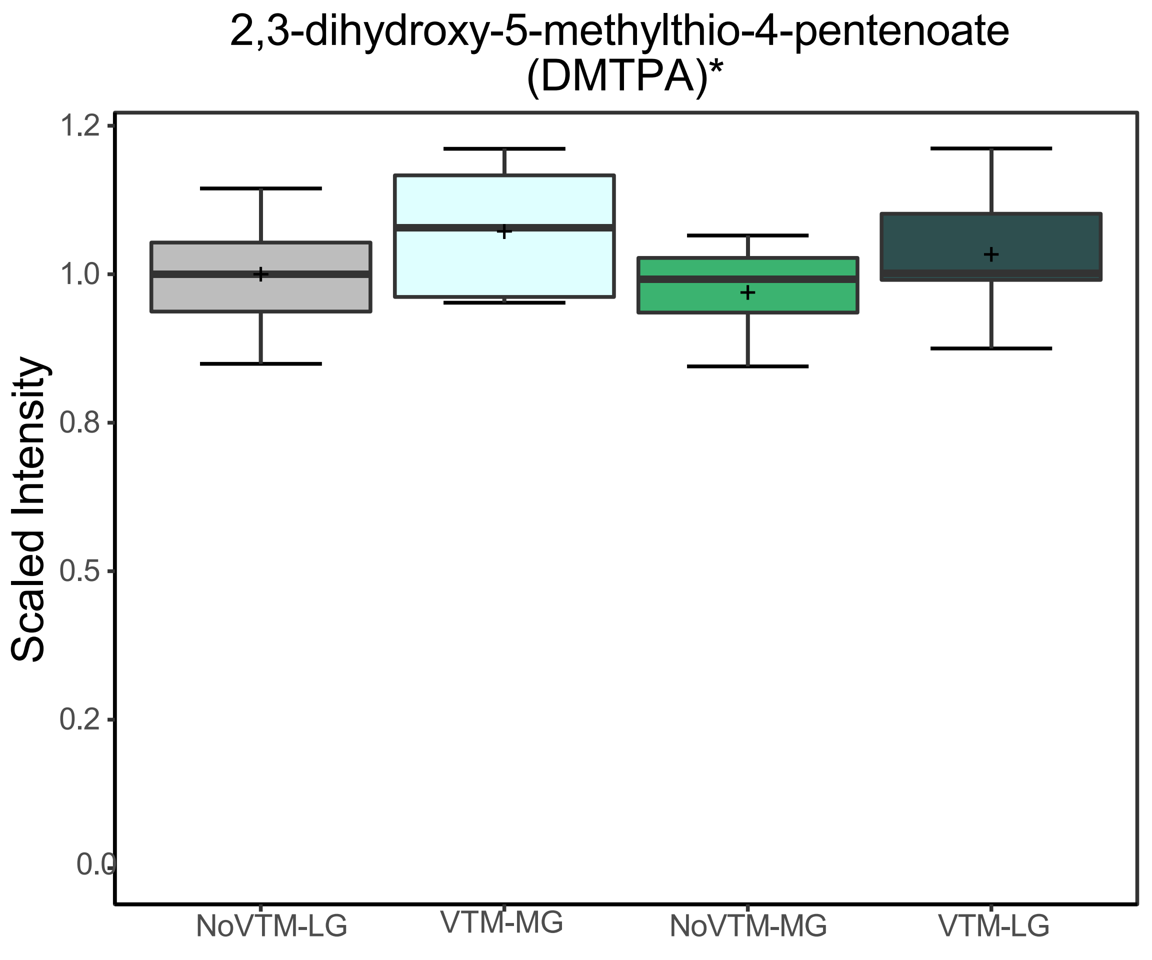
2,3-dihydroxy-5-methylthio-4-pentenoate
(DMTPA)*
Scaled Intensity
1.2
1.0
0.8
0.5
0.2
0.0
NoVTM-LG
VTM-MG
NoVTM-MG
VTM-LG

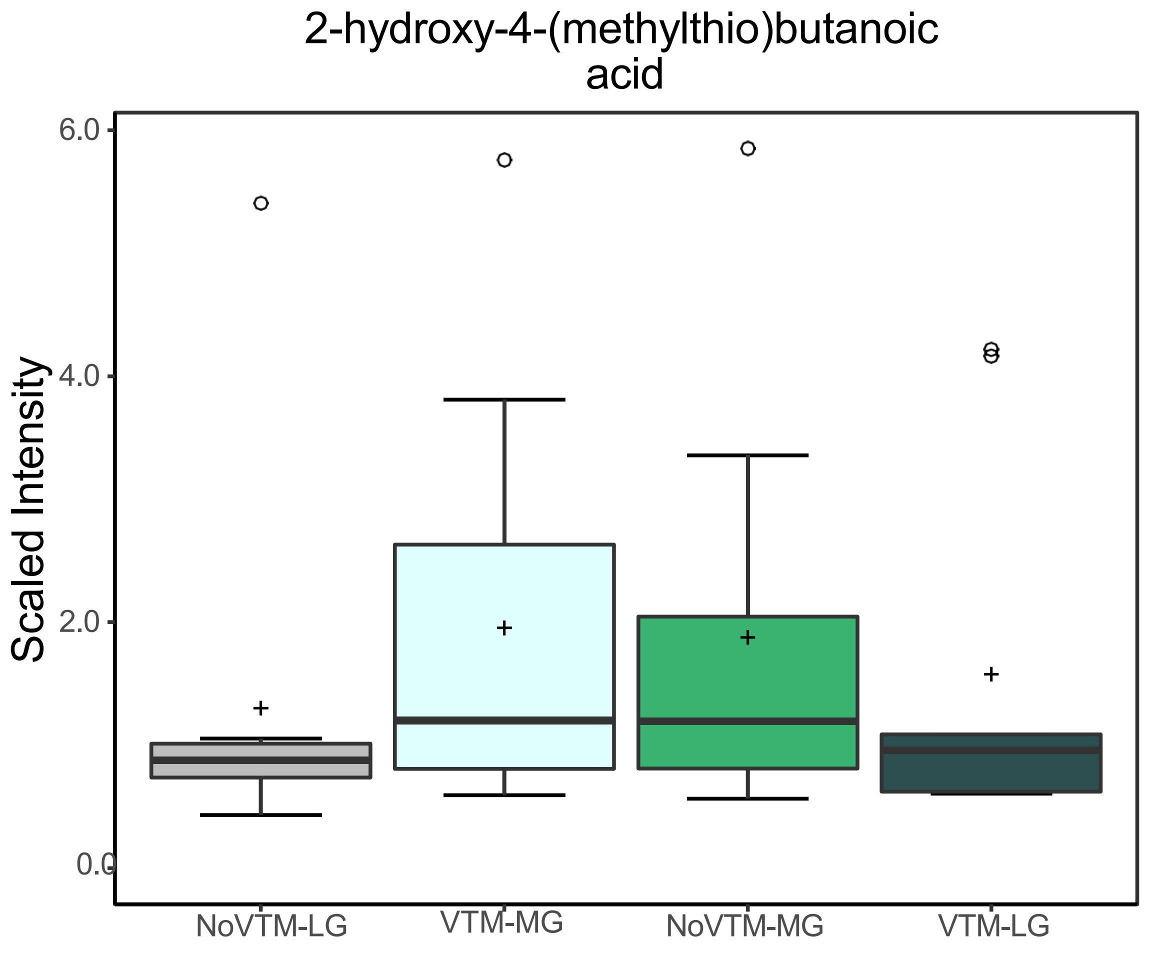
2-hydroxy-4-(methylthio)butanoic acid
Scaled Intensity
6.0
4.0
2.0
0.0
NoVTM-LG
VTM-MG
NoVTM-MG
VTM-LG

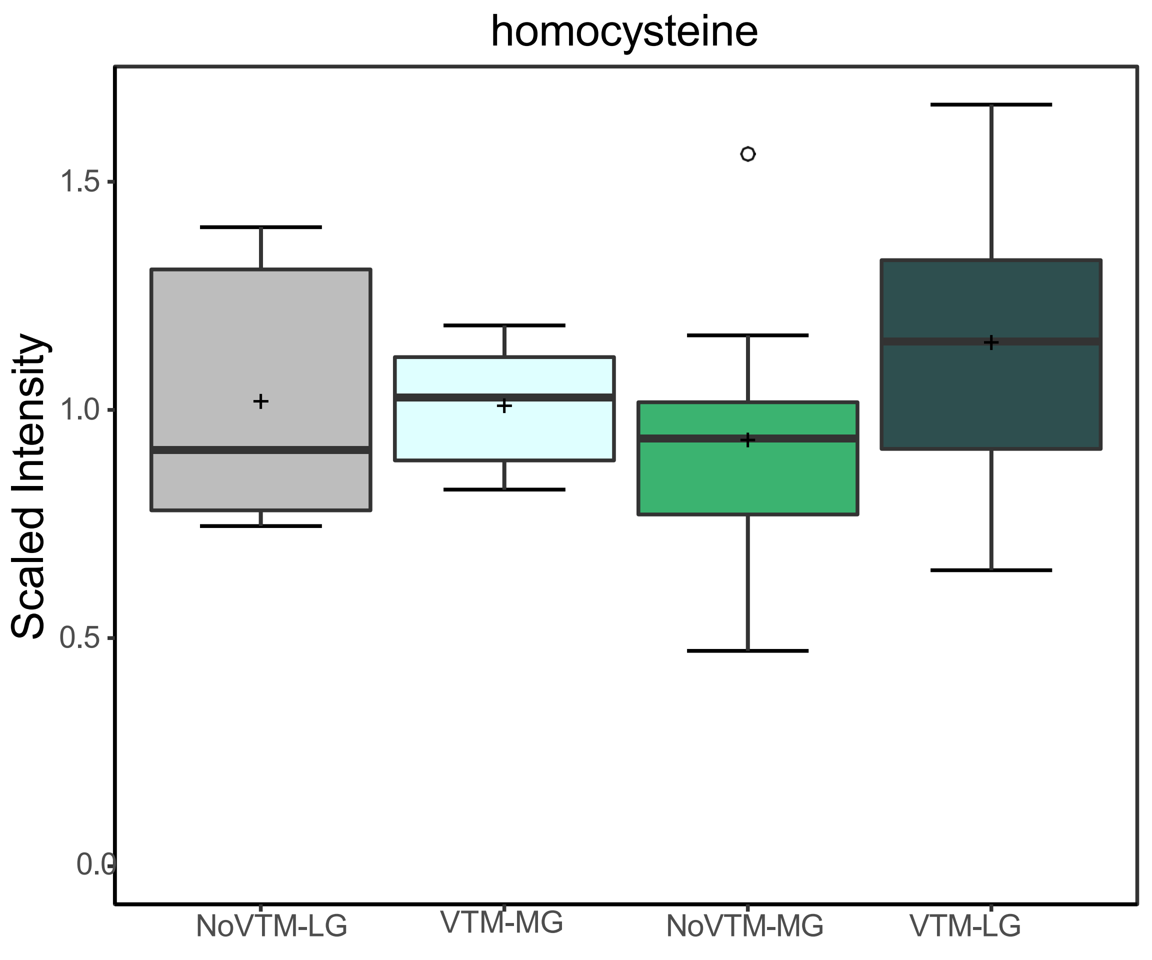
homocysteine
Scaled Intensity
1.5
1.0
0.5
0.0
NoVTM-LG
VTM-MG
NoVTM-MG
VTM-LG

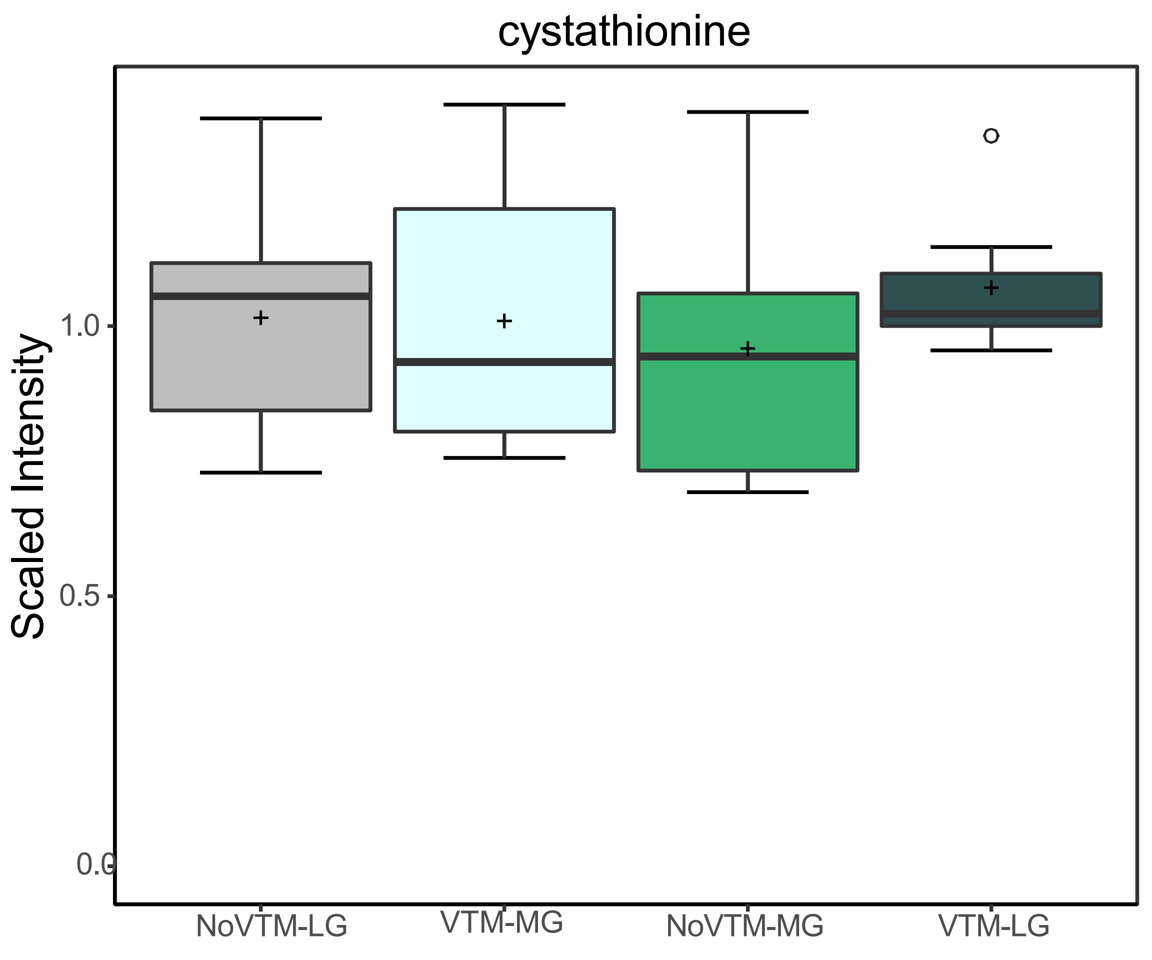
cystathionine
Scaled Intensity
1.0
0.5
0.0
NoVTM-LG
VTM-MG
NoVTM-MG
VTM-LG

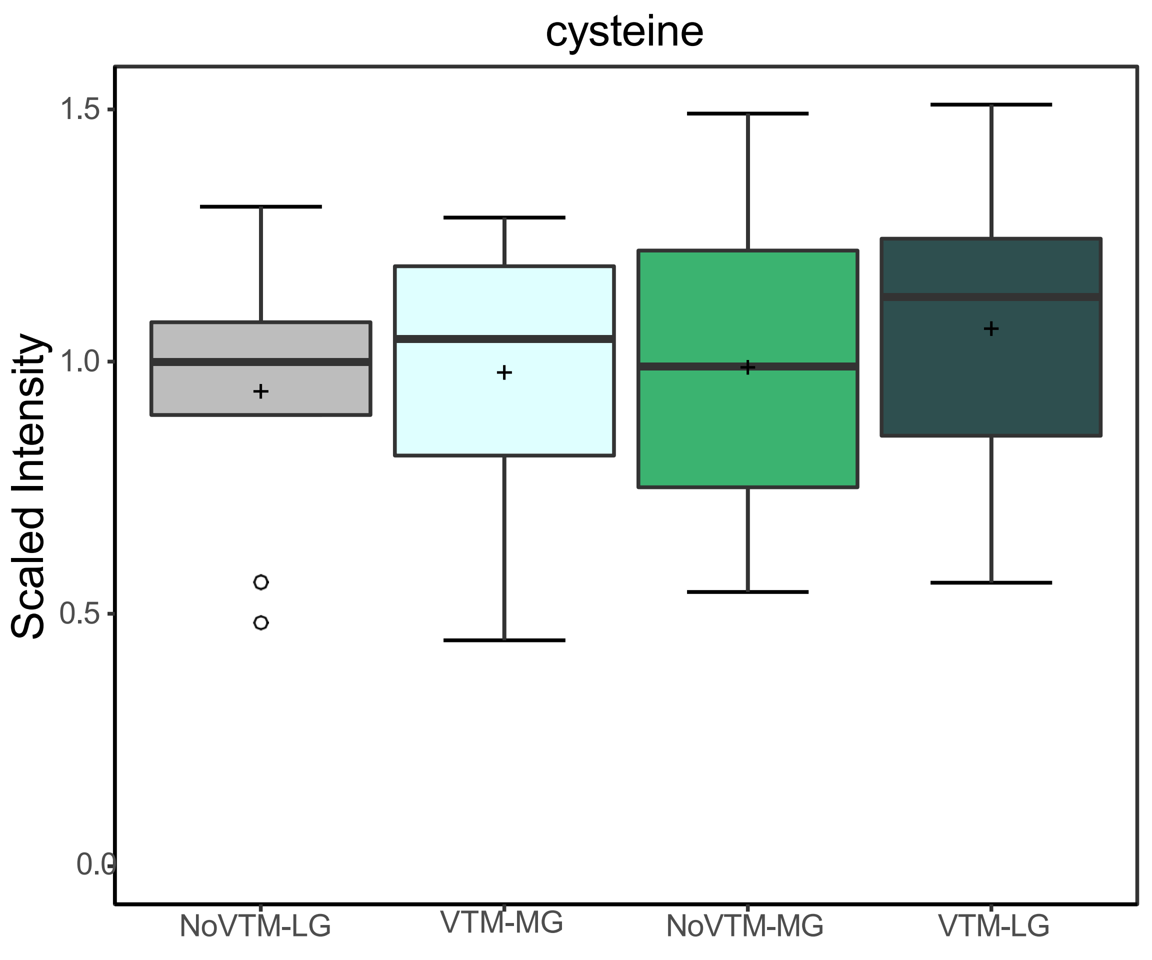

cysteine
Scaled Intensity
1.5
1.0
0.5
0.0
NoVTM-LG
VTM-MG
NoVTM-MG
VTM-LG

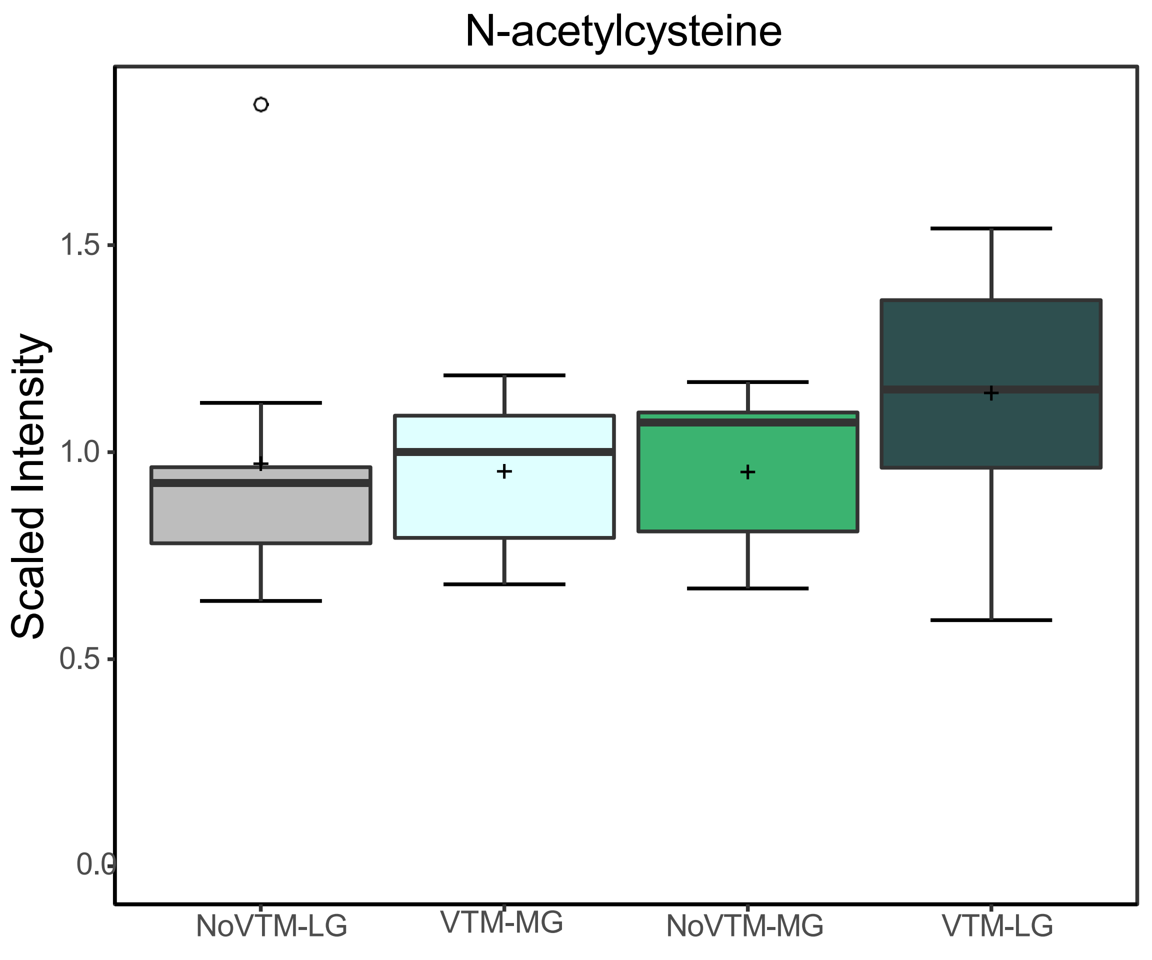

N-acetylcysteine
Scaled Intensity
1.5
1.0
0.5
0.0
NoVTM-LG
VTM-MG
NoVTM-MG
VTM-LG

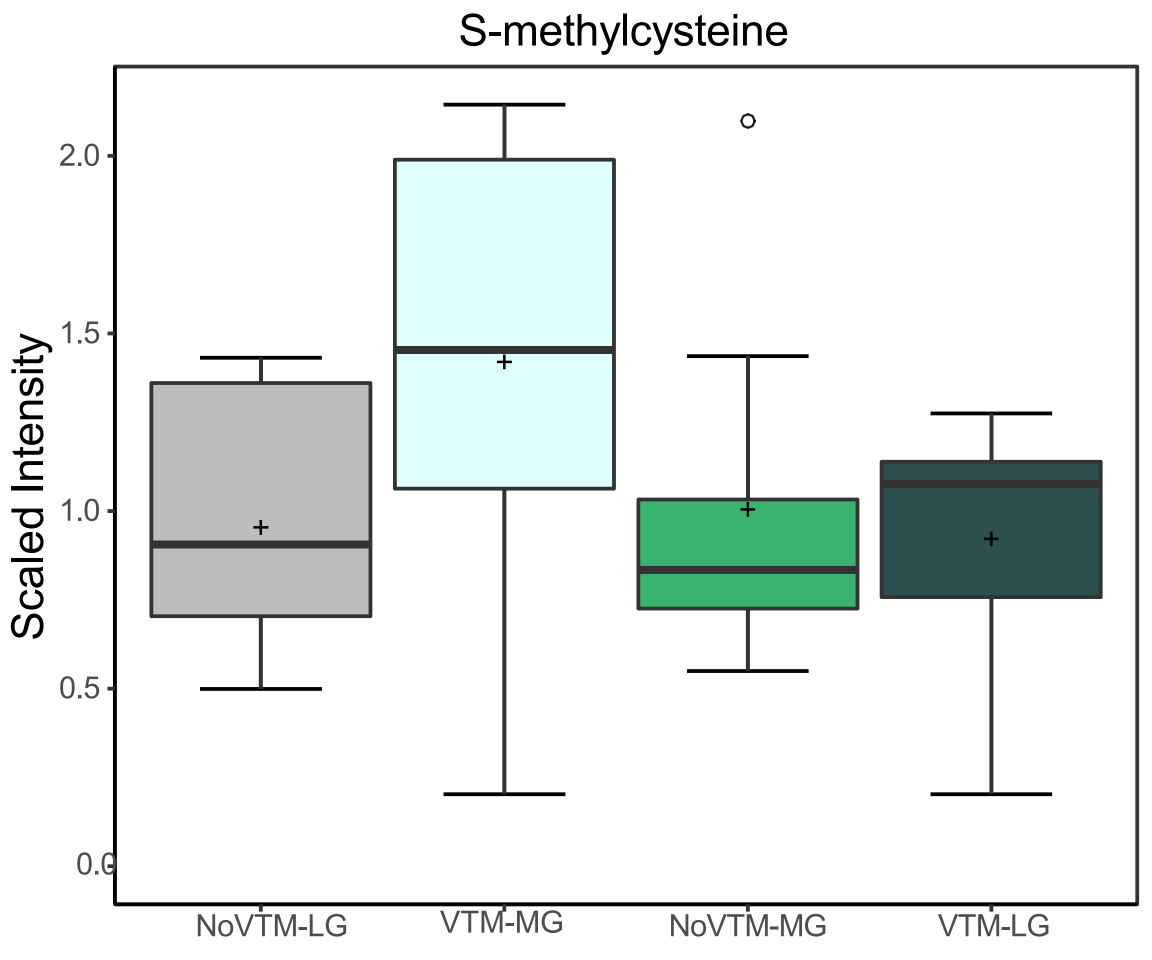

S-methylcysteine
Scaled Intensity
2.0
1.5
1.0
0.5
0.0
NoVTM-LG
VTM-MG
NoVTM-MG
VTM-LG

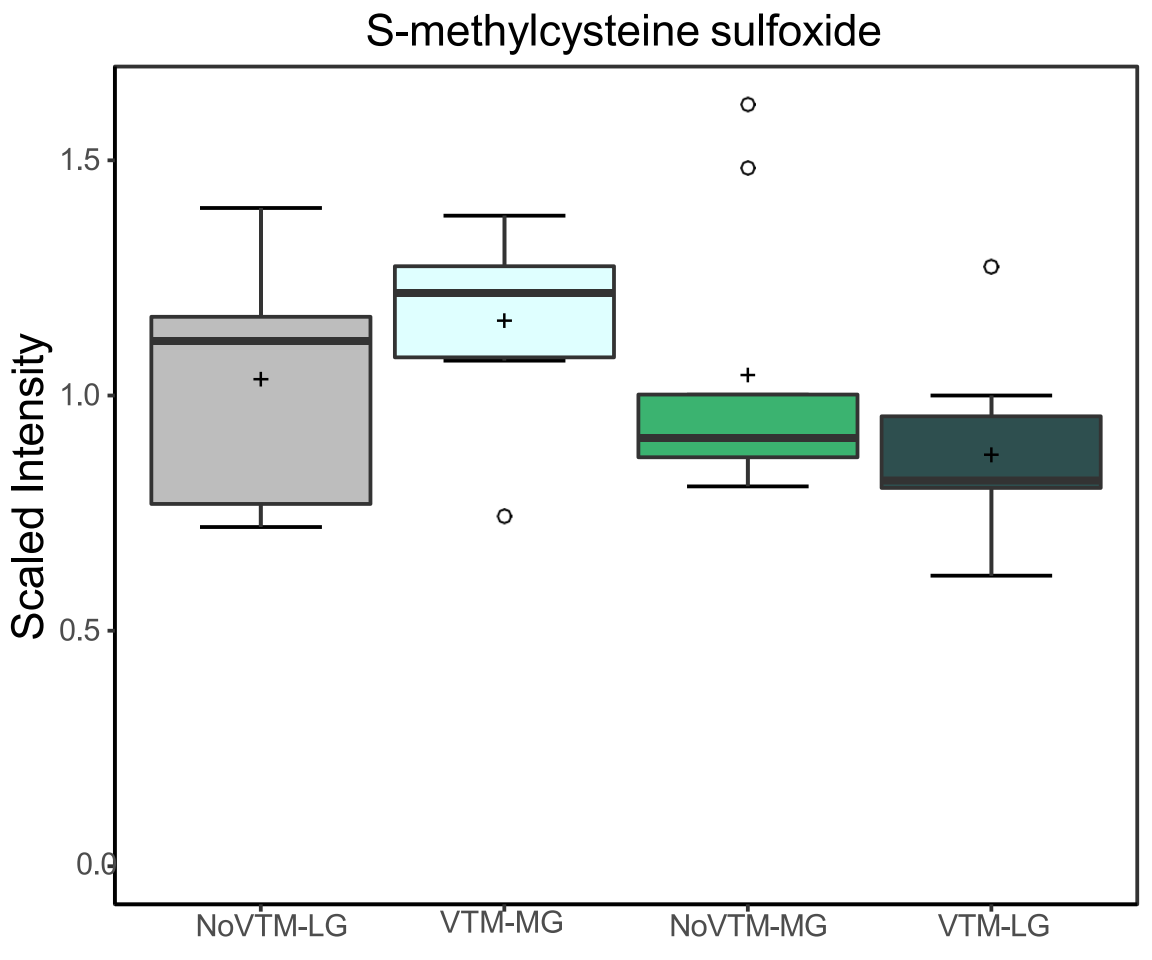
S-methylcysteine sulfoxide
Scaled Intensity
1.5
1.0
0.5
0.0
NoVTM-LG
VTM-MG
NoVTM-MG
VTM-LG

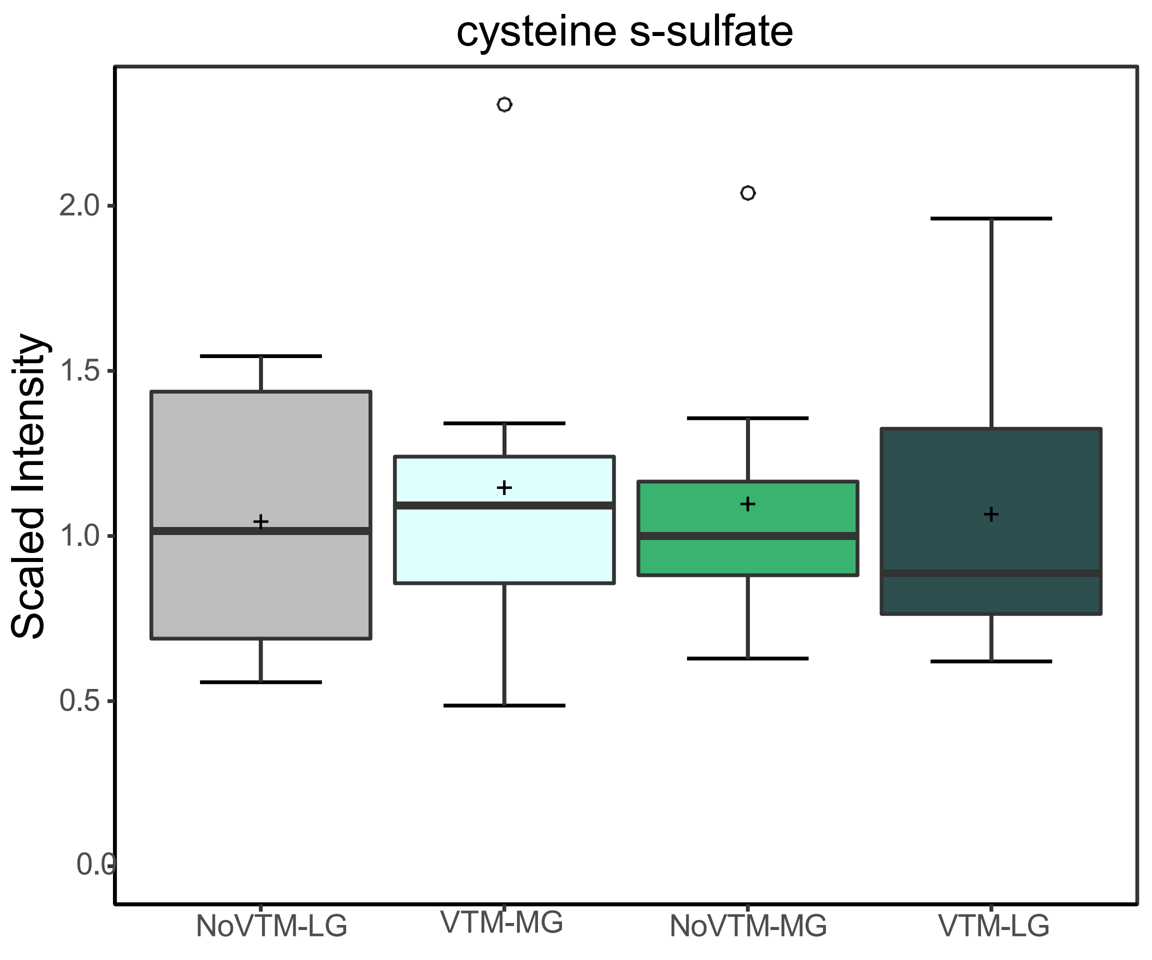

cysteine s-sulfate
Scaled Intensity
2.0
1.5
1.0
0.5
0.0
NoVTM-LG
VTM-MG
NoVTM-MG
VTM-LG

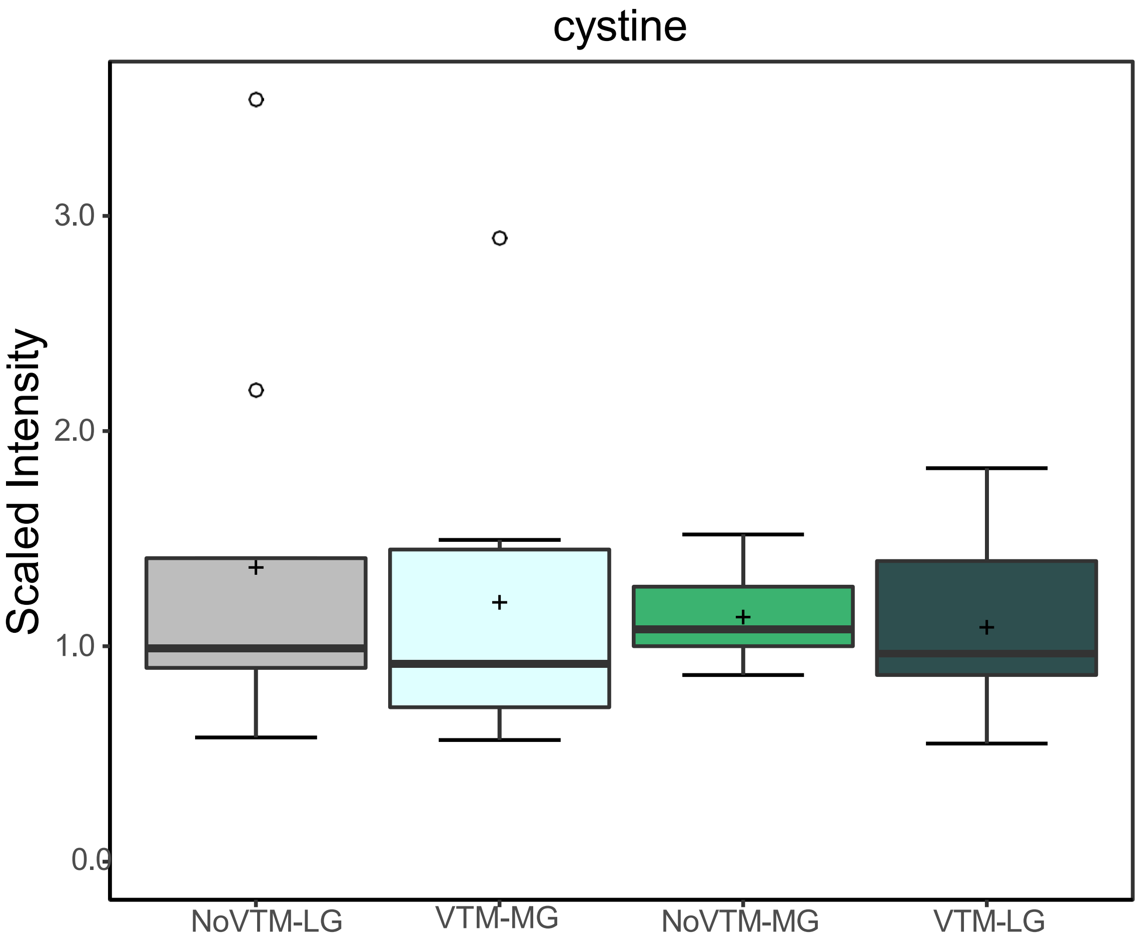

cystine
Scaled Intensity
3.0
2.0
1.0
0.0
NoVTM-LG
VTM-MG
NoVTM-MG
VTM-LG

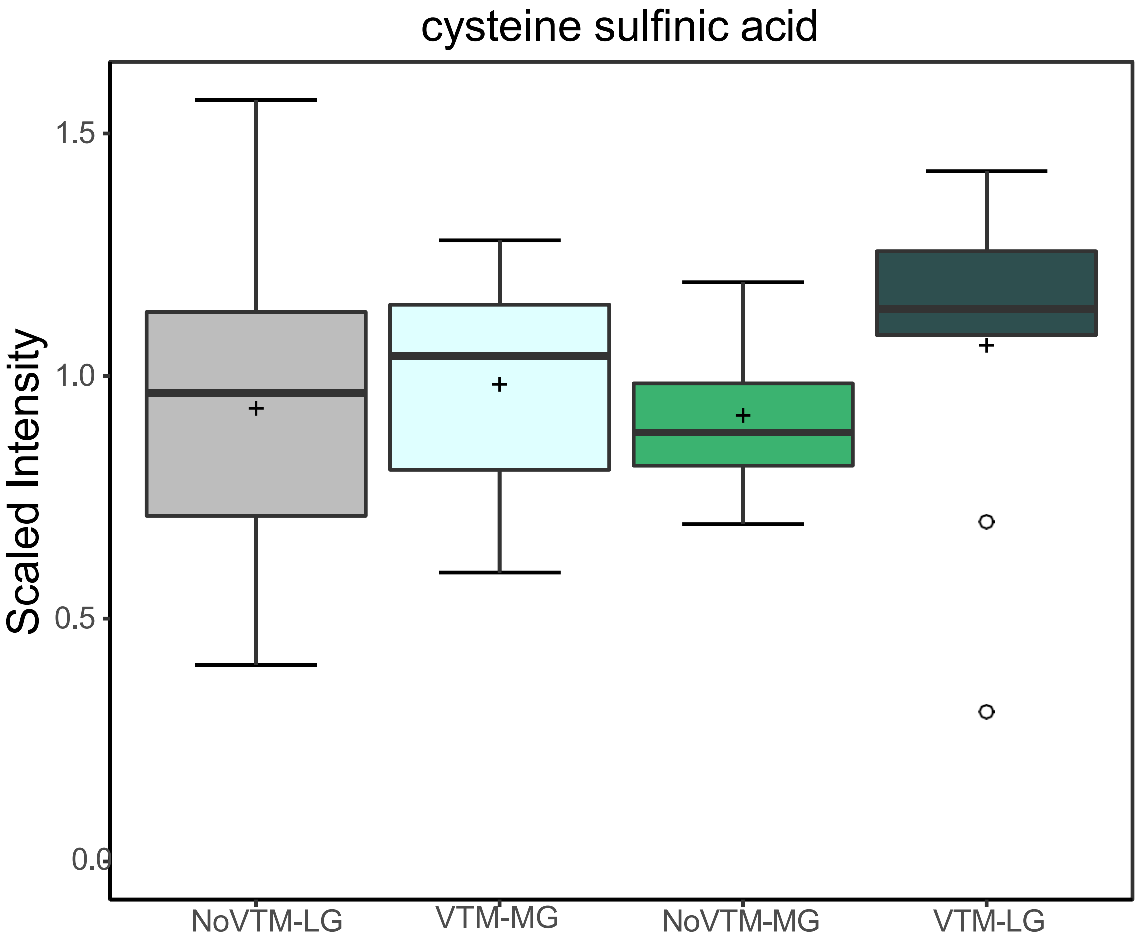

cysteine sulfinic acid
Scaled Intensity
1.5
1.0
0.5
0.0
NoVTM-LG
VTM-MG
NoVTM-MG
VTM-LG

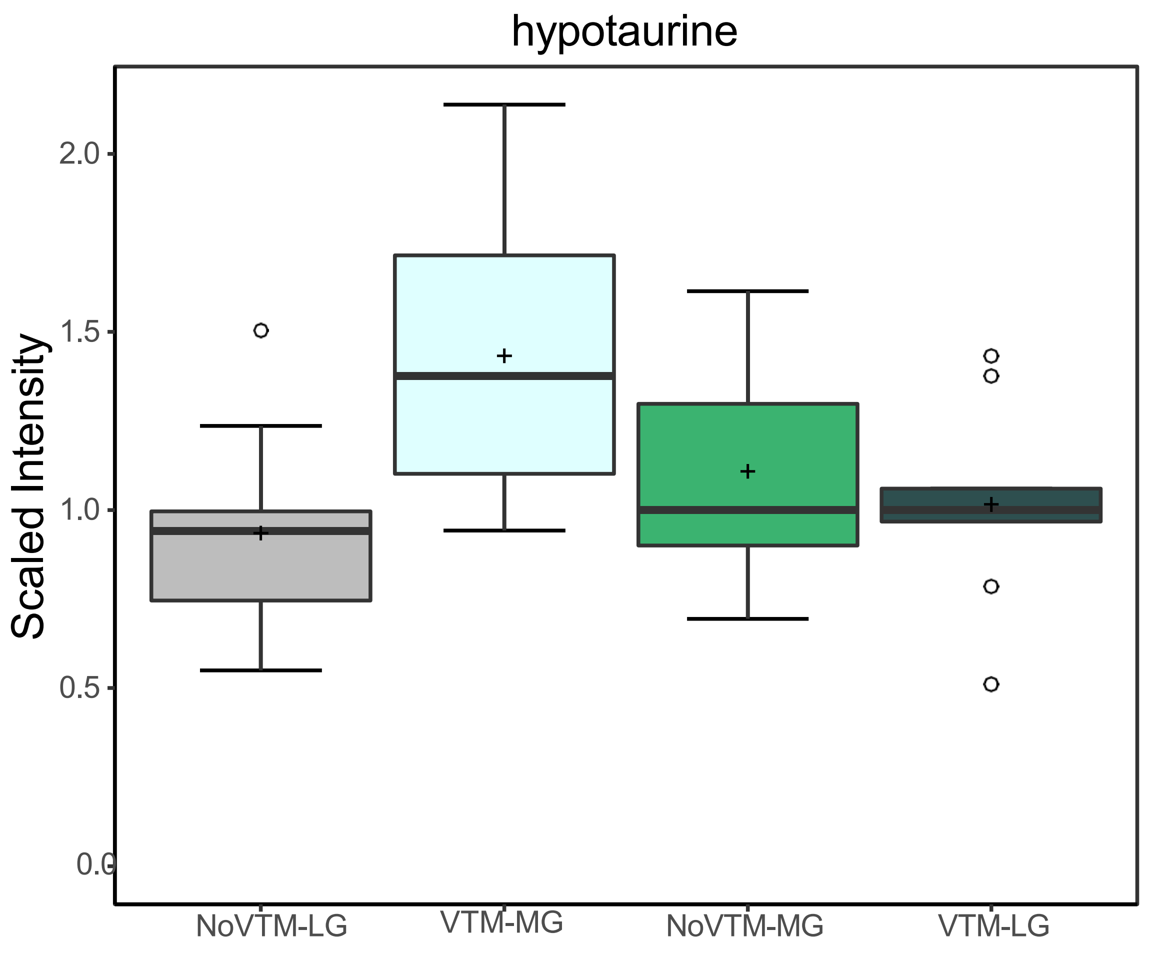

hypotaurine
Scaled Intensity
2.0
1.5
1.0
0.5
0.0
NoVTM-LG
VTM-MG
NoVTM-MG
VTM-LG

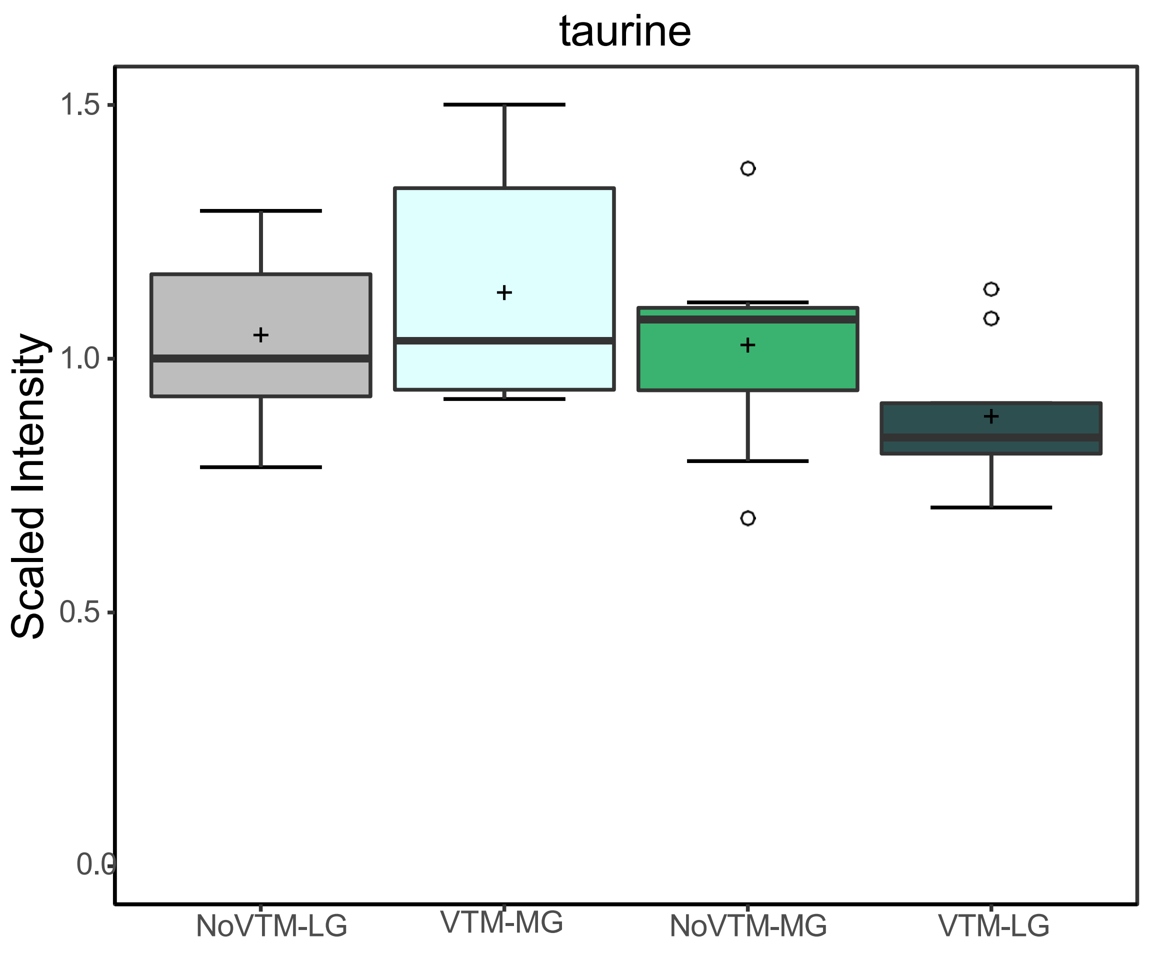

taurine
Scaled Intensity
1.5
1.0
0.5
0.0
NoVTM-LG
VTM-MG
NoVTM-MG
VTM-LG

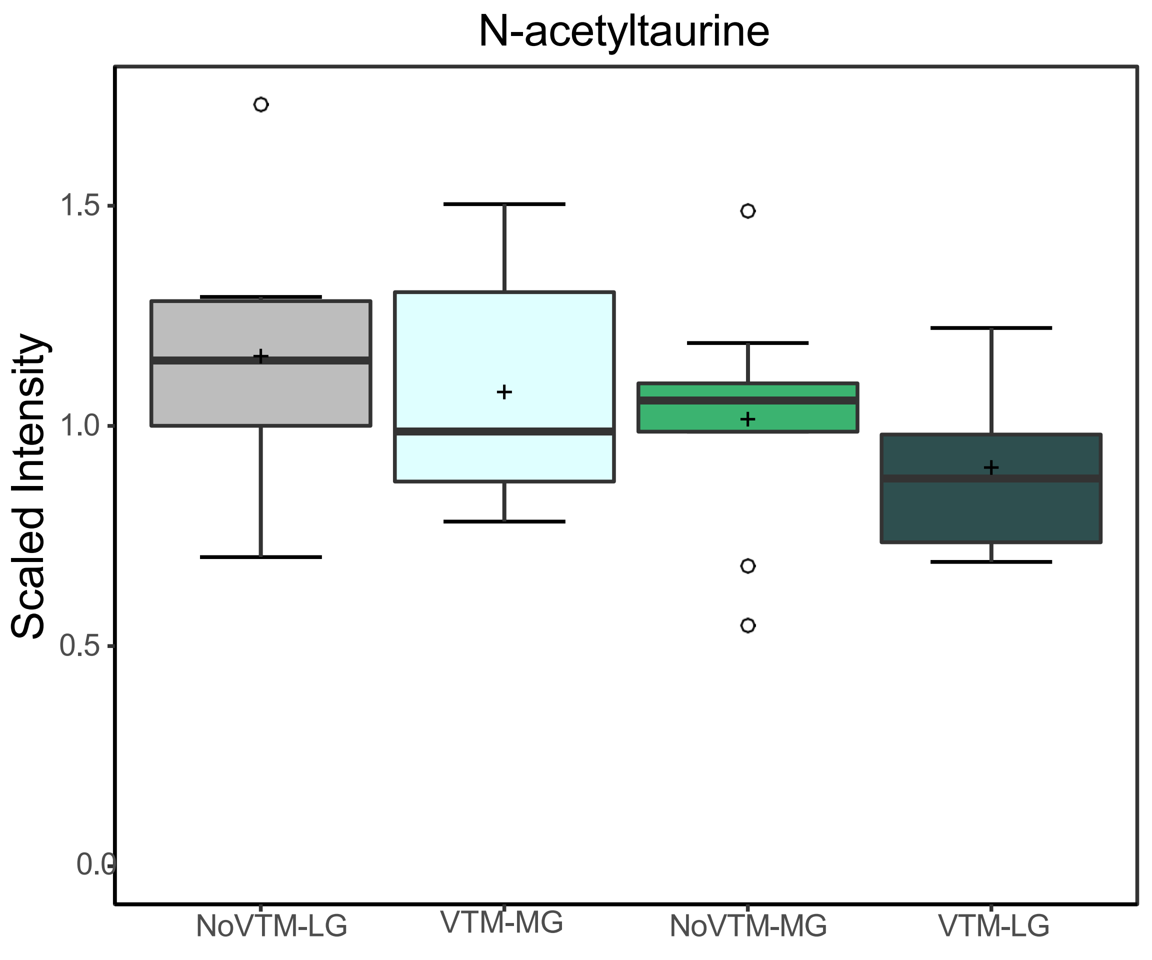
N-acetyltaurine
Scaled Intensity
1.5
1.0
0.5
0.0
NoVTM-LG
VTM-MG
NoVTM-MG
VTM-LG

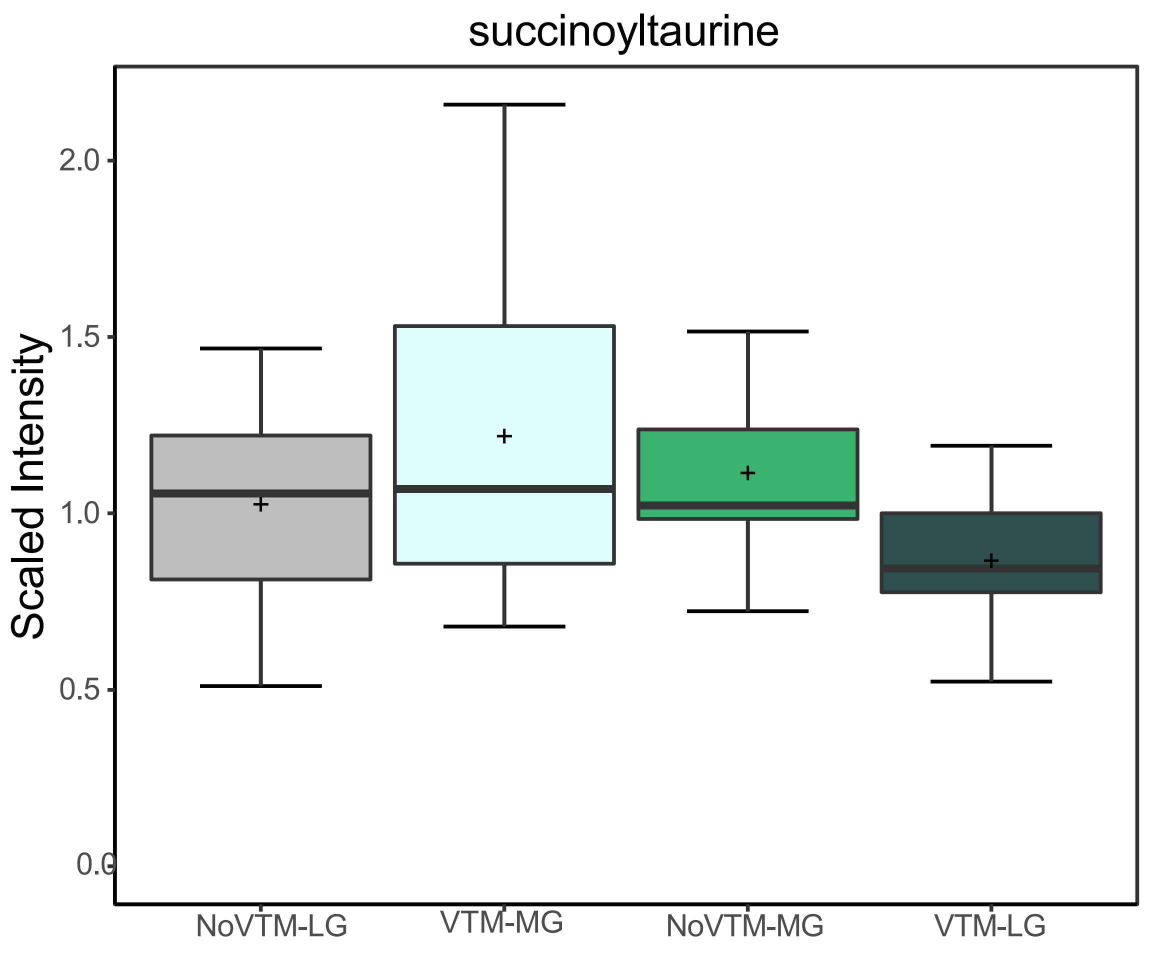
succinoyltaurine
Scaled Intensity
2.0
1.5
1.0
0.5
0.0
NoVTM-LG
VTM-MG
NoVTM-MG
VTM-LG

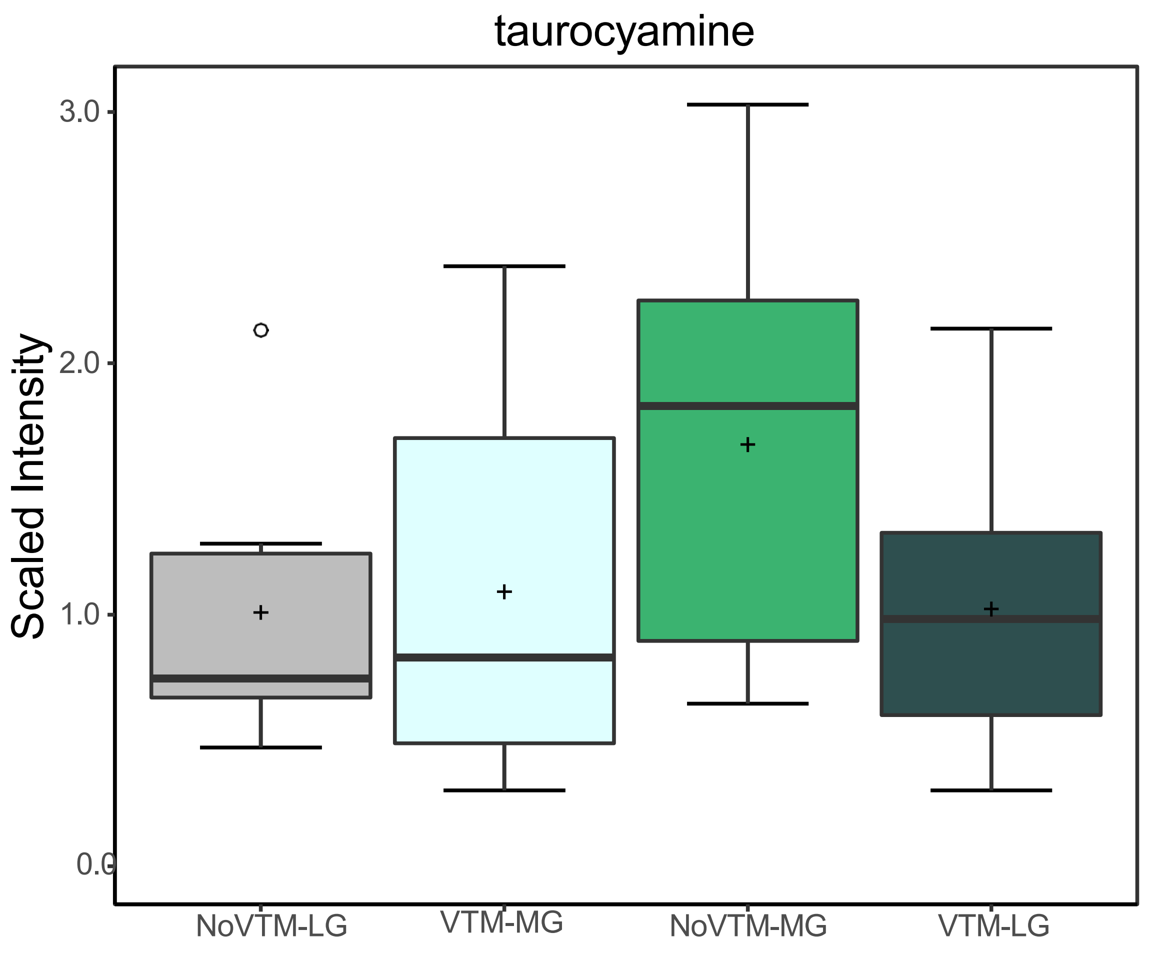
taurocyamine
Scaled Intensity
3.0
2.0
1.0
0.0
NoVTM-LG
VTM-MG
NoVTM-MG
VTM-LG

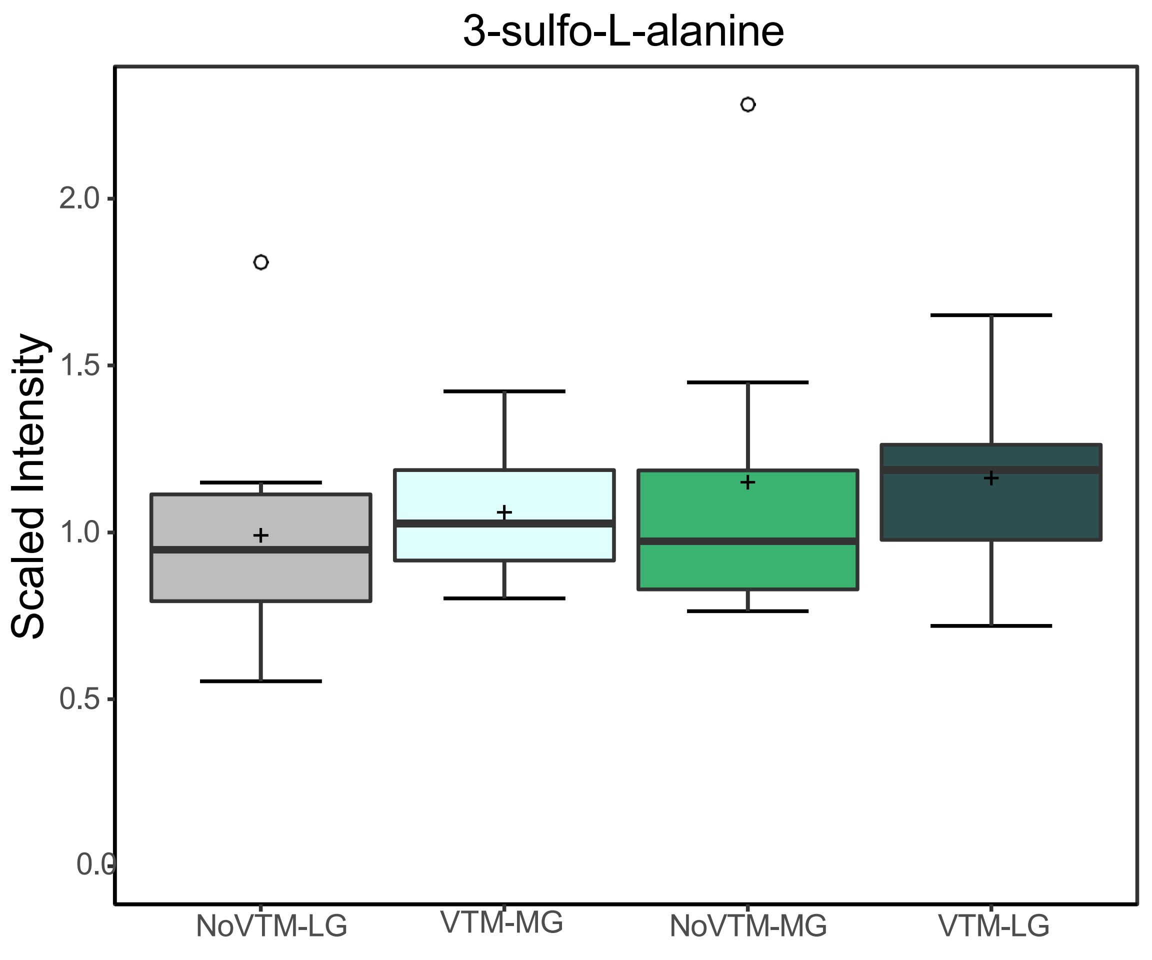
3-sulfo-L-alanine
Scaled Intensity
2.0
1.5
1.0
0.5
0.0
NoVTM-LG
VTM-MG
NoVTM-MG
VTM-LG

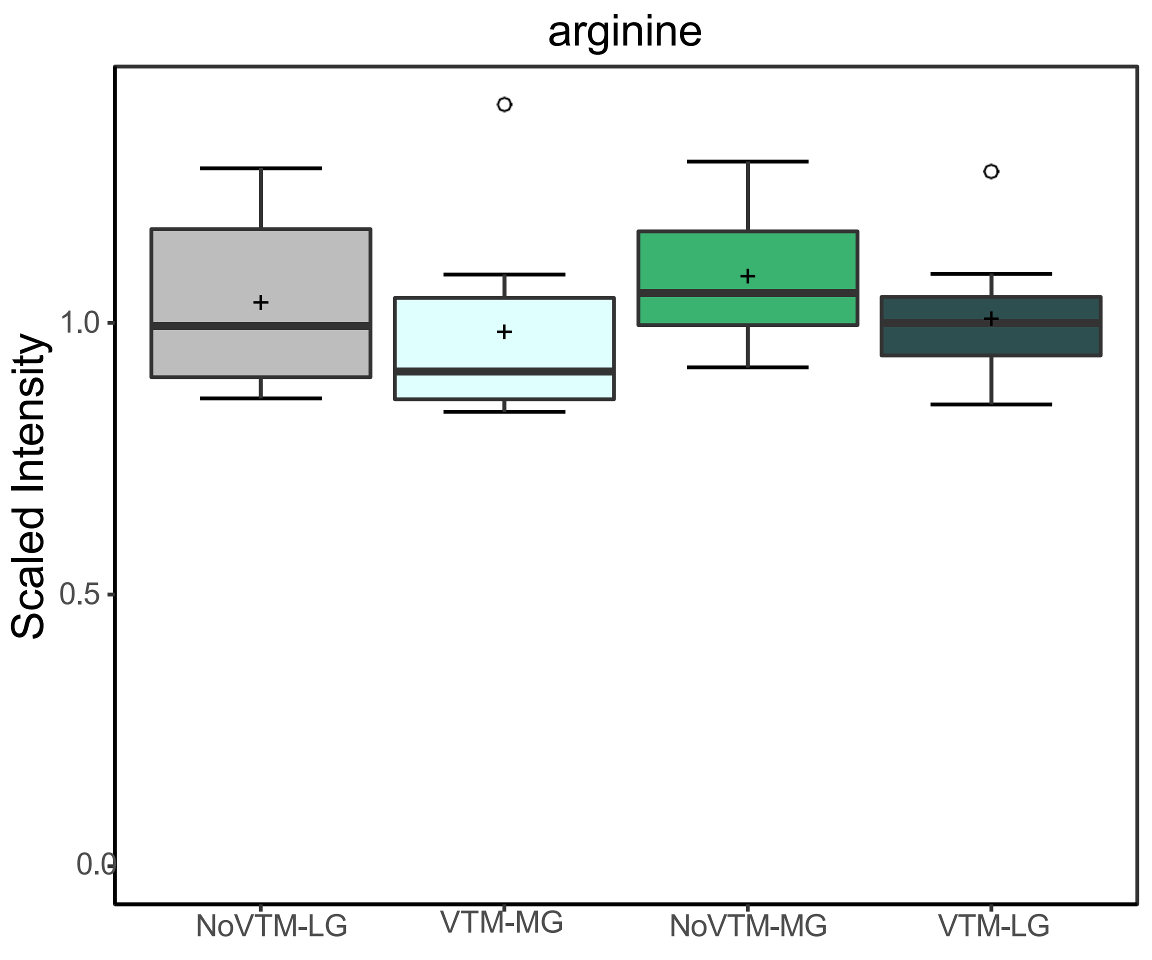
arginine
Scaled Intensity
1.0
0.5
0.0
NoVTM-LG
VTM-MG
NoVTM-MG
VTM-LG

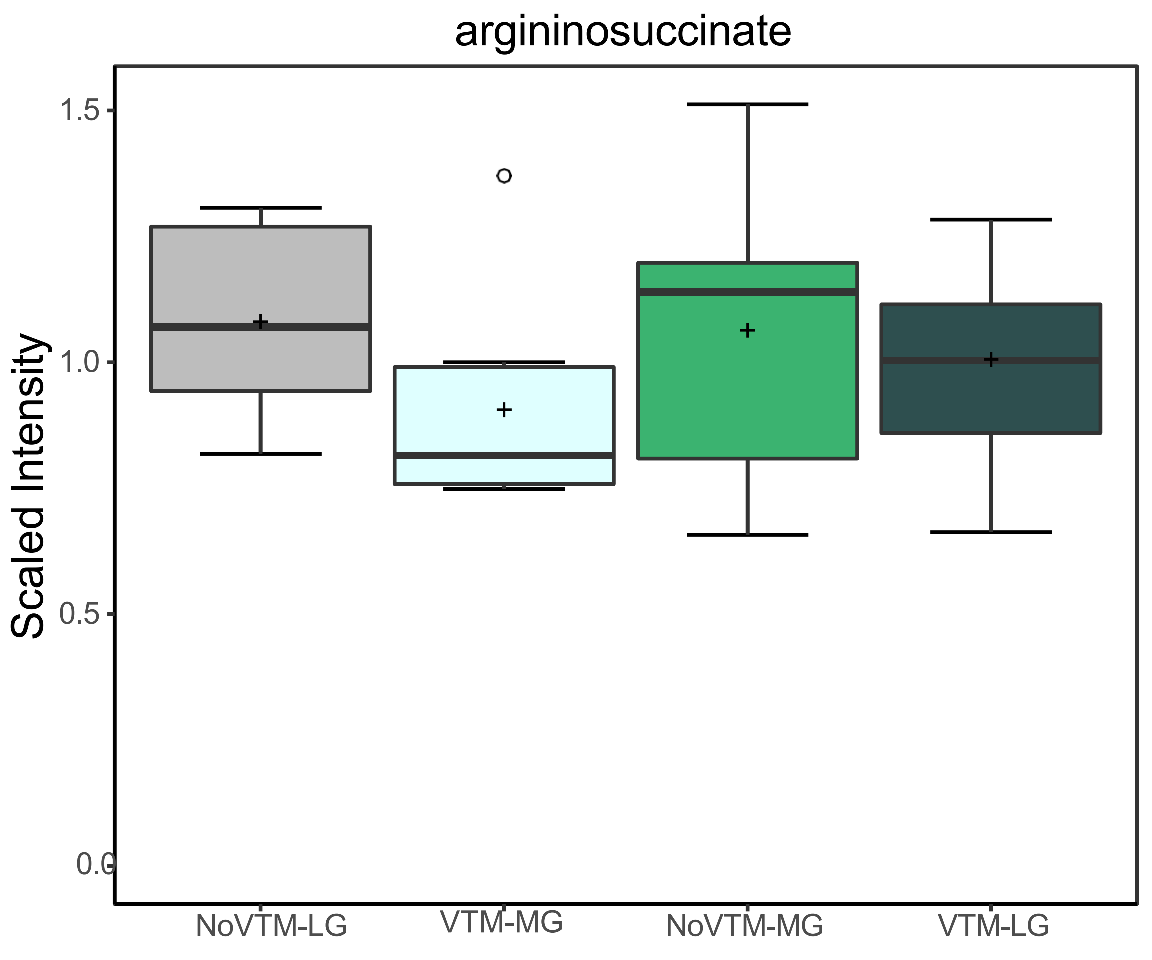
argininosuccinate
Scaled Intensity
1.5
1.0
0.5
0.0
NoVTM-LG
VTM-MG
NoVTM-MG
VTM-LG

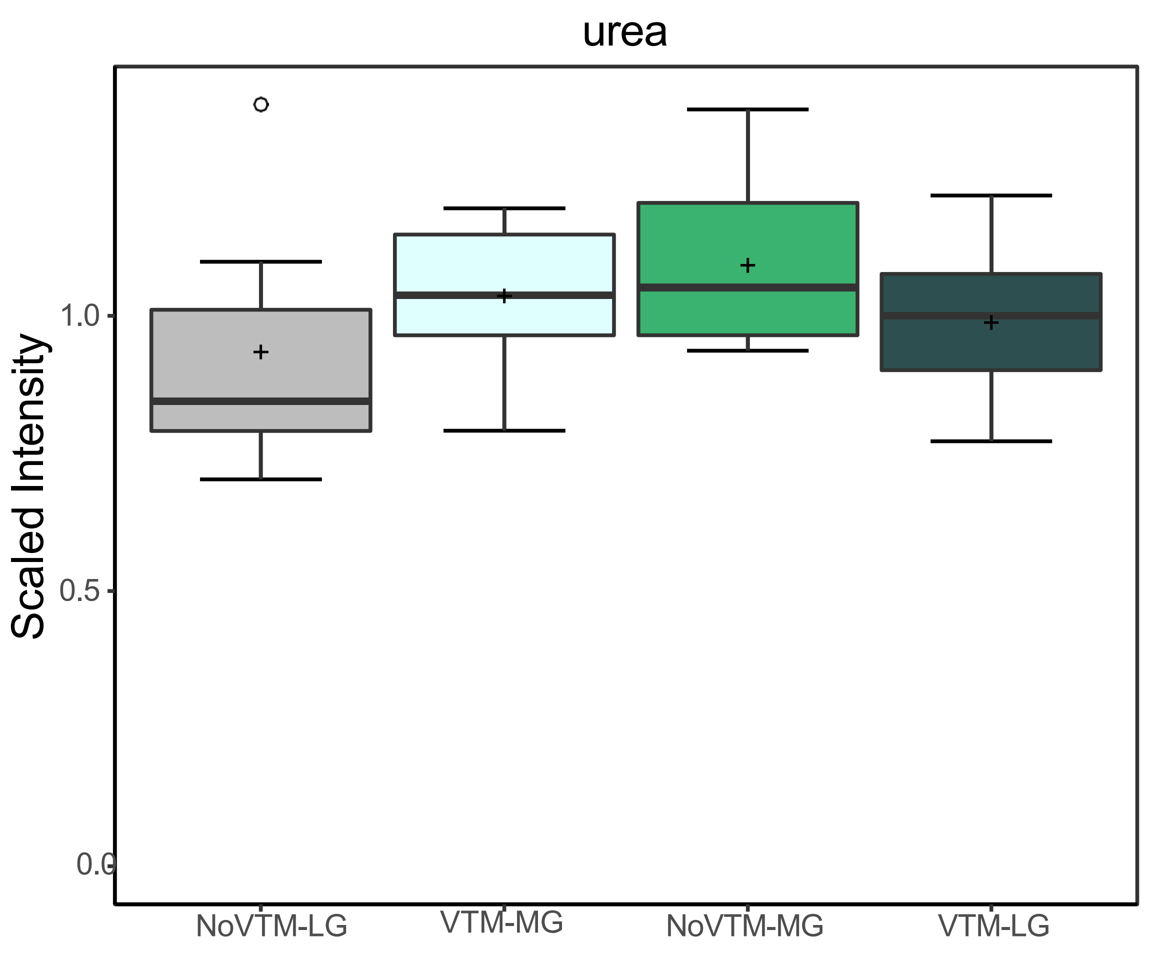
urea
Scaled Intensity
1.0
0.5
0.0
NoVTM-LG
VTM-MG
NoVTM-MG
VTM-LG

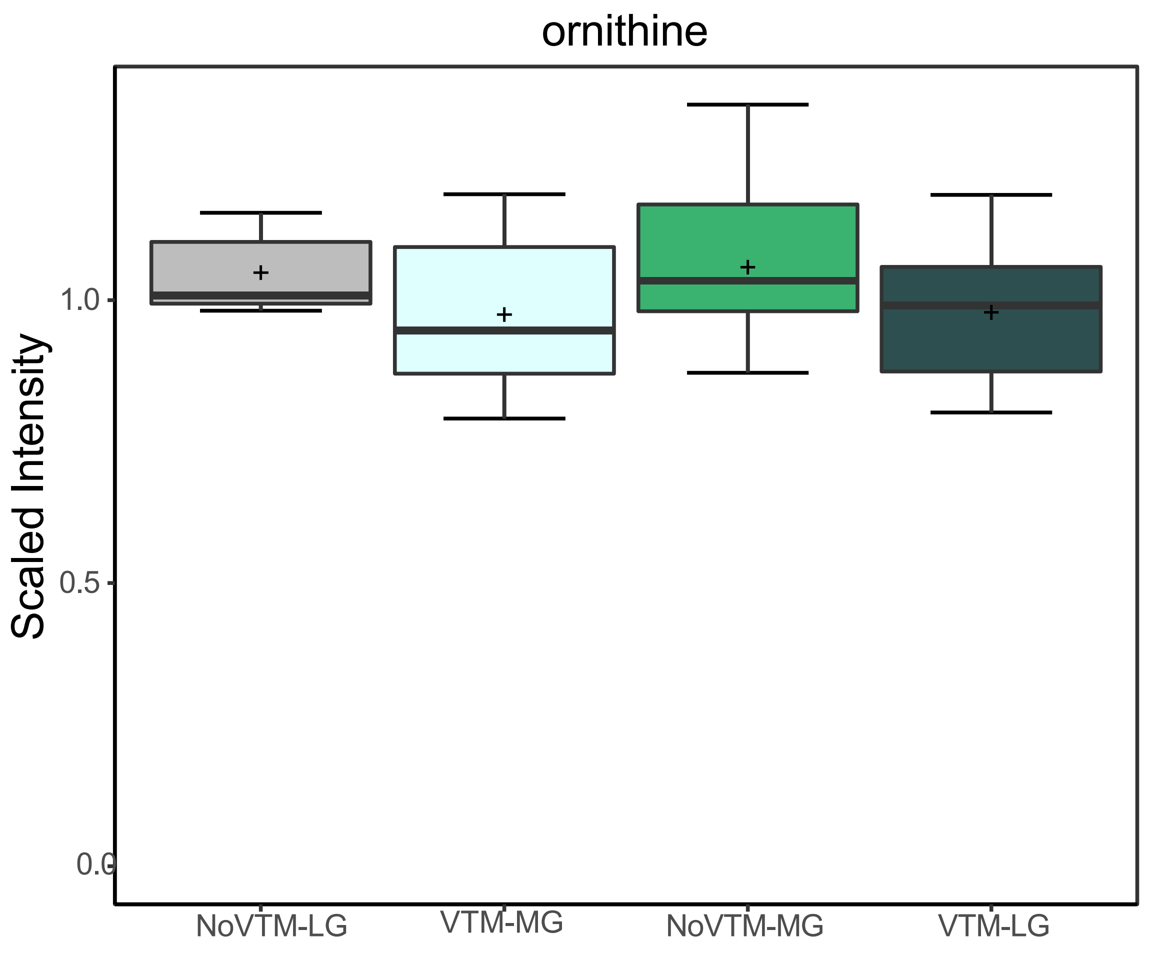
ornithine
Scaled Intensity
1.0
0.5
0.0
NoVTM-LG
VTM-MG
NoVTM-MG
VTM-LG

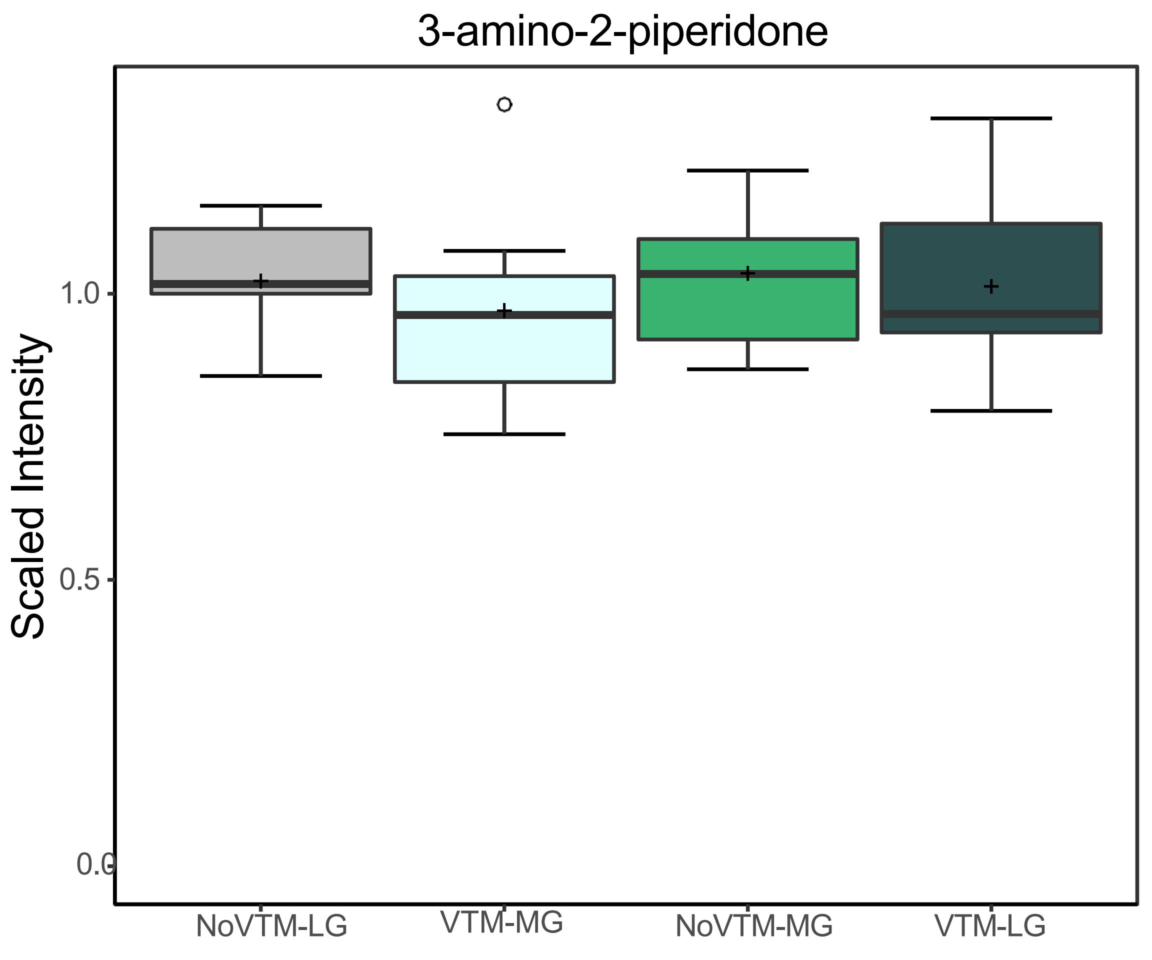
3-amino-2-piperidone
Scaled Intensity
1.0
0.5
0.0
NoVTM-LG
VTM-MG
NoVTM-MG
VTM-LG

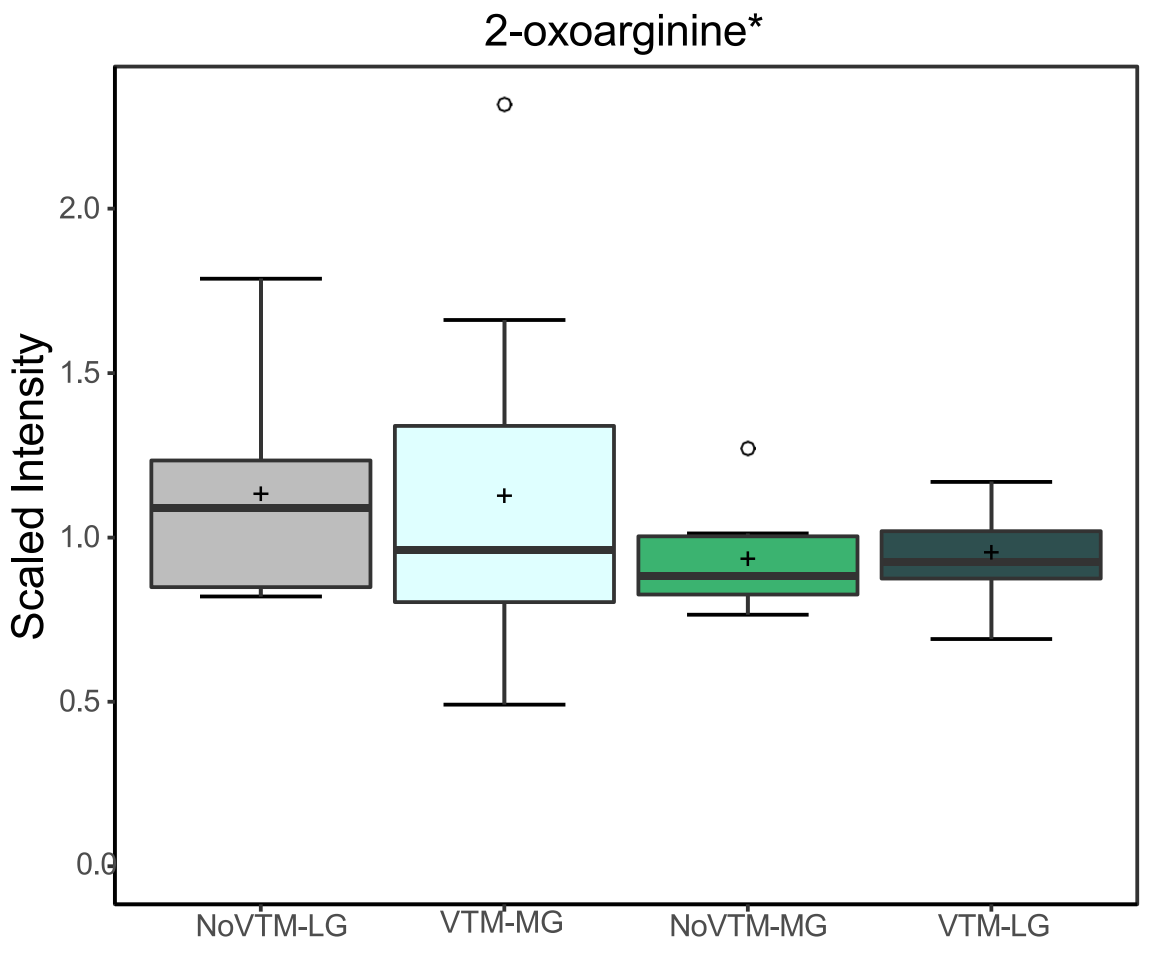
2-oxoarginine*
Scaled Intensity
2.0
1.5
1.0
0.5
0.0
NoVTM-LG
VTM-MG
NoVTM-MG
VTM-LG

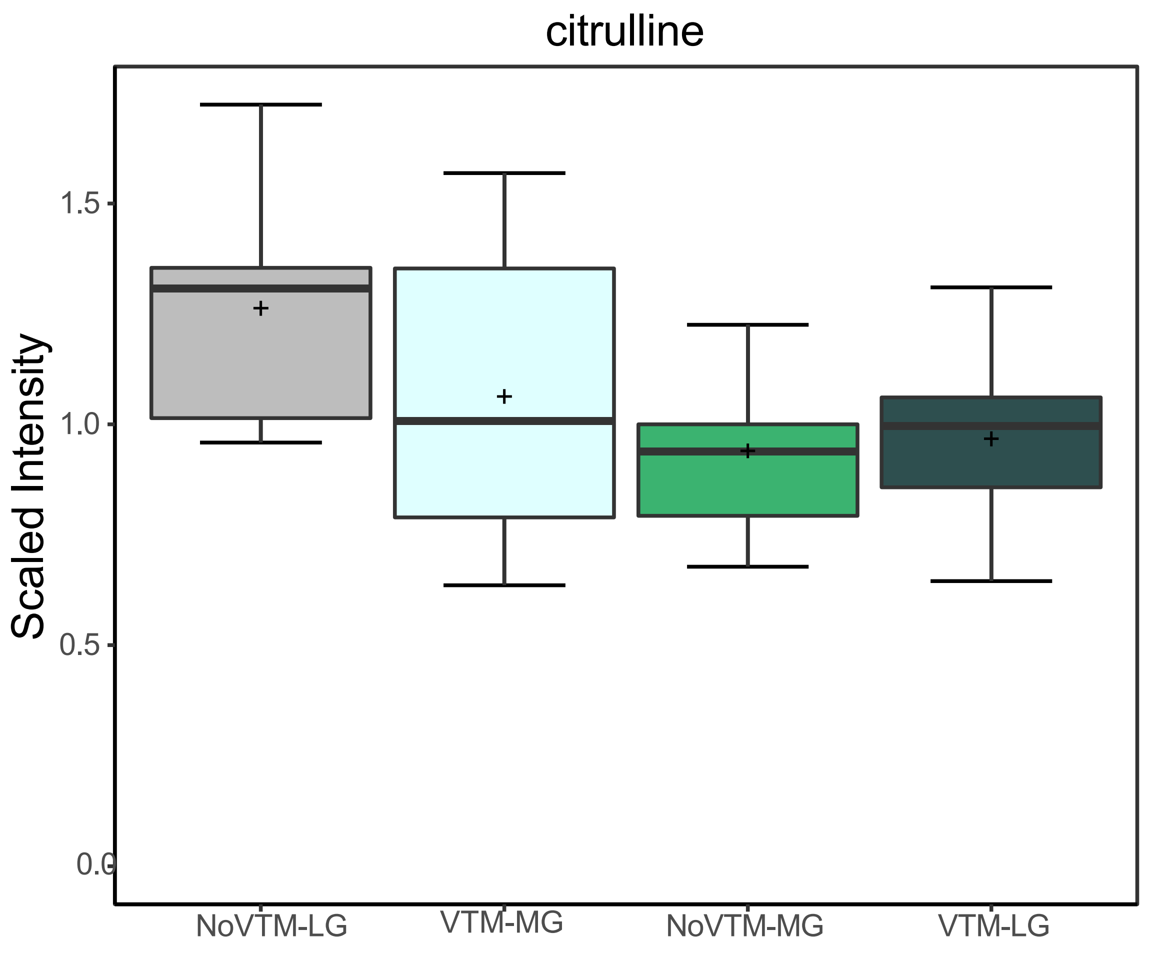
citrulline
Scaled Intensity
1.5
1.0
0.5
0.0
NoVTM-LG
VTM-MG
NoVTM-MG
VTM-LG

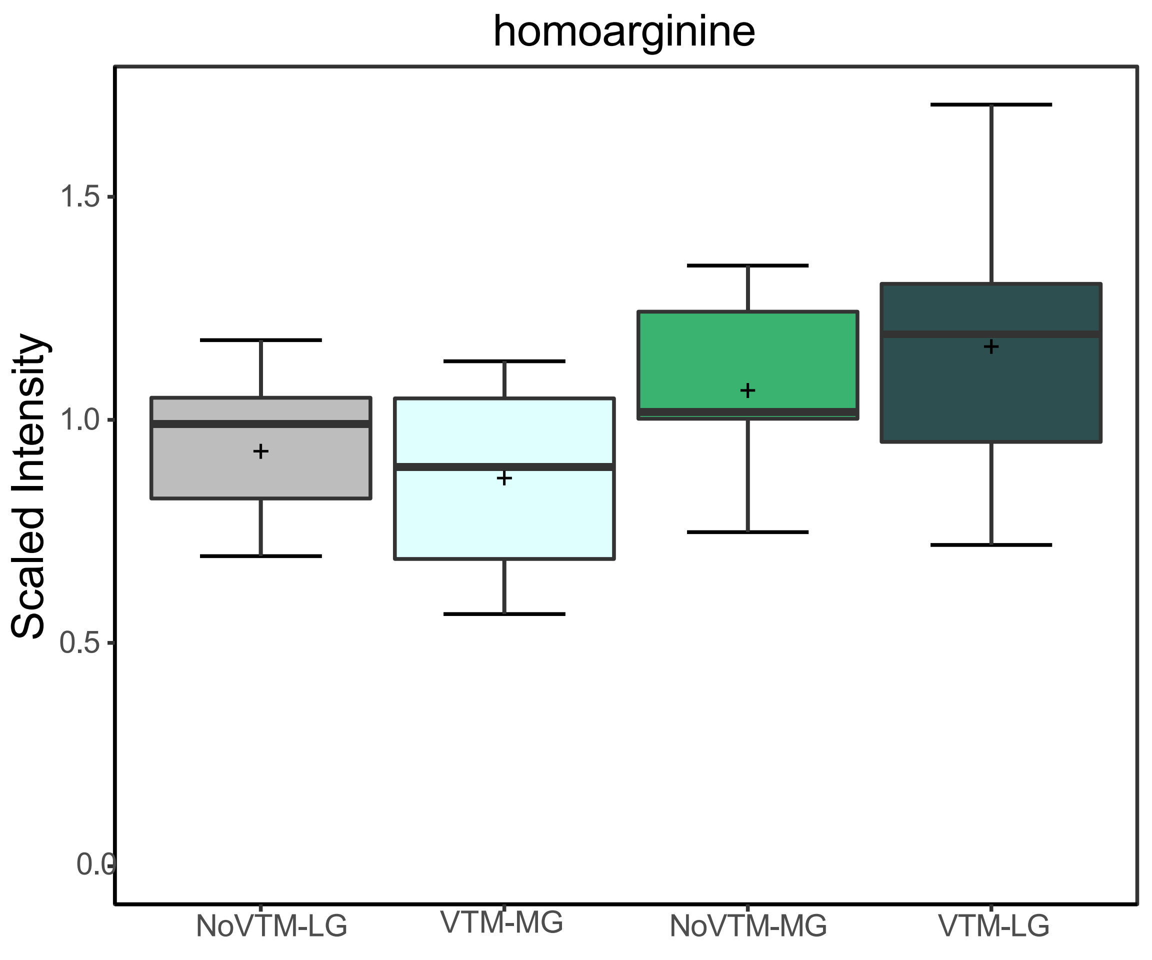
homoarginine
Scaled Intensity
1.5
1.0
0.5
0.0
NoVTM-LG
VTM-MG
NoVTM-MG
VTM-LG

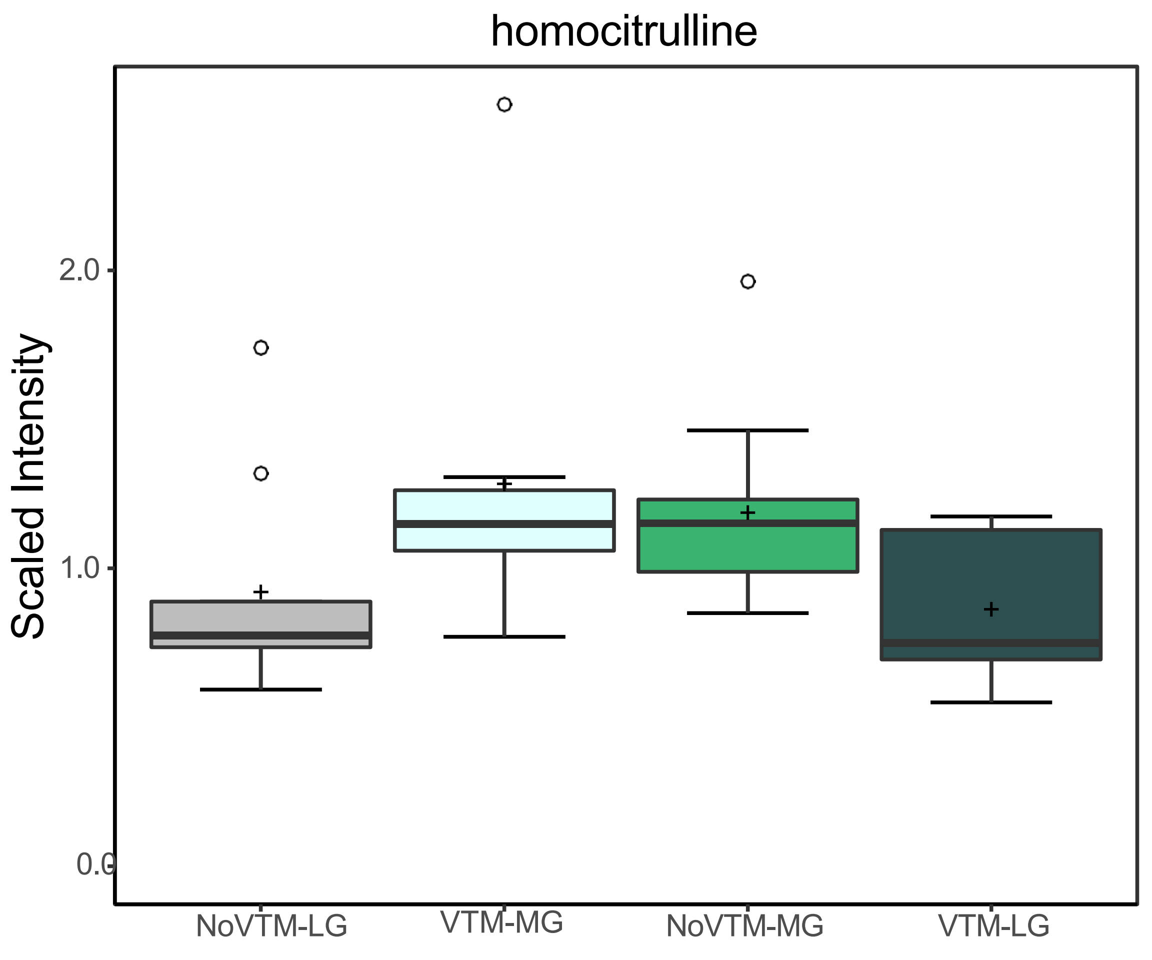
homocitrulline
Scaled Intensity
2.0
1.0
0.0
NoVTM-LG
VTM-MG
NoVTM-MG
VTM-LG

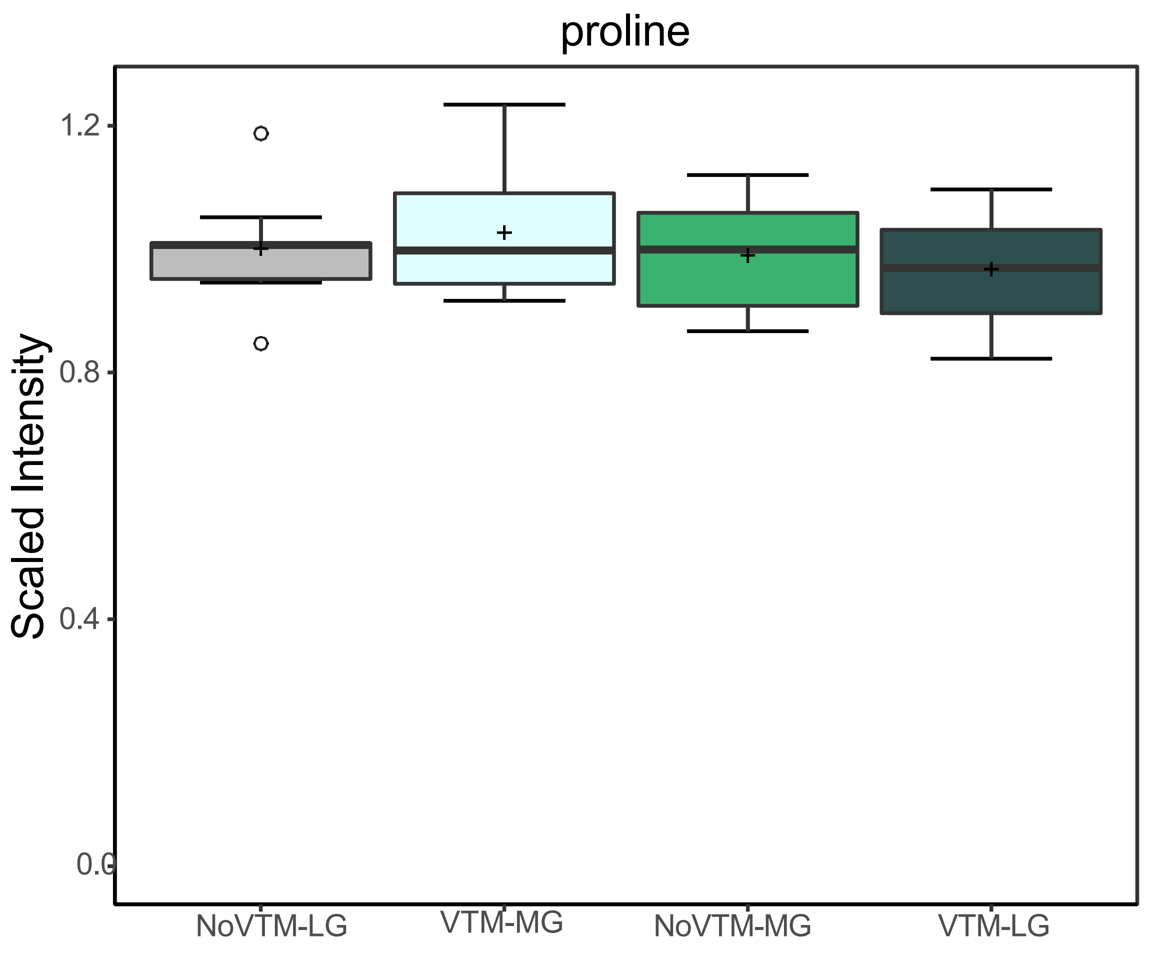

proline
Scaled Intensity
1.2
0.8
0.4
0.0
NoVTM-LG
VTM-MG
NoVTM-MG
VTM-LG

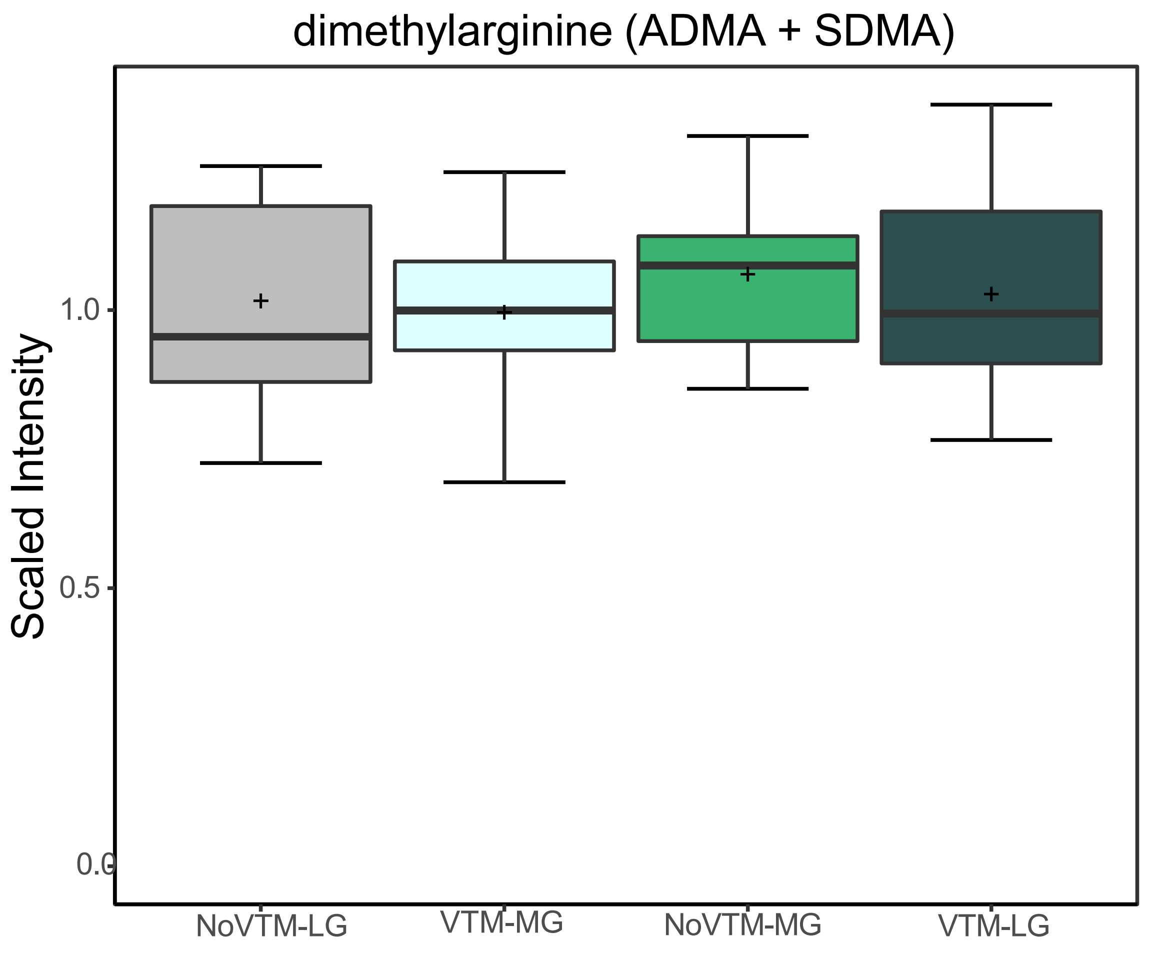

dimethylarginine (ADMA + SDMA)
Scaled Intensity
1.0
0.5
0.0
NoVTM-LG
VTM-MG
NoVTM-MG
VTM-LG

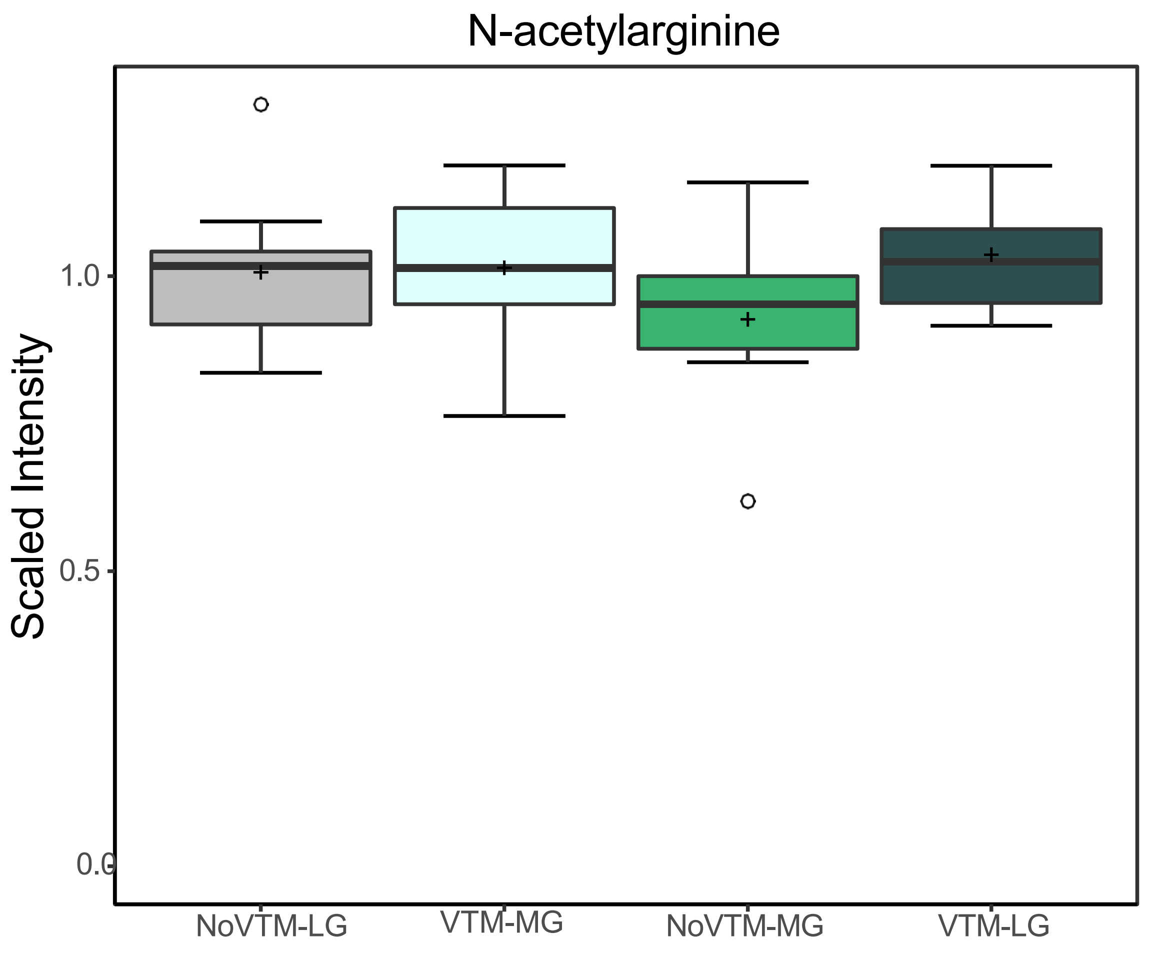
N-acetylarginine
Scaled Intensity
1.0
0.5
0.0
NoVTM-LG
VTM-MG
NoVTM-MG
VTM-LG

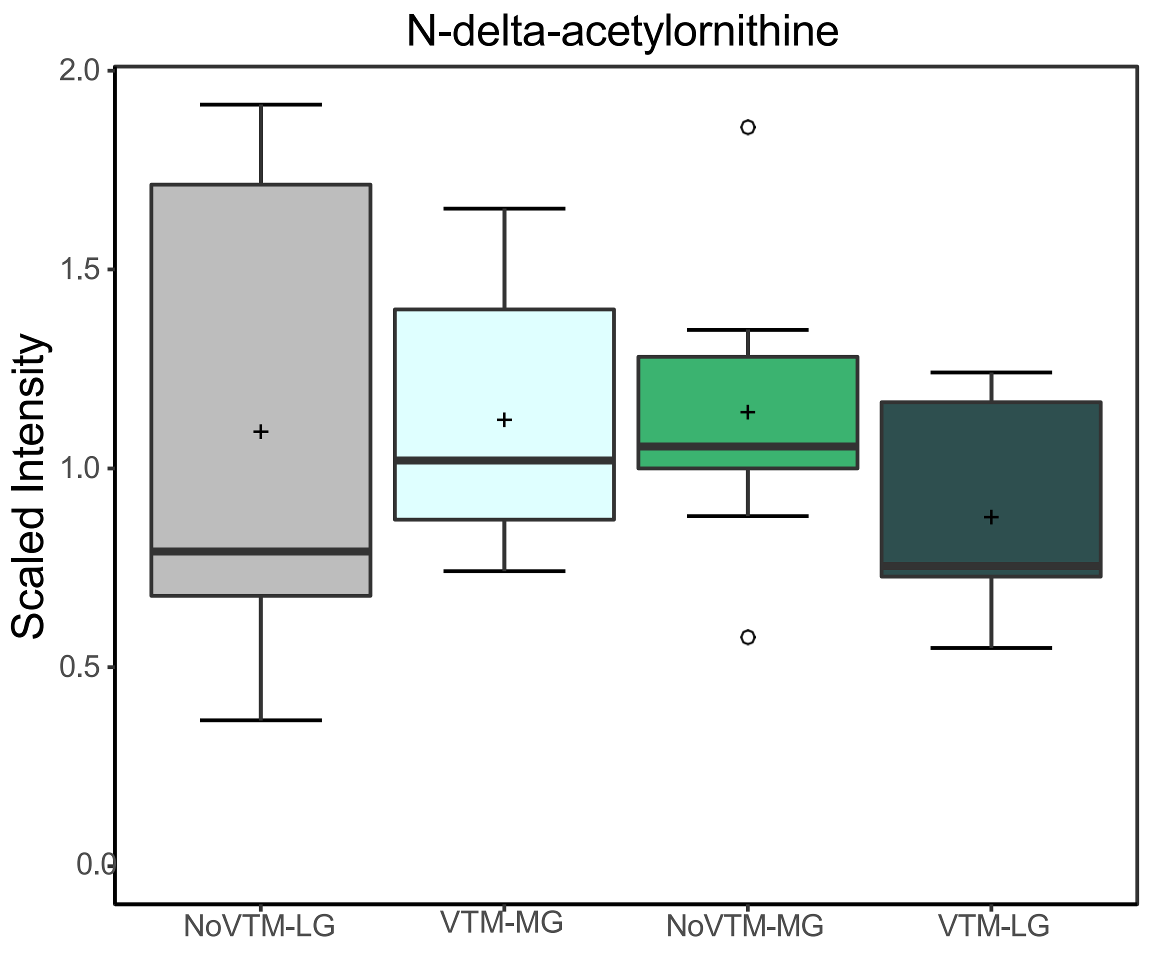

N-delta-acetylornithine
Scaled Intensity
2.0
1.5
1.0
0.5
0.0
NoVTM-LG
VTM-MG
NoVTM-MG
VTM-LG

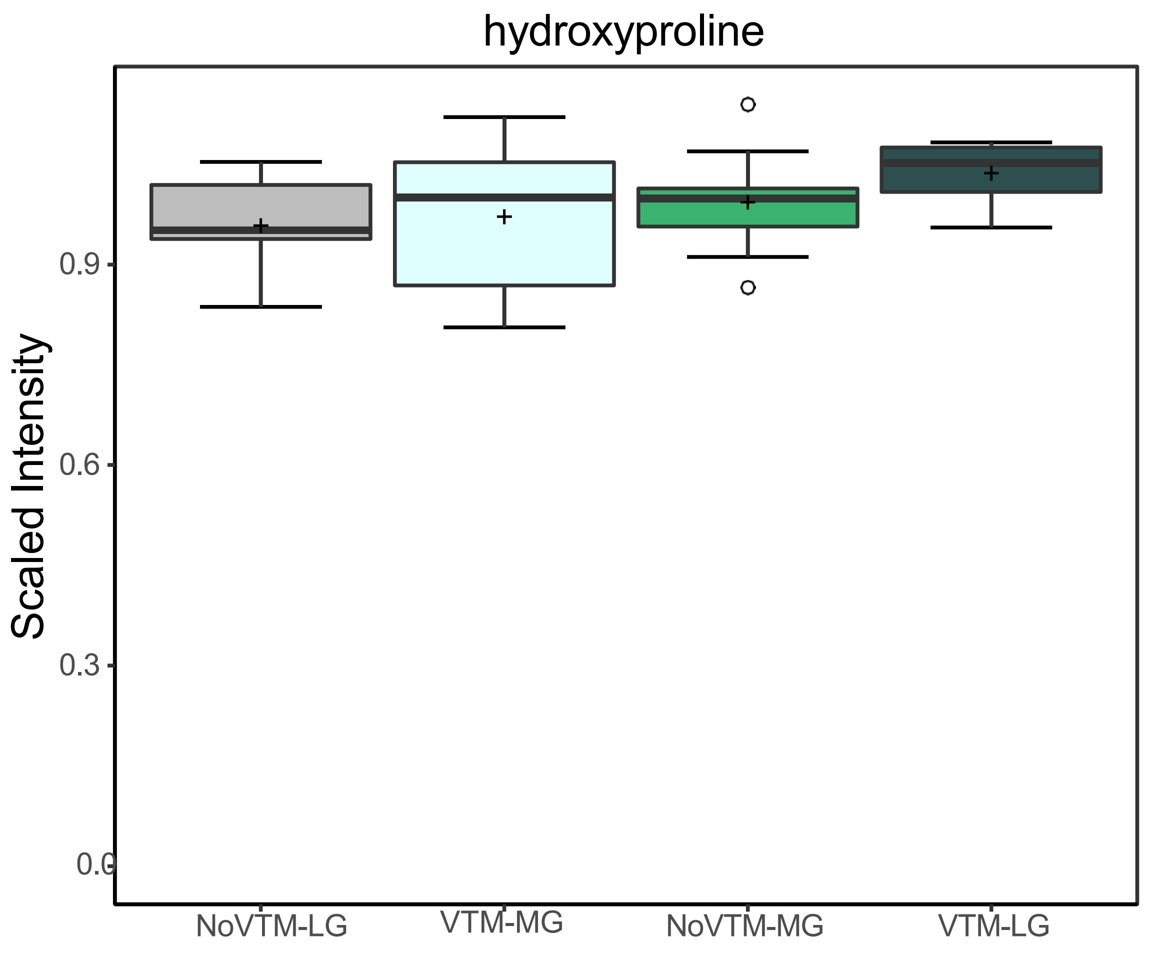
hydroxyproline
Scaled Intensity
0.9
0.6
0.3
0.0
NoVTM-LG
VTM-MG
NoVTM-MG
VTM-LG

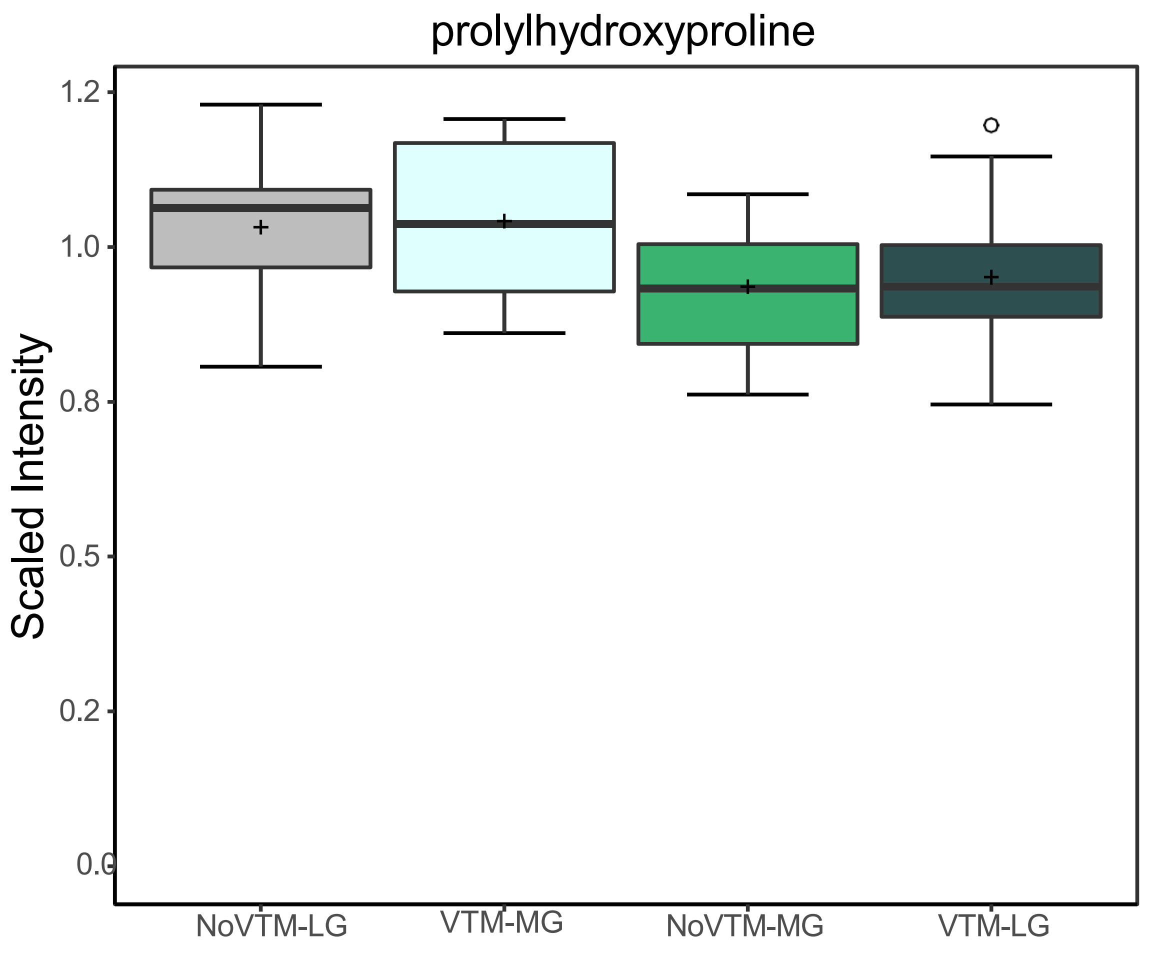
prolylhydroxyproline
Scaled Intensity
1.2
1.0
0.8
0.5
0.2
0.0
NoVTM-LG
VTM-MG
NoVTM-MG
VTM-LG

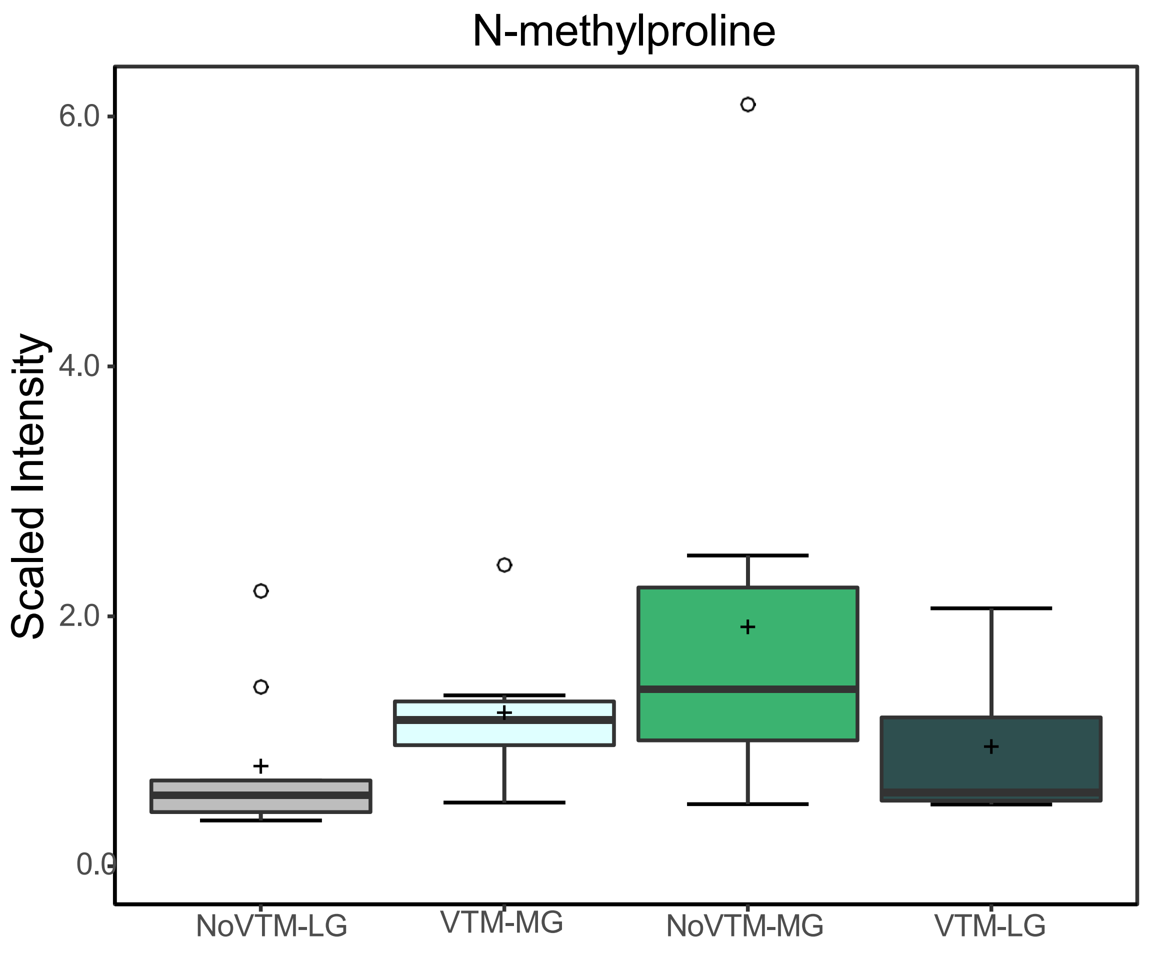
N-methylproline
Scaled Intensity
6.0
4.0
2.0
0.0
NoVTM-LG
VTM-MG
NoVTM-MG
VTM-LG

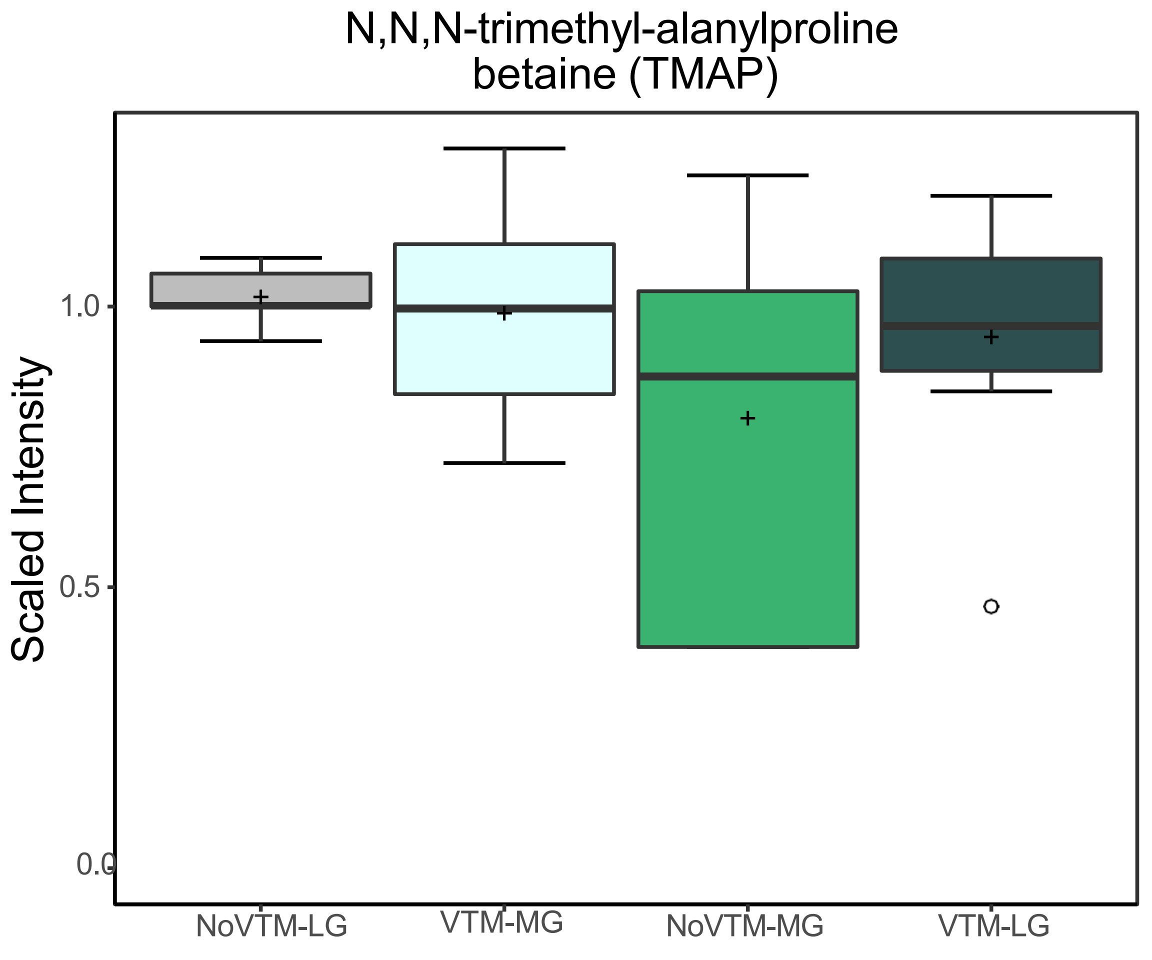

N,N,N-trimethyl-alanylproline
betaine (TMAP)
Scaled Intensity
1.0
0.5
0.0
NoVTM-LG
VTM-MG
NoVTM-MG
VTM-LG

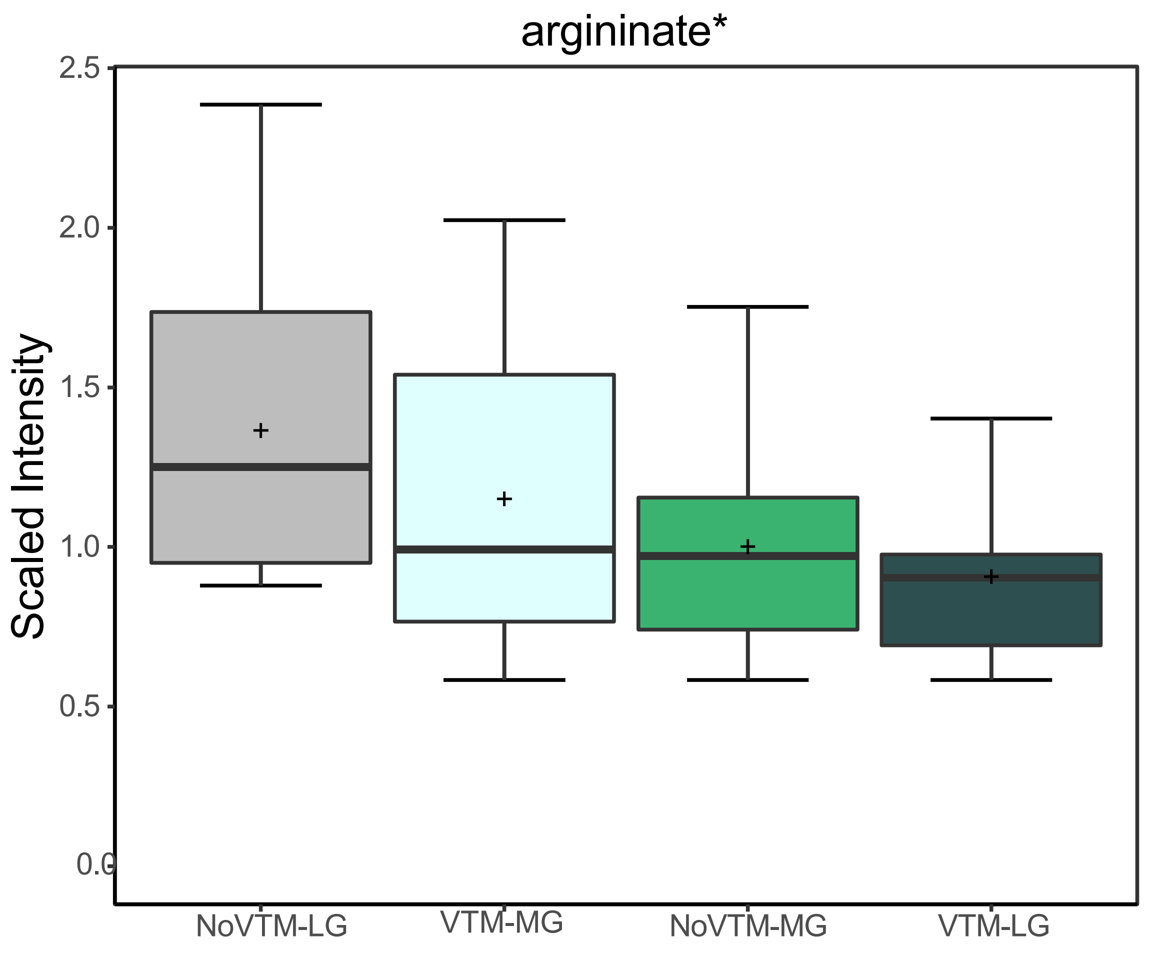
arginate*
Scaled Intensity
2.5
2.0
1.5
1.0
0.5
0.0
NoVTM-LG
VTM-MG
NoVTM-MG
VTM-LG

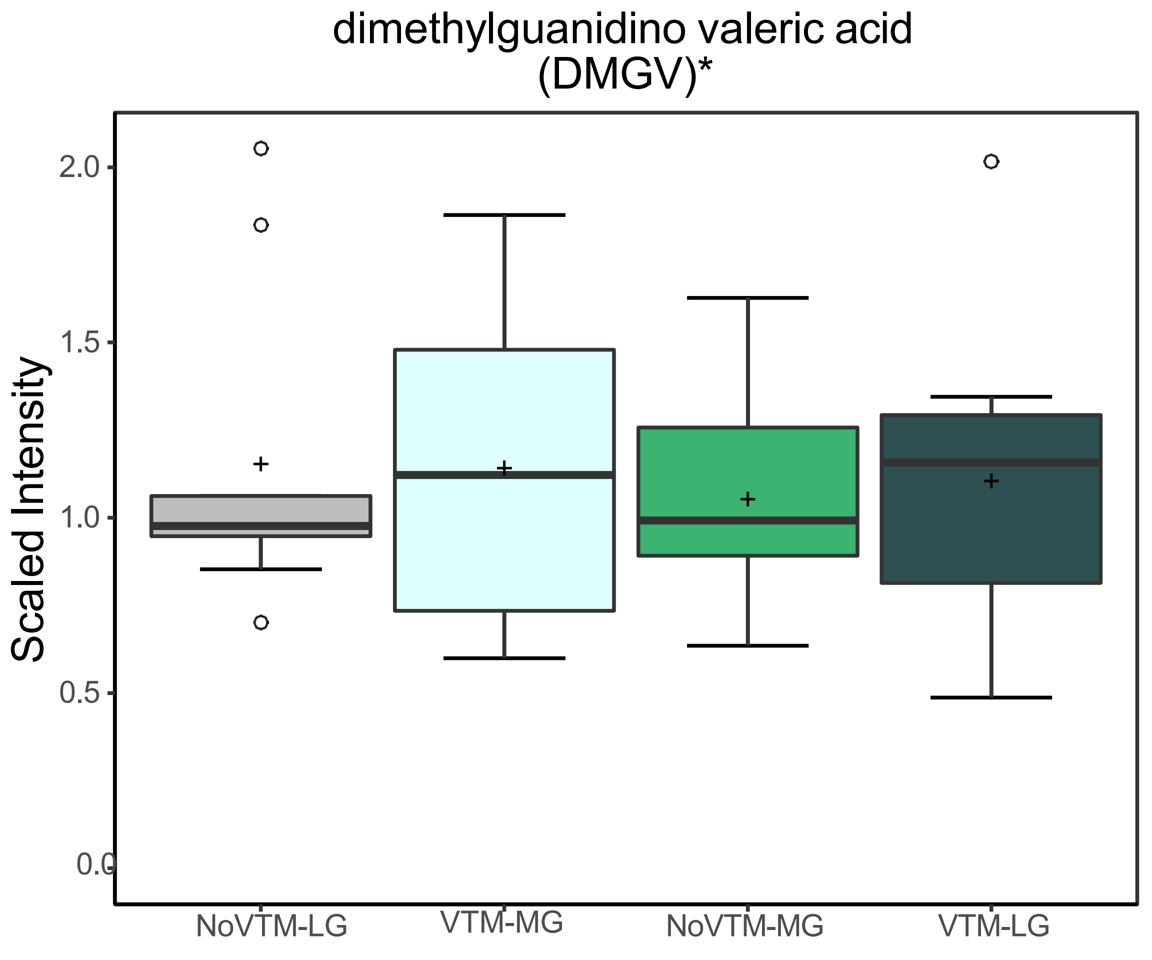

dimethylguanidino valeric acid
(DMGV)*
Scaled Intensity
2.0
1.5
1.0
0.5
0.0
NoVTM-LG
VTM-MG
NoVTM-MG
VTM-LG

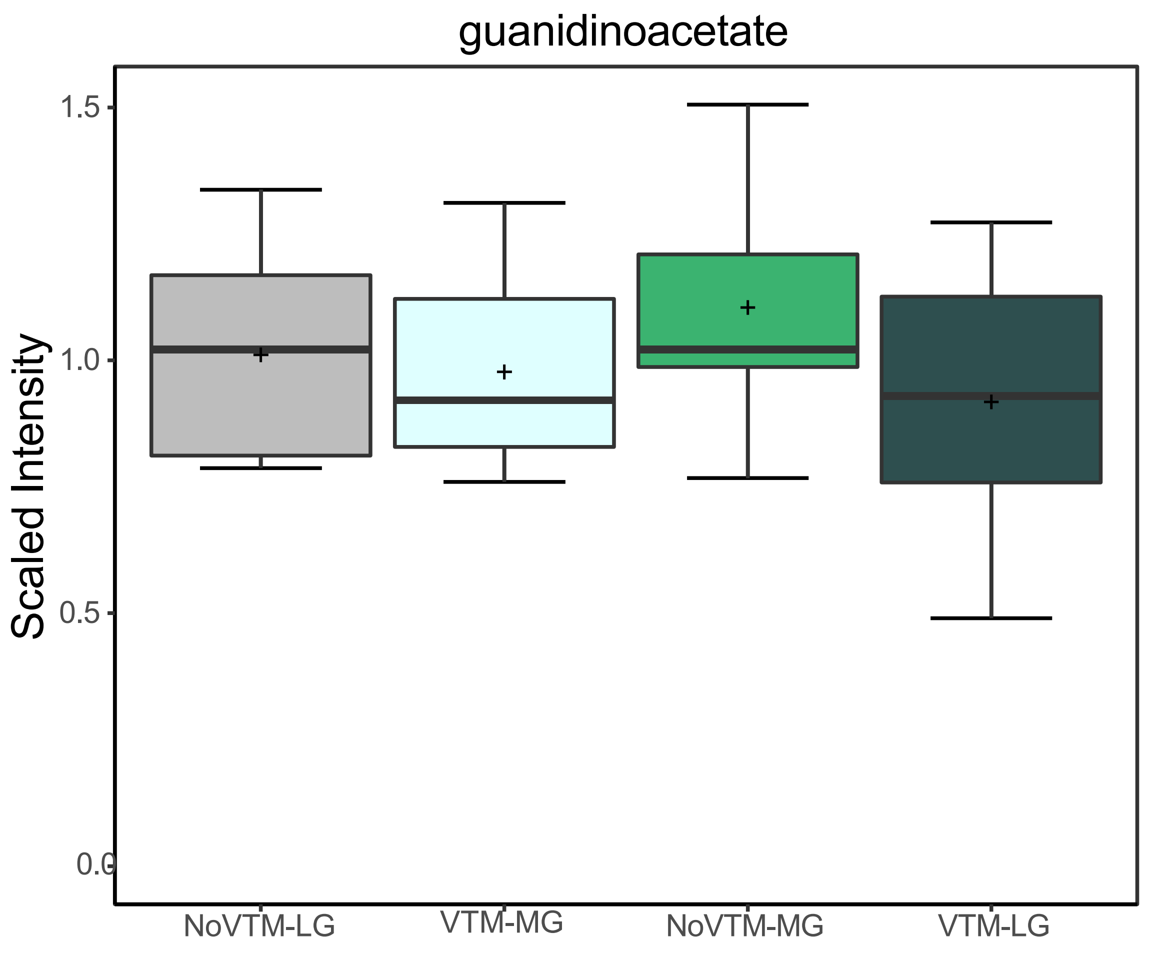
guanidinoacetate
Scaled Intensity
1.5
1.0
0.5
0.0
NoVTM-LG
VTM-MG
NoVTM-MG
VTM-LG

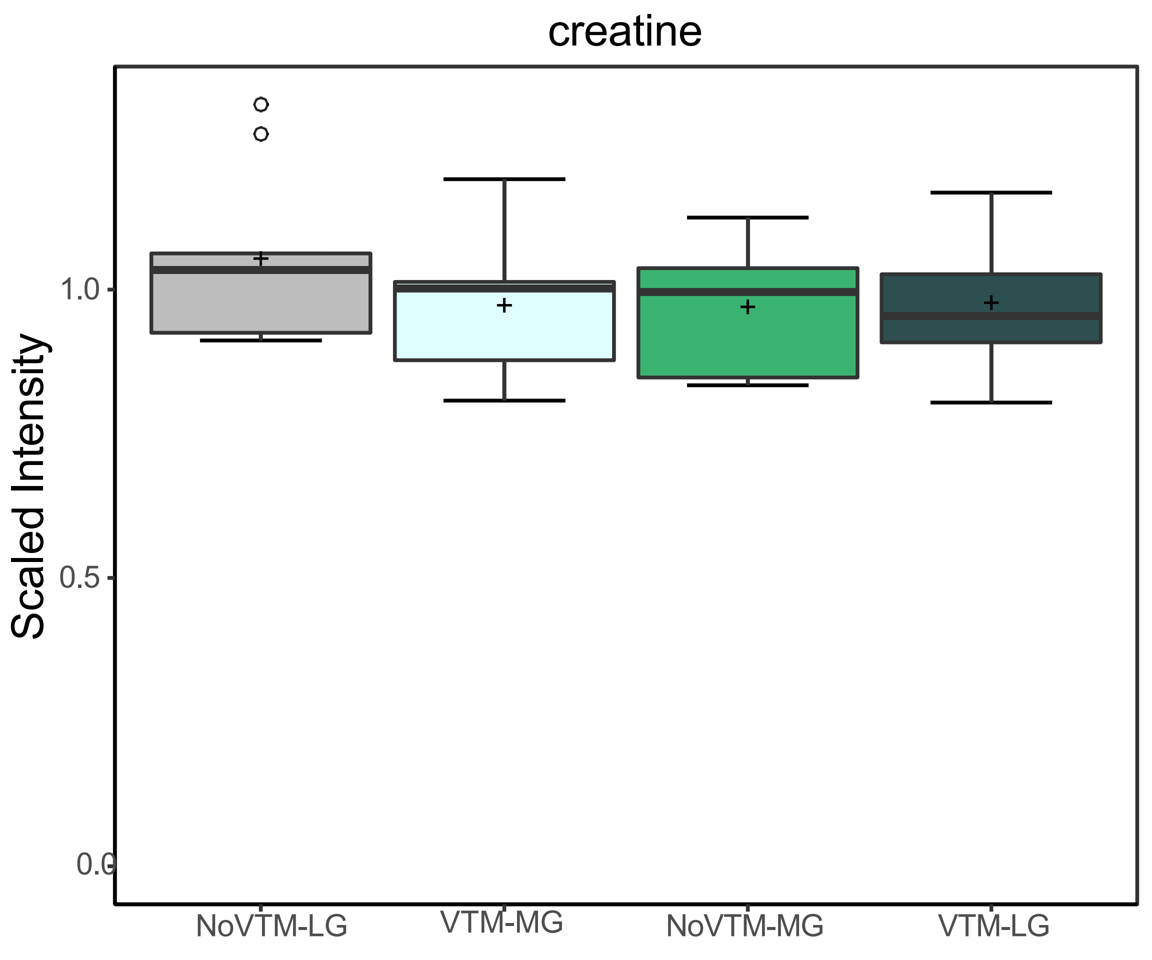
creatine
Scaled Intensity
1.0
0.5
0.0
NoVTM-LG
VTM-MG
NoVTM-MG
VTM-LG

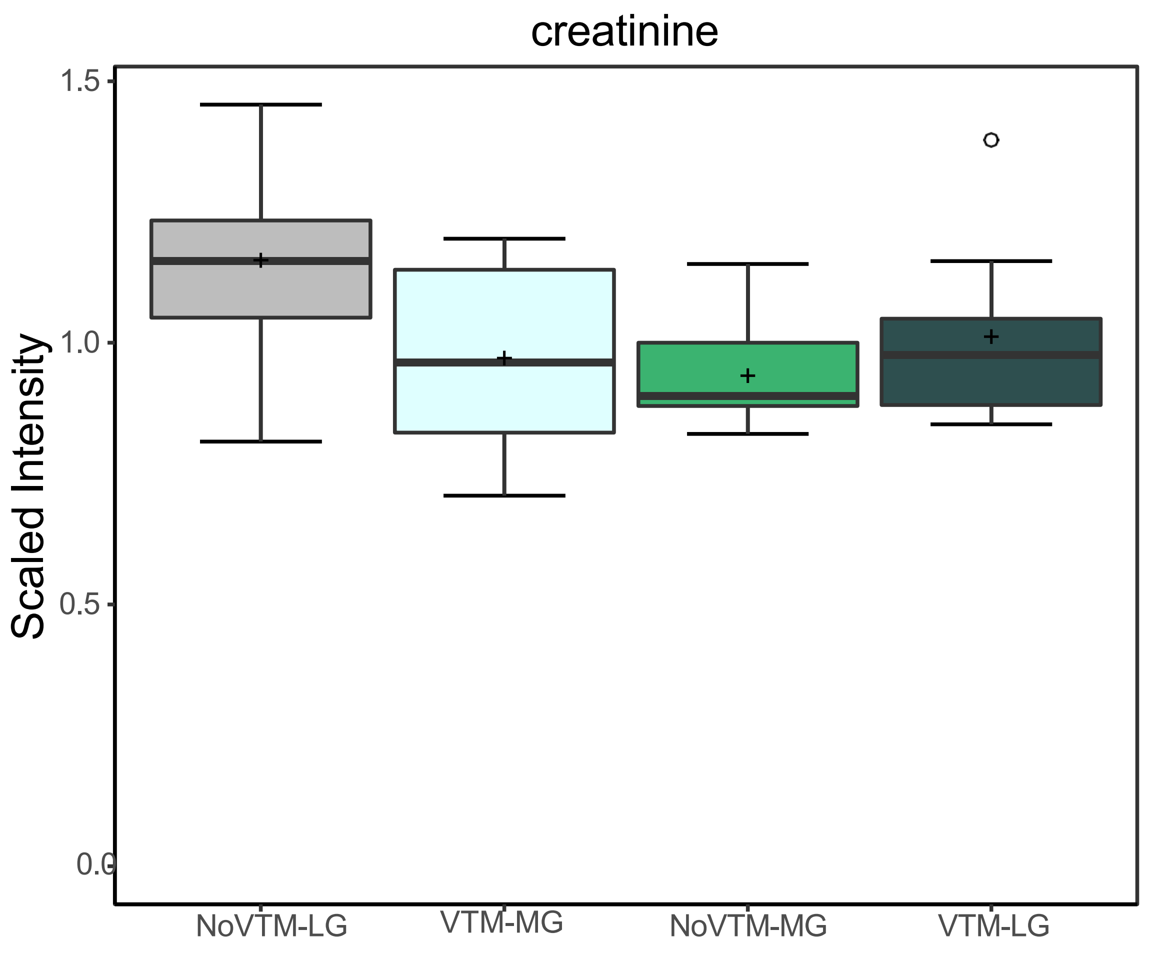
creatinine
Scaled Intensity
1.5
1.0
0.5
0.0
NoVTM-LG
VTM-MG
NoVTM-MG
VTM-LG

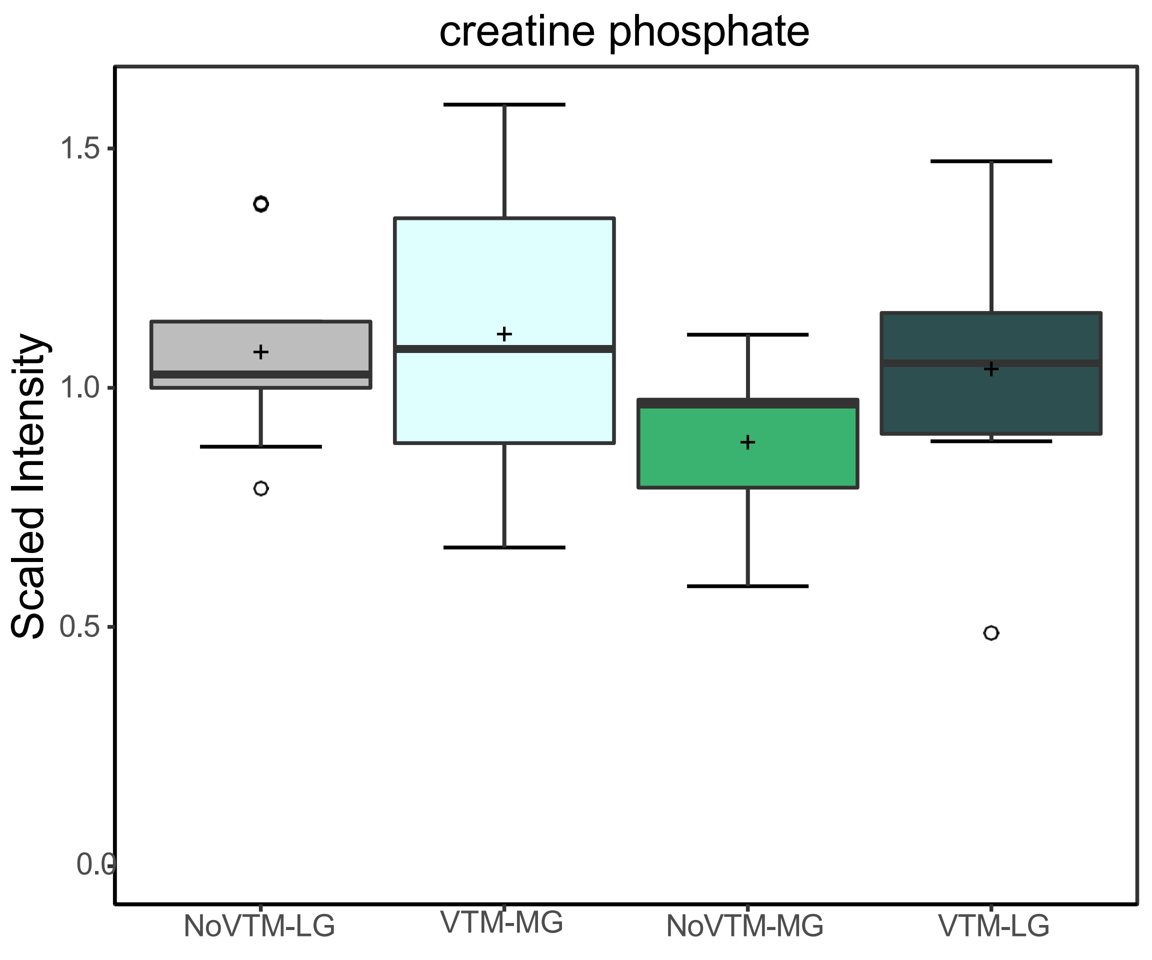
creatine phosphate
Scaled Intensity
1.5
1.0
0.5
0.0
NoVTM-LG
VTM-MG
NoVTM-MG
VTM-LG

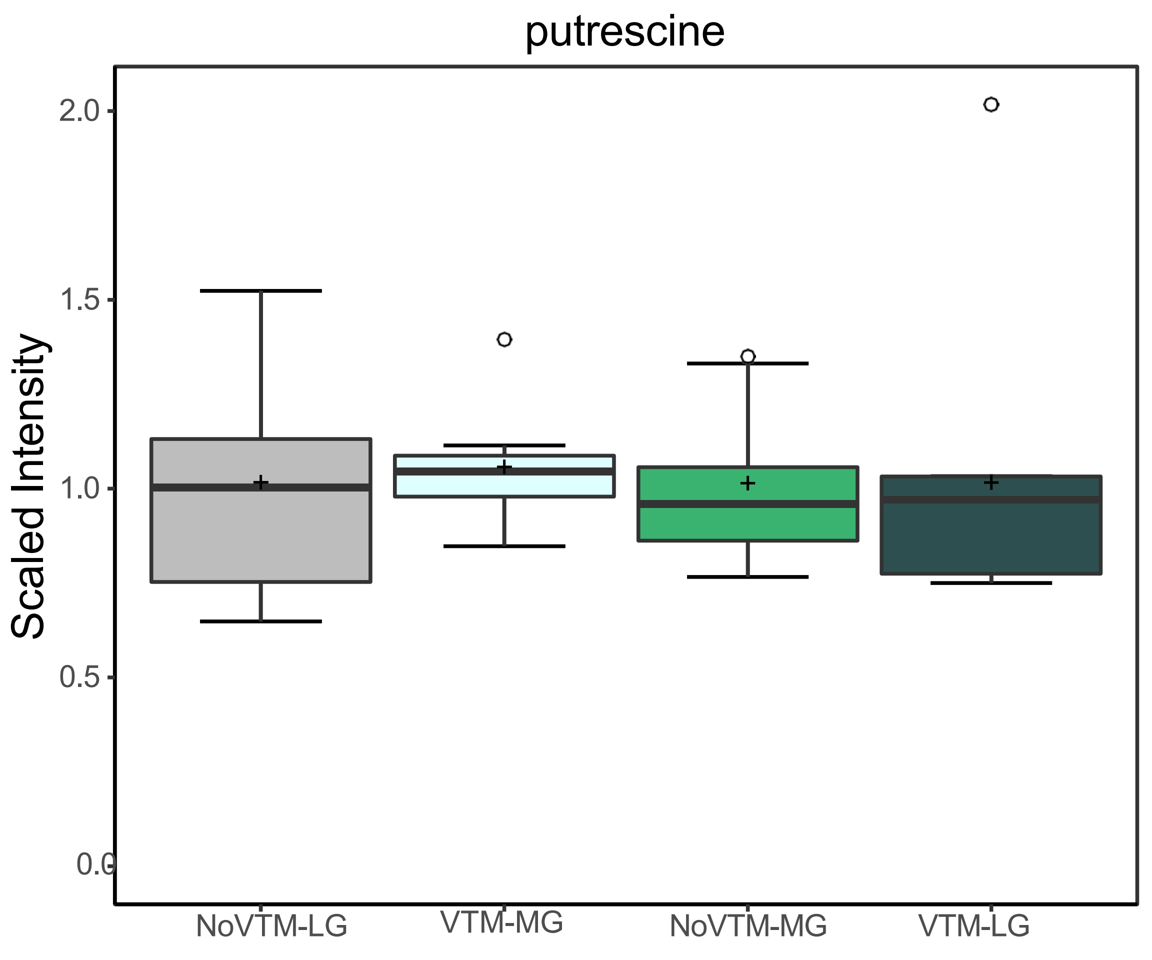

putrescine
Scaled Intensity
2.0
1.5
1.0
0.5
0.0
NoVTM-LG
VTM-MG
NoVTM-MG
VTM-LG

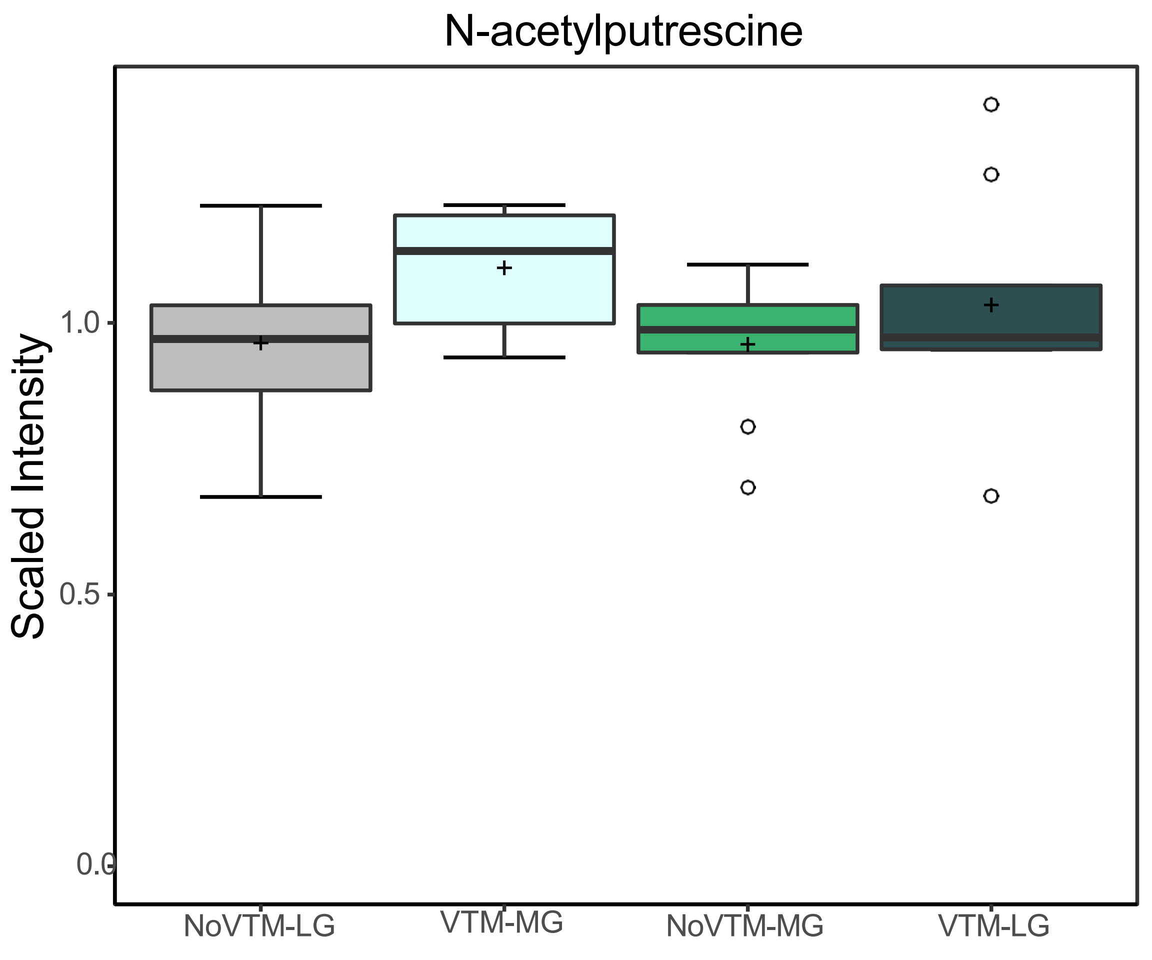
N-acetylputrescine
Scaled Intensity
1.0
0.5
0.0
NoVTM-LG
VTM-MG
NoVTM-MG
VTM-LG

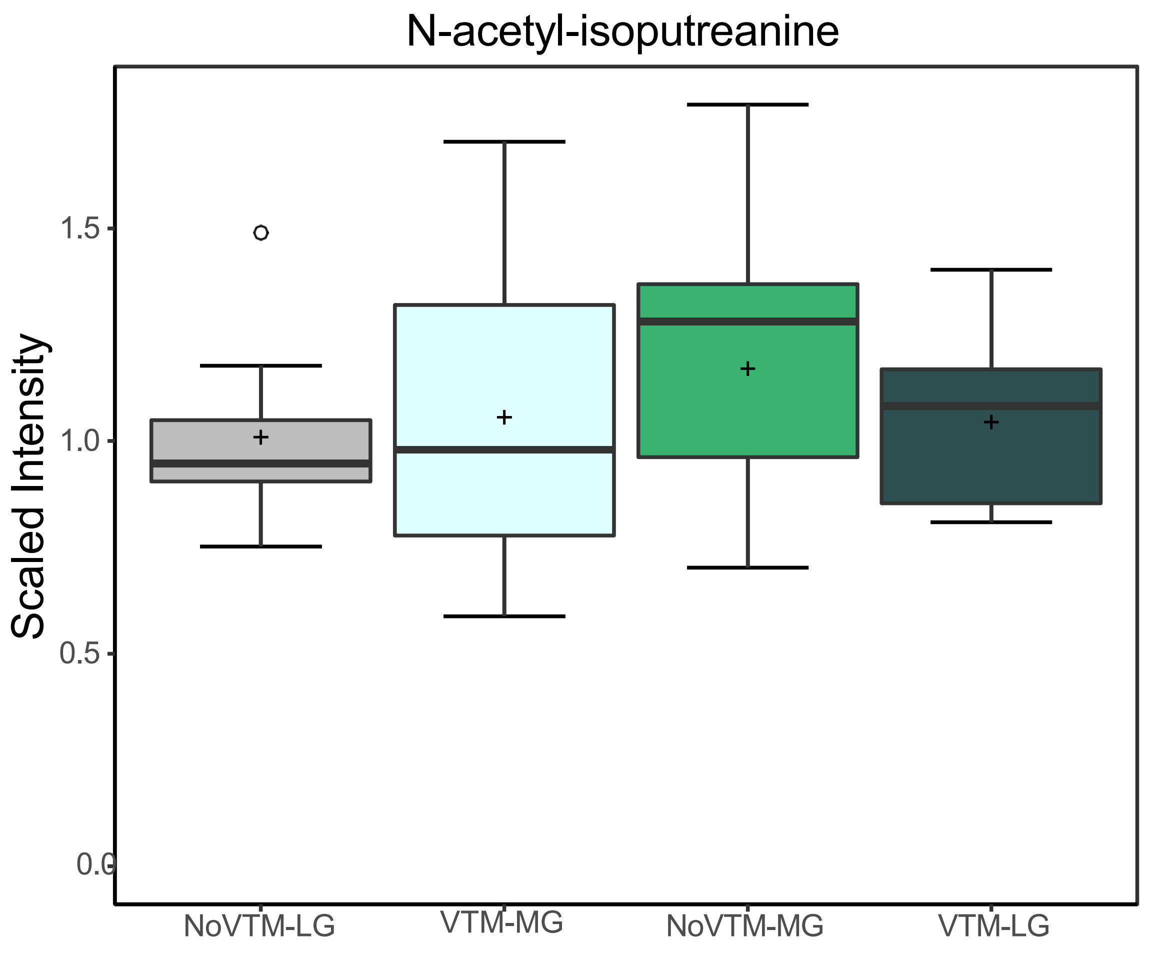
N-acetyl-isoputreanine
Scaled Intensity
1.5
1.0
0.5
0.0
NoVTM-LG
VTM-MG
NoVTM-MG
VTM-LG

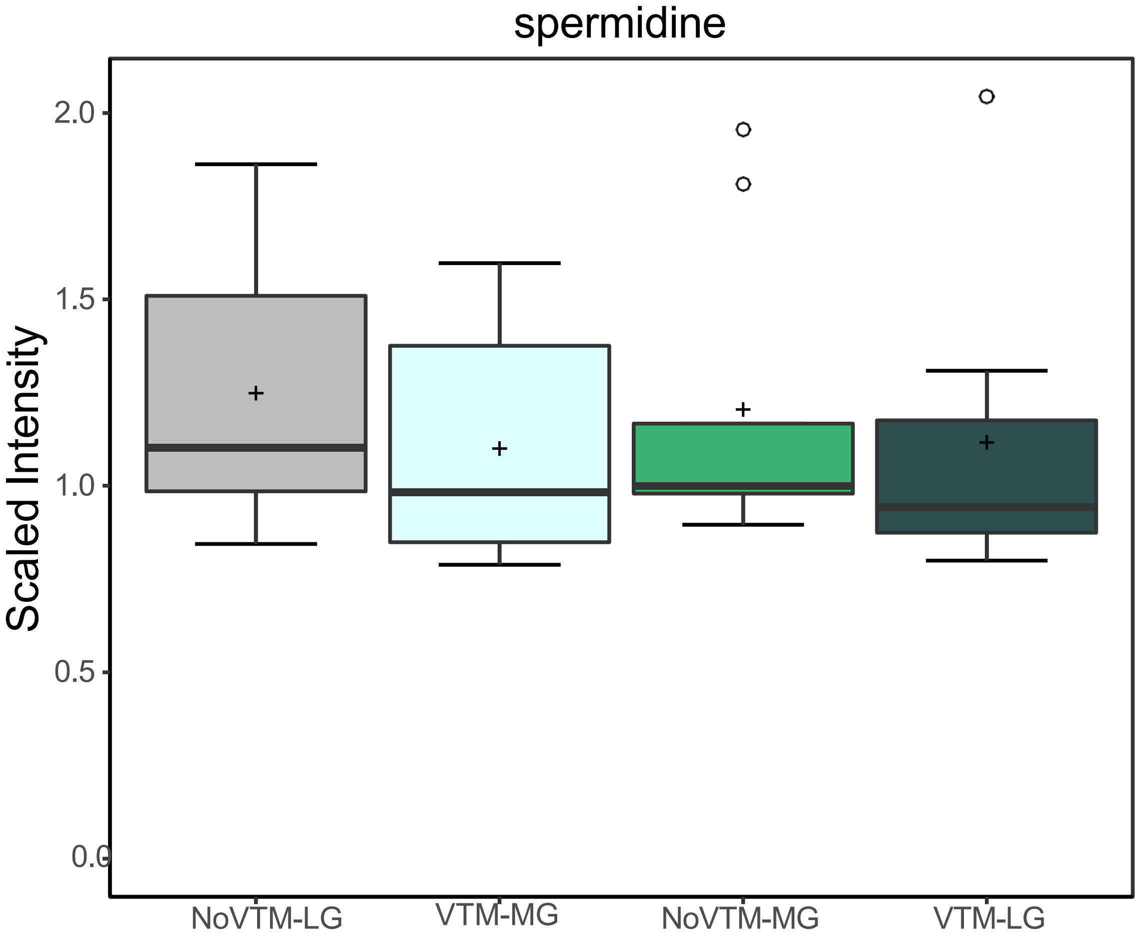

spermidine
Scaled Intensity
2.0
1.5
1.0
0.5
0.0
NoVTM-LG
VTM-MG
NoVTM-MG
VTM-LG

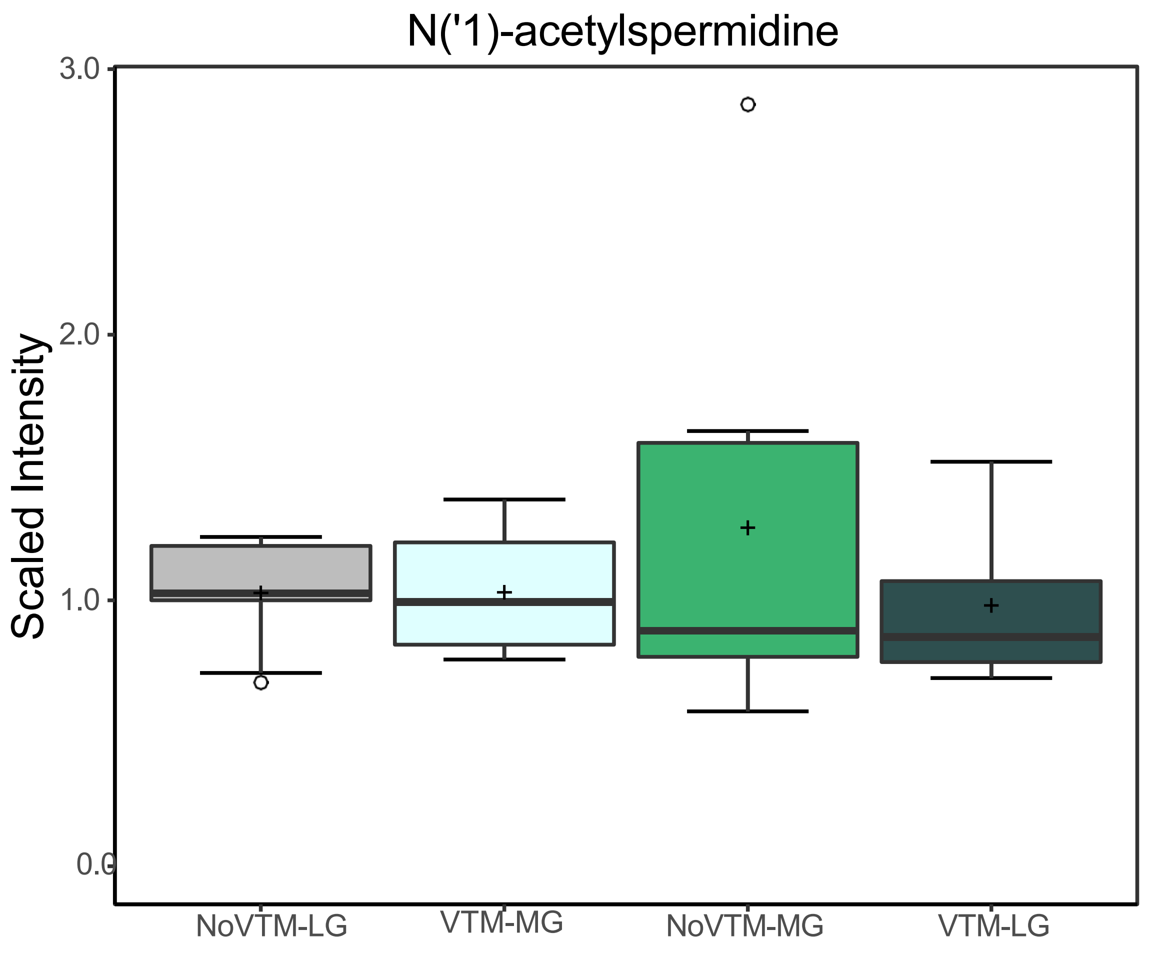

N('1)-acetylspermidine
Scaled Intensity
3.0
2.0
1.0
0.0
NoVTM-LG
VTM-MG
NoVTM-MG
VTM-LG

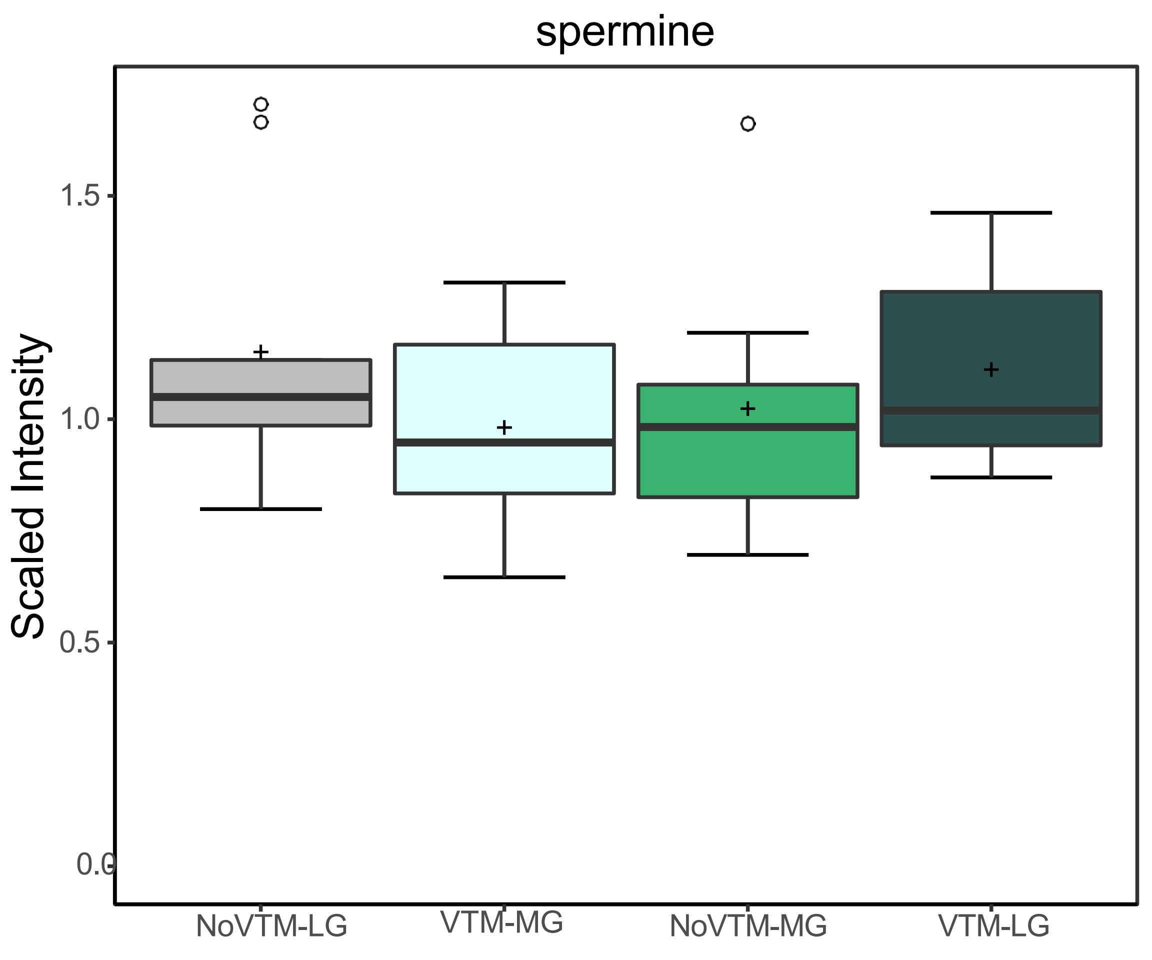
spermine
Scaled Intensity
1.5
1.0
0.5
0.0
NoVTM-LG
VTM-MG
NoVTM-MG
VTM-LG

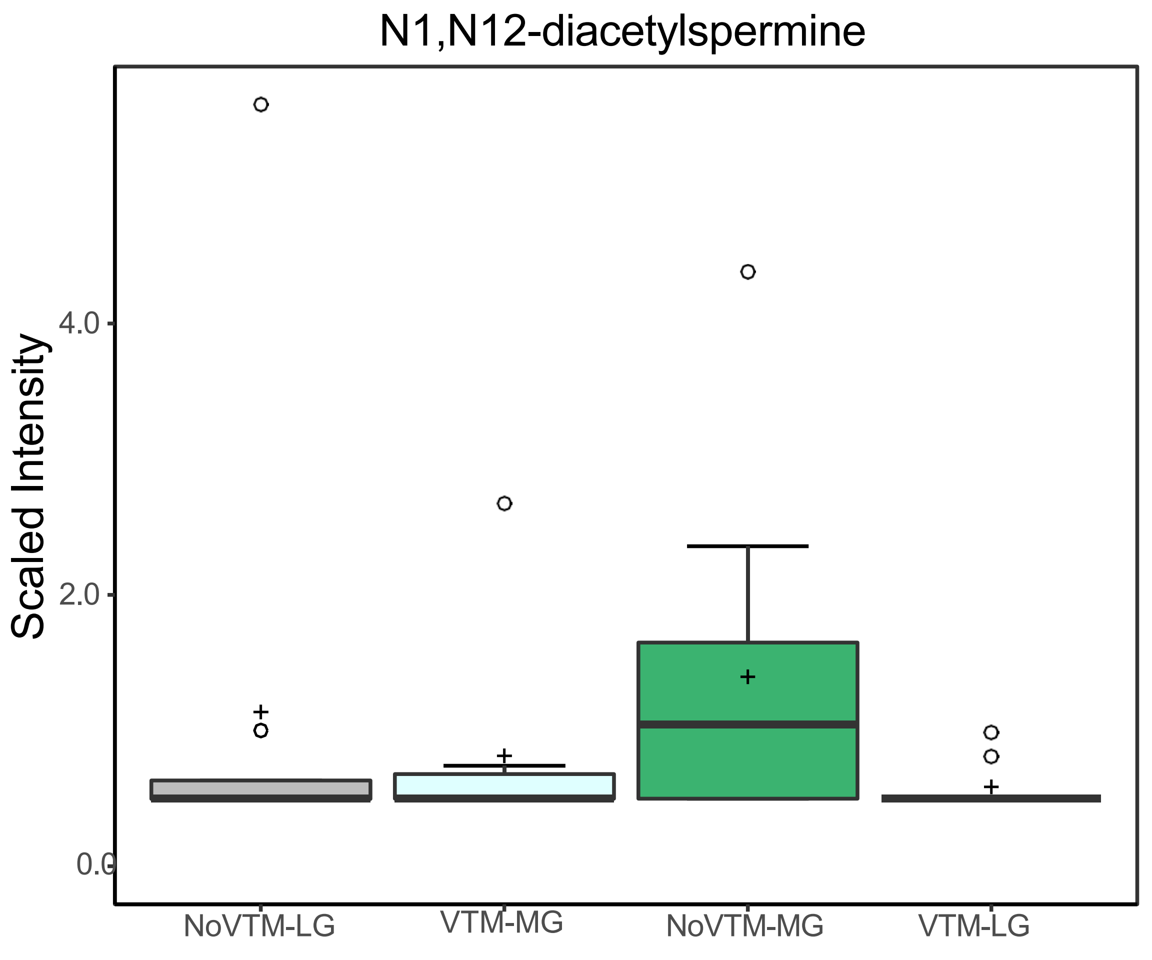

N1,N12-diacetylspermine
Scaled Intensity
4.0
2.0
0.0
NoVTM-LG
VTM-MG
NoVTM-MG
VTM-LG

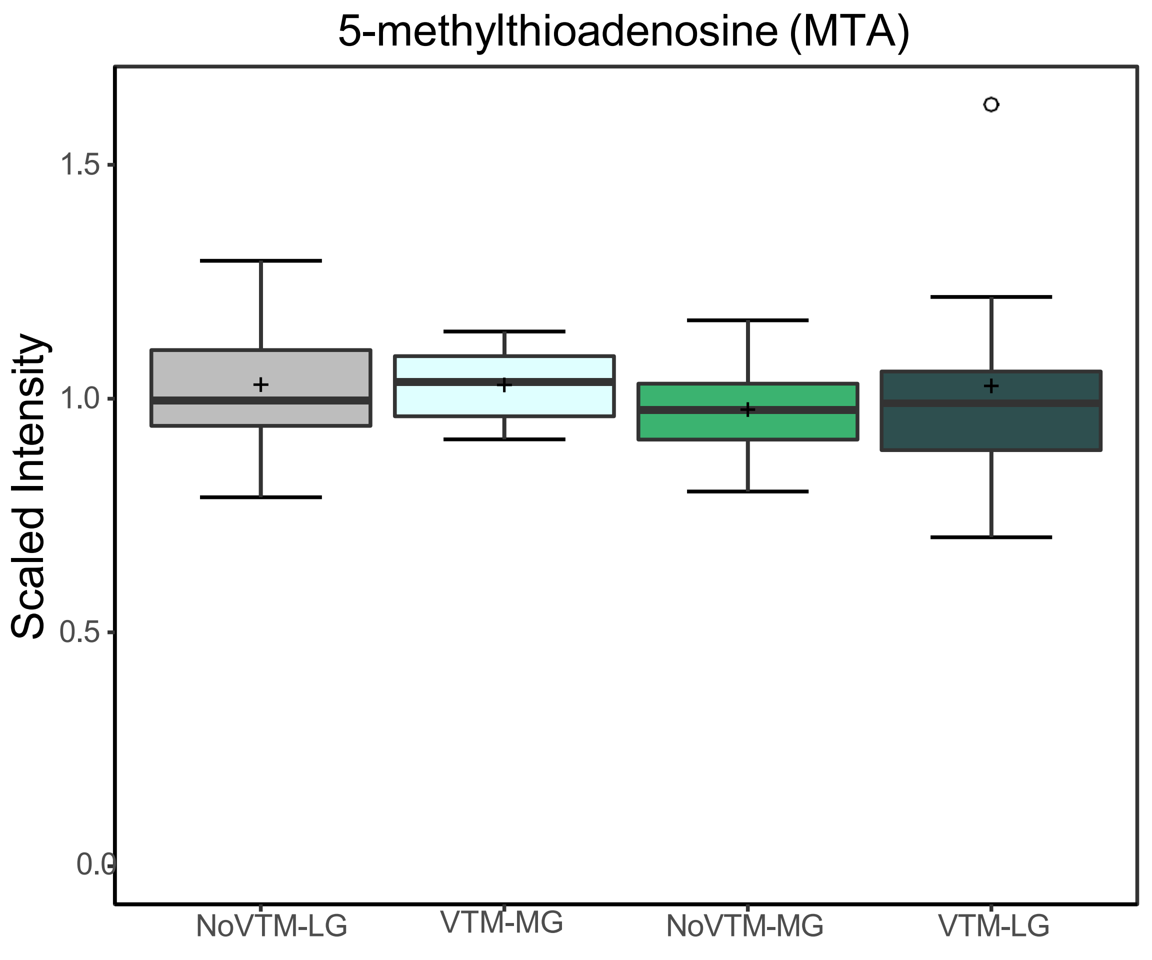
5-methylthioadenosine (MTA)
Scaled Intensity
1.5
1.0
0.5
0.0
NoVTM-LG
VTM-MG
NoVTM-MG
VTM-LG

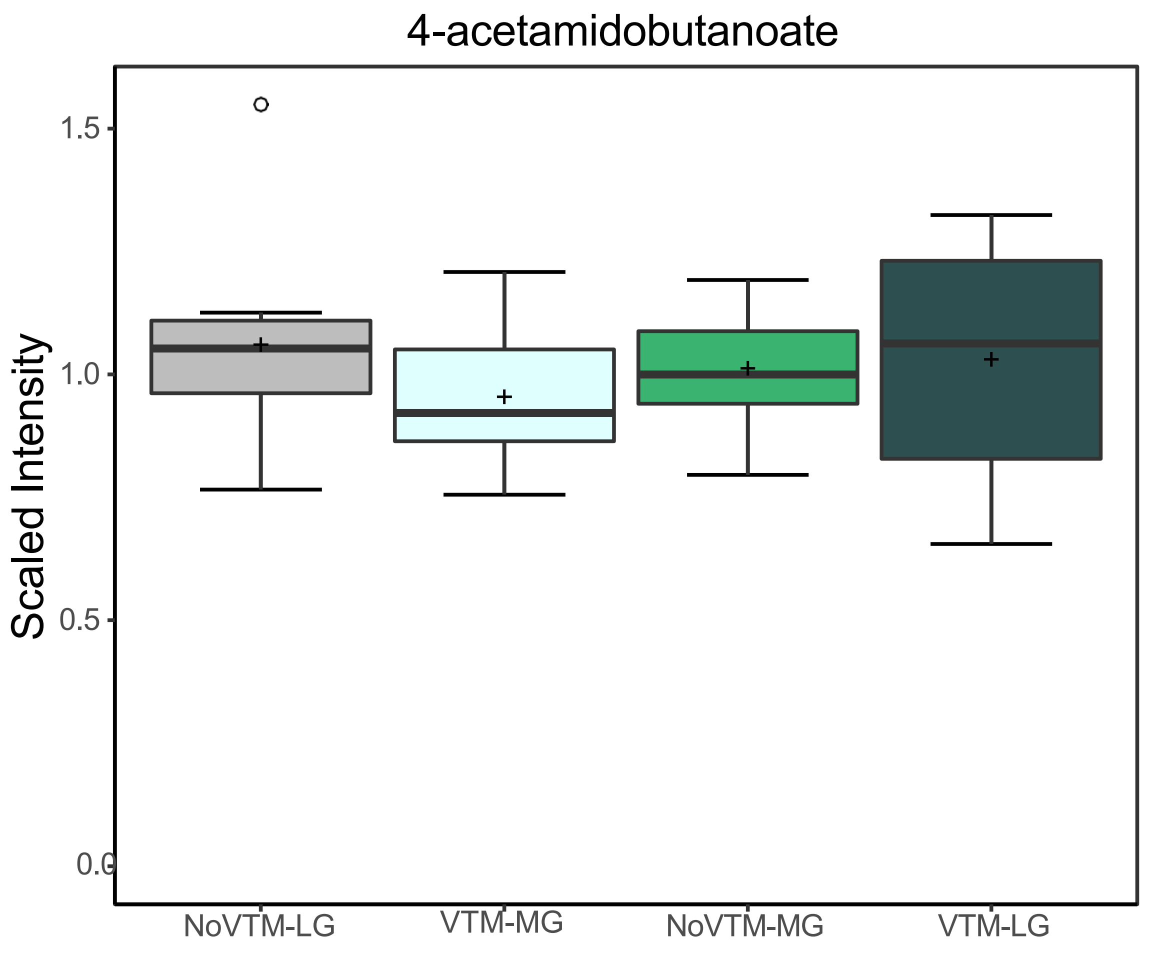

4-acetamidobutanoate
Scaled Intensity
1.5
1.0
0.5
0.0
NoVTM-LG
VTM-MG
NoVTM-MG
VTM-LG

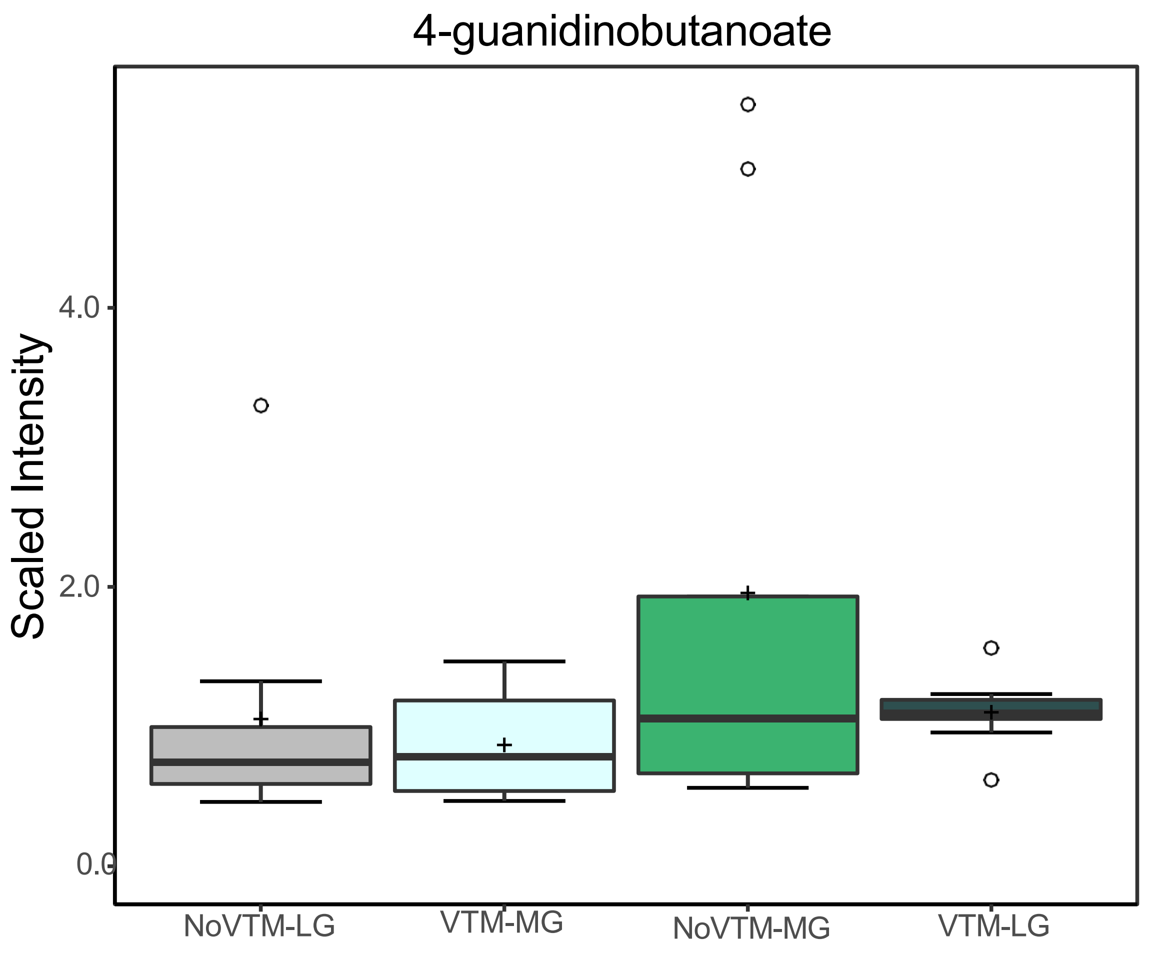

4-guanidinobutanoate
Scaled Intensity
4.0
2.0
0.0
NoVTM-LG
VTM-MG
NoVTM-MG
VTM-LG

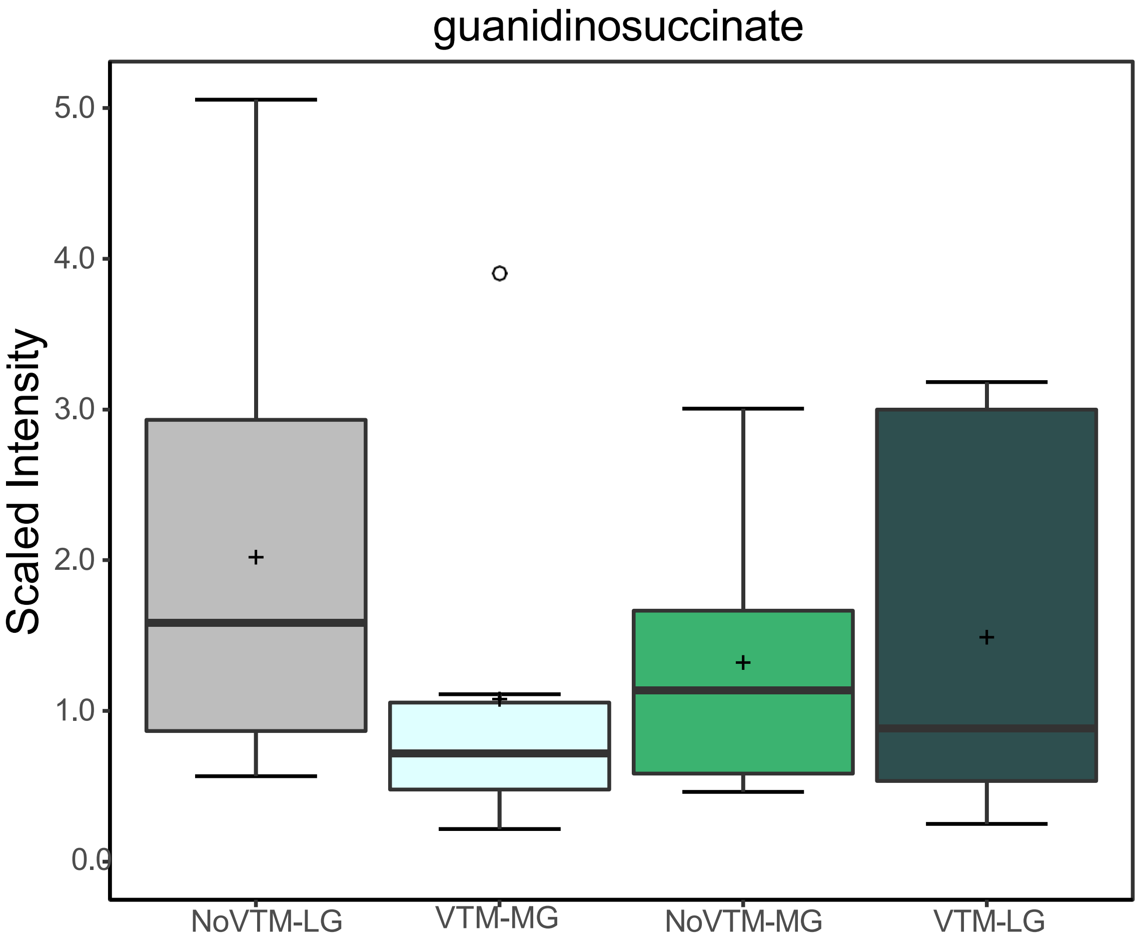
guanidinosuccinate
Scaled Intensity
5.0
4.0
3.0
2.0
1.0
0.0
NoVTM-LG
VTM-MG
NoVTM-MG
VTM-LG

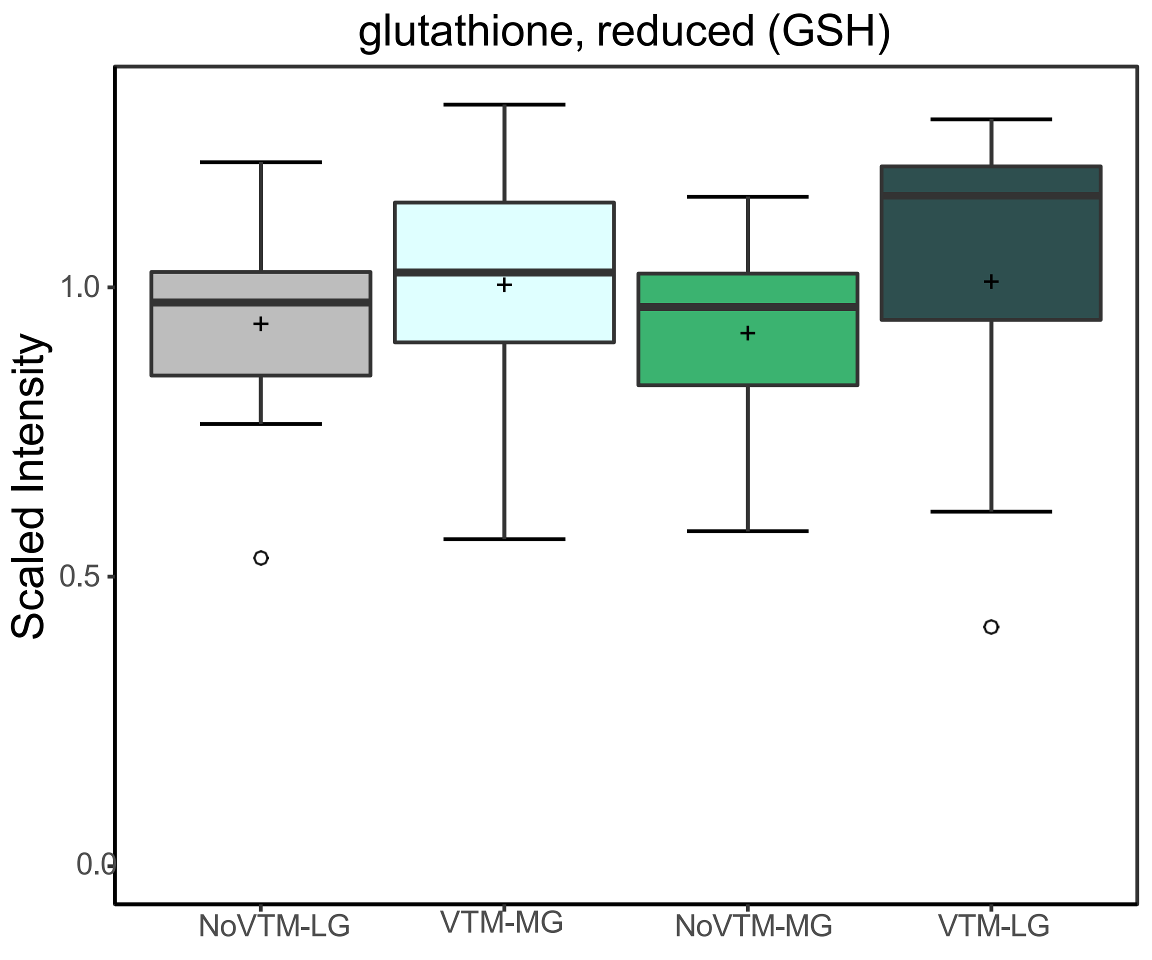
glutathione, reduced (GSH)
Scaled Intensity
1.0
0.5
0.0
NoVTM-LG
VTM-MG
NoVTM-MG
VTM-LG

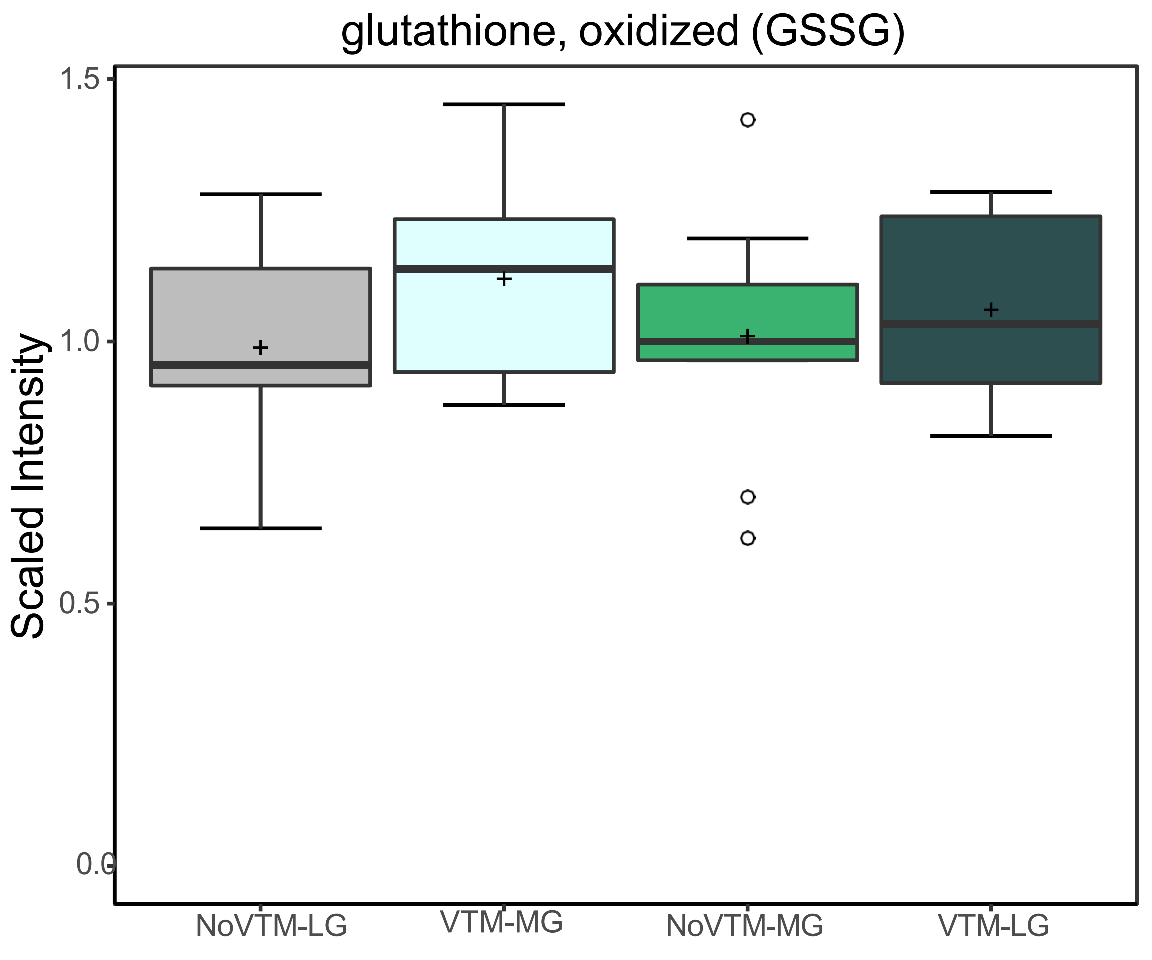

glutathione, oxidized (GSSG)
Scaled Intensity
1.5
1.0
0.5
0.0
NoVTM-LG
VTM-MG
NoVTM-MG
VTM-LG

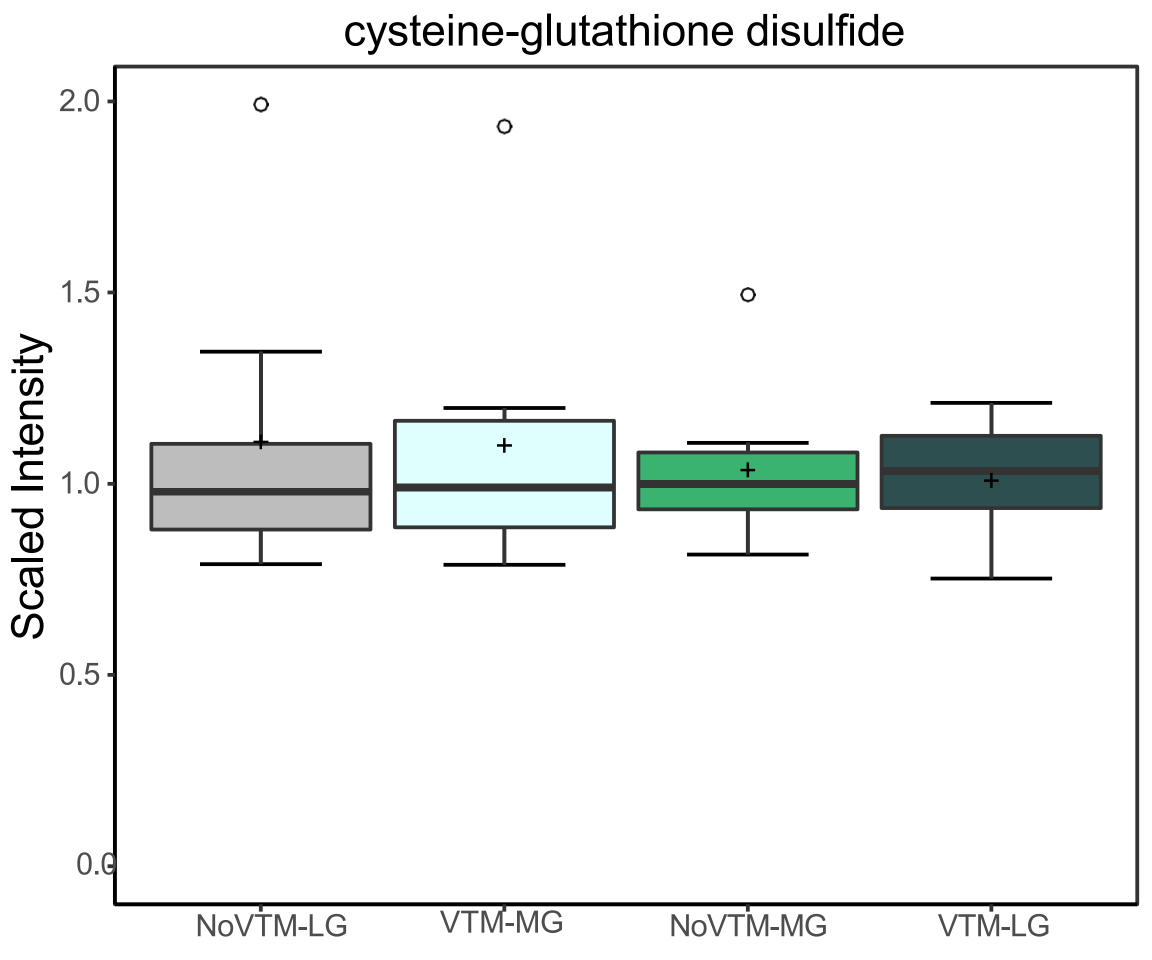

cysteine-glutathione disulfide
Scaled Intensity
2.0
1.5
1.0
0.5
0.0
NoVTM-LG
VTM-MG
NoVTM-MG
VTM-LG

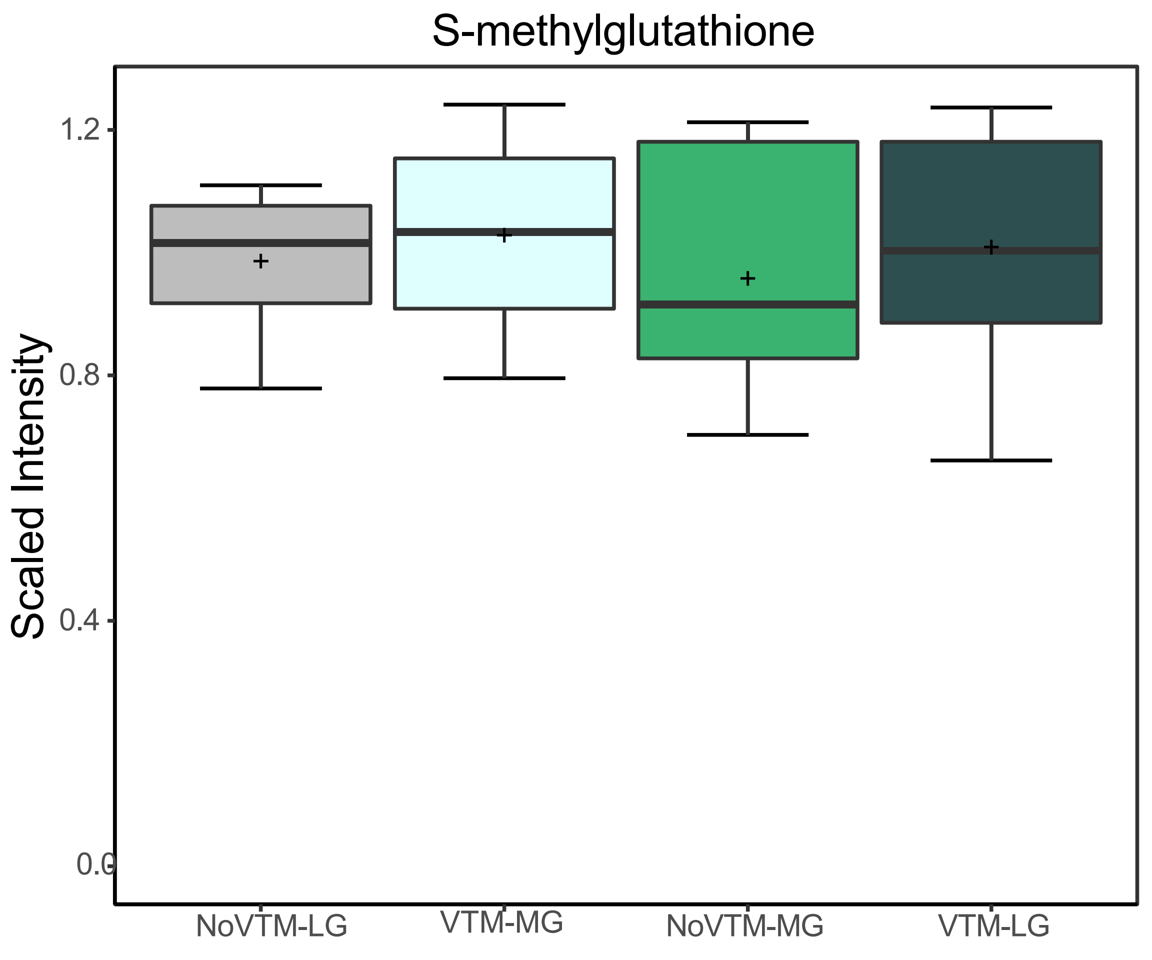

S-methylglutathione
Scaled Intensity
1.2
0.8
0.4
0.0
NoVTM-LG
VTM-MG
NoVTM-MG
VTM-LG

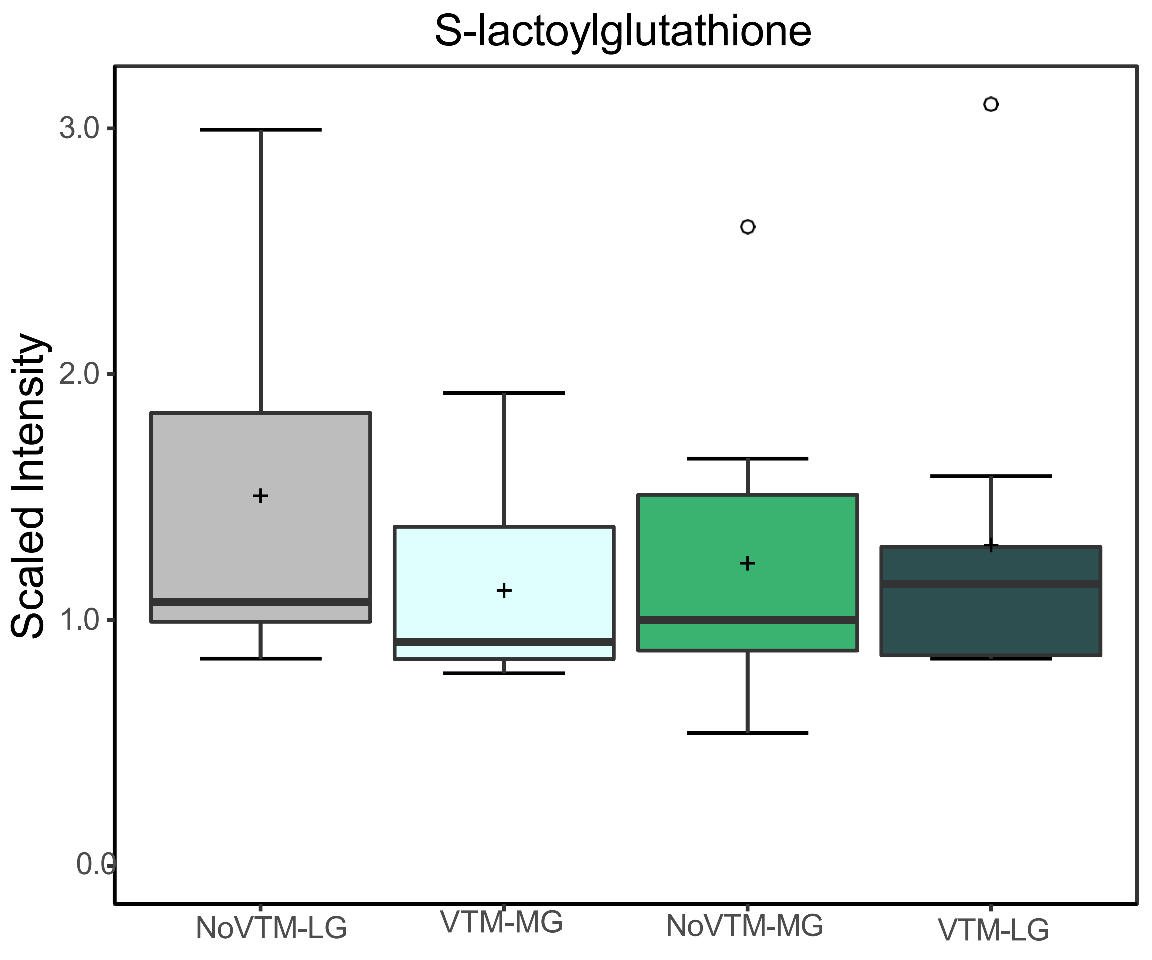
S-lactoylglutathione
Scaled Intensity
3.0
2.0
1.0
0.0
NoVTM-LG
VTM-MG
NoVTM-MG
VTM-LG

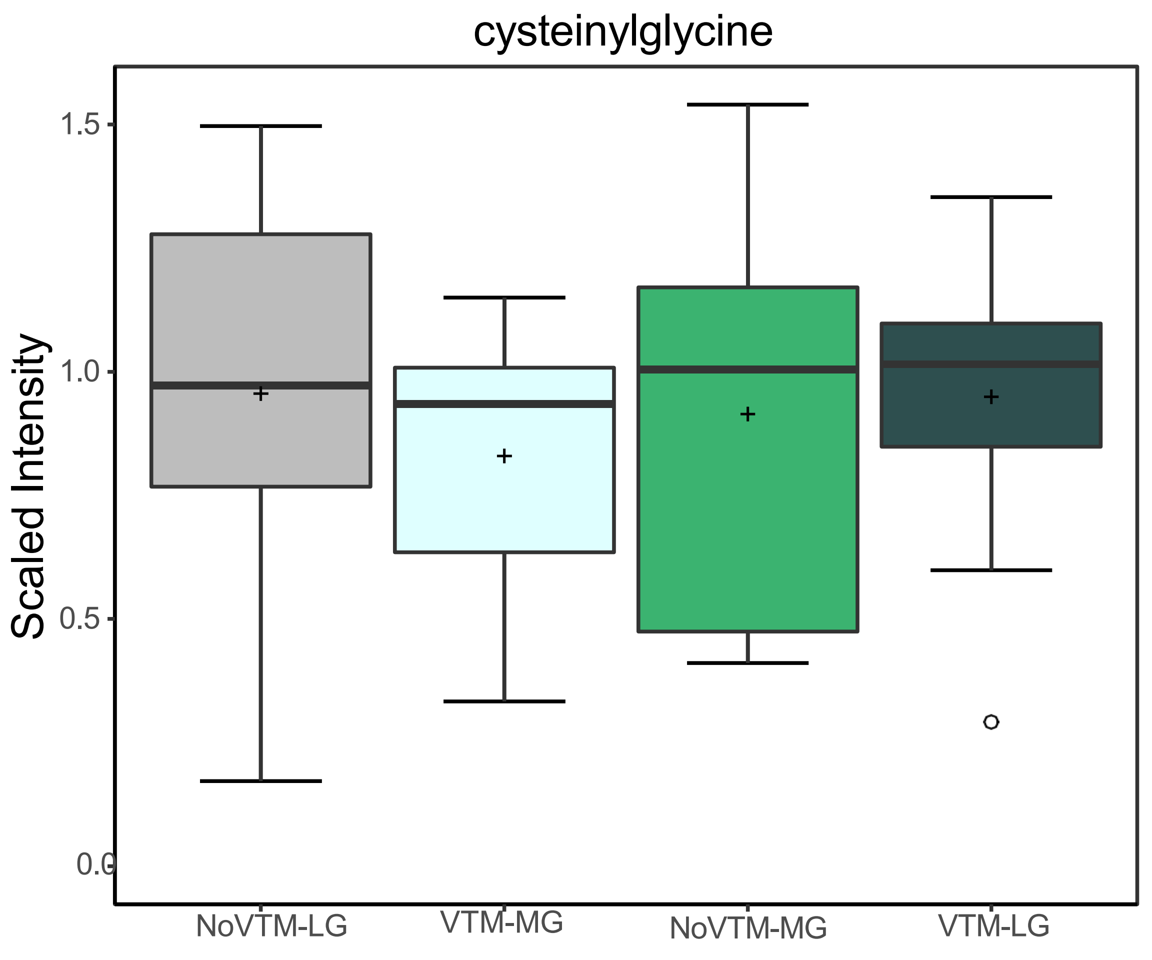
cysteinylglycine
Scaled Intensity
1.5
1.0
0.5
0.0
NoVTM-LG
VTM-MG
NoVTM-MG
VTM-LG

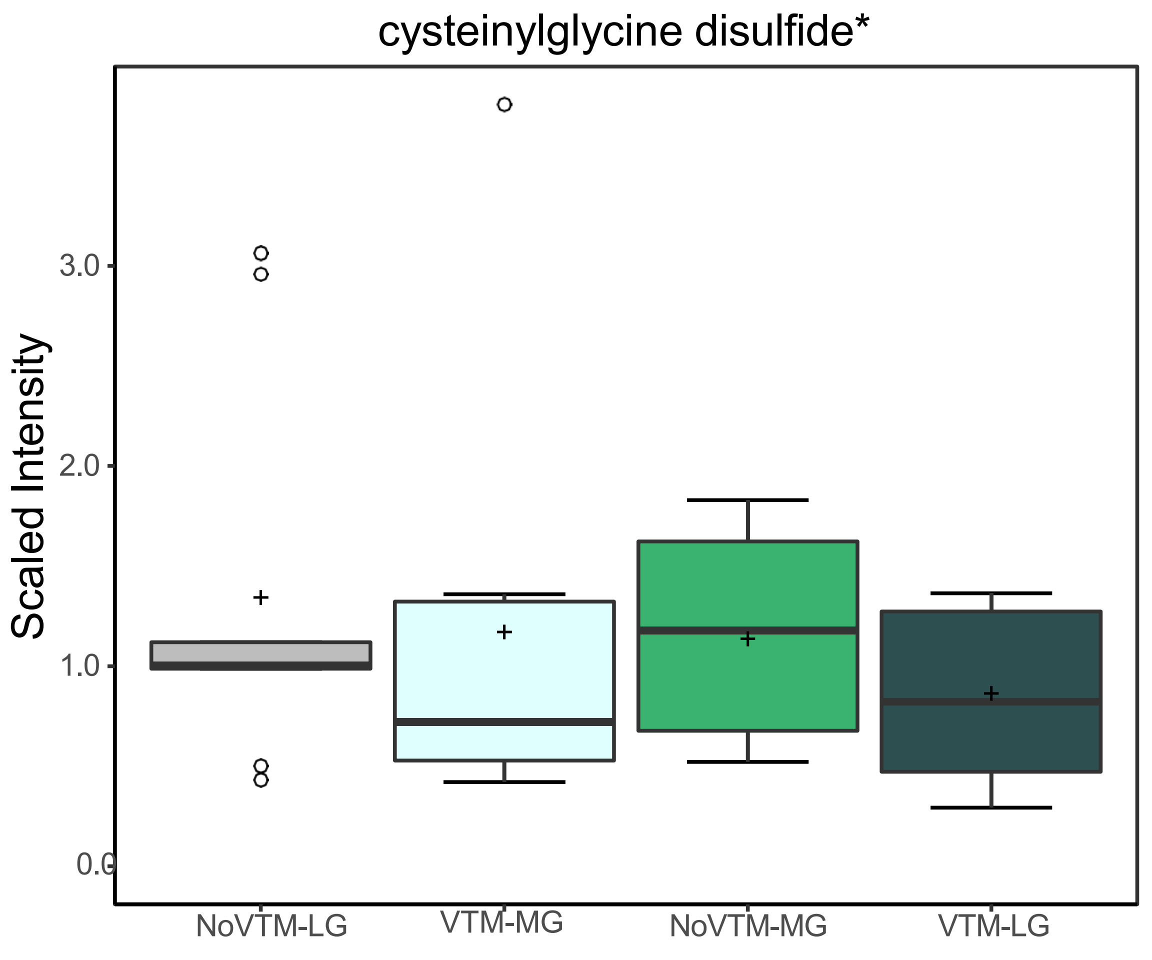
cysteinylglycine disulfide*
Scaled Intensity
3.0
2.0
1.0
0.0
NoVTM-LG
VTM-MG
NoVTM-MG
VTM-LG

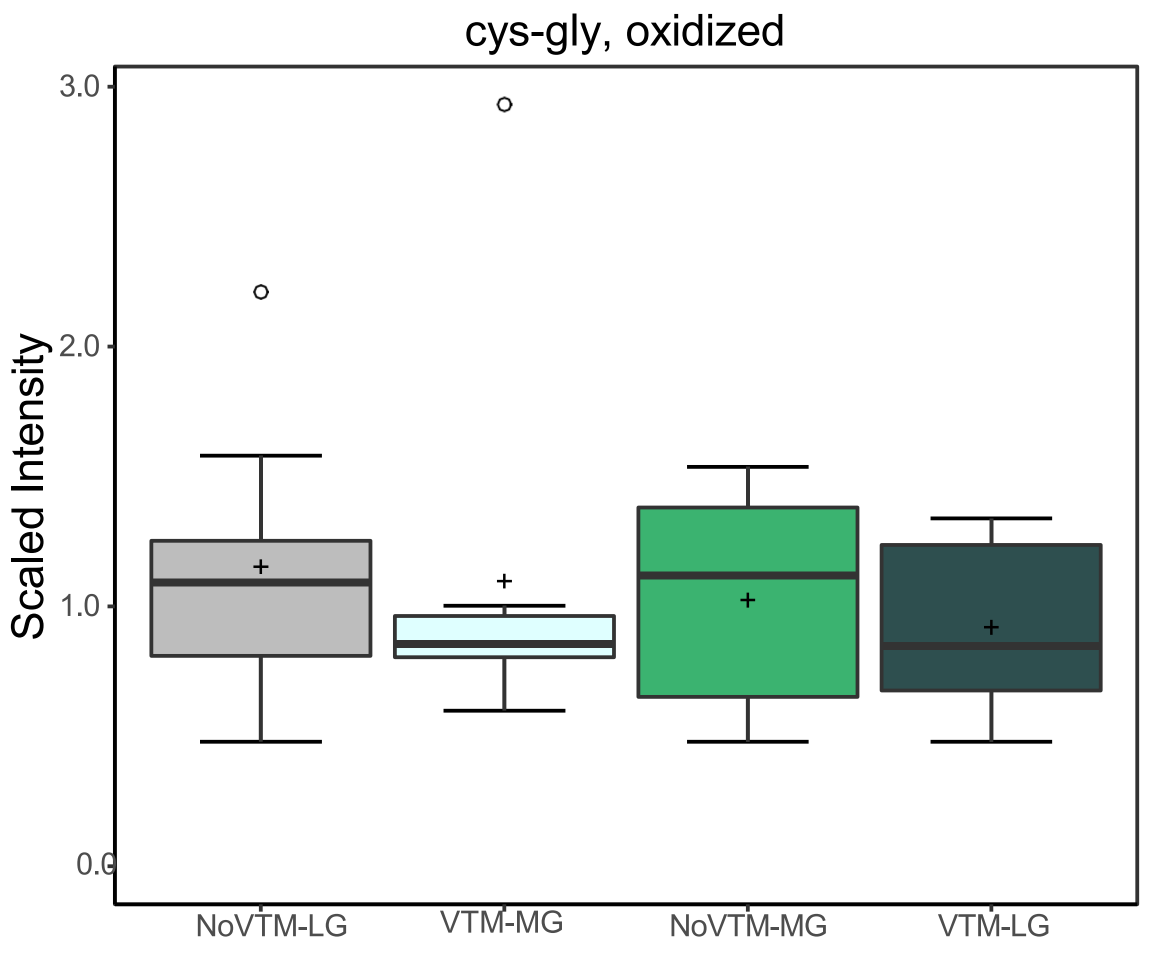

cys-gly, oxidized
Scaled Intensity
3.0
2.0
1.0
0.0
NoVTM-LG
VTM-MG
NoVTM-MG
VTM-LG

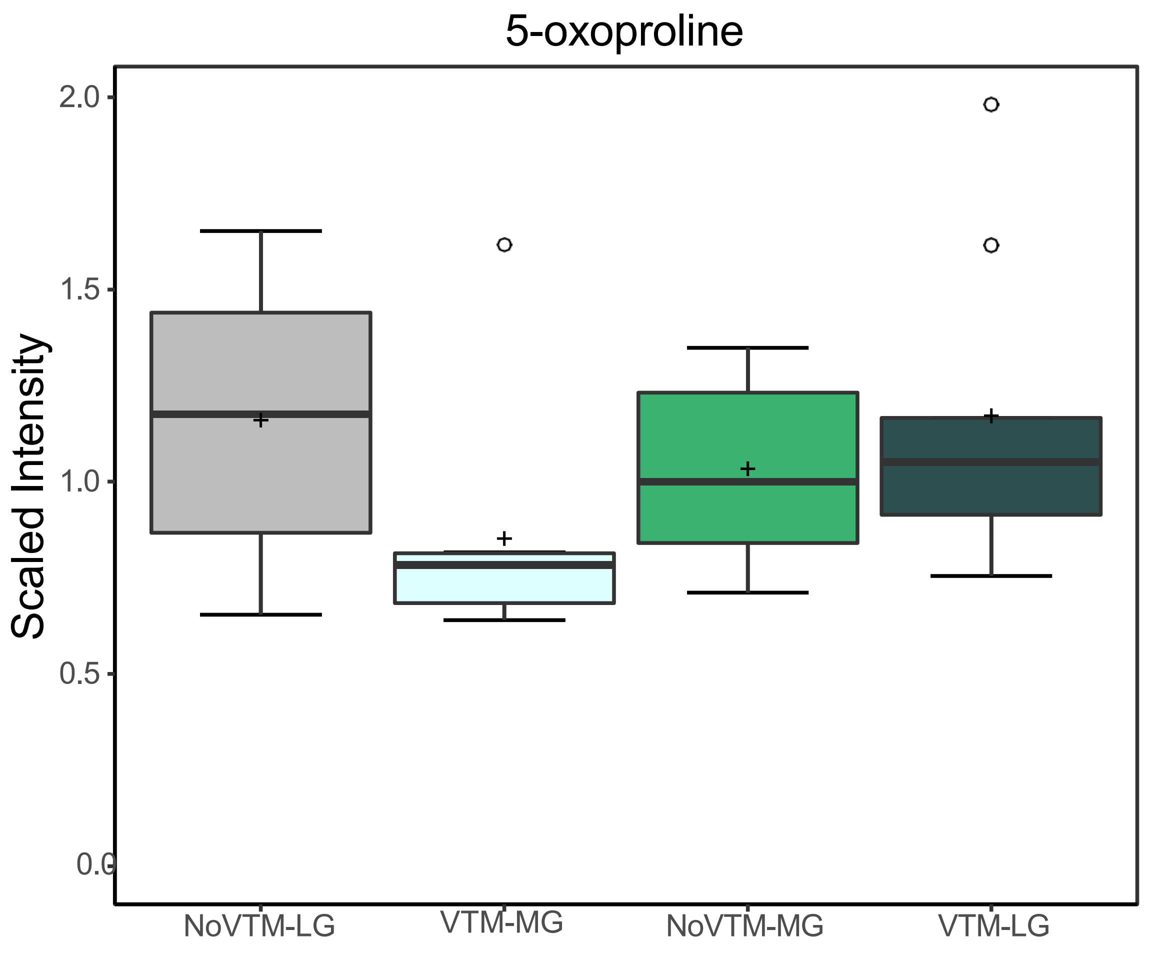

5-oxoproline
Scaled Intensity
2.0
1.5
1.0
0.5
0.0
NoVTM-LG
VTM-MG
NoVTM-MG
VTM-LG

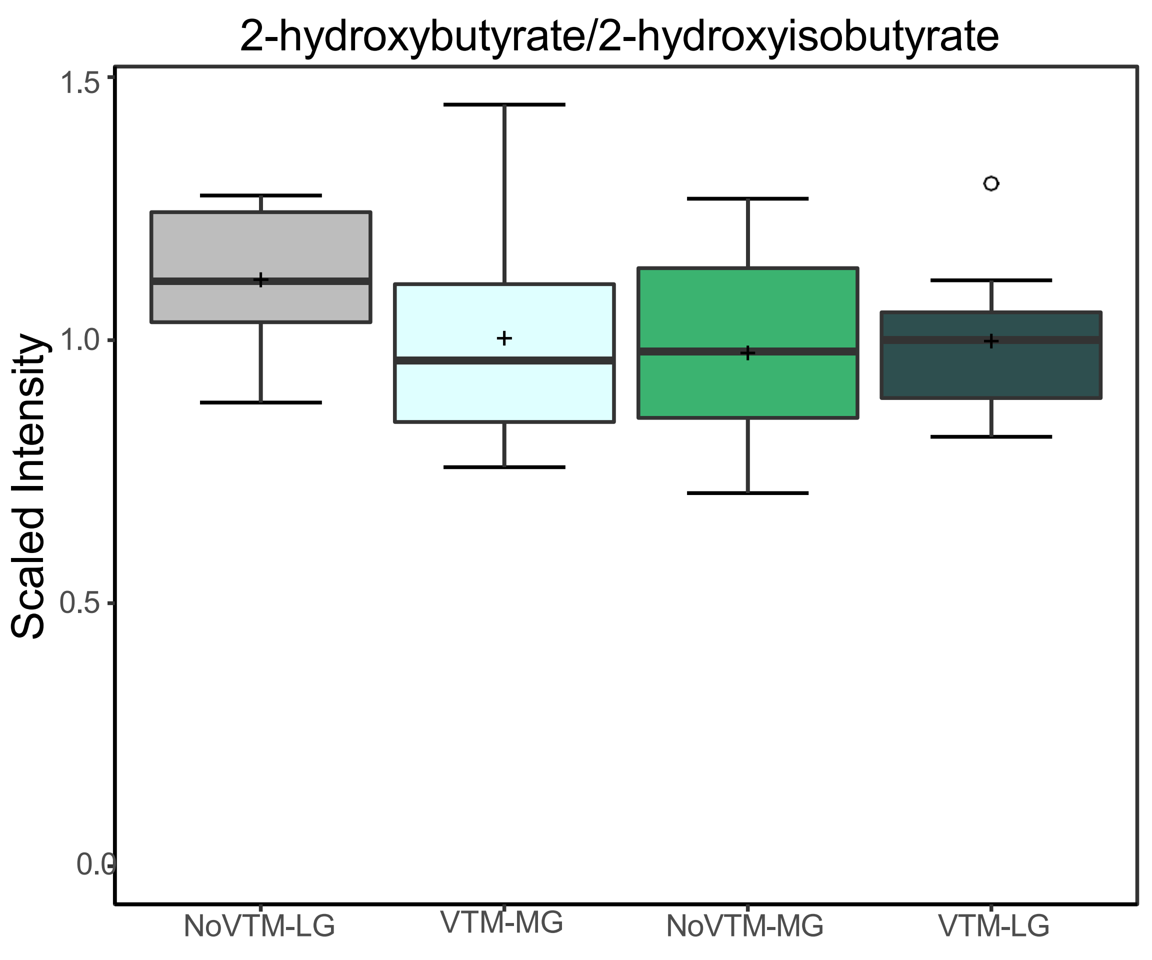
2-hydroxybutyrate/2-hydroxyisobutyrate
Scaled Intensity
1.5
1.0
0.5
0.0
NoVTM-LG
VTM-MG
NoVTM-MG
VTM-LG

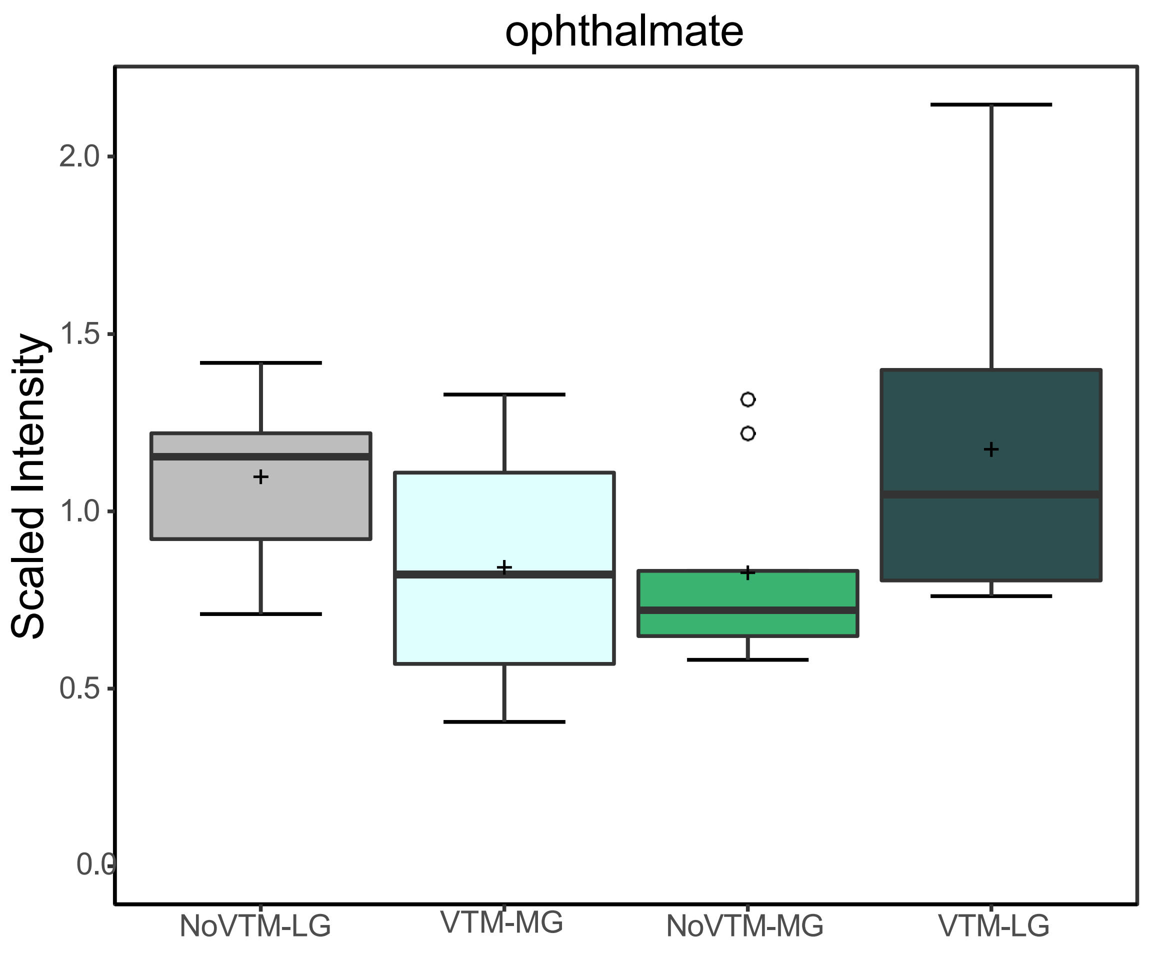
ophthalmate
Scaled Intensity
2.0
1.5
1.0
0.5
0.0
NoVTM-LG
VTM-MG
NoVTM-MG
VTM-LG

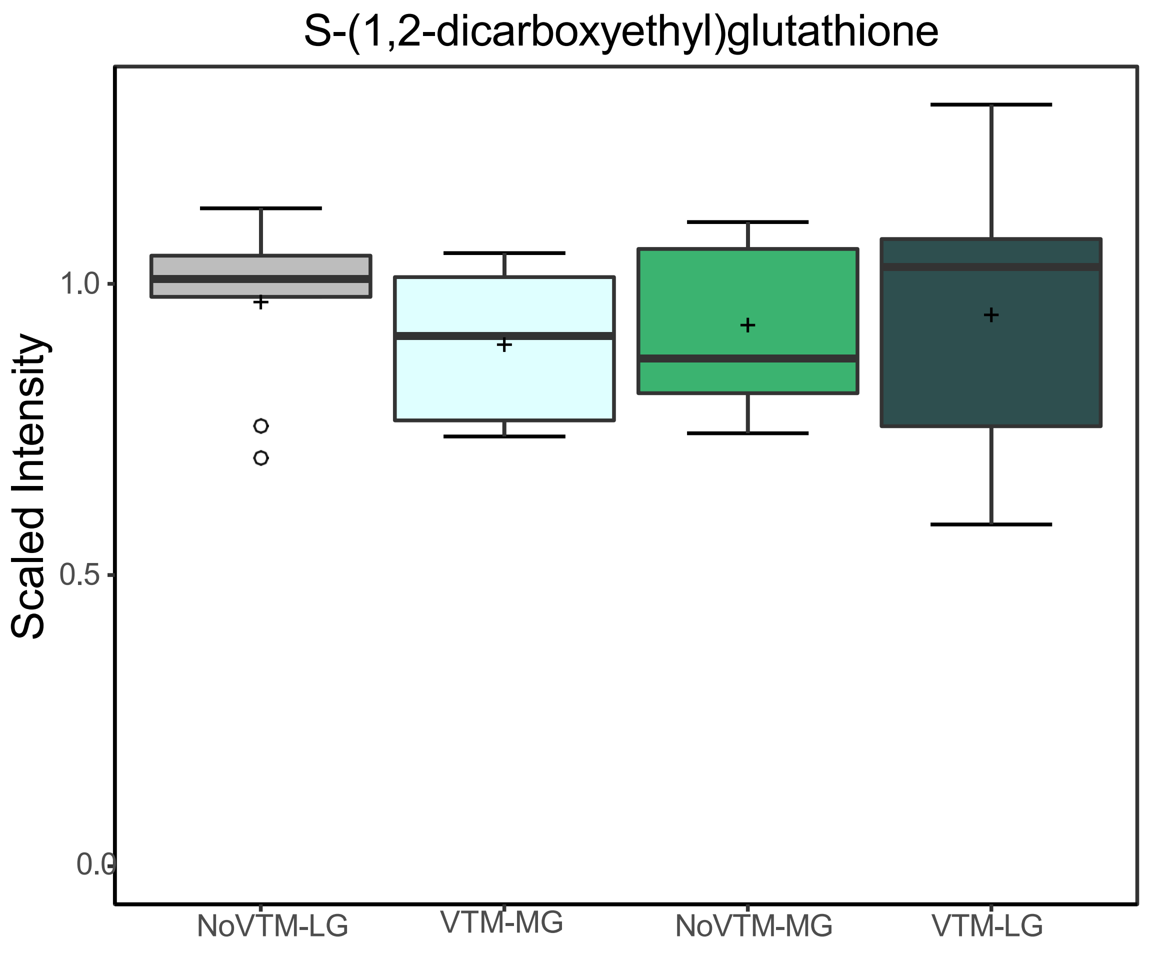

S-(1,2-dicarboxyethyl)glutathione
Scaled Intensity
1.0
0.5
0.0
NoVTM-LG
VTM-MG
NoVTM-MG
VTM-LG

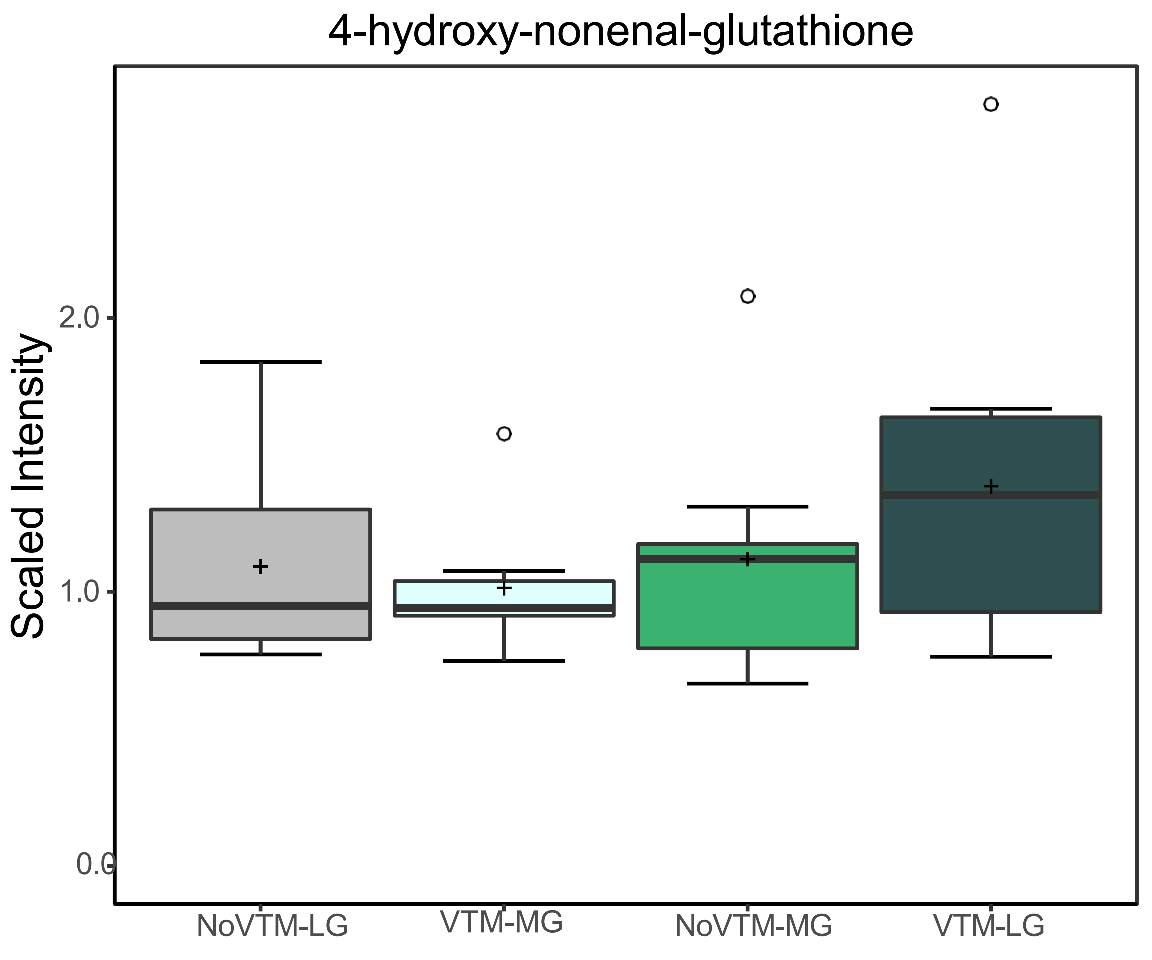

4-hydroxy-nonenal-glutathione
Scaled Intensity
2.0
1.0
0.0
NoVTM-LG
VTM-MG
NoVTM-MG
VTM-LG

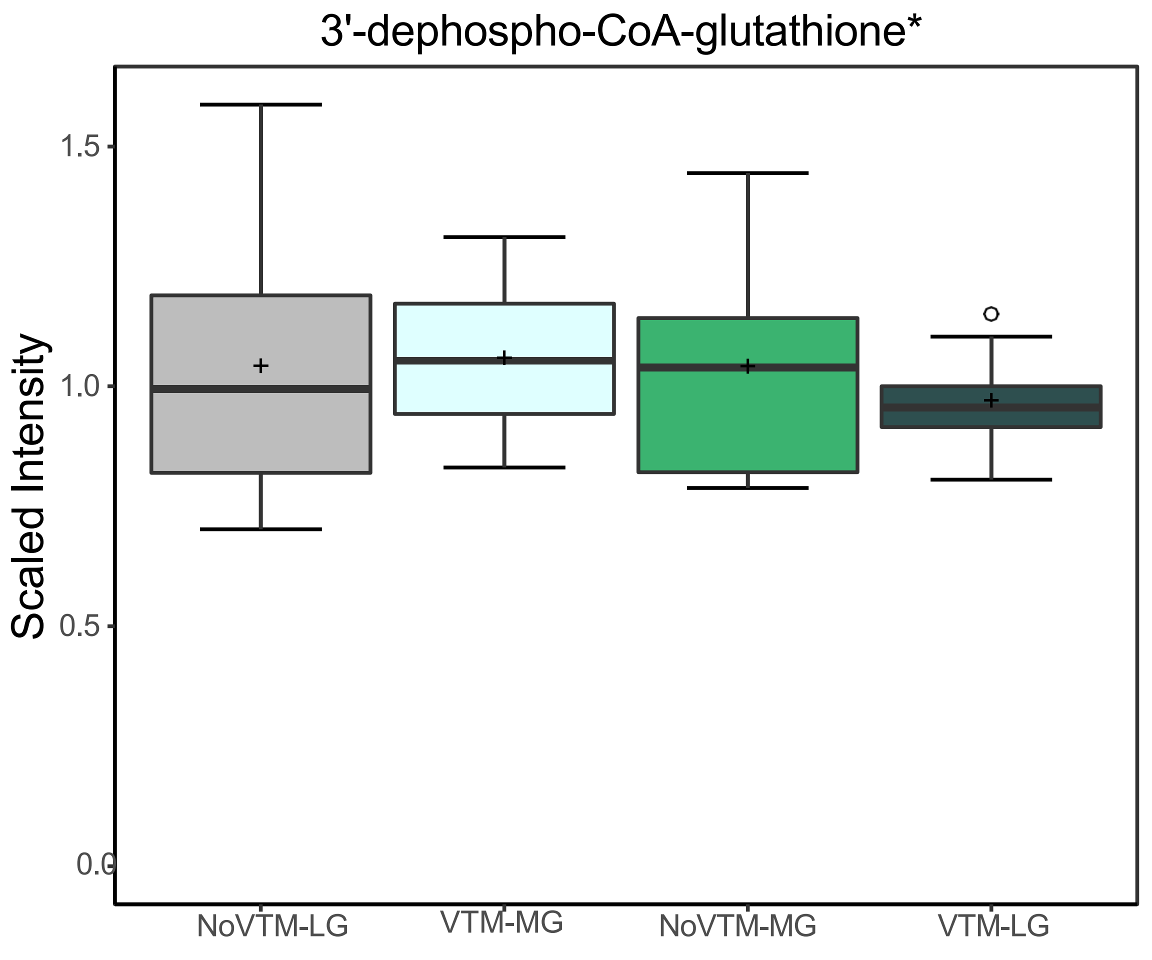
3'-dephospho-CoA-glutathione*
Scaled Intensity
1.5
1.0
0.5
0.0
NoVTM-LG
VTM-MG
NoVTM-MG
VTM-LG

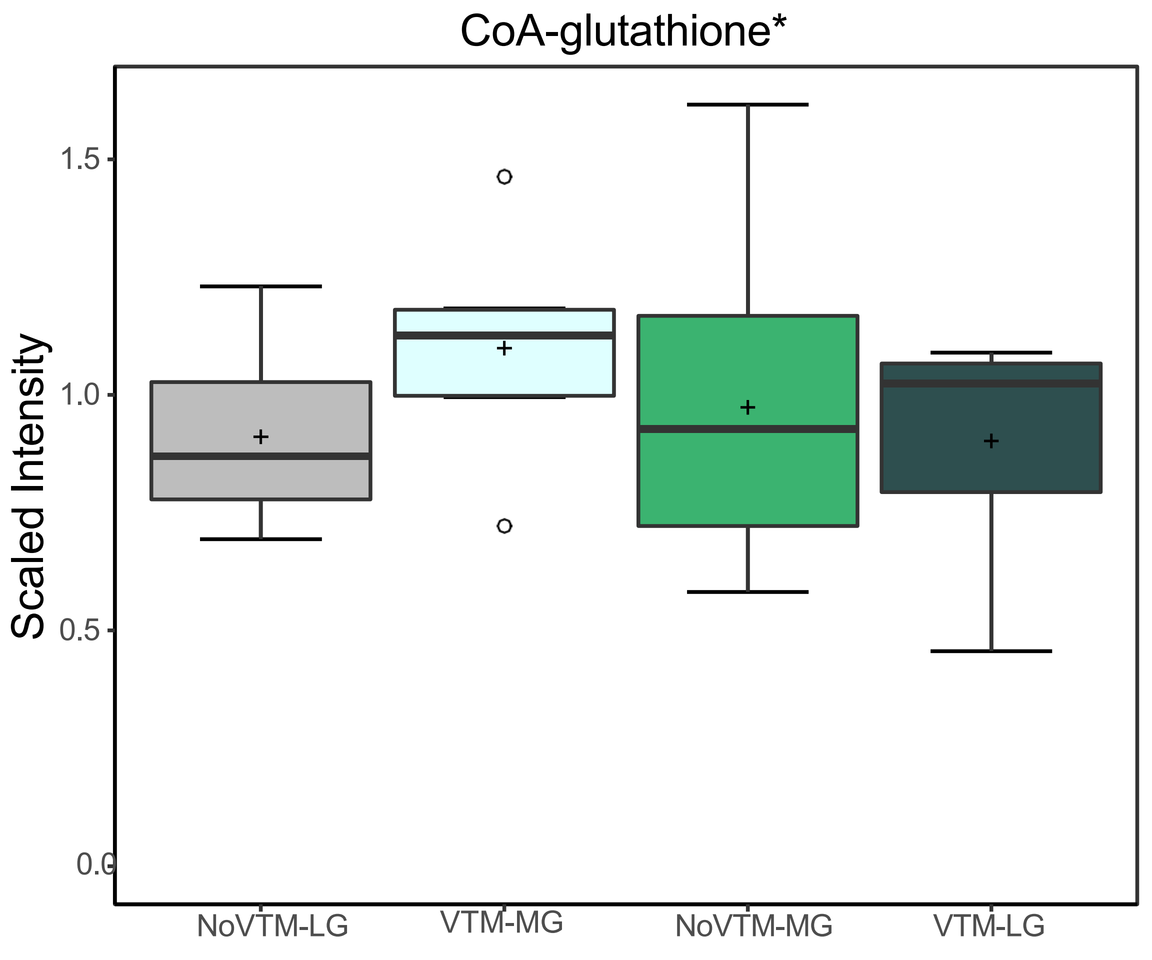
CoA-glutathione*
Scaled Intensity
1.5
1.0
0.5
0.0
NoVTM-LG
VTM-MG
NoVTM-MG
VTM-LG

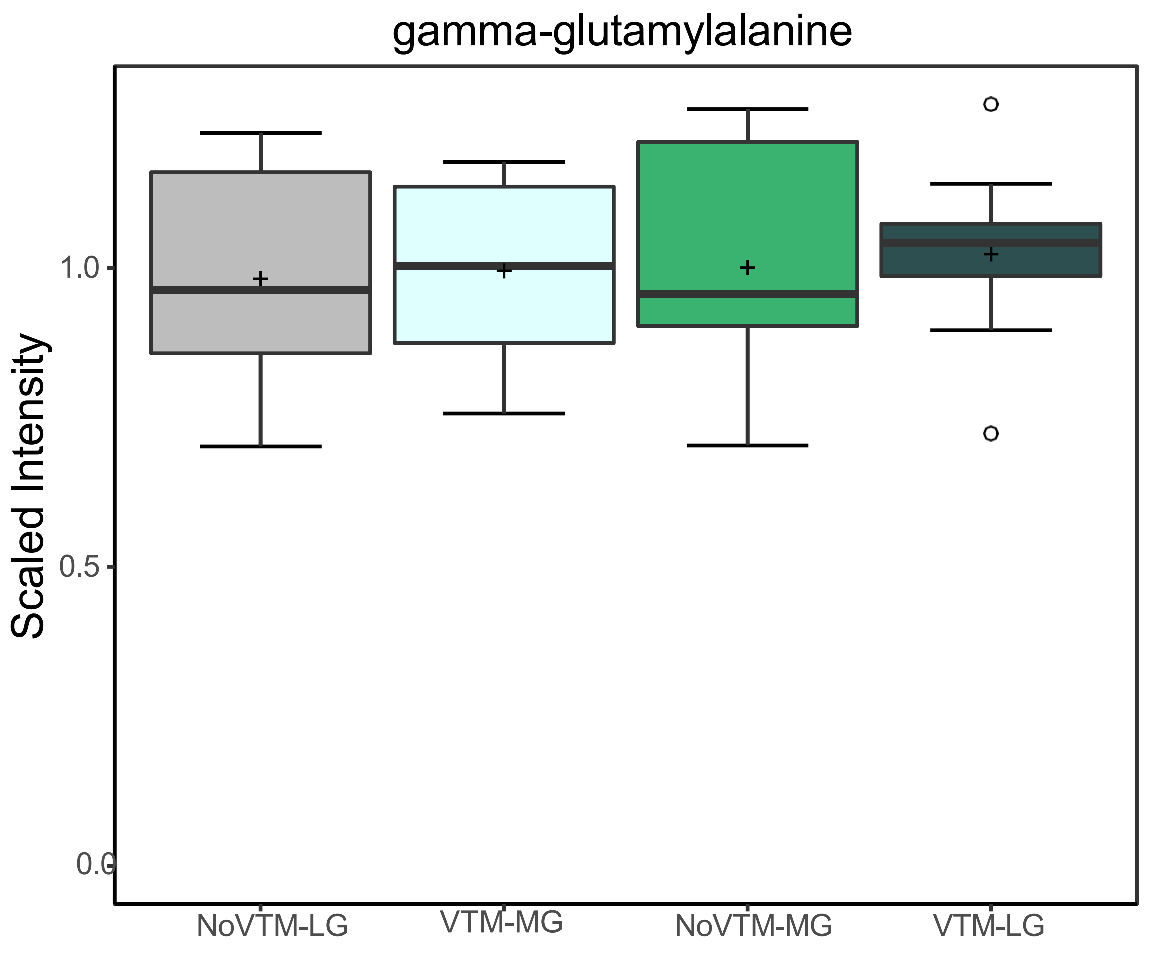
gamma-glutamylalanine
Scaled Intensity
1.0
0.5
0.0
NoVTM-LG
VTM-MG
NoVTM-MG
VTM-LG

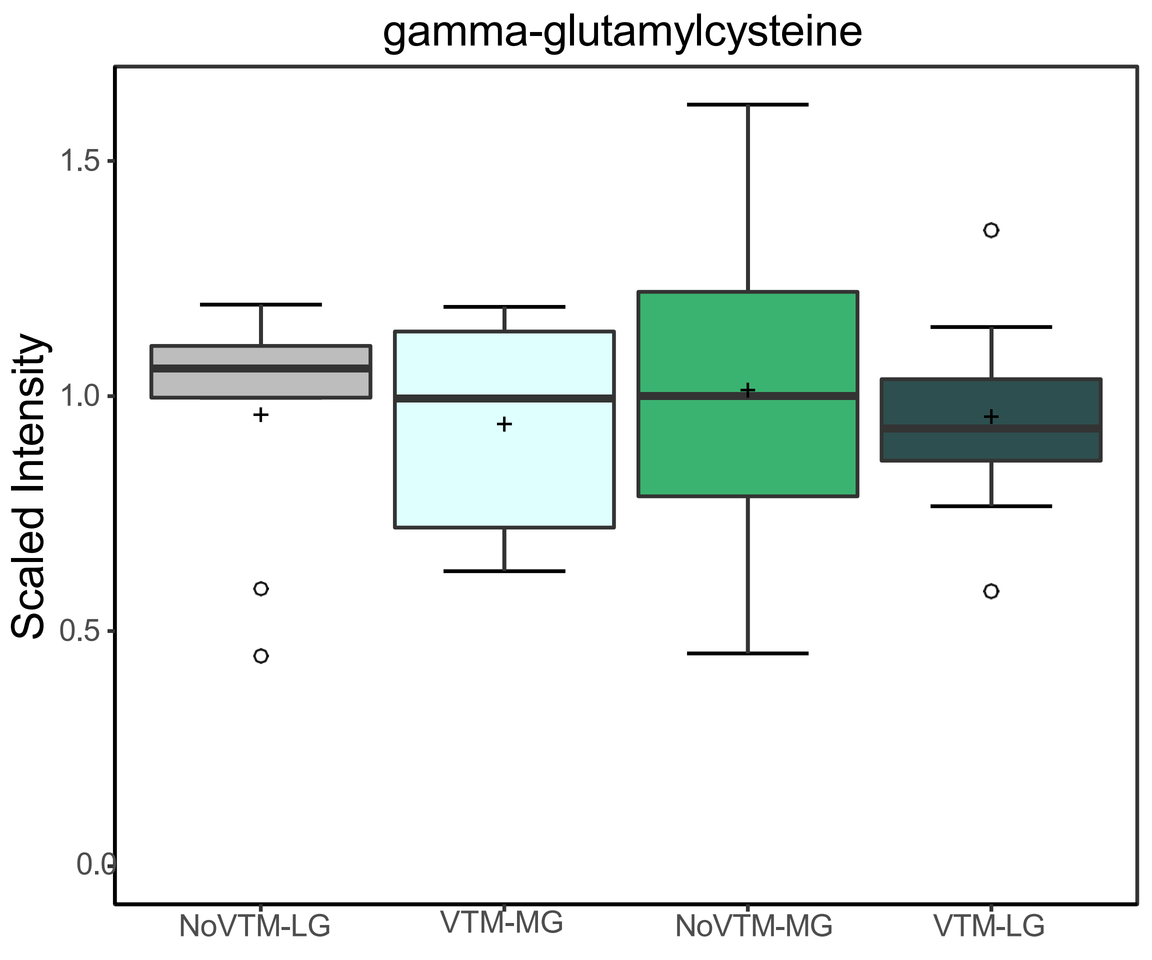

gamma-glutamylcysteine
Scaled Intensity
1.5
1.0
0.5
0.0
NoVTM-LG
VTM-MG
NoVTM-MG
VTM-LG

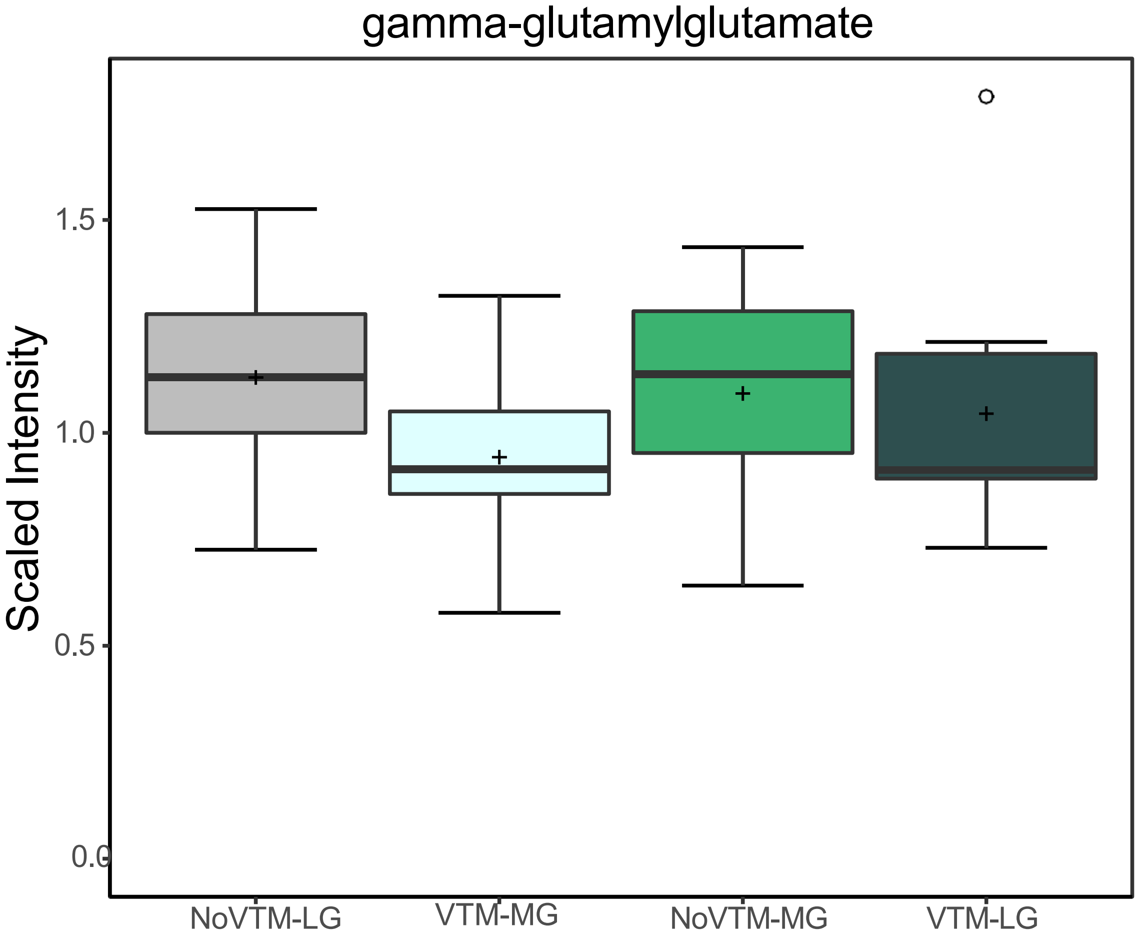
gamma-glutamylglutamate
Scaled Intensity
1.5
1.0
0.5
0.0
NoVTM-LG
VTM-MG
NoVTM-MG
VTM-LG

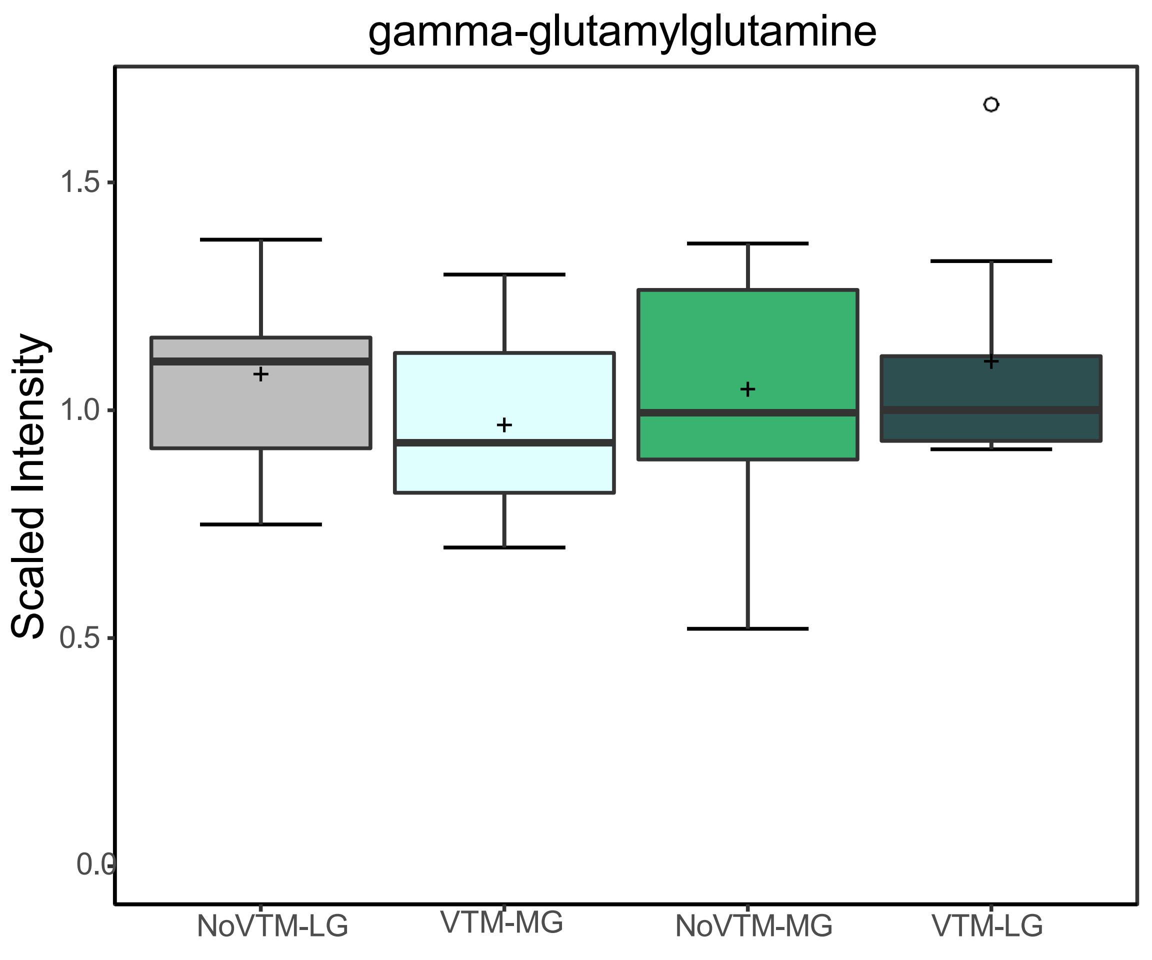

gamma-glutamylglutamine
Scaled Intensity
1.5
1.0
0.5
0.0
NoVTM-LG
VTM-MG
NoVTM-MG
VTM-LG

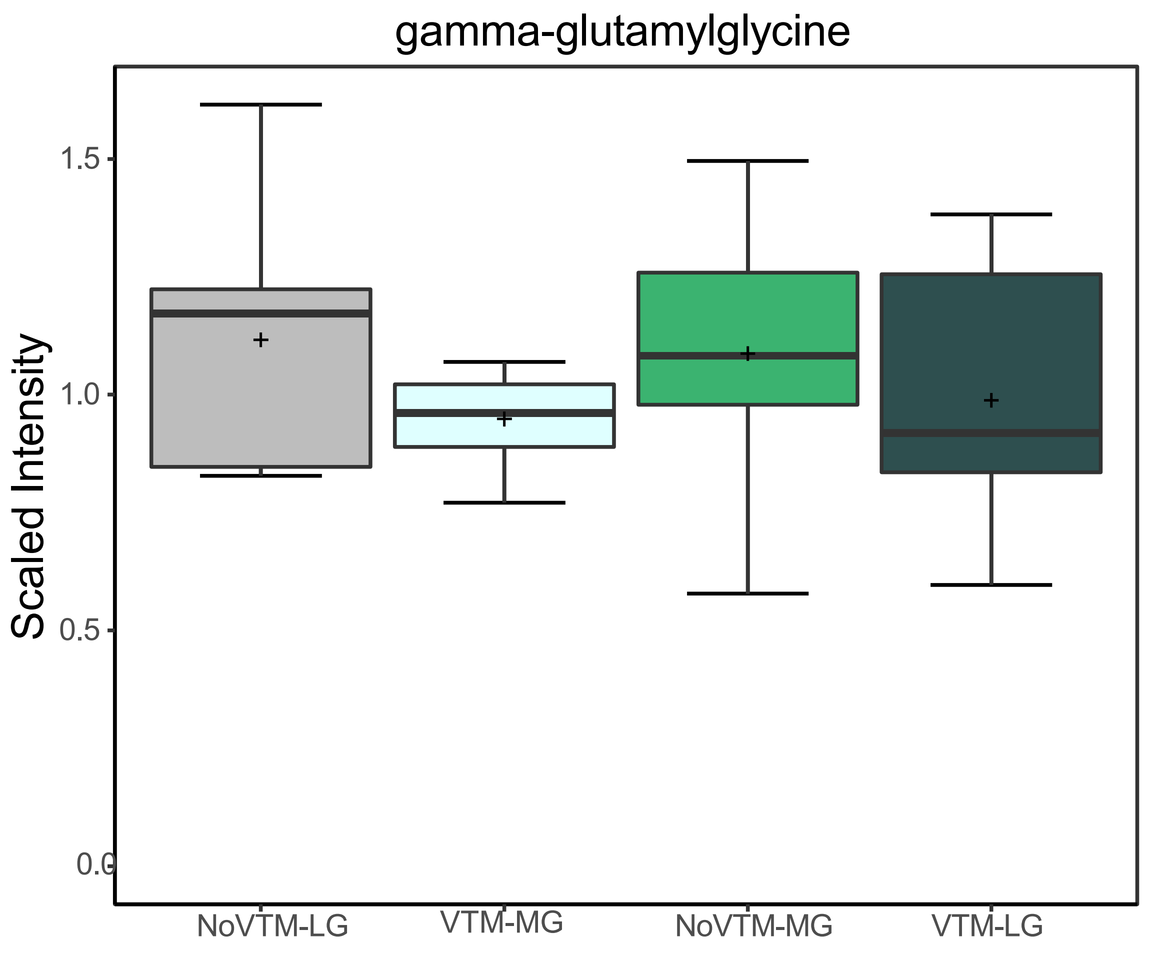
gamma-glutamylglycine
Scaled Intensity
1.5
1.0
0.5
0.0
NoVTM-LG
VTM-MG
NoVTM-MG
VTM-LG

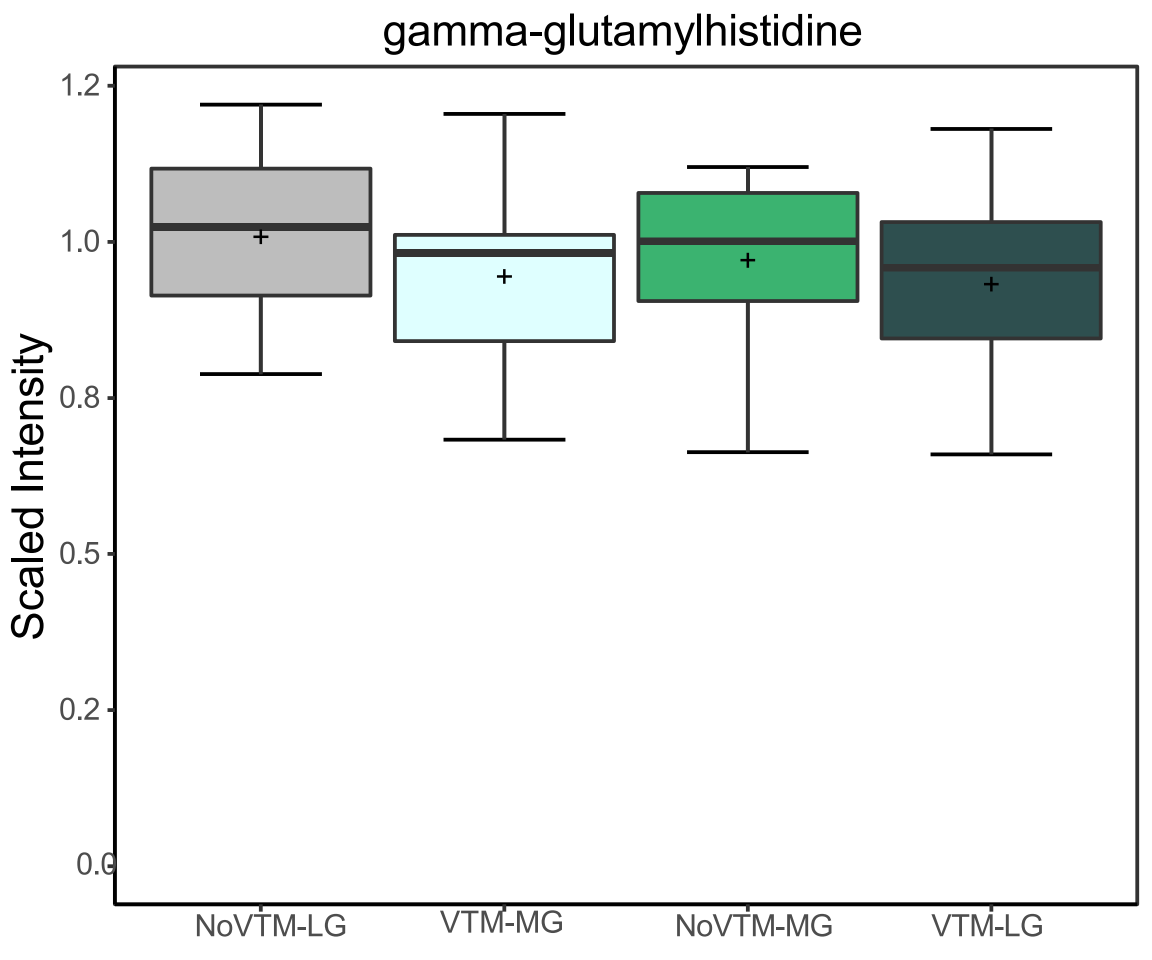

gamma-glutamylhistidine
Scaled Intensity
1.2
1.0
0.8
0.5
0.2
0.0
NoVTM-LG
VTM-MG
NoVTM-MG
VTM-LG

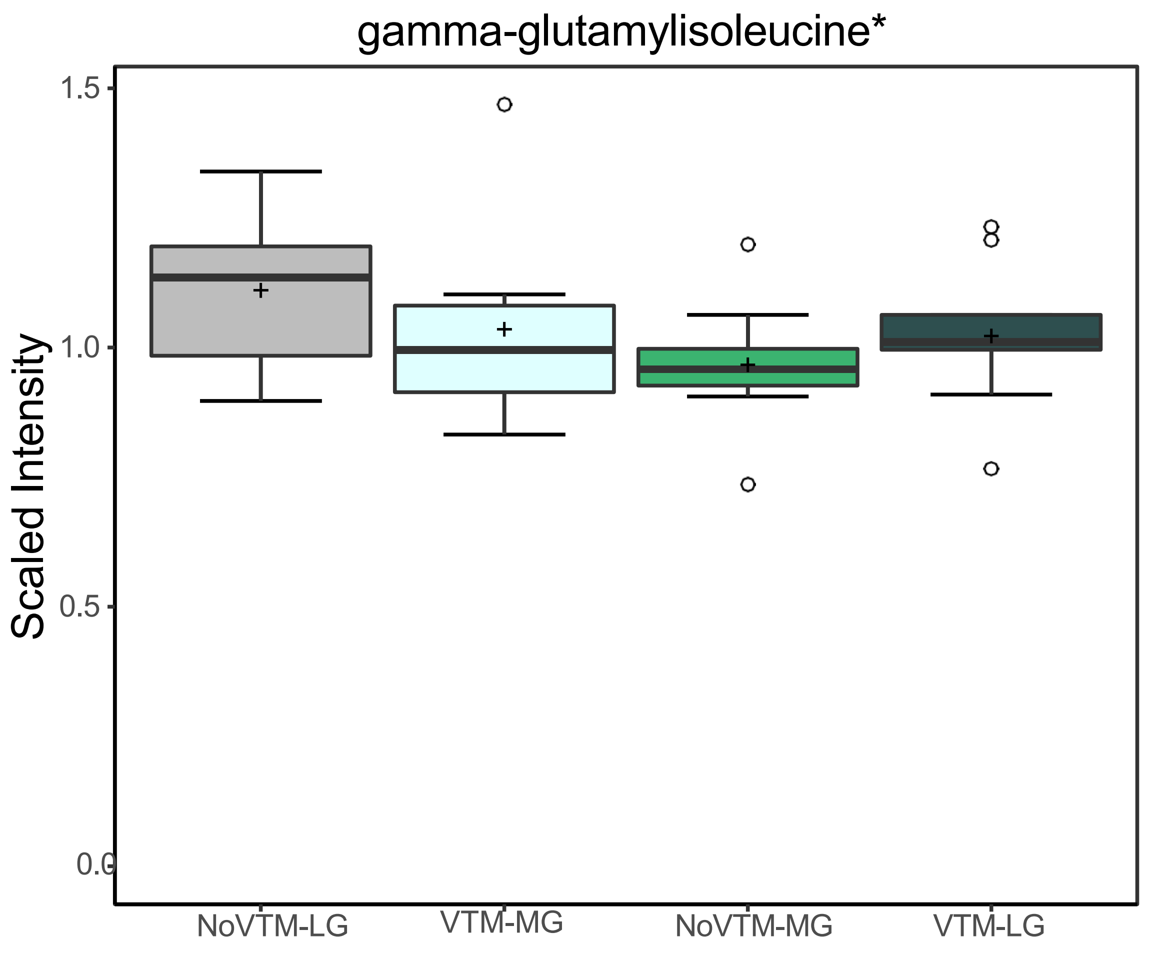
gamma-glutamylisoleucine*
Scaled Intensity
1.5
1.0
0.5
0.0
NoVTM-LG
VTM-MG
NoVTM-MG
VTM-LG

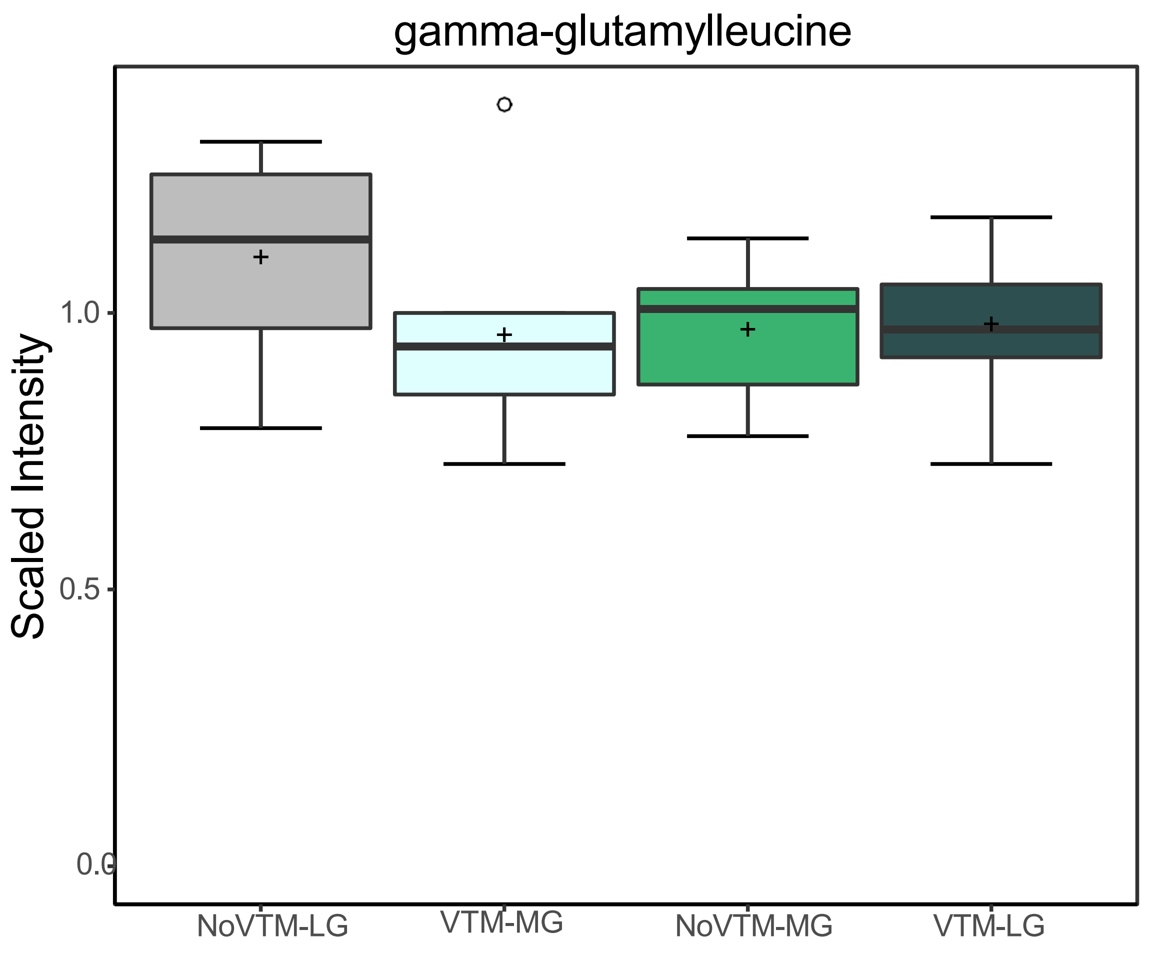

gamma-glutamylleucine
Scaled Intensity
1.0
0.5
0.0
NoVTM-LG
VTM-MG
NoVTM-MG
VTM-LG

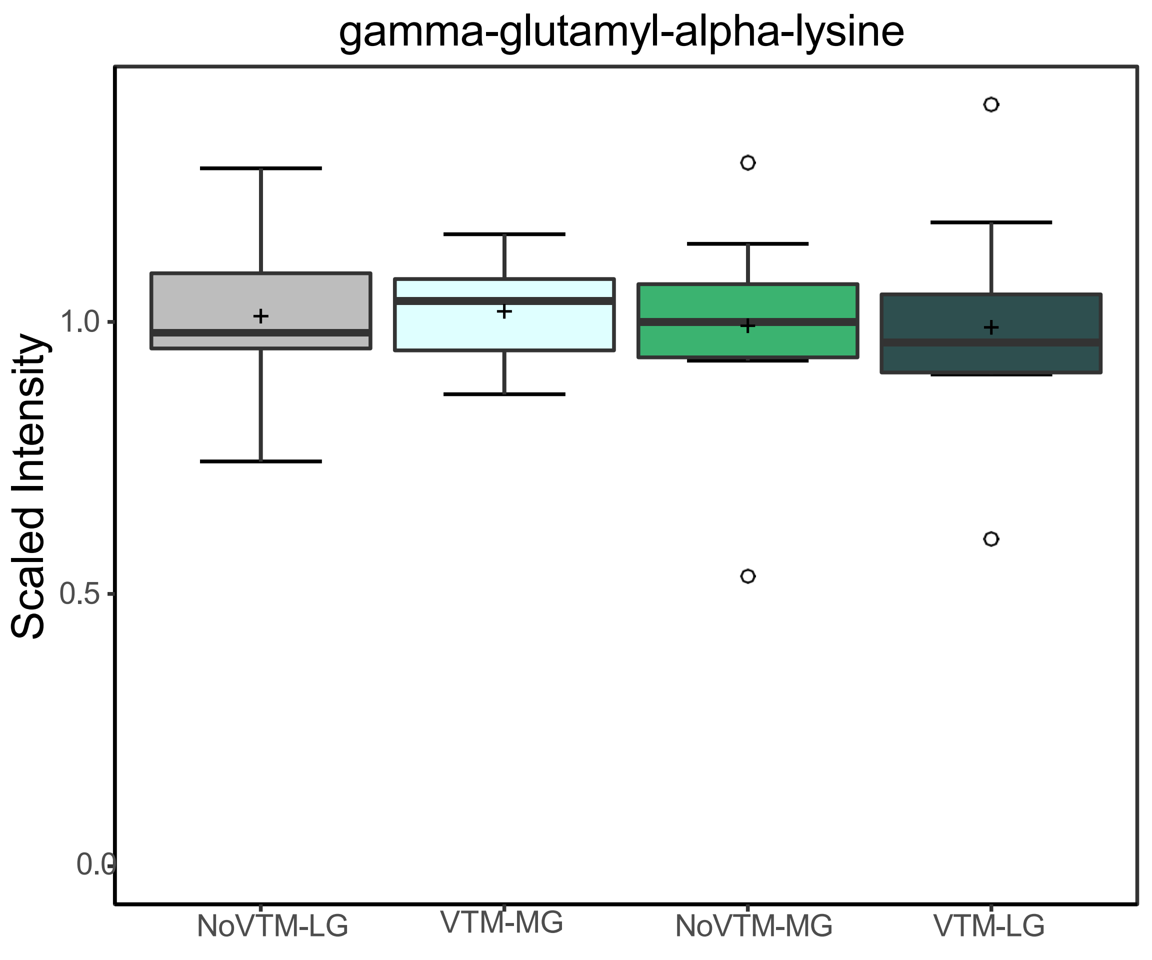
gamma-glutamyl-alpha-lysine
Scaled Intensity
1.0
0.5
0.0
NoVTM-LG
VTM-MG
NoVTM-MG
VTM-LG

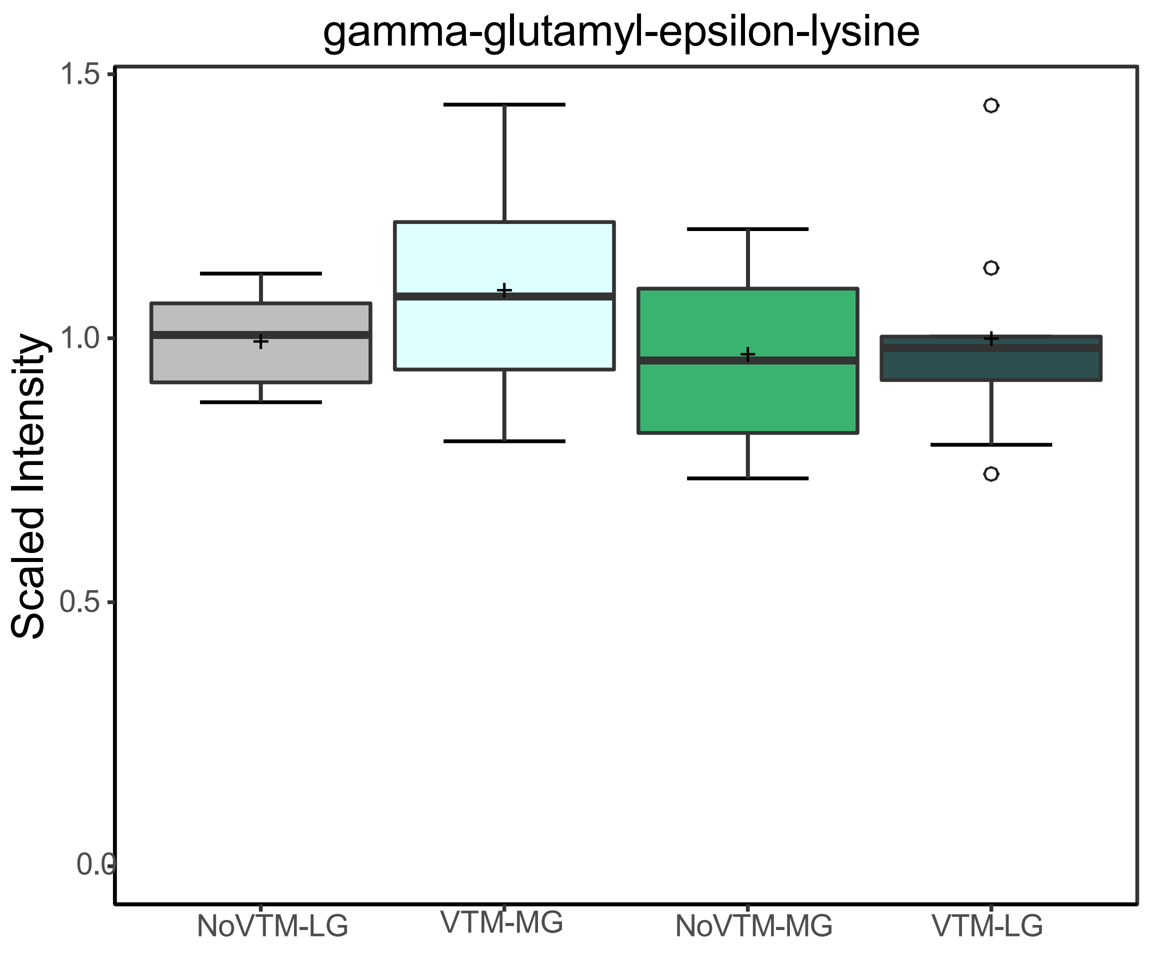

gamma-glutamyl-epsilon-lysine
Scaled Intensity
1.5
1.0
0.5
0.0
NoVTM-LG
VTM-MG
NoVTM-MG
VTM-LG

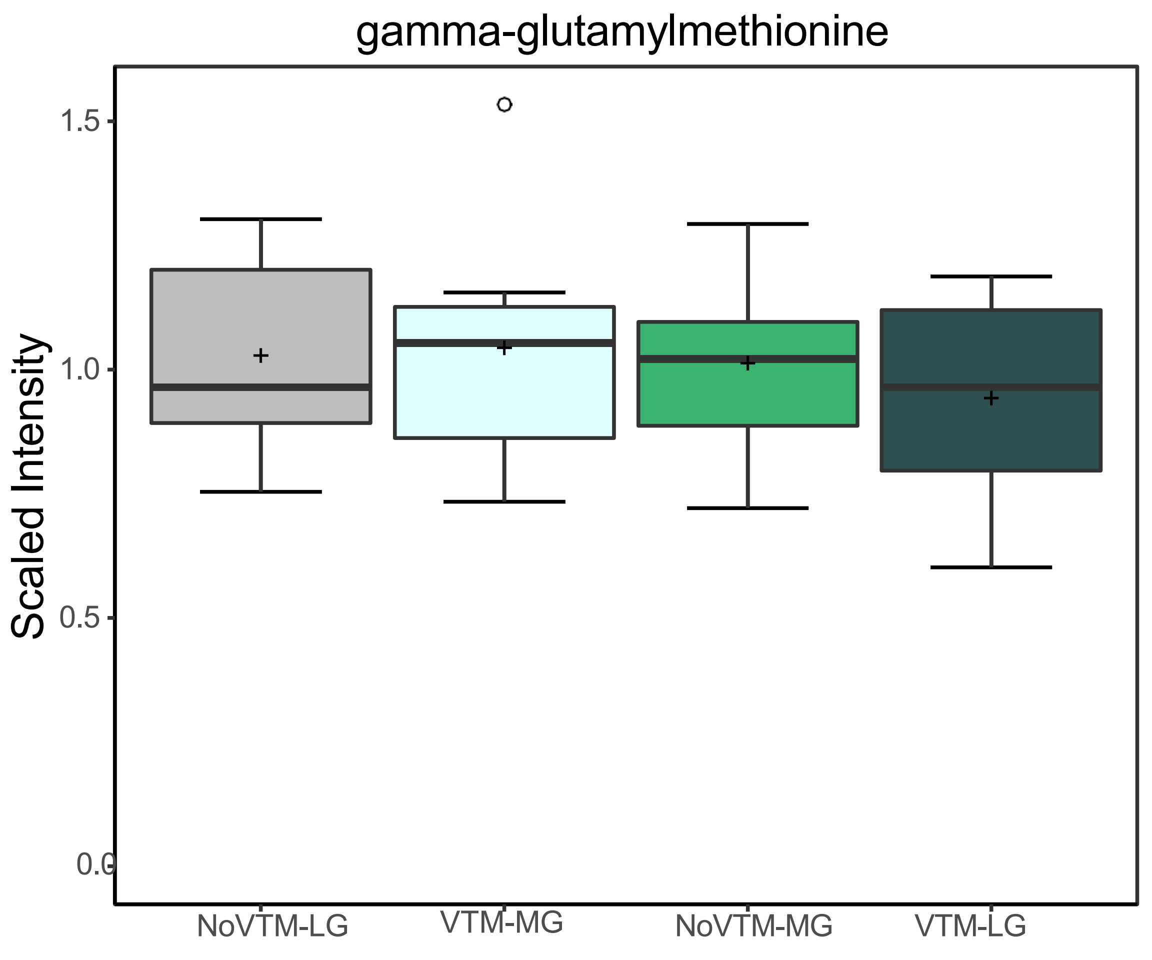
gamma-glutamylmethionine
Scaled Intensity
1.5
1.0
0.5
0.0
NoVTM-LG
VTM-MG
NoVTM-MG
VTM-LG

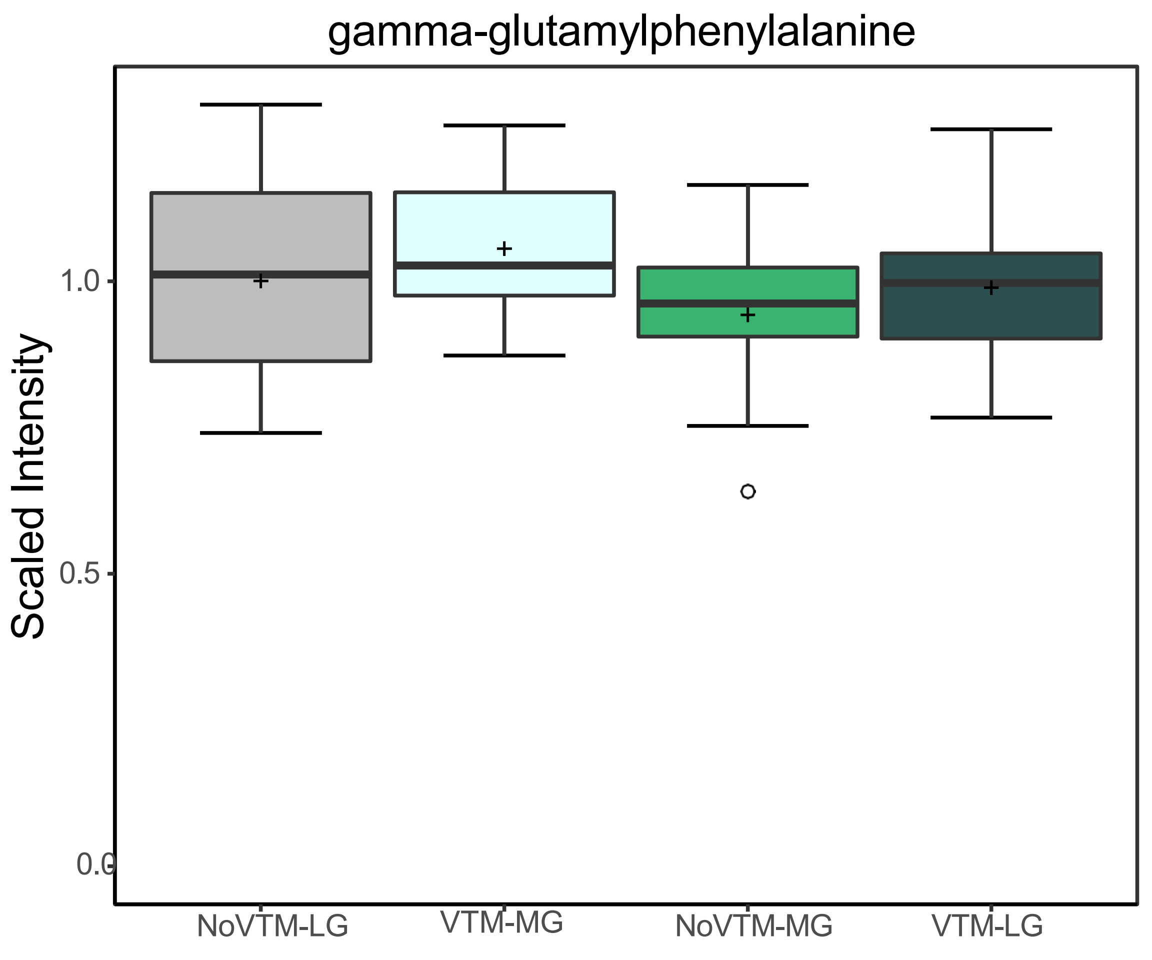

gamma-glutamylphenylalanine
Scaled Intensity
1.0
0.5
0.0
NoVTM-LG
VTM-MG
NoVTM-MG
VTM-LG

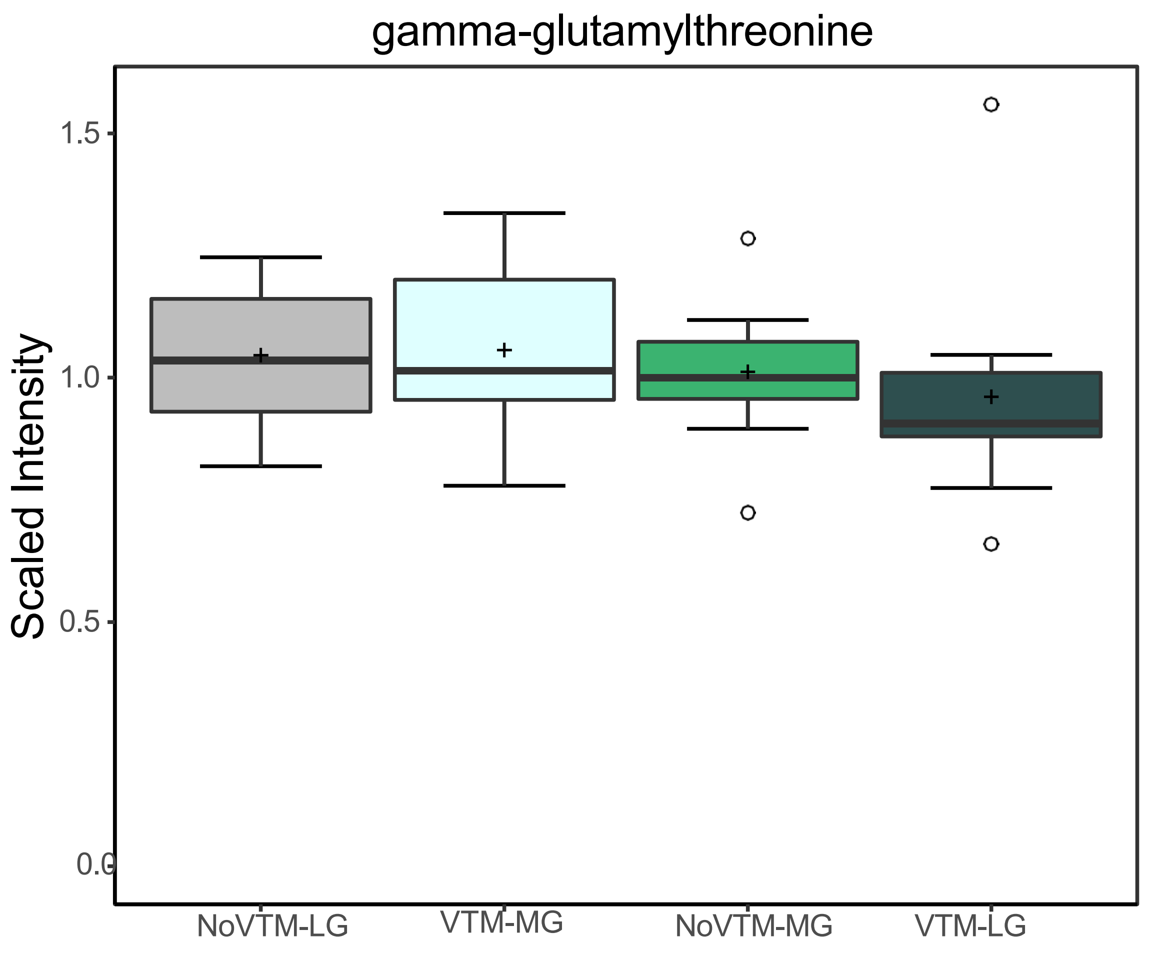
gamma-glutamylthreonine
Scaled Intensity
1.5
1.0
0.5
0.0
NoVTM-LG
VTM-MG
NoVTM-MG
VTM-LG

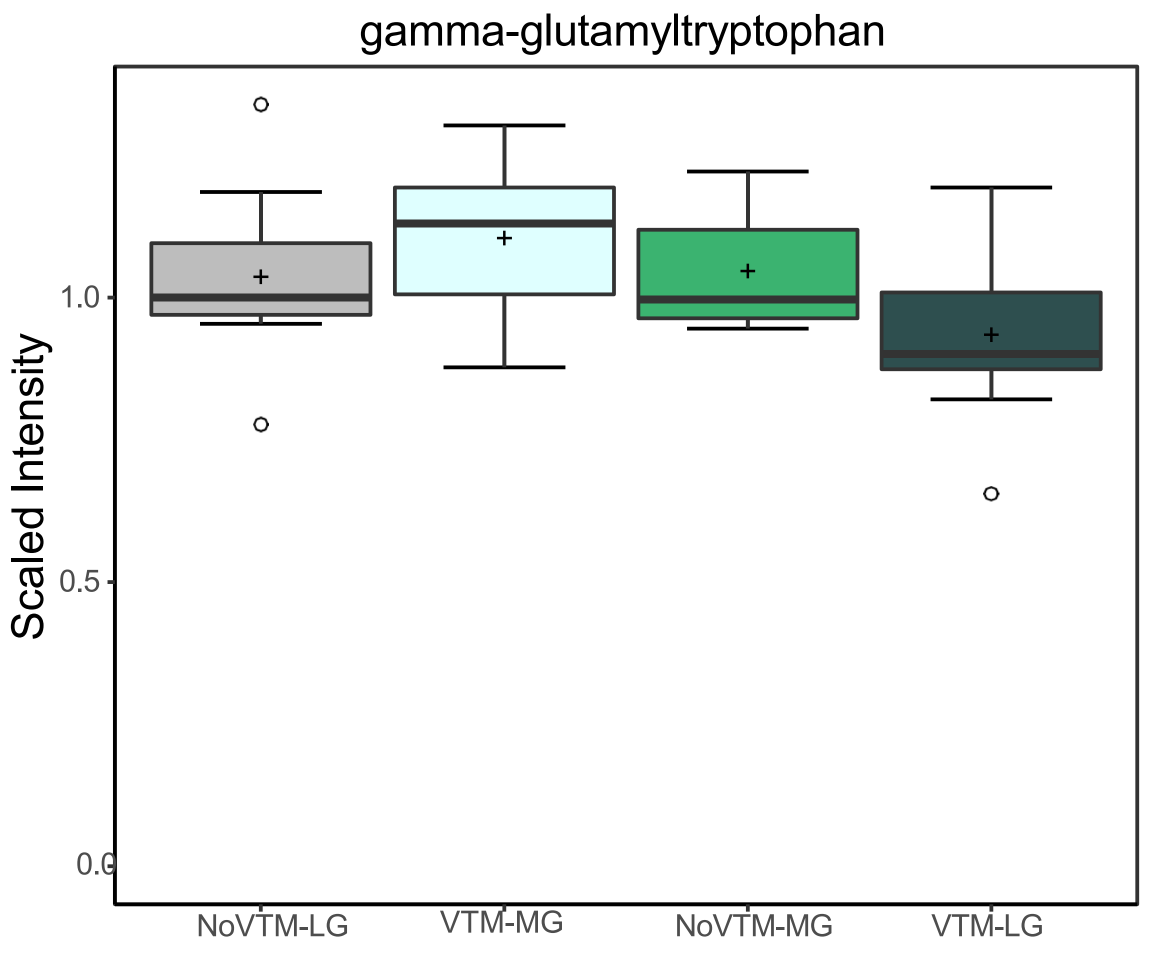
gamma-glutamyltryptophan
Scaled Intensity
1.0
0.5
0.0
NoVTM-LG
VTM-MG
NoVTM-MG
VTM-LG

gamma-glutamyltyrosine

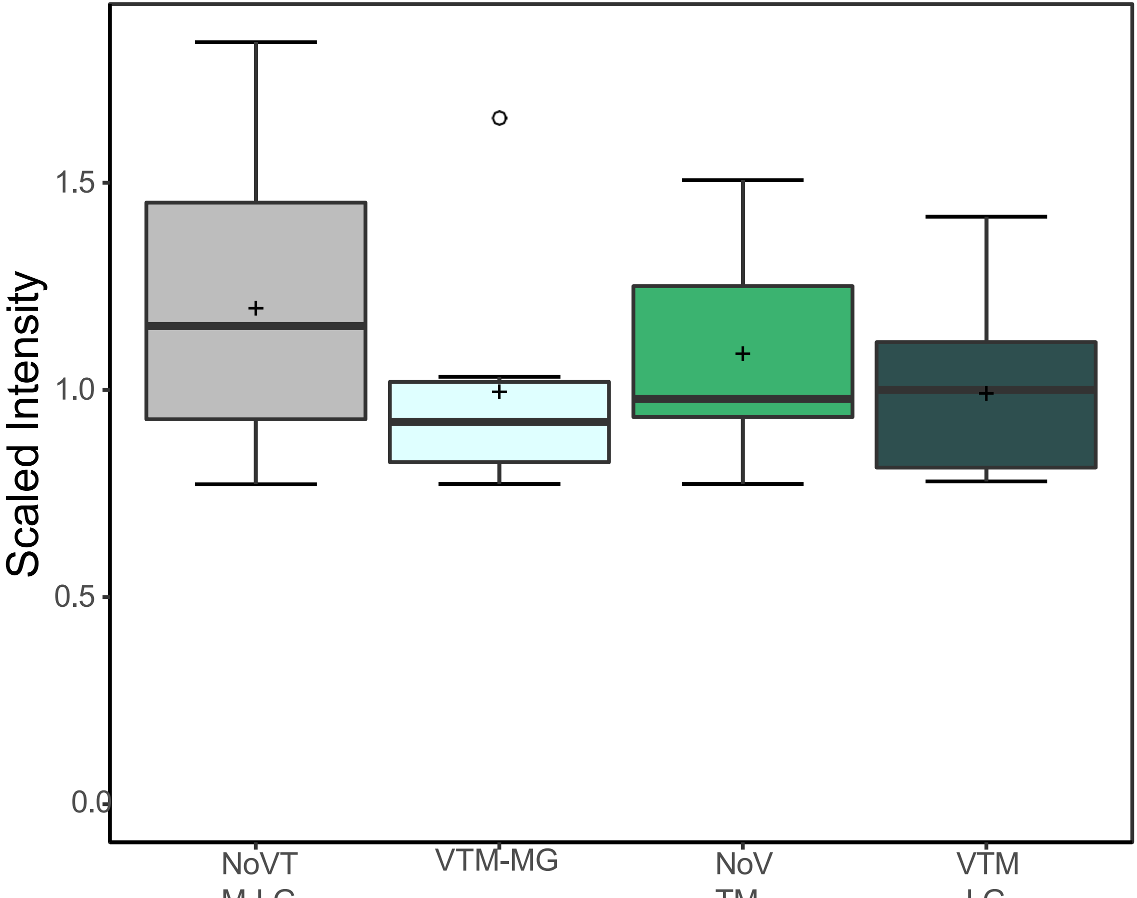

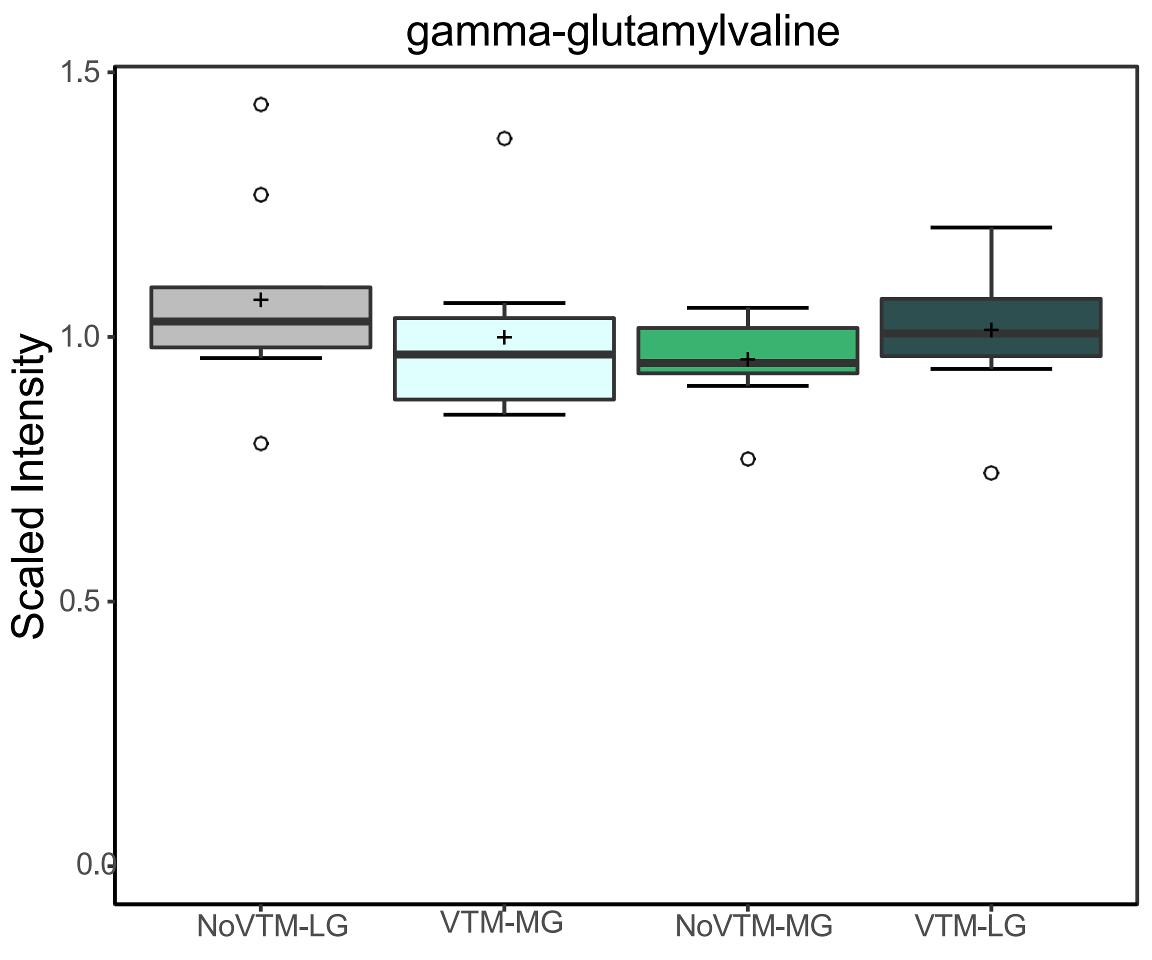

gamma-glutamylvaline
Scaled Intensity
1.5
1.0
0.5
0.0
NoVTM-LG
VTM-MG
NoVTM-MG
VTM-LG

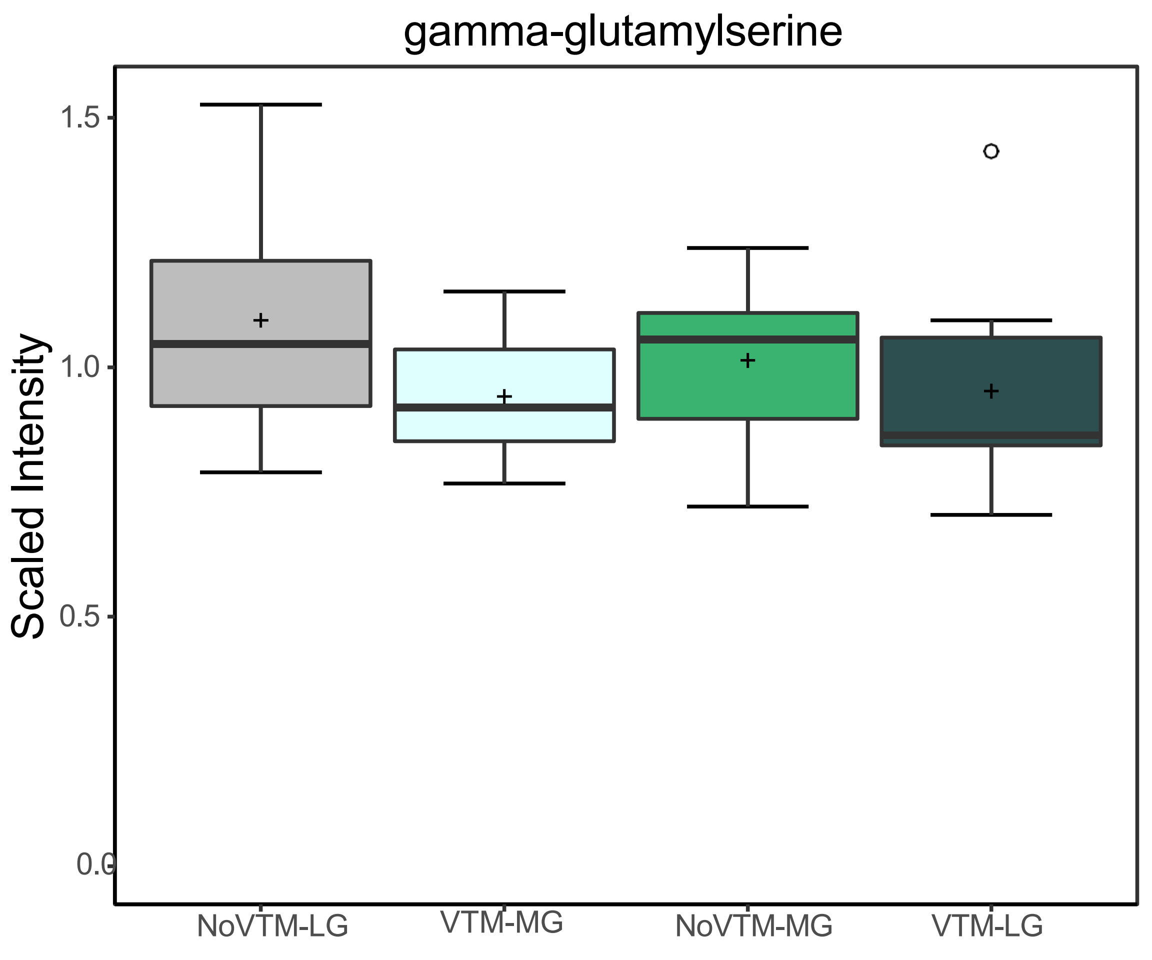
gamma-glutamylserine
Scaled Intensity
1.5
1.0
0.5
0.0
NoVTM-LG
VTM-MG
NoVTM-MG
VTM-LG

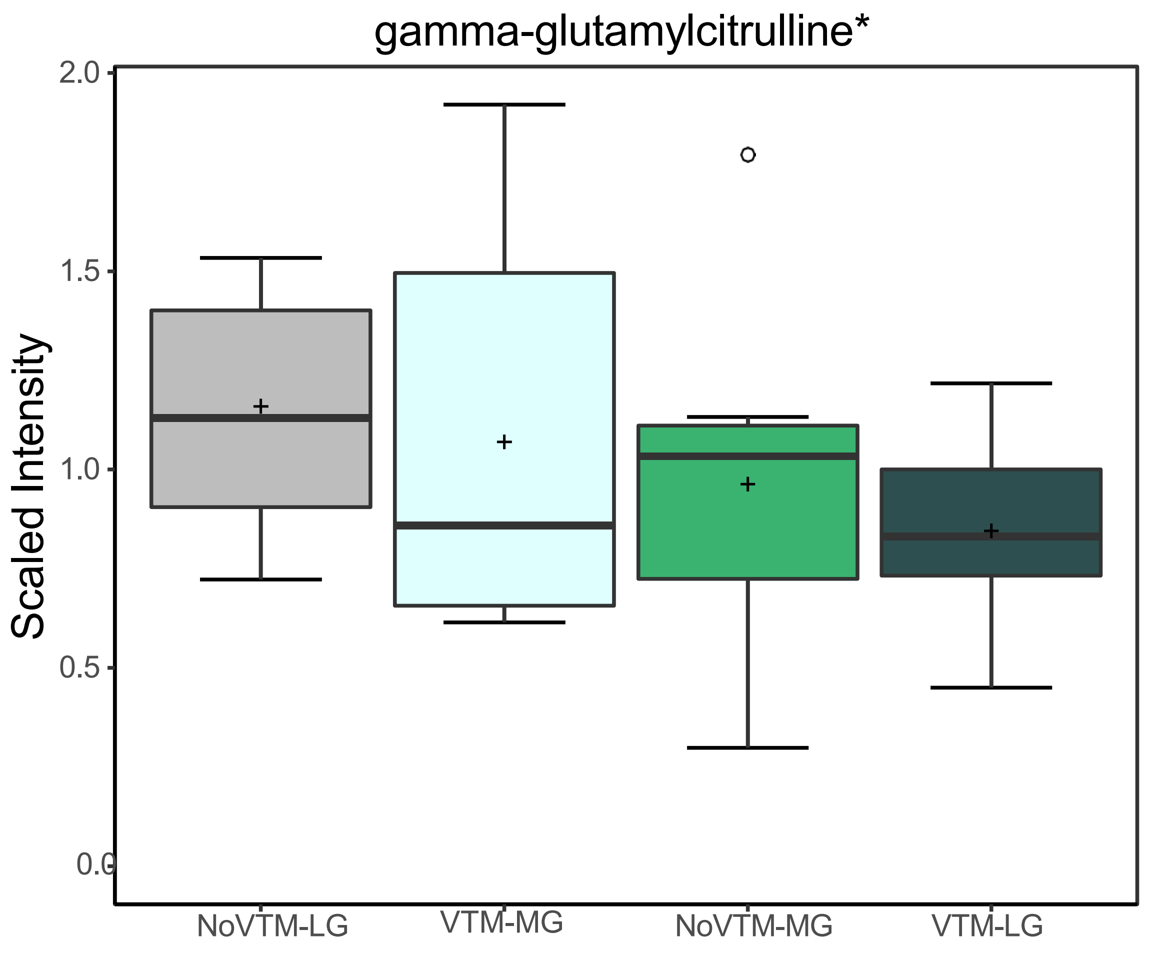
gamma-glutamylcitrulline*
Scaled Intensity
2.0
1.5
1.0
0.5
0.0
NoVTM-LG
VTM-MG
NoVTM-MG
VTM-LG

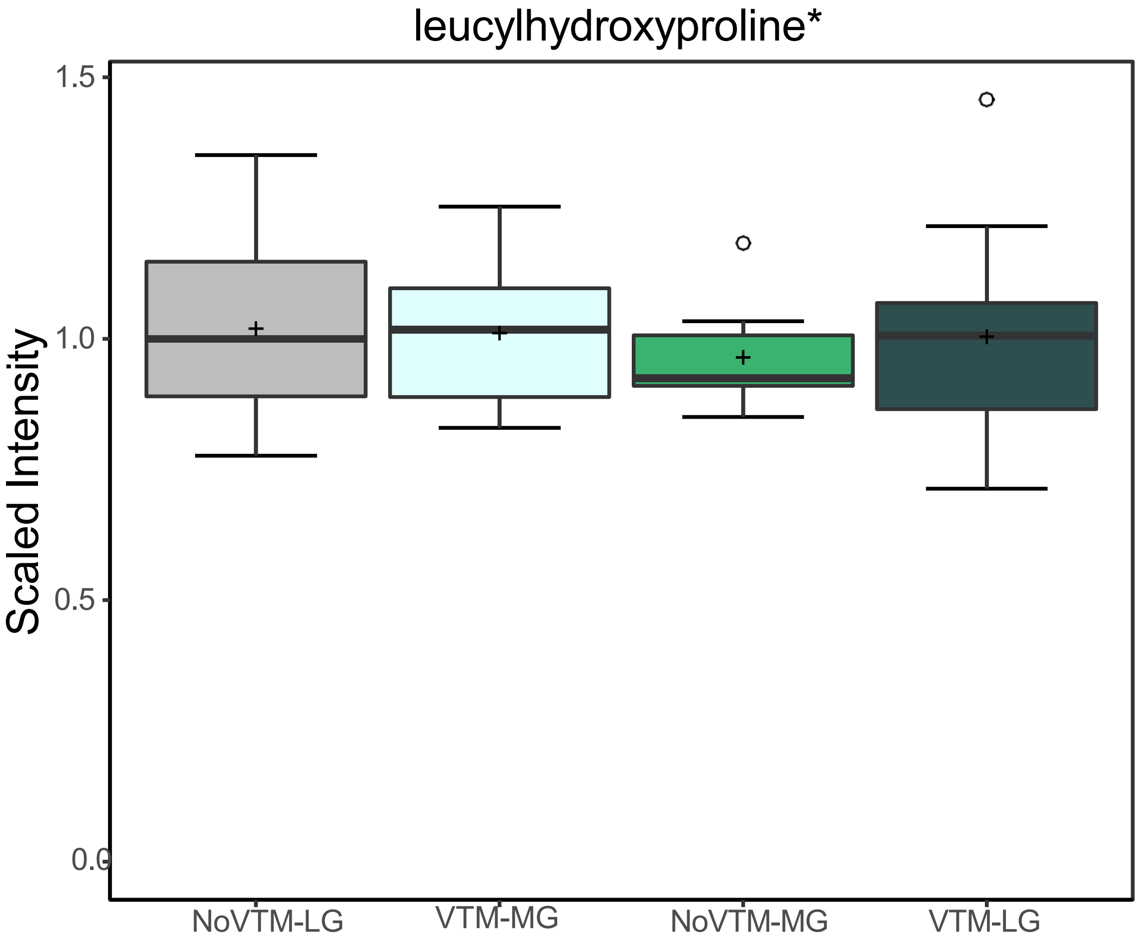

leucylhydroxyproline*
Scaled Intensity
1.5
1.0
0.5
0.0
NoVTM-LG
VTM-MG
NoVTM-MG
VTM-LG

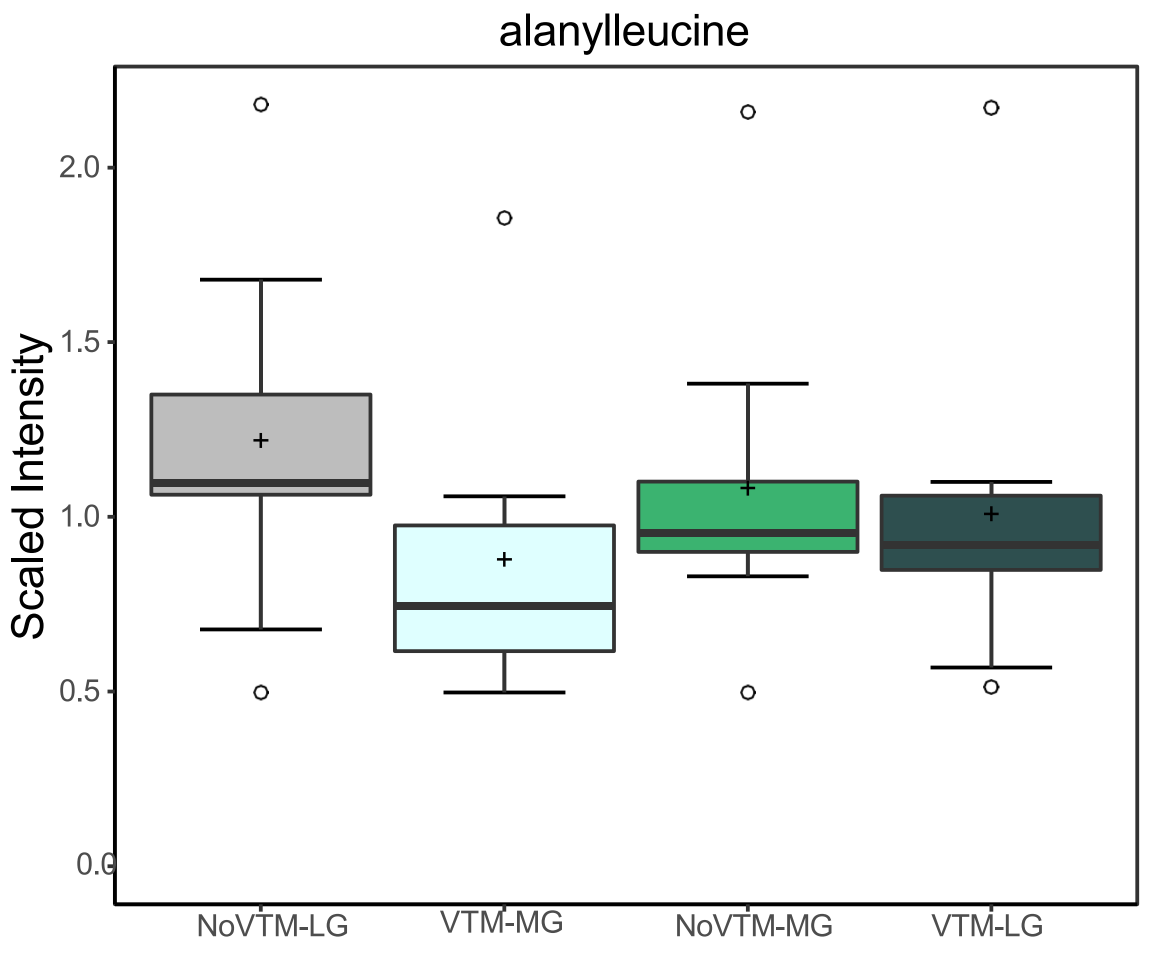
alanylleucine
Scaled Intensity
2.0
1.5
1.0
0.5
0.0
NoVTM-LG
VTM-MG
NoVTM-MG
VTM-LG

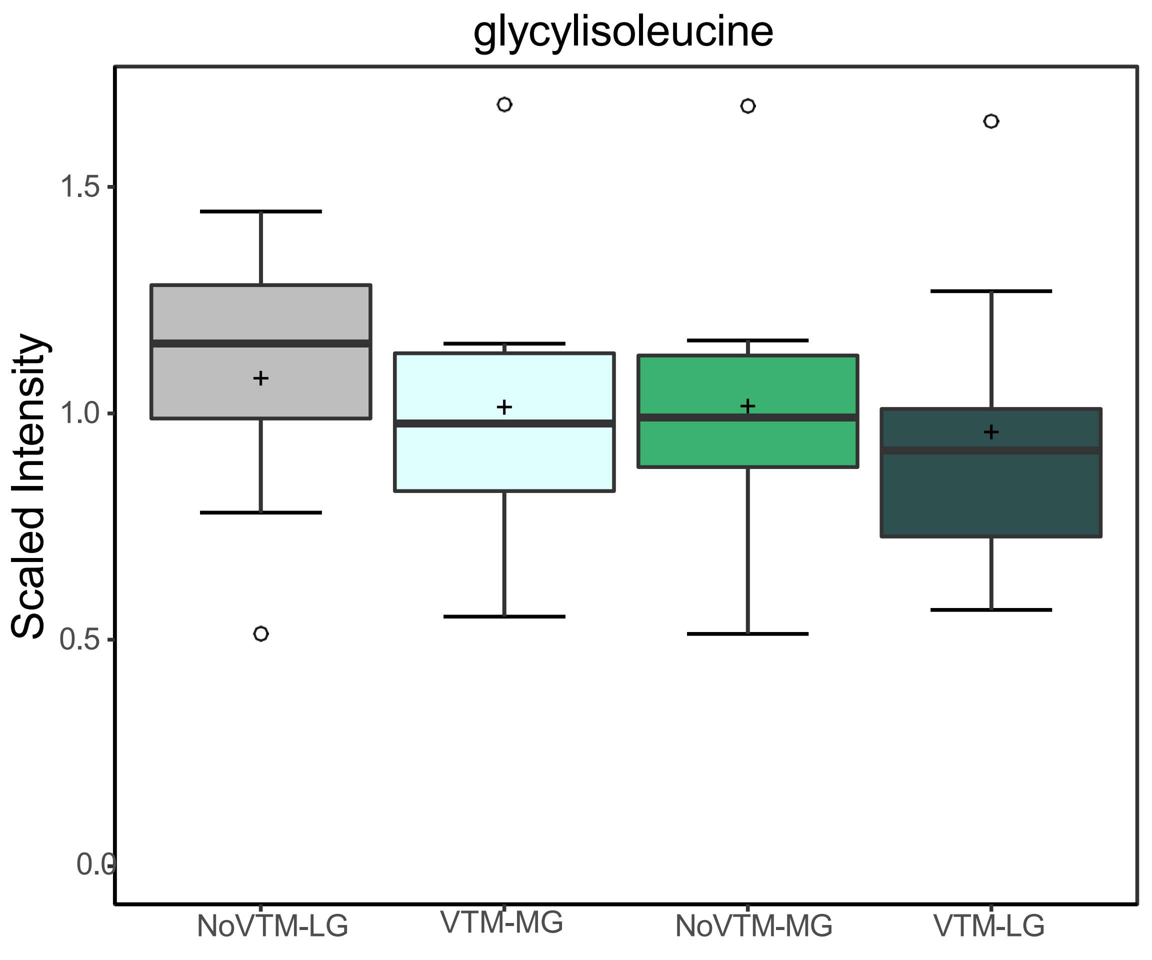

glycylisoleucine
Scaled Intensity
1.5
1.0
0.5
0.0
NoVTM-LG
VTM-MG
NoVTM-MG
VTM-LG

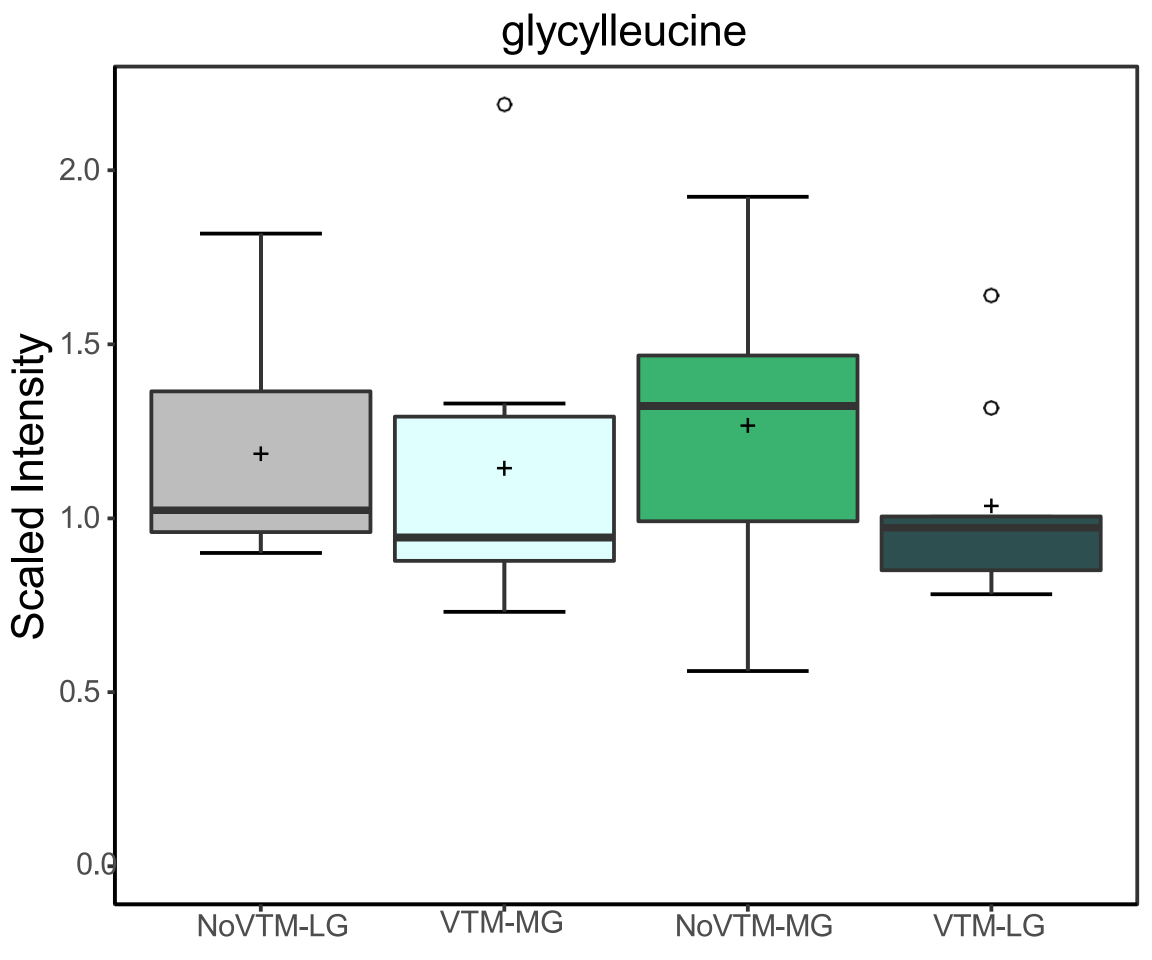
glycylleucine
Scaled Intensity
2.0
1.5
1.0
0.5
0.0
NoVTM-LG
VTM-MG
NoVTM-MG
VTM-LG

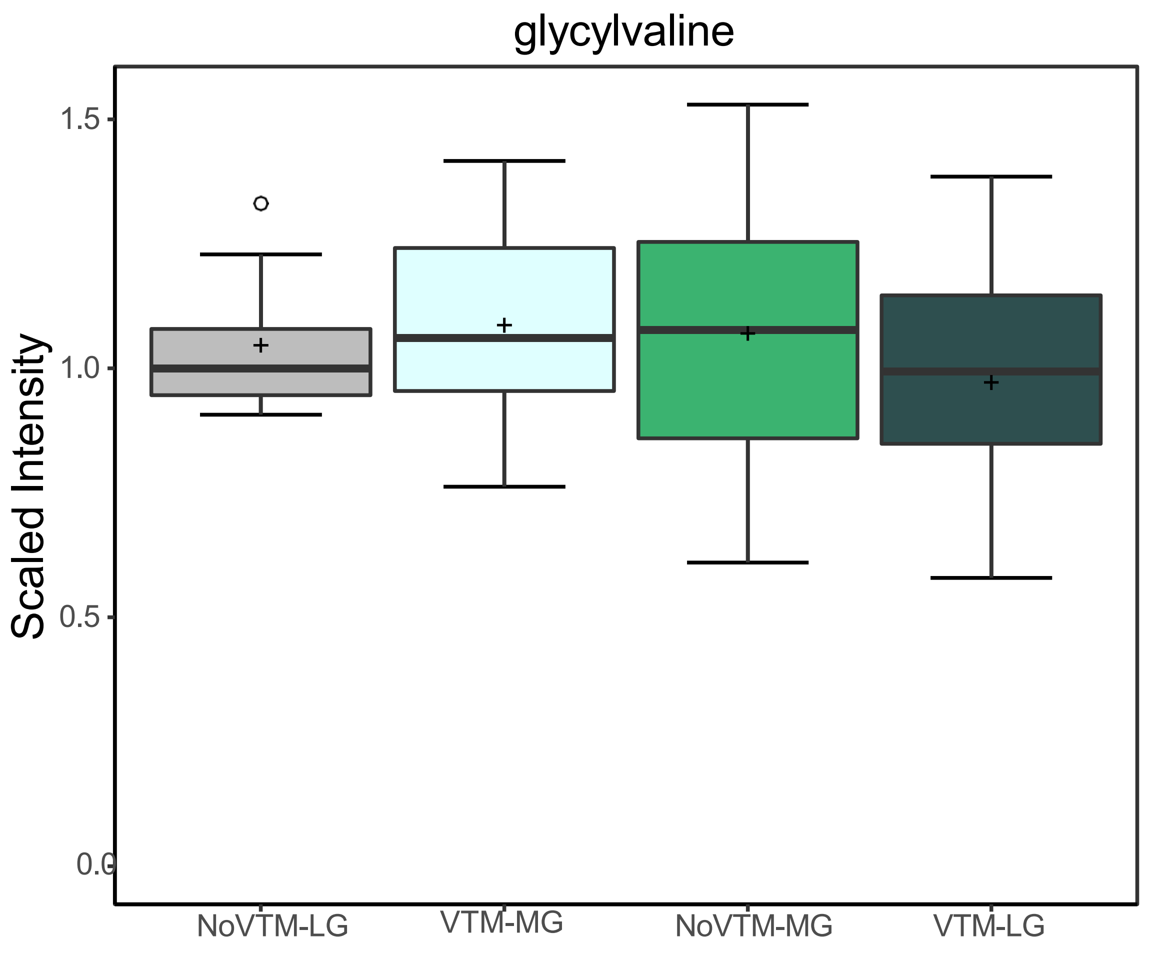

glycylvaline
Scaled Intensity
1.5
1.0
0.5
0.0
NoVTM-LG
VTM-MG
NoVTM-MG
VTM-LG

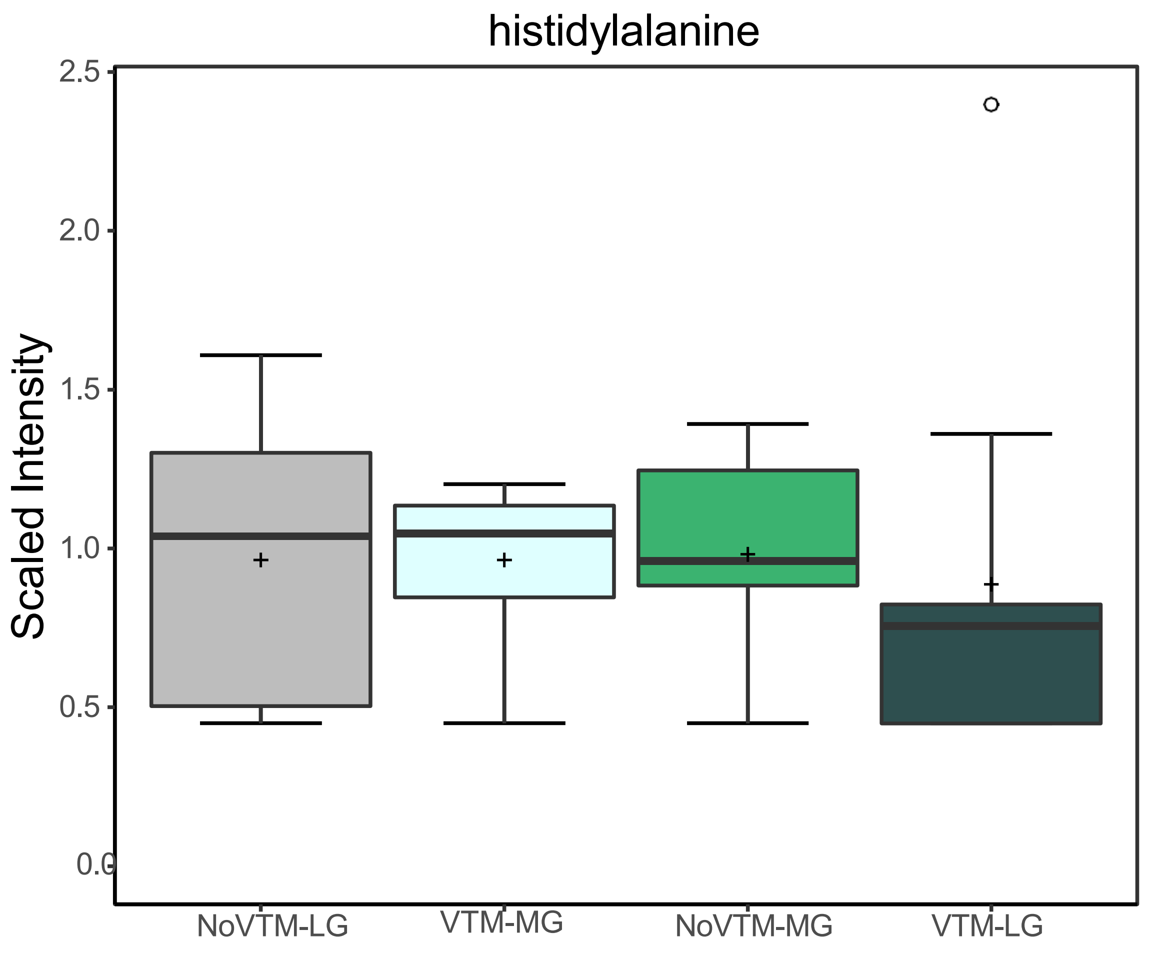
histidylalanine
Scaled Intensity
2.5
2.0
1.5
1.0
0.5
0.0
NoVTM-LG
VTM-MG
NoVTM-MG
VTM-LG

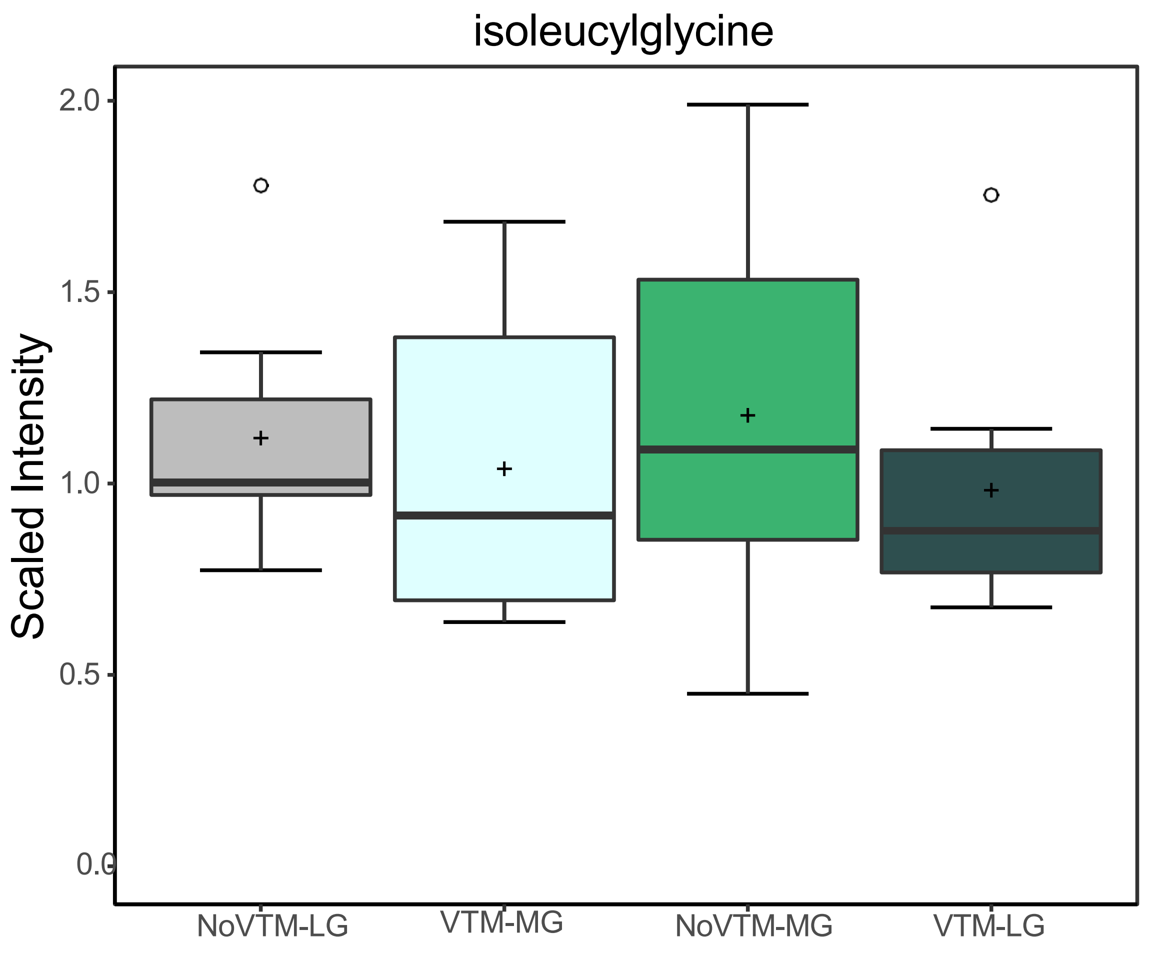

isoleucylglycine
Scaled Intensity
2.0
1.5
1.0
0.5
0.0
NoVTM-LG
VTM-MG
NoVTM-MG
VTM-LG

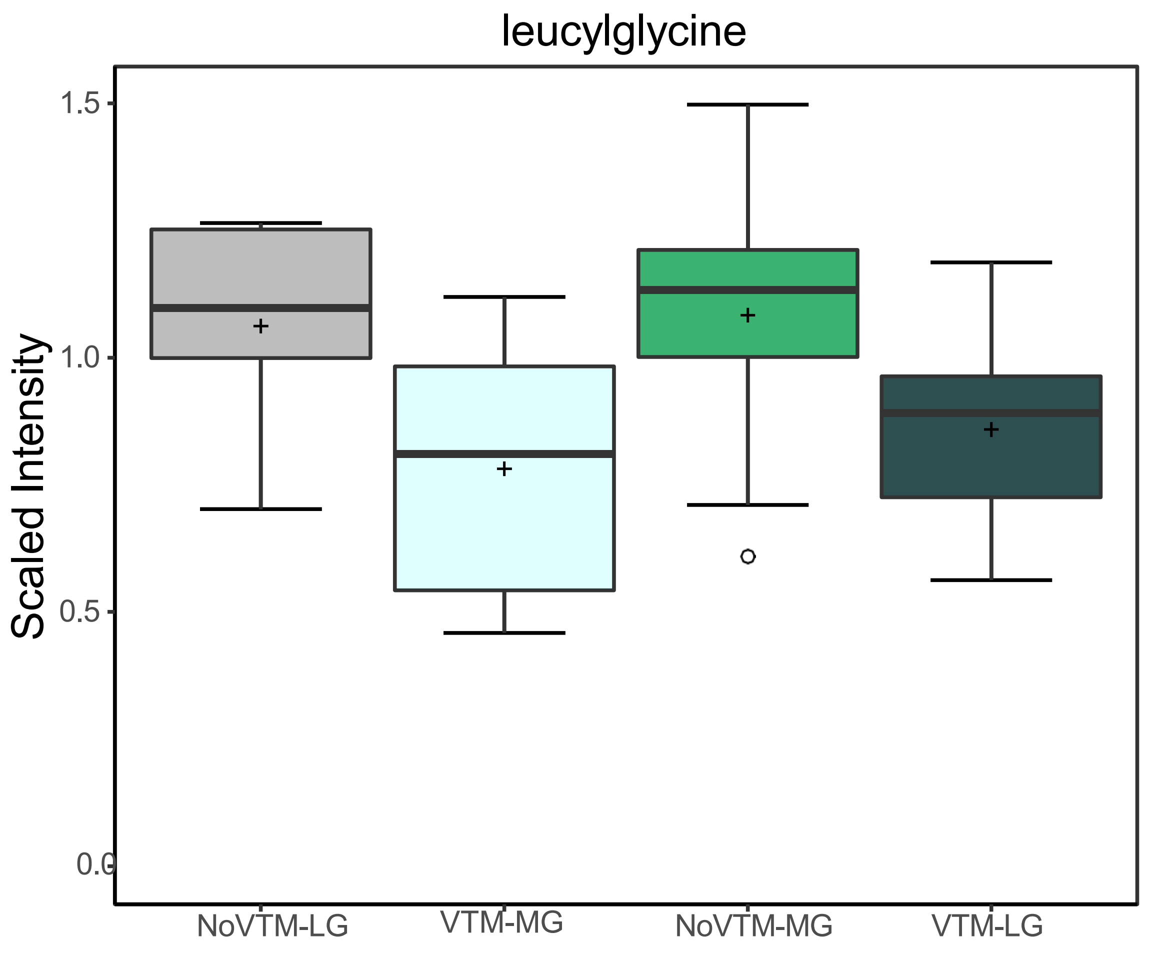
leucylglycine
Scaled Intensity
1.5
1.0
0.5
0.0
NoVTM-LG
VTM-MG
NoVTM-MG
VTM-LG

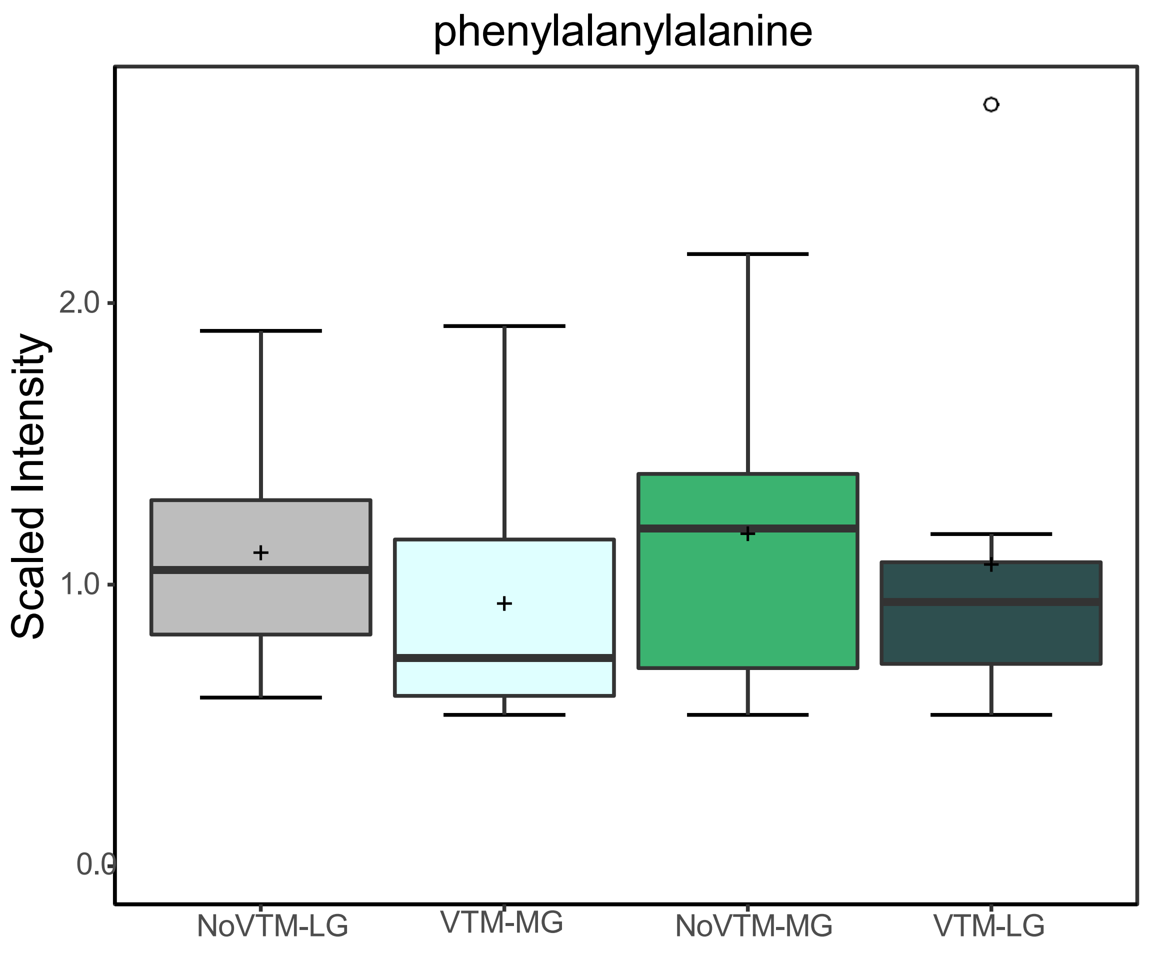

phenylalanylalanine
Scaled Intensity
2.0
1.0
0.0
NoVTM-LG
VTM-MG
NoVTM-MG
VTM-LG

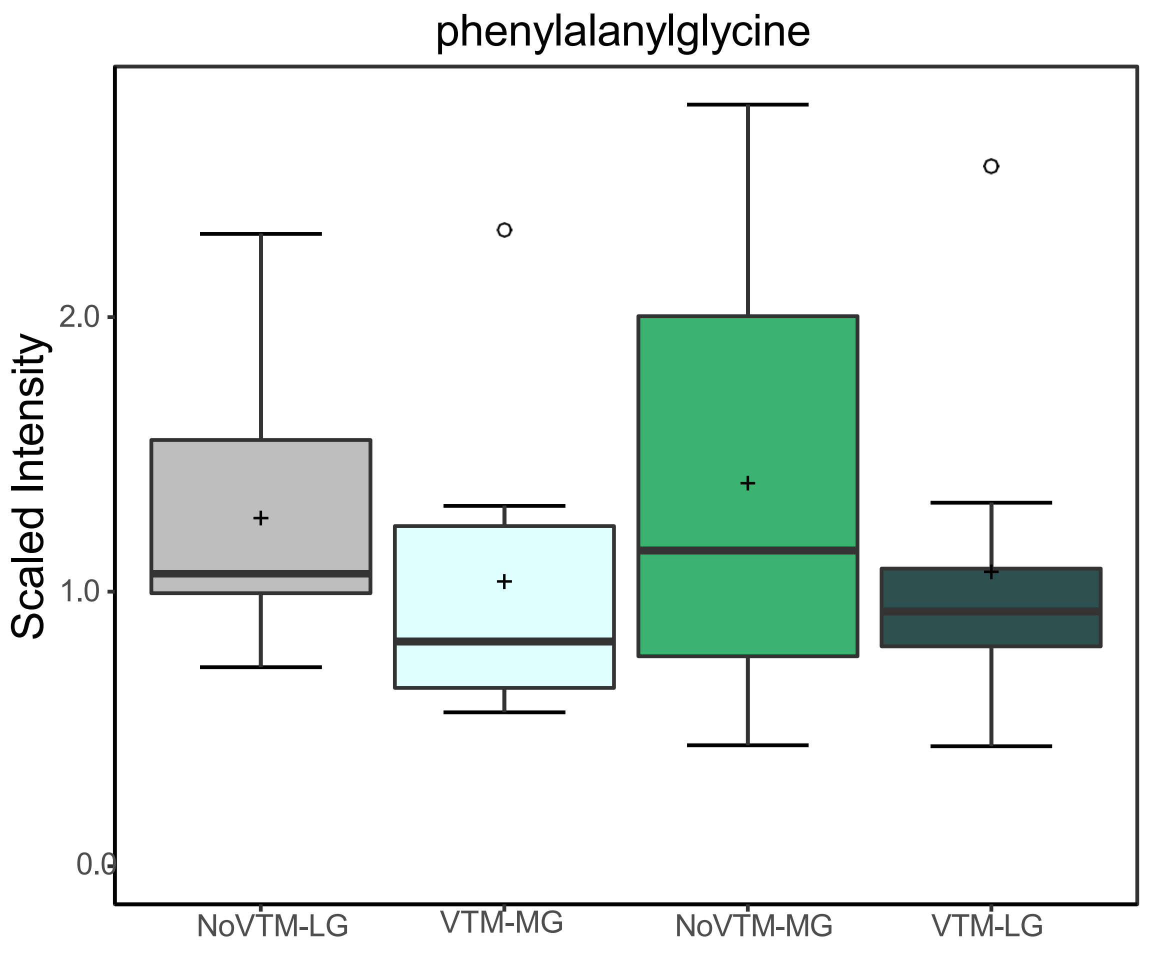

phenylalanylglycine
Scaled Intensity
2.0
1.0
0.0
NoVTM-LG
VTM-MG
NoVTM-MG
VTM-LG

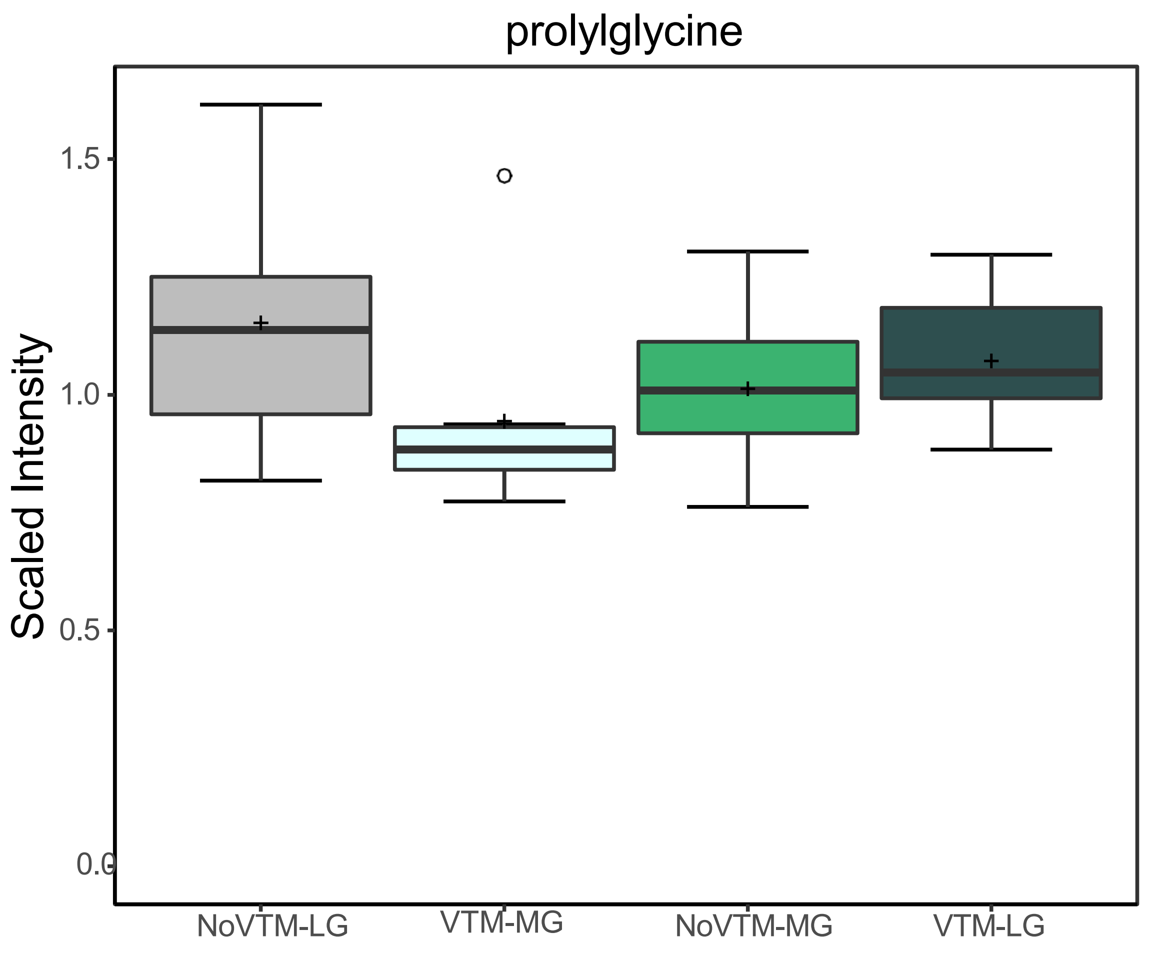
prolylglycine
Scaled Intensity
1.5
1.0
0.5
0.0
NoVTM-LG
VTM-MG
NoVTM-MG
VTM-LG

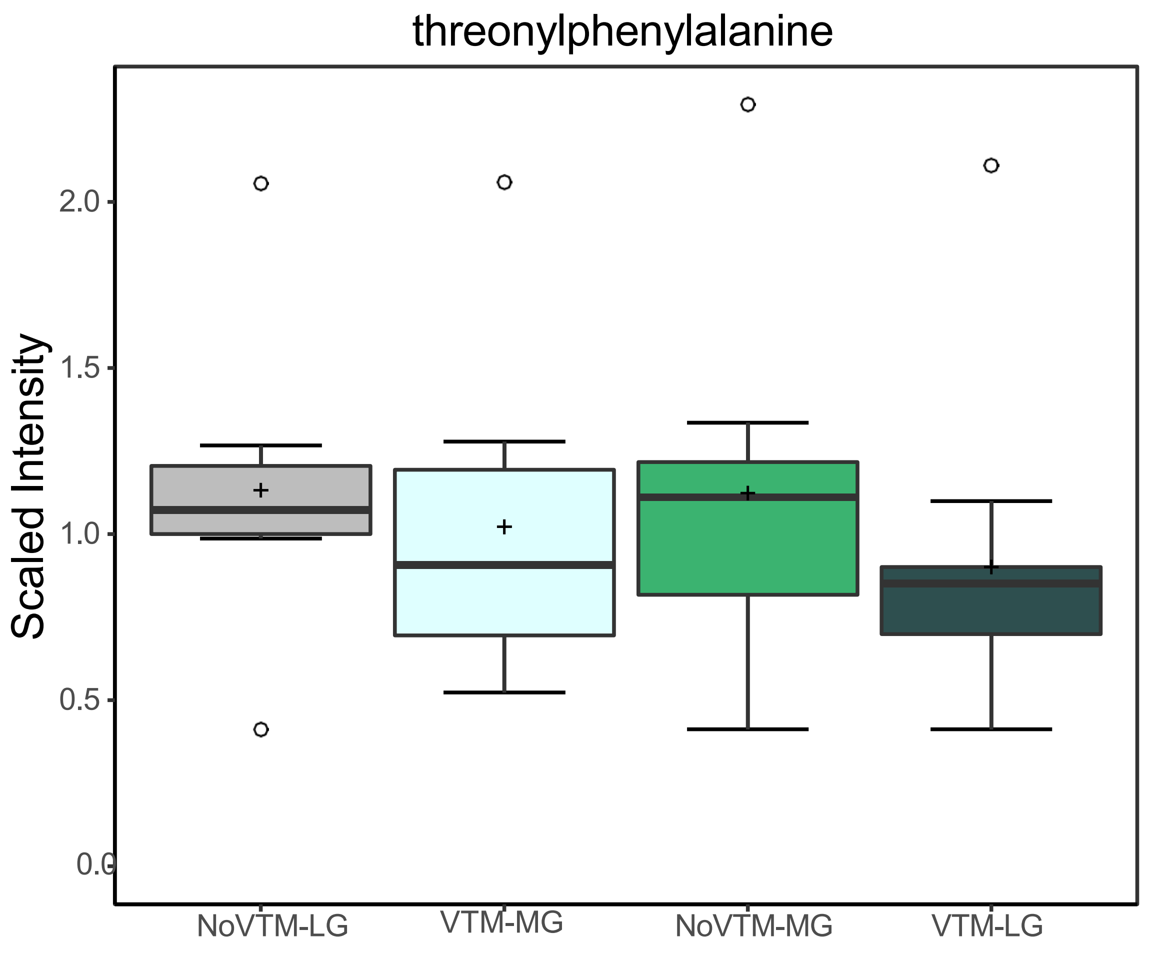
threonylphenylalanine
Scaled Intensity
2.0
1.5
1.0
0.5
0.0
NoVTM-LG
VTM-MG
NoVTM-MG
VTM-LG

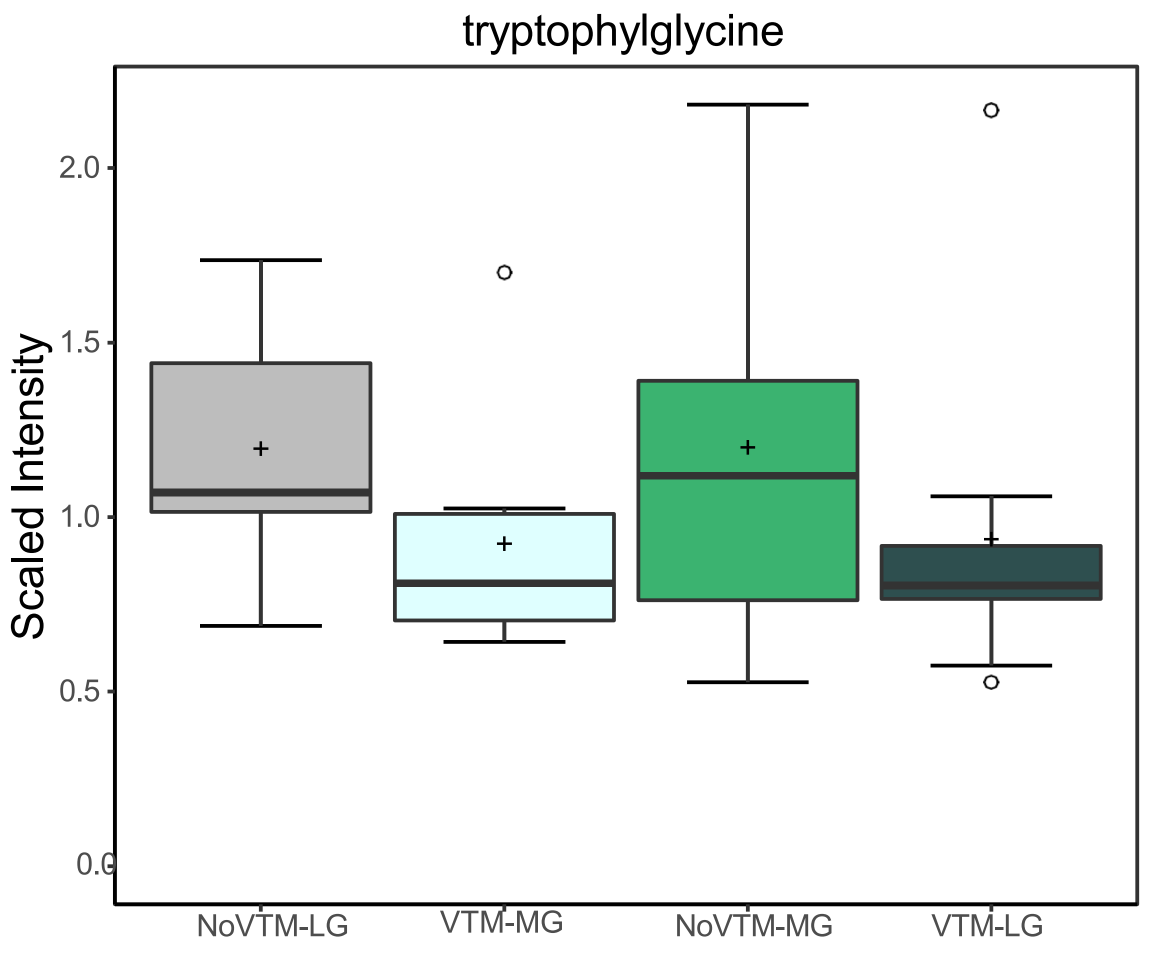
tryptophylglycine
Scaled Intensity
2.0
1.5
1.0
0.5
0.0
NoVTM-LG
VTM-MG
NoVTM-MG
VTM-LG

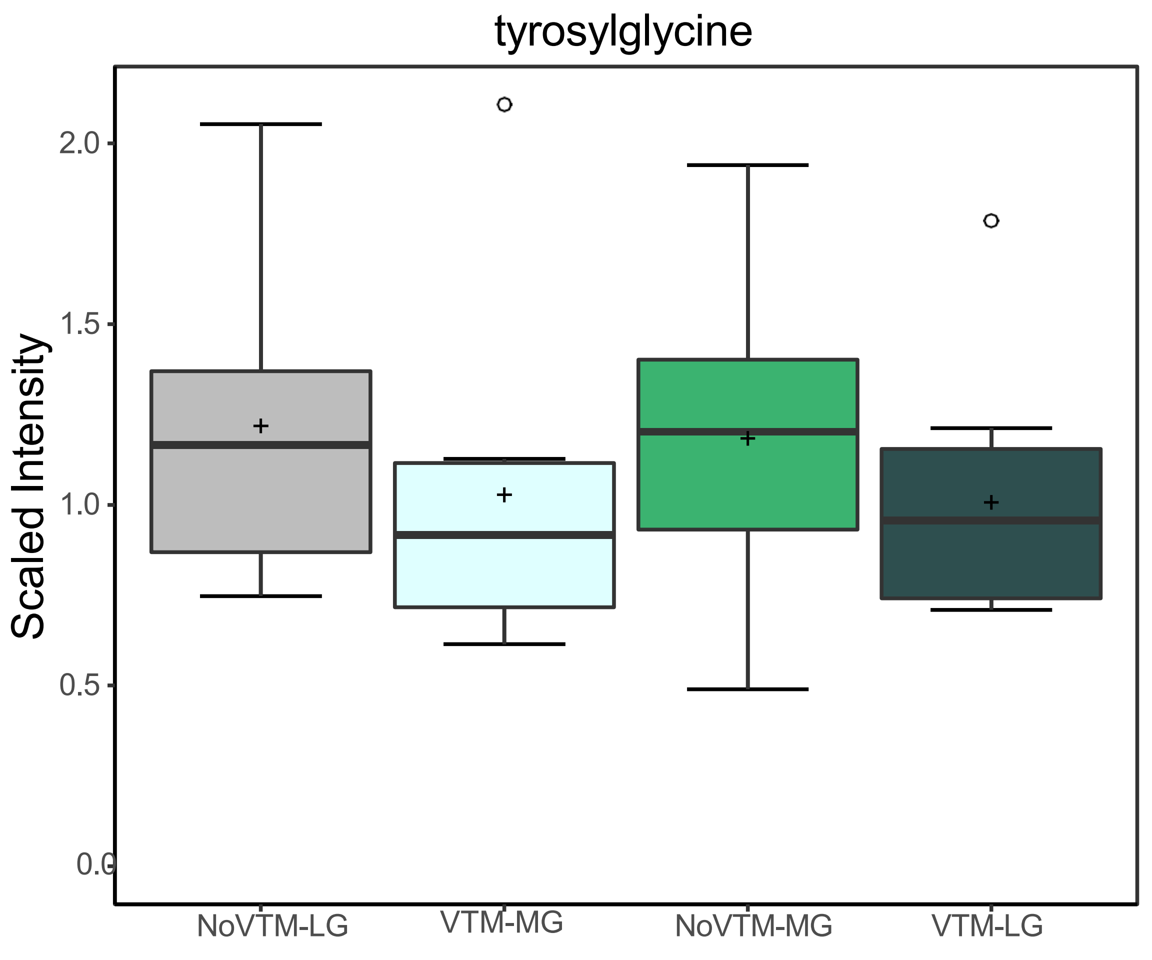
tyrosylglycine
Scaled Intensity
2.0
1.5
1.0
0.5
0.0
NoVTM-LG
VTM-MG
NoVTM-MG
VTM-LG

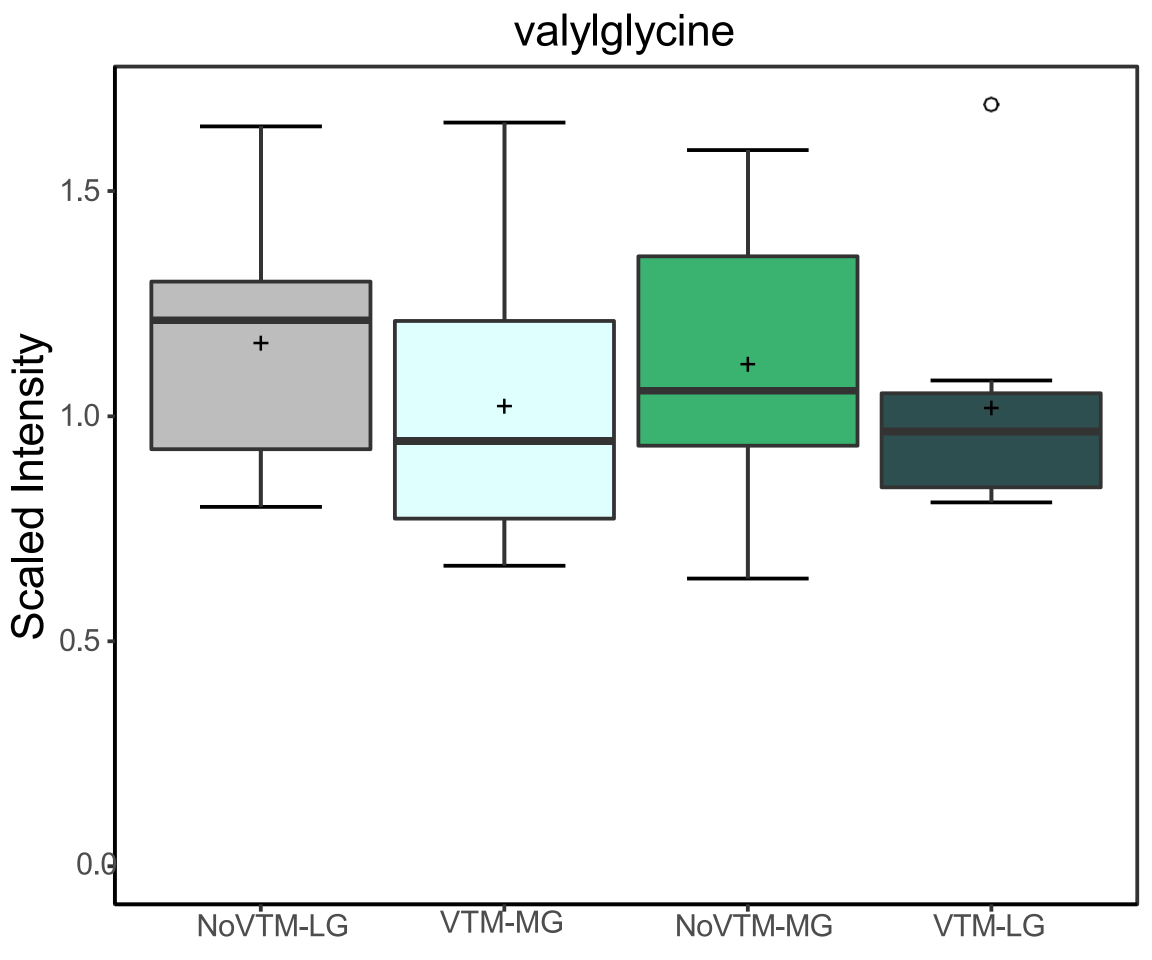
valylglycine
Scaled Intensity
1.5
1.0
0.5
0.0
NoVTM-LG
VTM-MG
NoVTM-MG
VTM-LG

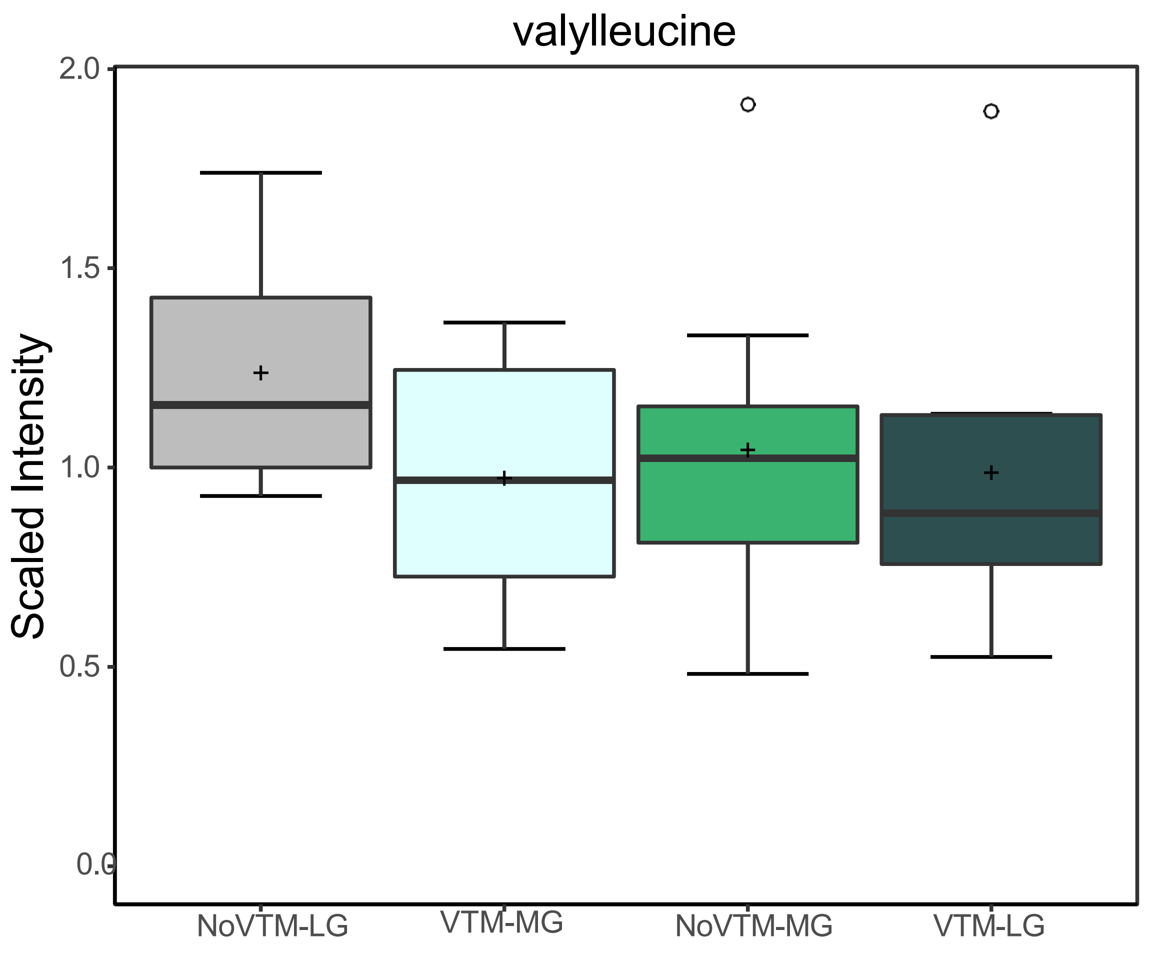
valylleucine
Scaled Intensity
2.0
1.5
1.0
0.5
0.0
NoVTM-LG
VTM-MG
NoVTM-MG
VTM-LG

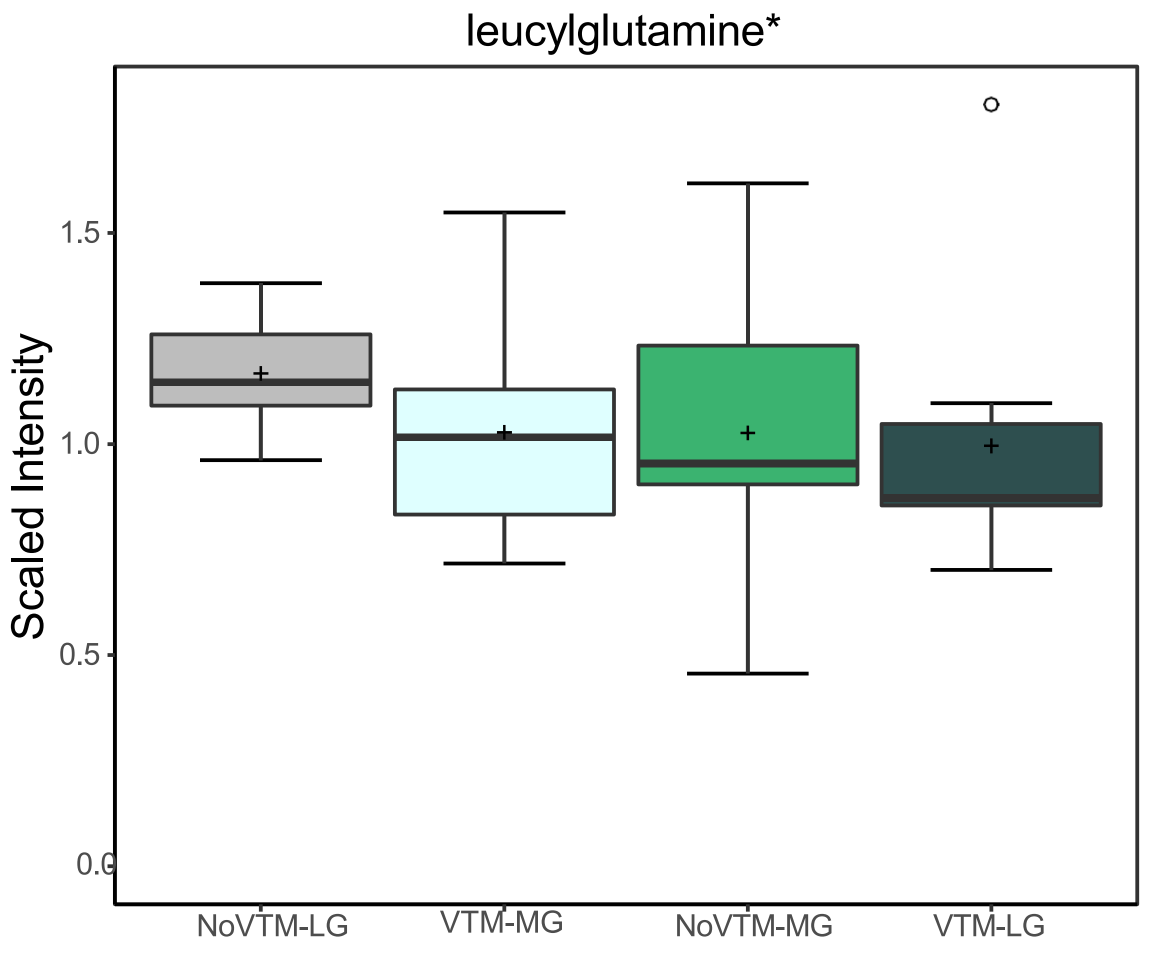

leucylglutamine*
Scaled Intensity
1.5
1.0
0.5
0.0
NoVTM-LG
VTM-MG
NoVTM-MG
VTM-LG

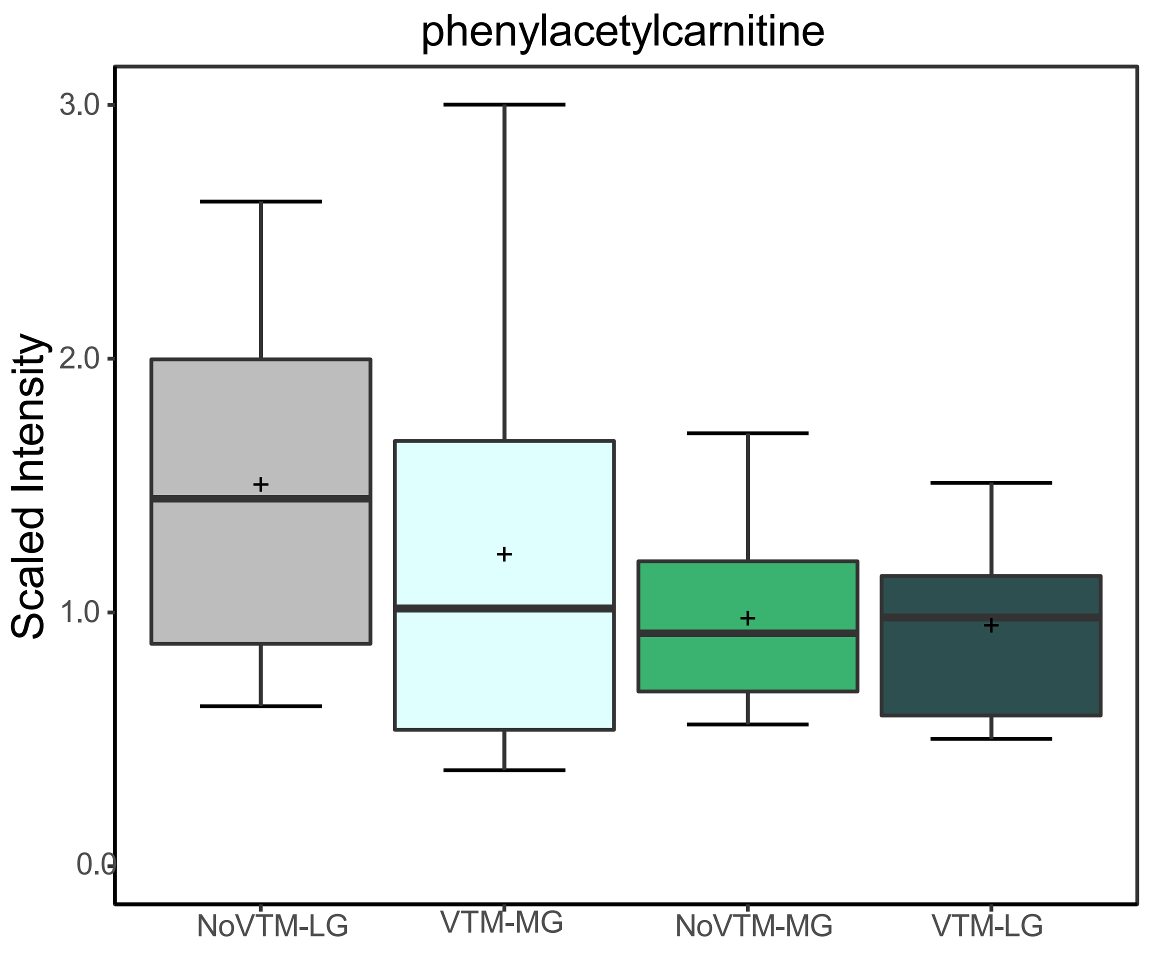
phenylacetylcarnitine
Scaled Intensity
3.0
2.0
1.0
0.0
NoVTM-LG
VTM-MG
NoVTM-MG
VTM-LG

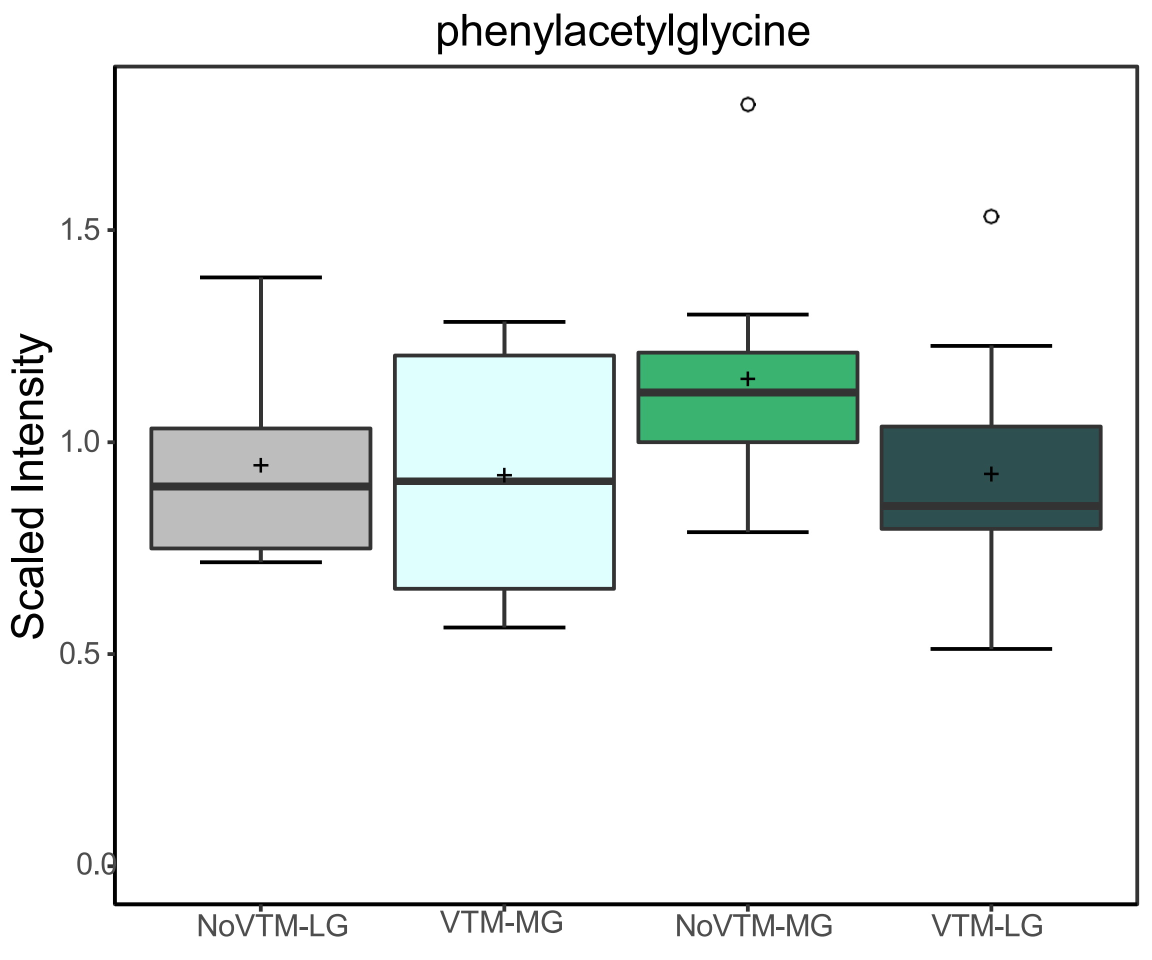
phenylacetylglycine
Scaled Intensity
1.5
1.0
0.5
0.0
NoVTM-LG
VTM-MG
NoVTM-MG
VTM-LG

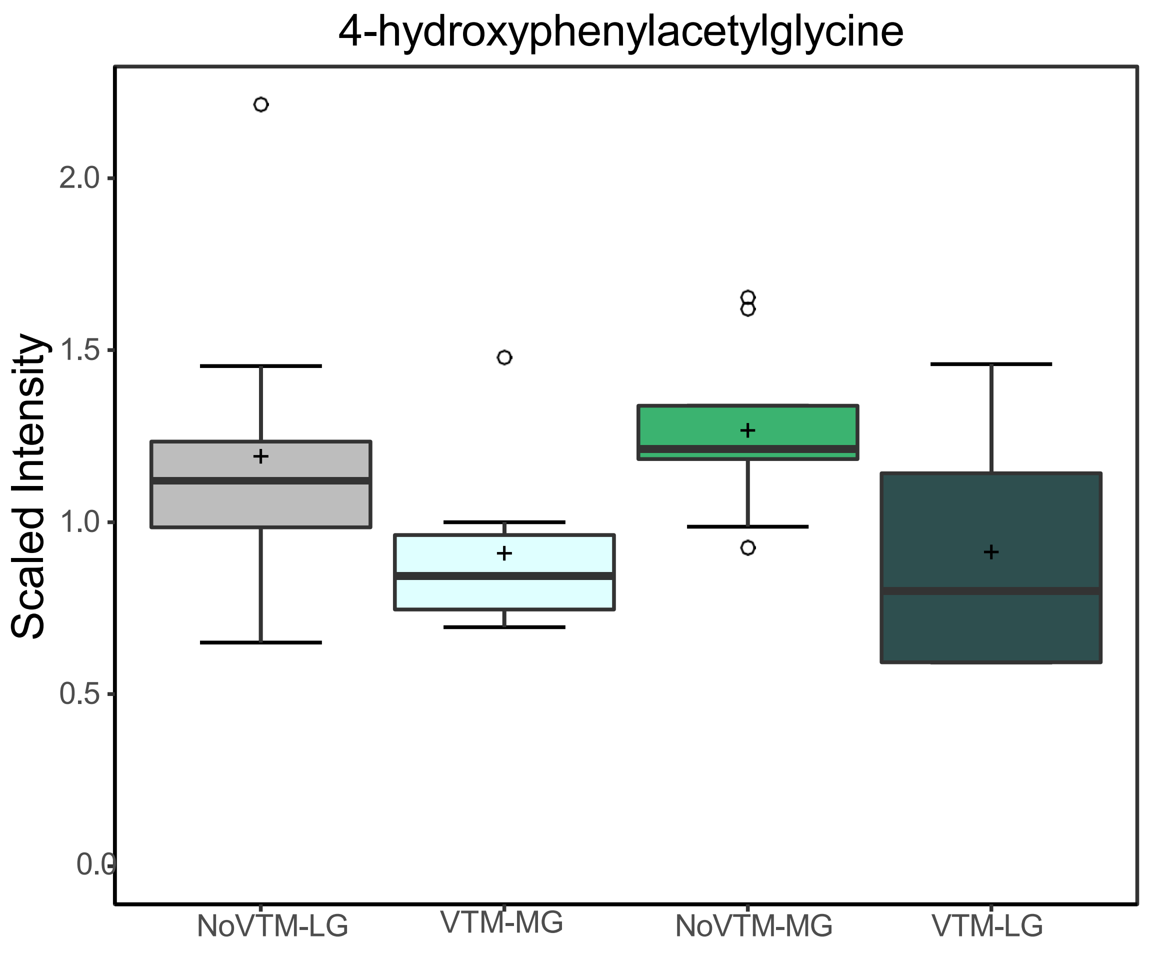
4-hydroxyphenylacetylglycine
Scaled Intensity
2.0
1.5
1.0
0.5
0.0
NoVTM-LG
VTM-MG
NoVTM-MG
VTM-LG

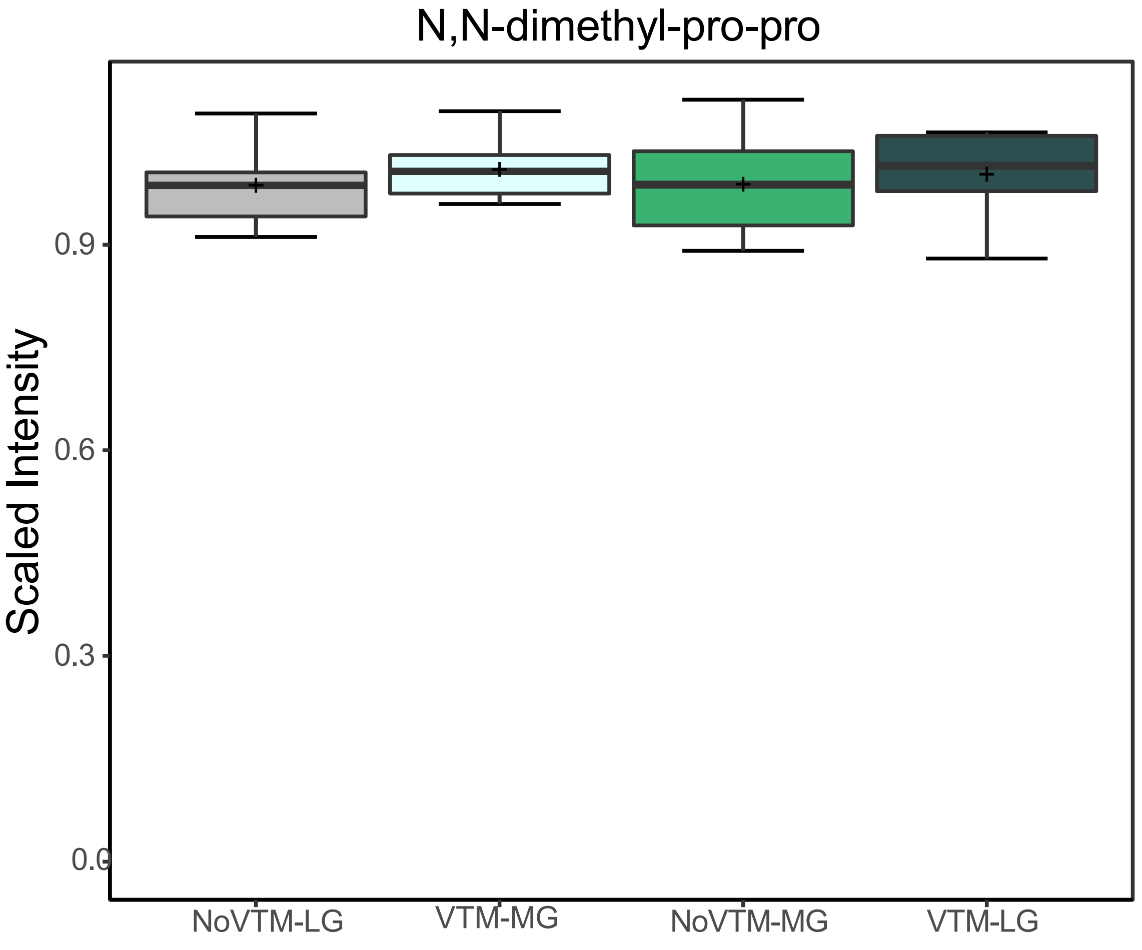
N,N-dimethyl-pro-pro
Scaled Intensity
0.9
0.6
0.3
0.0
NoVTM-LG
VTM-MG
NoVTM-MG
VTM-LG

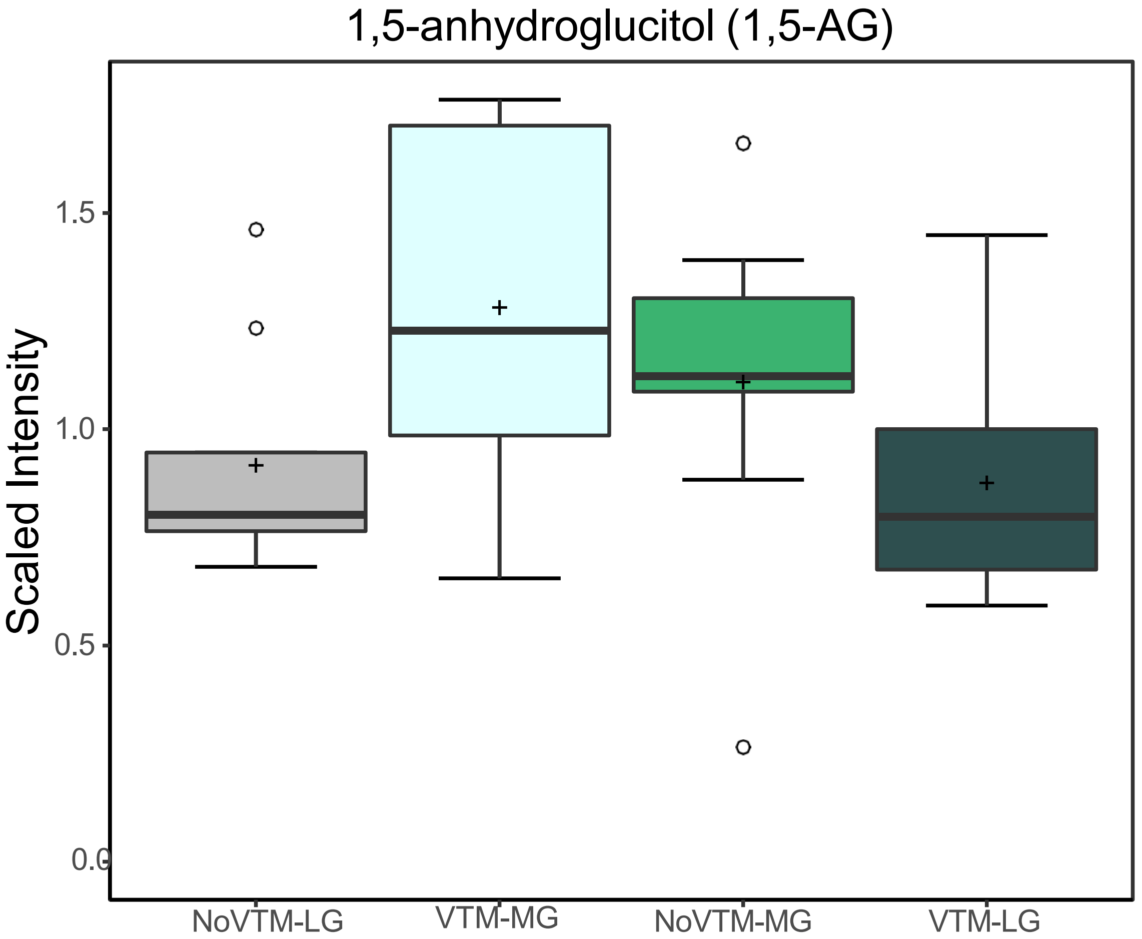
1,5-anhydroglucitol (1,5-AG)
Scaled Intensity
1.5
1.0
0.5
0.0
NoVTM-LG
VTM-MG
NoVTM-MG
VTM-LG

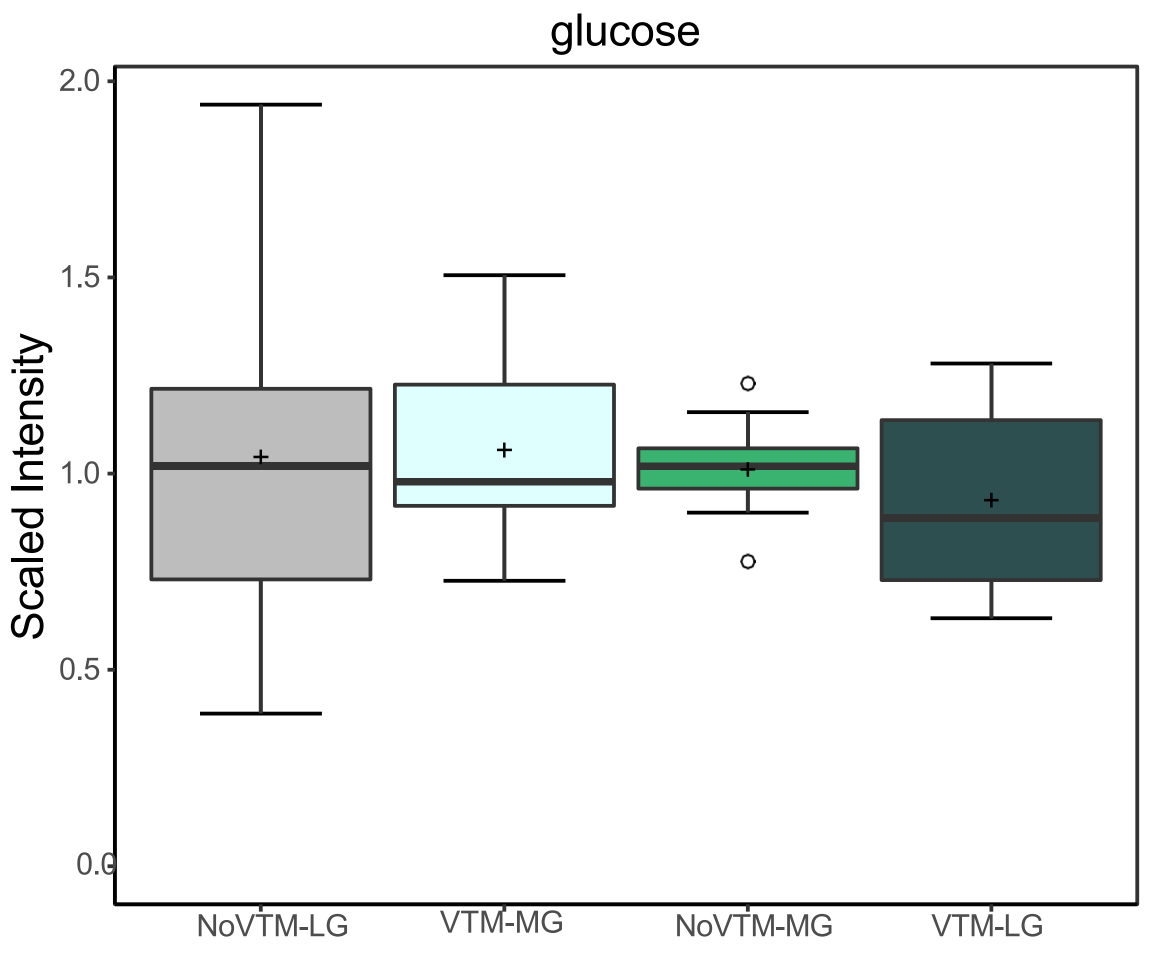
glucose
Scaled Intensity
2.0
1.5
1.0
0.5
0.0
NoVTM-LG
VTM-MG
NoVTM-MG
VTM-LG

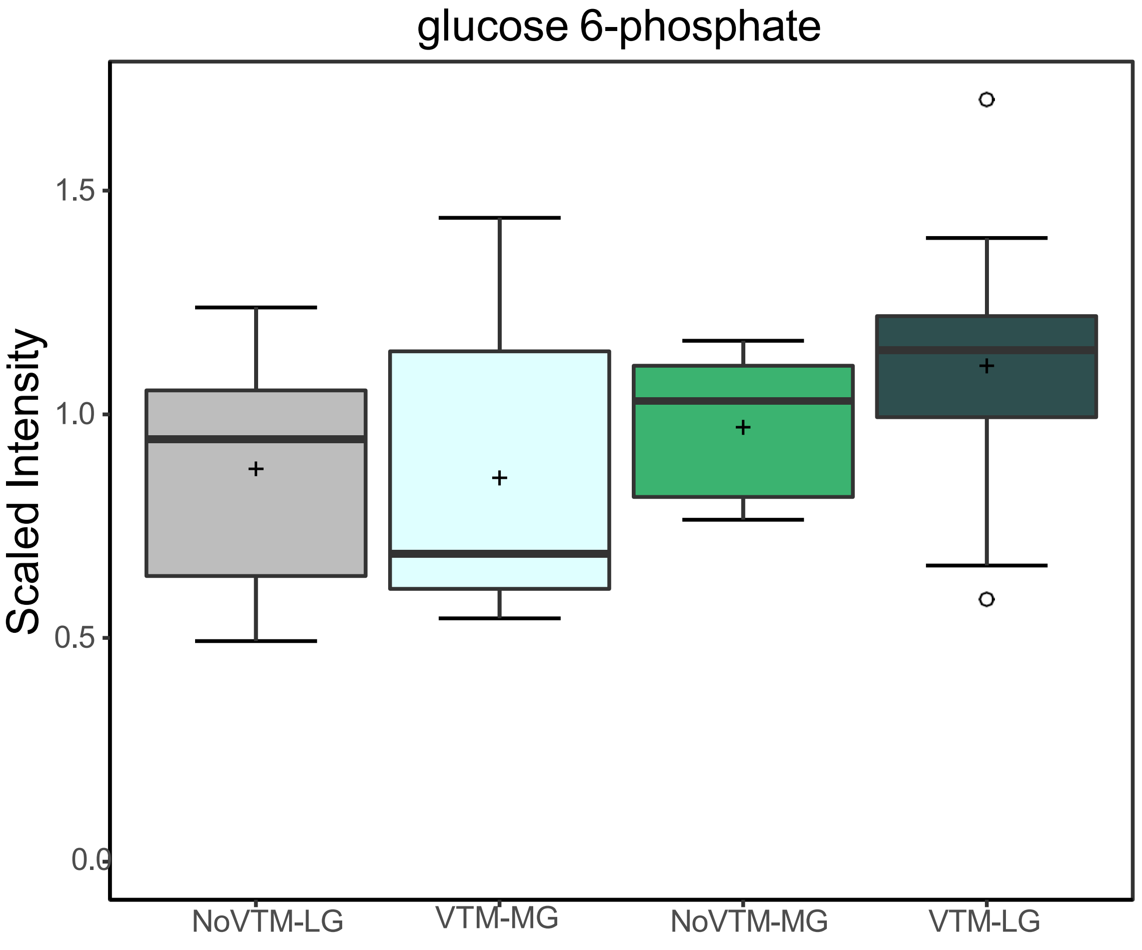
glucose 6-phosphate
Scaled Intensity
1.5
1.0
0.5
0.0
NoVTM-LG
VTM-MG
NoVTM-MG
VTM-LG

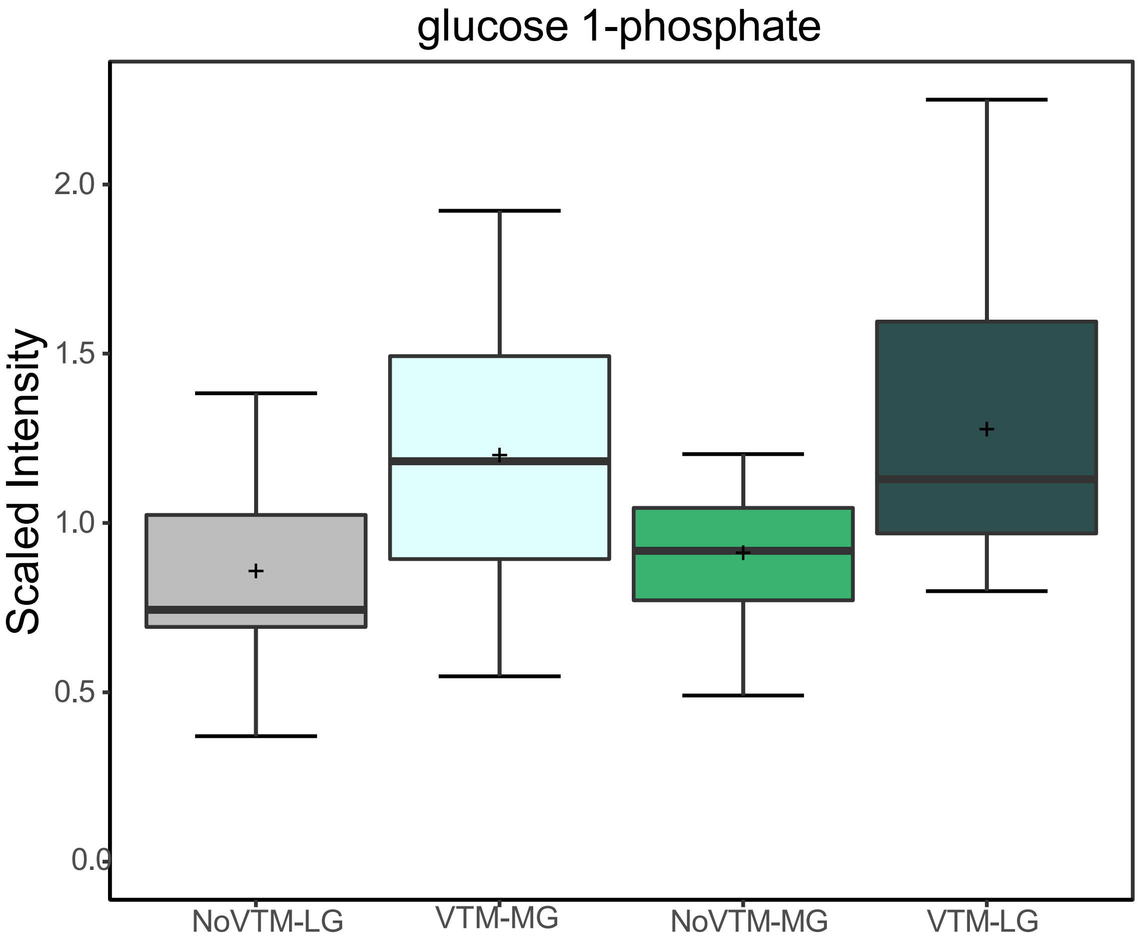

glucose 1-phosphate
Scaled Intensity
2.0
1.5
1.0
0.5
0.0
NoVTM-LG
VTM-MG
NoVTM-MG
VTM-LG

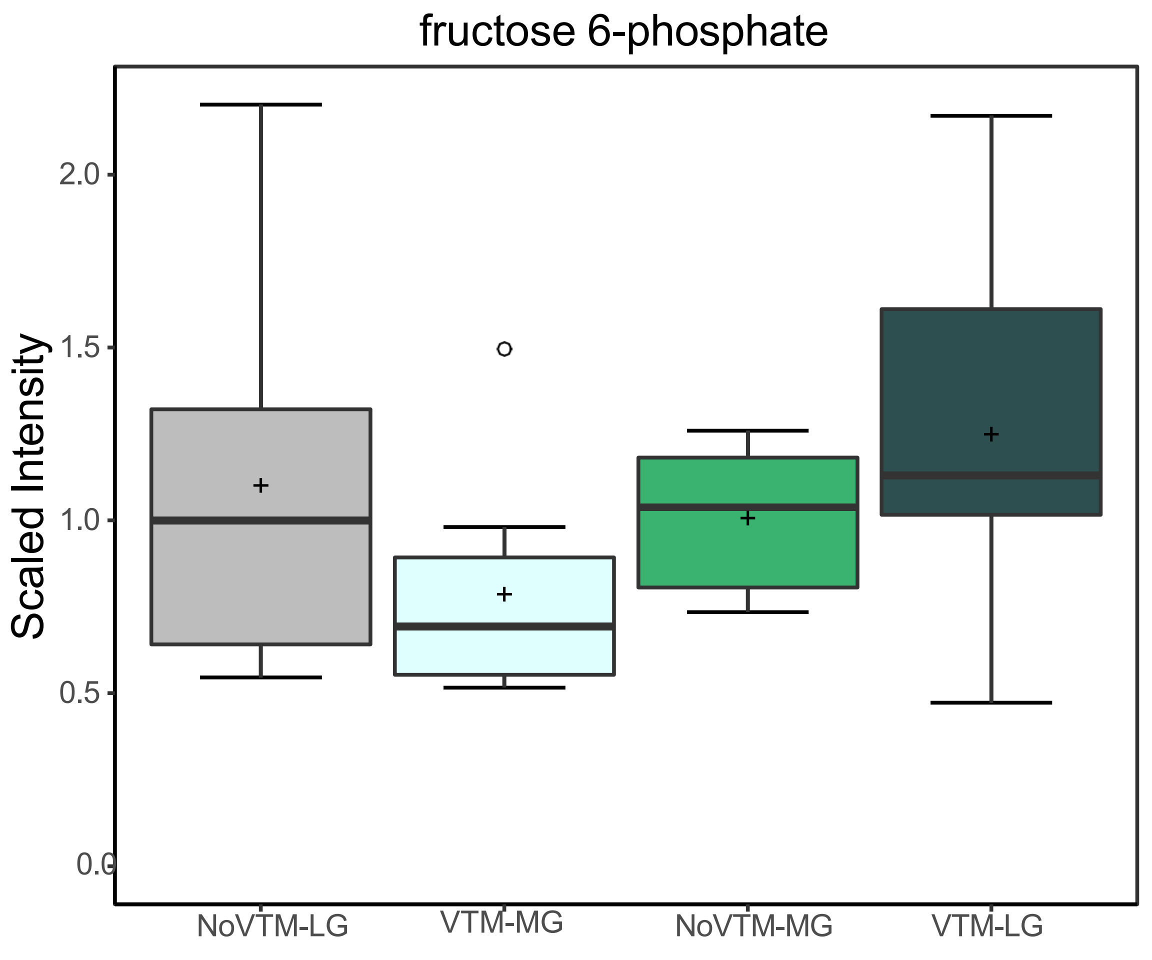

fructose 6-phosphate
Scaled Intensity
2.0
1.5
1.0
0.5
0.0
NoVTM-LG
VTM-MG
NoVTM-MG
VTM-LG

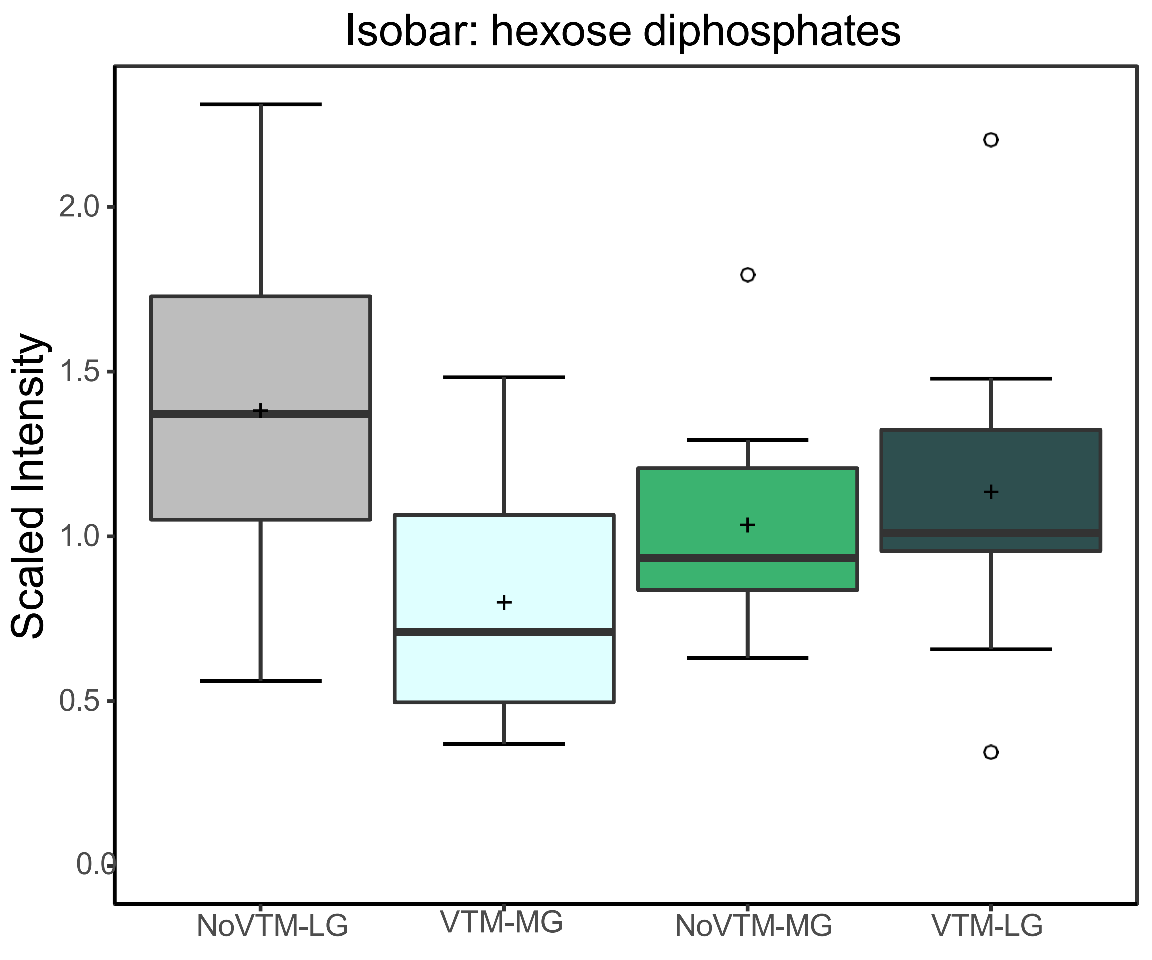
Isobar: hexose diphosphates
Scaled Intensity
2.0
1.5
1.0
0.5
0.0
NoVTM-LG
VTM-MG
NoVTM-MG
VTM-LG

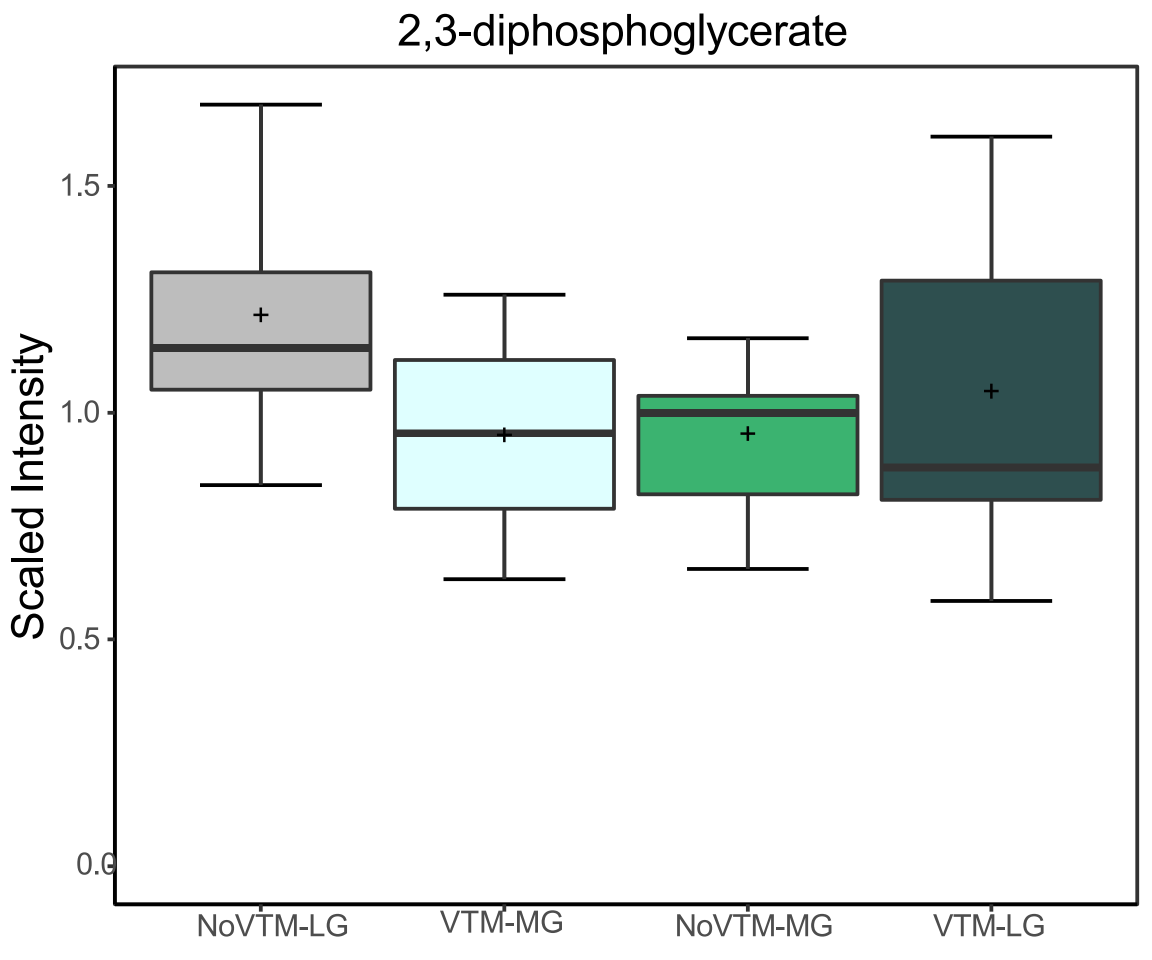
2,3-diphosphoglycerate
Scaled Intensity
1.5
1.0
0.5
0.0
NoVTM-LG
VTM-MG
NoVTM-MG
VTM-LG

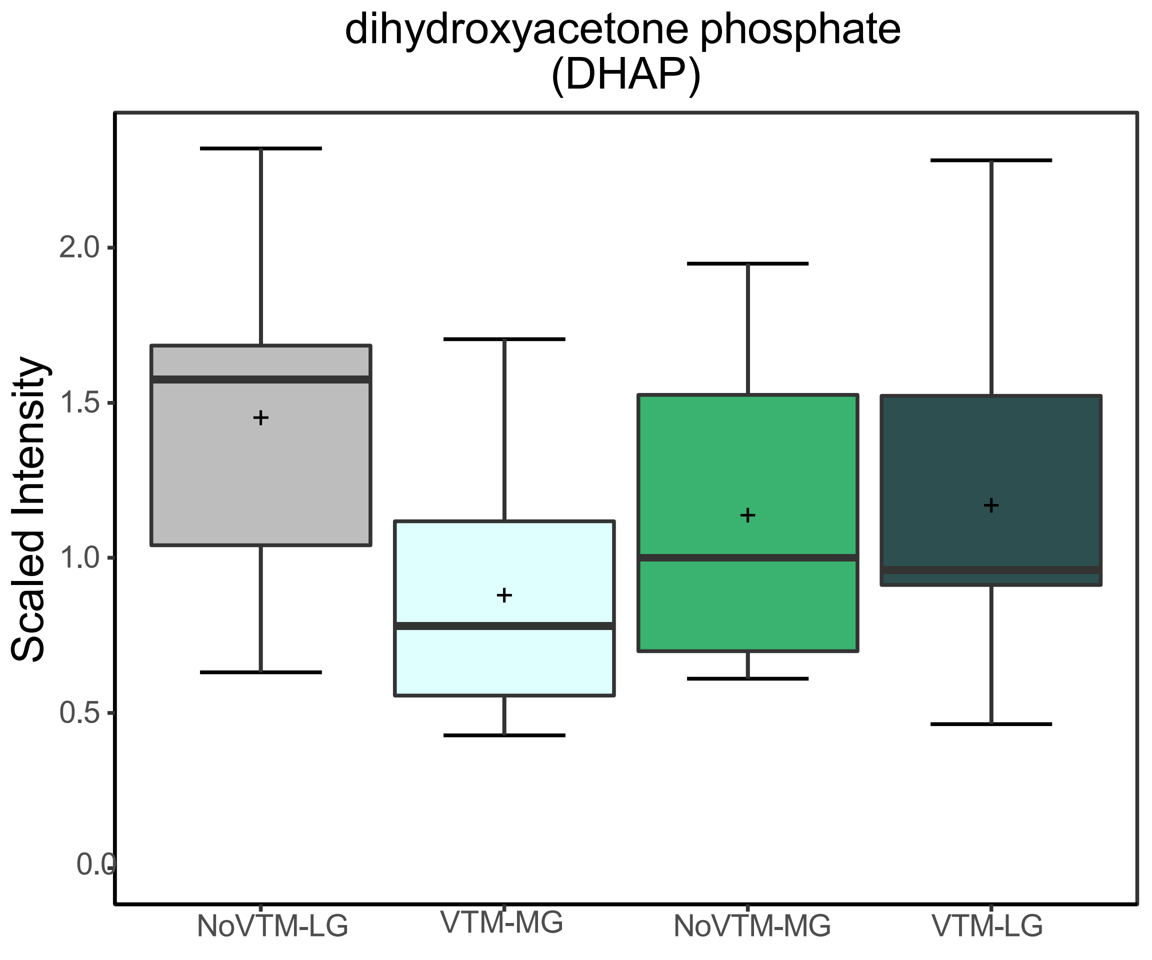

dihydroxyacetone phosphate
(DHAP)
Scaled Intensity
2.0
1.5
1.0
0.5
0.0
NoVTM-LG
VTM-MG
NoVTM-MG
VTM-LG

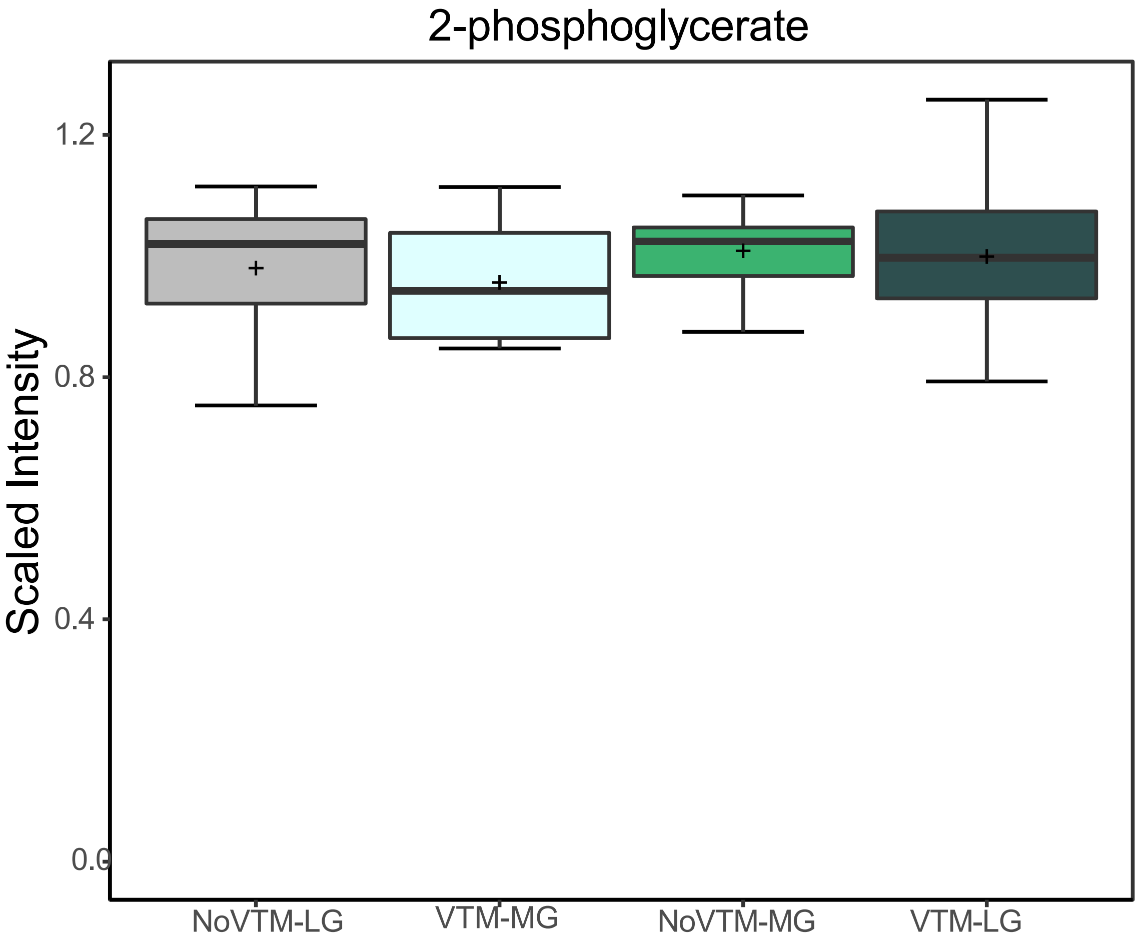
2-phosphoglycerate
Scaled Intensity
1.2
0.8
0.4
0.0
NoVTM-LG
VTM-MG
NoVTM-MG
VTM-LG

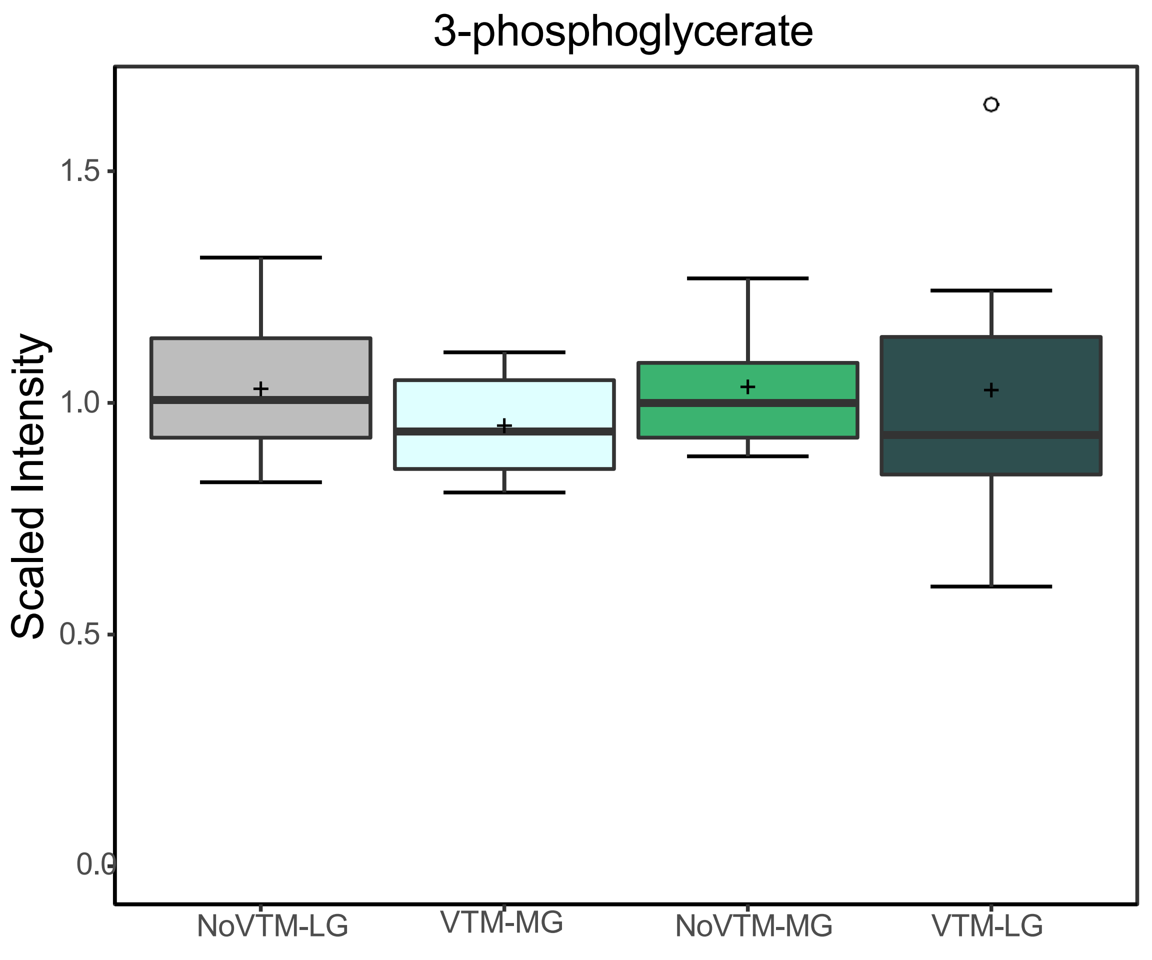
3-phosphoglycerate
Scaled Intensity
1.5
1.0
0.5
0.0
NoVTM-LG
VTM-MG
NoVTM-MG
VTM-LG

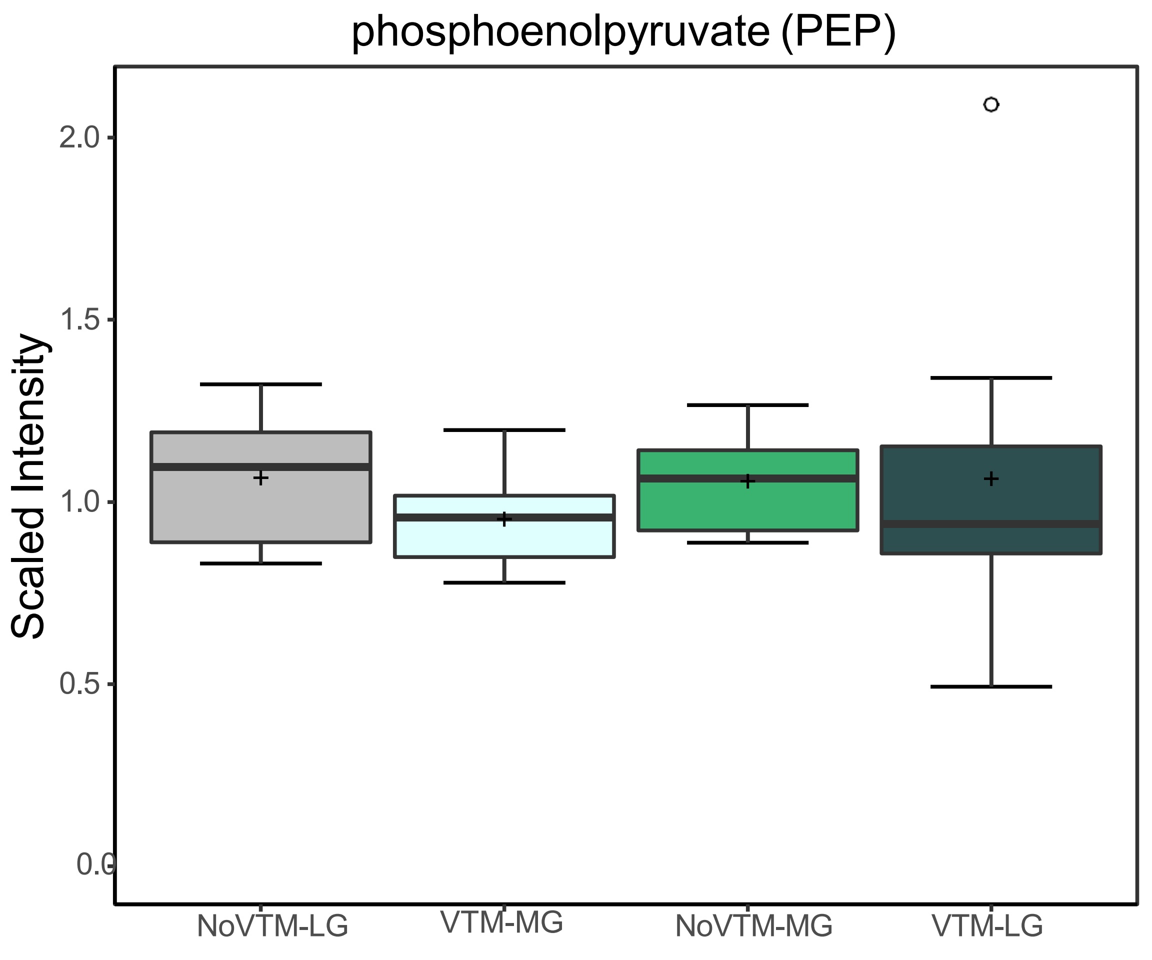

phosphoenolpyruvate (PEP)
Scaled Intensity
2.0
1.5
1.0
0.5
0.0
NoVTM-LG
VTM-MG
NoVTM-MG
VTM-LG

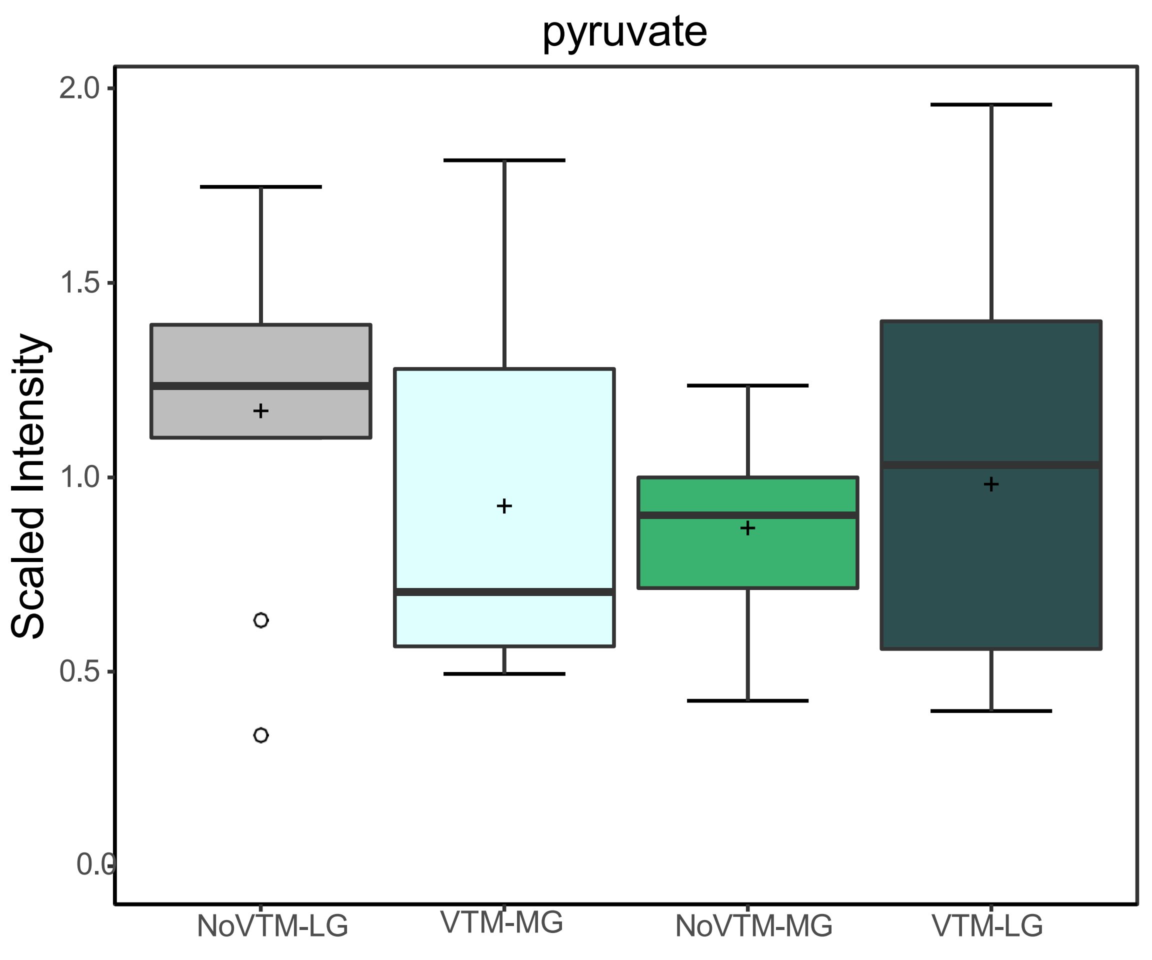
pyruvate
Scaled Intensity
2.0
1.5
1.0
0.5
0.0
NoVTM-LG
VTM-MG
NoVTM-MG
VTM-LG

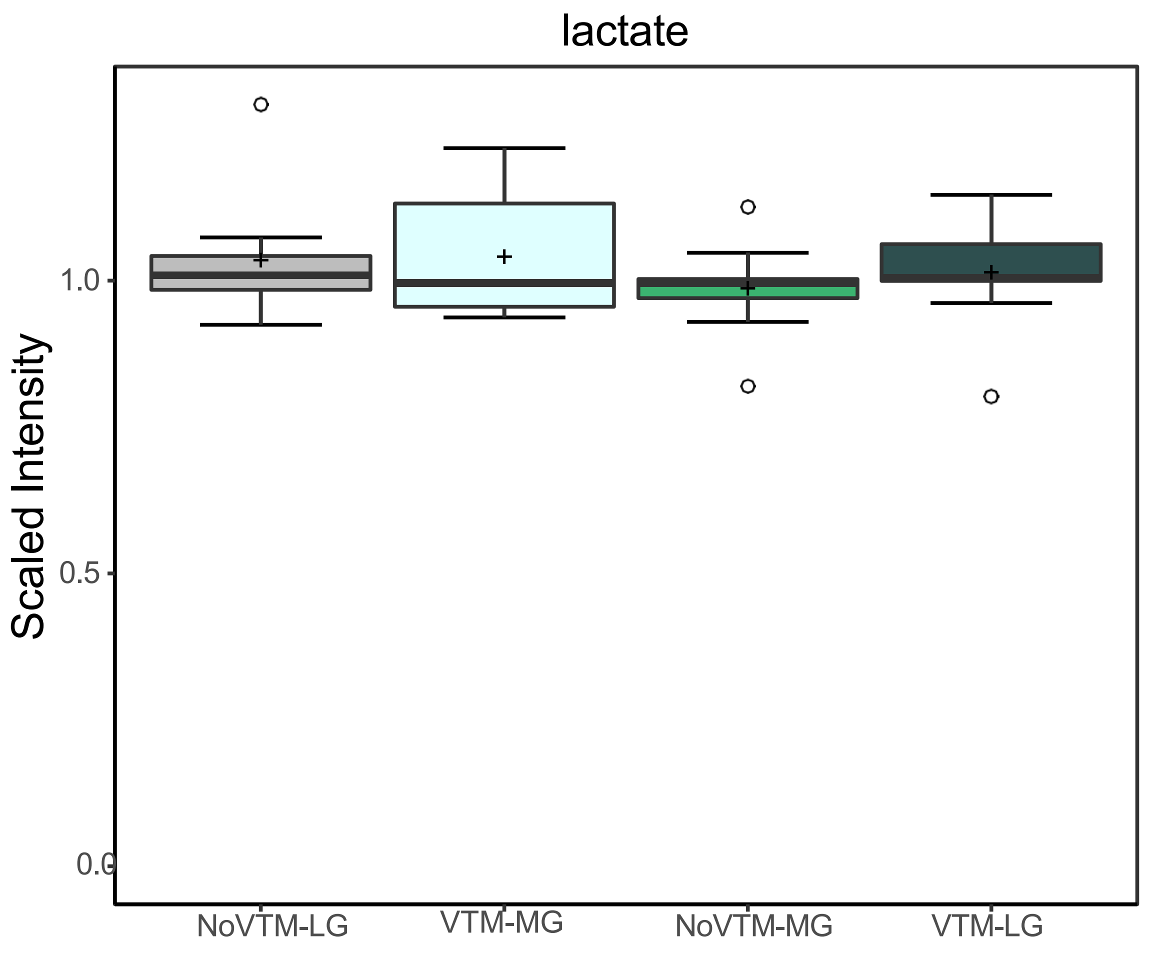
lactate
Scaled Intensity
1.0
0.5
0.0
NoVTM-LG
VTM-MG
NoVTM-MG
VTM-LG

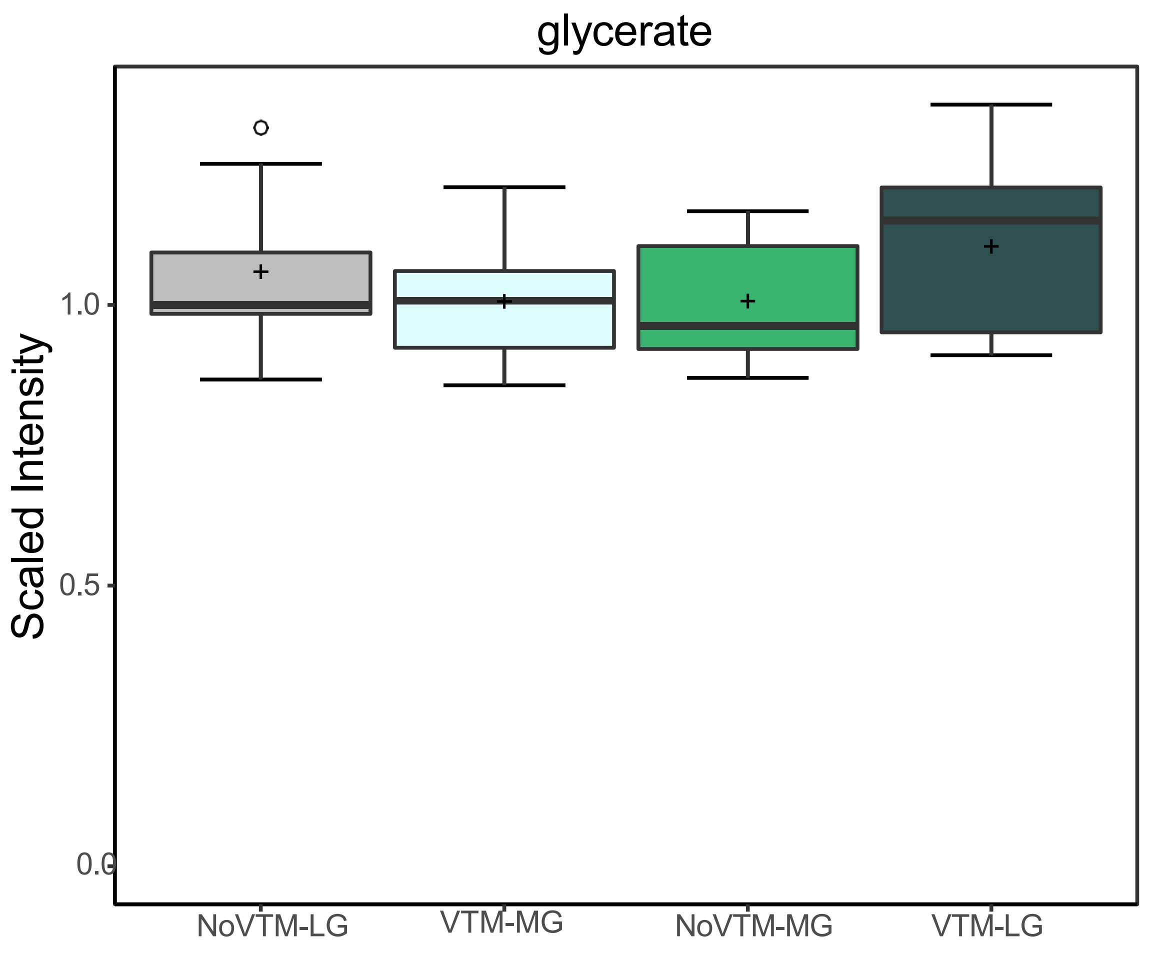
glycerate
Scaled Intensity
1.0
0.5
0.0
NoVTM-LG
VTM-MG
NoVTM-MG
VTM-LG

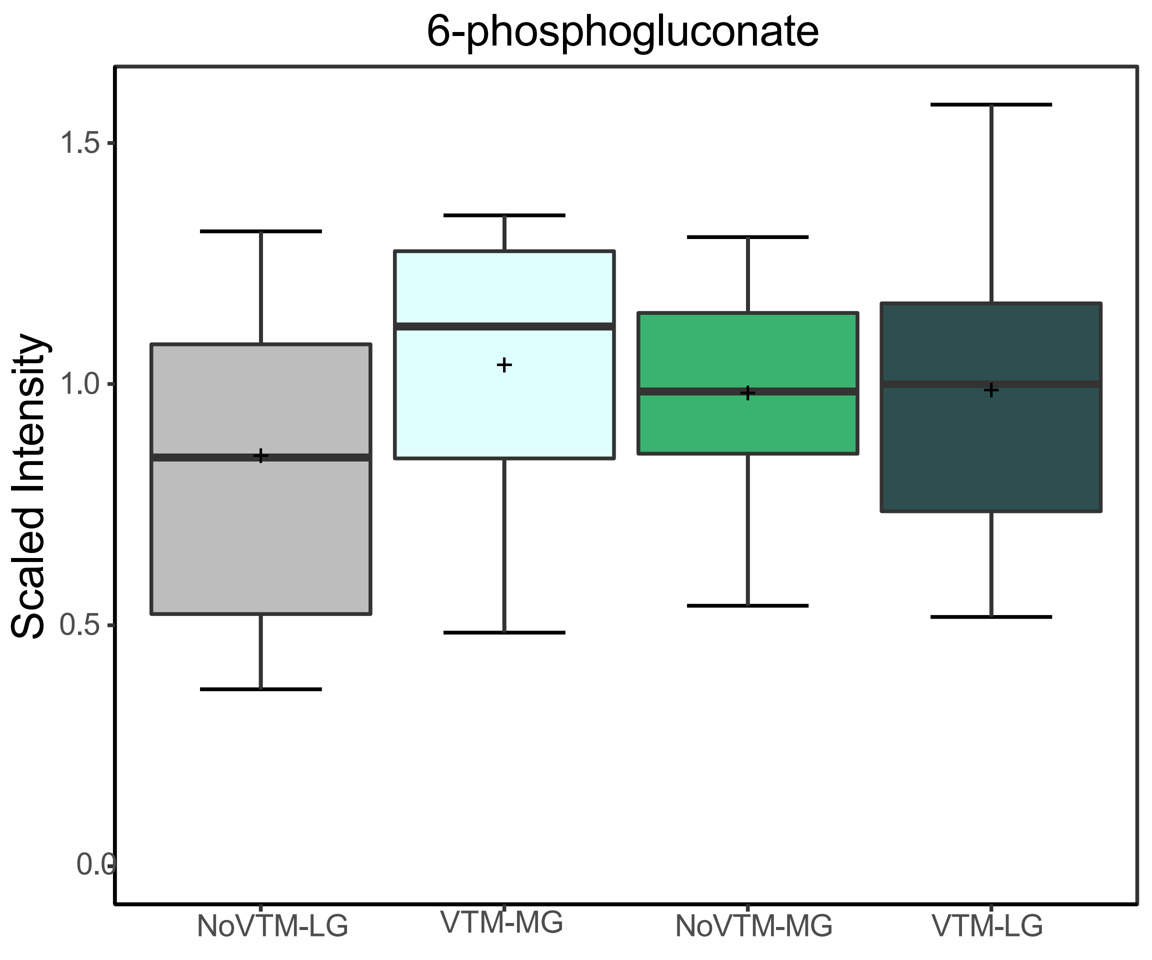

6-phosphogluconate
Scaled Intensity
1.5
1.0
0.5
0.0
NoVTM-LG
VTM-MG
NoVTM-MG
VTM-LG

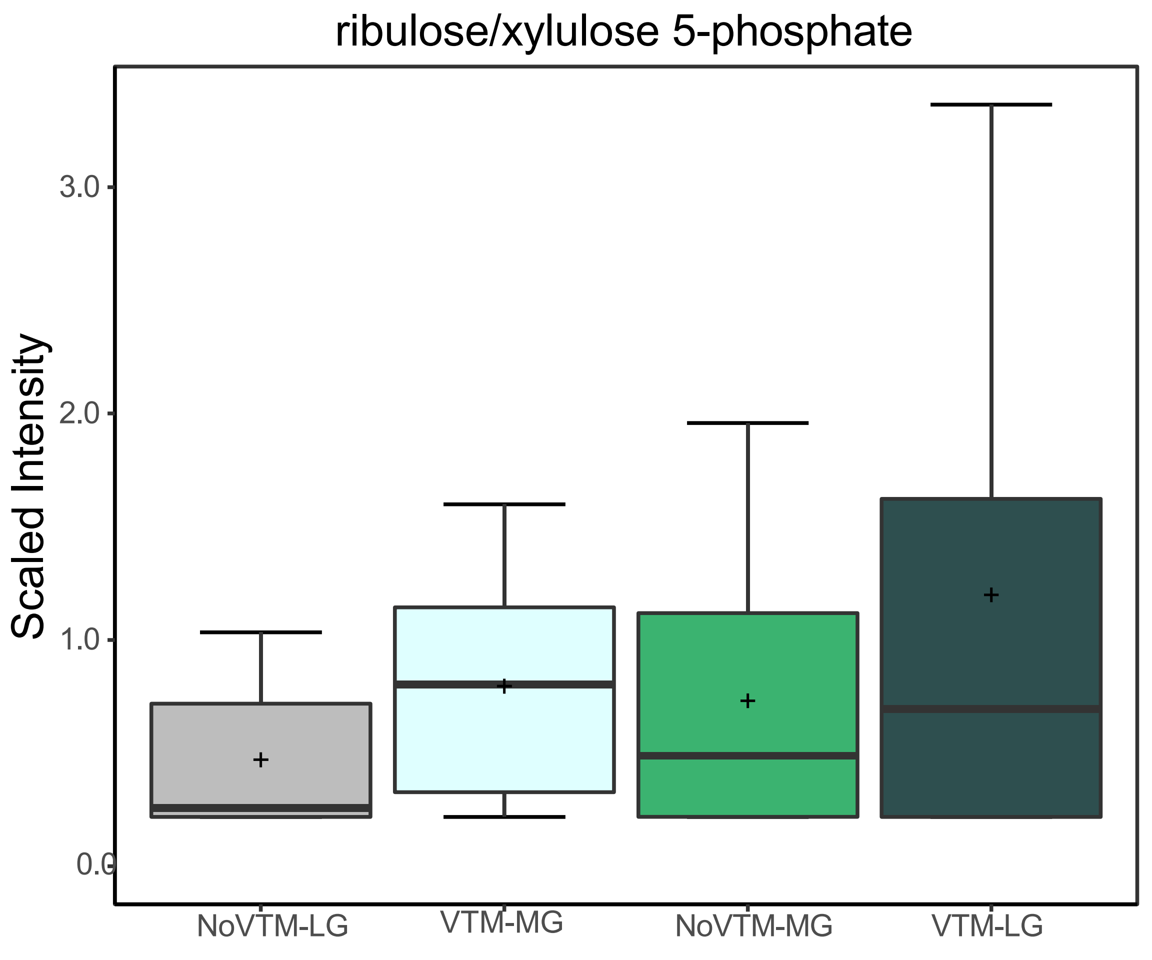

ribulose/xylulose 5-phosphate
Scaled Intensity
3.0
2.0
1.0
0.0
NoVTM-LG
VTM-MG
NoVTM-MG
VTM-LG

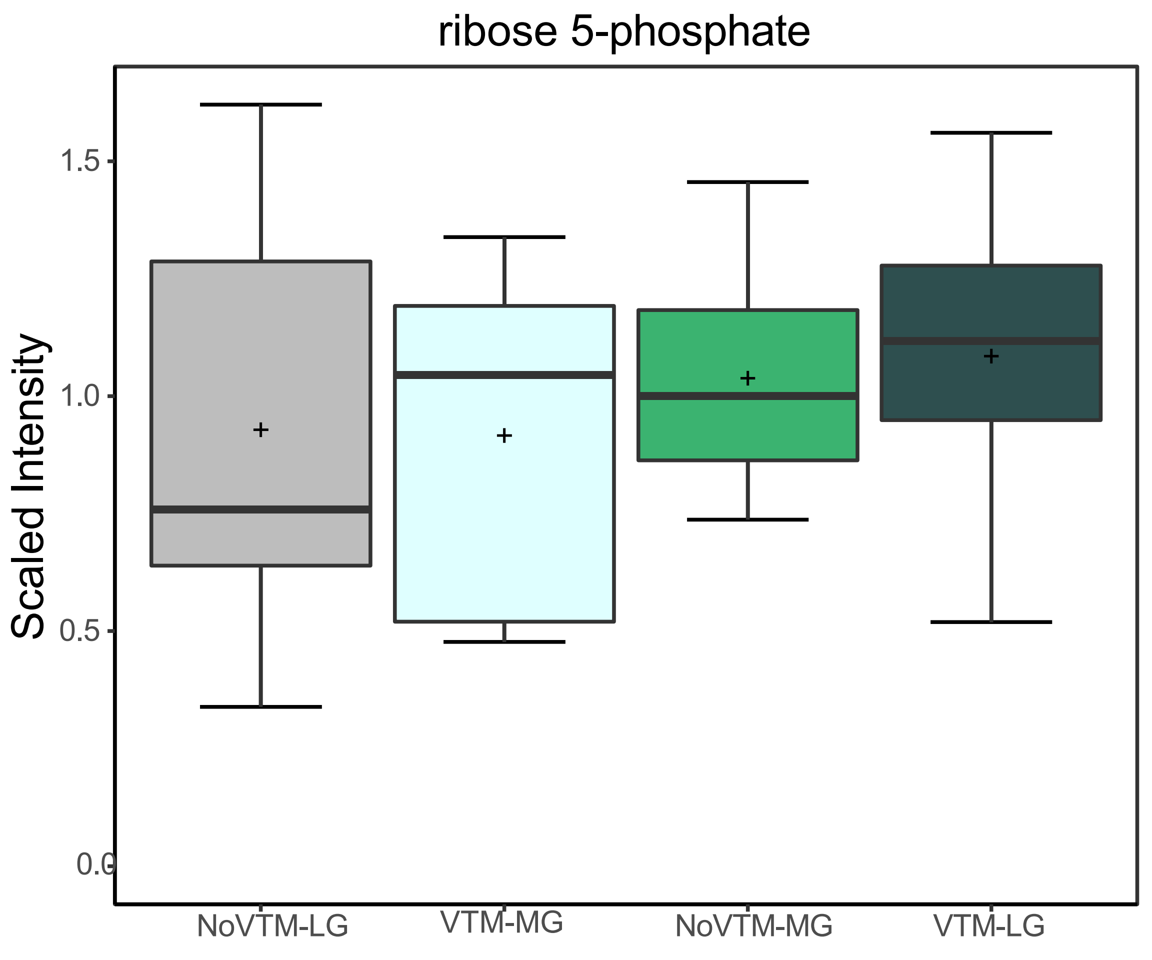

ribose 5-phosphate
Scaled Intensity
1.5
1.0
0.5
0.0
NoVTM-LG
VTM-MG
NoVTM-MG
VTM-LG

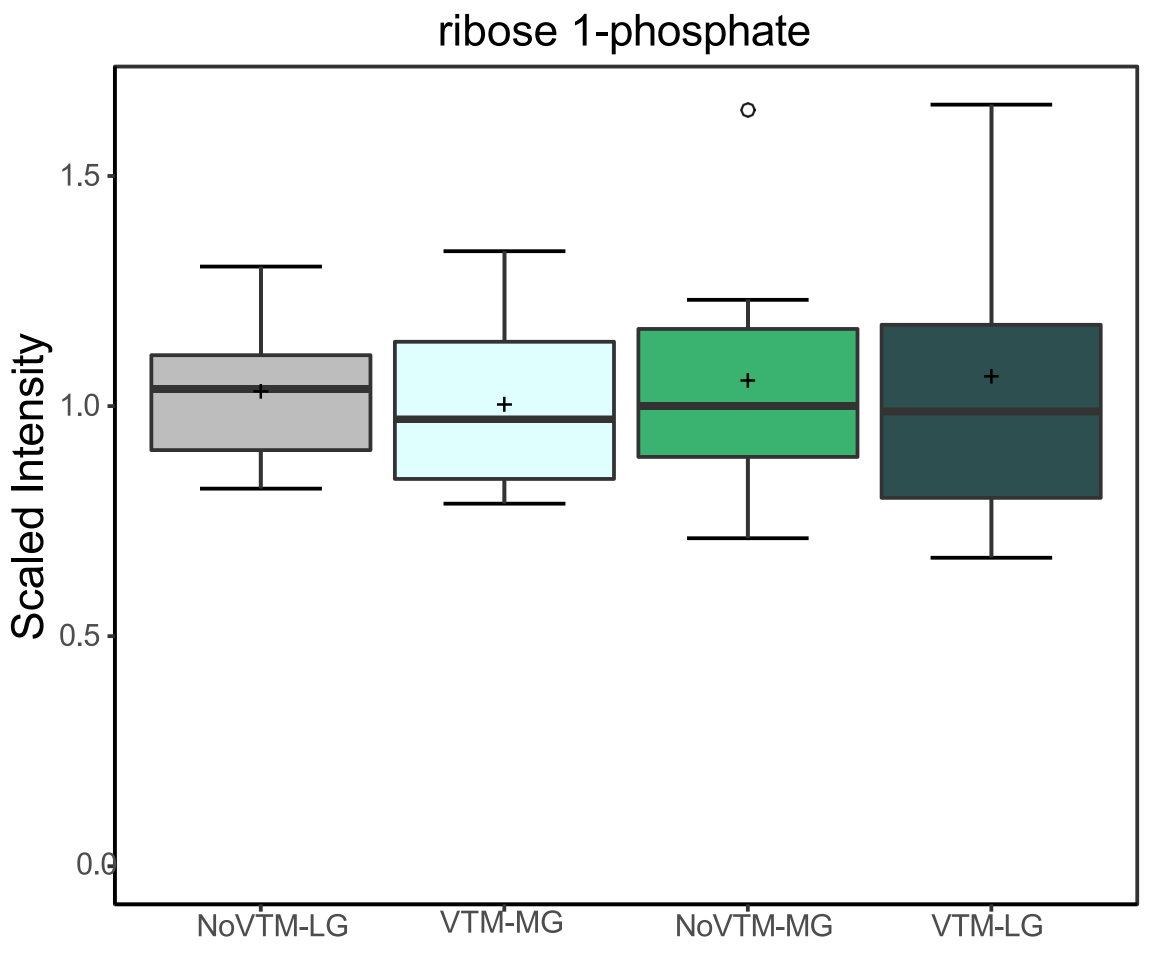

ribose 1-phosphate
Scaled Intensity
1.5
1.0
0.5
0.0
NoVTM-LG
VTM-MG
NoVTM-MG
VTM-LG

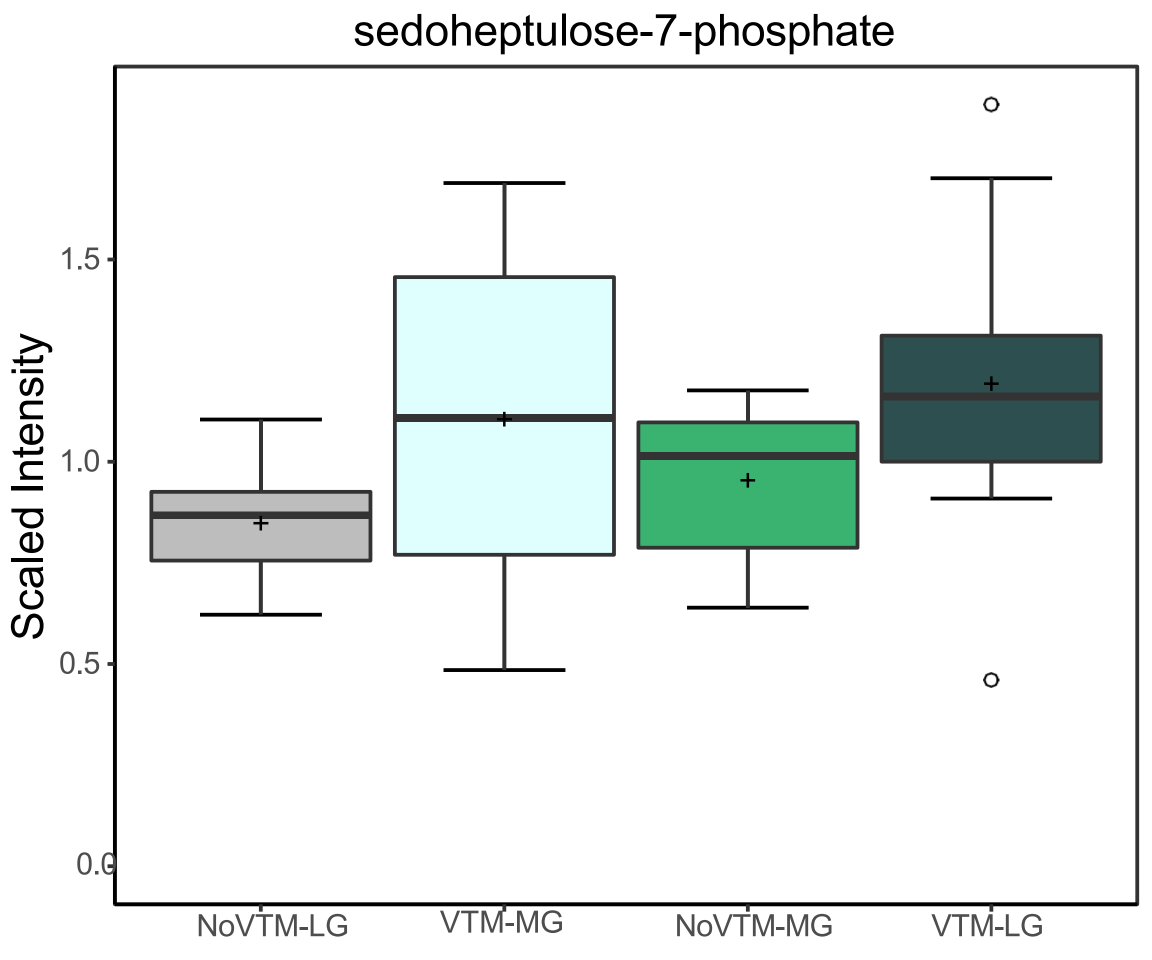

sedoheptulose-7-phosphate
Scaled Intensity
1.5
1.0
0.5
0.0
NoVTM-LG
VTM-MG
NoVTM-MG
VTM-LG

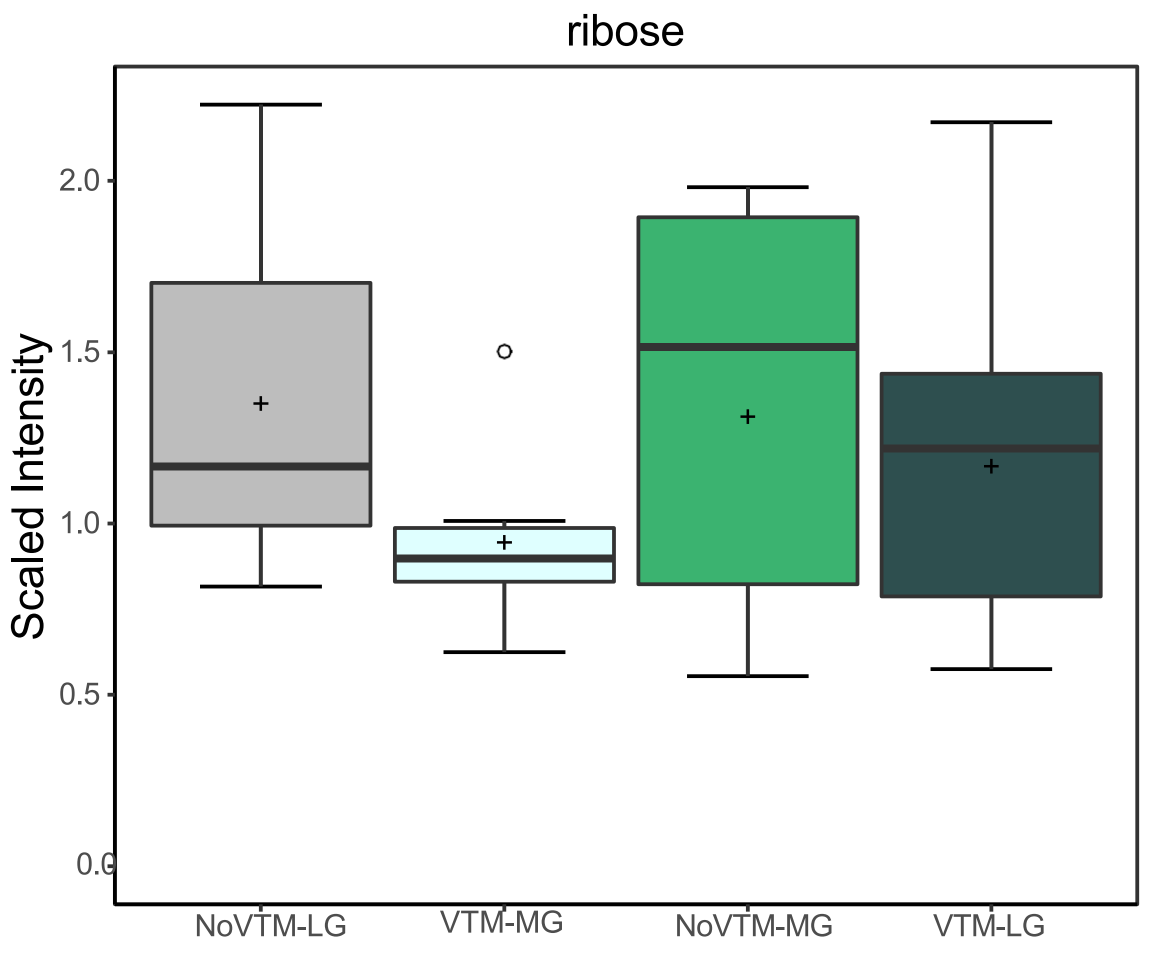
ribose
Scaled Intensity
2.0
1.5
1.0
0.5
0.0
NoVTM-LG
VTM-MG
NoVTM-MG
VTM-LG

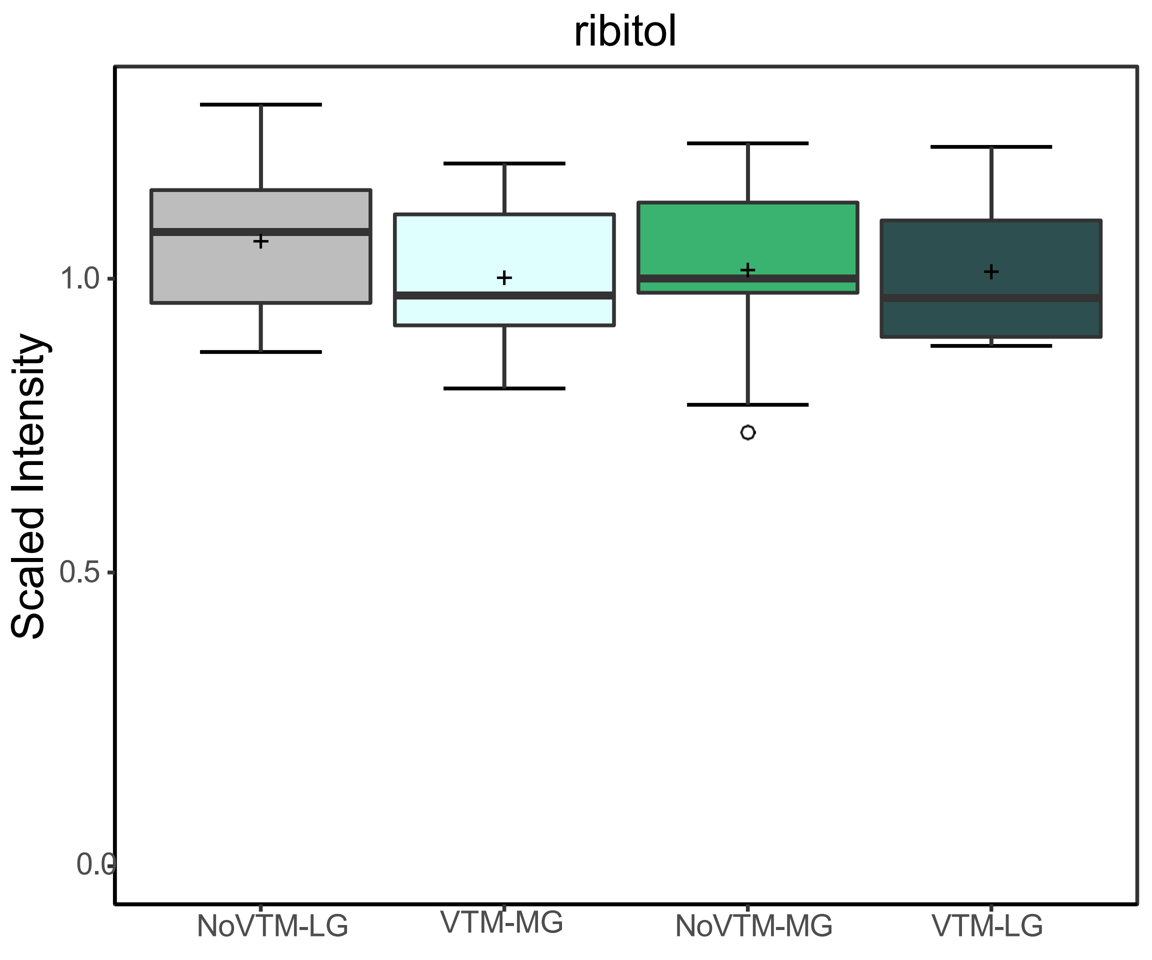
ribitol
Scaled Intensity
1.0
0.5
0.0
NoVTM-LG
VTM-MG
NoVTM-MG
VTM-LG

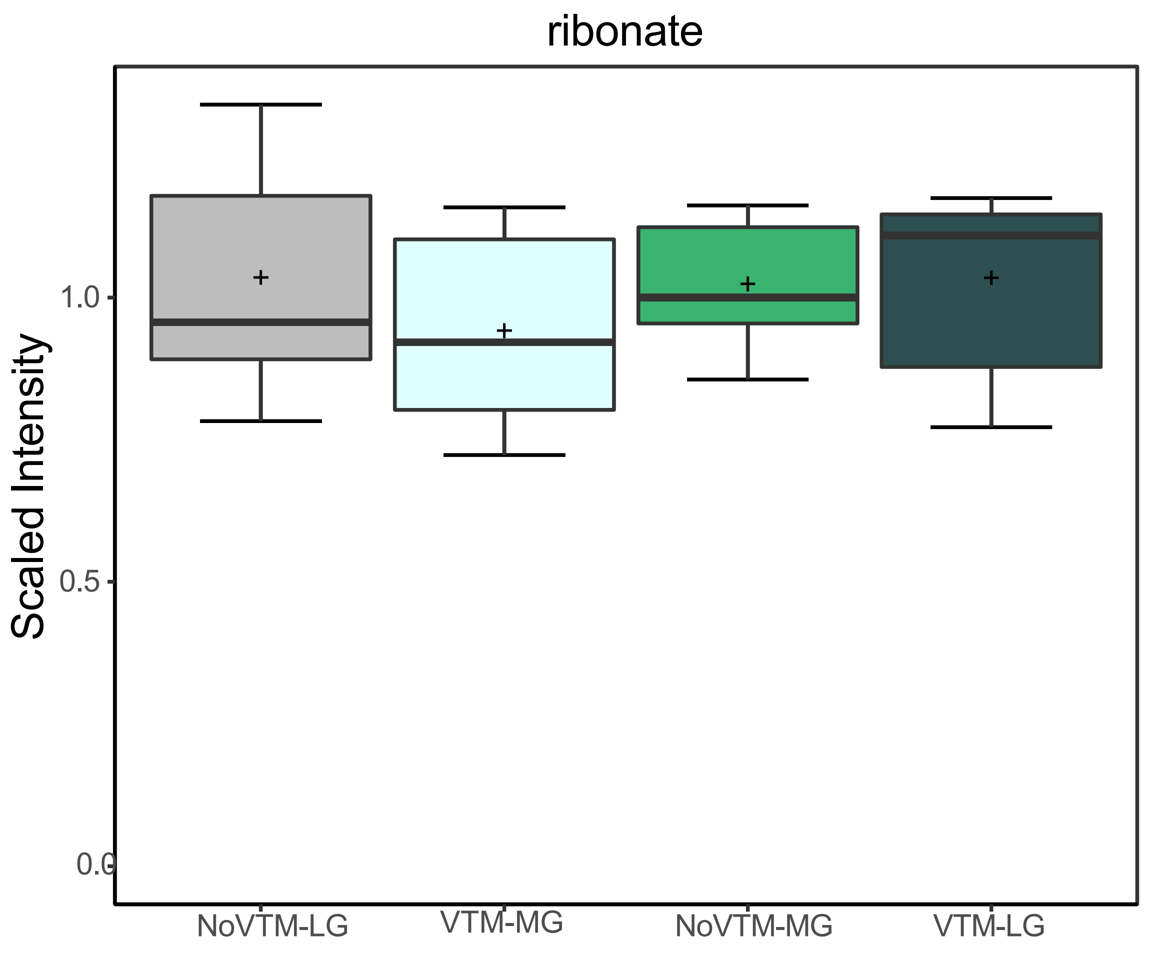
ribonate
Scaled Intensity
1.0
0.5
0.0
NoVTM-LG
VTM-MG
NoVTM-MG
VTM-LG

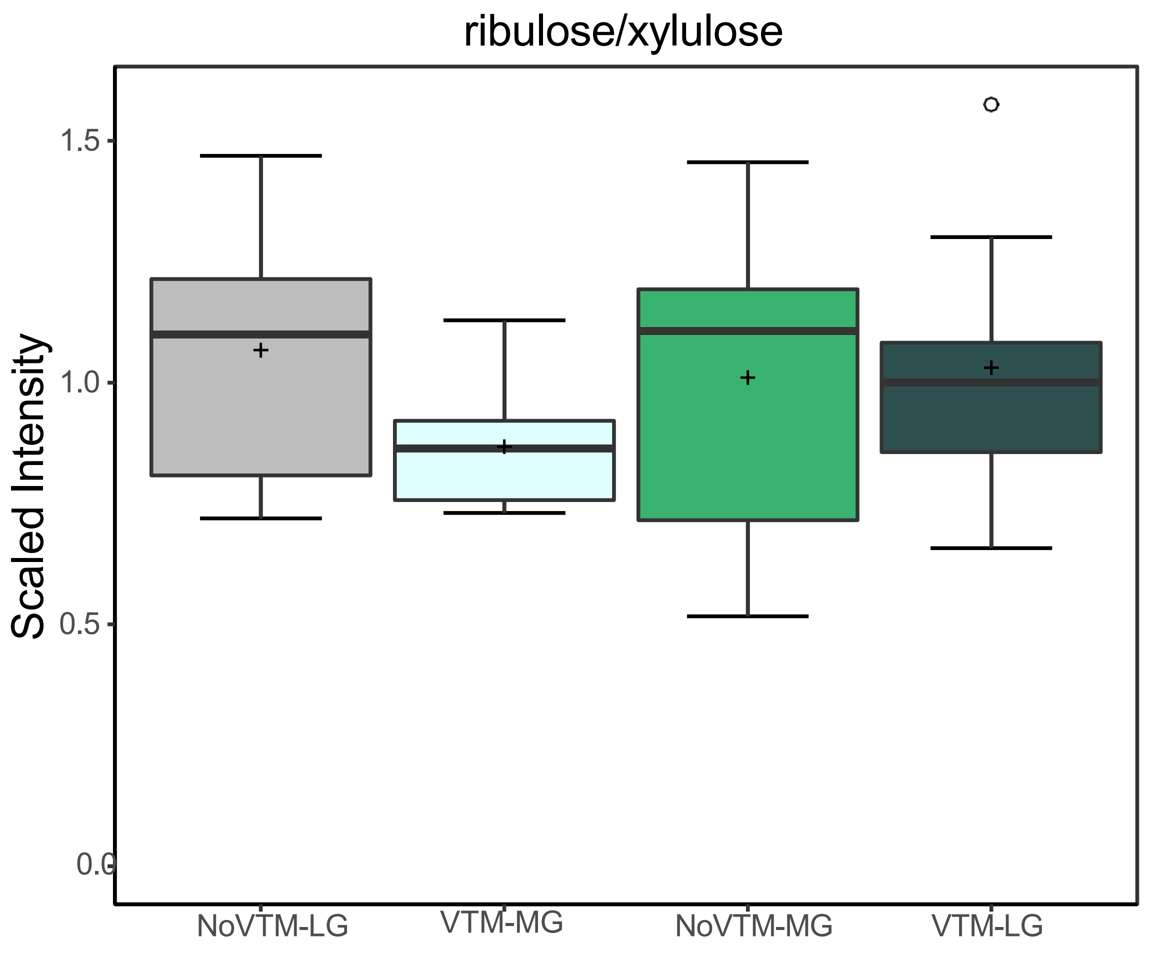
ribulose/xylulose
Scaled Intensity
1.5
1.0
0.5
0.0
NoVTM-LG
VTM-MG
NoVTM-MG
VTM-LG

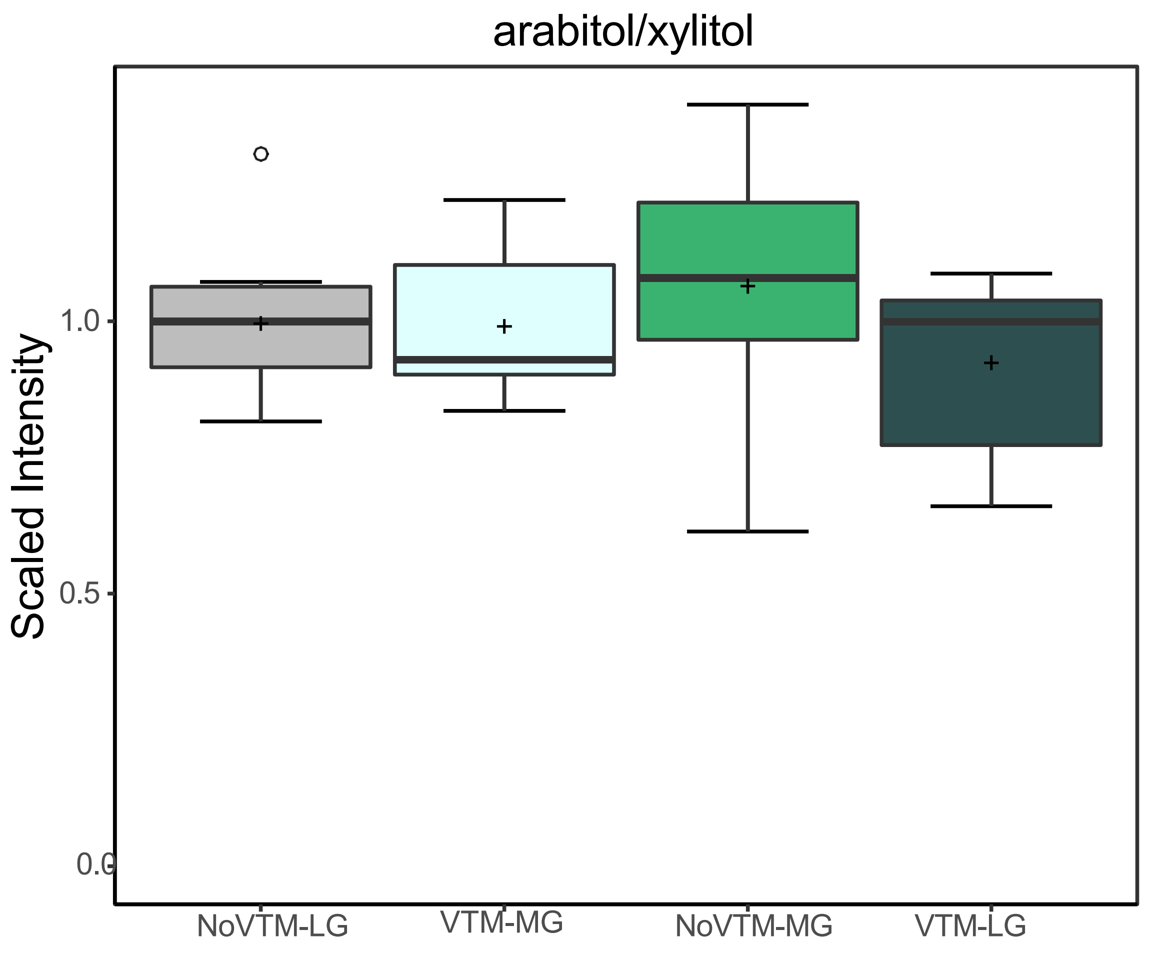

arabitol/xylitol
Scaled Intensity
1.0
0.5
0.0
NoVTM-LG
VTM-MG
NoVTM-MG
VTM-LG

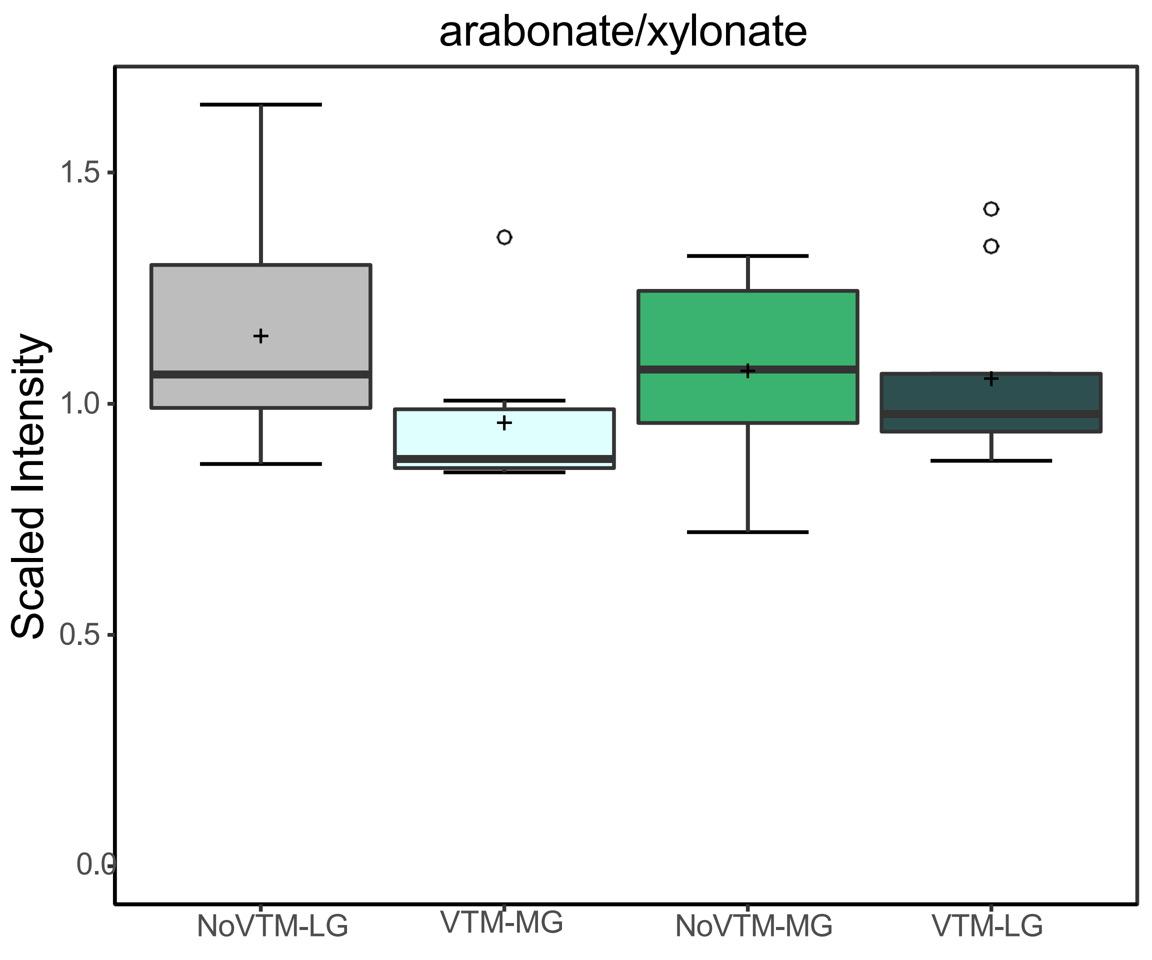

arabonate/xylonate
Scaled Intensity
1.5
1.0
0.5
0.0
NoVTM-LG
VTM-MG
NoVTM-MG
VTM-LG

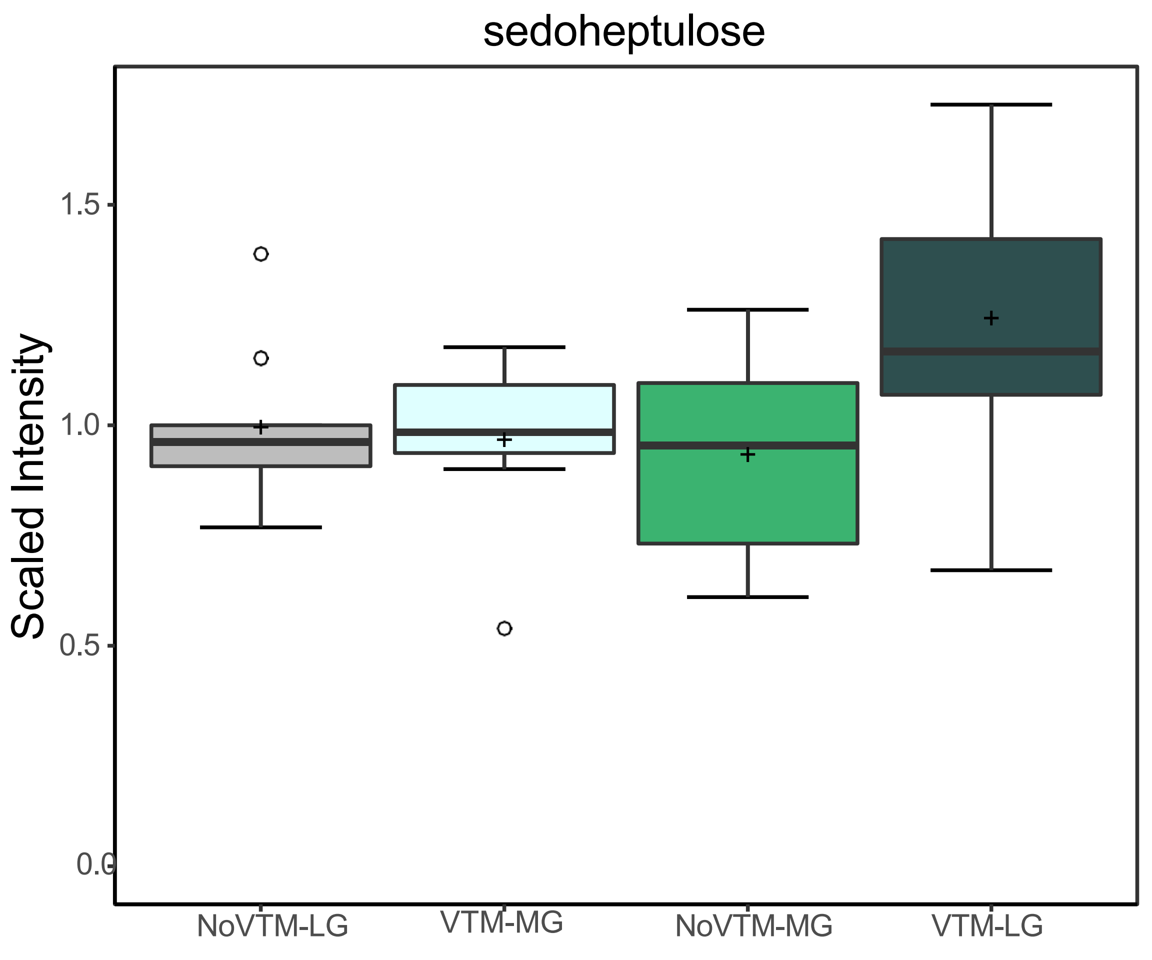

sedoheptulose
Scaled Intensity
1.5
1.0
0.5
0.0
NoVTM-LG
VTM-MG
NoVTM-MG
VTM-LG

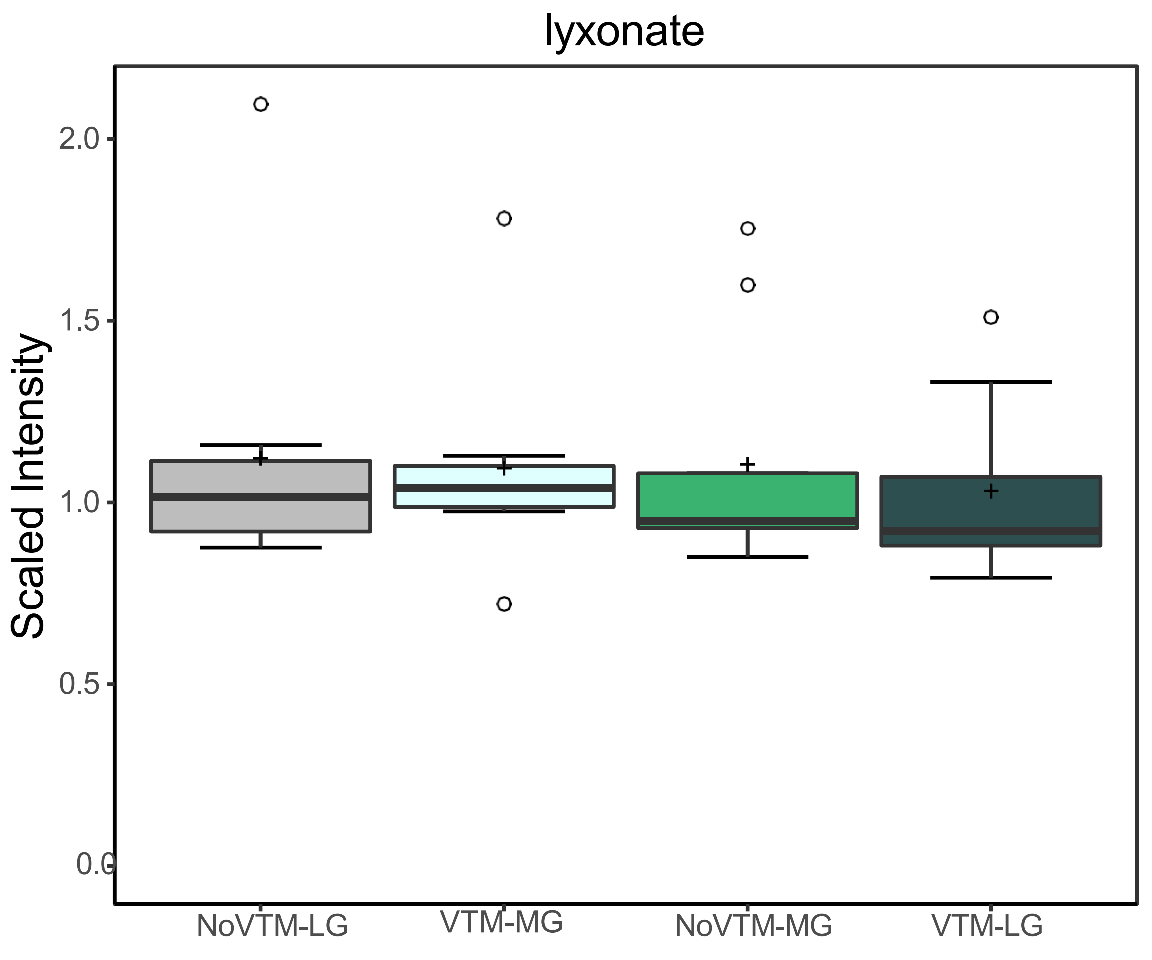
lyxonate
Scaled Intensity
2.0
1.5
1.0
0.5
0.0
NoVTM-LG
VTM-MG
NoVTM-MG
VTM-LG

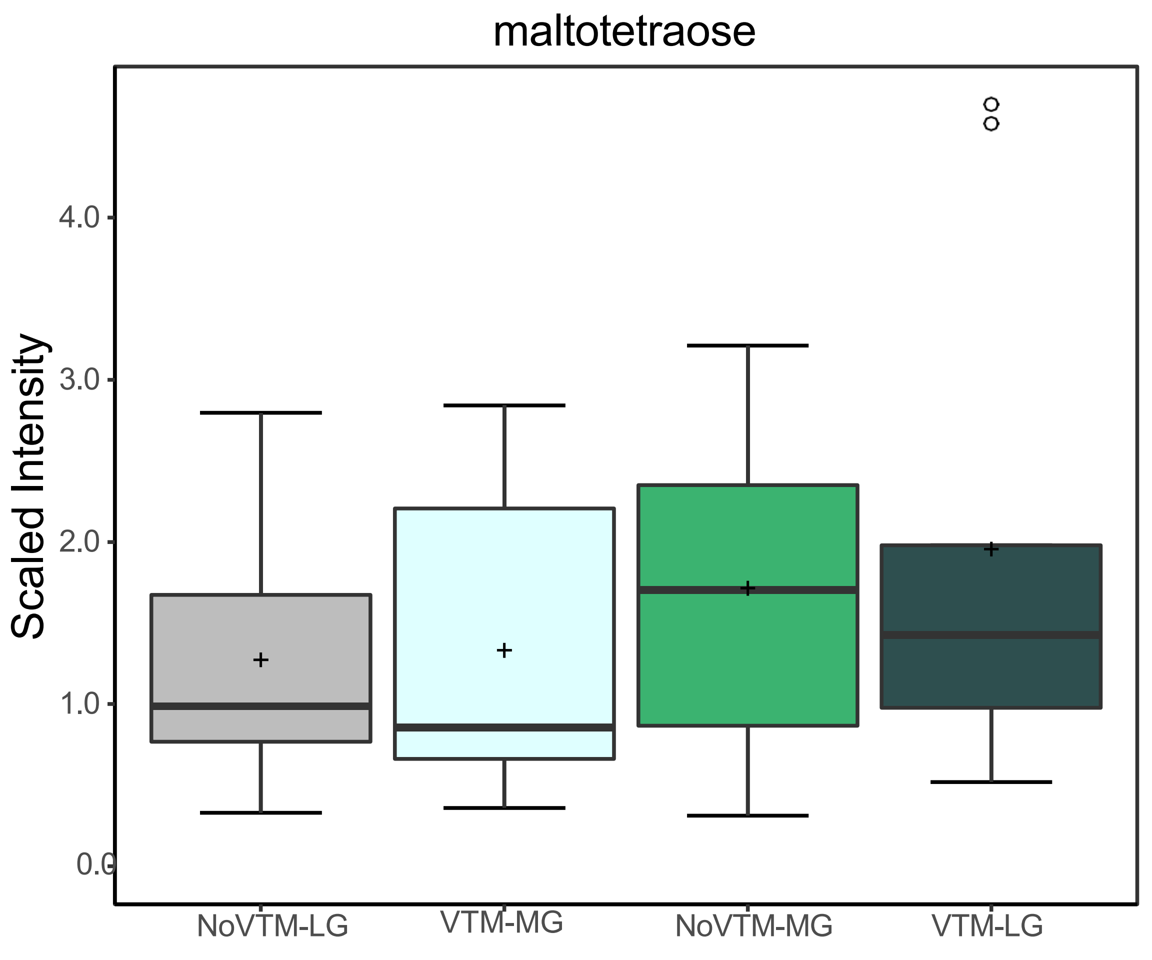

maltotetraose
Scaled Intensity
4.0
3.0
2.0
1.0
0.0
NoVTM-LG
VTM-MG
NoVTM-MG
VTM-LG

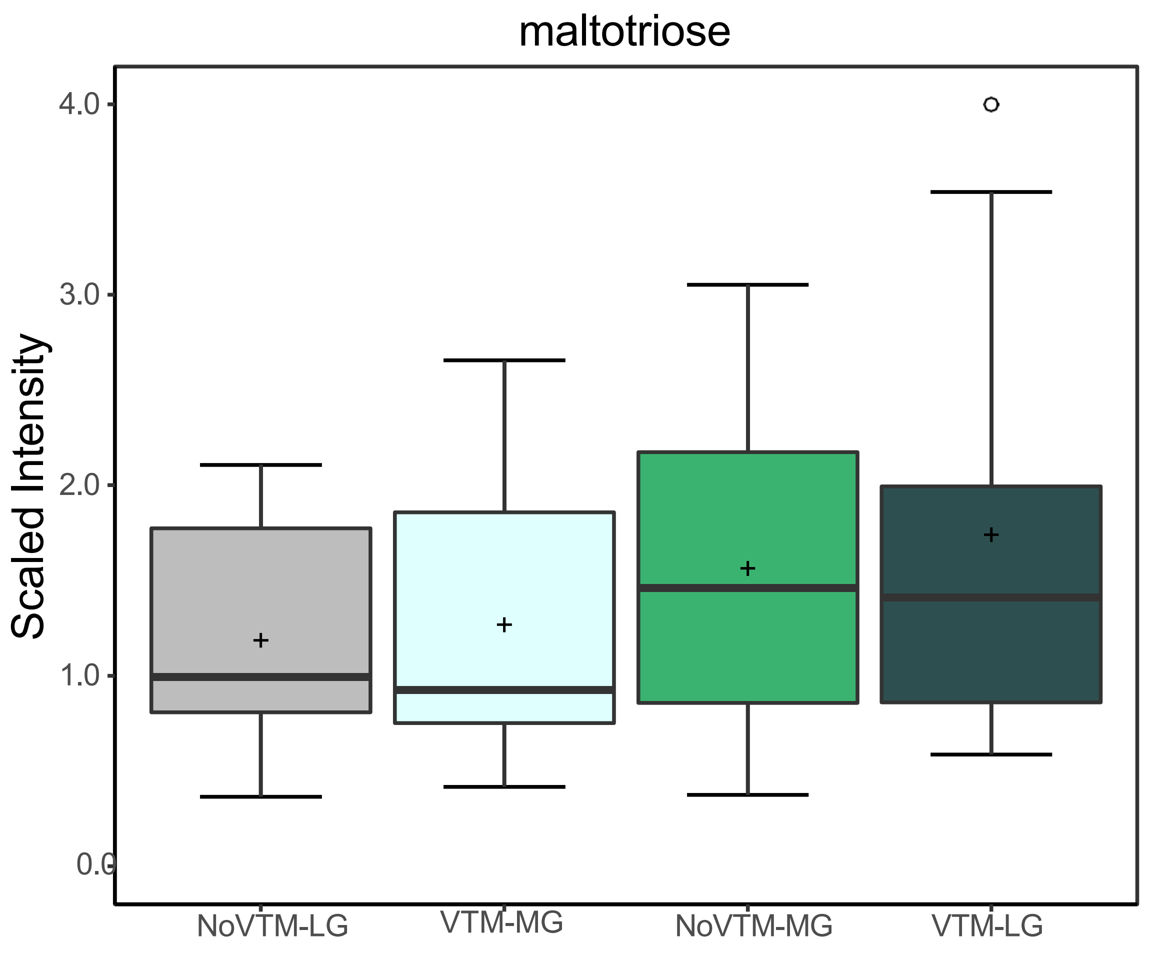

maltotriose
Scaled Intensity
4.0
3.0
2.0
1.0
0.0
NoVTM-LG
VTM-MG
NoVTM-MG
VTM-LG

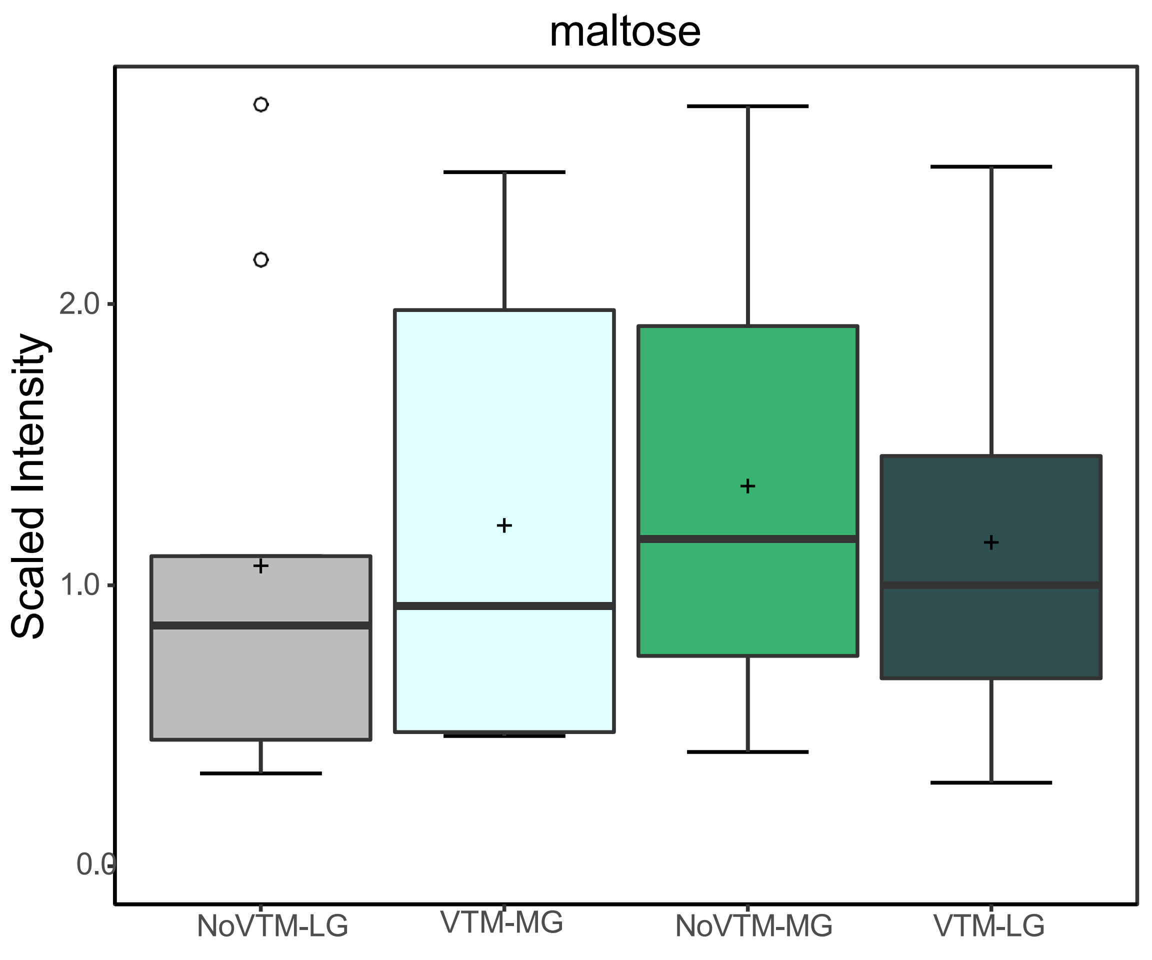

maltose
Scaled Intensity
2.0
1.0
0.0
NoVTM-LG
VTM-MG
NoVTM-MG
VTM-LG

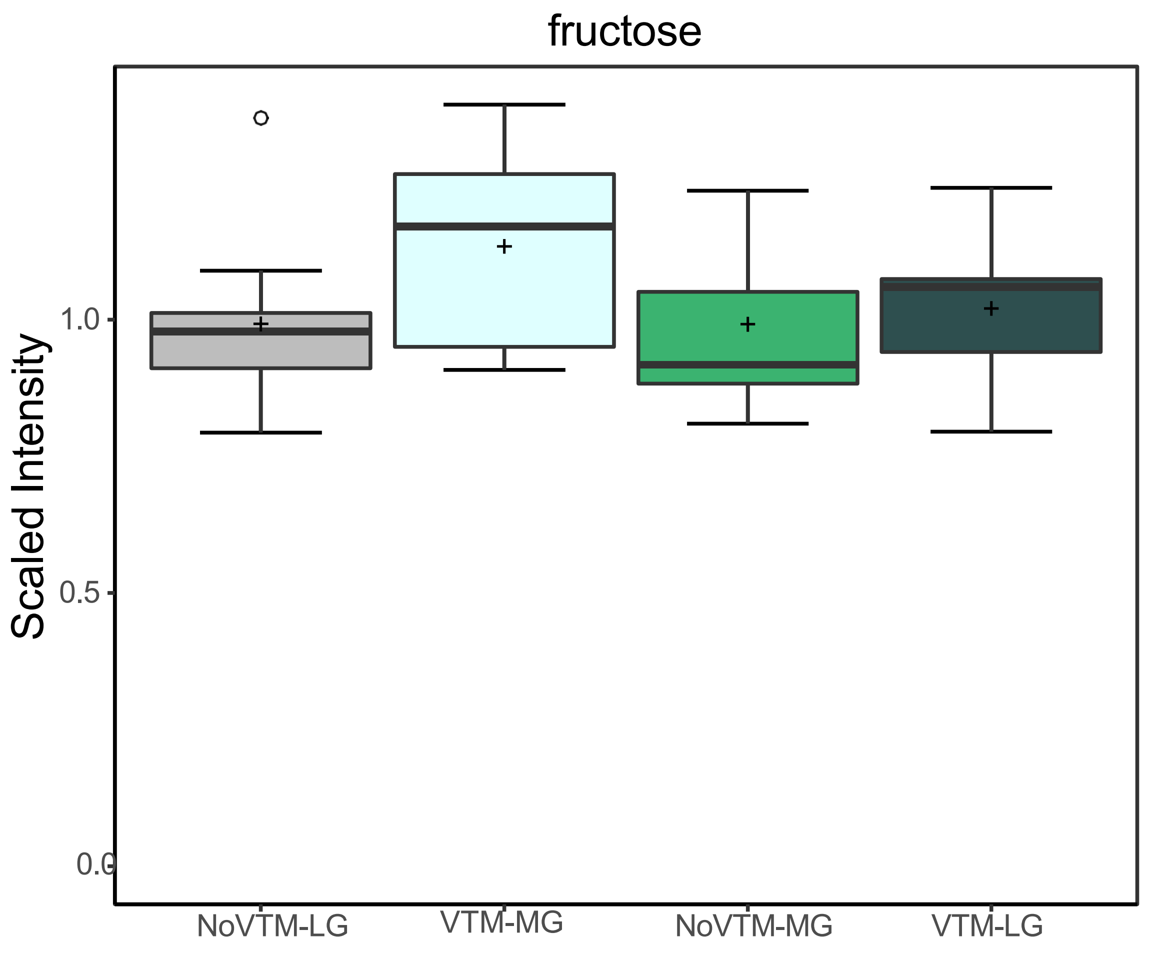
fructose
Scaled Intensity
1.0
0.5
0.0
NoVTM-LG
VTM-MG
NoVTM-MG
VTM-LG

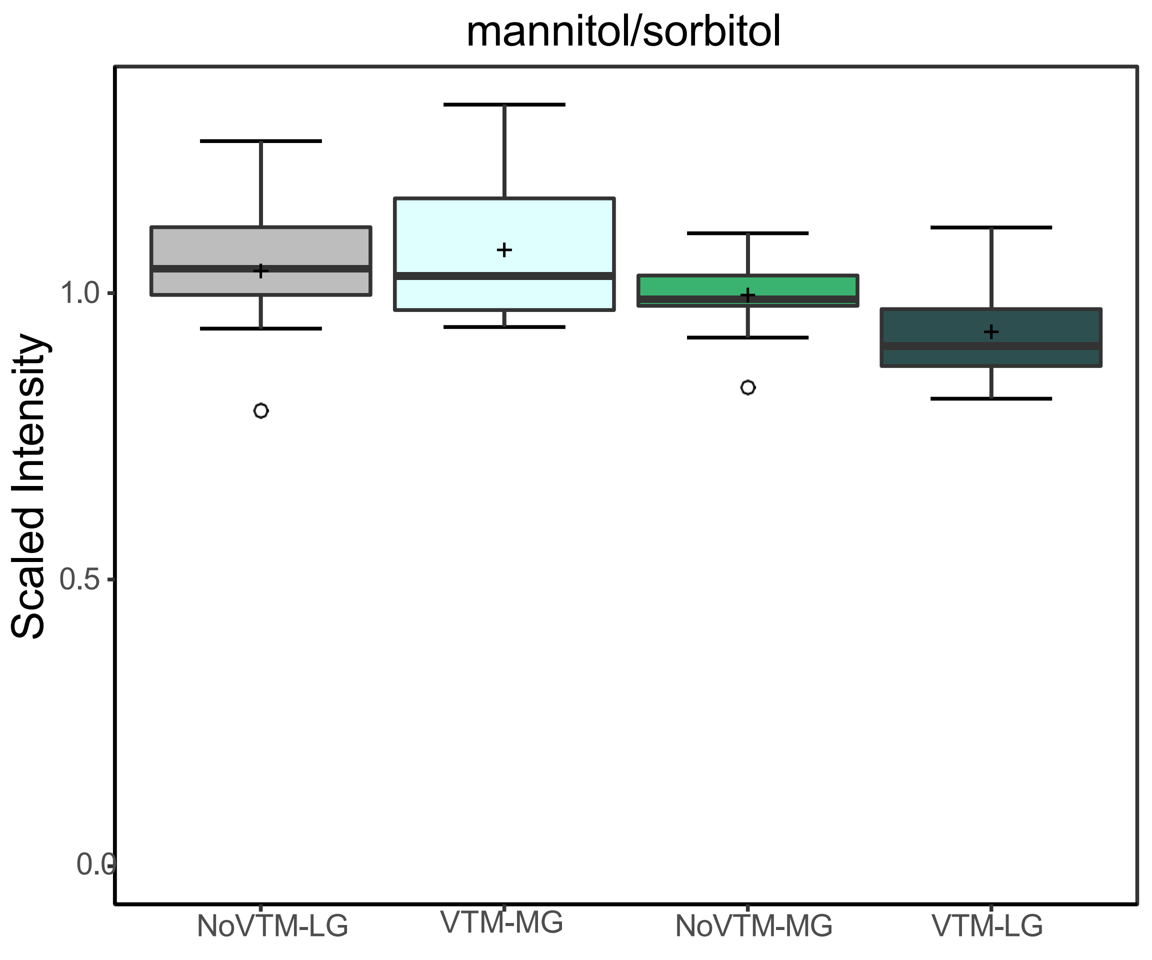

mannitol/sorbitol
Scaled Intensity
1.0
0.5
0.0
NoVTM-LG
VTM-MG
NoVTM-MG
VTM-LG

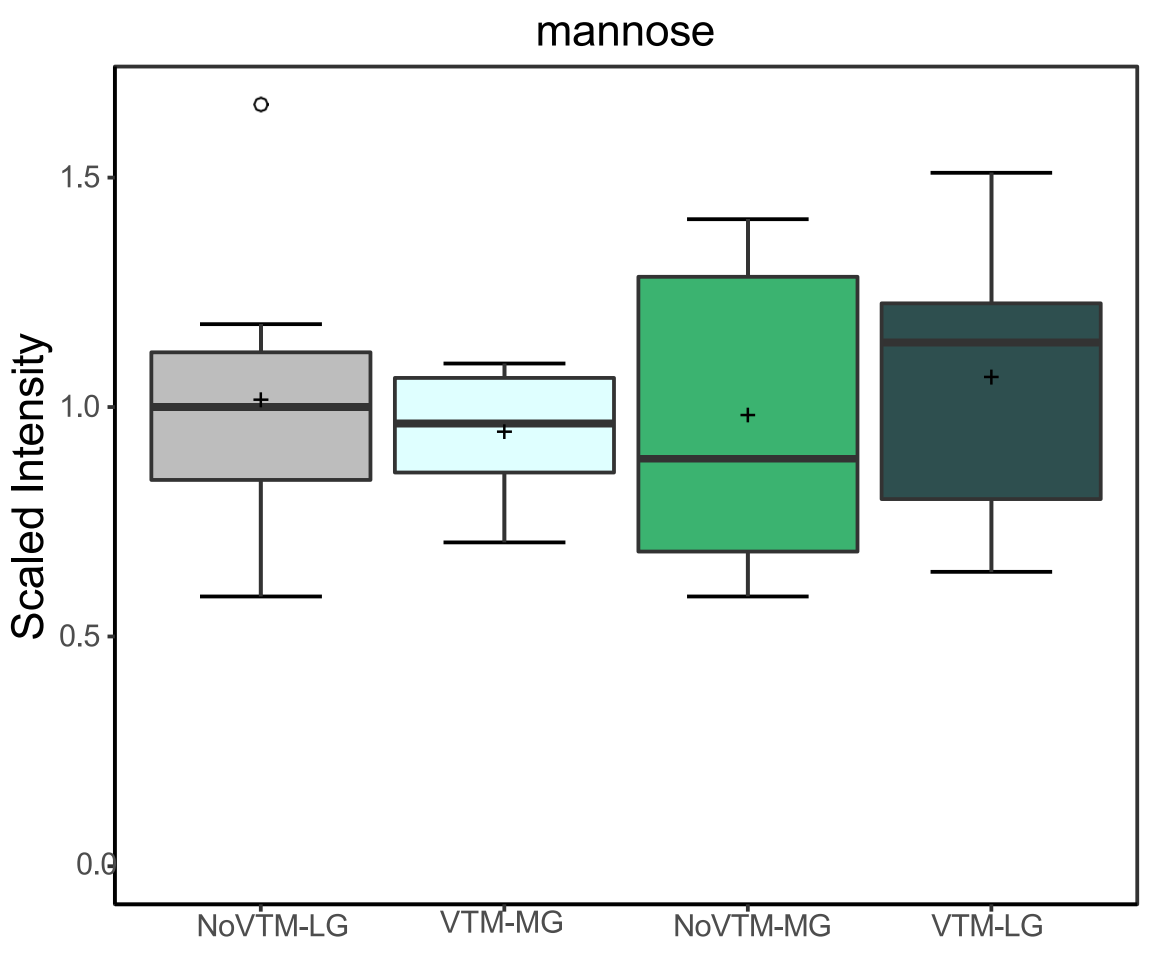
mannose
Scaled Intensity
1.5
1.0
0.5
0.0
NoVTM-LG
VTM-MG
NoVTM-MG
VTM-LG

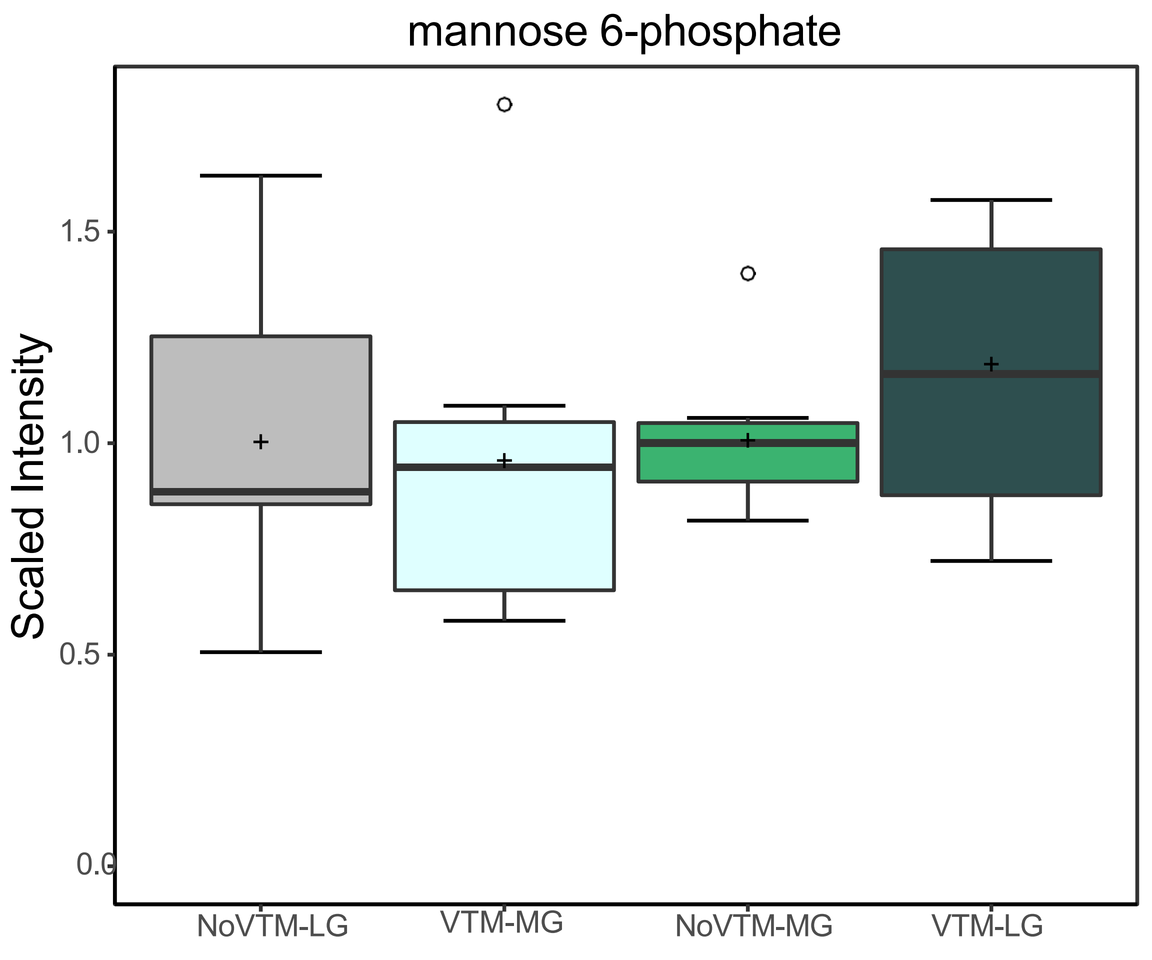

mannose 6-phosphate
Scaled Intensity
1.5
1.0
0.5
0.0
NoVTM-LG
VTM-MG
NoVTM-MG
VTM-LG

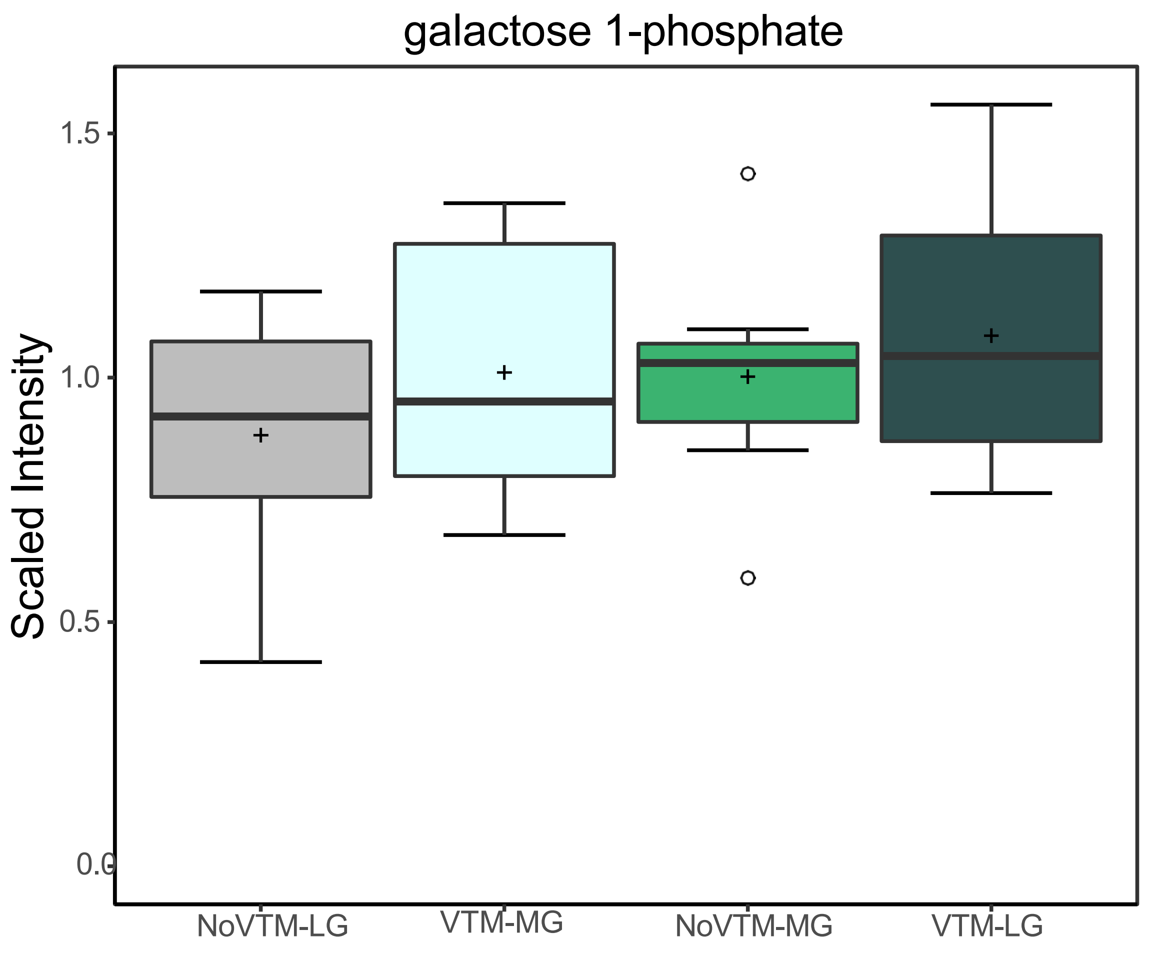

galactose 1-phosphate
Scaled Intensity
1.5
1.0
0.5
0.0
NoVTM-LG
VTM-MG
NoVTM-MG
VTM-LG

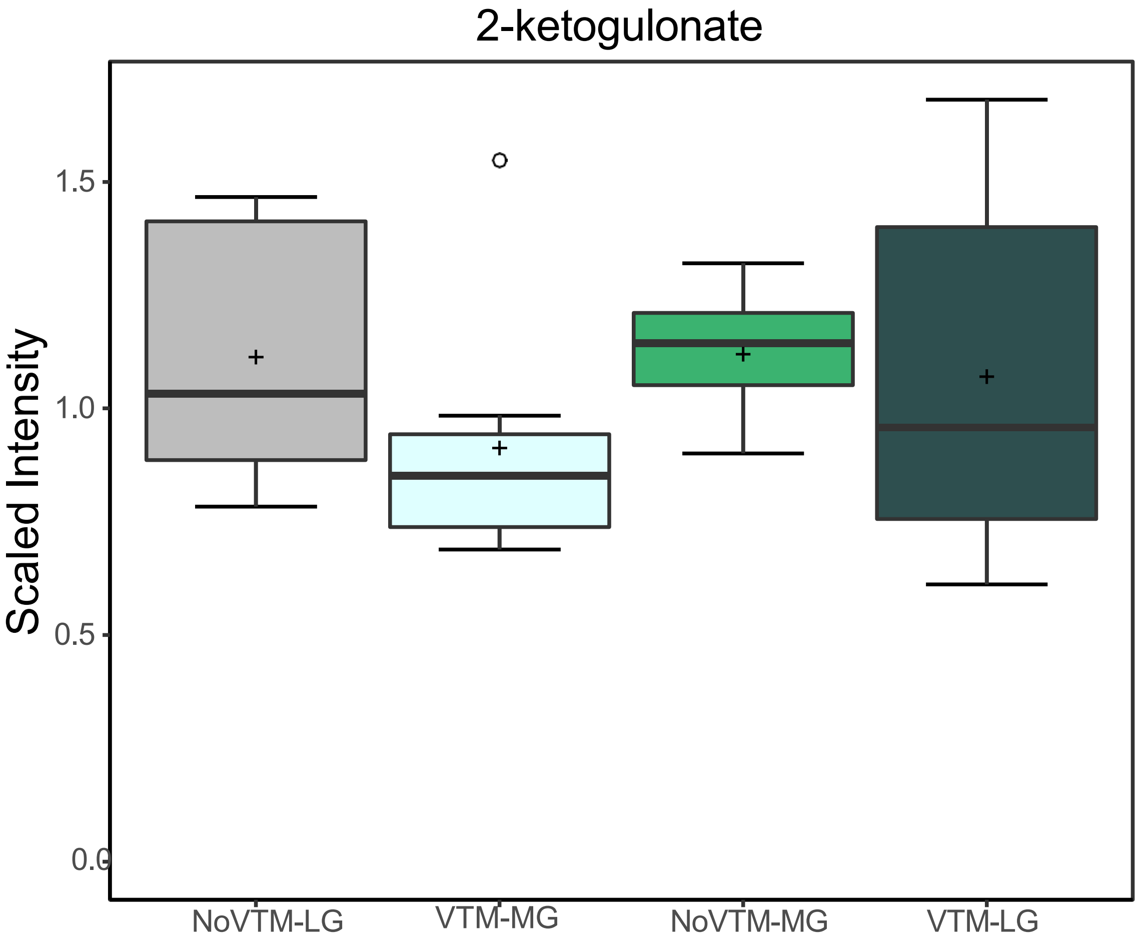
2-ketogulonate
Scaled Intensity
1.5
1.0
0.5
0.0
NoVTM-LG
VTM-MG
NoVTM-MG
VTM-LG

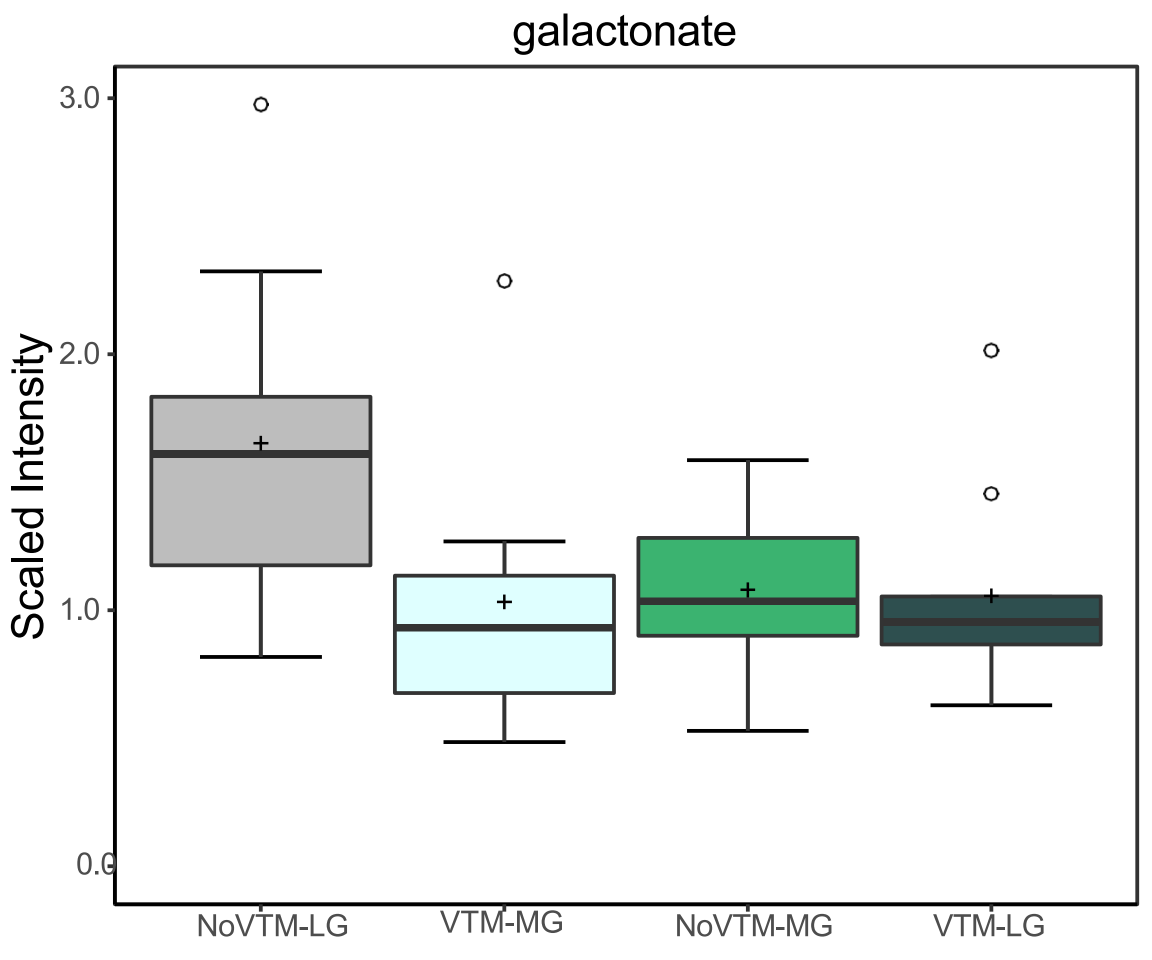
galactonate
Scaled Intensity
3.0
2.0
1.0
0.0
NoVTM-LG
VTM-MG
NoVTM-MG
VTM-LG

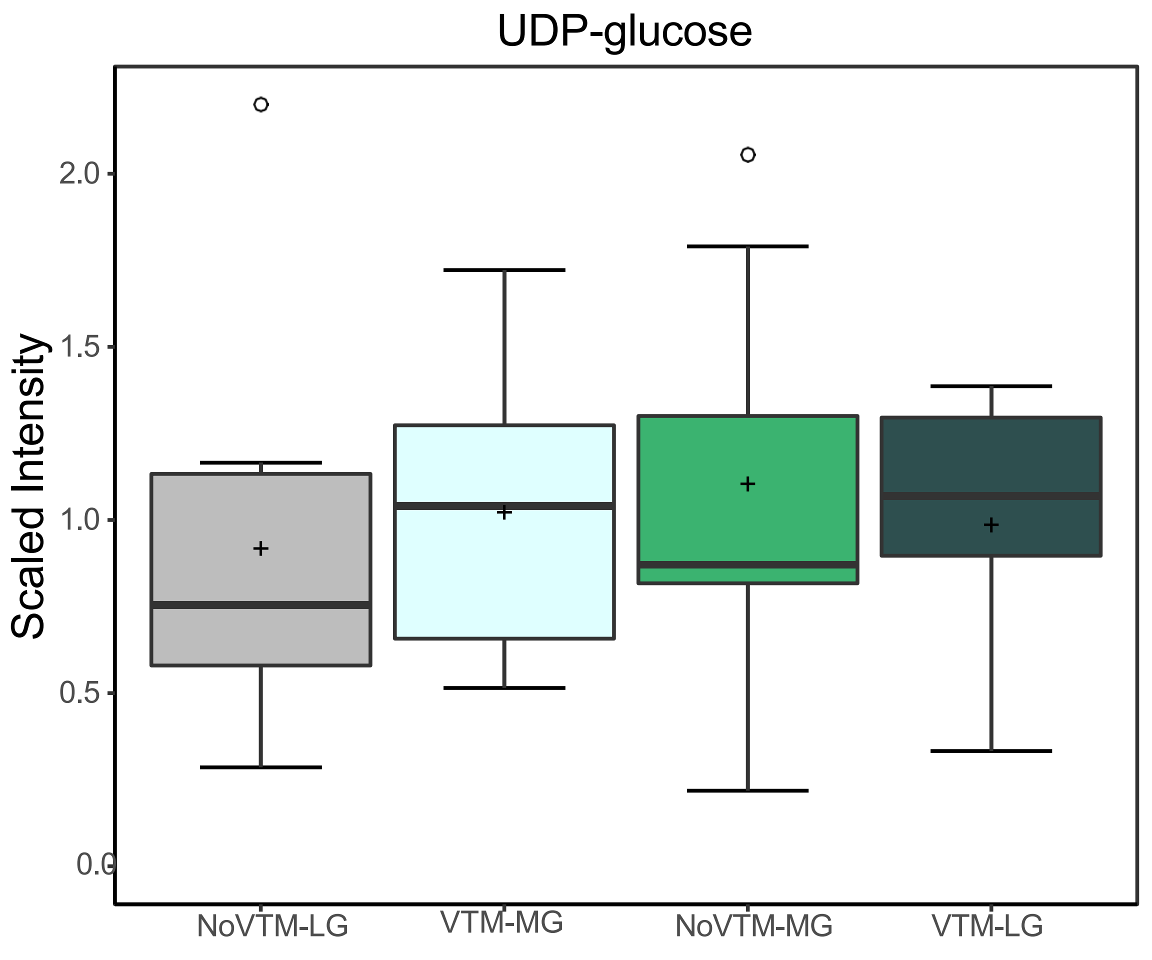

UDP-glucose
Scaled Intensity
2.0
1.5
1.0
0.5
0.0
NoVTM-LG
VTM-MG
NoVTM-MG
VTM-LG

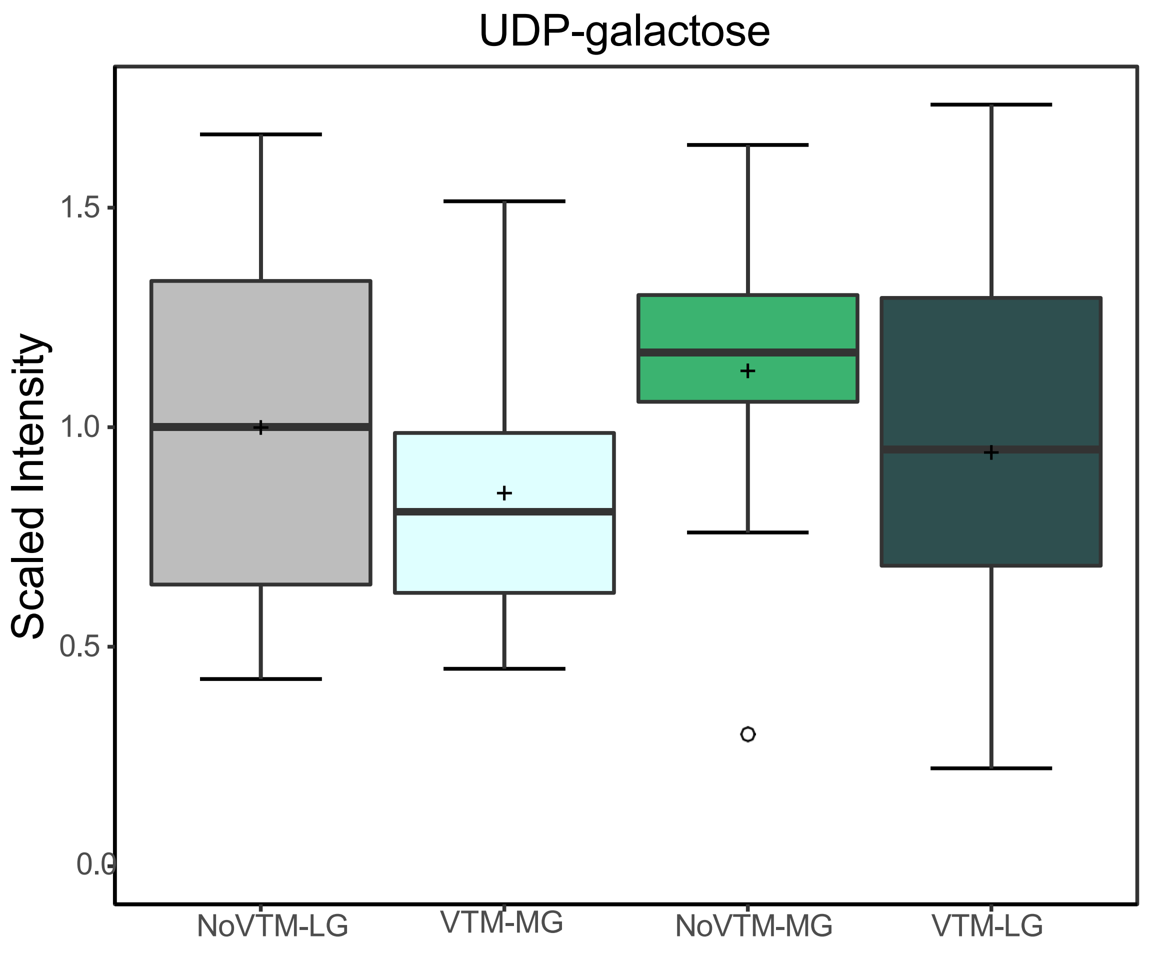
UDP-galactose
Scaled Intensity
1.5
1.0
0.5
0.0
NoVTM-LG
VTM-MG
NoVTM-MG
VTM-LG

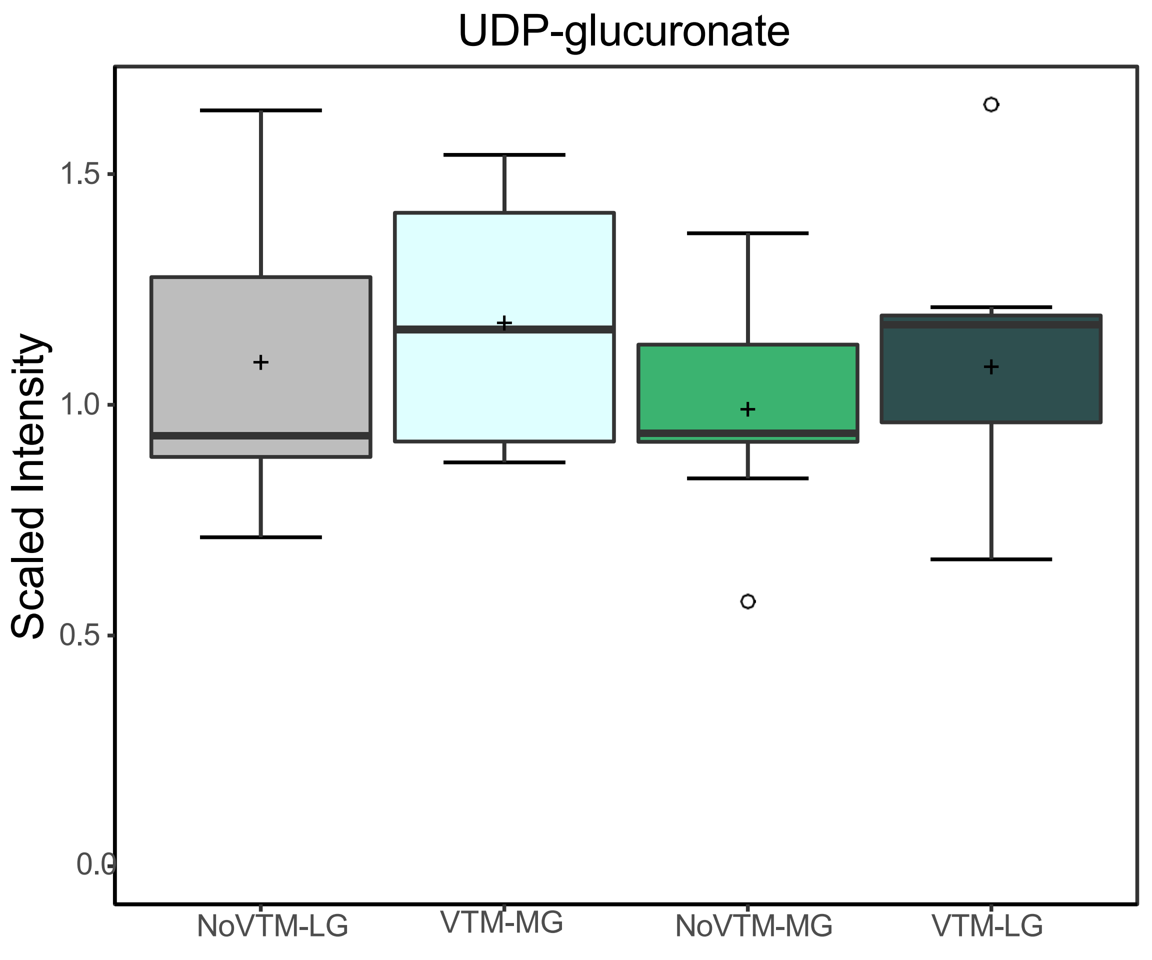

UDP-glucuronate
Scaled Intensity
1.5
1.0
0.5
0.0
NoVTM-LG
VTM-MG
NoVTM-MG
VTM-LG

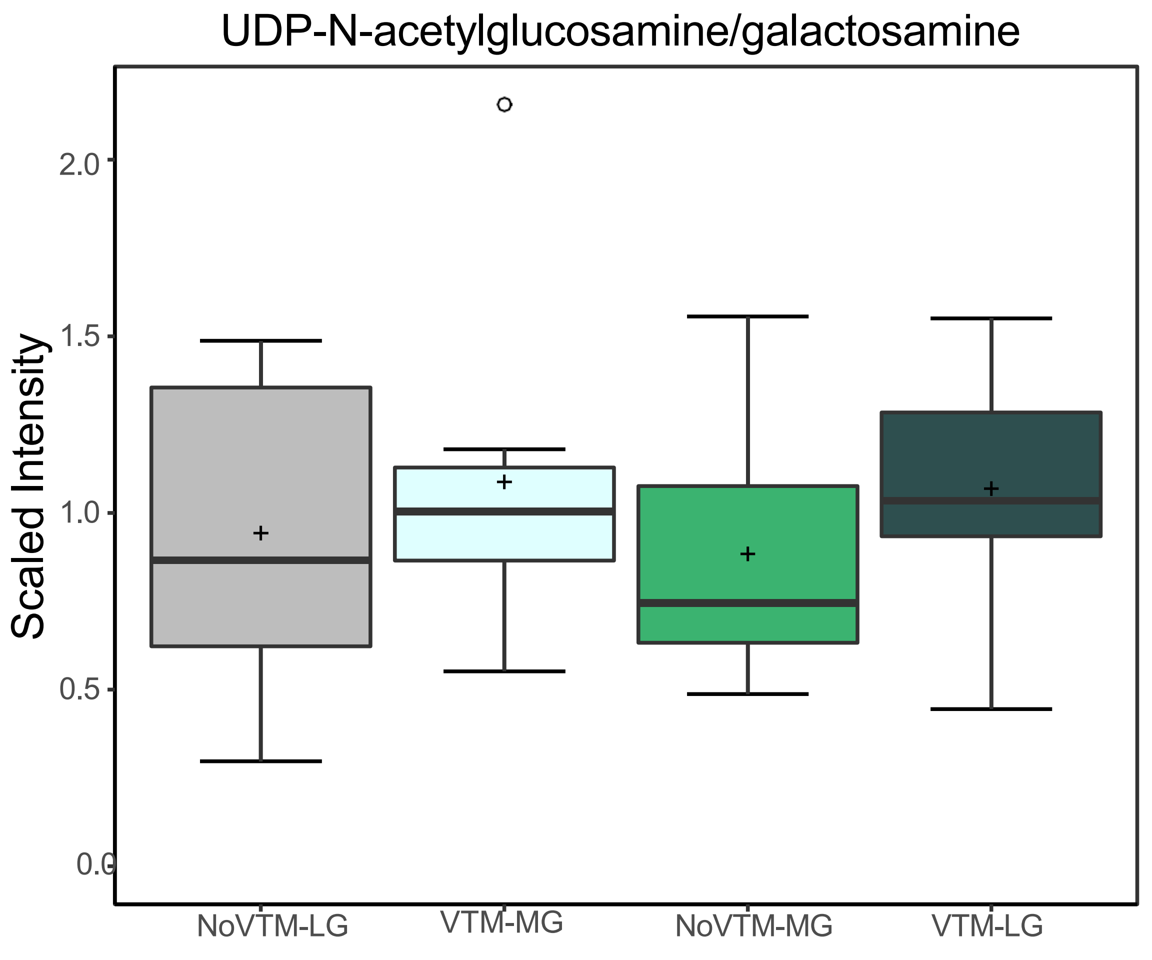

UDP-N-acetylglucosamine/galactosamine
Scaled Intensity
2.0
1.5
1.0
0.5
0.0
NoVTM-LG
VTM-MG
NoVTM-MG
VTM-LG

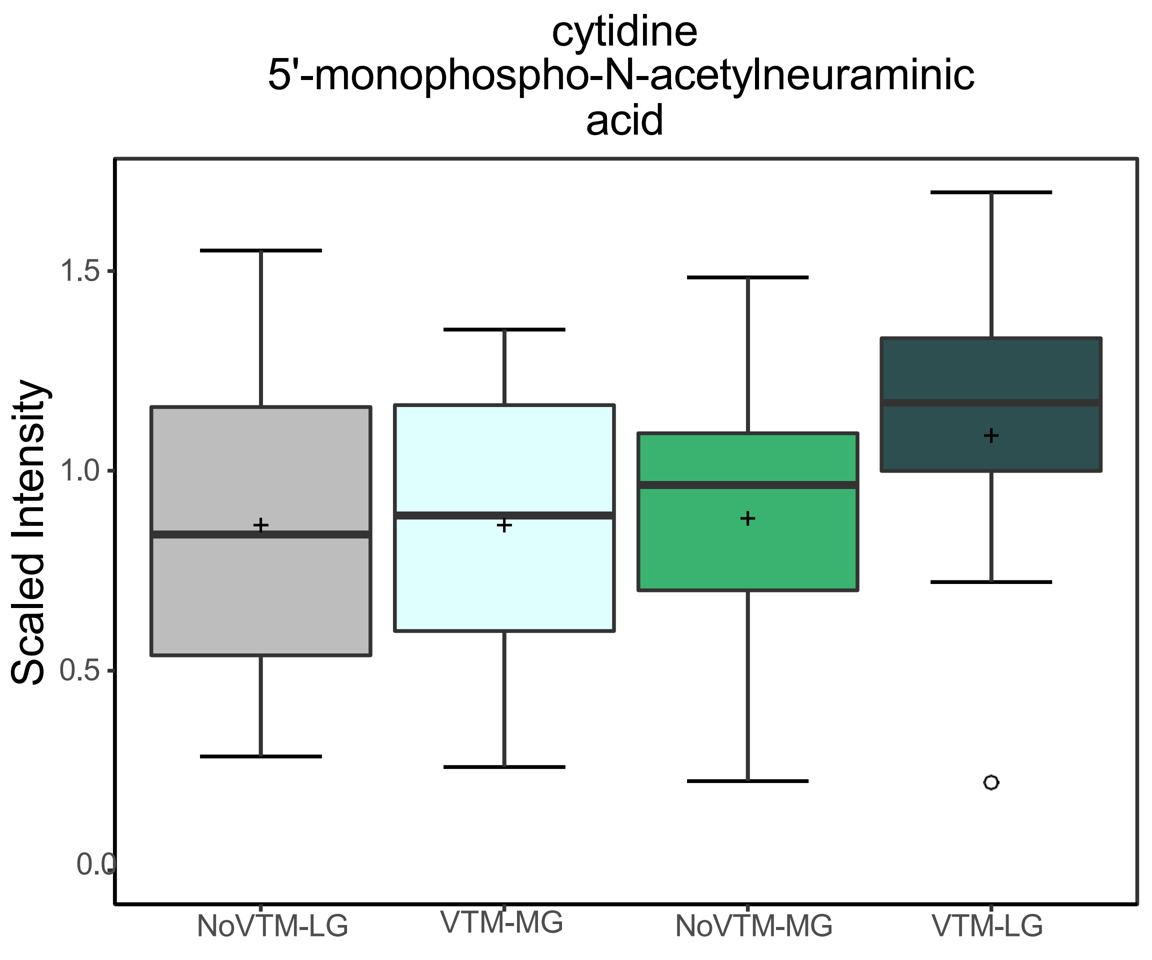

cytidine
5'-monophospho-N-acetylneuraminic
acid
Scaled Intensity
0.0
0.5
1.0
1.5
NoVTM-LG
VTM-MG
NoVTM-MG
VTM-LG

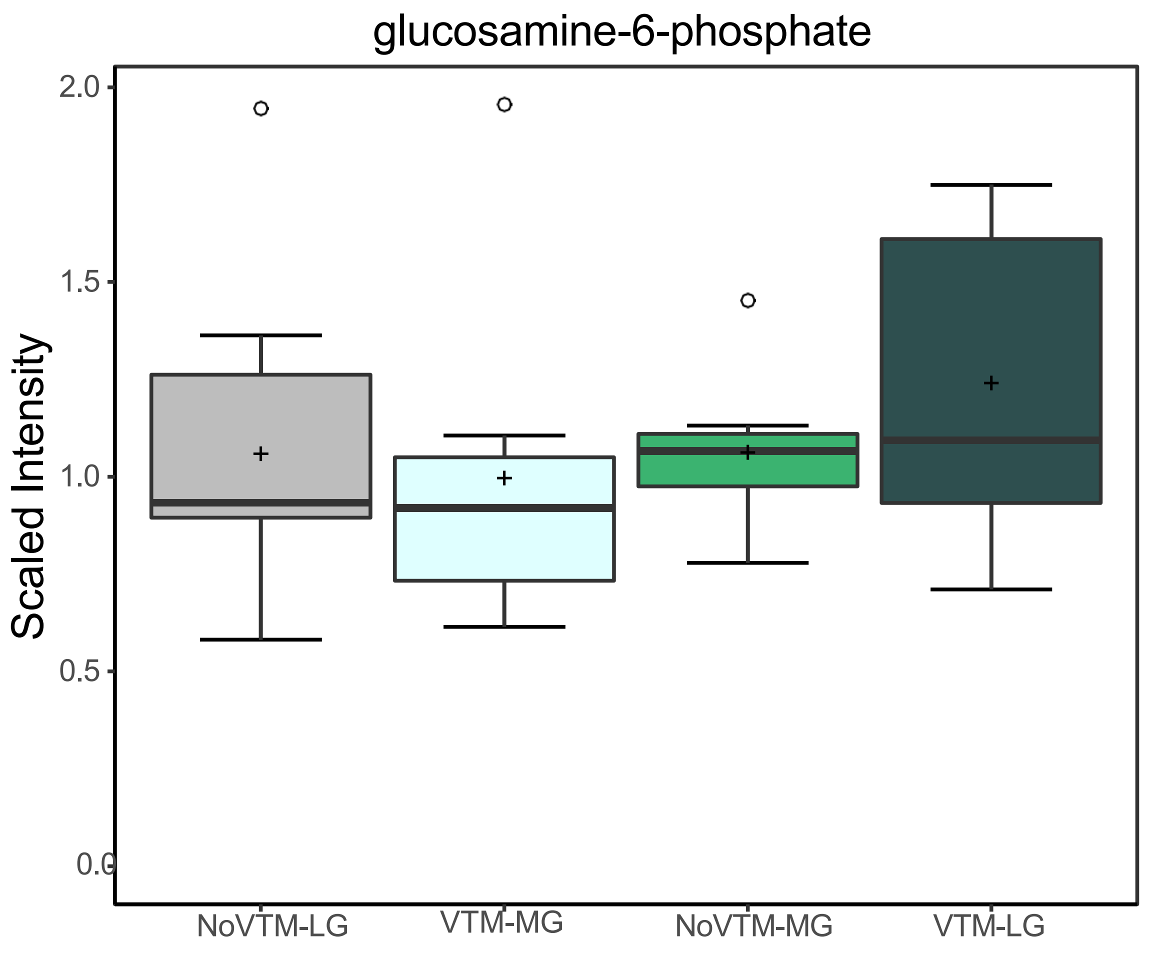

glucosamine-6-phosphate
Scaled Intensity
2.0
1.5
1.0
0.5
0.0
NoVTM-LG
VTM-MG
NoVTM-MG
VTM-LG

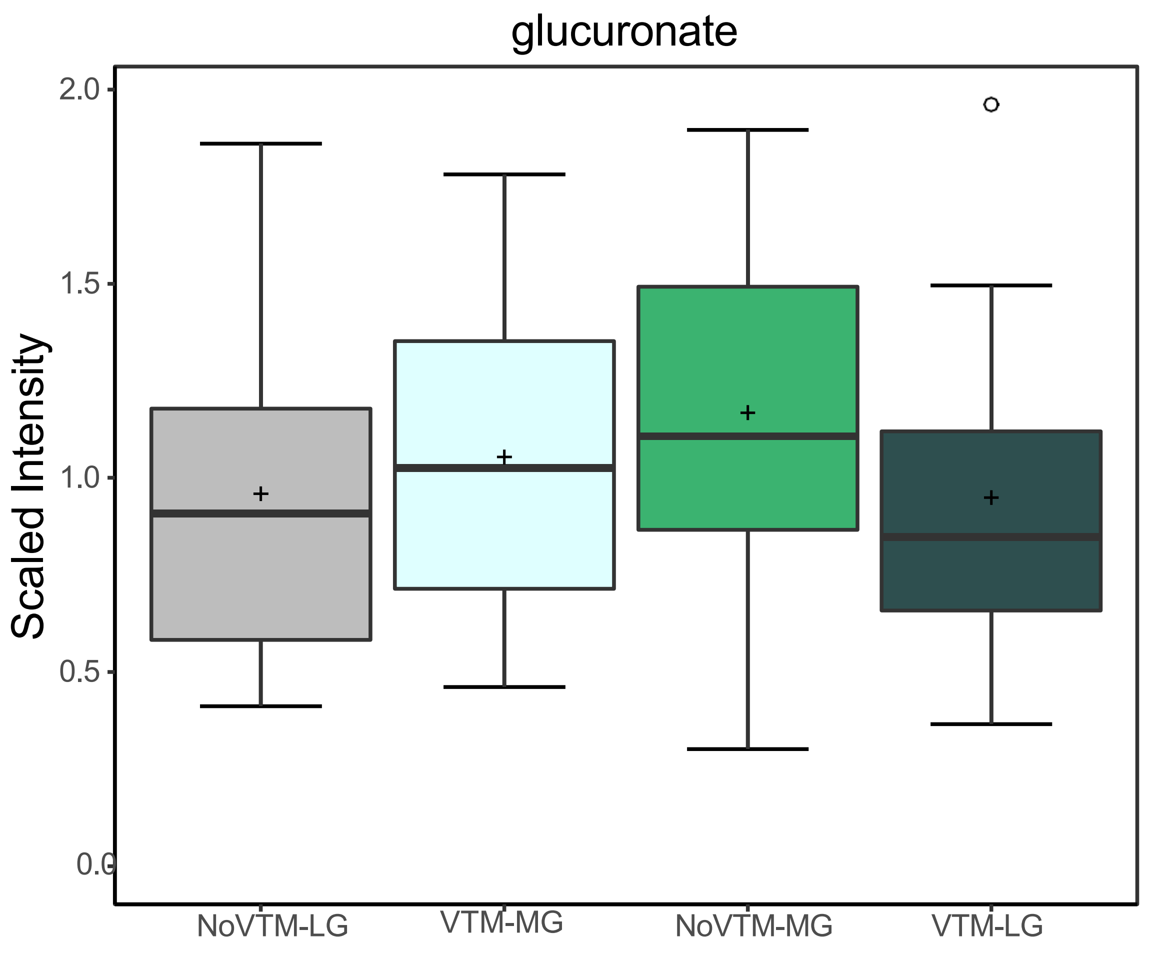
glucuronate
Scaled Intensity
2.0
1.5
1.0
0.5
0.0
NoVTM-LG
VTM-MG
NoVTM-MG
VTM-LG

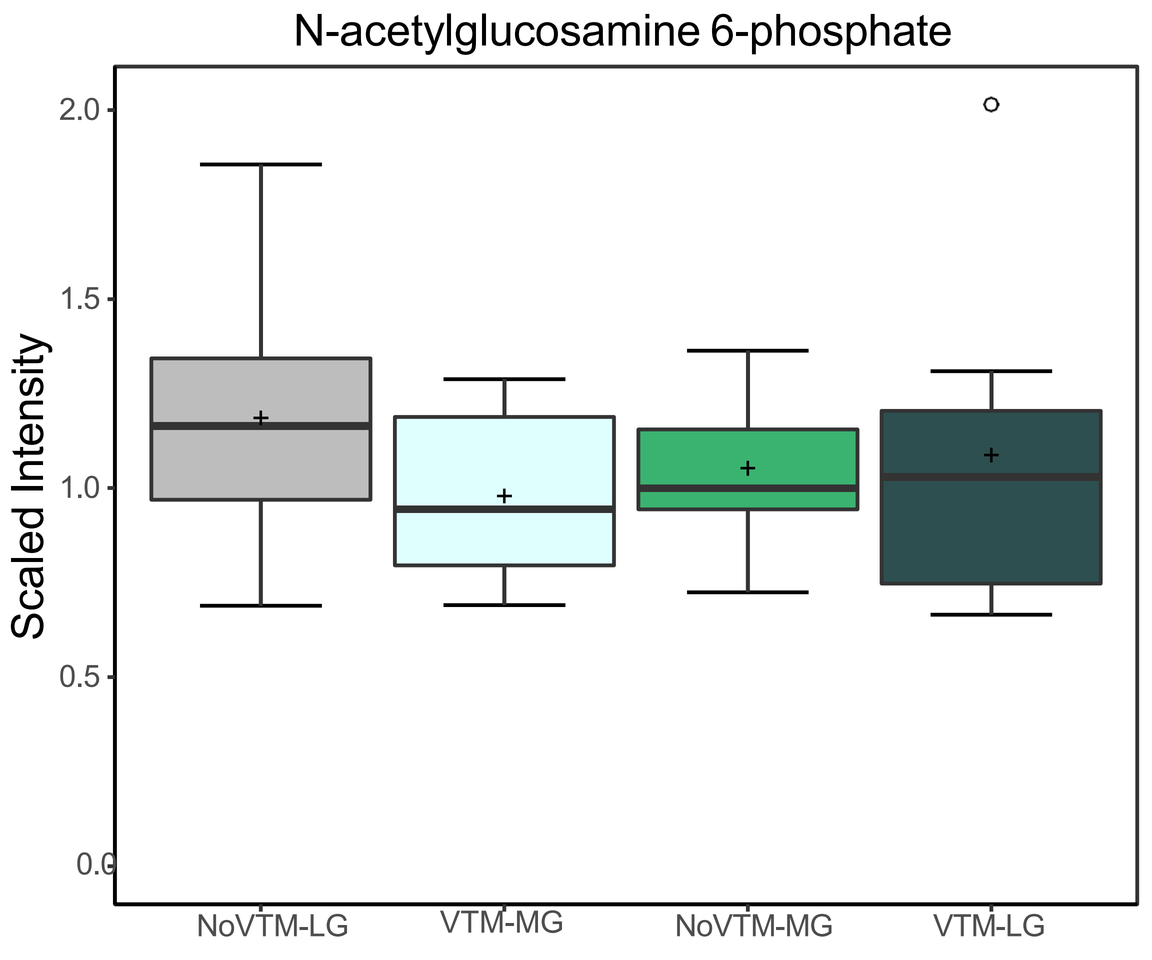
N-acetylglucosamine 6-phosphate
Scaled Intensity
2.0
1.5
1.0
0.5
0.0
NoVTM-LG
VTM-MG
NoVTM-MG
VTM-LG

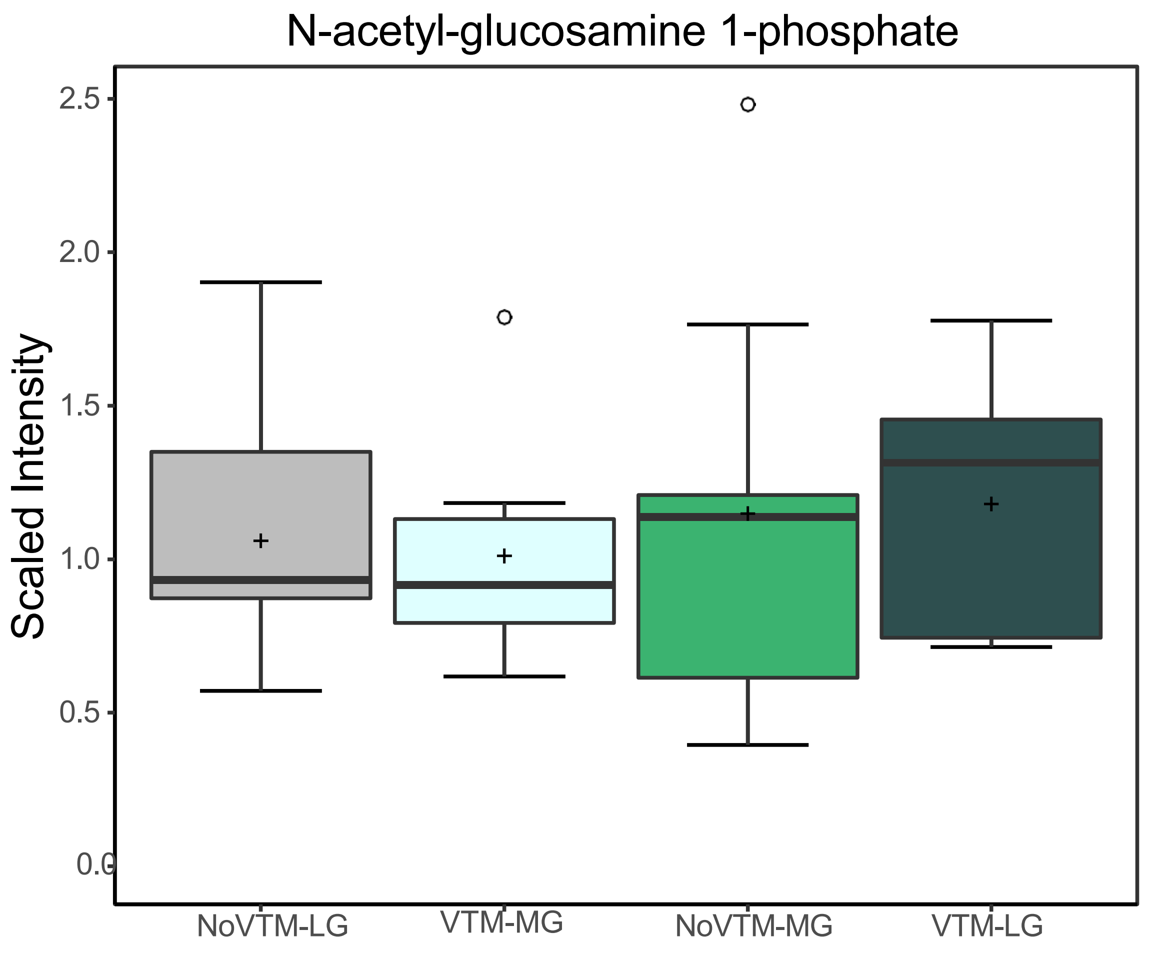

N-acetyl-glucosamine 1-phosphate
Scaled Intensity
2.5
2.0
1.5
1.0
0.5
0.0
NoVTM-LG
VTM-MG
NoVTM-MG
VTM-LG

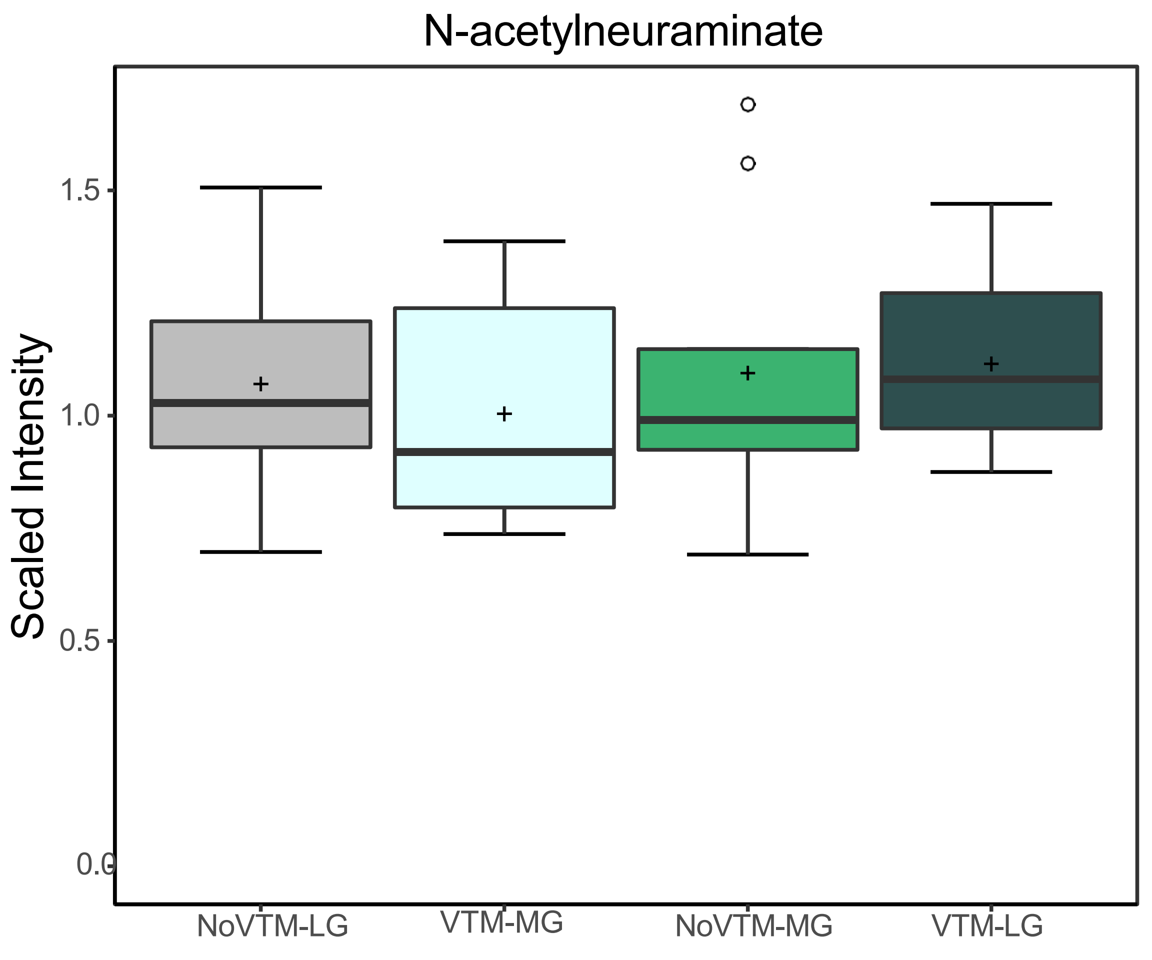

N-acetylneuraminate
Scaled Intensity
1.5
1.0
0.5
0.0
NoVTM-LG
VTM-MG
NoVTM-MG
VTM-LG

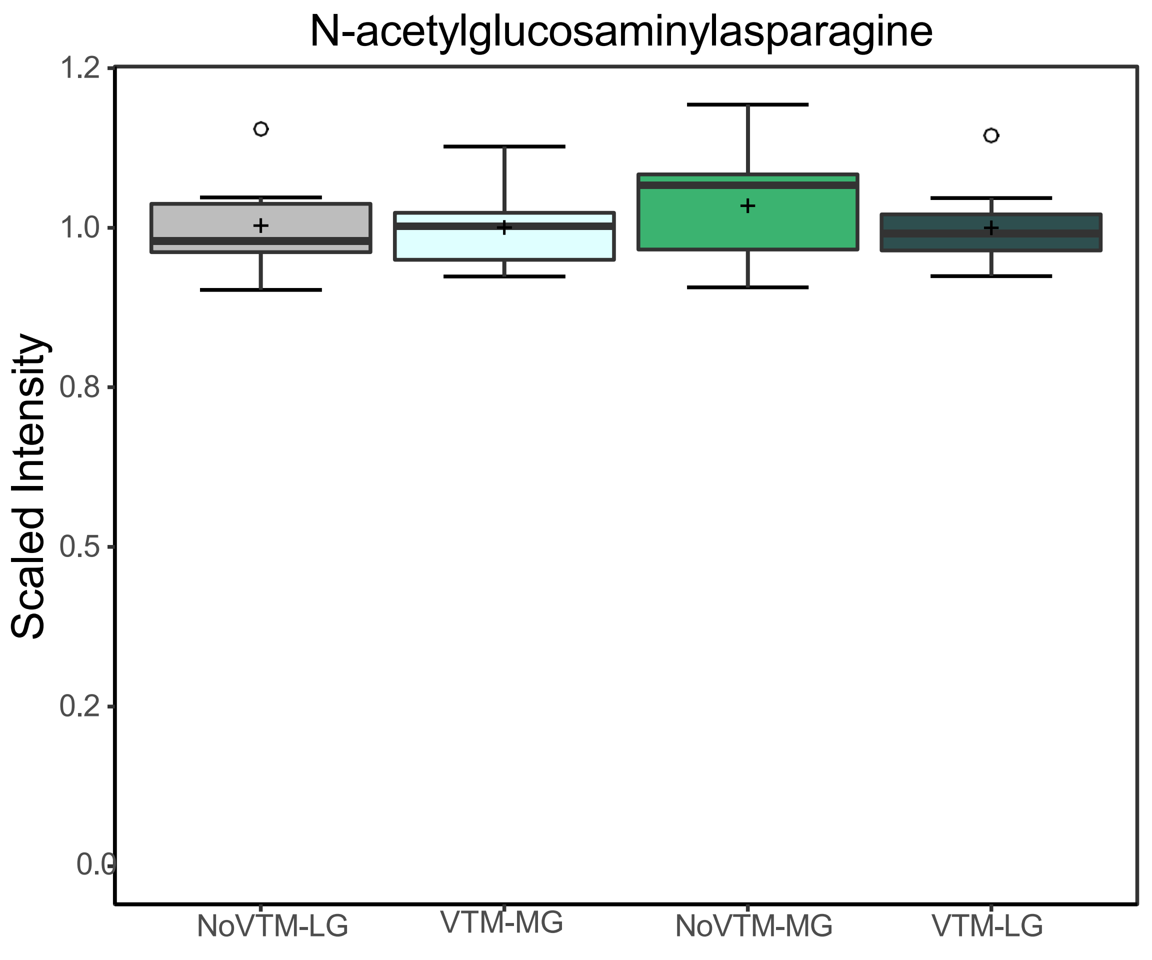
N-acetylglucosaminylasparagine
Scaled Intensity
1.2
1.0
0.8
0.5
0.2
0.0
NoVTM-LG
VTM-MG
NoVTM-MG
VTM-LG

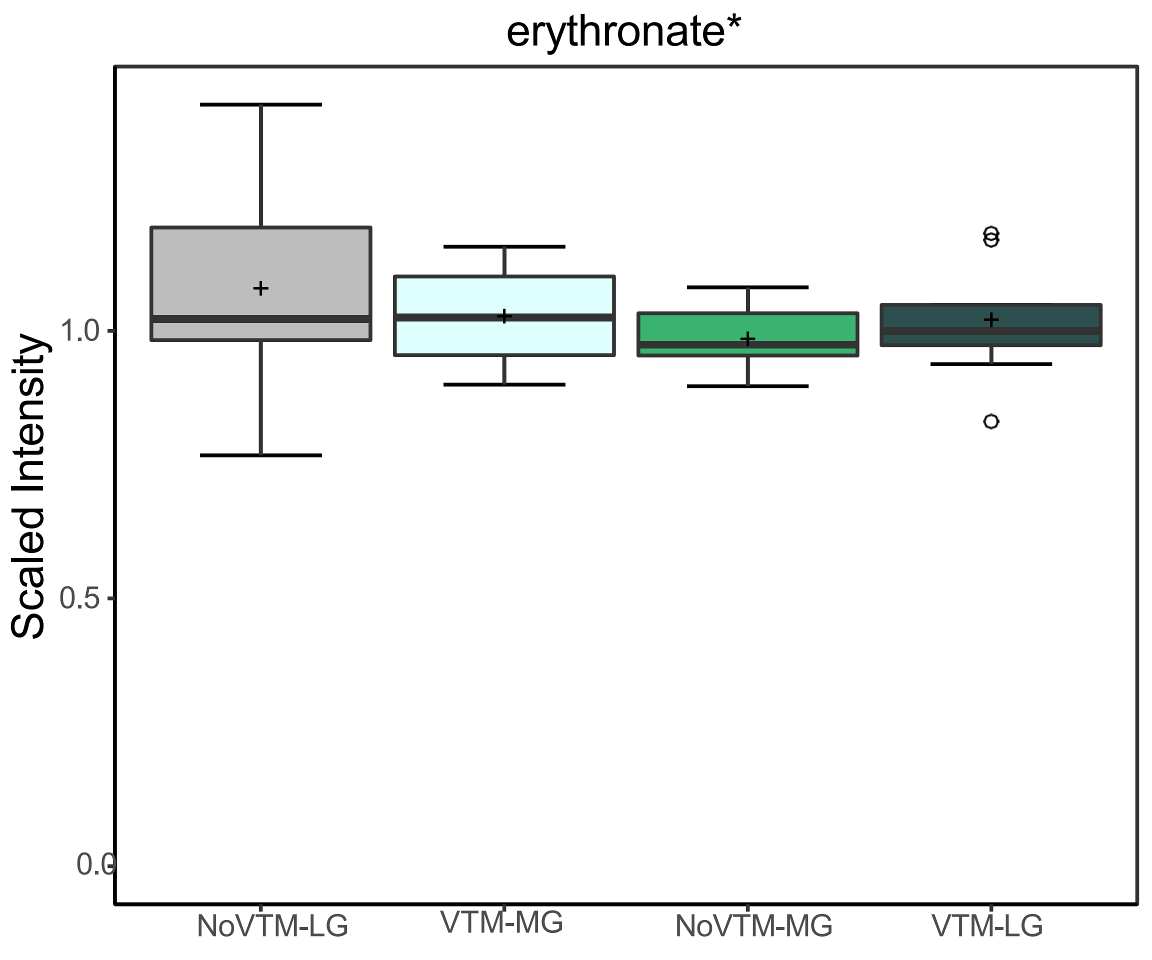

erythronate*
Scaled Intensity
1.0
0.5
0.0
NoVTM-LG
VTM-MG
NoVTM-MG
VTM-LG

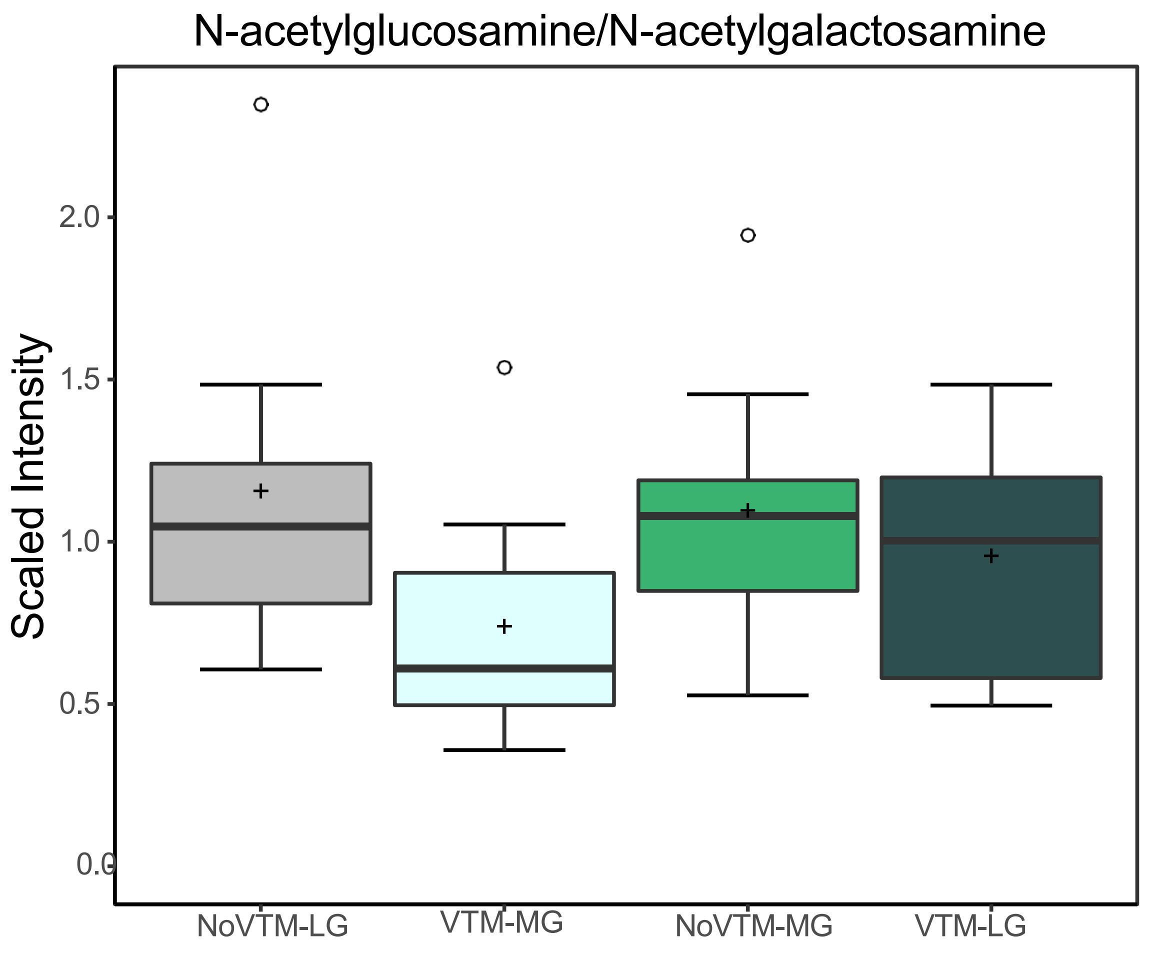
N-acetylglucosamine/N-acetylgalactosamine
Scaled Intensity
2.0
1.5
1.0
0.5
0.0
NoVTM-LG
VTM-MG
NoVTM-MG
VTM-LG

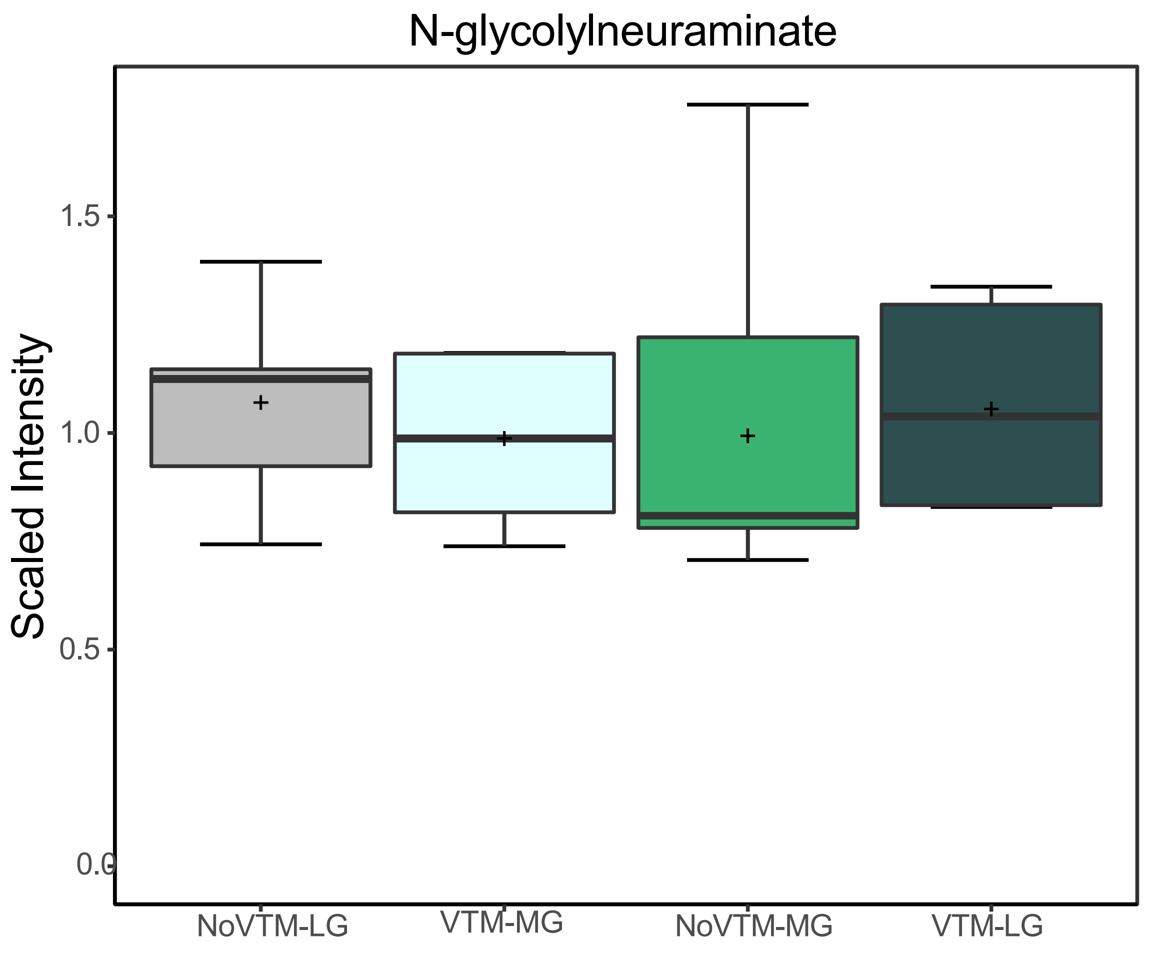
N-glycolylneuraminate
Scaled Intensity
1.5
1.0
0.5
0.0
NoVTM-LG
VTM-MG
NoVTM-MG
VTM-LG

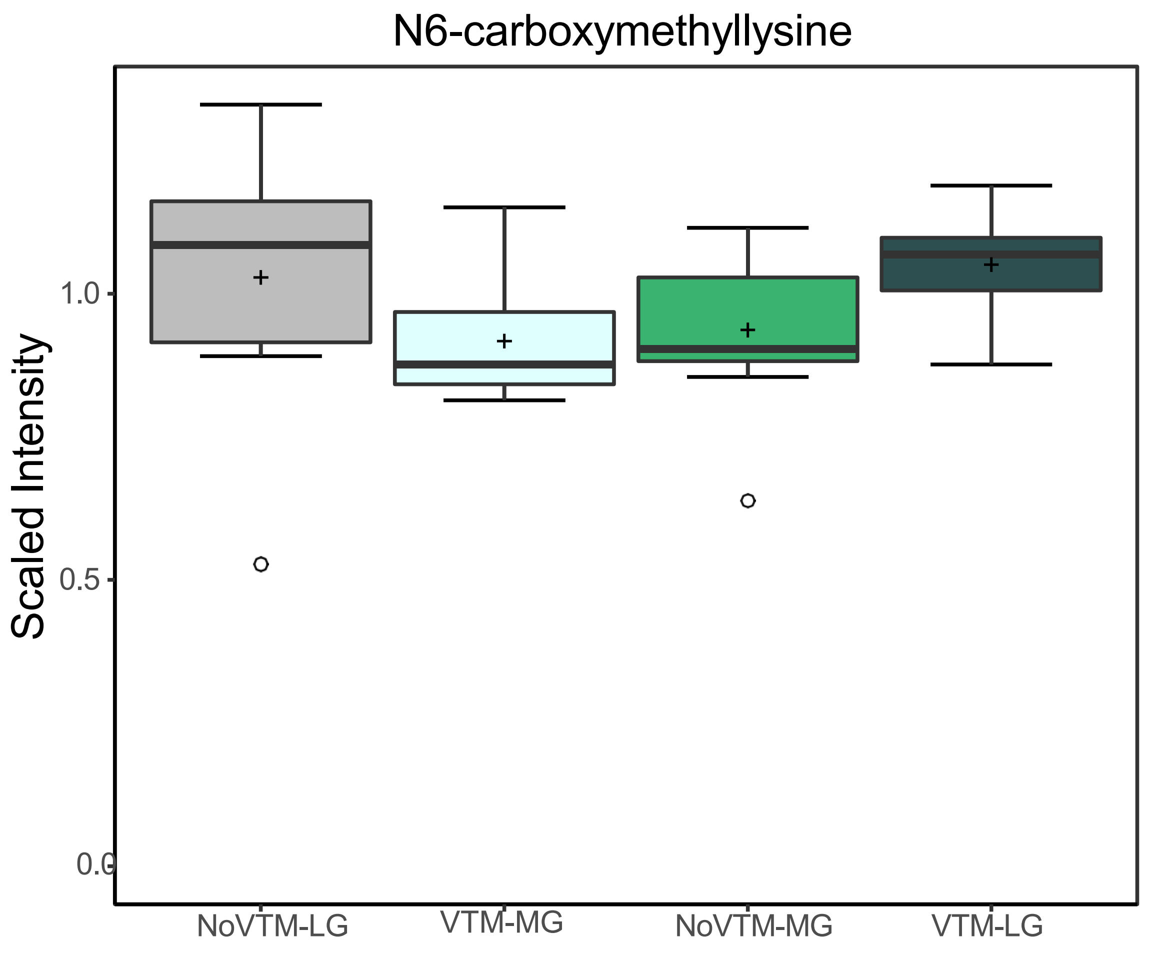
N6-carboxymethyllysine
Scaled Intensity
1.0
0.5
0.0
NoVTM-LG
VTM-MG
NoVTM-MG
VTM-LG

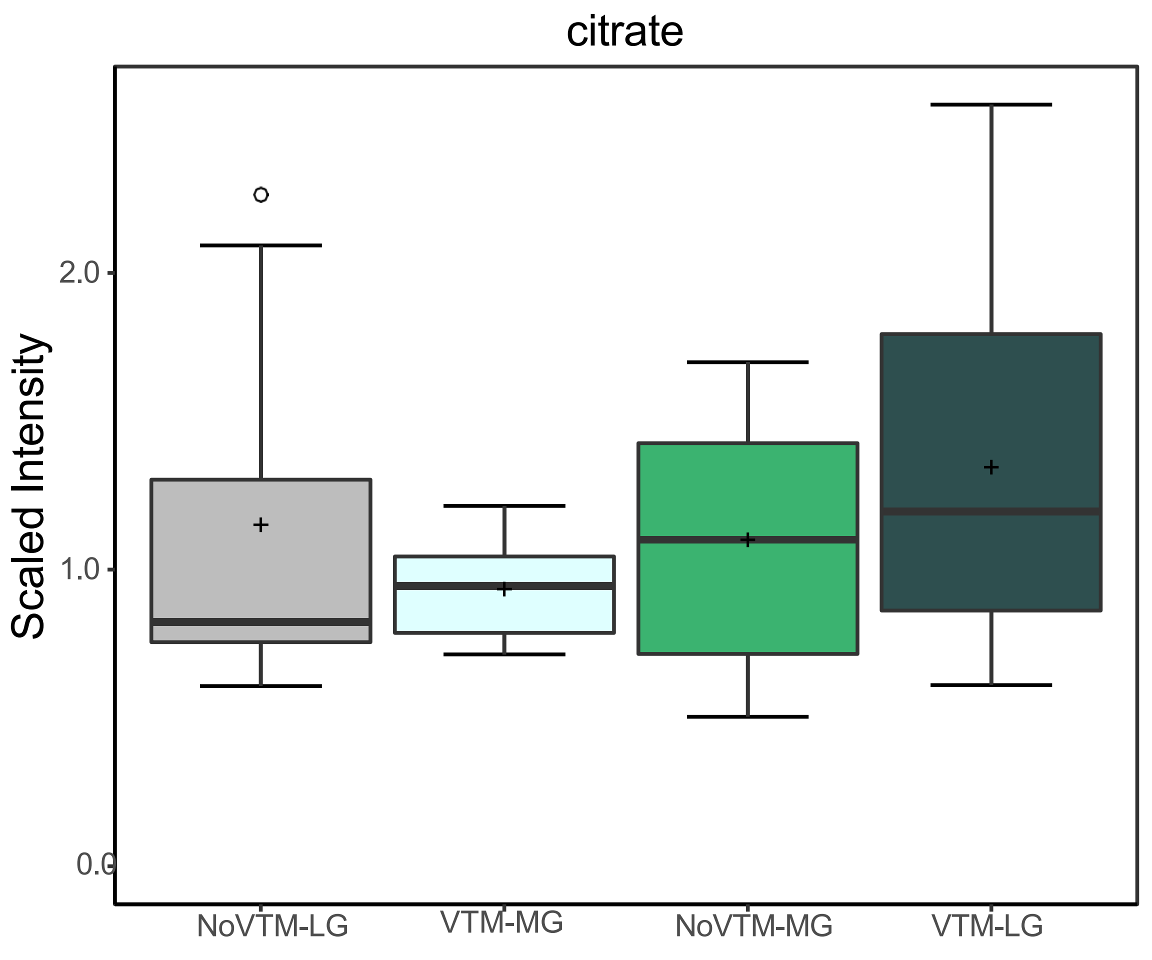

citrate
Scaled Intensity
2.0
1.0
0.0
NoVTM-LG
VTM-MG
NoVTM-MG
VTM-LG

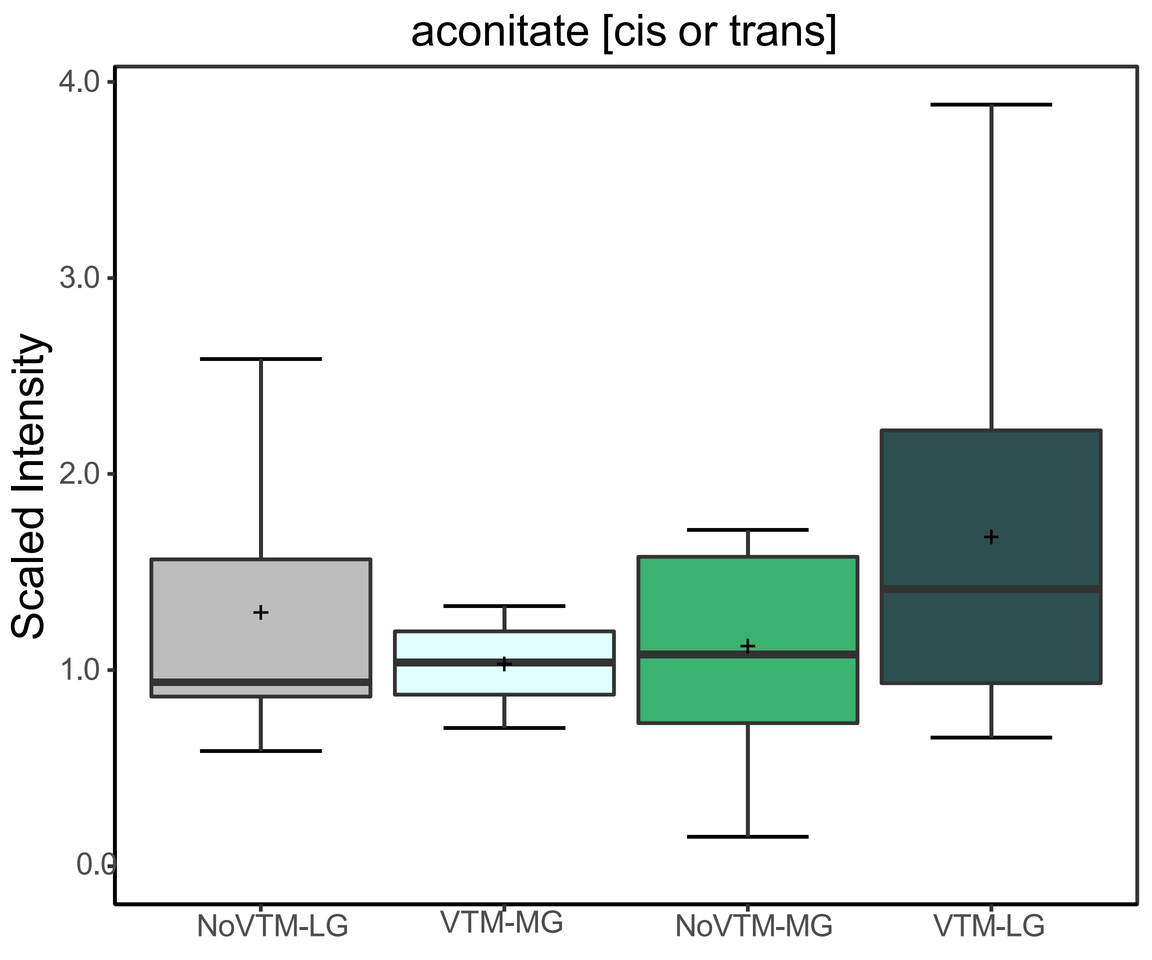
aconitate [cis or trans]
Scaled Intensity
4.0
3.0
2.0
1.0
0.0
NoVTM-LG
VTM-MG
NoVTM-MG
VTM-LG

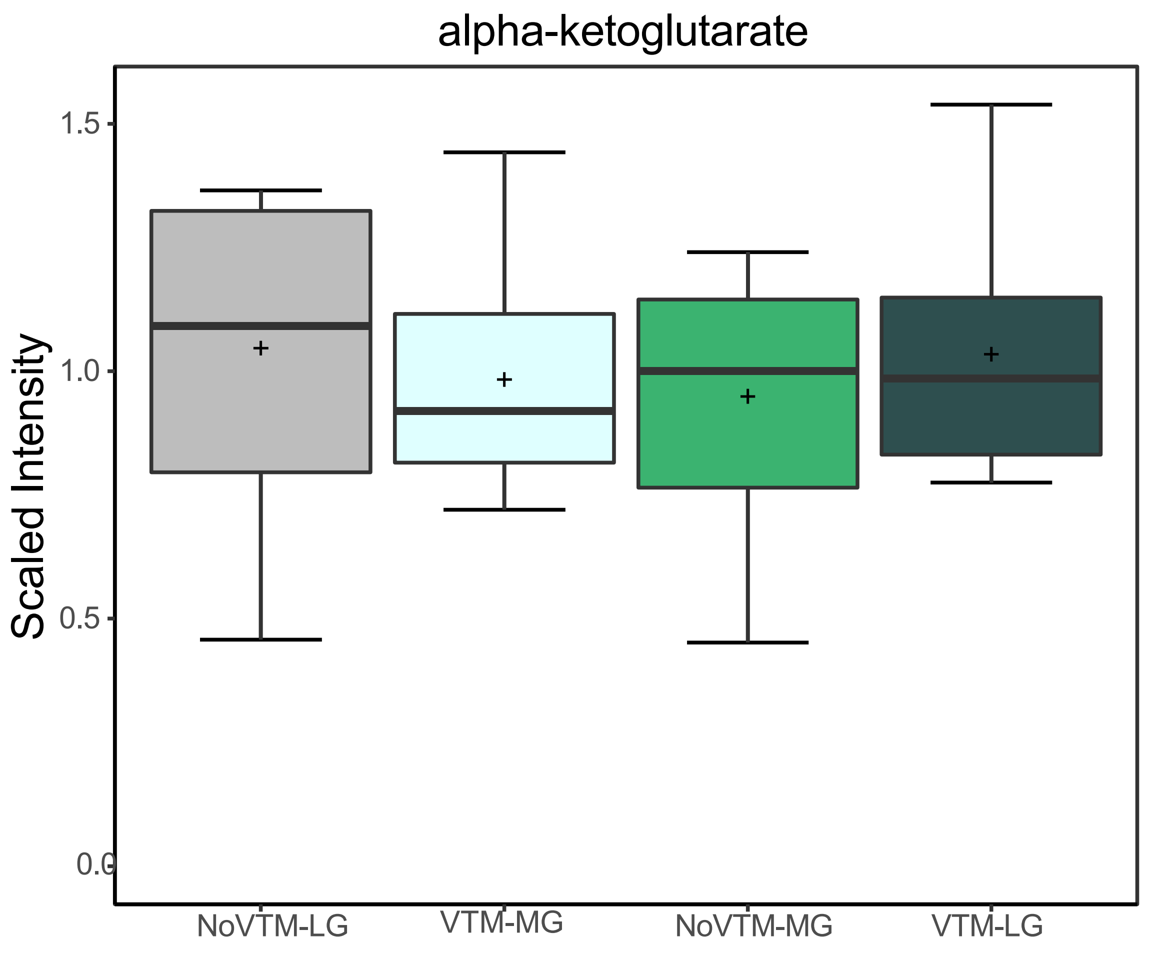

alpha-ketoglutarate
Scaled Intensity
1.5
1.0
0.5
0.0
NoVTM-LG
VTM-MG
NoVTM-MG
VTM-LG

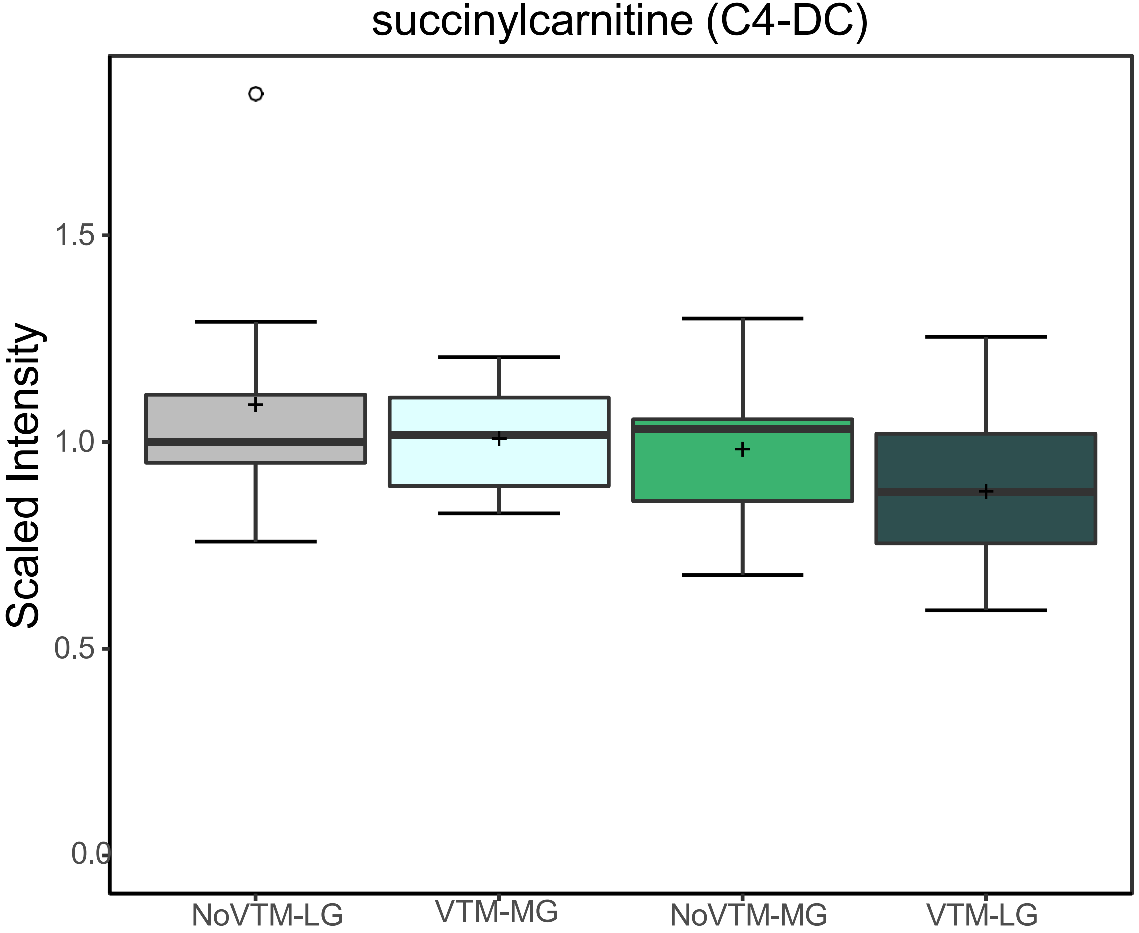
succinylcarnitine (C4-DC)
Scaled Intensity
1.5
1.0
0.5
0.0
NoVTM-LG
VTM-MG
NoVTM-MG
VTM-LG

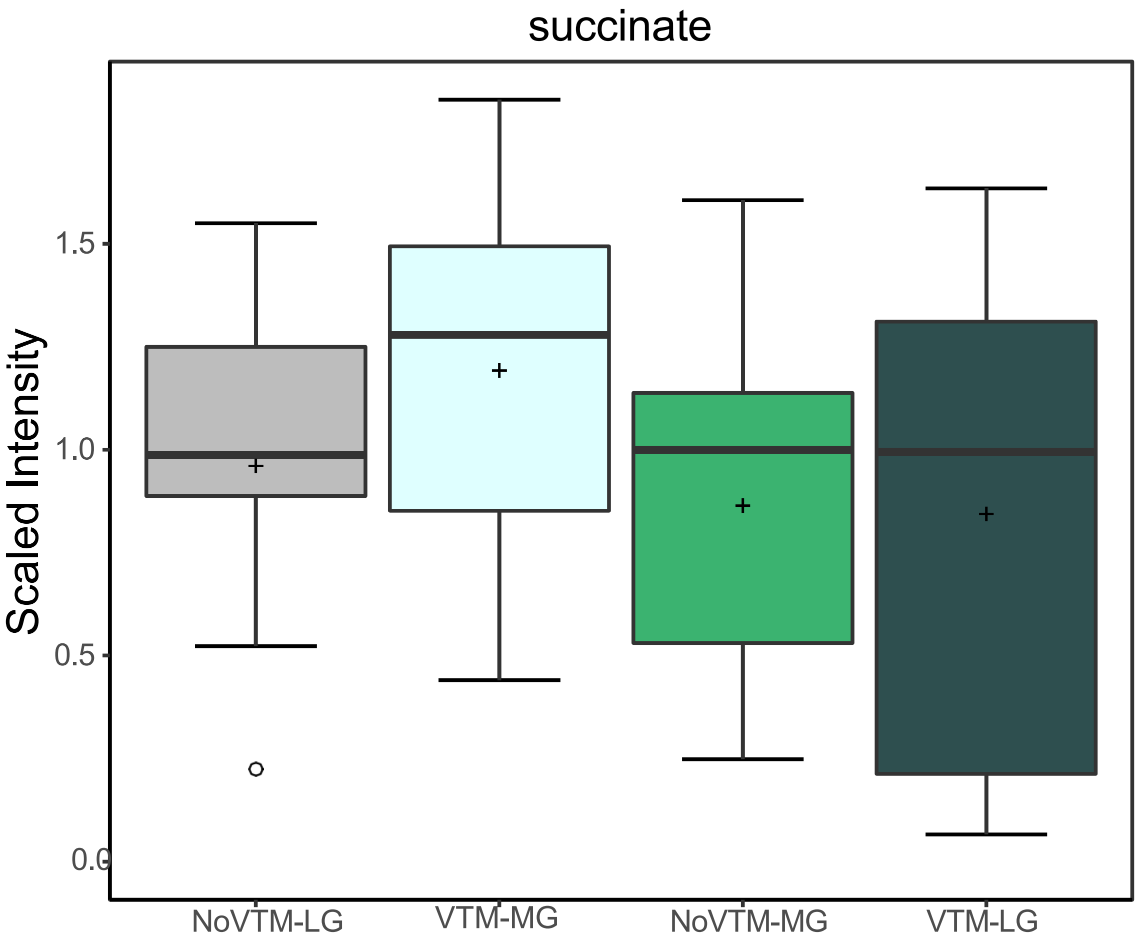

succinate
Scaled Intensity
1.5
1.0
0.5
0.0
NoVTM-LG
VTM-MG
NoVTM-MG
VTM-LG

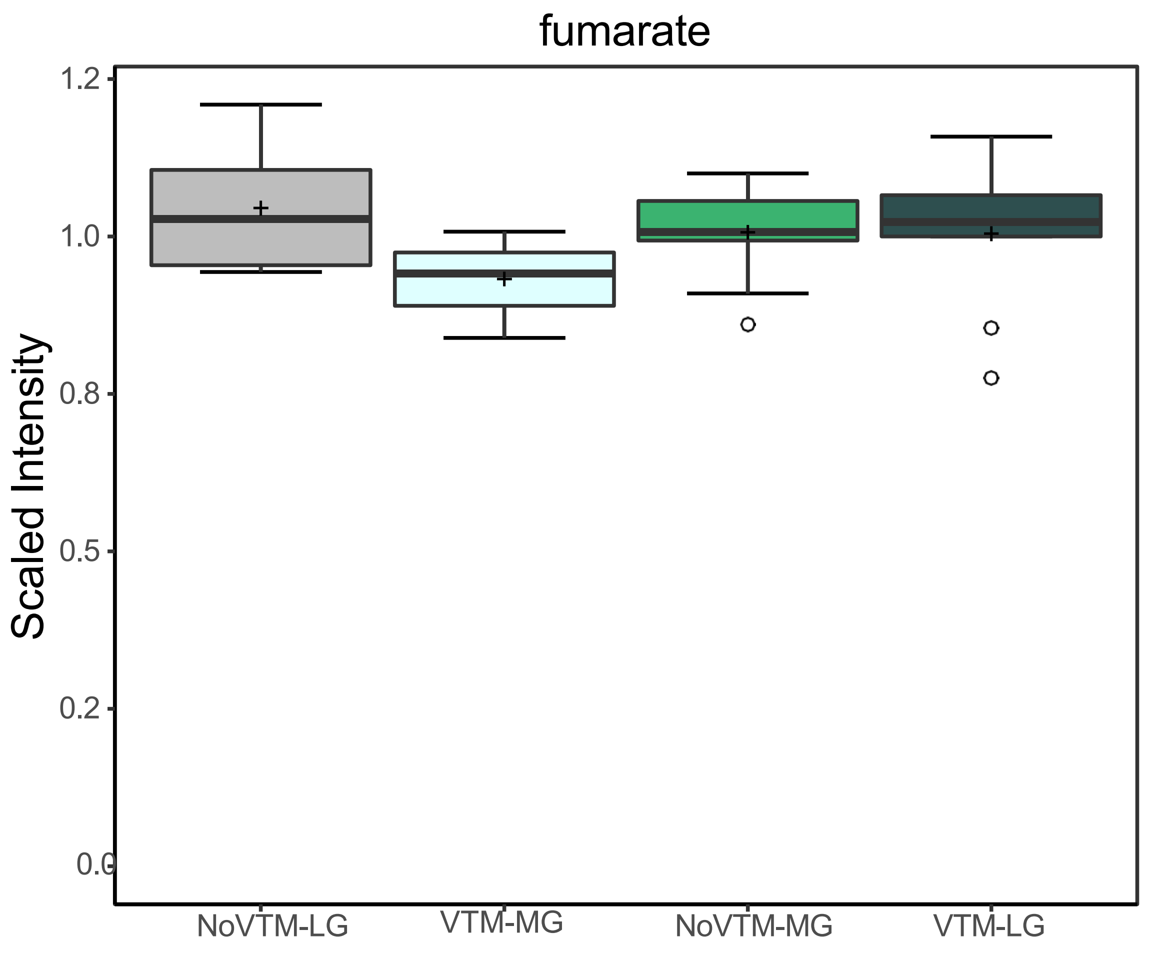
fumarate
Scaled Intensity
1.2
1.0
0.8
0.5
0.2
0.0
NoVTM-LG
VTM-MG
NoVTM-MG
VTM-LG

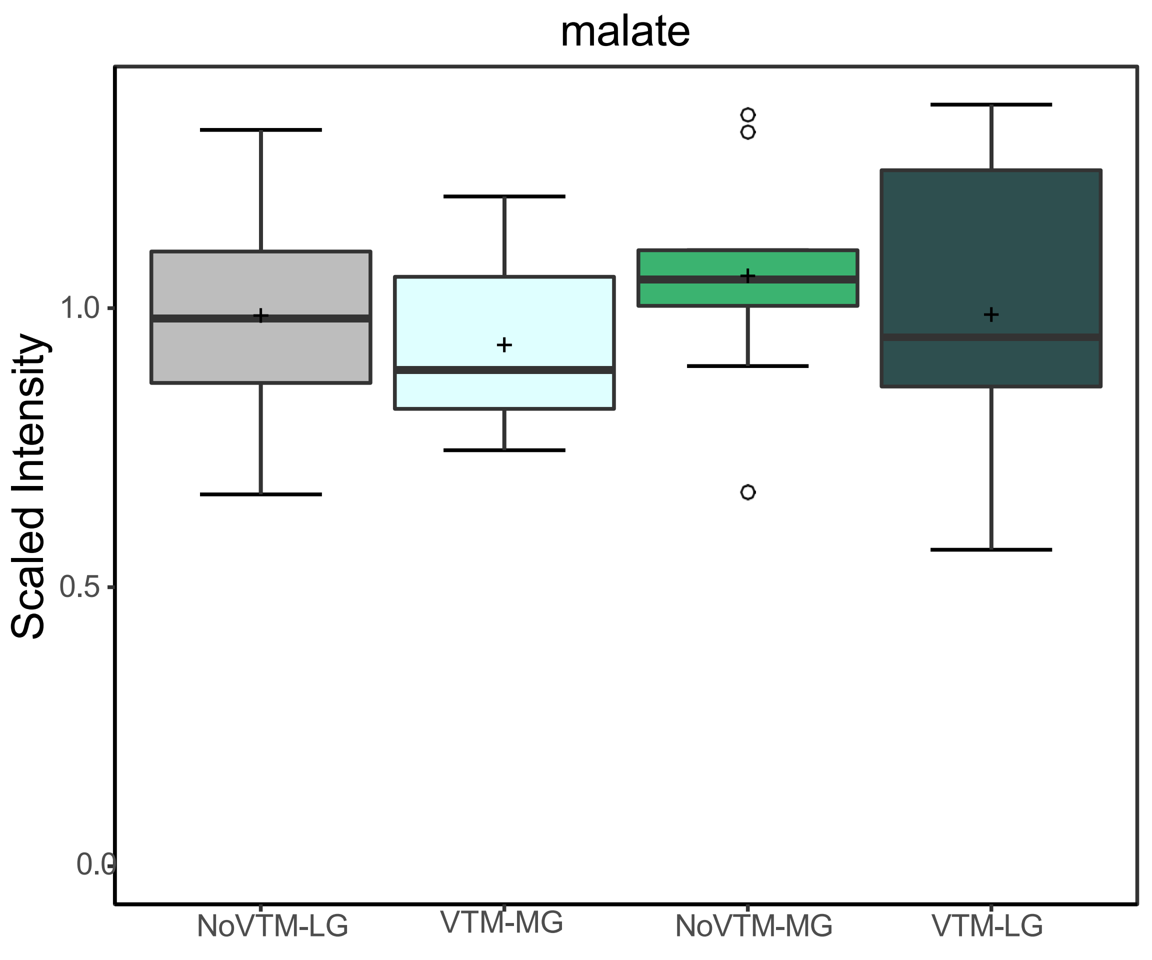

malate
Scaled Intensity
1.0
0.5
0.0
NoVTM-LG
VTM-MG
NoVTM-MG
VTM-LG

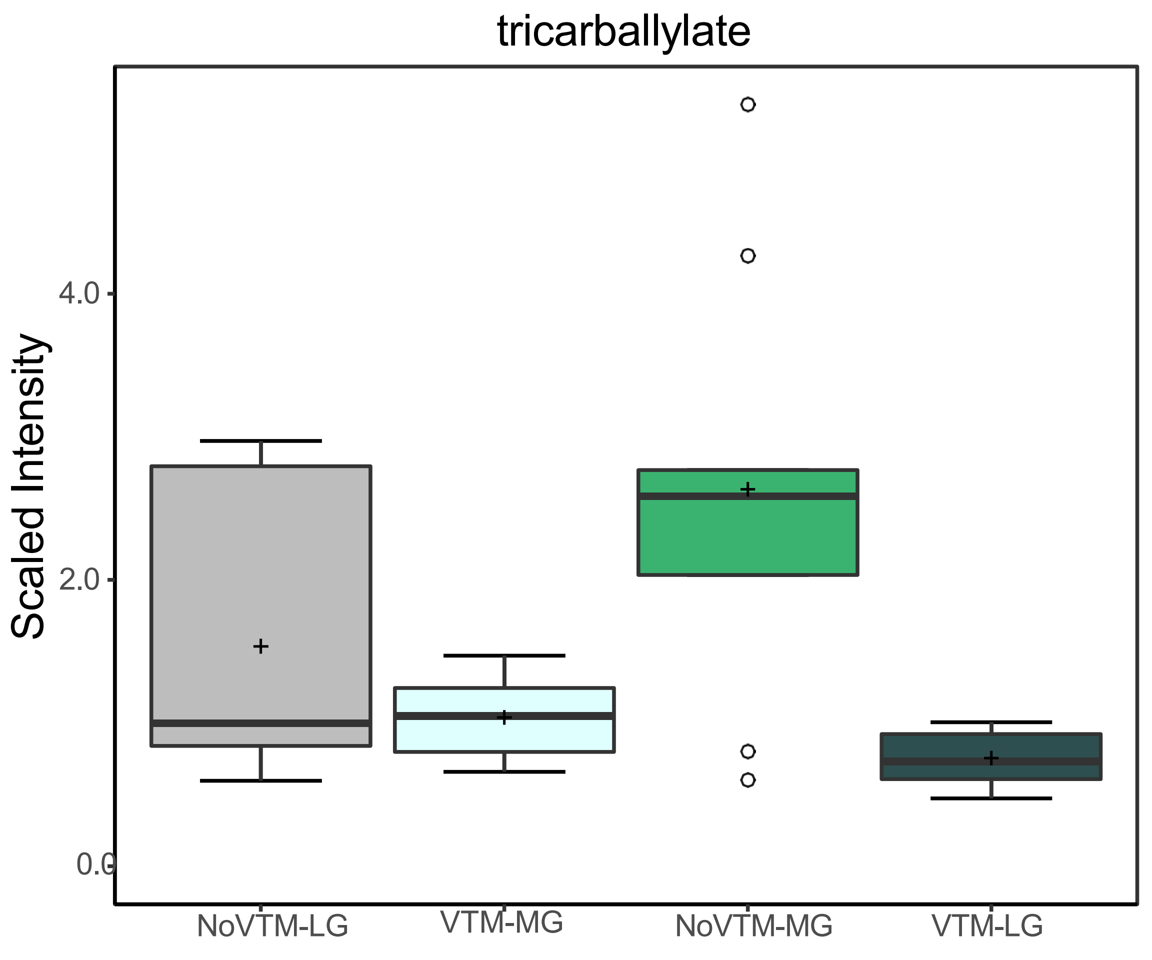

tricarballylate
Scaled Intensity
4.0
2.0
0.0
NoVTM-LG
VTM-MG
NoVTM-MG
VTM-LG

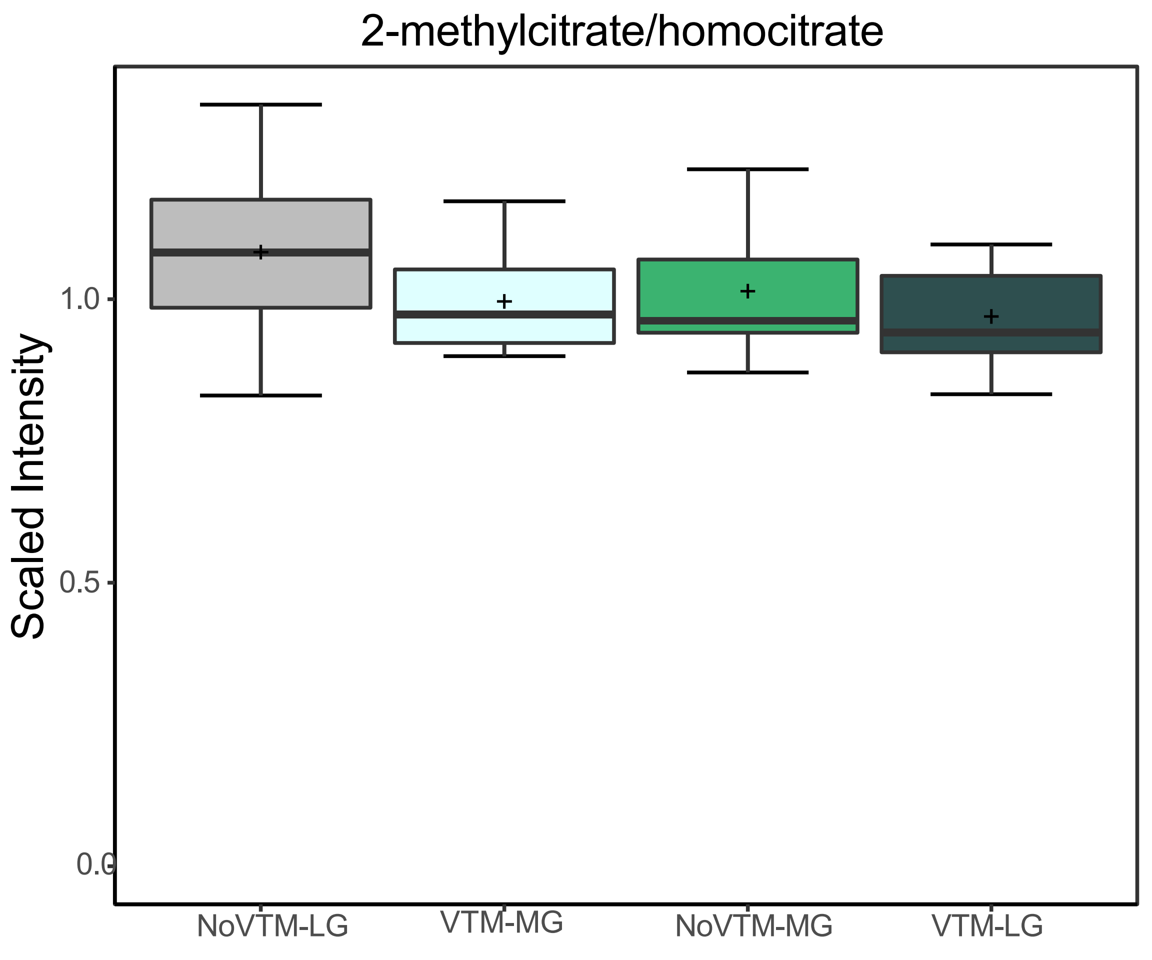

2-methylcitrate/homocitrate
Scaled Intensity
1.0
0.5
0.0
NoVTM-LG
VTM-MG
NoVTM-MG
VTM-LG

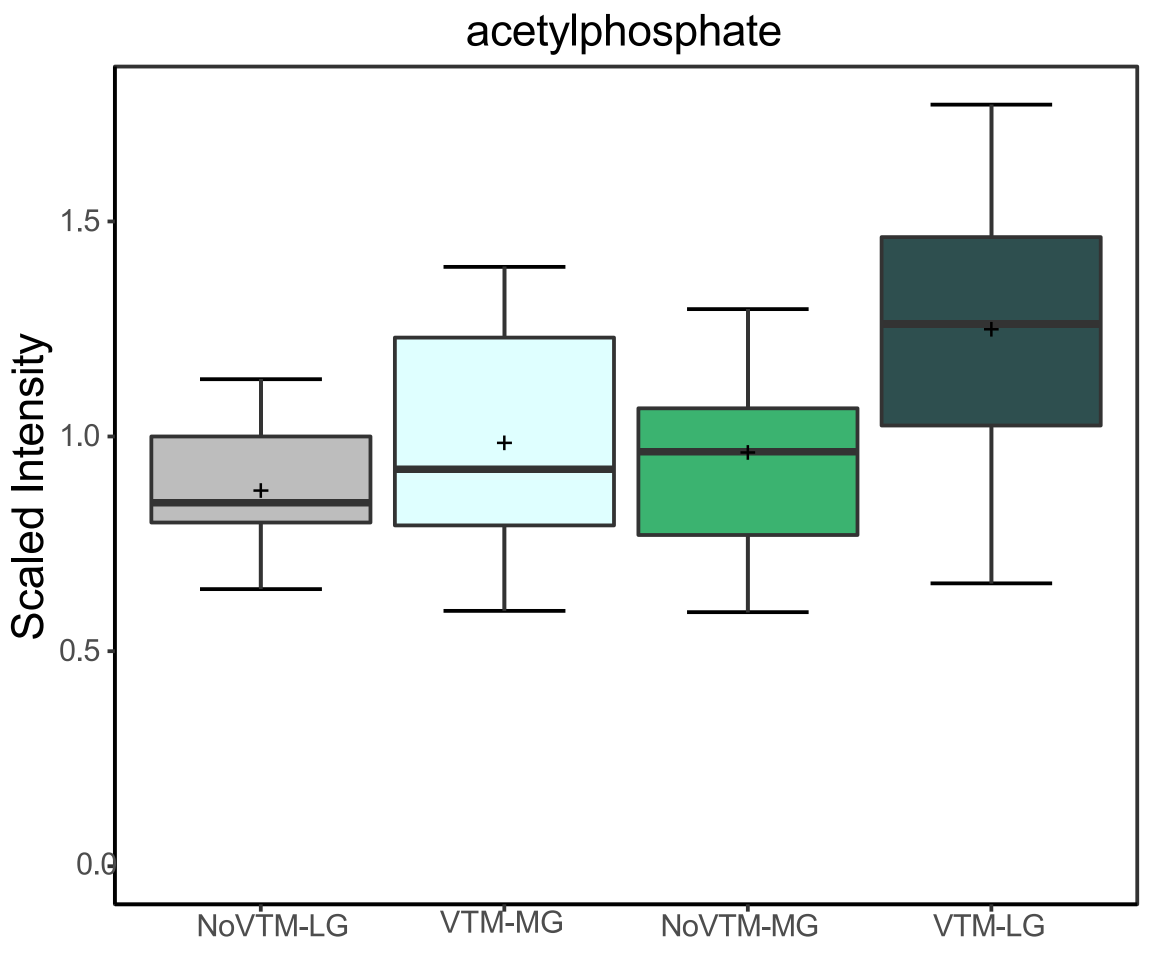
acetylphosphate
Scaled Intensity
1.5
1.0
0.5
0.0
NoVTM-LG
VTM-MG
NoVTM-MG
VTM-LG

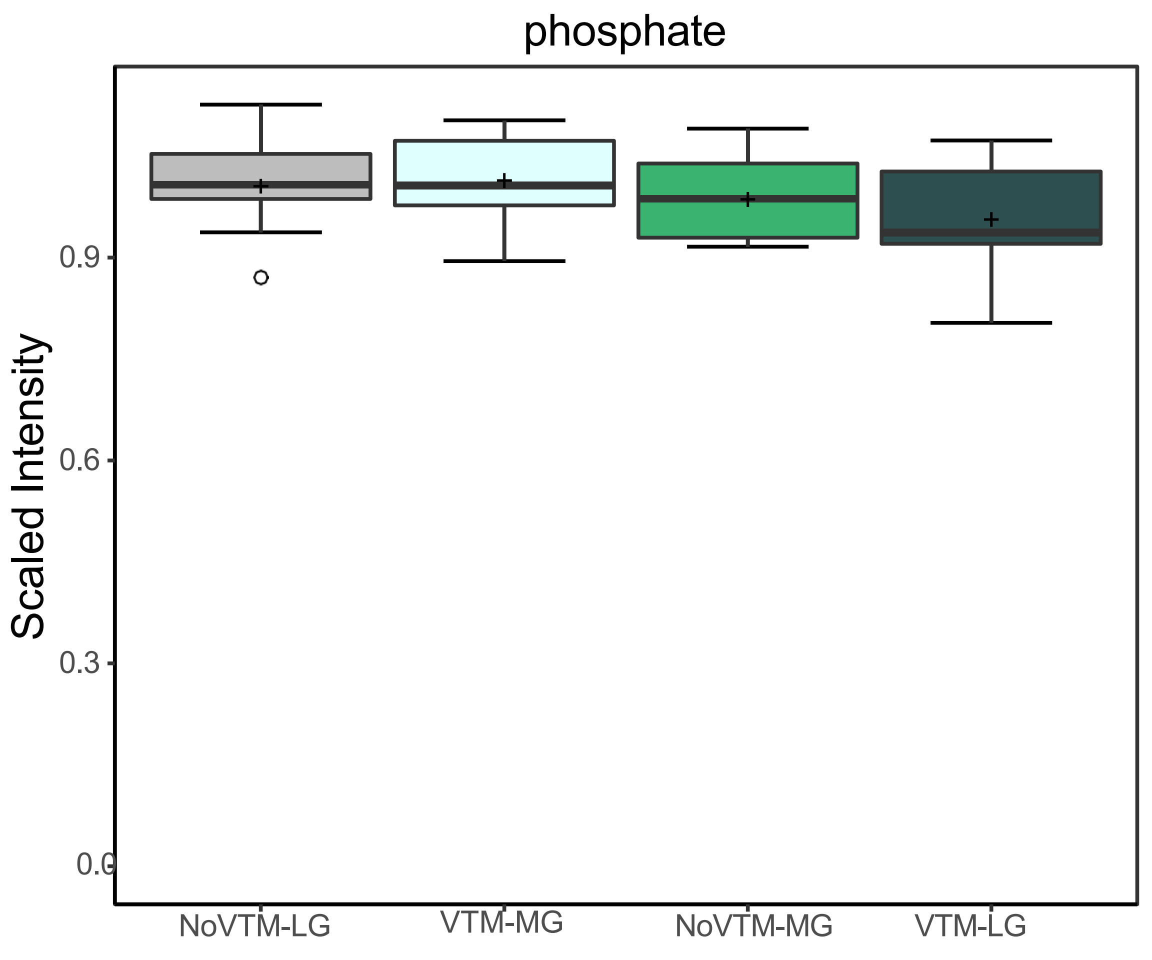

phosphate
Scaled Intensity
0.9
0.6
0.3
0.0
NoVTM-LG
VTM-MG
NoVTM-MG
VTM-LG
